# Advancing Stable Isotope Analysis with Orbitrap-MS for Fatty Acid Methyl Esters and Complex Lipid Matrices

**DOI:** 10.1021/jasms.5c00092

**Published:** 2025-06-17

**Authors:** Gabriel F. dos Santos, Giovanni B. Bevilaqua, Alexis Gilbert, Hugo G. Machado, Maxime Julien, Gesiane S. Lima, Nerilson M. Lima, Júlio C. O. Ribeiro, Alexandre A. Ferreira, Ygor S. Rocha, Boniek Gontijo

**Affiliations:** † Chemistry Institute, 67824 Federal University of Goiás, Goiânia, Goiás 74690-900, Brazil; ‡ Institute for Marine and Atmospheric Research Utrecht (IMAU), Utrecht University, 3526 kV Utrecht, The Netherlands; § Université de Nantes, CNRS, CEISAM UMR 6230, F-44000 Nantes, France; ∥ Institute of Chemistry, Federal University of Alfenas, Alfenas, Minas Gerais 37130-001, Brazil; ⊥ Division of Geochemistry, PETROBRAS Research and Development Center (CENPES), PETROBRAS, Rua Horácio Macedo, Ilha do Fundão, Rio de Janeiro 21941-915, Brazil

## Abstract

Isotopic analysis plays a crucial role in different scientific fields, offering valuable insights that aid in elucidating biosynthetic pathways, determining geographic origin, and identifying product adulteration. Established mass spectrometry techniques for isotopic analysis require the conversion of samples into gases prior to introduction into the systems. Moreover, the ionization process in these methods is destructive, potentially leading to the loss of essential molecular structure information. Thus, alternative analytical methods, such as Orbitrap-MS, could be a useful tool to determine stable isotope ratios. This paper describes an Orbitrap-based method using stearic acid methyl ester as a model molecule to determine the stable isotopic ratios of fatty acids and fatty acid methyl esters (FAMEs) in different vegetable butters. Orbitrap analyses were performed in positive ionization mode with both [M + H]^+^ and [M + Na]^+^ ions considered for the analysis. Nine standards (Std 1-Std 9) and three vegetable butters (cupuaçu, cocoa, and shea) were employed in the study. The standards were employed to develop the method and were measured using HPLC and a dual-inlet system. Both injections achieved high precision (<1.5‰) when compared with the IRMS data; however, the HPLC showed the most accuracy and was selected for direct injection measurement of the natural samples. Our results demonstrated the efficiency of the ESI-Orbitrap system in differentiating sources based on δ^13^C values. This study not only advances the use of high-resolution mass spectrometry for isotope analysis but also opens new avenues for applying stable isotopes in food sciences.

## Graphical abstract

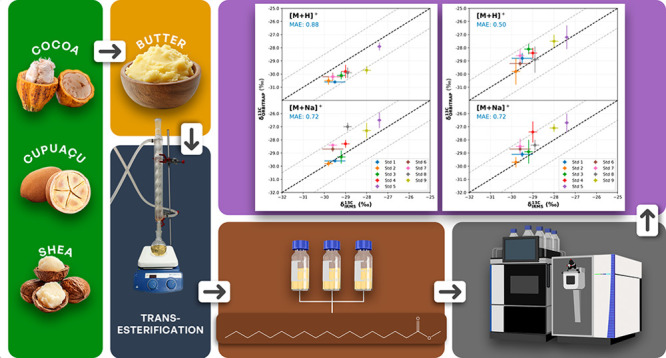

## Introduction

Stable isotope variations provide a unique and unparalleled tool for understanding natural processes, offering a bridge between atomic-level interactions and their larger-scale effects. These variations play a critical role across various scientific disciplines, including environmental chemistry,^1,2^ geochemistry,^3–6^ forensics,^7,8^ medicine and biochemistry,^9,10^ anthropology,^11^ and fundamental chemistry.^12–15^ The separation of isotopologues results in distinct distributions within natural or synthetic materials. These distributions, shaped by the compound’s synthesis, storage, and degradation history, provide valuable insights. The predictable fractionations caused by specific chemical and physical processes make isotopic composition a powerful tool for unraveling the history of compounds.^14,16^

Conventional methods for isotope ratio determination, such as isotope ratio mass spectrometry (IRMS), rely on magnetic sector mass spectrometers. However, this technique typically requires the analyte to be converted into simple molecular gases such as CO_2_, H_2_, N_2_, O_2_, and SO_2_ for the isotope ratio measurement.^14,17,18^ Thus, the resulting isotopic ratio represents the average for a given element over the entire molecule or sample.^15^ This approach limits the ability to examine isotopic variations at specific molecular positions or measure multiply substituted (“clumped”) isotope species, which could enhance current interpretations or open up new applications.

Recent advancements in isotopic ratio analysis using high-resolution mass spectrometers, such as Orbitrap-type systems coupled with electrospray ionization (ESI) source, have significantly expanded the diversity of molecules that can be analyzed. Orbitrap analyzers can reach resolving powers of up to 480,000 at *m*/*z* 200, depending on the transient acquisition time, making it possible to distinguish isobaric species that differ by only a few millidaltons.^19^ These advanced instruments offer exceptional mass accuracy and precision, resolving numerous isobaric species in compounds containing elements like H, C, N, O, and S.

A growing body of research has focused on developing and evaluating the Orbitrap system for isotopic ratio measurements of polar compounds. Prominent works by Eiler et al. (2017),^15^ Hofmann et al. (2020),^14^ Neubauer et al. (2020),^20^ Hilkert et al. (2021),^13^ Mueller et al. (2022),^18^ and Weiss et al. (2023)^21^ have demonstrated the capabilities of Orbitrap analyzers for high-precision isotopic measurements. These studies have primarily explored small compounds such as acetate,^18^ nitrate,^20^ phosphate,^22^ and amino acids.^14,21^ Recently, Kantnerová et al. (2024)^19^ published a protocol as a guide for isotope measurements using Orbitrap MS

Although Orbitrap MS has proven effective for analyzing smaller polar compounds, its application to larger, structurally diverse molecules such as fatty acids and FAMEs remains underexplored. Addressing this research gap could yield valuable insights given the pivotal roles of fatty acids in biological systems, including their significance in health, metabolism, and energy storage.^23–25^ For instance, isotopic analysis of fatty acids could provide crucial information about their biosynthetic pathways, distinguish dietary and metabolic sources, and elucidate their functions in various physiological and pathological contexts.

The absence of studies applying Orbitrap MS to fatty acid isotopic analysis presents an opportunity to extend its utility to larger, more complex biomolecules. Advancing this methodology could benefit various disciplines, from biochemistry to nutrition and environmental science. This study aims to bridge this gap by introducing a method to measure the ^13^C isotopic ratios of fatty acids in food products using ESI-Orbitrap MS. Stearic acid methyl ester was chosen as a proof of concept due to its importance and relevance as a representative fatty acid.^26^

## Materials and Methods

### Preparation of ^13^C-Enriched Stearic Acid Methyl Ester Standards

Natural abundance stearic acid (CAS: 57-11-4) was purchased from Sigma-Aldrich (Product number: S4751). Stearic acid-1-^13^C (CAS: 85541-42-0) was purchased from Sigma Alrdrich (Product number: 299162). ^13^C-enriched stearic acid methyl esters were prepared by spiking a natural abundance stearic acid with ^13^C-labeled stearic acid. The mixture was then dissolved in CHCl_3_ and diluted with a solution of natural abundance stearic acid to obtained different ^13^C-enriched samples. Each sample was then individually methylated following the method of Julien et al. (2022).^27^

The ^13^C isotopic composition of methyl stearate samples were measured at the Tokyo Institute of Technology (Japan) as described in Julien et al. (2022). Briefly, the samples were injected into a gas chromatography (GC) coupled to an isotope ratio mass spectrometer (DeltaPlusXP, Thermo Fisher Scientific) via a combustion furnace. High purity helium (>99.99%) was used as the carrier gas. Samples were diluted in hexane before injection in the GC. The GC was equipped with a capillary column (DB-5, 30 m × 0.32 mm i.d., 0.25 μm film thickness; Agilent J&W) and the injection was made using a 10 μL syringe. The chromatographic conditions were as follows: injector temperature 250 °C; split ratio 10:1; flow rate at 1.5 mL/min; initial oven temperature was 50 °C maintained for 5 min then raised to 250 °C at 10 °C/min and maintained for 10 min. The combustion furnace operated at 960 °C and contained a ceramic tube packed with CuO, NiO and Pt wires. The generated CO_2_ was then analyzed in the IRMS. The isotopic standardization was made using working standards described in Julien et al. (2022).^27^

### HPLC and MS Instrumentation

A Vanquish Neo UHPLC system (Thermo Fisher Scientific) was coupled with an Orbitrap Exploris 240 Mass Spectrometer (Thermo Fisher Scientific) equipped with an OptaMax NG ion source featuring an API inlet. The ion source was further configured with a heated electrospray ionization (HESI-II) probe (Thermo Fisher Scientific).

The ionization settings for positive ionization mode were as follows: sheath gas flow rate of 2, auxiliary gas flow rate of 2, sweep gas flow rate of 0, spray voltage of 3.4 kV, capillary temperature of 300 °C, and S-lens RF level set to 70. The mass spectrometer was operated in Full Scan mode with a scan range of *m*/*z* 298–302 ([M + H]^+^) and 320–325 ([M + Na]^+^) with a resolution of 60,000. The acquisition utilized two microscans per scan, an automatic gain control (AGC) target of 5e5, and a maximum injection time of 100 ms. Data acquisition was performed using Xcalibur software (Thermo Fisher Scientific).

### Isotope Analysis by ESI-Orbitrap-MS

#### HPLC Injection Mode

The Vanquish Neo System, configured with nanoflow settings, was coupled with an Orbitrap Exploris 240 for injection of the FAMEs solutions. The HPLC was operated without a chromatographic column, functioning as an autosampler for direct flow injection analysis. A flow rate of 5 μL min^–1^ carried the samples in LC/MS-grade methanol. The HPLC was equipped with a 100 μL injection loop, from which 50 μL was injected, producing a broad plateau peak over 10 min. To minimize contamination, the total analysis time was set to 15 min. Sequences were arranged in alternating blocks, with one injection per reference and sample.

#### Dual Inlet Mode

In order to achieve the continuous alternating delivery of a reference and a sample, we employed a digitally controlled syringe pump (Fusion 100, Chemyx) equipped with two 500 μL Hamilton syringes. The pump was connected to a 6-port valve (Rheodyne) using a short length of 0.5 μm peek, ensuring minimal dead volume and consistent flow. After an initial system conditioning period of a least 30 min to stabilize the flow and ensure operational consistency, reference/sample comparisons were conducted over 35 min period. The 6-port valve was alternated every 5 min, ensuring a precise and reproducible flow pattern throughout the experiment. This approach resulted in a bracketed experimental design consisting of seven alternating events: reference and sample. Specifically, the sequence included three replicate sample injections and four replicate reference injections, providing a robust framework for accurate comparison and minimizing variability.

### Data Processing

The extraction and processing of ion signals were performed using IsotoPy, an in-house software developed by our research group at Federal University of Goiás (available at http://www.isotopy.com.br or in the reference article). IsotoPy is written in Python 3 and utilizes the RawFileReader library provided by Thermo Fisher Scientific (available at github.com/thermofisherlsms/RawFileReader). Although this library is written in C# (.NET), it was successfully imported and integrated into the Python environment to enable high-performance access to proprietary.RAW files.

IsotoPy enables the extraction of critical information, including isotopologue distribution, scan data, peak intensities, peak noise levels, and other parameters essential for the calculation of isotope ratios and δ-values. The ion count was calculated using eq 1, the same used by Thermo Fisher’s IsoX software, as described in the recent publication by Kantnerová et al. (2024)^19^

1 $$\mathrm{ion count}=\frac{S}{N}\times 3\times \sqrt{\frac{\mathrm{Rn}}{r}}\times \sqrt{\mu }$$

where *S* is the ion intensity, *N* is the peak noise, the constant 3 is a rounded approximation of the number of charges corresponding to the noise at the resolution settings used, which has been experimentally determined,^15,28^ Rn is the reference resolution of 240,000 at which the constant 3 was determined,^19^ *r* is the mass resolution, and μ is the number of microscans.

The isotopic ratio is defined as the ratio of ion counts, and since the experimental constants, mass resolution and number of microscans are identical for both isotopologues in our setup, the final calculation ultimately depends only on the signal intensity and peak noise, as shown in eq 2 for ^13^C.

2 $$\mathrm{isotopic ratio}=\frac{S_{13C}/N_{13C}}{S_{12C}/N_{12C}}$$

The IsotoPy software not only extracts relevant data but also performs automated postprocessing steps, including outlier scan detection, statistical filtering, and the calculation of δ^13^C values referenced to international standards. This integrated pipeline provides a robust and precise solution for Orbitrap-based isotope data analysis and was essential to the present study (see Supporting Information for additional details). In this paper, IsotoPy was specifically used to calculate both ^13^C ratio and δ values, offering a robust and precise solution for isotope data analysis.

### Calculation of δ-Values

The delta (δ) value is defined as δ_sample/STD_ = [(R_sample_/R_STD_) – 1] × 1000, where sample represents a sample/analyte, STD represents the primary reference standard, and R is the isotope abundance ratio (^13^C/^12^C, ^2^H/^1^H, ^18^O/^16^O, and ^17^O/^16^O). δ^13^C values were calculated and reported relative to the standard used in the analysis. However, for the best comprehension, it is necessary to express the δ^13^C relative to the Vienna Pee Dee Belemnite (VPDB). These values were then expressed relative to the δ^13^C_VPDB_ scale using the value of δ^13^C_std/VPDB_ as shown in equation δ^13^C_sample/VPDB_ = δ^13^C_sample/std_ + δ^13^C_std/VPDB_ + [(δ^13^C_sample/std_. δ^13^C_std/VPDB_)/1000].^13,18^

### Vegetable Butter Samples

Three vegetable butters—shea, cupuaçu, and cocoa—were analyzed using this methodology to determine the δ^13^C_VPDB_ value of stearic acid methyl ester. These butters, sourced from the local market, were specifically chosen due to their high stearic acid content, ensuring a robust assessment of the method’s applicability to natural lipid samples. Prior to analysis, all vegetable butters were submitted to a transesterification process. approximately 50 mg of butter were individually transferred to a 125 mL round-bottom flask, and 60 mL of methanol and 1 mL of sulfuric acid were added. The solution was heated in an oil bath at 75 °C under reflux for 18 h. After this step, the solution was washed vigorously with 120 mL of sodium bicarbonate at 0.35 mol L^–1^ and 60 mL of ethyl acetate. The ethyl acetate phase was then separated using a separation funnel. The collected organic phase was dried with sodium sulfate and subjected to a rotary evaporator until all solvent was completely removed. Finally, the FAMEs were kept at −20 °C until further analysis by HPLC-ESI-Orbitrap.

### GC-MS Analysis of Vegetable Butters

Following transesterification, all vegetable butter were analyzed by a gas chromatography instrument coupled to a triple quadrupole analyzer (GC-MS, TSQ 9610 ThermoFisher) for composition characterization. A TG – 5SilMS capillary column with a dimension of 0,25 mm x 30m; 0,25 μm (Thermo Scientific, Waltham, USA) was used for the separation of FAMEs. The initial temperature was set to 150 °C and held for 2 min before being increased to 230 °C at a rate of 4 °C per minute, where it was held for 5 min. A split ratio of 1:50 was used, with helium as the carrier gas at a flow rate of 0.8 mL min-1. The injector and detector temperatures were maintained at 240 and 260 °C, respectively. The mass spectrometer operated with an electron impact (EI) ionization source at 70 eV.

## Results and Discussion

### Isotopologue Detection and Analytical Validation

The isotopic analysis of organic compounds is a cornerstone in chemical and biological research. It enables the tracing of origins, authentication of products, and insights into metabolic pathways.^29–31^ This study focuses on the isotopic determination of stearic acid methyl ester (C_19_H_38_O_2_), which was selected as a model compound due to its isotopic complexity and relevance to food science, environmental analysis, and biochemical research.^32,33^ Its isotopic profile includes several key isotopologues: the monoisotopic peak (M_0_, 80.18%), singly substituted ^13^C (5.35%), doubly substituted ^13^C_2_ (0.15%), and ^18^O (0.33%). These relative abundances align with theoretical predictions, establishing a realistic and robust framework for evaluating advanced analytical techniques.

The Orbitrap MS instrument has proven to be a powerful tool for isotopic measurements.^13,18,20,29^ The soft ionization achieved in this analysis enables the simultaneous observation of a wide range of isotopologues. The detection of isotopologues such as ^13^C and ^13^C_2_, even at natural abundances as low as 0.15%, underscores its sensitivity and specificity. This capability becomes especially significant when analyzing complex matrices, where isotopic fractionation or trace-level isotopologues can critically influence outcomes. Figure 1 illustrates these capabilities, with Figure 1a showing a stable Total Ion Chromatogram (TIC) from an HPLC-ESI-Orbitrap MS analysis for a 15 min run, and Figure 1b,c highlighting the precise resolution of M_0_, M_1_, and M_2_ isotopologues for [M + H]^+^ and [M + Na]^+^ ions. The distinct isotopic peaks and enhanced intensity of the [M + Na]^+^ ion, approximately 10-fold greater than [M + H]^+^ (Supporting Information, Figure S1), emphasize the importance of exploring multiple ionization pathways for comprehensive analysis.

**Figure 1:**
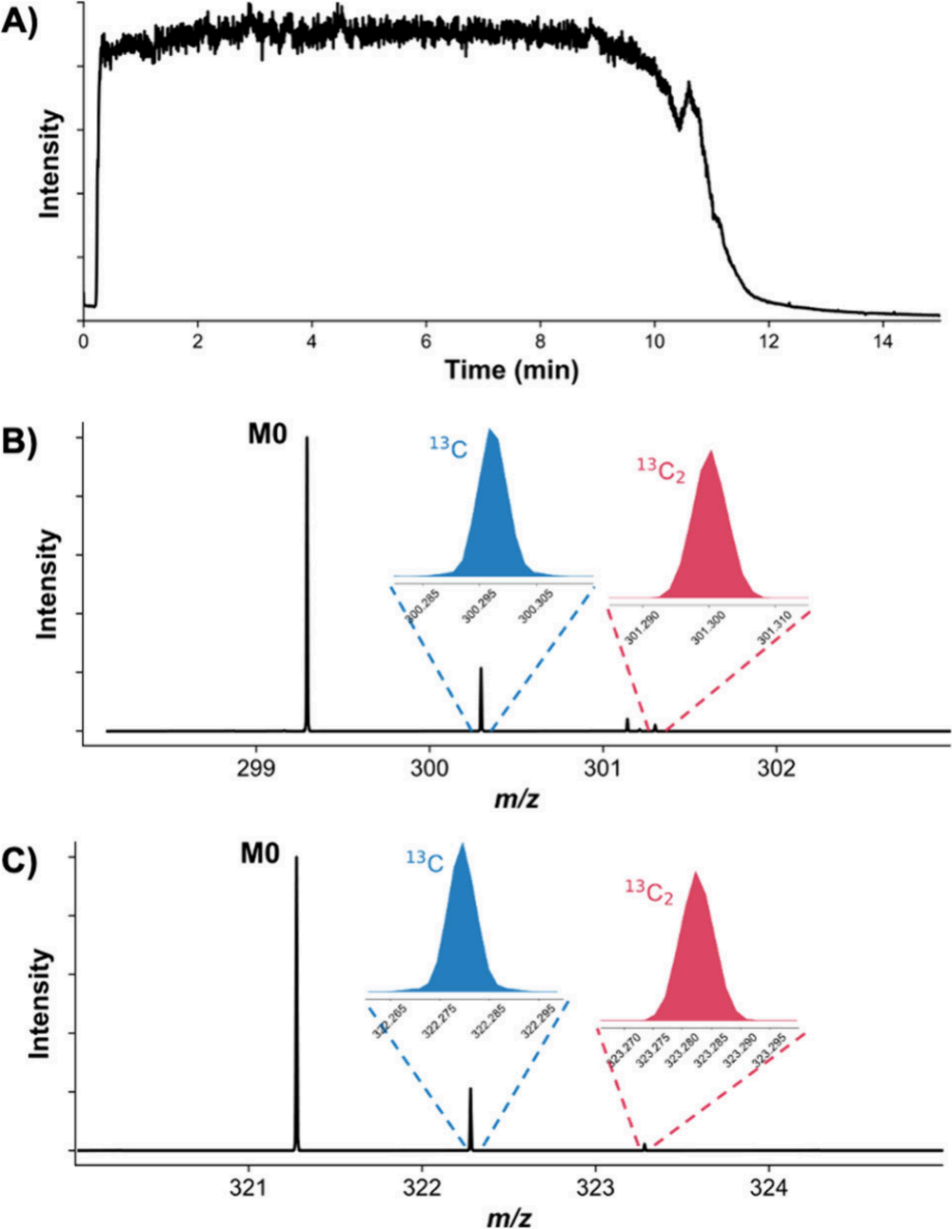
Chromatographic stability and isotopologue detection using ESI-Orbitrap MS for stearic acid methyl ester. (a) Total Ion Chromatogram (TIC) demonstrating excellent peak resolution and stability over a 15 min run by HPLC-ESI-Orbitrap MS. (b) SIM spectrum highlighting the detection of isotopologues M_0_, M_1_ (^13^C), and M_2_ (^13^C_2_) for the [M + H]^+^ ion. (c) SIM spectrum showing isotopologues for the [M + Na]^+^ ion, with enhanced signal intensity compared to [M + H]^+^, demonstrating the impact of sodium adduction.

To achieve comprehensive isotopic characterization, this study employed a dual-method approach integrating GC-C-IRMS^21^ and Electrospray Ionization-Orbitrap Mass Spectrometry (ESI-Orbitrap MS). This methodology builds upon prior research,^13,15,18–21,29^ which has demonstrated strong agreement between these techniques for smaller molecules, by extending the analysis to a larger and more complex compound: stearic acid methyl ester. Each method contributes unique strengths to the analysis. GC-C-IRMS, the gold standard for bulk isotopic measurements, provided precise δ^13^C values that served as a critical benchmark. In contrast, Orbitrap MS offered unparalleled resolution and sensitivity, enabling high-precision detection and quantification of individual isotopologues, including rare species like ^13^C_2_ and ^18^O, which are inaccessible through bulk analysis alone.

The δ^13^C values of stearic acid methyl ester standards were initially measured using GC-C-IRMS to obtain accurate reference values based on a well-established technique. This step ensured consistency across standards, providing precise and reproducible δ^13^C measurements (Table 1).

**Table 1:** Stearic Acid Methyl Ester Standards and Their Respective δ^13^C_VPDB_ Values

Name	δ^13^C_VPDB_ (‰)	SD
Reference	–27.8	0.4
Std 1	–29.5	0.5
Std 2	–29.8	0.2
Std 3	–29.2	0.2
Std 4	–29.0	0.2
Std 5	–27.4	0.1
Std 6	–29.6	0.5
Std 7	–29.6	0.2
Std 8	–28.9	0.2
Std 9	–28.5	0.2

This study primarily aimed to assess the reliability of Orbitrap MS for bulk isotope composition measurements of fatty acids. While GC-C-IRMS provides highly accurate δ^13^C values, it lacks the mass resolution to resolve molecular isotopologues. Conversely, Orbitrap MS offers high-resolution mass spectrometry with the potential to characterize intramolecular isotopic distributions, but its application for isotope ratio measurements requires validation. Here, we compared the δ^13^C values obtained using Orbitrap MS with those measured by GC-C-IRMS.

It is important to clarify that GC-C-IRMS was used solely for calibration purposes, as it provides bulk isotope composition rather than position-specific or clumped isotopologue measurements. The combination of GC-C-IRMS and Orbitrap MS was not intended to merge the advantages of both techniques but rather to ensure proper calibration of the Orbitrap for bulk isotope measurements. Therefore, our results focus on evaluating the Orbitrap’s capability to accurately measure δ^13^C values in methyl stearate, highlighting its potential for isotopic characterization in natural samples

### Ionization Pathways and Analytical Sensitivity

The ionization pathways observed in Orbitrap MS provide valuable insights into the analytical sensitivity and isotopic accuracy achieved in this study. Stearic acid methyl ester displayed two dominant ionization forms: [M + H]^+^ and [M + Na]^+^ (Supporting Information, Figure S1). While [M + H]^+^ is the standard ionization pathway in electrospray ionization (ESI), the [M + Na]^+^ ion demonstrated a significantly higher signal intensity, approximately 10-fold greater. Even across a range of tuning parameters, the sensitivity for [M + Na]^+^ remained consistent, maintaining its superior signal intensity compared to [M + H]^+^.

This enhancement is likely attributed to residual sodium introduced during the synthesis of fatty acid methyl esters (FAMEs), which typically involves alkali metal bases. Sodium adduction not only stabilizes the molecular ion but also improves ionization efficiency, resulting in a stronger and more consistent signal.

These two ionization pathways raise concerns about potential isotopic fractionation, as the analyte population could be split between [M + H]^+^ and [M + Na]^+^ forms. However, the results shown in Figure 2 from Dual Inlet and HPLC ESI-Orbitrap MS analysis demonstrate that fractionation was minimal. Figure 2 presents the δ^13^C values obtained for both pathways across all standards, highlighting their strong agreement with GC-C-IRMS measurements. Deviations consistently remained below 1.5‰ for most samples.

**Figure 2:**
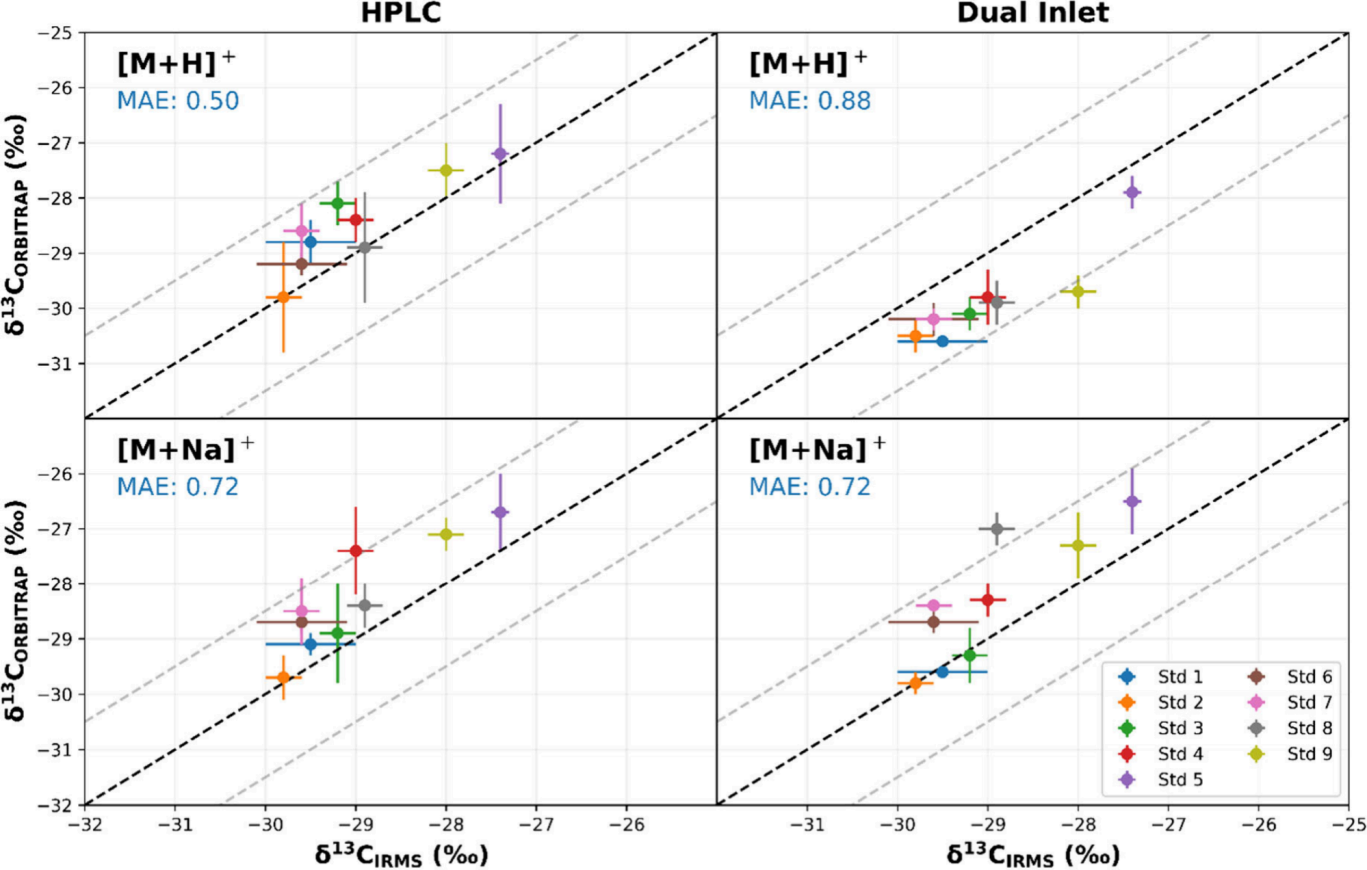
Comparison of δ^13^C values for stearic acid methyl ester standards obtained using Dual Inlet and HPLC ESI-Orbitrap MS. δ^13^C values for [M + H]^+^ and [M + Na]^+^ ions, showing deviations from the GC-C-IRMS measurements. Error bars represent standard deviations across replicates. Additionally, a summary of MAEs and average deviations for each ionization pathway are shown.

To quantify the analytical accuracy, the Mean Absolute Error (MAE) was calculated as shown in eq 3:

3 $$\mathrm{MAE}=\frac{1}{n}\overset{n}{\underset{i=1}{\sum }}\left|\delta^{13}C_{\mathrm{Orbitrap},i}-\delta^{13}C_{\mathrm{IRMS},i}\right|$$

where *n* is the number of standard compounds analyzed, δ^13^C_Orbitrap*,i*_ is the value obtained by Orbitrap MS, and δ^13^C_IRMS*,i*_ is the corresponding GC-C-IRMS value. This metric summarizes the average magnitude of deviation without regard to direction.

The MAE for each for each ionization pathway is also presented in Figure 2. The slightly lower MAE observed for [M + Na]^+^ ions by Dual Inlet ESI-Orbitrap MS suggests that their enhanced signal intensity and stability contribute to enhanced analytical precision.

The measurement using a Dual Inlet system consists of the sequential delivery of samples by direct infusion into the ionization source via a dual syringe pump. In this method, 50 μM stearic acid methyl ester standards were analyzed by alternating 5 min infusions (35 min total) of the sample and reference, totaling seven blocks (Blocks 1, 3, 5, and 7 for the isotopic reference, and Blocks 2, 4, and 6 for the samples). Although the Dual Inlet method has some limitations compared to HPLC-based sample introduction, it is highly precise for analyzing different compounds.^13,18,19^

By alternating between standards and samples in a controlled manner, the system minimizes potential isotopic fractionation and ensures consistent ionization conditions. This stability is reflected in the results shown in Figure 2, where both [M + H]^+^ and [M + Na]^+^ pathways provided comparable isotopic ratios, confirming the reliability of the Orbitrap MS platform even under dual-pathway detection conditions.

In addition to the Dual Inlet ESI-Orbitrap MS measurements, HPLC-ESI-Orbitrap analysis was performed for all stearic acid methyl ester standards. Figure 2 illustrates this complementarity approach, showing the strong agreement between δ^13^C values obtained using both methods for the [M + H]^+^ and [M + Na]^+^ isotopologues across all standards. Deviations remained consistently below 1.5‰ for most samples, with [M + Na]^+^ isotopologues showing slightly lower MAE due to enhanced ionization efficiency. This alignment reinforces the robustness of the dual-method strategy (DI-ESI-Orbitrap and HPLC-ESI-Orbitrap), even when applied to larger molecules with complex isotopic distributions.

The methodological framework presented in this study exemplifies a significant advancement in isotopic analysis by extending the application of Orbitrap MS to larger and more complex molecules, such as stearic acid methyl ester. While IRMS remains the gold standard for accurate bulk isotopic measurements, the study focuses on Orbitrap MS’s high-resolution capabilities for resolving molecular isotopologues with unprecedented detail, including clumped isotopes such as ^13^C_2_, ^13^C^15^N, and ^13^C^18^O. Validating Orbitrap MS data against GC-C-IRMS ensures the reliability of this approach and highlights its potential for bridging the gap between bulk and individual molecule isotopic analyses.

This framework highlights how Orbitrap MS, with its high sensitivity and precision in measuring bulk isotope composition, enhances the study of isotopic variations in complex compounds and matrices. This approach is particularly valuable for applications requiring accurate δ^13^C measurements, such as food authentication, environmental monitoring, and metabolic pathway studies, where high-resolution isotope analysis can provide critical insights.

By using stearic acid methyl ester as a model compound, this study underscores the robustness and versatility of Orbitrap MS in addressing challenges posed by larger molecules with intricate isotopic compositions. The findings lay a foundation for expanding isotopic research to other complex systems. The subsequent sections will delve into the technical performance of the methods used and explore their broader implications for advancing isotopic analysis.

Overall, the minimal fractionation observed in this study underscores the importance of controlling experimental parameters, such as solvent composition and sample delivery. Residual sodium from the synthesis process, while contributing to the [M + Na]^+^ pathway, ultimately enhanced the precision and sensitivity of isotopic measurements without introducing significant bias. The ability to measure isotopic ratios with high accuracy across DI-ESI and HPLC-ESI demonstrates the robustness of Orbitrap MS for isotopic studies in diverse fields, including food authentication, environmental monitoring, and metabolic pathway research.

### Methodological Comparison: DI-ESI vs HPLC-ESI Orbitrap MS

The performance of DI-ESI and HPLC-ESI in Orbitrap MS analysis was evaluated to understand their suitability for isotopic studies of stearic acid methyl ester. Both methods demonstrated high precision and reliable mass resolution of individual isotopologues.

DI-ESI employs a dual-syringe system for sample delivery, alternately introducing isotopic standards and analyte solutions directly into the ionization source. This method enables rapid analysis and is particularly effective for high-throughput workflows. However, as shown in Support Information, , DI-ESI exhibited occasional signal fluctuations during the 35 min analysis, reflecting limitations in stability over extended acquisition times. Additionally, the DI-ESI sample introduction is limited by syringe volume, as a 0.5–1 mL syringe does not allow for extended analyses. Despite this, DI-ESI achieved accurate δ^13^C measurements for both [M + H]^+^ and [M + Na]^+^ ions, with deviations consistently below 1.5‰ from the GC-C-IRMS measurement values, as demonstrated in Figure 2.

This study used HPLC-ESI as an automated injection system without chromatographic separation. The implementation of a 100 μL injection loop enhanced stability and reproducibility compared to DI-ESI. The automated nature of the HPLC injector enhances flow rate stability and reduces risks of fluctuations during the analysis. In addition, it allows for longer analysis times compared to the DI approach. The δ^13^C values obtained via HPLC-ESI demonstrated a slightly lower MAE than DI-ESI. For [M + H]^+^ ions, the MAE was 0.52‰ for HPLC-ESI versus 0.86‰ for DI-ESI, while for [M + Na]^+^ ions, the MAE was 0.71‰ and 0.72‰, respectively, as summarized in Table 2. These results underscore the ability of the automated injection system to enhance analytical accuracy.

**Table 2:** δ^13^C Values for both [M + H]^+^ and [M + Na]^+^ Ions Analyzed Using Dual Inlet and HPLC Injection and Their Residue Compared to the Expected Values (Obtained by GC-C-IRMS)

Stds^a^	δ^13^C_[M+H]_ ± SD	Residue	δ^13^C_[M+H]_ ± SD	Residue
Dual Inlet				
1	–30.6 ± 0.1	–1.1	–29.6 ± 0.1	–0.1
2	–30.5 ± 0.3	–0.7	–29.8 ± 0.2	0.0
3	–30.1 ± 0.3	–0.9	–29.3 ± 0.5	–0.1
4	–29.8 ± 0.5	–0.8	–28.3 ± 0.3	0.7
5	–27.9 ± 0.3	–0.5	–26.5 ± 0.6	0.9
6	–30.2 ± 0.3	–0.6	–28.7 ± 0.2	0.9
7	–30.2 ± 0.2	–0.6	–28.4 ± 0.1	1.2
8	–29.9 ± 0.4	–1.0	–27.0 ± 0.3	1.9
9	–29.7 ± 0.3	–1.7	–27.3 ± 0.6	0.7
HPLC				
1	–28.8 ± 0.4	0.7	–29.1 ± 0.2	0.4
2	–29.8 ± 1.0	0.0	–29.7 ± 0.4	0.1
3	–28.1 ± 0.4	1.1	–28.9 ± 0.9	0.3
4	–28.4 ± 0.4	0.6	–27.4 ± 0.8	1.6
5	–27.2 ± 0.9	0.2	–26.7 ± 0.7	0.7
6	–29.2 ± 0.2	0.4	–28.7 ± 0.2	0.9
7	–28.6 ± 0.5	1.0	–28.5 ± 0.6	1.1
8	–28.9 ± 1.0	0.0	–28.4 ± 0.4	0.5
9	–27.5 ± 0.5	0.5	–27.1 ± 0.3	0.9

a Stds = standards.

This result highlights the impact of sodium adduction on signal intensity and demonstrates that both injection methods can maintain consistent and accurate isotope ratio measurements. From a practical perspective, the choice between DI-ESI and HPLC-ESI depends on the analytical requirements:

- **DI-ESI** is highly effective for rapid screening and simpler matrices due to its direct and time-efficient workflow. It is especially suitable for applications that prioritize speed and require minimal sample preparation.
- **HPLC-ESI**, used in this study as an in-line injection system without chromatographic separation (i.e., as an autosampler), this setup offers greater reproducibility and slightly enhanced accuracy, making it particularly advantageous for studies that require consistent injections and minimal analytical variability.

In this study, both methods proved reliable for isotopic analysis (Table 2), even though the number of scans for DI-ESI was significantly lower due to syringe volume limitations. HPLC-ESI offered a slight advantage in terms of stability and reproducibility, whereas DI-ESI excelled in simplicity and operational efficiency. Selecting the appropriate injection method ensures optimal analytical performance, balancing speed, precision, and reproducibility to meet the specific demands of isotopic investigations.

### Application to Complex Mixtures: Vegetable butters

To demonstrate the practical applicability of the developed methodology, cocoa, shea, and cupuaçu butters were analyzed as representative complex matrices. These vegetable butters were chosen for their high stearic acid content and their relevance in food science and industrial applications. The analysis aimed to evaluate the robustness of the Orbitrap MS platform in handling complex samples while enabling isotopic differentiation among the butters. The butters were subjected to a transesterification process to convert triglycerides into fatty acid methyl esters (FAMEs), enabling the efficient ionization of stearic acid methyl ester for isotopic analysis. This derivatization step was essential for ensuring compatibility with the Orbitrap MS method. The transesterification process was highly effective, yielding methyl stearate percentages of 36.5% for shea butter, 36.4% for cocoa butter, and 42.3% for cupuaçu butter. The success of the transesterification was confirmed through GC-MS, which detected the predominant formation of FAMEs in the *m*/*z* range of 250–350. In addition to stearic acid methyl ester, other FAMEs were identified, such as palmitic acid methyl ester, oleic acid methyl ester (most abundant in cupuaçu butter), and linoleic acid methyl ester (Supporting Information, Table S1). Furthermore, the Orbitrap MS spectra of the samples showed well-defined and distinguishable peaks corresponding to the expected isotopologues, demonstrating the method’s high mass accuracy and the robustness of the preparatory workflow for complex lipid matrices.

The HPLC-ESI-Orbitrap MS method was used to measure δ^13^C values for stearic acid methyl ester derived from each vegetable butter sample. Although HPLC-ESI-Orbitrap MS exhibited good reproducibility, full scan acquisitions revealed the presence of additional ions within the mass window used to monitor the target isotopologues in butter samples. These signals, although mass-resolved, were not initially recognized as potentially impactful. As later discussed by Mueller et al. (2023),^34^ such coisolated species can still influence the ionization efficiency of analytes, potentially affecting the apparent isotope ratios. In this study, contaminant signal contribution remained below 10% in most cases; however, in a few instances, values slightly exceeded this threshold. To address this, each individual sample was analyzed in triplicate across three separate bracketed runs (7 blocks each) to ensure statistical robustness. Examples of analytical blocks with the highest contamination levels for each butter type are shown in Figure S3 (Supporting Information).

The results, presented in Figure 3, revealed distinct isotopic profiles for the three butters despite their similar fatty acid compositions. Statistical analysis confirmed significant differences between cupuaçu and both cocoa and shea butters (*p* < 0.05, 95% confidence), while no significant difference was observed between cocoa and shea (p = 0.24). These findings further demonstrate the method’s capacity to distinguish subtle variations in isotopic signatures among closely related plant-derived products.

**Figure 3:**
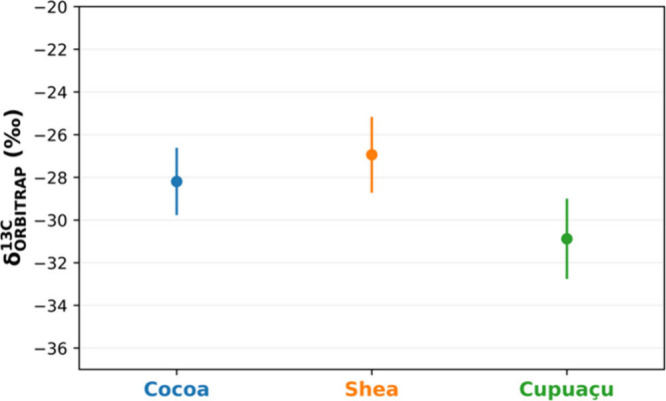
δ^13^C values for stearic acid methyl ester derived from cocoa, shea, and cupuaçu butters measured using HPLC-ESI-Orbitrap MS. The isotopic differentiation among the butters reflects variations in growing conditions and processing methods, consistent with their C3 photosynthetic origins. Error bars indicate the 95% confidence interval calculated from nine δ^13^C values obtained across three independent bracketed analyses per sample.

The δ^13^C values for stearic acid in cocoa butter reported in the literature, based on a single study, range from −31.2‰ to −39.8‰.^35^ Although the values obtained in our analysis were slightly more positive, they remain within the expected range for C3 photosynthetic pathway typical of the source plants and are consistent with previously reported values for cacao powder (δ^13^C −20.5‰ to −29.1‰).^36,37^ The slight variations observed among the butters reflect differences in geographic origin, environmental conditions, and processing methods, highlighting the method’s sensitivity in detecting subtle isotopic shifts.^35^

The isotopic differentiation highlights the method’s utility for food authentication and origin tracing. For instance, the δ^13^C signatures observed for cupuaçu butter (−30.9 ‰), a less-studied fat, allow its statistical distinction from cocoa and shea butters, which it is often used to replace in food formulations.

These findings highlight the potential of Orbitrap MS to expand its applications into new directions, particularly for analyzing complex lipid-rich matrices such as edible oils and processed food products. The precision and reproducibility of δ^13^C measurements provide a solid foundation for applications beyond food science. These include tracing natural products’ geographic origins, verifying high-value goods’ authenticity, and investigating metabolic pathways in biological systems. The seamless integration of Orbitrap MS with complementary techniques, such as GC-MS for verifying transesterification, enhances the reliability and comprehensiveness of the analytical process, establishing it as a valuable tool for multifaceted investigations.

The ability to differentiate vegetable butters based on their isotopic signatures underscores the versatility and robustness of Orbitrap MS in addressing challenges associated with the isotopic analysis of complex matrices. By successfully transitioning from controlled laboratory settings to real-world applications, the method demonstrates its ability to bridge the gap between experimental research and practical implementation. This advancement reinforces its role in traditional applications and opens avenues for innovative exploration across scientific, industrial, and environmental domains, establishing Orbitrap MS as a pivotal technology for future isotopic studies.

## Conclusion

In this study, we successfully developed an accurate method for analyzing stable isotope ratios of fatty acid methyl esters (FAMEs) using ESI-Orbitrap mass spectrometry. Our approach demonstrated strong potential for isotope analysis in complex lipid matrices and real samples by targeting stearic acid methyl ester as a model compound. The method’s sensitivity and reproducibility emphasize its applicability for isotopic analyses in various fields, including food authenticity and environmental studies, and it holds notable promise for assessing isotopic fingerprints in natural products and tracing metabolic pathways. The analysis of vegetable butters, such as cocoa, shea, and cupuaçu, demonstrated the ESI-Orbitrap system’s capability to differentiate sources based on δ^13^C values, with results consistent with conventional IRMS. Moreover, this methodology’s ability to efficiently handle natural product matrices and high precision indicates its potential for broader applications where isotopic composition is a marker for origin and production processes. Although promising, further studies are warranted to better understand and quantify potential matrix effects on apparent isotope ratios, particularly in complex sample types. Additionally, future studies should explore further refinements of this method across other lipid and fatty acid matrices and consider extending this analytical approach to other compounds of environmental and biochemical interest. This study advances the application of high-resolution mass spectrometry for isotope analysis and establishes a foundation for new insights into stable isotope applications in environmental, biological, and food sciences.

## Supplementary Material





## Notes

Comparison between [M + H]^+^ and [M + Na]^+^ ionization pathways for stearic acid methyl ester using ESI-Orbitrap MS and HPLC ESI Orbitrap, TIC obtained from the analysis of stearic acid methyl ester at 50 μM using Dual Inlet ESI(−)-Orbitrap MS, representative averaged full scan mass spectra from the analysis blocks of cocoa, cupuaçu, and shea butter samples, GC-MS analysis of cocoa, shea, and cupuaçu butter after FAMEs derivatizations, and supplementary text providing detailed information on data processing procedure (PDF)
Individual PDF reports for all standard and butter sample analyses (ZIP)
