# Supplementary material for: Advancing Stable Isotope Analysis with Orbitrap-MS for Fatty Acid Methyl Esters and Complex Lipid Matrices
Source: J Am Soc Mass Spectrom. 2025 Jun 17;36(7):1527–35. doi: 10.1021/jasms.5c00092 (PMC12339014; doi:10.1021/jasms.5c00092)
Supplement: Supplementary file 1 [file js5c00092_si_001.pdf]

Supporting Information

Advancing Stable Isotope Analysis with Orbitrap-MS for Fatty Acid Methyl Esters and Complex Lipid Matrices

Gabriel F. dos Santos, [a*] Giovanni B. Bevilaqua, [a] Alexis Gilbert, [b] Hugo G. Machado, [a] Maxime Julien, [c] Gesiane S. Lima, [a] Nerilson M. Lima, [a,d] Júlio C. O. Ribeiro, [a] Alexandre A. Ferreira, [e] Ygor S. Rocha, [e] Boniek G. Vaz [a*]

[a] Chemistry Institute, Federal University of Goiás, Goiânia, Goiás,74690-900, Brazil

[b] Earth-Life Science Institute, Tokyo Institute of Technology, Meguro, 152-8550, Tokyo, Japan.

[c] Université de Nantes, CNRS, CEISAM UMR 6230, F-44000 Nantes, France.

[d] Institute of Chemistry, Federal University of Alfenas, Alfenas, MG 37130-001, Brazil

[e] Division of Geochemistry, PETROBRAS Research and Development Center (CENPES), PETROBRAS, Rua Horácio Macedo, Ilha do Fundão, Rio de Janeiro, RJ 21941-915, Brazil

*Corresponding author.
E-mail address: gfs.dossantos@gmail.com (G. F. dos Santos)
E-mail address: boniek@ufg.br (B. G. Vaz)

Table of contents:

Figure S1. Isotopologue resolution comparison between $[M+H]^+$ and $[M+Na]^+$ ionization pathways for stearic acid methyl ester using ESI-Orbitrap MS and HPLC ESI Orbitrap._The mass spectrum for M_0, M_1 (^{13}C), and M_2 ($^{13}C_2$) isotopologues illustrate sharper peaks and higher intensity for the $[M+Na]^+$ pathway, highlighting its superior isotopic resolution and sensitivity……………………………………………………………………………...**S2**

Figure S2. The TIC was obtained from the analysis of stearic acid methyl ester at 50 µM using Dual Inlet ESI(-)-Orbitrap MS. The analysis involved alternating 5-minute infusions between reference and sample, yielding a total runtime of 35 minutes…………………………………**S2**

Figure S3. Representative averaged full scan mass spectra from the analysis blocks of cocoa, cupuaçu and shea butter samples that exhibited the highest apparent contamination levels within the analytical mass window…………………………………………………………………**S3**

Table S1. GC-MS analysis of Cocoa, Shea, and Cupuaçu butter after FAMEs derivatizations……………………………………………………………….………………....**S3**

Supplementary Text. Data Processing Details...…………………………………………….....**S4**

References……………………………………………………………………………………...**S5**

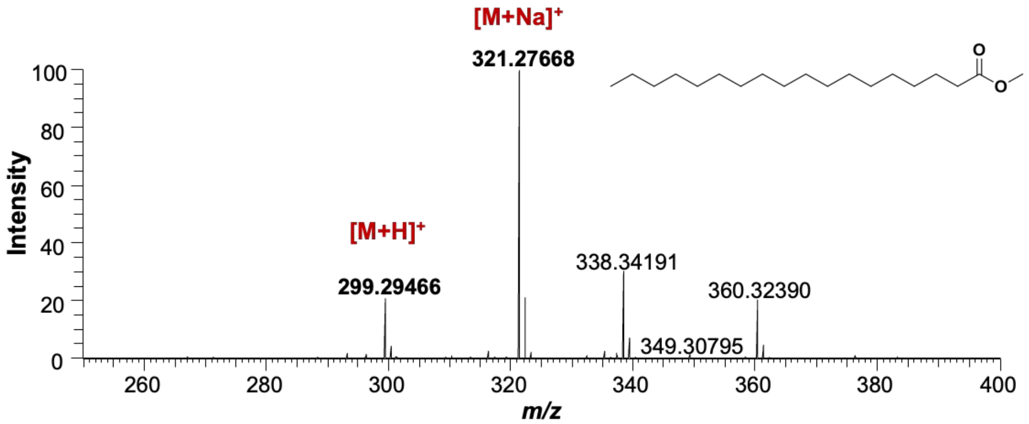

Figure S1. Isotopologue resolution comparison between $[M+H]^+$ and $[M+Na]^+$ ionization pathways for stearic acid methyl ester using ESI-Orbitrap MS and HPLC ESI Orbitrap._The mass spectrum for M_0, M_1 (^{13}C), and M_2 ($^{13}C_2$) isotopologues illustrate sharper peaks and higher intensity for the $[M+Na]^+$ pathway, highlighting its superior isotopic resolution and sensitivity.

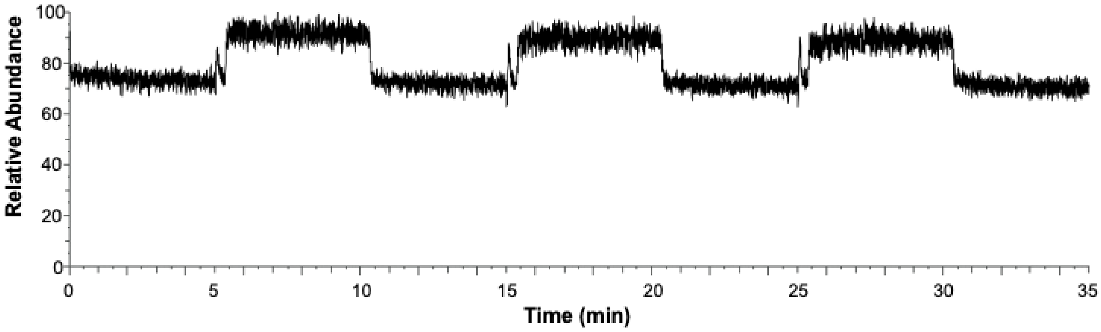

Figure S2. The TIC was obtained from the analysis of stearic acid methyl ester at 50 µM using Dual Inlet ESI(-)-Orbitrap MS. The analysis involved alternating 5-minute infusions between reference and sample, yielding a total runtime of 35 minutes.

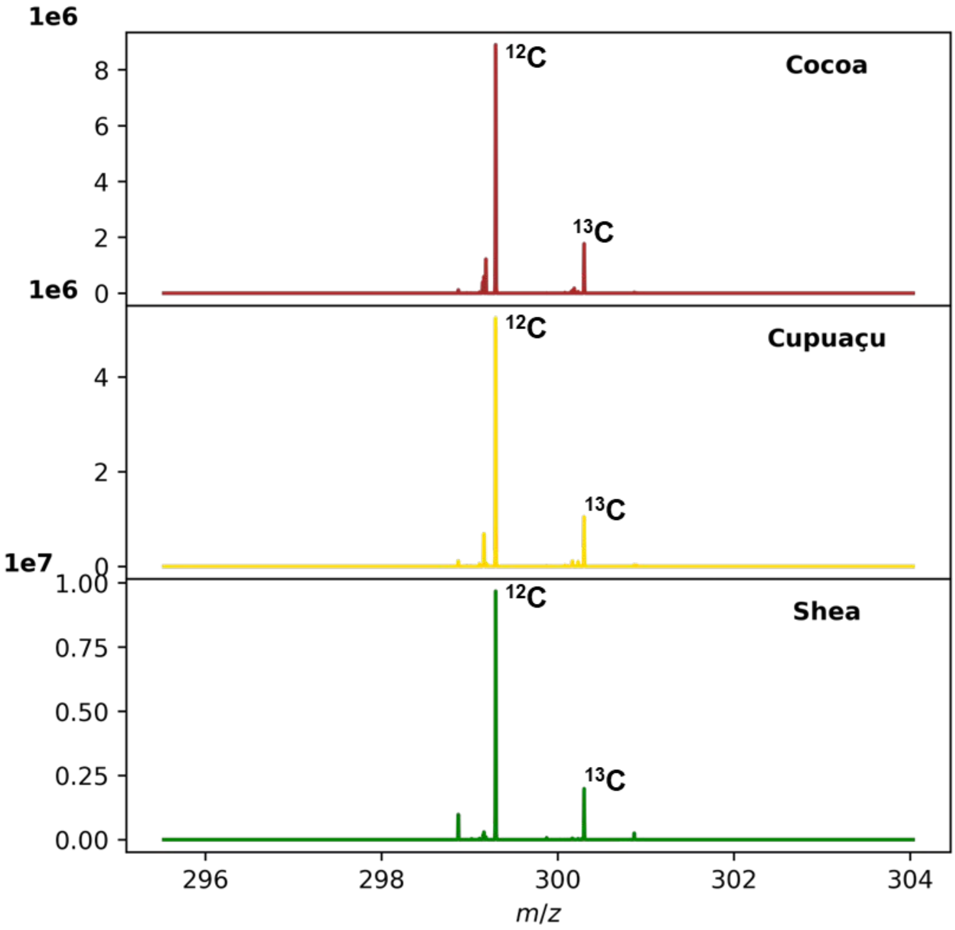

Figure S3. Representative average full scan mass spectra from the analysis blocks of cocoa, cupuaçu, and shea butter samples that showed the highest levels of apparent contamination within the analytical mass range.

Table S1. GC-MS analysis of Cocoa, Shea, and Cupuaçu butter after FAMEs derivatizations.

Compound	Cocoa (%)	Shea (%)	Cupuaçu (%)
Lauric acid methyl ester	-	3.14	-
Palmitic acid methyl ester	26.85	29.43	6.59
Linoleic acid methyl ester	2.65	6.22	3.60
Oleic acid methyl ester	34.11	22.58	47.49
Stearic acid methyl ester	36.40	36.56	42.31
Unkown	-	2.06	-

Data Processing Details

As described in the main text, IsotoPy is an in-house software developed by our research group that performs parameter extraction using the Thermo Fisher Scientific RawFileReader library and calculates ion counts using the same equation as the IsoX software. This section provides additional details on the subsequent steps of data processing.

After extracting relevant scan parameters and computing ion counts, IsotoPy generates a '.isopy' file, which is structurally similar to the '.isox' files produced by Thermo Fisher's IsoX software. Consequently, users can initiate processing using any of the following file types: '.RAW', '.ISOPY', or '.ISOX'. If the user opts to begin with a .RAW file, it is necessary to provide a '.tsv' file that specifies the m/z of the isotopologues of interest, their respective charge states, and the mass tolerance window for ion identification in each scan. Depending on the analysis methodology, input formatting differs:

- For Dual Inlet experiments, the user provides a single file and must specify the number of blocks and the duration of each block.
- For Flow Injection (direct HPLC injection without a chromatographic column), the user must provide one file per block, corresponding to the individual sample or standard injections.

A recurring feature in high-resolution isotopic analyses is the occurrence of zero scans, in which one of the isotopologues is not detected. IsotoPy offers two strategies for handling these scans: (1) remove the scan entirely, or (2) assign a zero-intensity value to the missing isotopologue, as proposed by Csernica et al. (2023).[1] In this study, zero scans were excluded from further processing.

Prior to full processing, the data is typically trimmed to remove portions of the spectrum affected by injection artifacts or signal instability. In Dual Inlet mode, the first minute of each block—known as the switch time—is removed to eliminate artifacts from valve switching. In Flow Injection mode, both the first minute (dead volume) and the final minutes (post-elution baseline) are excluded, as they correspond to low or unstable signal regions. In this study, for analyses conducted using the Dual Inlet methodology, the first minute of each block was removed. For analyses performed using the Flow Injection methodology, each injection block was conducted over a 15-minute period, and the TIC was trimmed between minutes 1 and 8.

Within the retained spectral range, IsotoPy performs outlier scan removal. Multiple filtering strategies are available in the software; for this study, we used the Median Absolute Deviation (MAD) Score method, applying a 2-MAD threshold with a sliding window.

After preprocessing, the isotopic ratio is calculated on a scan-by-scan basis as the ratio between ion counts of the relevant isotopologues. An average isotopic ratio is then computed for each block. The isotopic ratio of each sample block is compared to the mean ratio of the neighboring standard blocks, and $\delta^{13}C$ values are calculated accordingly.

To ensure robustness, IsotoPy implements several diagnostic procedures throughout the processing pipeline. These include checks for peak resolution consistency, normality tests, acquisition error assessment, and reproducibility metrics.

Accompanying this Supporting Information are PDF reports for each replicate analysis of cocoa, shea, and cupuaçu butters, as well as for each standard compound analyzed. These reports detail all processing steps described here. Within these reports, the following sections are provided:

1. Pre Processing:

This section summarizes the preprocessing steps applied to each acquisition block. It includes the trimming of non-informative time intervals and the removal of outlier scans using the MAD Score method. It presents the effective time range used for each block, the number of valid scans retained, a representative plot showing removed outliers, and the processed TIC profile, with associated minimum, maximum, and average intensity values, along with error estimates.

2. Block Parameters:

This section presents the main parameters calculated for each individual analysis block. Key metrics include the isotopic ratio, number of scans, effective ion count, standard deviation (STD), standard error of the mean (SEM), and relative standard error (RSE).

Additionally, it reports the acquisition error ($\sigma_{AE,}$ defined as the RSE in ‰) and shot-noise estimates. The AE/SN (acquisition error to shot-noise) ratio remained close to 1 across most blocks, well below the critical threshold of 2. Values above 2 are typically associated with poor quality data, as previously reported by Csernica et al. (2023).[1]

These parameters are shown in tabular form and are complemented by graphical outputs, including: the cumulative and average isotopic ratio over time for each block; a histogram of isotopic ratios; and a comparison plot showing the evolution of acquisition error versus shot-noise throughout each analysis block.

3. Delta Informations:

This section presents the $\delta^{13}C$ values calculated for each sample block, as well as the corrected values based on the reference standard used in each analysis. The standard error of the mean (SEM) is calculated from the variability of the isotope ratios within each sample block and is reported as σ_{SEM}, reflecting intra-block precision. The variability among the $\delta^{13}C$ values obtained from replicated analyses is expressed as σ_{ER} (experimental reproducibility), providing an estimate of overall measurement reproducibility.

Additionally, the final average $\delta^{13}C$ value is also reported, representing the global delta associated with the analyzed sample. This value is corrected to the VPDB scale and reflects the result of isotopic analysis.

References

(1) Csernica, T.; Bhattacharjee, S.; Eiler, J. Accuracy and Precision of ESI-Orbitrap-IRMS Observations of Hours to Tens of Hours via Reservoir Injection. *Int J Mass Spectrom* **2023**, *490*. https://doi.org/10.1016/j.ijms.2023.117084.
