# Supplementary material for: Advancing Stable Isotope Analysis with Orbitrap-MS for Fatty Acid Methyl Esters and Complex Lipid Matrices
Source: J Am Soc Mass Spectrom. 2025 Jun 17;36(7):1527–35. doi: 10.1021/jasms.5c00092 (PMC12339014; doi:10.1021/jasms.5c00092)
Supplement: Supplementary file 2 [file js5c00092_si_002.zip › reports by IsotoPy Software/butters/Cocoa_rep3.pdf]

Cocoa butter (replicate 3)
Isotope Analysis report from IsotoPy

Flow Injection

1. Pre Processing

1.1. Block Time and Scan Information

Information about sample and standard block times and scans:

Block	Injected	Initial Time	End Time	Number of scans
1	standard	1	8	1269
2	sample	16	23	1305
3	standard	31	38	1325
4	sample	46	53	1291
5	standard	61	68	1280
6	sample	76	83	1298
7	standard	91	98	1289

1.2. Outlier Removal

A total of 1982 scans were considered outliers and removed using the MAD method

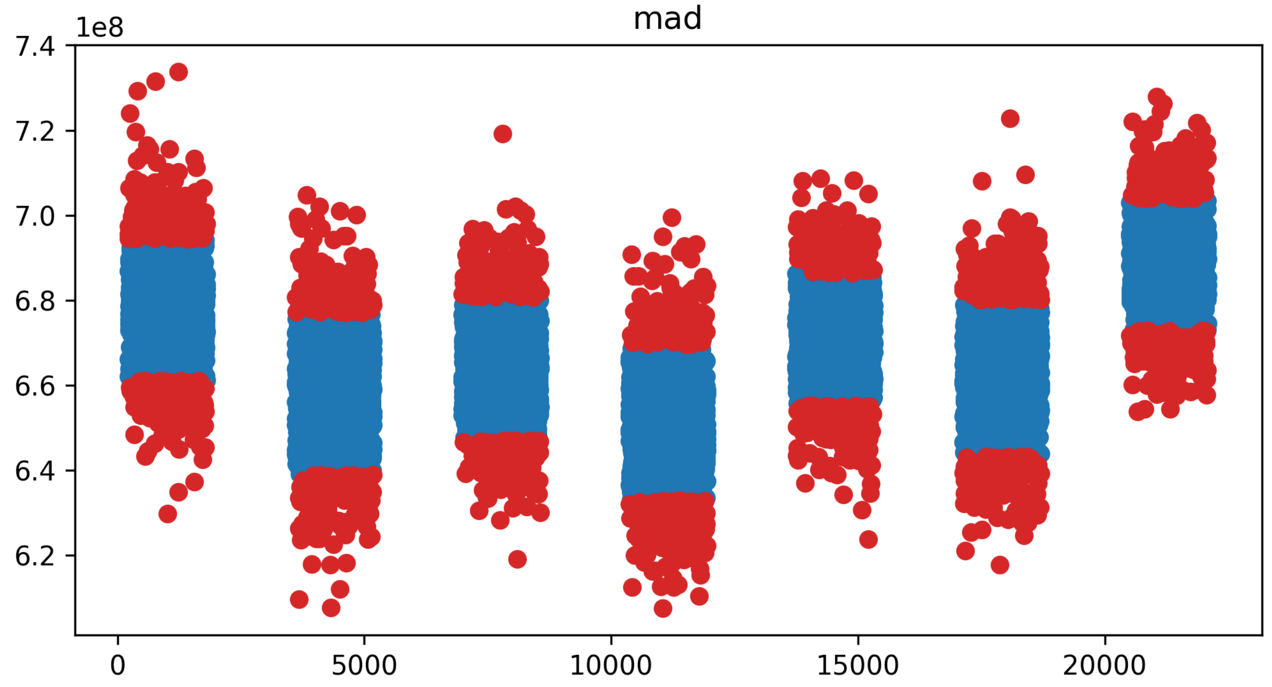

1.3. Total Ion Current (TIC)

TIC of all blocks

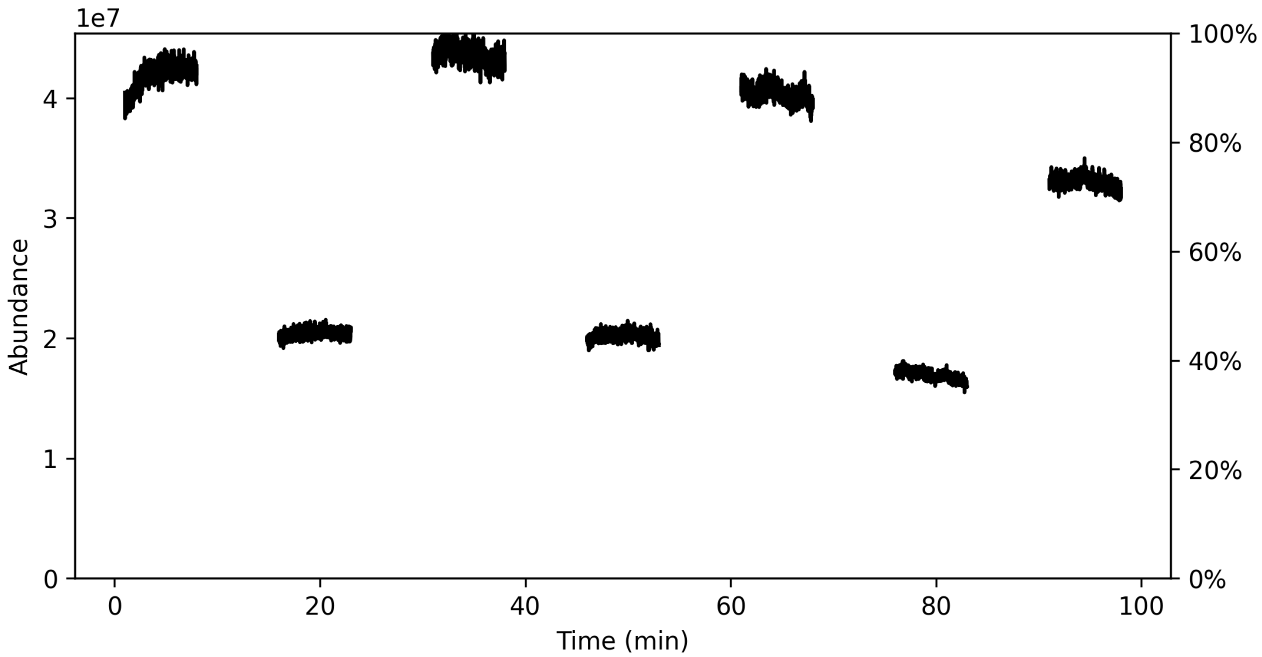

Block	TIC min	TIC max	TIC mean	RSD (%)
1	3.83e+07	4.41e+07	4.19e+07	2.49
2	1.92e+07	2.15e+07	2.04e+07	1.69
3	4.13e+07	4.54e+07	4.36e+07	1.61
4	1.90e+07	2.14e+07	2.01e+07	1.73
5	3.81e+07	4.24e+07	4.04e+07	1.60
6	1.55e+07	1.81e+07	1.69e+07	2.25
7	3.15e+07	3.50e+07	3.30e+07	1.51

2. Block Parameters

The Isotopic Ratio of the blocks were calculated by 'Mean'

2.1. 13C/M0

Block	Number of scans	Effective number of ions	Isotopic Ratio	STD	SEM	RSE
1	1269	1.73e+07	0.196880	0.001667	0.000047	0.000238
2	1305	1.71e+07	0.196210	0.001718	0.000048	0.000242
3	1325	1.86e+07	0.197097	0.001648	0.000045	0.000230
4	1291	1.74e+07	0.196515	0.001766	0.000049	0.000250
5	1280	1.84e+07	0.196962	0.001690	0.000047	0.000240
6	1298	1.76e+07	0.196816	0.001720	0.000048	0.000242
7	1289	1.87e+07	0.196917	0.001711	0.000048	0.000242

Errors and Test Paramters

Block	Acquisition Error (permil)	Shot-Noise (permil)	AE/SN ratio	Shapiro Wilk (p_value)	D'Agostino (p_value)
1	0.238	0.240	0.990	0.628	0.333
2	0.242	0.242	1.003	0.235	0.433
3	0.230	0.232	0.991	0.467	0.464
4	0.250	0.240	1.042	0.397	0.276
5	0.240	0.233	1.027	0.459	0.400
6	0.242	0.239	1.016	0.517	0.484
7	0.242	0.231	1.046	0.191	0.351

Isotopic Ratio and Errors of the Blocks

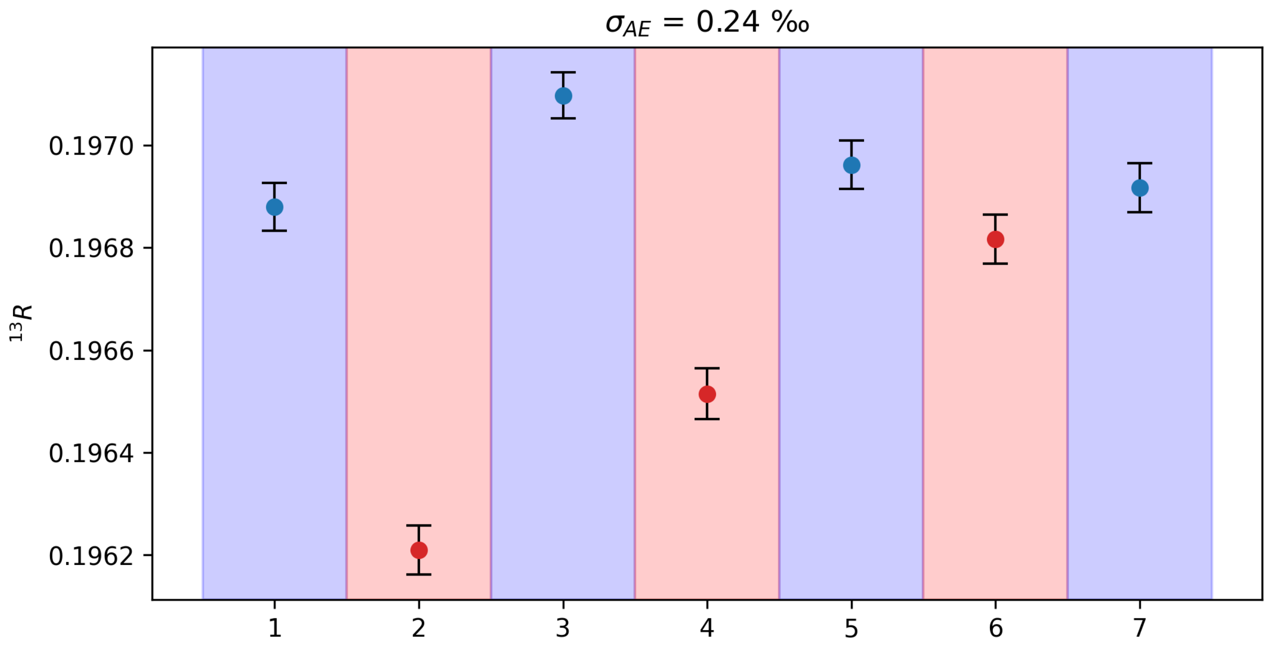

Cumulative Isotopic Ratio

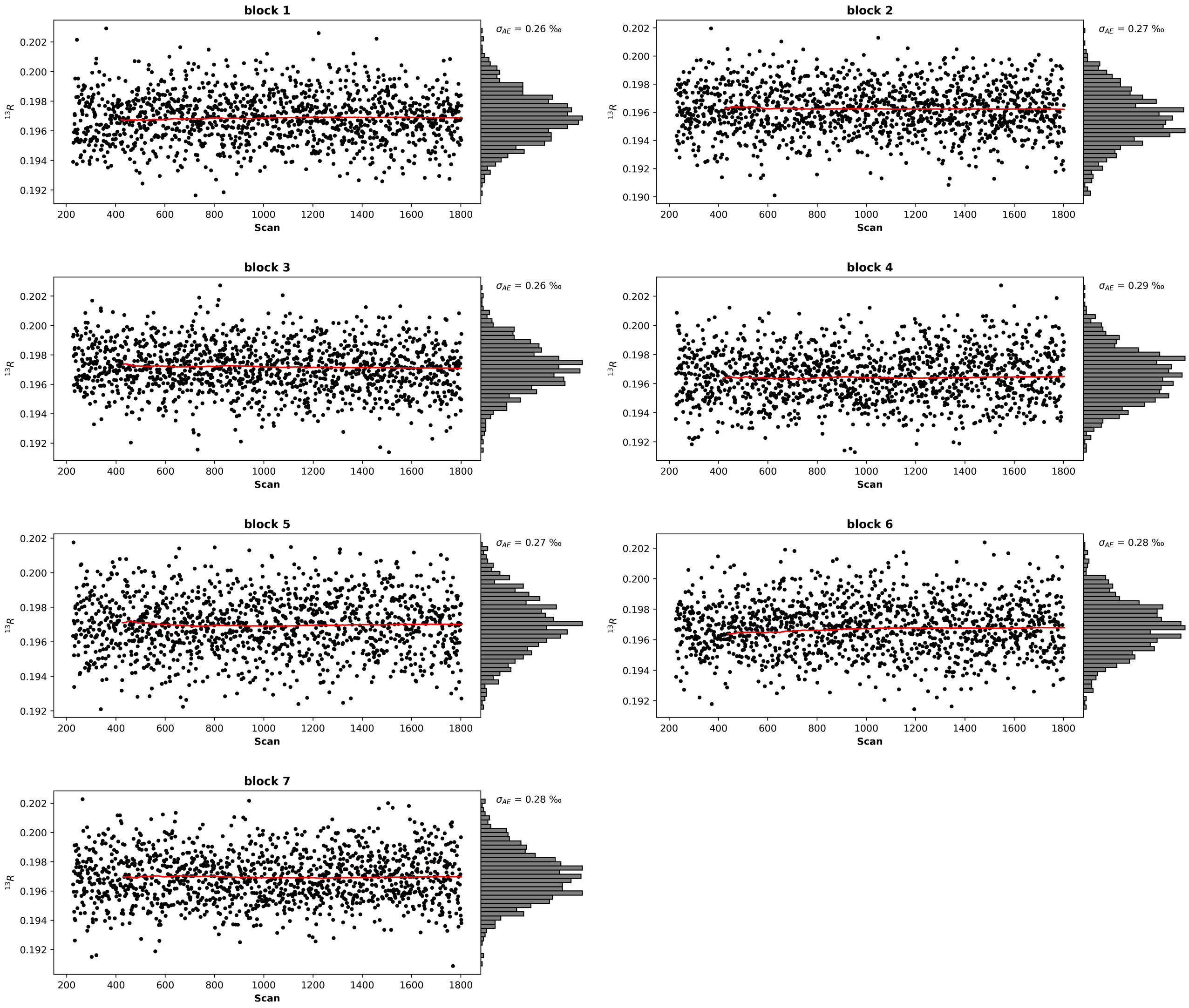

Acquisition Error and Shot-Noise

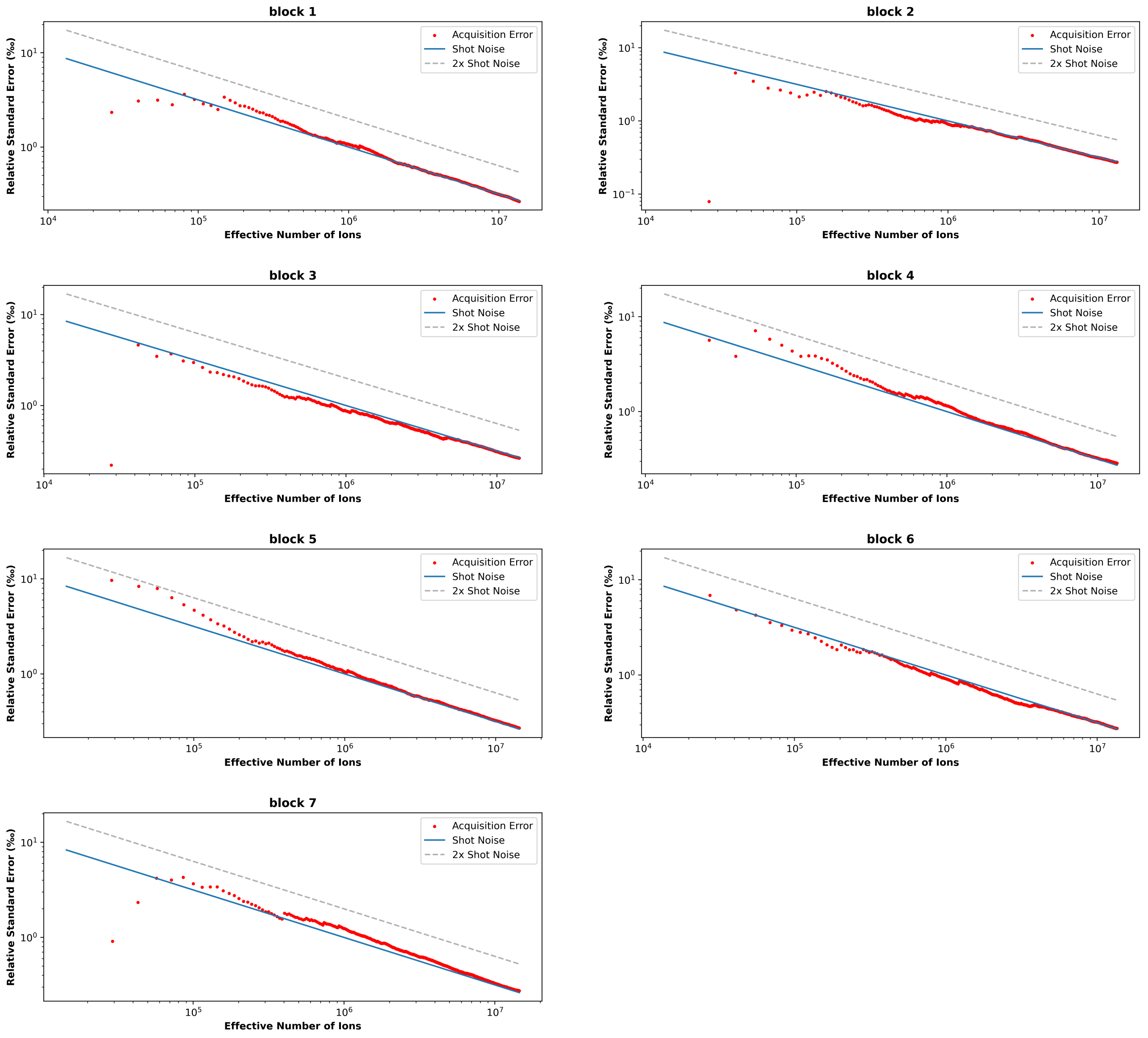

3. Delta Informations

Deltas were calculated by 'Average Of Neighboring Block Ratios'

3.1. 13C

Delta 13C was corrected by -27.80

Block	SEM	Delta corrected	Delta
2	0.24	-31.64	-3.95
4	0.25	-30.34	-2.61
6	0.24	-28.41	-0.62

Delta (corrected) of the Sample Blocks

σ_{sem} = 0.24 ‰

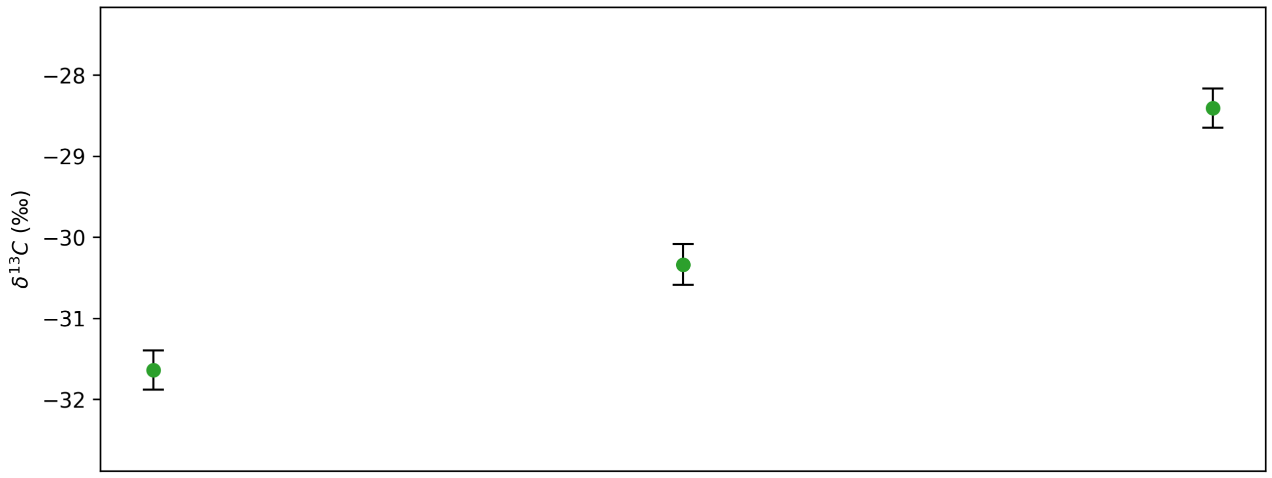

Average Delta (corrected)

σ_{ER} = 1.33 ‰

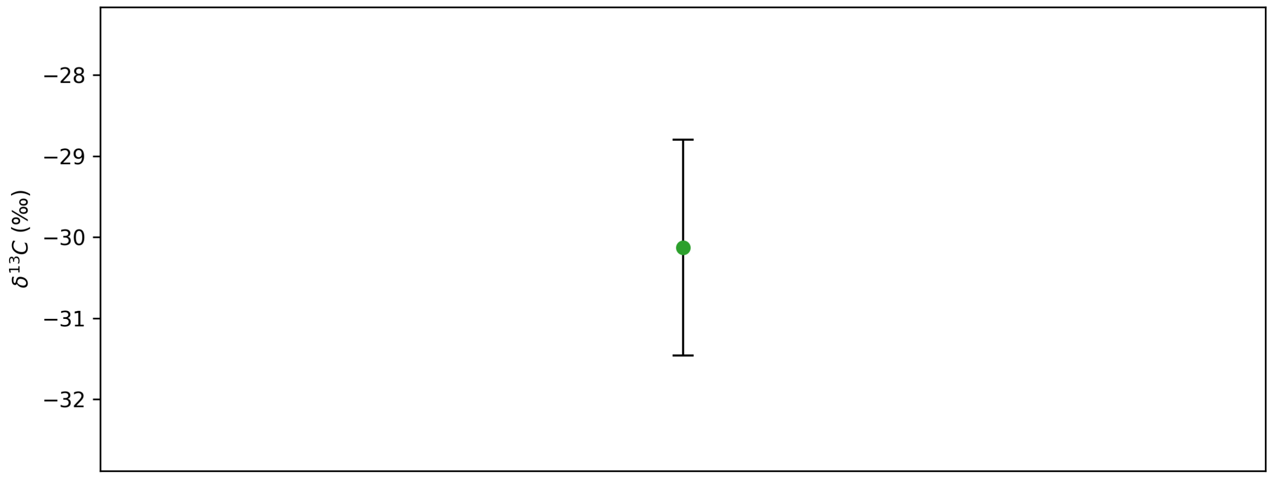

The final corrected average delta was -30.13 with a standard deviation of 1.33. Here the standard deviation is called reproducibility error.
