# Supplementary material for: Advancing Stable Isotope Analysis with Orbitrap-MS for Fatty Acid Methyl Esters and Complex Lipid Matrices
Source: J Am Soc Mass Spectrom. 2025 Jun 17;36(7):1527–35. doi: 10.1021/jasms.5c00092 (PMC12339014; doi:10.1021/jasms.5c00092)
Supplement: Supplementary file 2 [file js5c00092_si_002.zip › reports by IsotoPy Software/butters/Cupuac╠ou_rep3.pdf]

Cupuaçu butter (replicate 3)
Isotope Analysis report from IsotoPy

Flow Injection

1. Pre Processing

1.1. Block Time and Scan Information

Information about sample and standard block times and scans:

Block	Injected	Initial Time	End Time	Number of scans
1	standard	1	8	1303
2	sample	16	23	1281
3	standard	31	38	1255
4	sample	46	53	1326
5	standard	61	68	1280
6	sample	76	83	1294
7	standard	91	98	1283

1.2. Outlier Removal

A total of 2017 scans were considered outliers and removed using the MAD method

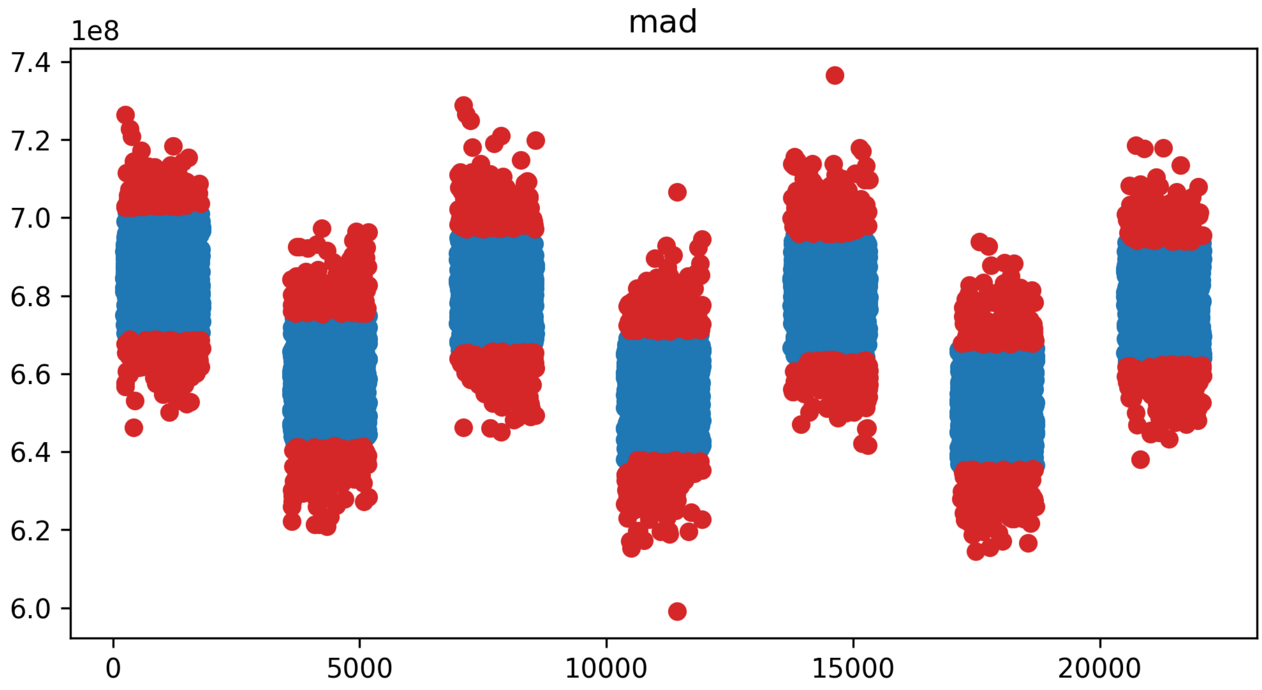

1.3. Total Ion Current (TIC)

TIC of all blocks

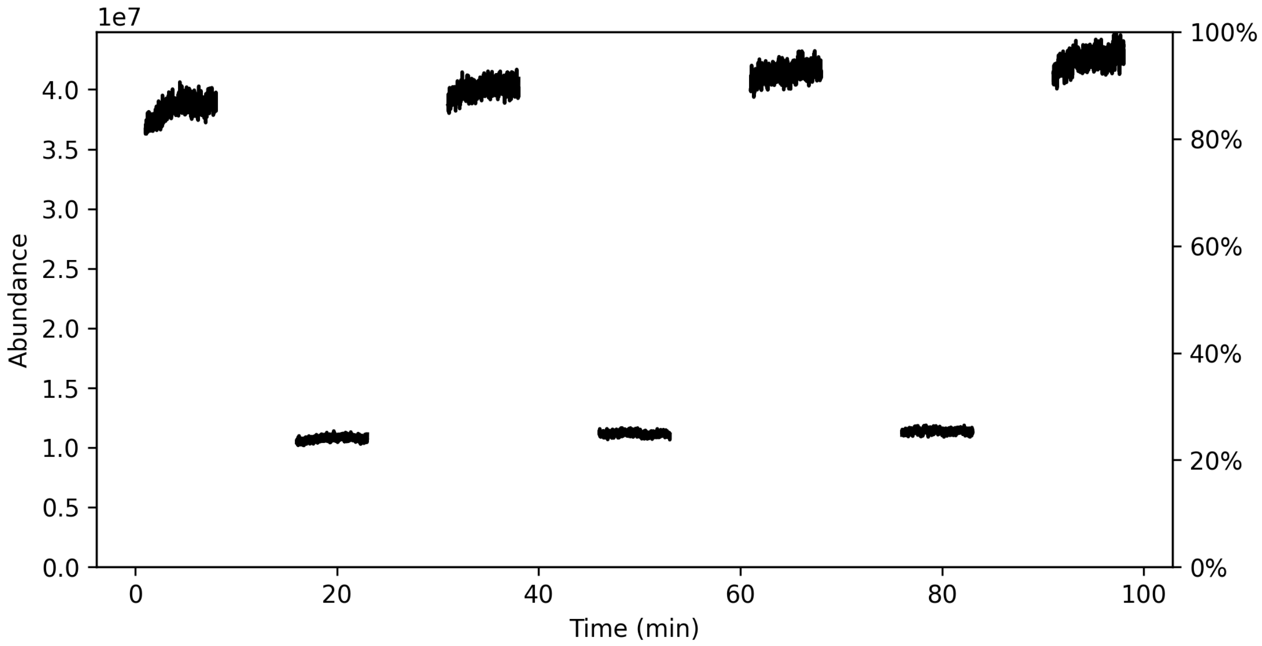

Block	TIC min	TIC max	TIC mean	RSD (%)
1	3.63e+07	4.06e+07	3.84e+07	2.04
2	1.02e+07	1.14e+07	1.07e+07	1.79
3	3.80e+07	4.17e+07	4.00e+07	1.47
4	1.07e+07	1.16e+07	1.12e+07	1.49
5	3.94e+07	4.32e+07	4.14e+07	1.46
6	1.09e+07	1.19e+07	1.14e+07	1.51
7	4.01e+07	4.48e+07	4.25e+07	1.70

2. Block Parameters

The Isotopic Ratio of the blocks were calculated by 'Mean'

2.1. 13C/M0

Block	Number of scans	Effective number of ions	Isotopic Ratio	STD	SEM	RSE
1	1303	1.73e+07	0.196793	0.001681	0.000047	0.000237
2	1281	1.59e+07	0.195729	0.001640	0.000046	0.000234
3	1255	1.66e+07	0.196941	0.001672	0.000047	0.000240
4	1326	1.66e+07	0.195788	0.001752	0.000048	0.000246
5	1280	1.69e+07	0.197109	0.001744	0.000049	0.000247
6	1294	1.62e+07	0.196008	0.001700	0.000047	0.000241
7	1283	1.69e+07	0.197070	0.001605	0.000045	0.000227

Errors and Test Paramters

Block	Acquisition Error (permil)	Shot-Noise (permil)	AE/SN ratio	Shapiro Wilk (p_value)	D'Agostino (p_value)
1	0.237	0.241	0.983	0.554	0.265
2	0.234	0.251	0.934	0.210	0.775
3	0.240	0.245	0.976	0.387	0.654
4	0.246	0.246	0.999	0.130	0.117
5	0.247	0.243	1.016	0.008	0.108
6	0.241	0.248	0.970	0.075	0.054
7	0.227	0.243	0.936	0.700	0.740

Isotopic Ratio and Errors of the Blocks

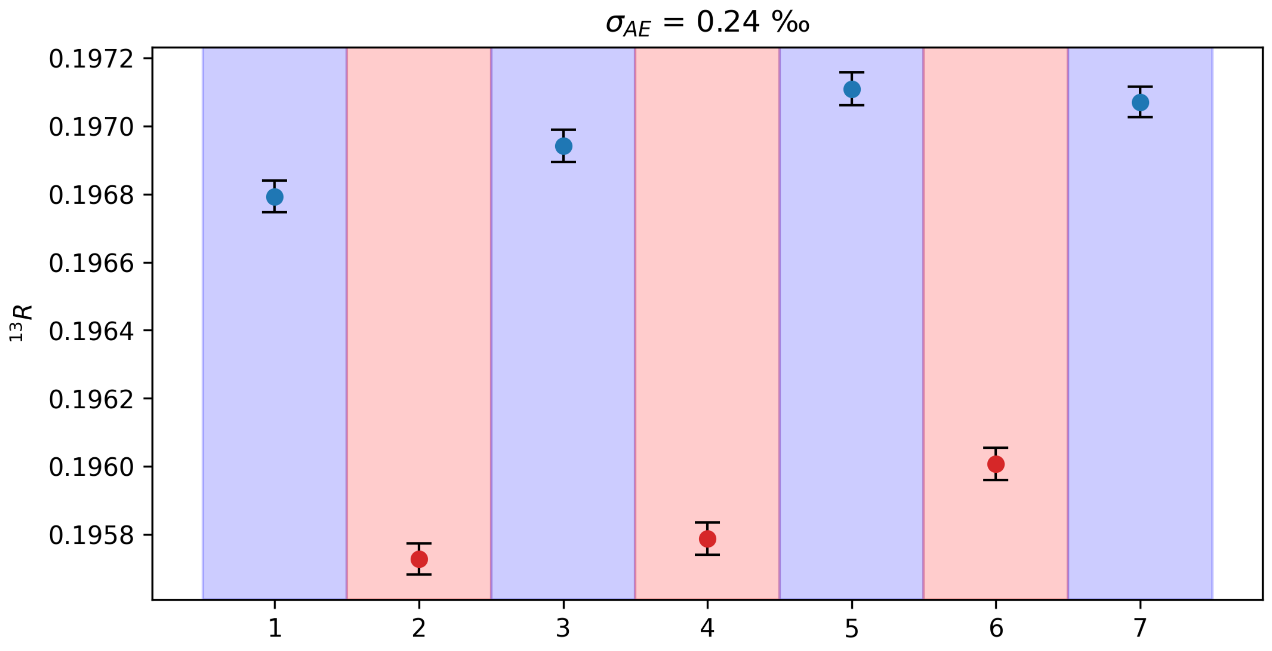

Cumulative Isotopic Ratio

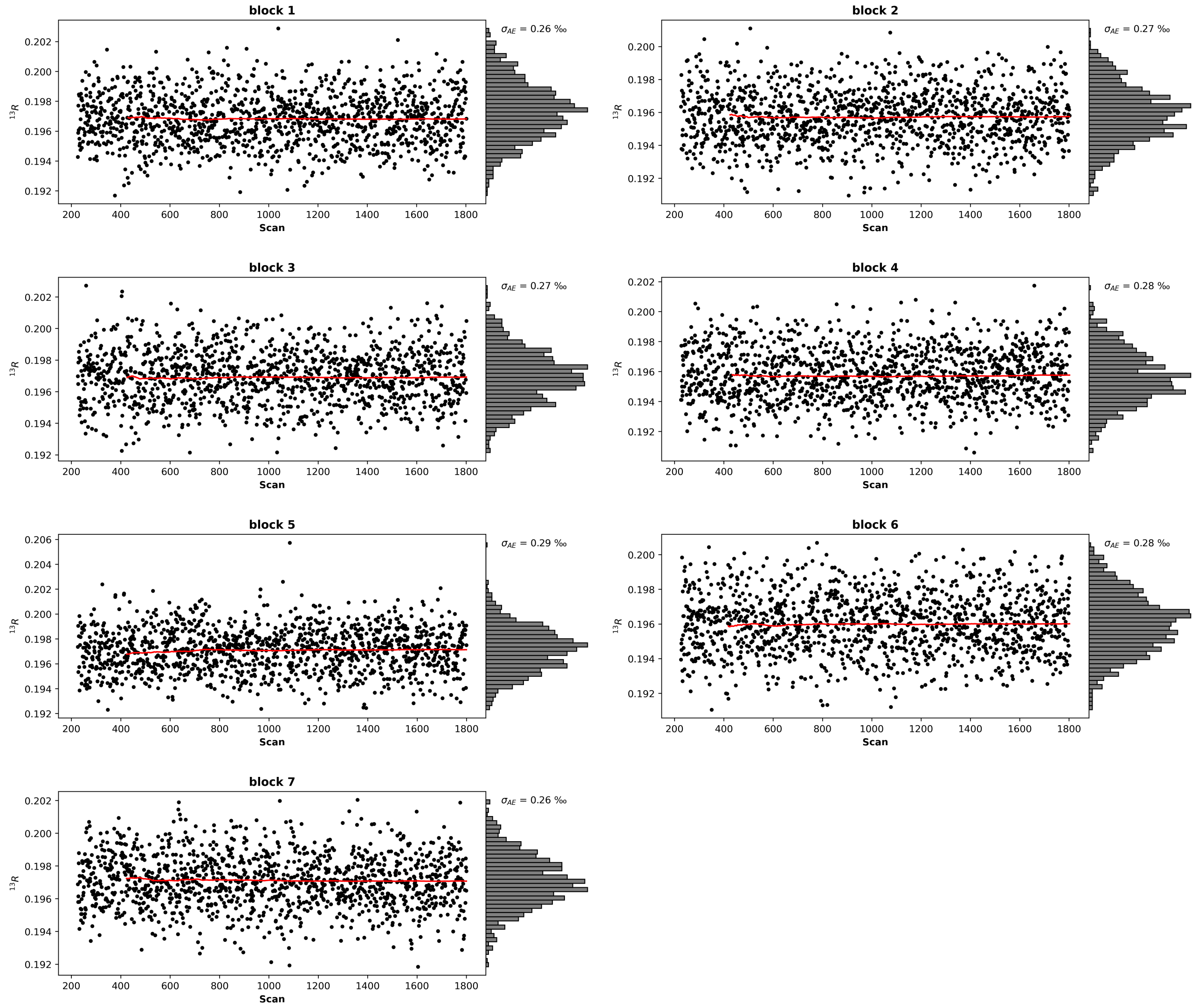

Acquisition Error and Shot-Noise

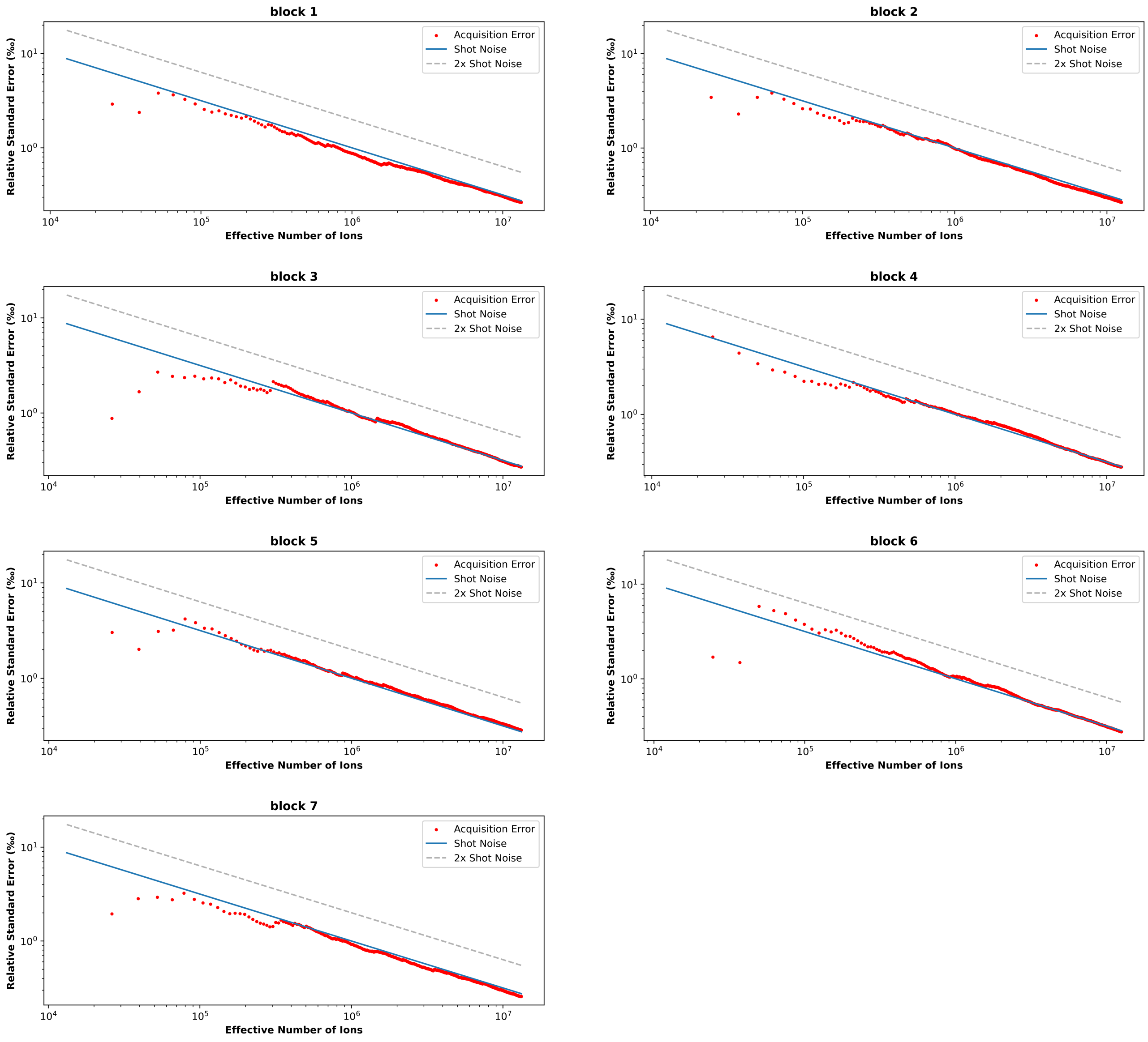

3. Delta Informations

Deltas were calculated by 'Average Of Neighboring Block Ratios'

3.1. 13C

Delta 13C was corrected by -27.80

Block	SEM	Delta corrected	Delta
2	0.23	-33.42	-5.78
4	0.24	-33.91	-6.28
6	0.24	-33.14	-5.49

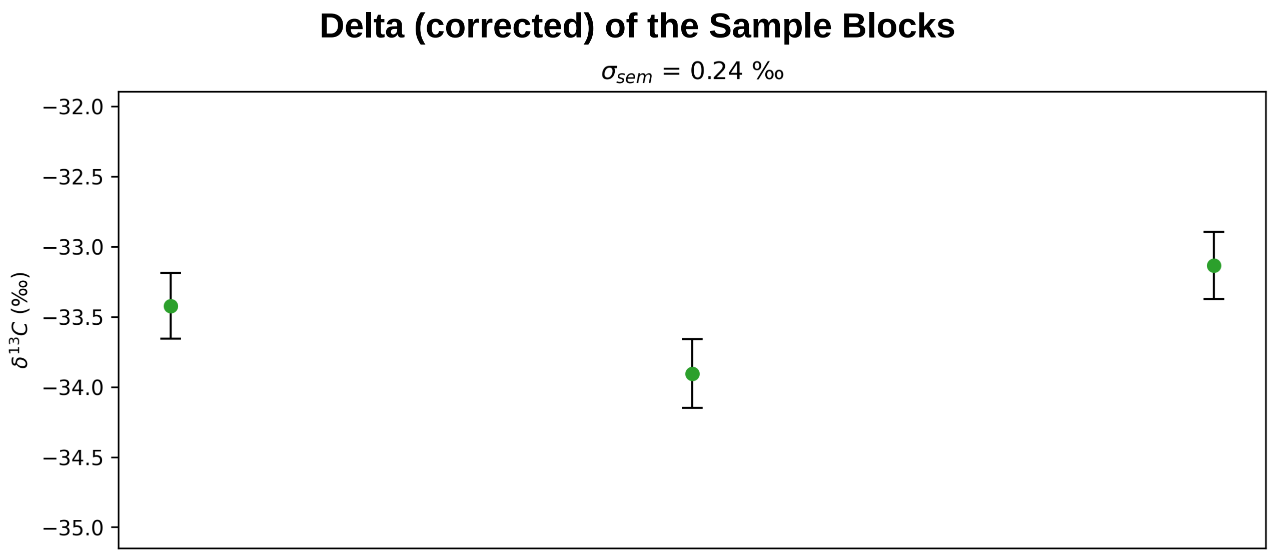

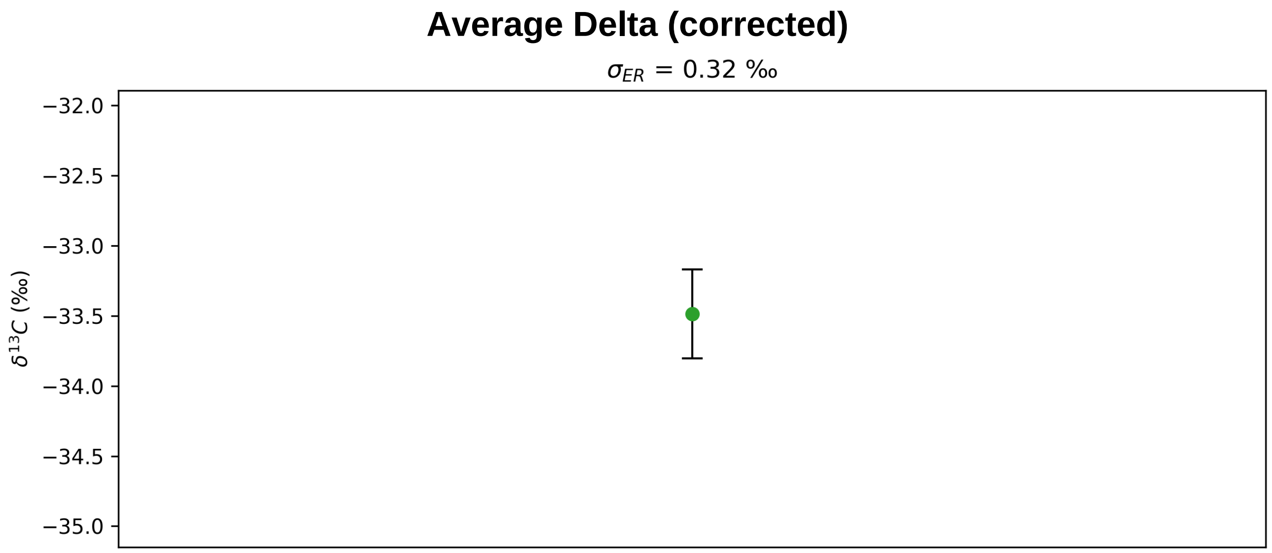

The final corrected average delta was -33.49 with a standard deviation of 0.32. Here the standard deviation is called reproducibility error.
