# Supplementary material for: Advancing Stable Isotope Analysis with Orbitrap-MS for Fatty Acid Methyl Esters and Complex Lipid Matrices
Source: J Am Soc Mass Spectrom. 2025 Jun 17;36(7):1527–35. doi: 10.1021/jasms.5c00092 (PMC12339014; doi:10.1021/jasms.5c00092)
Supplement: Supplementary file 2 [file js5c00092_si_002.zip › reports by IsotoPy Software/butters/Cupuac╠ou_rep2.pdf]

Cupuaçu butter (replicate 2)
Isotope Analysis report from IsotoPy

Flow Injection

1. Pre Processing

1.1. Block Time and Scan Information

Information about sample and standard block times and scans:

Block	Injected	Initial Time	End Time	Number of scans
1	standard	1	7	1183
2	sample	16	23	1269
3	standard	31	38	1288
4	sample	46	53	1309
5	standard	61	68	1287
6	sample	76	83	1313
7	standard	91	98	1284

1.2. Outlier Removal

A total of 2113 scans were considered outliers and removed using the MAD method

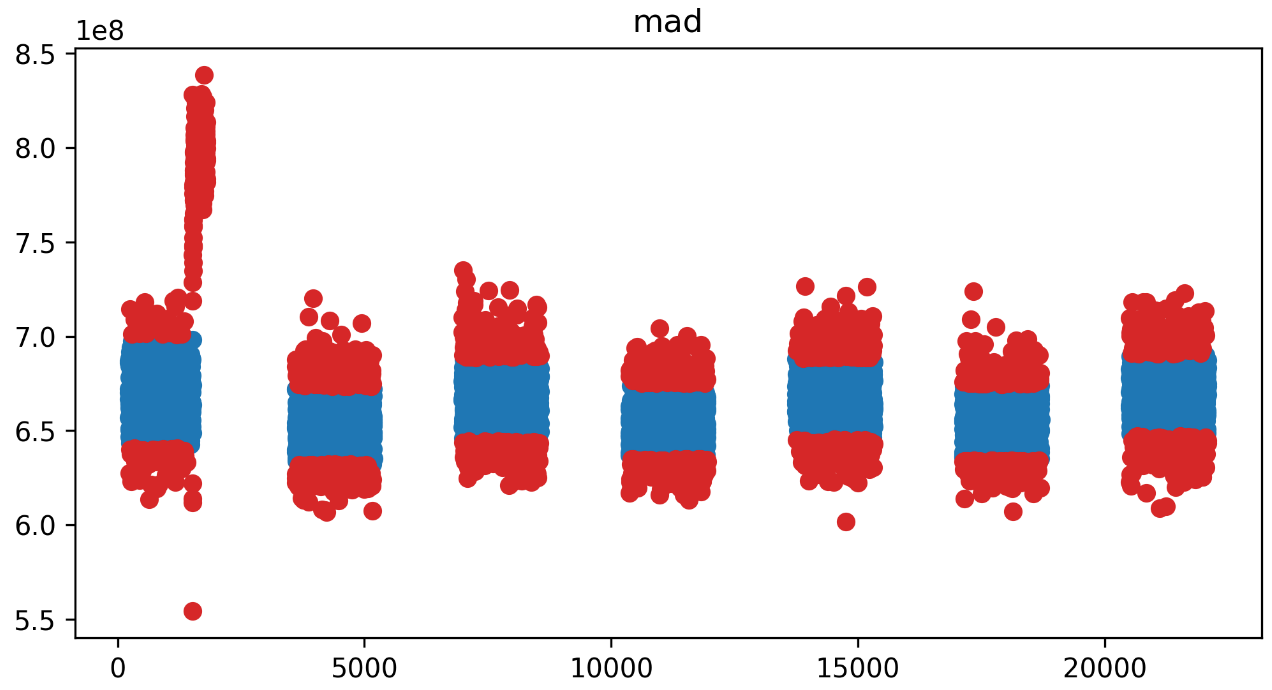

1.3. Total Ion Current (TIC)

TIC of all blocks

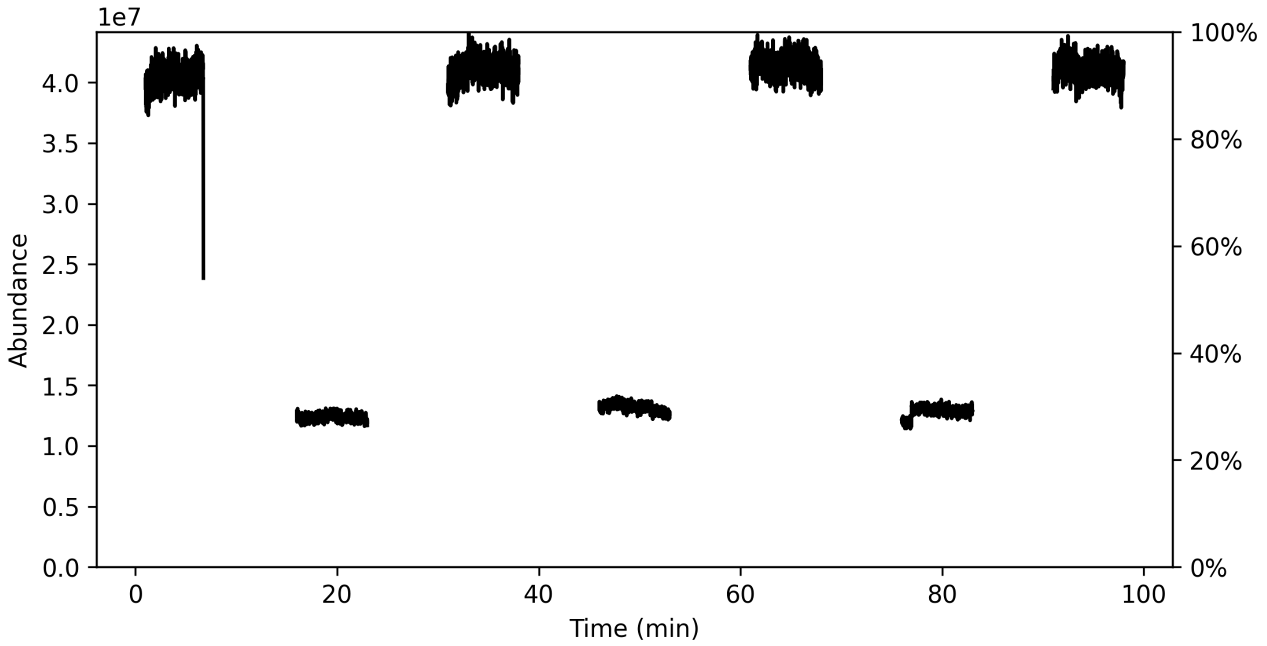

Block	TIC min	TIC max	TIC mean	RSD (%)
1	2.39e+07	4.31e+07	4.05e+07	2.51
2	1.16e+07	1.31e+07	1.24e+07	2.29
3	3.81e+07	4.42e+07	4.11e+07	2.16
4	1.22e+07	1.41e+07	1.32e+07	2.67
5	3.89e+07	4.39e+07	4.14e+07	2.01
6	1.14e+07	1.38e+07	1.28e+07	3.40
7	3.79e+07	4.39e+07	4.10e+07	1.95

2. Block Parameters

The Isotopic Ratio of the blocks were calculated by 'Mean'

2.1. 13C/M0

Block	Number of scans	Effective number of ions	Isotopic Ratio	STD	SEM	RSE
1	1183	1.56e+07	0.196463	0.001689	0.000049	0.000250
2	1269	1.59e+07	0.195955	0.001758	0.000049	0.000252
3	1288	1.69e+07	0.196569	0.001693	0.000047	0.000240
4	1309	1.63e+07	0.195780	0.001667	0.000046	0.000235
5	1287	1.69e+07	0.196497	0.001666	0.000046	0.000236
6	1313	1.64e+07	0.195749	0.001729	0.000048	0.000244
7	1284	1.69e+07	0.196501	0.001674	0.000047	0.000238

Errors and Test Paramters

Block	Acquisition Error (permil)	Shot-Noise (permil)	AE/SN ratio	Shapiro Wilk (p_value)	D'Agostino (p_value)
1	0.250	0.253	0.987	0.485	0.546
2	0.252	0.251	1.003	0.085	0.125
3	0.240	0.243	0.986	0.506	0.745
4	0.235	0.247	0.951	0.529	0.851
5	0.236	0.243	0.971	0.683	0.281
6	0.244	0.247	0.986	0.424	0.644
7	0.238	0.243	0.977	0.894	0.530

Isotopic Ratio and Errors of the Blocks

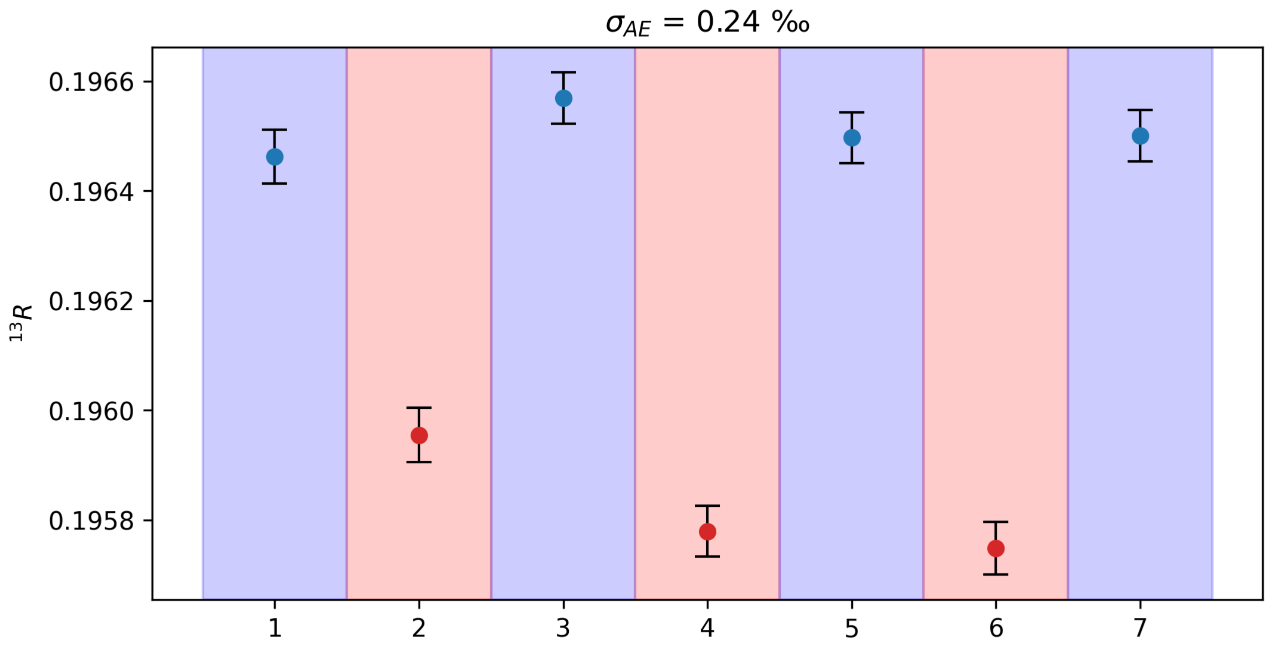

Cumulative Isotopic Ratio

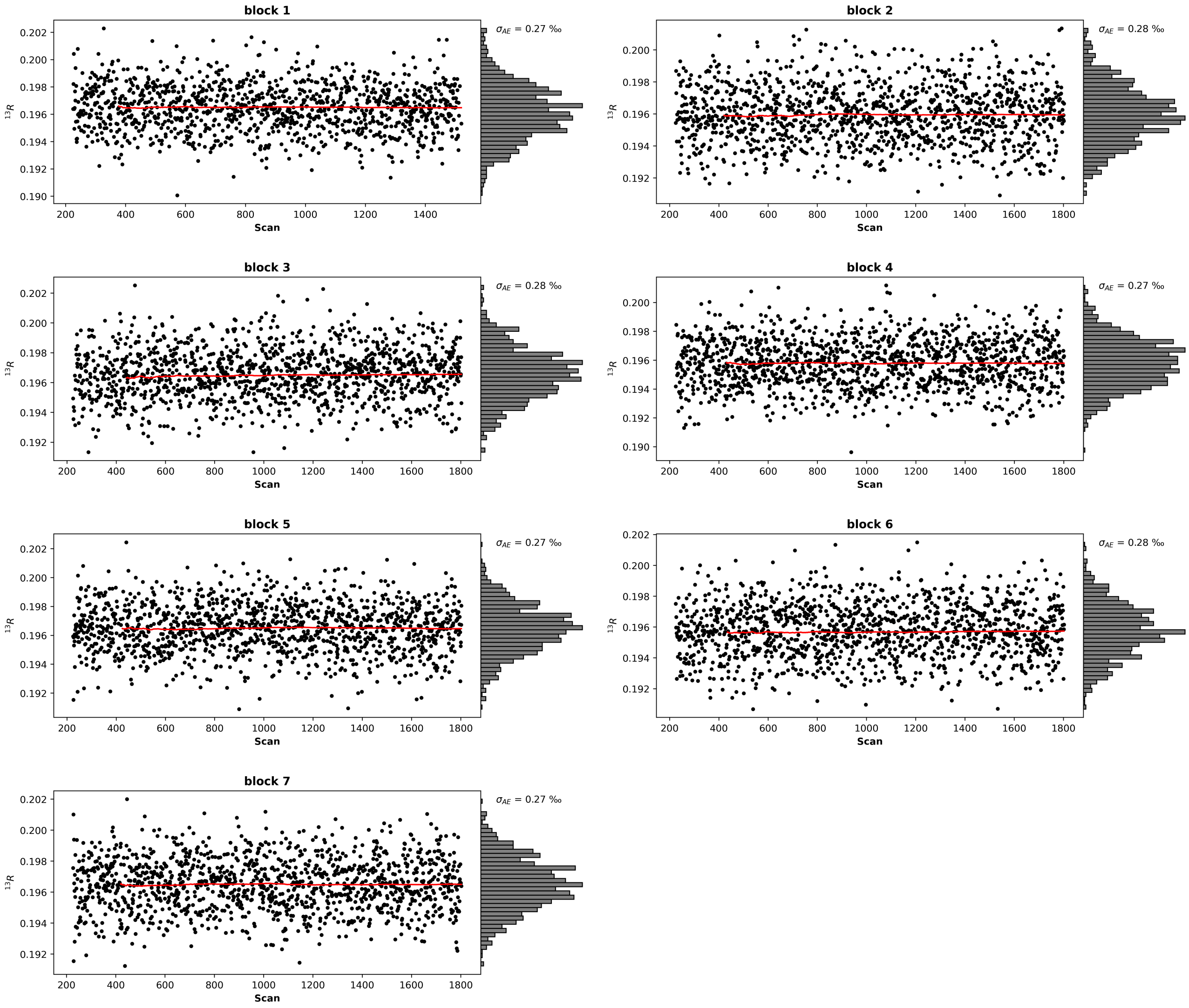

Acquisition Error and Shot-Noise

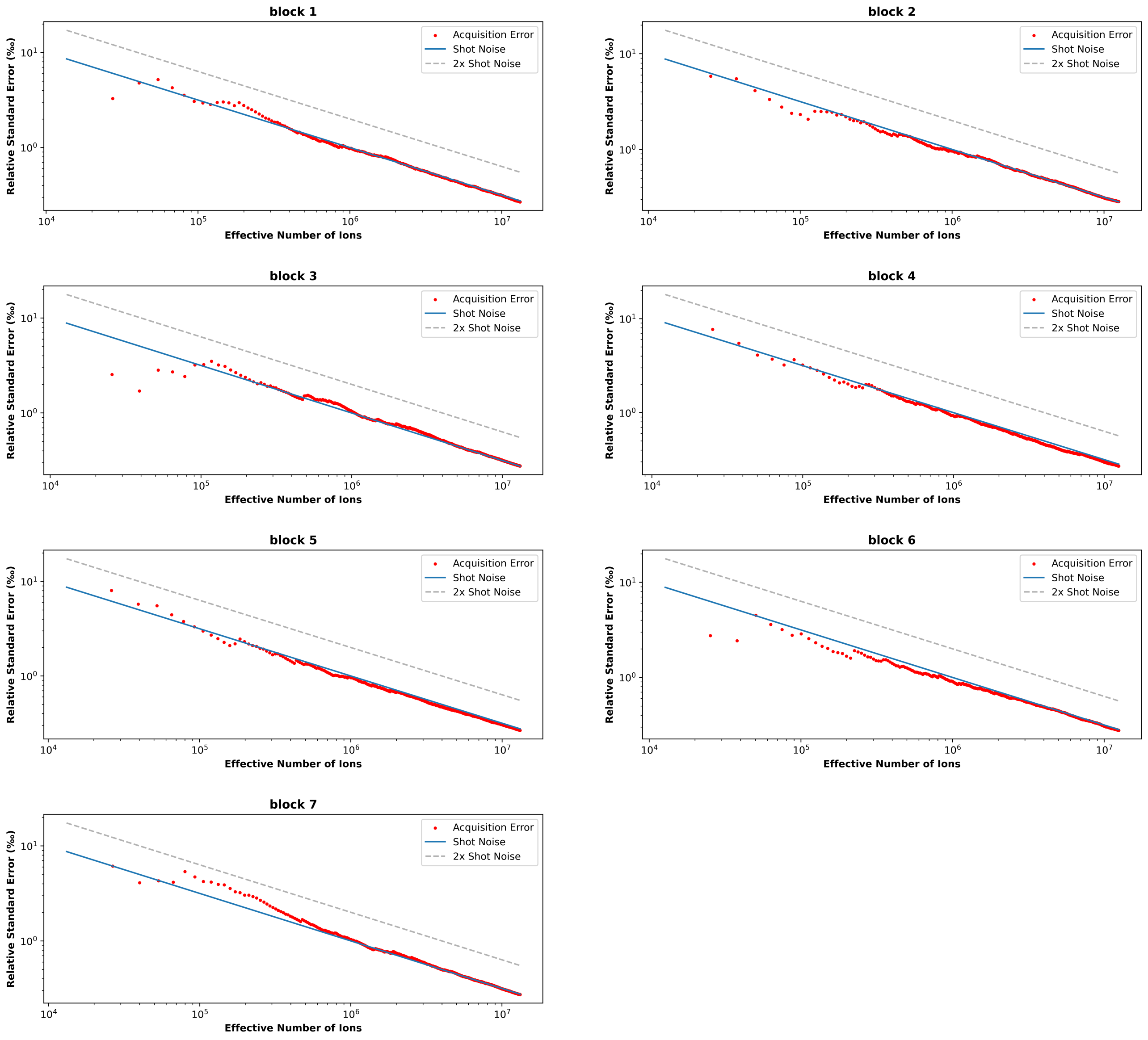

3. Delta Informations

Deltas were calculated by 'Average Of Neighboring Block Ratios'

3.1. 13C

Delta 13C was corrected by -27.80

Block	SEM	Delta corrected	Delta
2	0.25	-30.57	-2.85
4	0.23	-31.53	-3.83
6	0.24	-31.51	-3.82

Delta (corrected) of the Sample Blocks

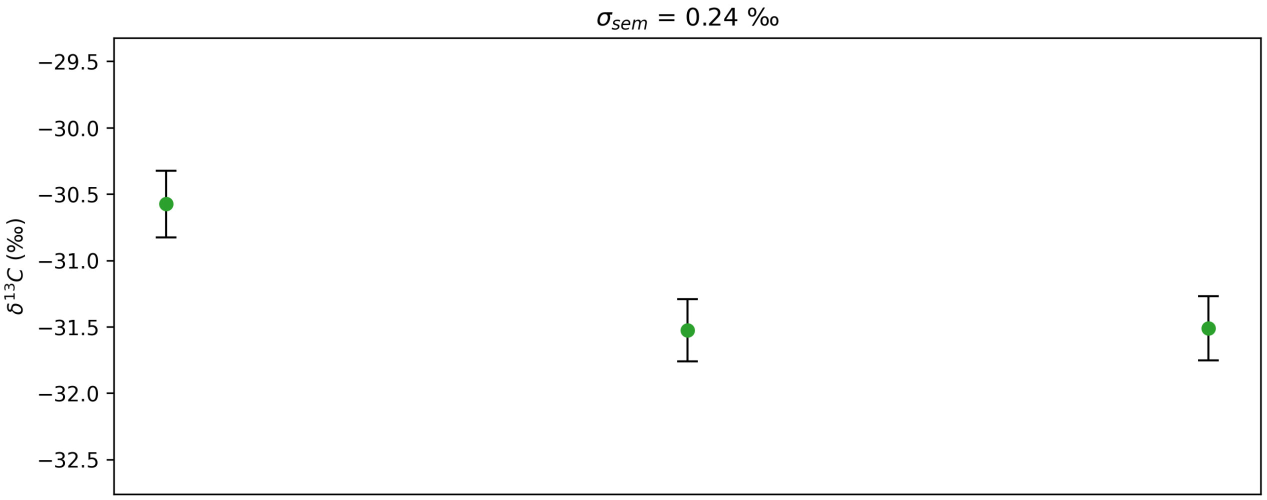

Average Delta (corrected)

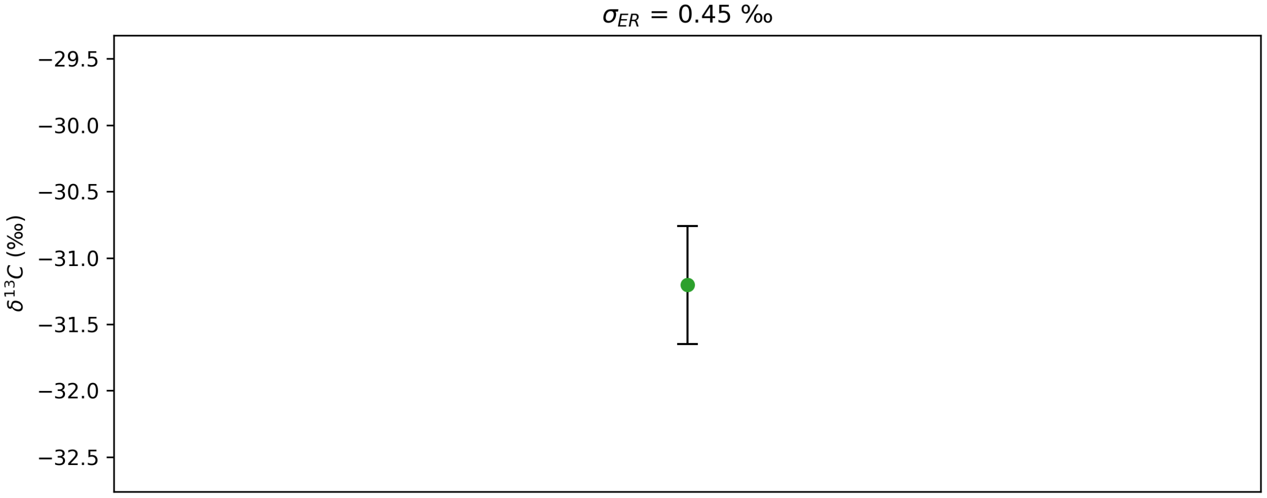

The final corrected average delta was -31.20 with a standard deviation of 0.45. Here the standard deviation is called reproducibility error.
