# Supplementary material for: Advancing Stable Isotope Analysis with Orbitrap-MS for Fatty Acid Methyl Esters and Complex Lipid Matrices
Source: J Am Soc Mass Spectrom. 2025 Jun 17;36(7):1527–35. doi: 10.1021/jasms.5c00092 (PMC12339014; doi:10.1021/jasms.5c00092)
Supplement: Supplementary file 2 [file js5c00092_si_002.zip › reports by IsotoPy Software/butters/Cocoa_rep2.pdf]

Cocoa butter (replicate 2)
Isotope Analysis report from IsotoPy
Flow Injection

1. Pre Processing

1.1. Block Time and Scan Information

Information about sample and standard block times and scans:

Block	Injected	Initial Time	End Time	Number of scans
1	standard	1	8	1318
2	sample	18	23	1083
3	standard	31	38	1266
4	sample	46	53	1313
5	standard	61	68	1266
6	sample	76	83	1280
7	standard	91	97	1153

1.2. Outlier Removal

A total of 2361 scans were considered outliers and removed using the MAD method

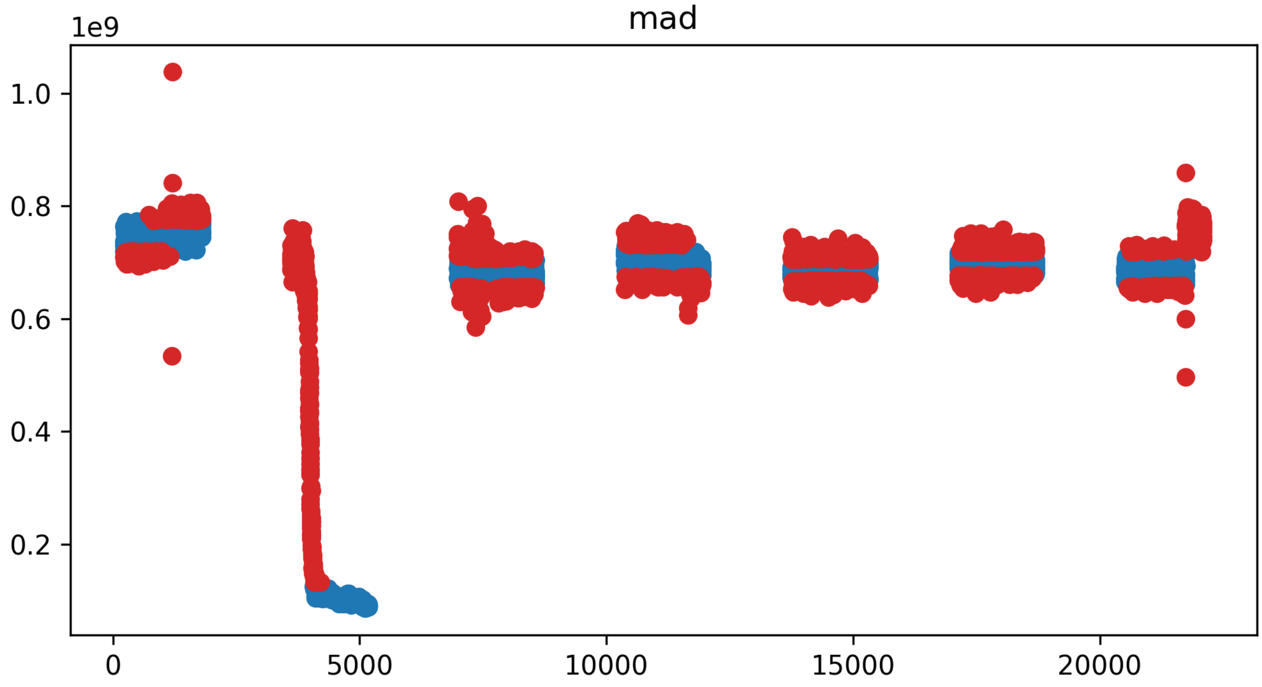

1.3. Total Ion Current (TIC)

TIC of all blocks

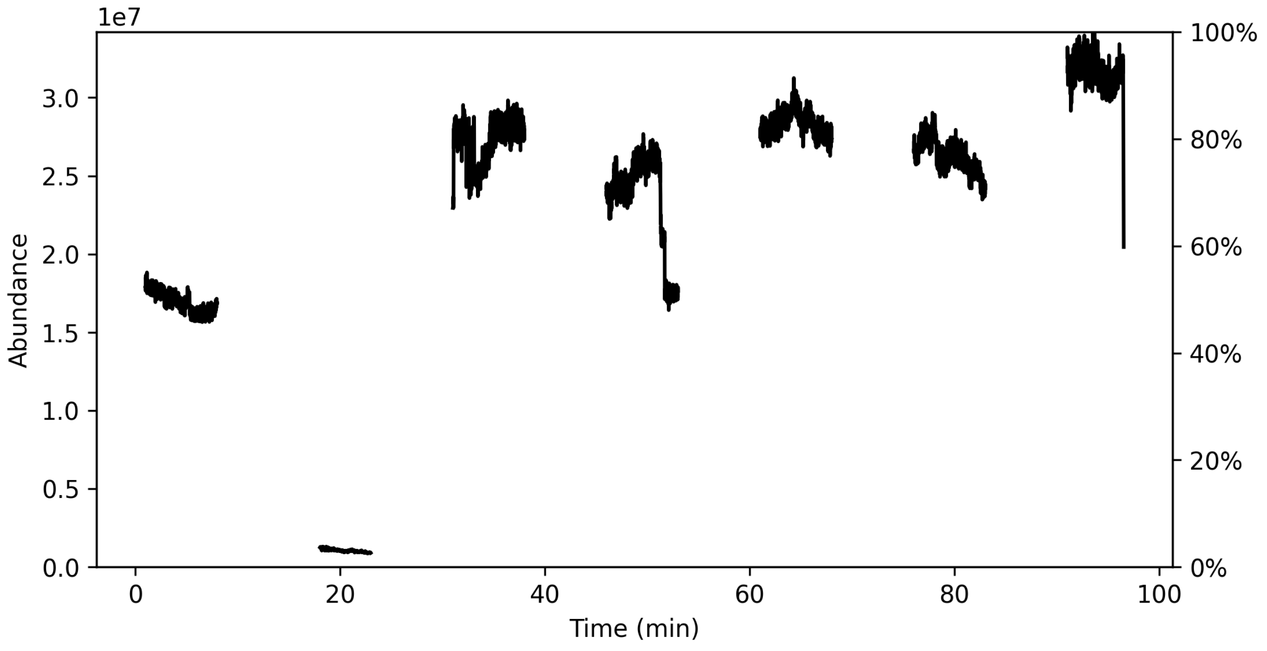

Block	TIC min	TIC max	TIC mean	RSD (%)
1	1.56e+07	1.88e+07	1.70e+07	3.90
2	8.67e+05	1.29e+06	1.04e+06	8.30
3	2.29e+07	2.98e+07	2.73e+07	4.50
4	1.64e+07	2.77e+07	2.37e+07	12.74
5	2.63e+07	3.13e+07	2.84e+07	2.74
6	2.35e+07	2.90e+07	2.63e+07	3.91
7	2.04e+07	3.42e+07	3.18e+07	2.78

2. Block Parameters

The Isotopic Ratio of the blocks were calculated by 'Mean'

2.1. 13C/M0

Block	Number of scans	Effective number of ions	Isotopic Ratio	STD	SEM	RSE
1	1318	1.70e+07	0.195106	0.001711	0.000047	0.000241
2	1083	4.30e+06	0.193104	0.003812	0.000116	0.000600
3	1266	1.64e+07	0.195825	0.001668	0.000047	0.000239
4	1313	1.59e+07	0.195994	0.001702	0.000047	0.000240
5	1266	1.66e+07	0.195929	0.001720	0.000048	0.000247
6	1280	1.57e+07	0.196379	0.001746	0.000049	0.000248
7	1153	1.52e+07	0.196177	0.001727	0.000051	0.000259

Errors and Test Paramters

Block	Acquisition Error (permil)	Shot-Noise (permil)	AE/SN ratio	Shapiro Wilk (p_value)	D'Agostino (p_value)
1	0.241	0.243	0.995	0.696	0.928
2	0.600	0.482	1.243	0.178	0.155
3	0.239	0.247	0.969	0.557	0.451
4	0.240	0.251	0.955	0.704	0.757
5	0.247	0.246	1.004	0.817	0.837
6	0.248	0.252	0.985	0.389	0.715
7	0.259	0.256	1.012	0.647	0.516

Isotopic Ratio and Errors of the Blocks

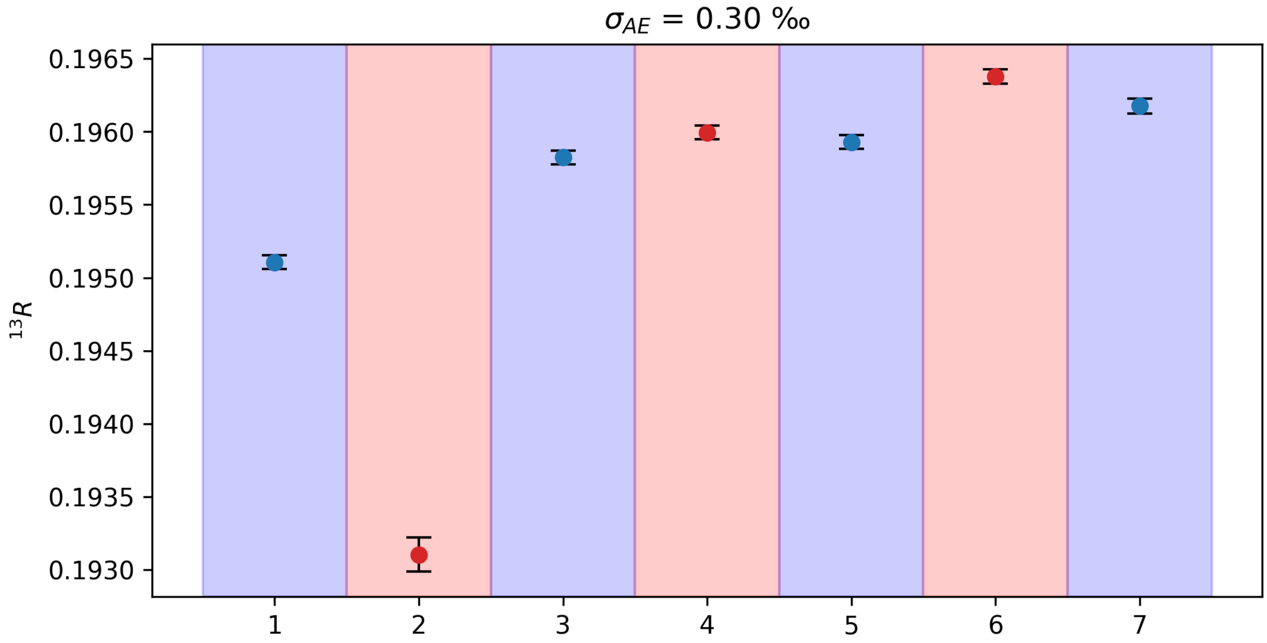

Cumulative Isotopic Ratio

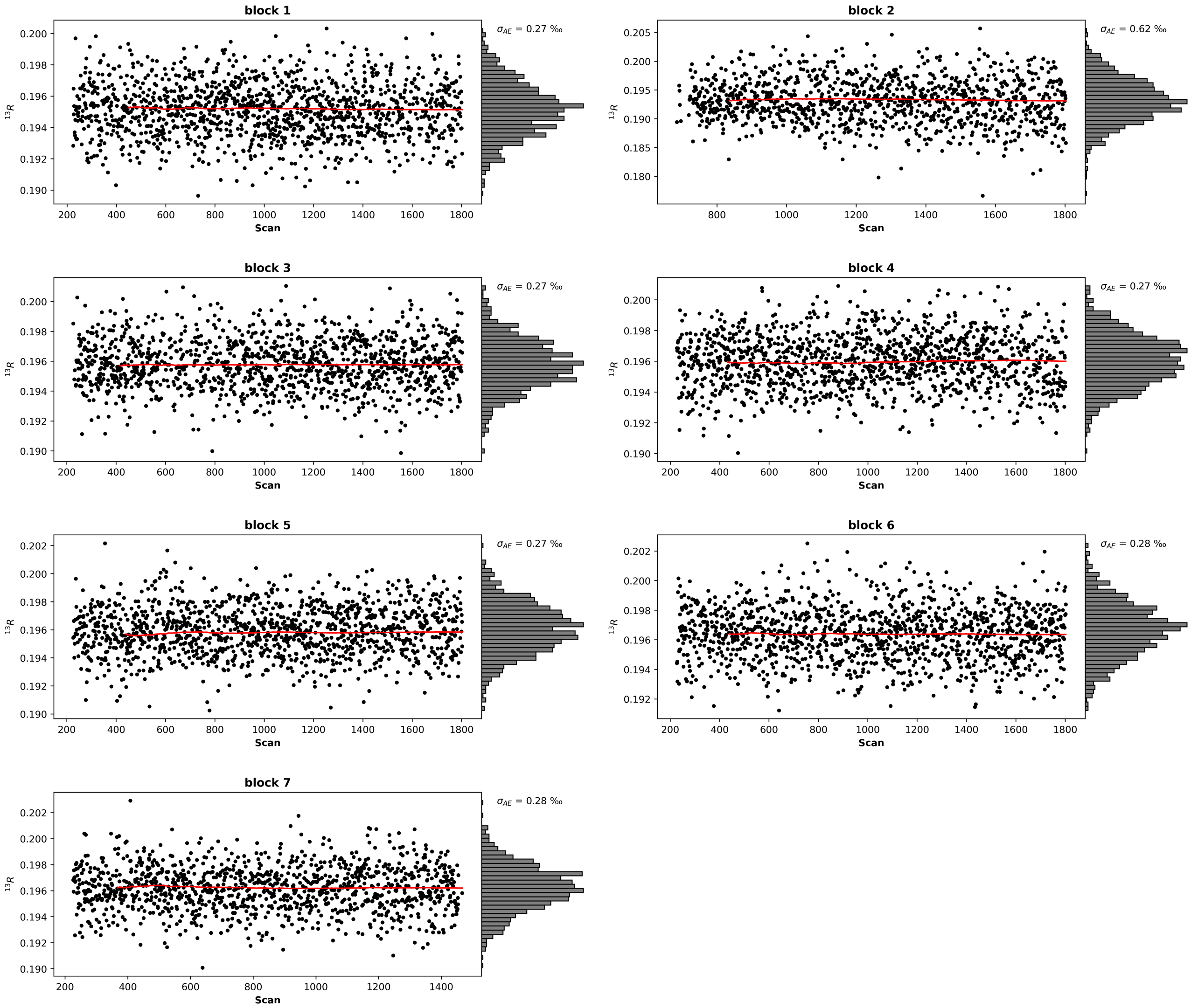

Acquisition Error and Shot-Noise

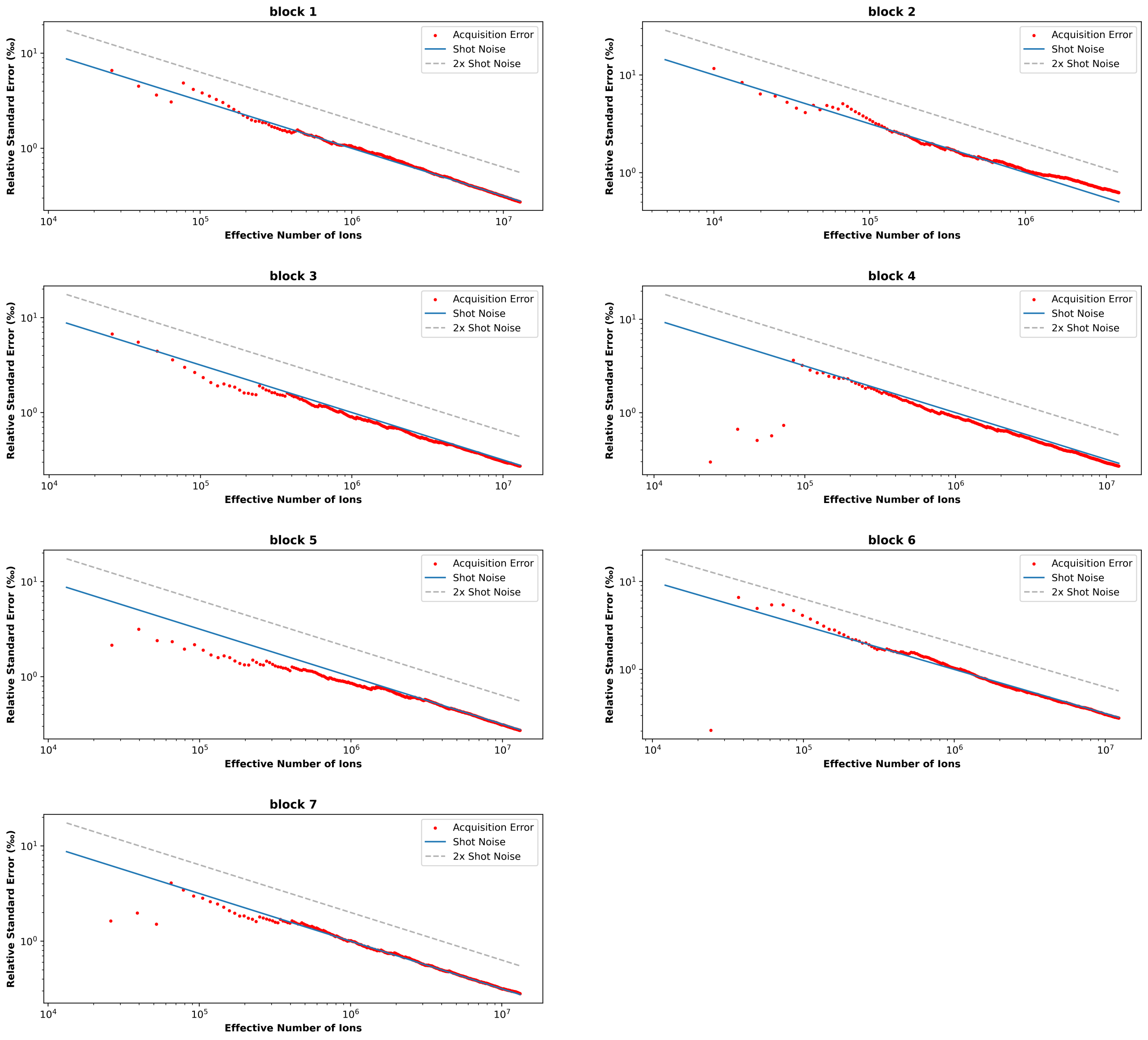

3. Delta Informations

Deltas were calculated by 'Average Of Neighboring Block Ratios'

3.1. 13C

Delta 13C was corrected by -27.80

Block	SEM	Delta corrected	Delta
2	0.59	-39.54	-12.08
4	0.24	-27.22	0.60
6	0.25	-26.18	1.66

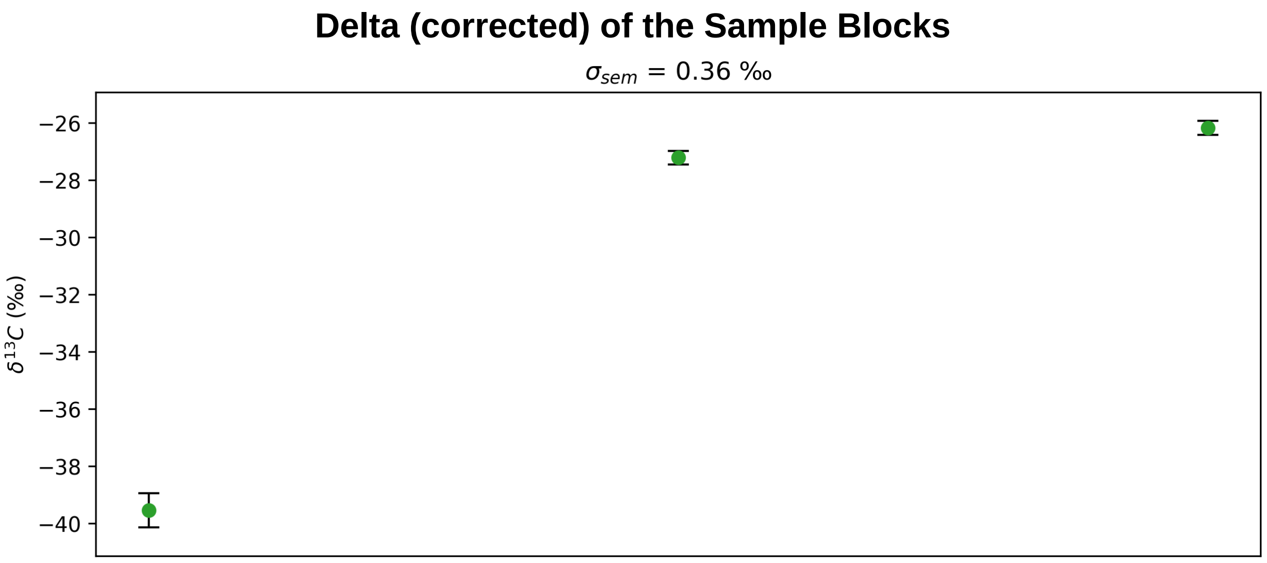

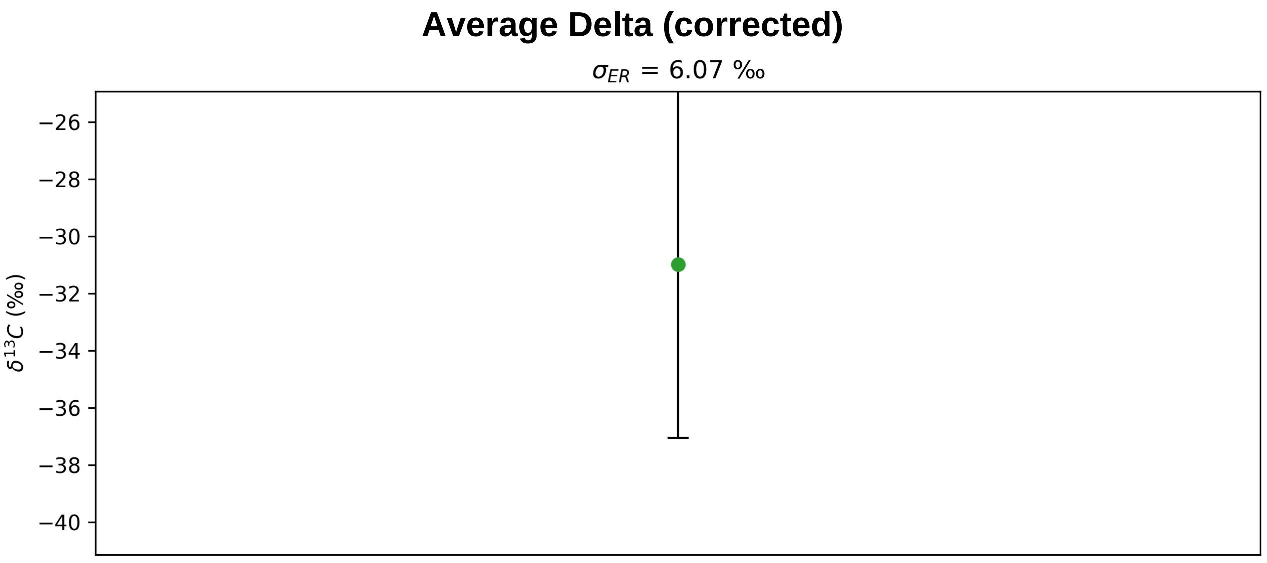

The final corrected average delta was -30.98 with a standard deviation of 6.07. Here the standard deviation is called reproducibility error.
