# Supplementary material for: Advancing Stable Isotope Analysis with Orbitrap-MS for Fatty Acid Methyl Esters and Complex Lipid Matrices
Source: J Am Soc Mass Spectrom. 2025 Jun 17;36(7):1527–35. doi: 10.1021/jasms.5c00092 (PMC12339014; doi:10.1021/jasms.5c00092)
Supplement: Supplementary file 2 [file js5c00092_si_002.zip › reports by IsotoPy Software/butters/Cupuac╠ou_rep1.pdf]

# Cupuaçu butter (replicate 1)
# Isotope Analysis report from IsotoPy

Flow Injection

## 1. Pre Processing

### 1.1. Block Time and Scan Information

Information about sample and standard block times and scans:

| Block | Injected | Initial Time | End Time | Number of scans |
|---|---|---|---|---|
| 1 | standard | 1 | 8 | 1294 |
| 2 | sample | 16 | 23 | 1290 |
| 3 | standard | 31 | 38 | 1291 |
| 4 | sample | 46 | 53 | 1295 |
| 5 | standard | 61 | 68 | 1279 |
| 6 | sample | 76 | 83 | 1285 |
| 7 | standard | 91 | 98 | 1298 |

### 1.2. Outlier Removal

A total of 2007 scans were considered outliers and removed using the MAD method

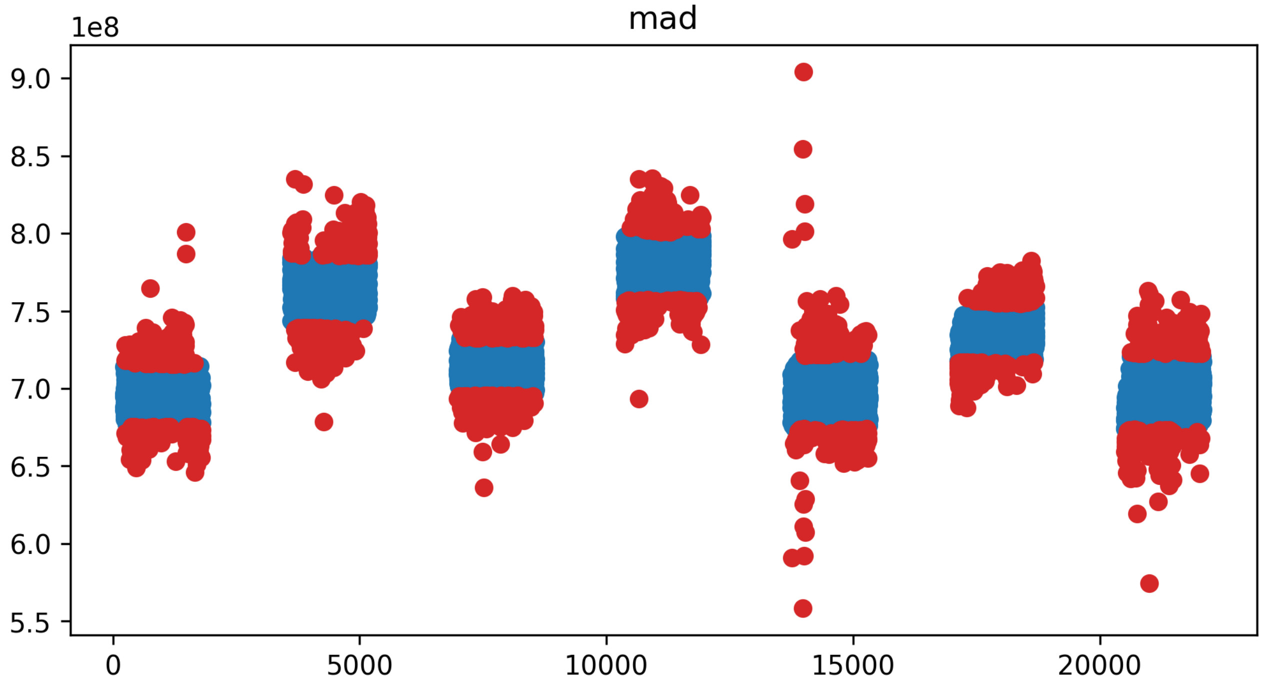

## 1.3. Total Ion Current (TIC)

TIC of all blocks

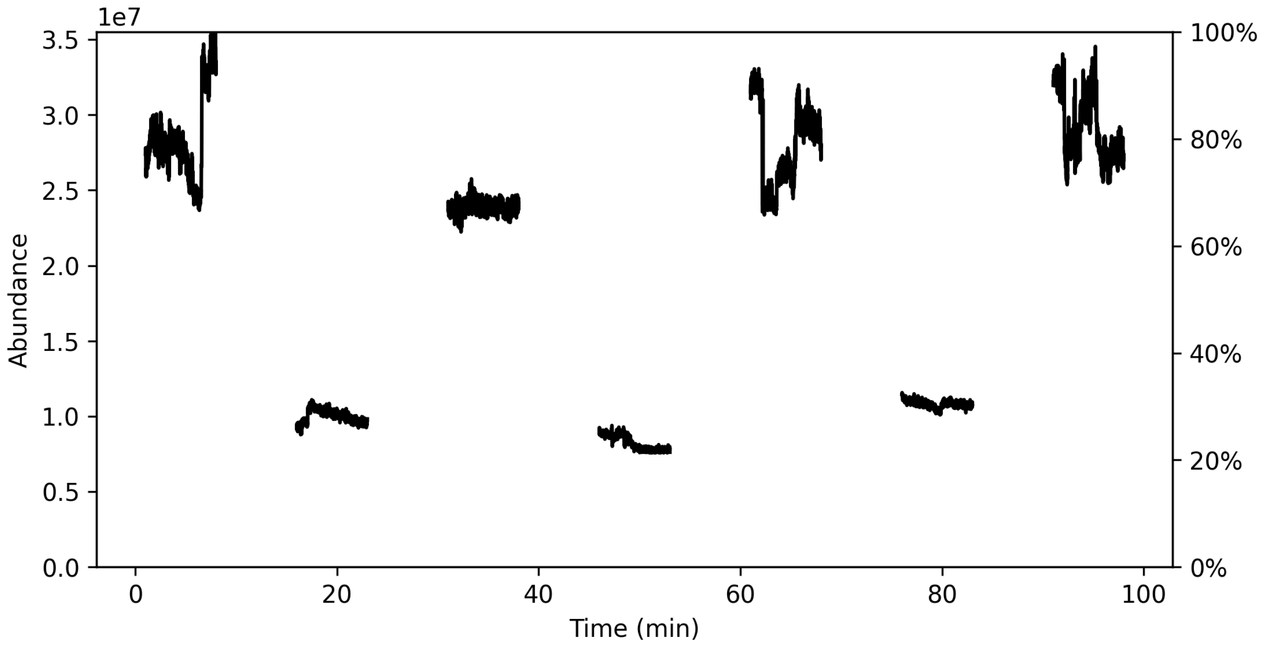


| Block | TIC min | TIC max | TIC mean | RSD (%) |
|---:|---:|---:|---:|---:|
| 1 | 2.37e+07 | 3.55e+07 | 2.84e+07 | 9.40 |
| 2 | 8.78e+06 | 1.11e+07 | 9.99e+06 | 4.17 |
| 3 | 2.22e+07 | 2.58e+07 | 2.39e+07 | 1.74 |
| 4 | 7.58e+06 | 9.38e+06 | 8.23e+06 | 6.15 |
| 5 | 2.34e+07 | 3.31e+07 | 2.82e+07 | 9.46 |
| 6 | 1.01e+07 | 1.16e+07 | 1.08e+07 | 2.09 |
| 7 | 2.54e+07 | 3.45e+07 | 2.89e+07 | 7.22 |

# 2. Block Parameters

The Isotopic Ratio of the blocks were calculated by 'Mean'

## 2.1. 13C/M0

| Block | Number of scans | Effective number of ions | Isotopic Ratio | STD | SEM | RSE |
|---|---|---|---|---|---|---|
| 1 | 1294 | 1.69e+07 | 0.197053 | 0.001685 | 0.000047 | 0.000238 |
| 2 | 1290 | 1.58e+07 | 0.196815 | 0.001700 | 0.000047 | 0.000240 |
| 3 | 1291 | 1.70e+07 | 0.196864 | 0.001701 | 0.000047 | 0.000240 |
| 4 | 1295 | 1.57e+07 | 0.197301 | 0.001837 | 0.000051 | 0.000259 |
| 5 | 1279 | 1.68e+07 | 0.197185 | 0.001722 | 0.000048 | 0.000244 |
| 6 | 1285 | 1.59e+07 | 0.196765 | 0.001655 | 0.000046 | 0.000234 |
| 7 | 1298 | 1.72e+07 | 0.196988 | 0.001616 | 0.000045 | 0.000228 |

**Errors and Test Paramters**

| Block | Acquisition Error (permil) | Shot-Noise (permil) | AE/SN ratio | Shapiro Wilk (p_value) | D'Agostino (p_value) |
|---|---|---|---|---|---|
| 1 | 0.238 | 0.243 | 0.978 | 0.611 | 0.376 |
| 2 | 0.240 | 0.251 | 0.956 | 0.681 | 0.524 |
| 3 | 0.240 | 0.243 | 0.990 | 0.233 | 0.531 |
| 4 | 0.259 | 0.252 | 1.026 | 0.709 | 0.406 |
| 5 | 0.244 | 0.244 | 1.001 | 0.283 | 0.183 |
| 6 | 0.234 | 0.251 | 0.935 | 0.735 | 0.915 |
| 7 | 0.228 | 0.241 | 0.945 | 0.352 | 0.355 |

# Isotopic Ratio and Errors of the Blocks

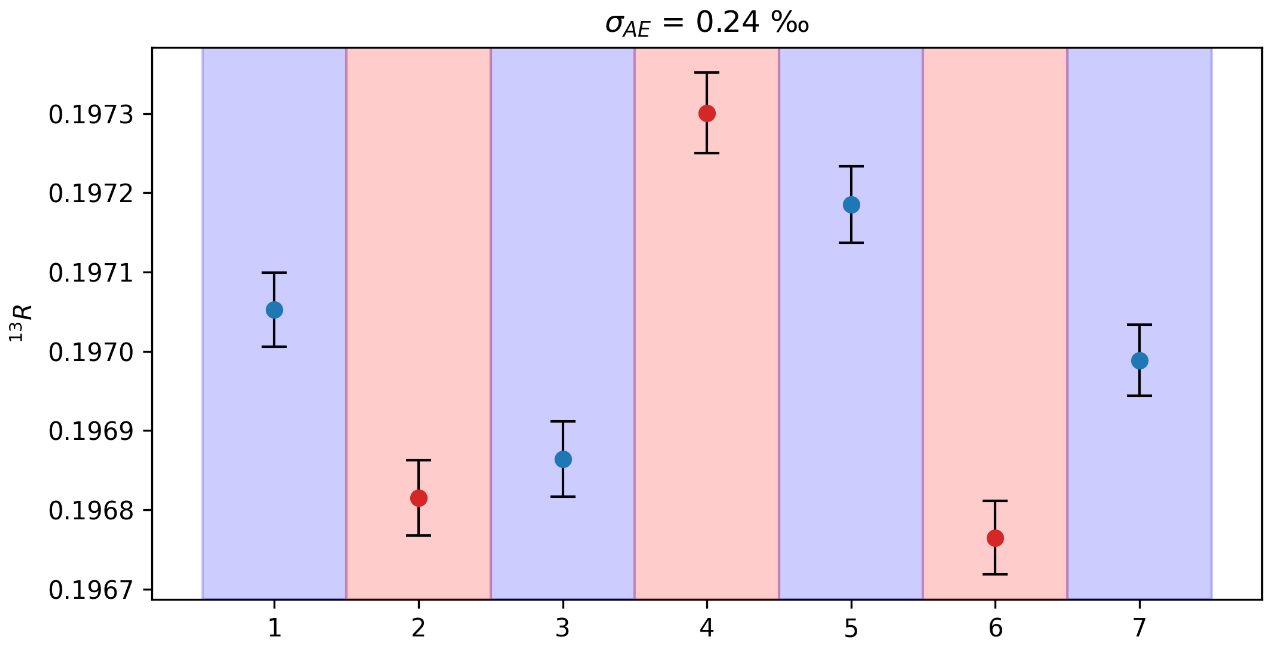


# Cumulative Isotopic Ratio

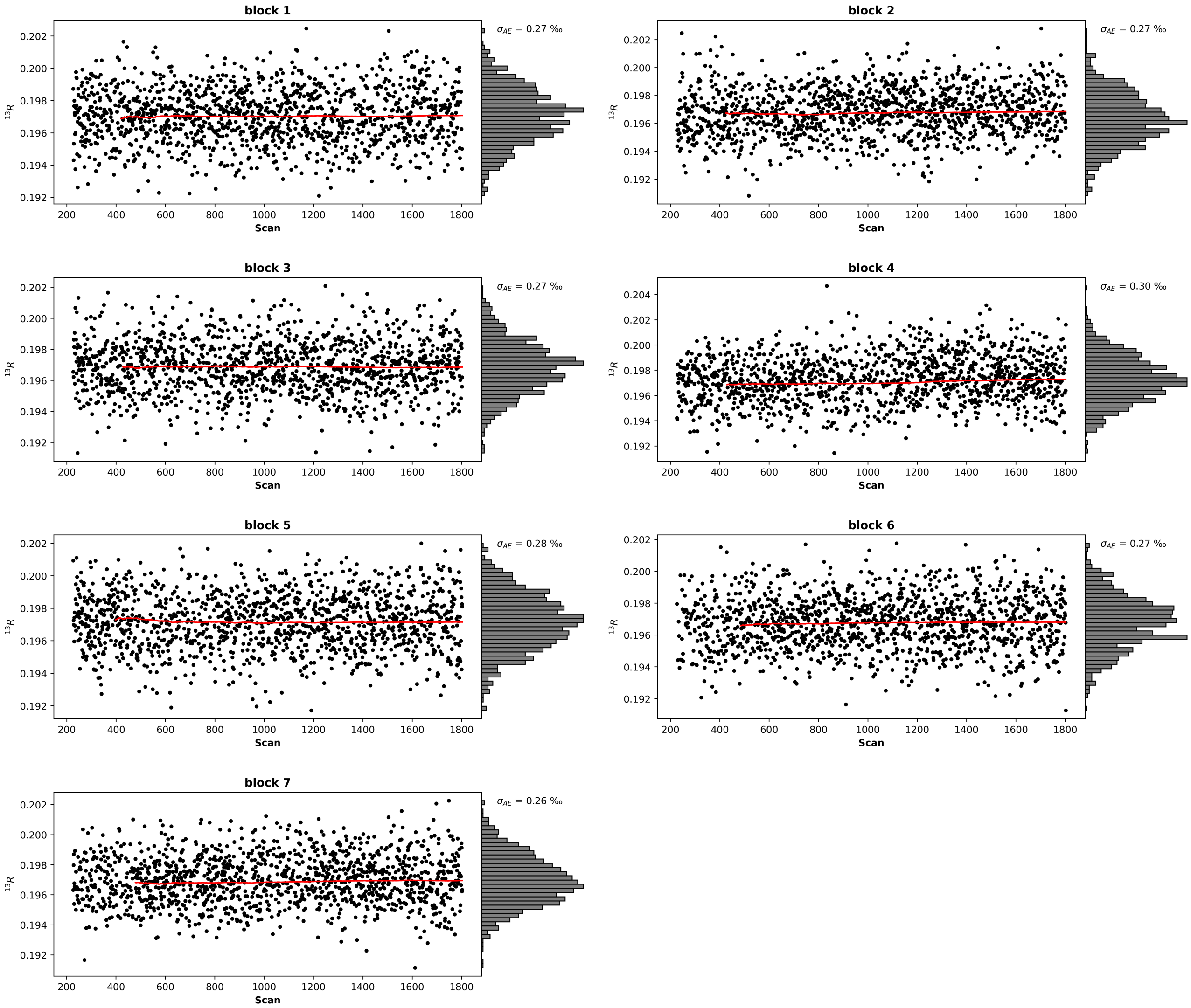

# Acquisition Error and Shot-Noise

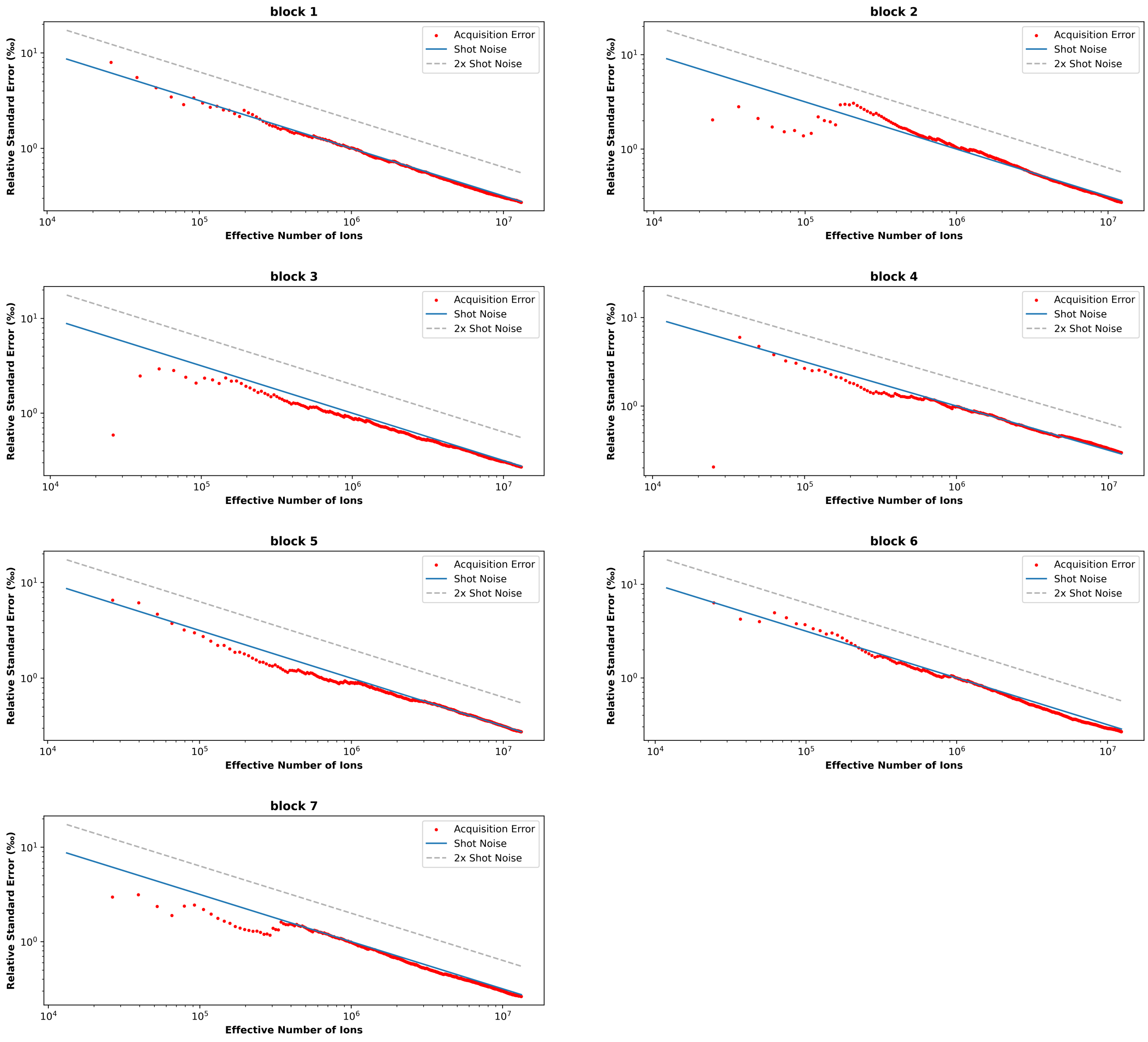

# 3. Delta Informations

Deltas were calculated by 'Average Of Neighboring Block Ratios'

## 3.1. 13C

Delta 13C was corrected by -27.80

| Block | SEM | Delta corrected | Delta |
|---|---|---|---|
| 2 | 0.24 | -28.51 | -0.73 |
| 4 | 0.26 | -26.44 | 1.40 |
| 6 | 0.23 | -29.39 | -1.63 |

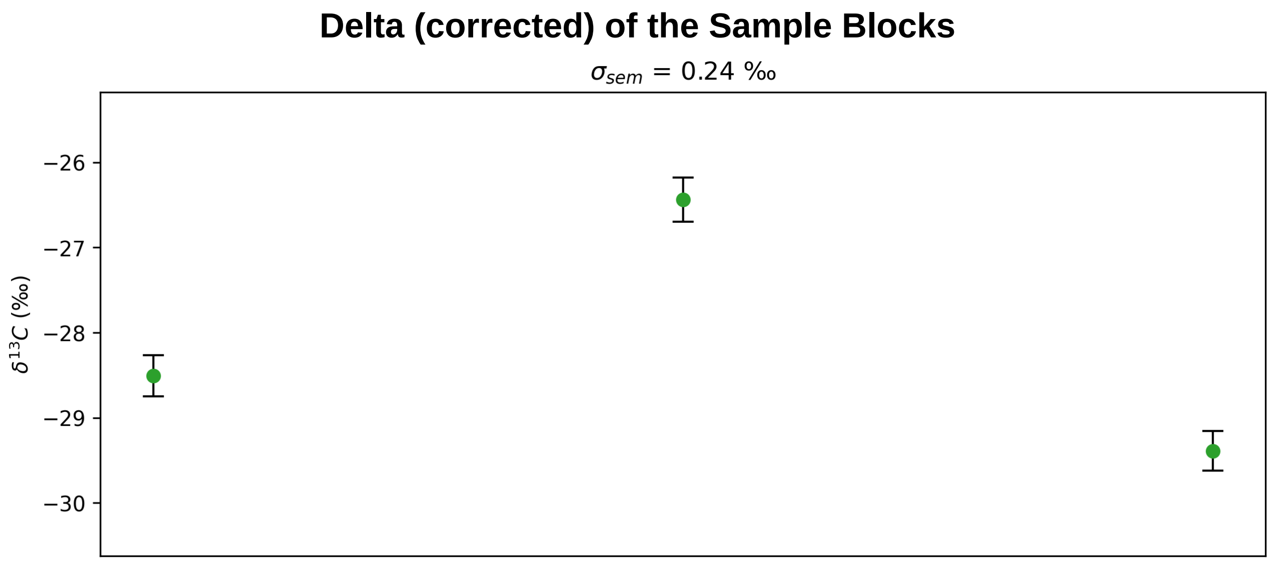


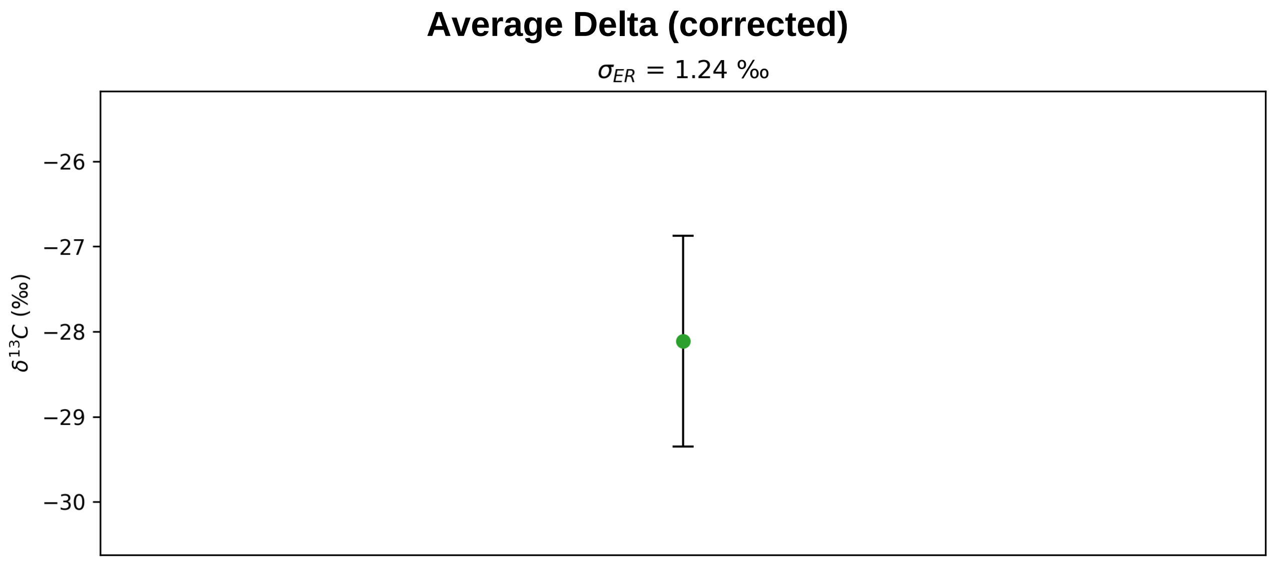


The final corrected average delta was -28.11 with a standard deviation of 1.24. Here the standard deviation is called reproducibility error.
