# Supplementary material for: Advancing Stable Isotope Analysis with Orbitrap-MS for Fatty Acid Methyl Esters and Complex Lipid Matrices
Source: J Am Soc Mass Spectrom. 2025 Jun 17;36(7):1527–35. doi: 10.1021/jasms.5c00092 (PMC12339014; doi:10.1021/jasms.5c00092)
Supplement: Supplementary file 2 [file js5c00092_si_002.zip › reports by IsotoPy Software/butters/Cocoa_rep1.pdf]

Cocoa butter (replicate 1)
Isotope Analysis report from IsotoPy

Flow Injection

1. Pre Processing

1.1. Block Time and Scan Information

Information about sample and standard block times and scans:

Block	Injected	Initial Time	End Time	Number of scans
1	standard	1	8	1298
2	sample	16	23	1343
3	standard	31	38	1295
4	sample	46	53	1317
5	standard	61	68	1283
6	sample	76	83	1298
7	standard	91	98	1327

1.2. Outlier Removal

A total of 1878 scans were considered outliers and removed using the MAD method

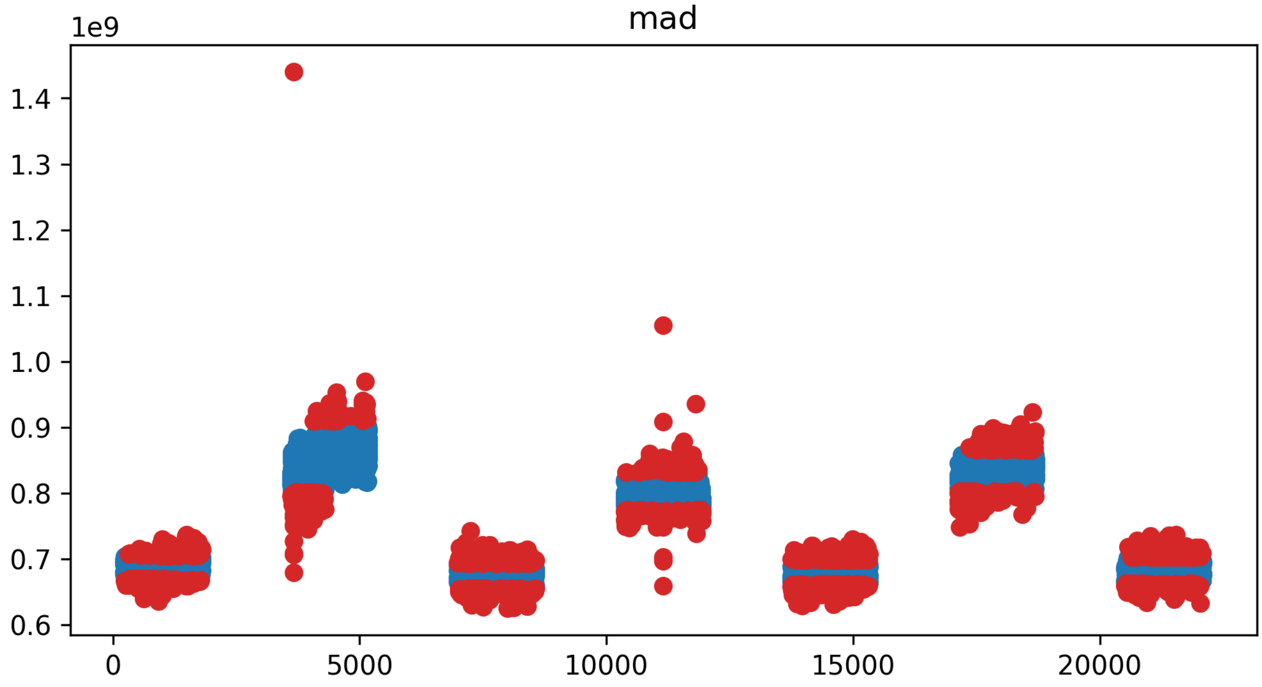

1.3. Total Ion Current (TIC)

TIC of all blocks

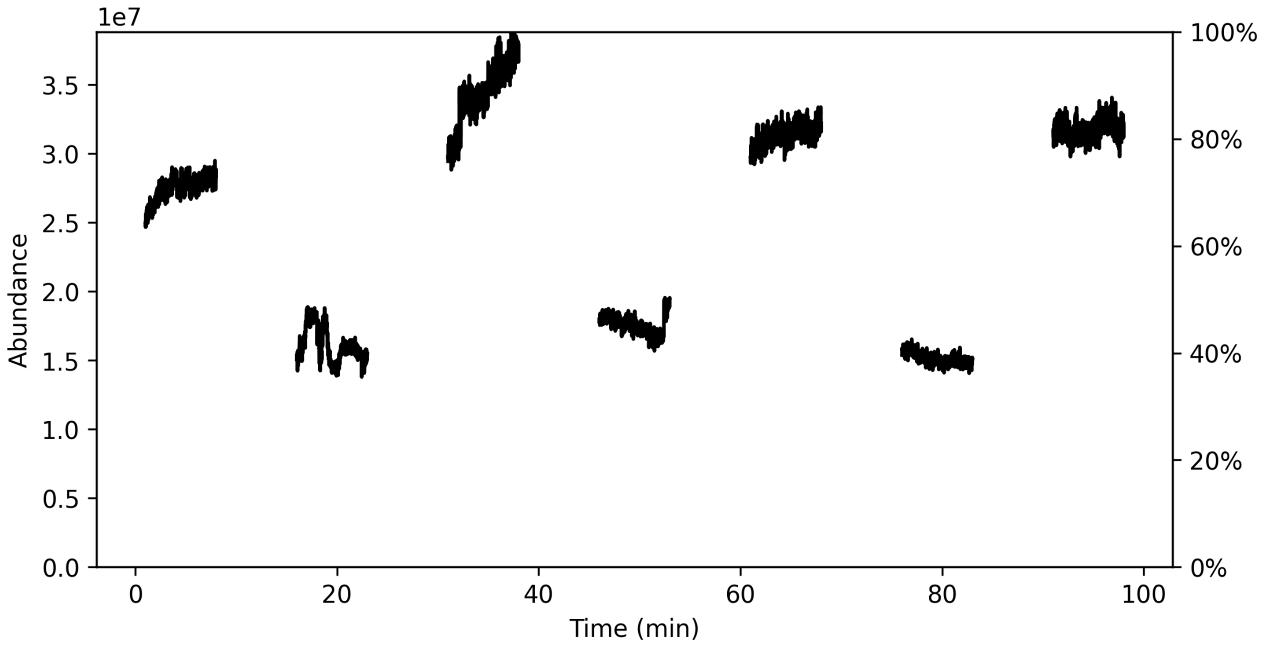

Block	TIC min	TIC max	TIC mean	RSD (%)
1	2.47e+07	2.95e+07	2.75e+07	3.33
2	1.38e+07	1.89e+07	1.60e+07	7.23
3	2.88e+07	3.88e+07	3.45e+07	6.52
4	1.57e+07	1.95e+07	1.76e+07	3.90
5	2.92e+07	3.34e+07	3.14e+07	2.30
6	1.41e+07	1.65e+07	1.51e+07	2.71
7	2.98e+07	3.41e+07	3.18e+07	2.14

2. Block Parameters

The Isotopic Ratio of the blocks were calculated by 'Mean'

2.1. 13C/M0

Block	Number of scans	Effective number of ions	Isotopic Ratio	STD	SEM	RSE
1	1298	1.67e+07	0.196540	0.001635	0.000045	0.000231
2	1343	1.58e+07	0.196820	0.001750	0.000048	0.000243
3	1295	1.67e+07	0.196842	0.001658	0.000046	0.000234
4	1317	1.57e+07	0.196990	0.001712	0.000047	0.000239
5	1283	1.65e+07	0.196981	0.001706	0.000048	0.000242
6	1298	1.54e+07	0.197003	0.001765	0.000049	0.000249
7	1327	1.71e+07	0.197089	0.001700	0.000047	0.000237

Errors and Test Paramters

Block	Acquisition Error (permil)	Shot-Noise (permil)	AE/SN ratio	Shapiro Wilk (p_value)	D'Agostino (p_value)
1	0.231	0.244	0.944	0.601	0.627
2	0.243	0.252	0.964	0.176	0.356
3	0.234	0.245	0.955	0.111	0.541
4	0.239	0.252	0.948	0.474	0.248
5	0.242	0.246	0.983	0.872	0.947
6	0.249	0.255	0.974	0.708	0.417
7	0.237	0.242	0.978	0.078	0.162

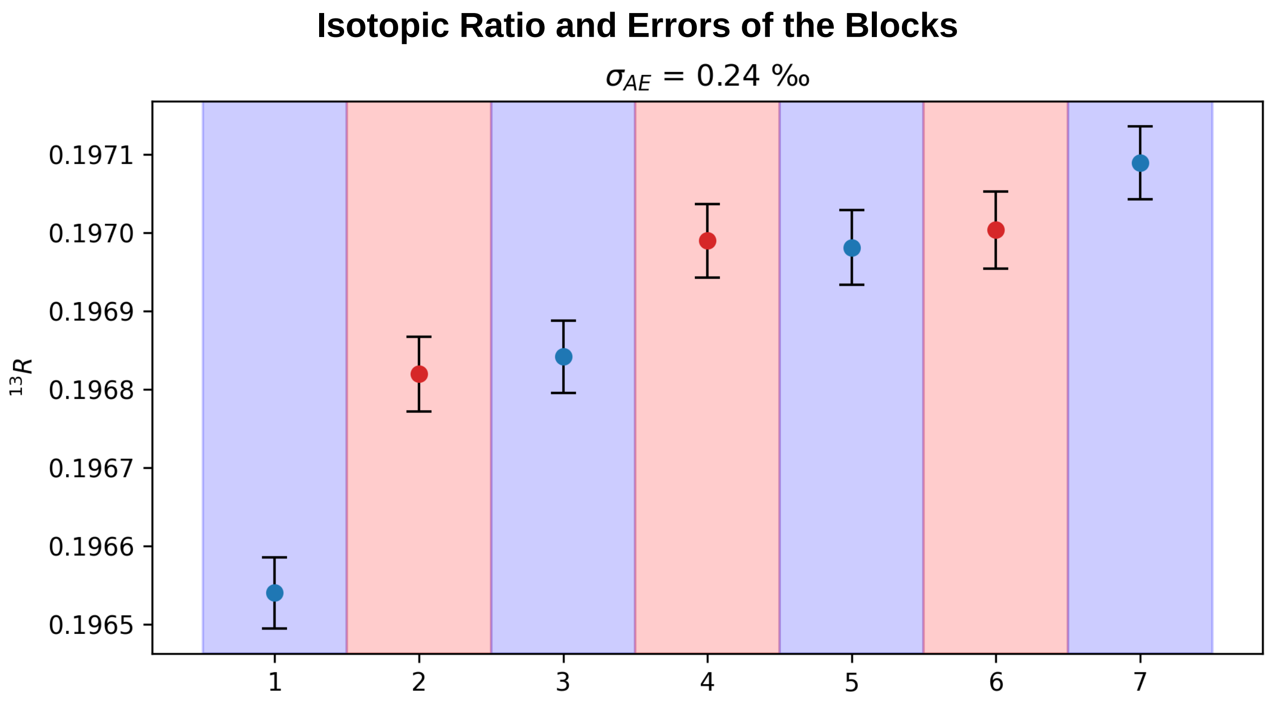

Isotopic Ratio and Errors of the Blocks
σAE = 0.24 ‰
13R
0.1965
0.1966
0.1967
0.1968
0.1969
0.1970
0.1971
1
2
3
4
5
6
7

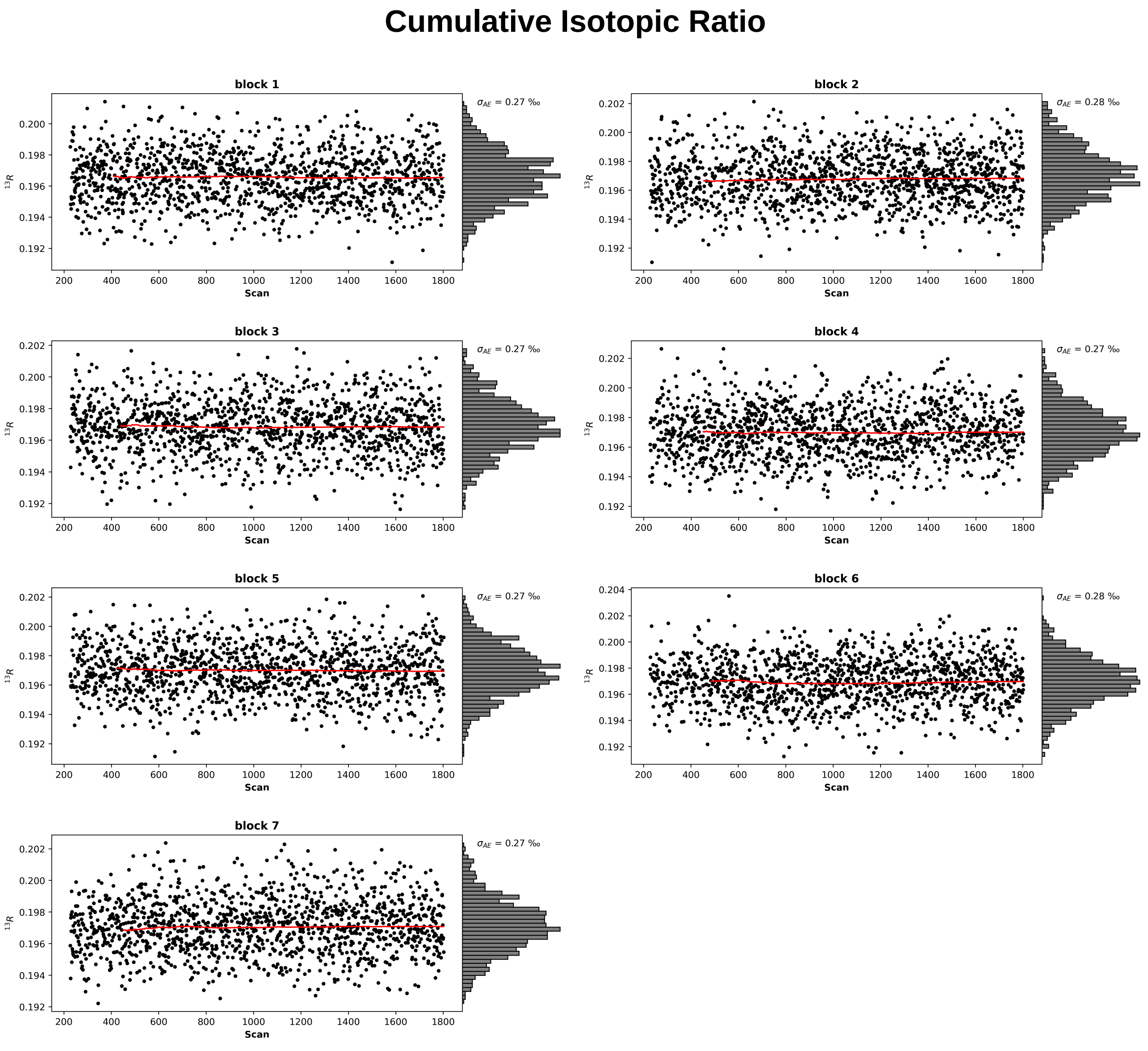

Cumulative Isotopic Ratio
block 1
σAE = 0.27 ‰
block 2
σAE = 0.28 ‰
block 3
σAE = 0.27 ‰
block 4
σAE = 0.27 ‰
block 5
σAE = 0.27 ‰
block 6
σAE = 0.28 ‰
block 7
σAE = 0.27 ‰
13R
Scan

Acquisition Error and Shot-Noise

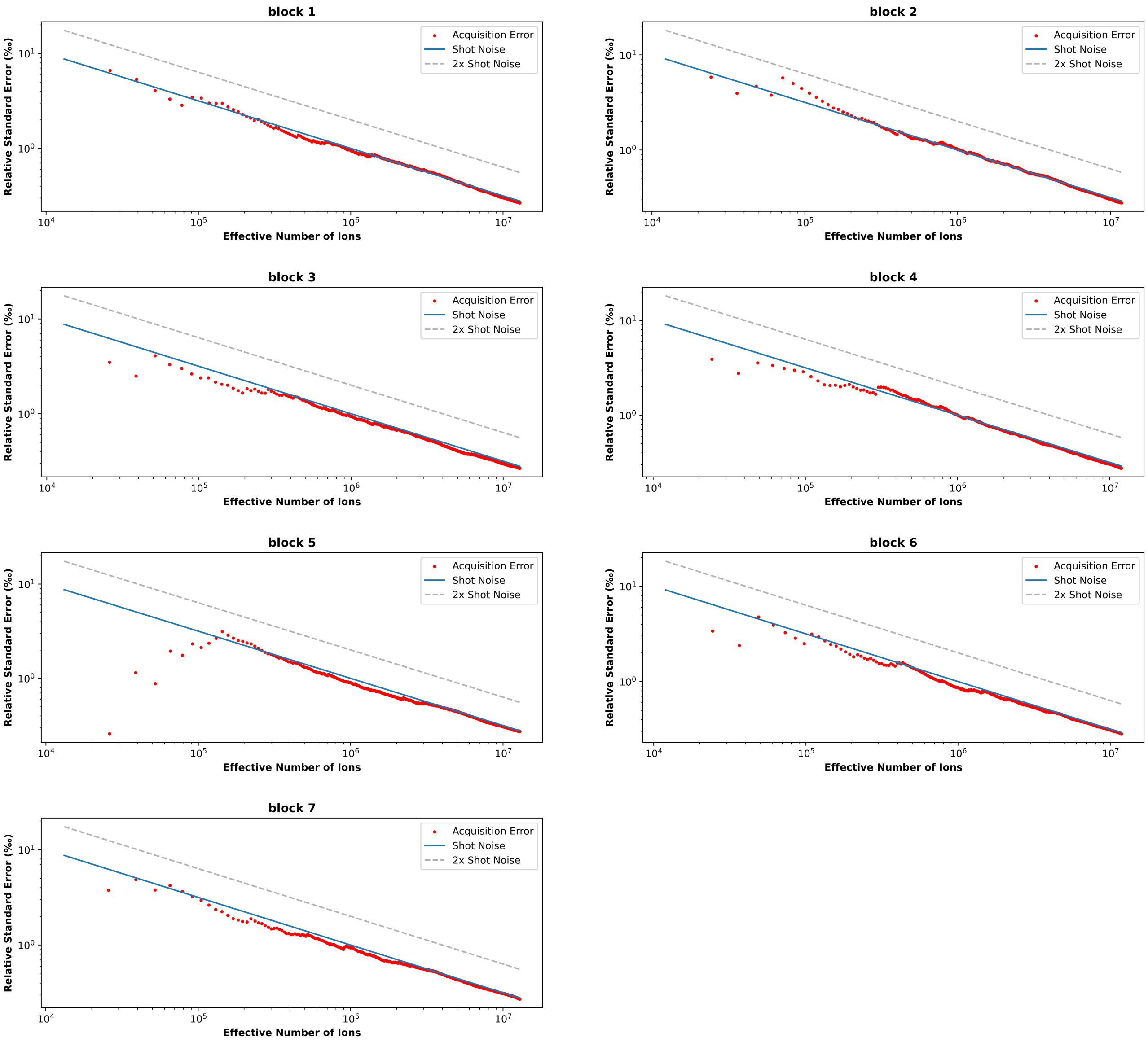

3. Delta Informations

Deltas were calculated by 'Average Of Neighboring Block Ratios'

3.1. 13C

Delta 13C was corrected by -27.80

Block	SEM	Delta corrected	Delta
2	0.24	-27.16	0.65
4	0.24	-27.41	0.40
6	0.25	-27.96	-0.16

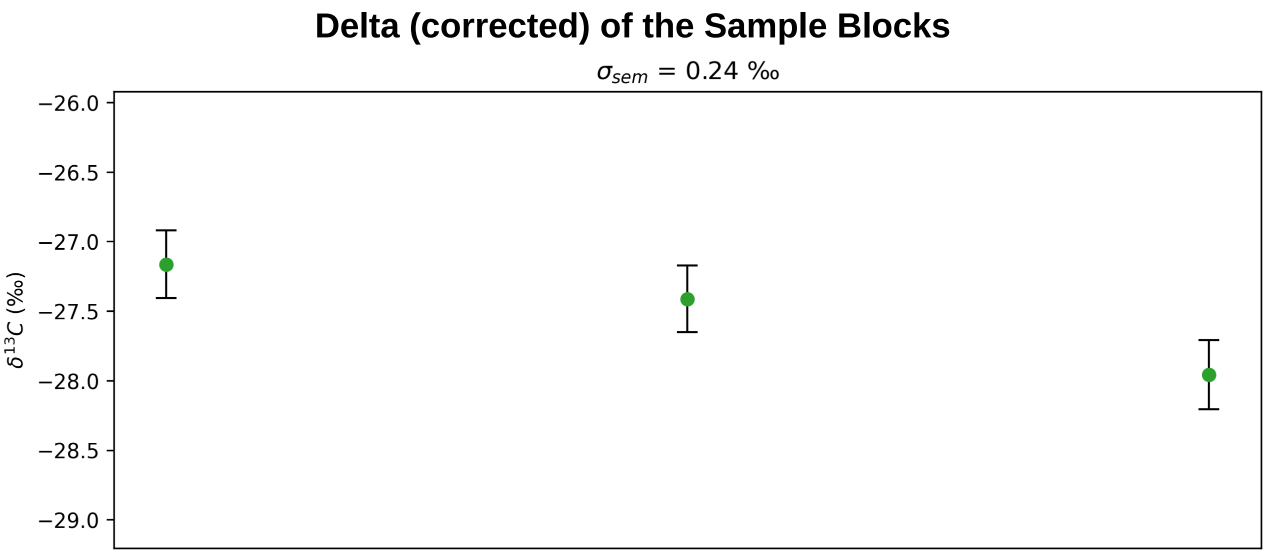

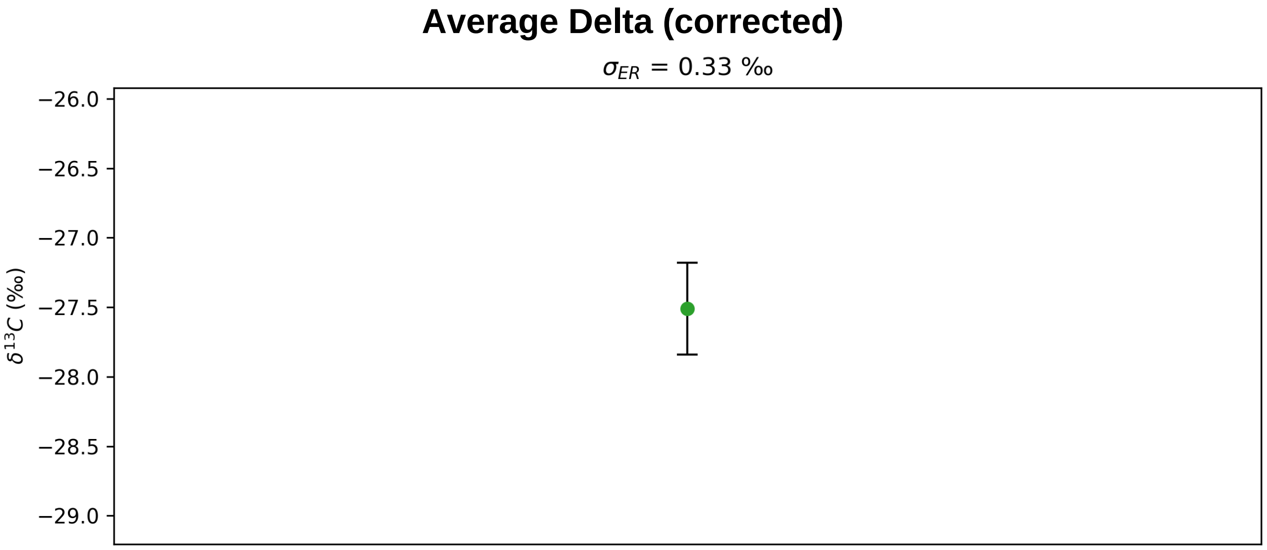

The final corrected average delta was -27.51 with a standard deviation of 0.33. Here the standard deviation is called reproducibility error.
