# Supplementary material for: Advancing Stable Isotope Analysis with Orbitrap-MS for Fatty Acid Methyl Esters and Complex Lipid Matrices
Source: J Am Soc Mass Spectrom. 2025 Jun 17;36(7):1527–35. doi: 10.1021/jasms.5c00092 (PMC12339014; doi:10.1021/jasms.5c00092)
Supplement: Supplementary file 2 [file js5c00092_si_002.zip › reports by IsotoPy Software/butters/Shea_rep1.pdf]

Shea butter (replicate 1)
Isotope Analysis report from IsotoPy
Flow Injection

1. Pre Processing

1.1. Block Time and Scan Information

Information about sample and standard block times and scans:

Block	Injected	Initial Time	End Time	Number of scans
1	standard	1	8	1387
2	sample	16	23	1290
3	standard	31	38	1277
4	sample	46	53	1296
5	standard	61	68	1289
6	sample	76	83	1318
7	standard	91	98	1302

1.2. Outlier Removal

A total of 1880 scans were considered outliers and removed using the MAD method

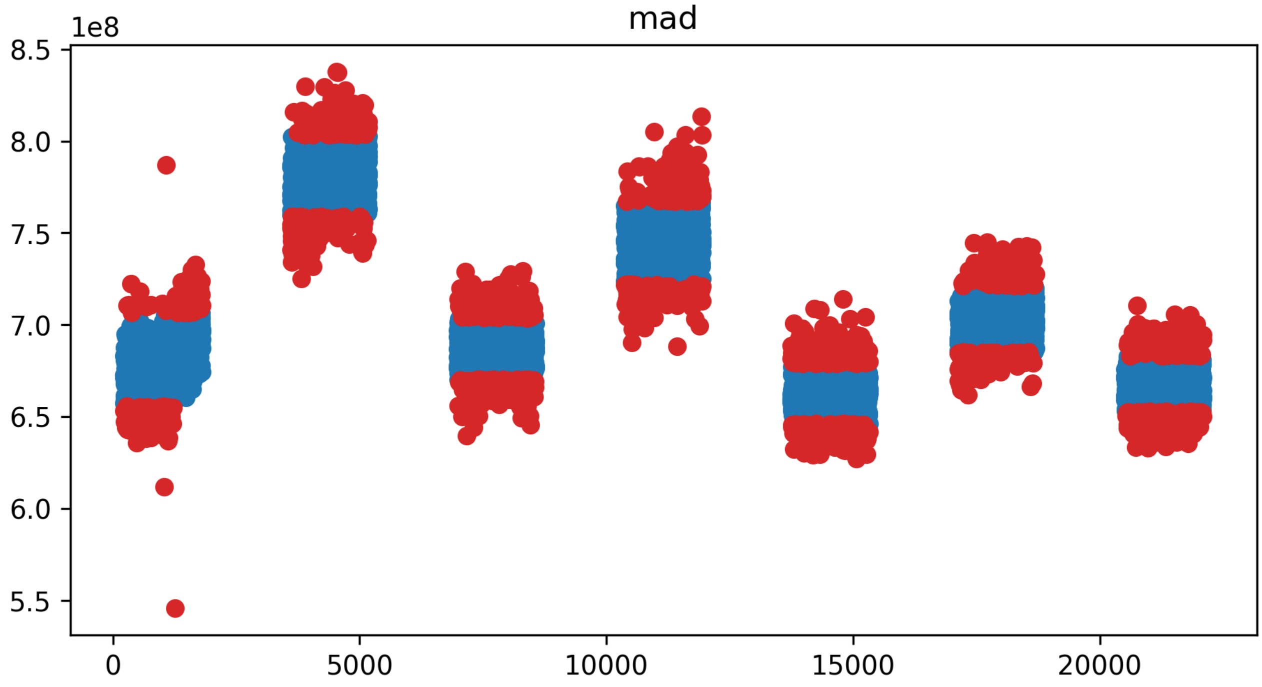

1.3. Total Ion Current (TIC)

TIC of all blocks

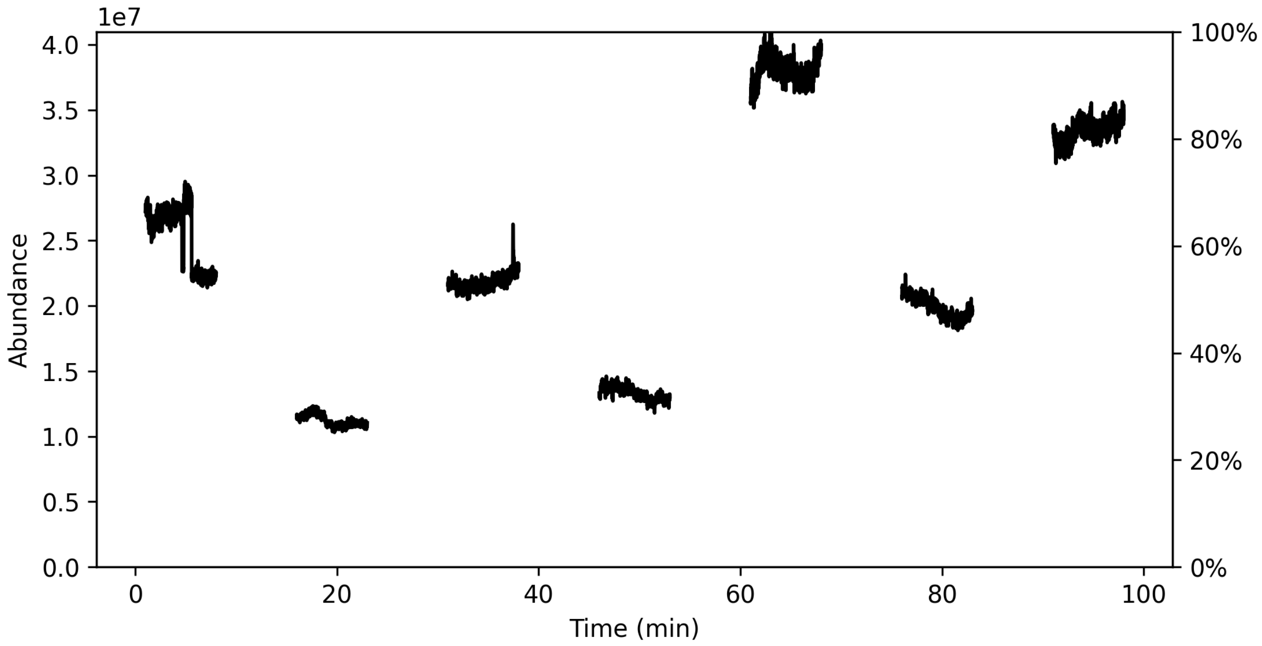

Block	TIC min	TIC max	TIC mean	RSD (%)
1	2.14e+07	2.95e+07	2.54e+07	9.20
2	1.03e+07	1.23e+07	1.12e+07	3.82
3	2.05e+07	2.62e+07	2.18e+07	2.65
4	1.18e+07	1.46e+07	1.33e+07	3.63
5	3.52e+07	4.10e+07	3.81e+07	2.61
6	1.81e+07	2.24e+07	1.99e+07	3.88
7	3.09e+07	3.56e+07	3.34e+07	2.43

2. Block Parameters

The Isotopic Ratio of the blocks were calculated by 'Mean'

2.1. 13C/M0

Block	Number of scans	Effective number of ions	Isotopic Ratio	STD	SEM	RSE
1	1387	1.80e+07	0.197000	0.001698	0.000046	0.000231
2	1290	1.64e+07	0.196676	0.001665	0.000046	0.000236
3	1277	1.66e+07	0.197028	0.001643	0.000046	0.000233
4	1296	1.65e+07	0.196971	0.001729	0.000048	0.000244
5	1289	1.68e+07	0.197541	0.001688	0.000047	0.000238
6	1318	1.68e+07	0.197051	0.001664	0.000046	0.000233
7	1302	1.69e+07	0.197377	0.001660	0.000046	0.000233

Errors and Test Paramters

Block	Acquisition Error (permil)	Shot-Noise (permil)	AE/SN ratio	Shapiro Wilk (p_value)	D'Agostino (p_value)
1	0.231	0.236	0.982	0.710	0.679
2	0.236	0.247	0.954	0.553	0.727
3	0.233	0.245	0.951	0.192	0.126
4	0.244	0.246	0.991	0.859	0.532
5	0.238	0.244	0.974	0.186	0.347
6	0.233	0.244	0.953	0.160	0.147
7	0.233	0.243	0.958	0.632	0.689

Isotopic Ratio and Errors of the Blocks

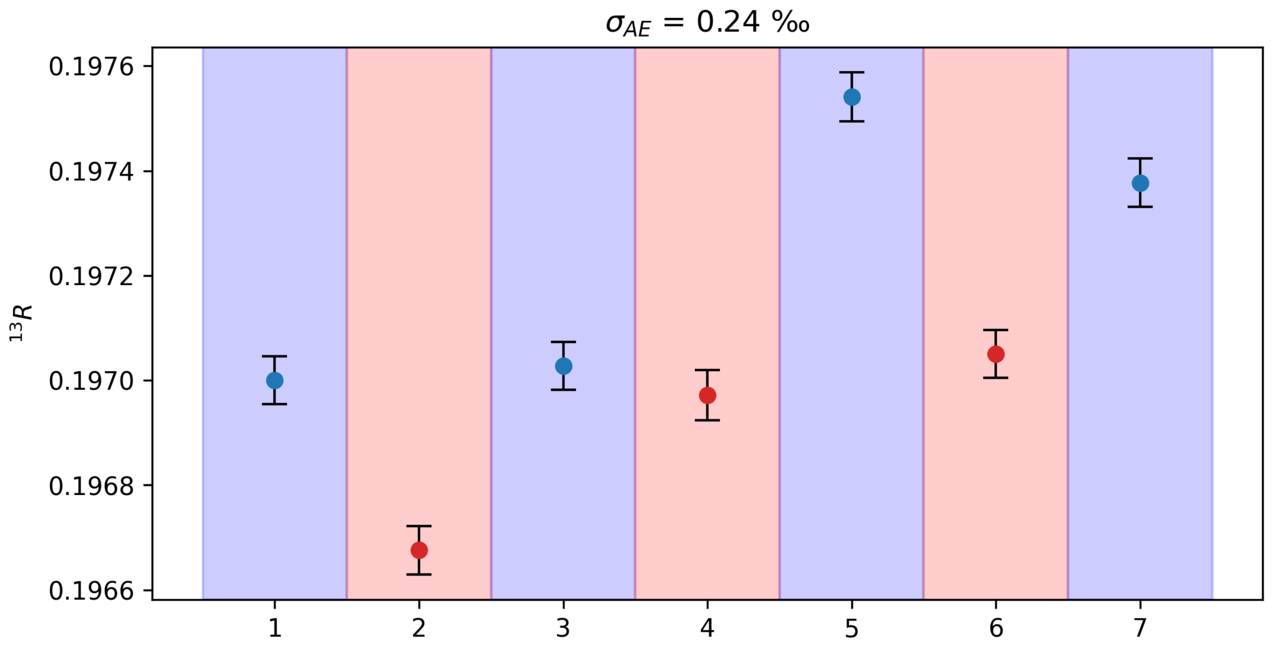

Cumulative Isotopic Ratio

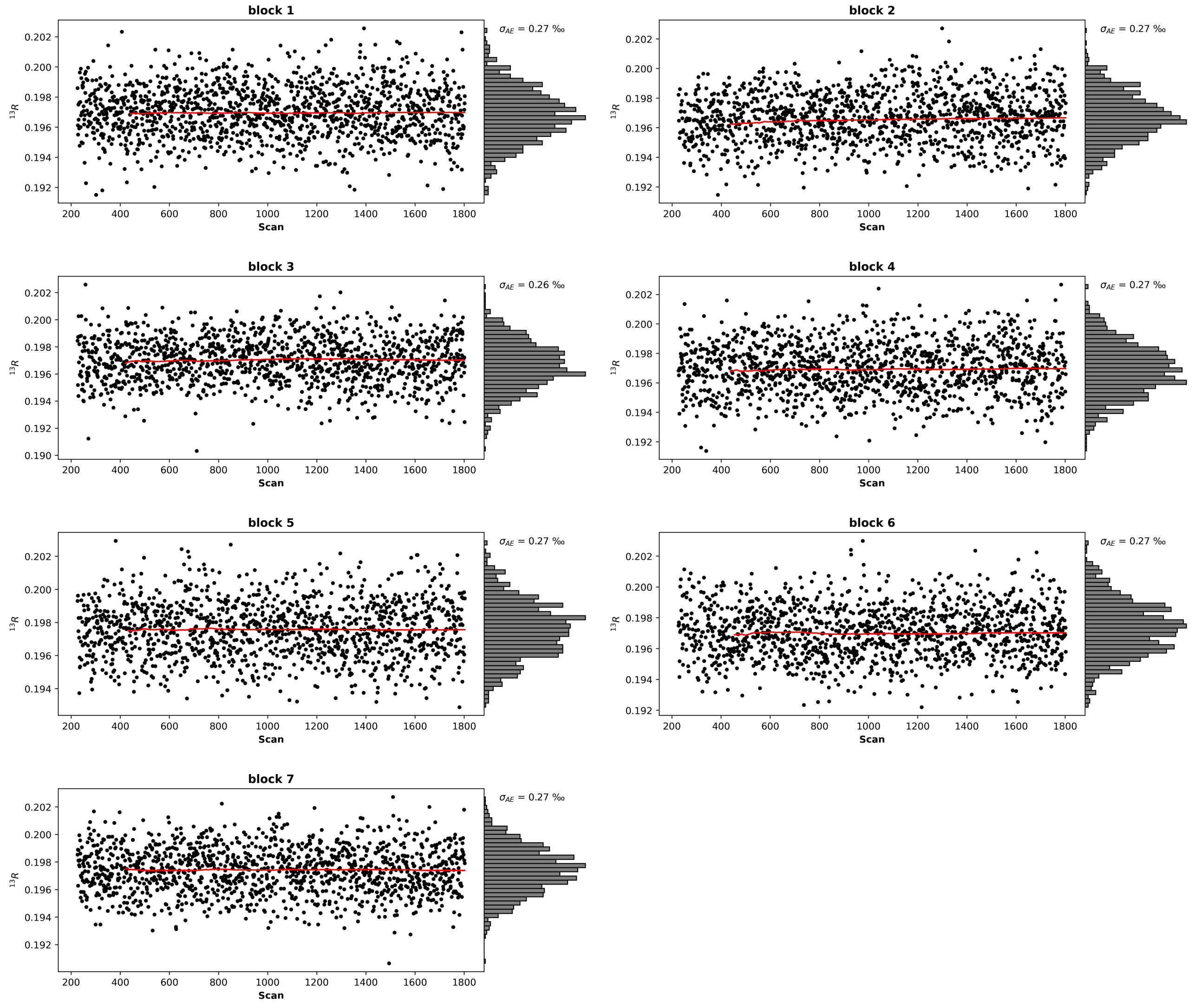

Acquisition Error and Shot-Noise

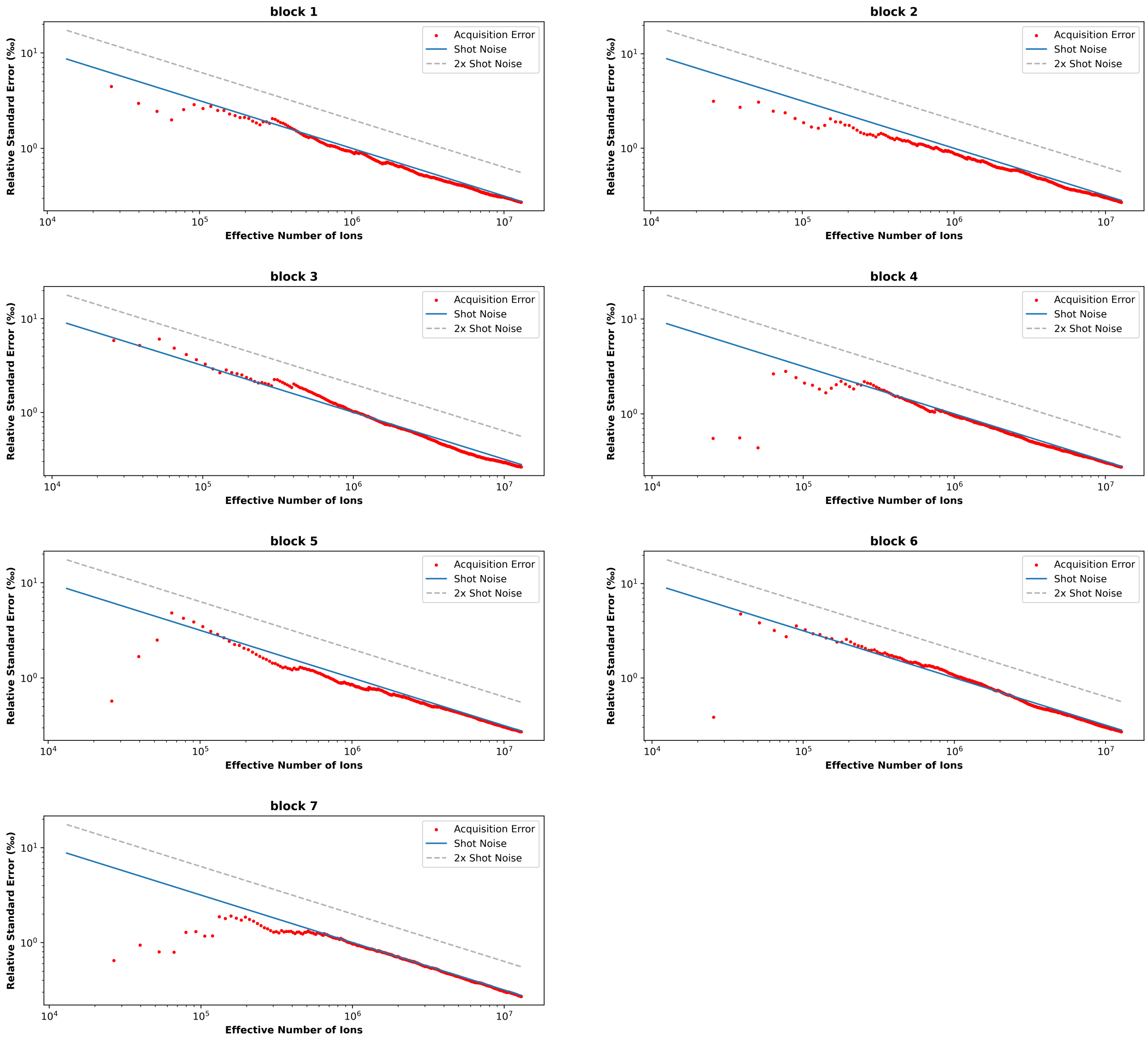

3. Delta Informations

Deltas were calculated by 'Average Of Neighboring Block Ratios'

3.1. 13C

Delta 13C was corrected by -27.80

Block	SEM	Delta corrected	Delta
2	0.24	-29.47	-1.72
4	0.24	-29.34	-1.59
6	0.23	-29.81	-2.07

Delta (corrected) of the Sample Blocks

σ_{sem} = 0.24 ‰

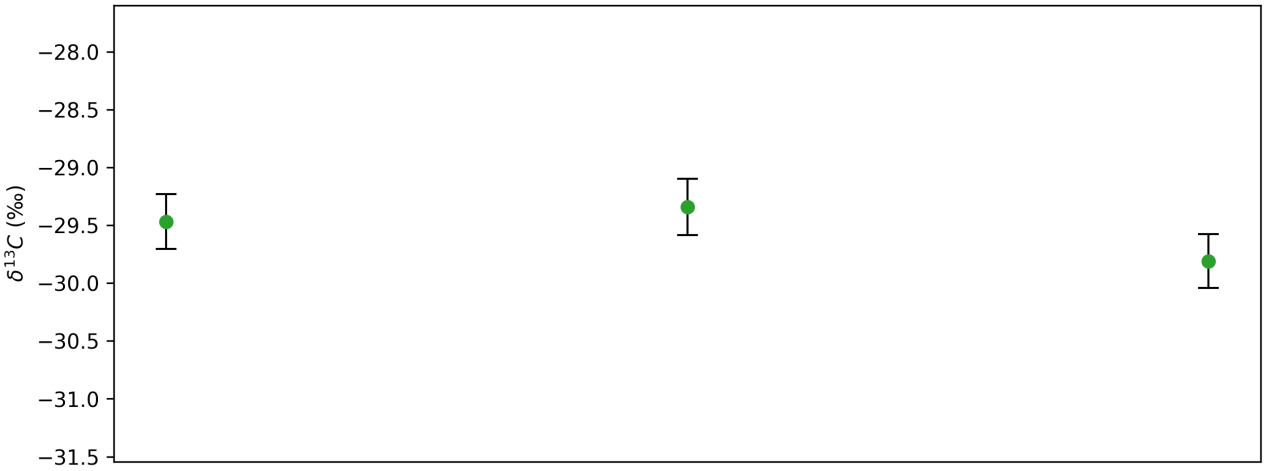

Average Delta (corrected)

σ_{ER} = 0.20 ‰

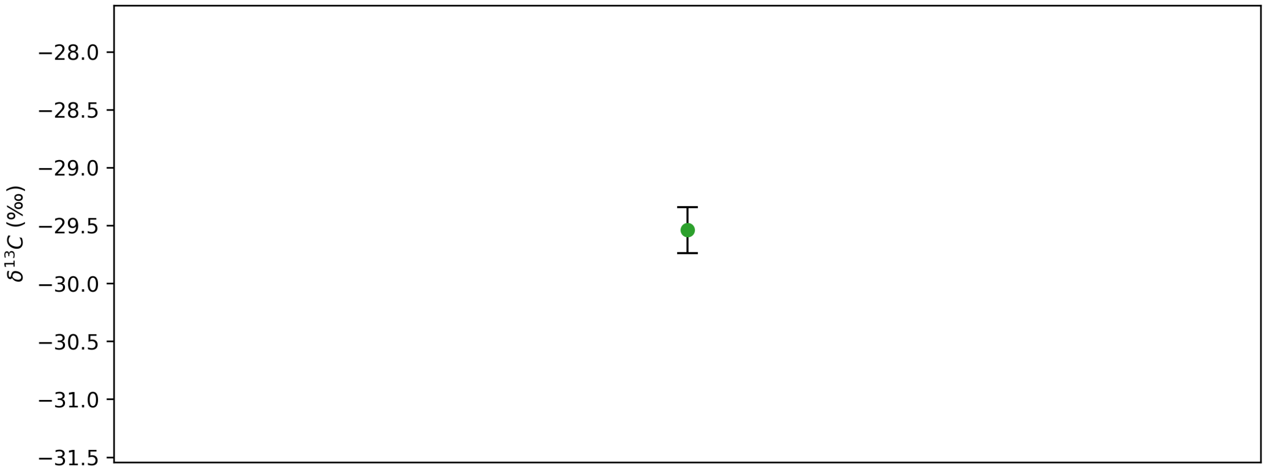

The final corrected average delta was -29.54 with a standard deviation of 0.20. Here the standard deviation is called reproducibility error.
