# Supplementary material for: Advancing Stable Isotope Analysis with Orbitrap-MS for Fatty Acid Methyl Esters and Complex Lipid Matrices
Source: J Am Soc Mass Spectrom. 2025 Jun 17;36(7):1527–35. doi: 10.1021/jasms.5c00092 (PMC12339014; doi:10.1021/jasms.5c00092)
Supplement: Supplementary file 2 [file js5c00092_si_002.zip › reports by IsotoPy Software/butters/Shea_rep2.pdf]

# Shea butter (replicate 2)
# Isotope Analysis report from IsotoPy

Flow Injection

# 1. Pre Processing

## 1.1. Block Time and Scan Information

Information about sample and standard block times and scans:

| Block | Injected | Initial Time | End Time | Number of scans |
|---|---|---|---|---|
| 1 | standard | 1 | 8 | 1266 |
| 2 | sample | 16 | 23 | 1318 |
| 3 | standard | 31 | 38 | 1305 |
| 4 | sample | 46 | 53 | 1259 |
| 5 | standard | 61 | 68 | 1289 |
| 6 | sample | 76 | 83 | 1308 |
| 7 | standard | 91 | 98 | 1289 |

## 1.2. Outlier Removal

A total of 2005 scans were considered outliers and removed using the MAD method

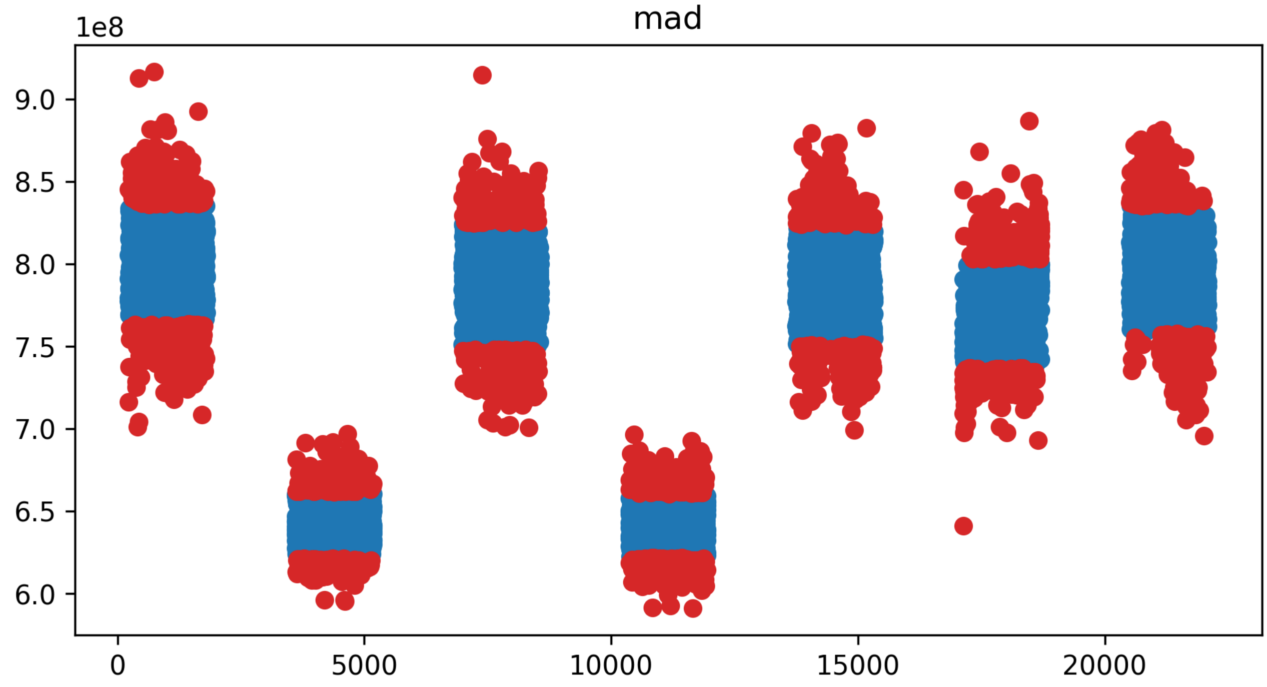

## 1.3. Total Ion Current (TIC)

TIC of all blocks

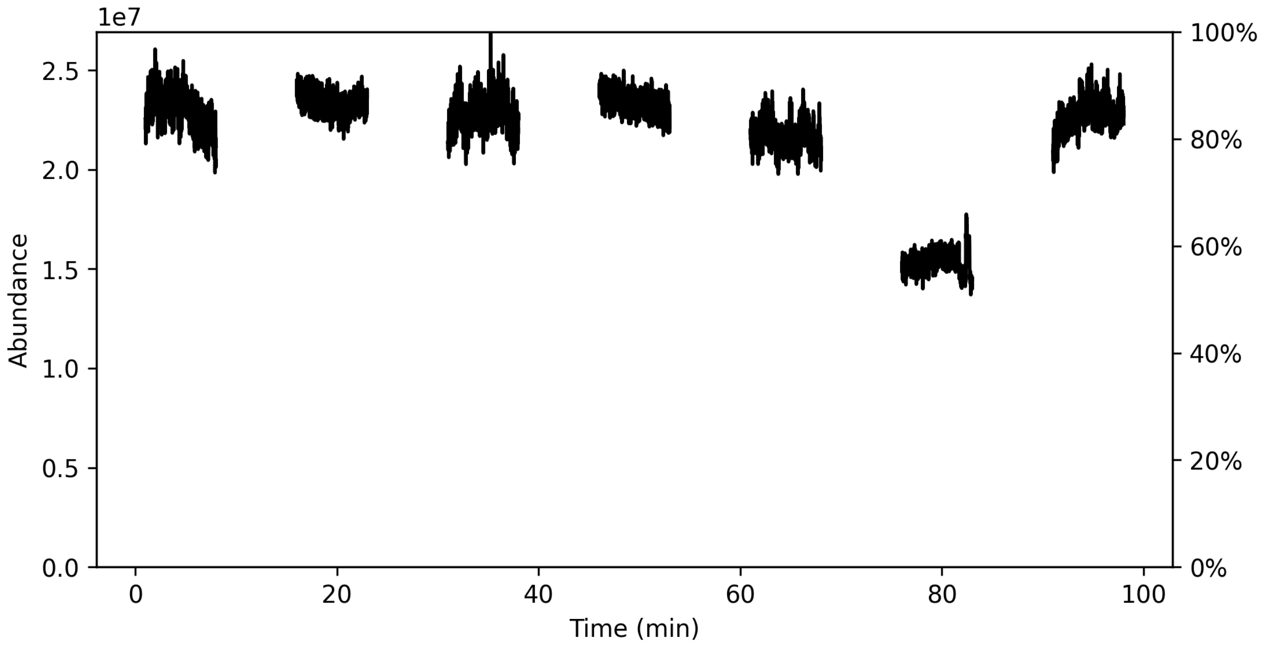


| Block | TIC min | TIC max | TIC mean | RSD (%) |
|---:|---:|---:|---:|---:|
| 1 | 1.98e+07 | 2.61e+07 | 2.30e+07 | 4.23 |
| 2 | 2.15e+07 | 2.48e+07 | 2.34e+07 | 2.18 |
| 3 | 2.03e+07 | 2.69e+07 | 2.28e+07 | 3.71 |
| 4 | 2.17e+07 | 2.50e+07 | 2.34e+07 | 2.20 |
| 5 | 1.98e+07 | 2.40e+07 | 2.16e+07 | 3.10 |
| 6 | 1.37e+07 | 1.78e+07 | 1.54e+07 | 3.41 |
| 7 | 1.99e+07 | 2.53e+07 | 2.27e+07 | 3.37 |

# 2. Block Parameters

The Isotopic Ratio of the blocks were calculated by 'Mean'

## 2.1. 13C/M0

| Block | Number of scans | Effective number of ions | Isotopic Ratio | STD | SEM | RSE |
|---|---|---|---|---|---|---|
| 1 | 1266 | 1.62e+07 | 0.196623 | 0.001727 | 0.000049 | 0.000247 |
| 2 | 1318 | 1.72e+07 | 0.197415 | 0.001709 | 0.000047 | 0.000238 |
| 3 | 1305 | 1.68e+07 | 0.196690 | 0.001780 | 0.000049 | 0.000250 |
| 4 | 1259 | 1.63e+07 | 0.197456 | 0.001696 | 0.000048 | 0.000242 |
| 5 | 1289 | 1.65e+07 | 0.196588 | 0.001792 | 0.000050 | 0.000254 |
| 6 | 1308 | 1.58e+07 | 0.197097 | 0.001765 | 0.000049 | 0.000247 |
| 7 | 1289 | 1.64e+07 | 0.196701 | 0.001781 | 0.000050 | 0.000252 |

**Errors and Test Paramters**

| Block | Acquisition Error (permil) | Shot-Noise (permil) | AE/SN ratio | Shapiro Wilk (p_value) | D'Agostino (p_value) |
|---|---|---|---|---|---|
| 1 | 0.247 | 0.249 | 0.993 | 0.123 | 0.108 |
| 2 | 0.238 | 0.241 | 0.990 | 0.387 | 0.311 |
| 3 | 0.250 | 0.244 | 1.025 | 0.048 | 0.374 |
| 4 | 0.242 | 0.248 | 0.977 | 0.892 | 0.869 |
| 5 | 0.254 | 0.246 | 1.032 | 0.553 | 0.648 |
| 6 | 0.247 | 0.252 | 0.983 | 0.416 | 0.300 |
| 7 | 0.252 | 0.247 | 1.022 | 0.007 | 0.035 |

# Isotopic Ratio and Errors of the Blocks

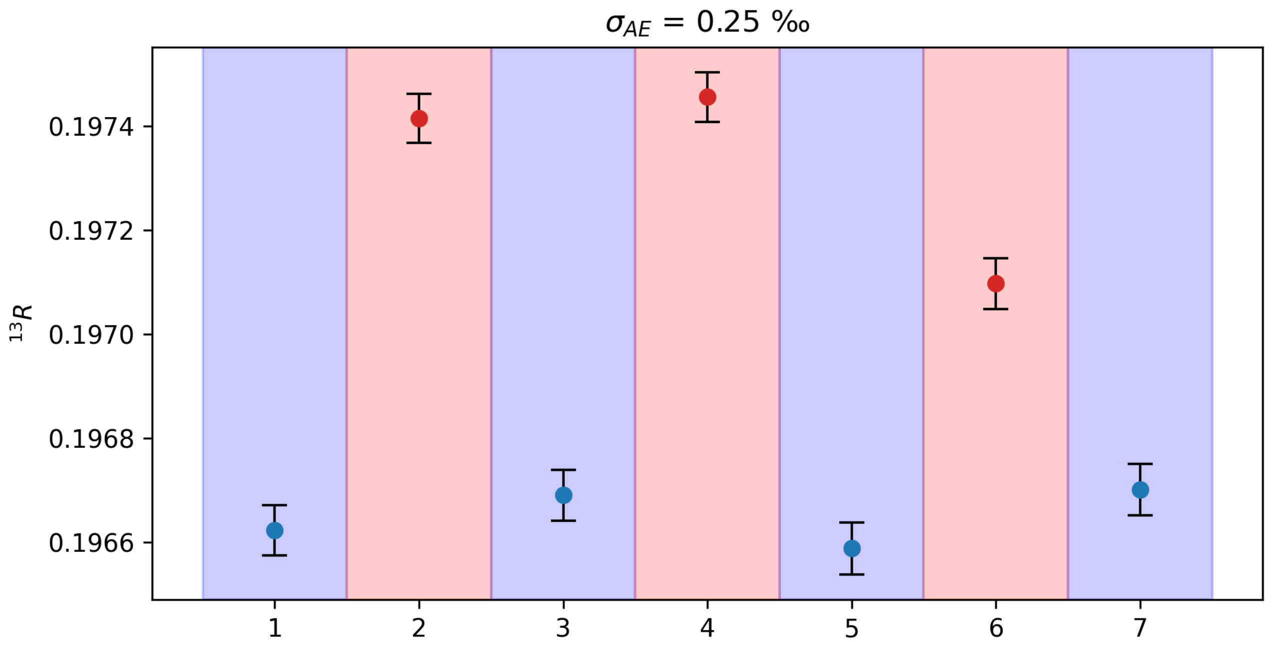


# Cumulative Isotopic Ratio

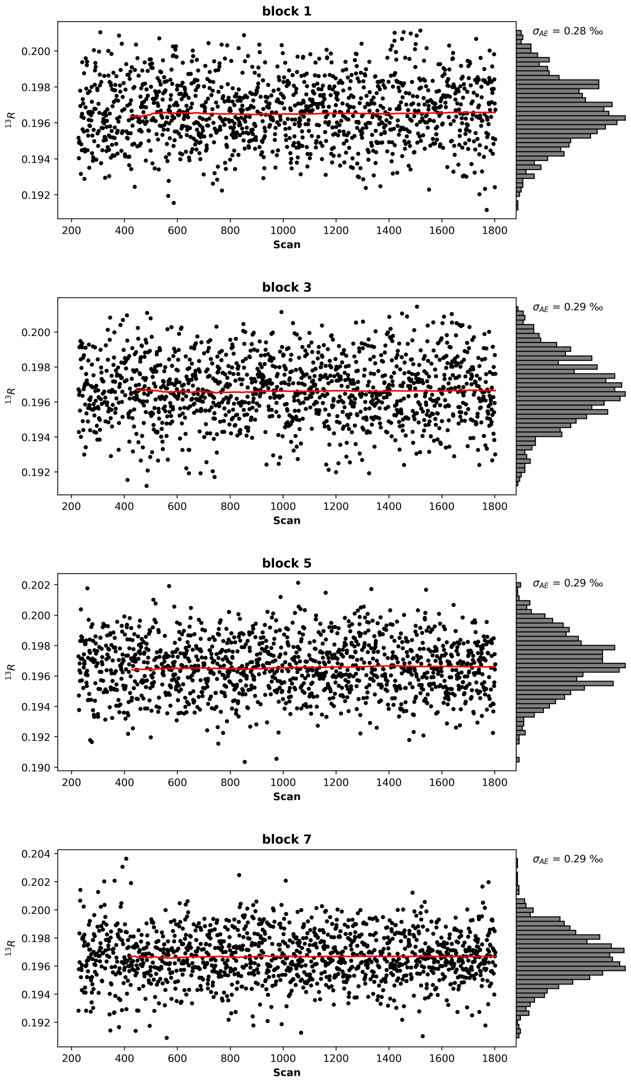

# Acquisition Error and Shot-Noise

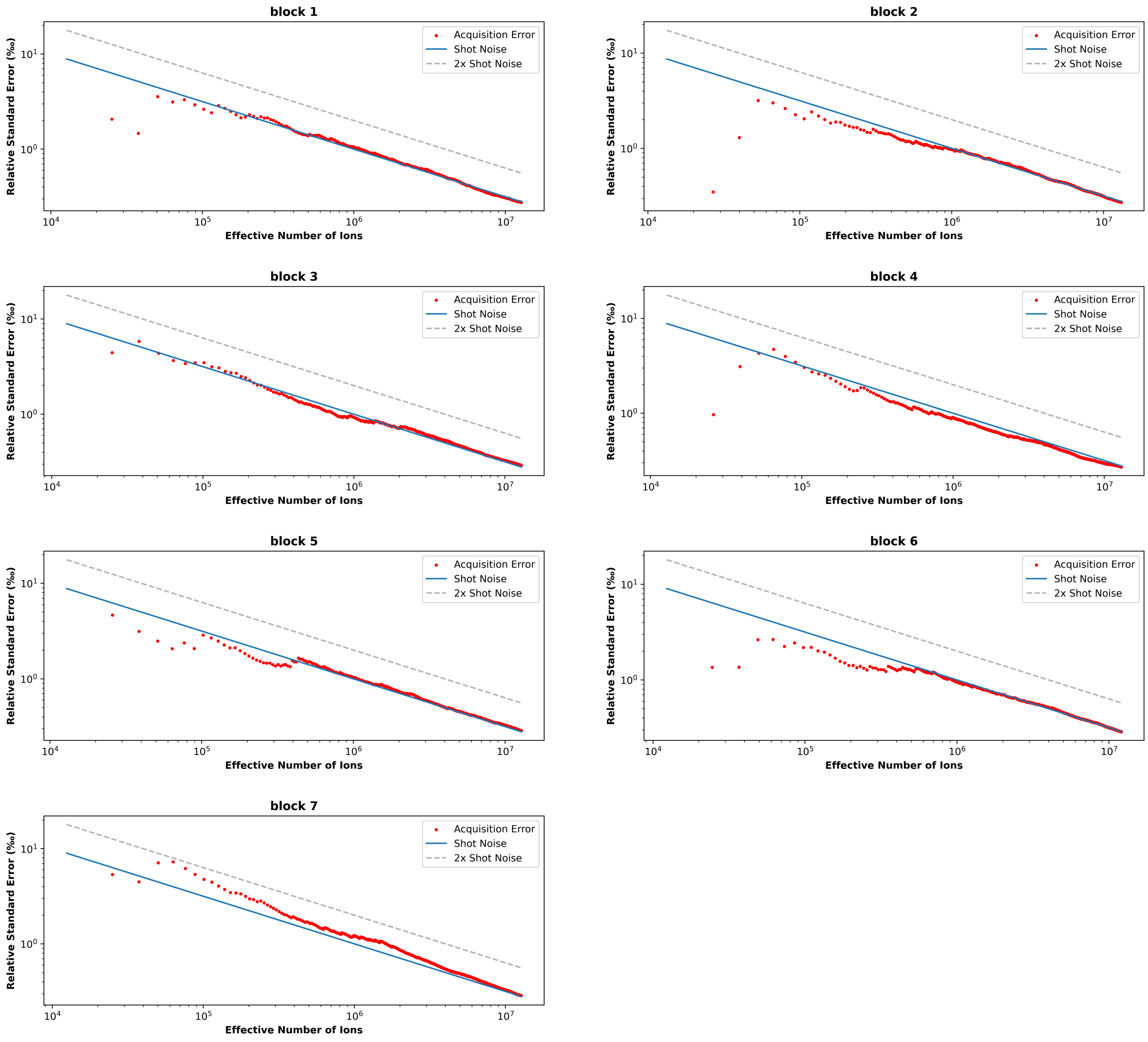

# 3. Delta Informations

Deltas were calculated by 'Average Of Neighboring Block Ratios'

## 3.1. 13C

Delta 13C was corrected by -27.80

| Block | SEM | Delta corrected | Delta |
|---|---|---|---|
| 2 | 0.24 | -24.05 | 3.86 |
| 4 | 0.24 | -23.76 | 4.15 |
| 6 | 0.25 | -25.56 | 2.30 |

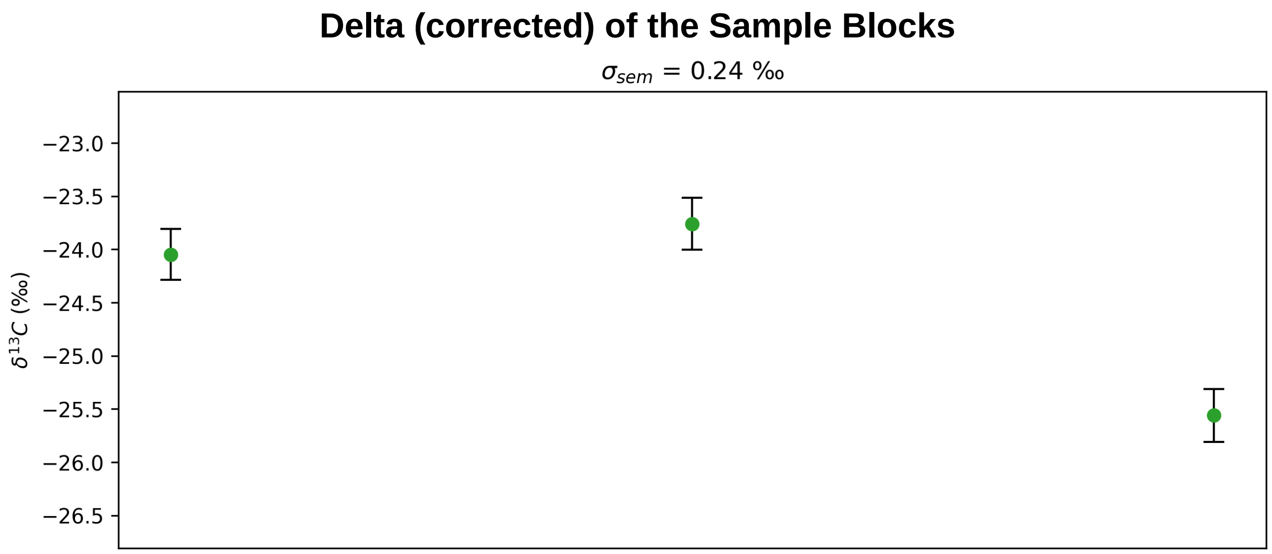


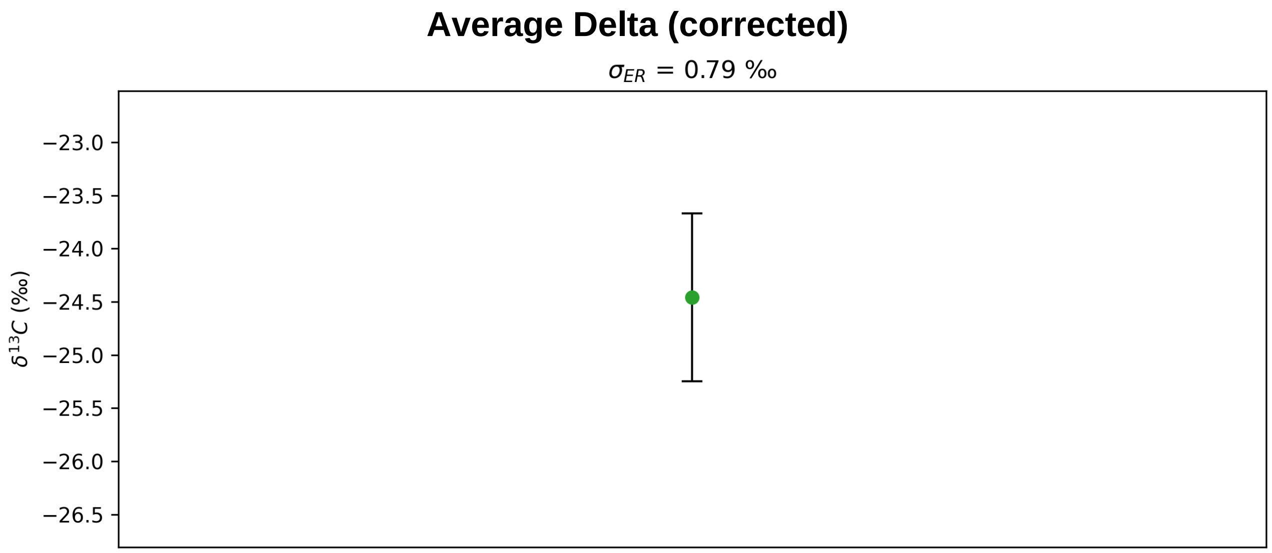


The final corrected average delta was -24.46 with a standard deviation of 0.79. Here the standard deviation is called reproducibility error.
