# Supplementary material for: Advancing Stable Isotope Analysis with Orbitrap-MS for Fatty Acid Methyl Esters and Complex Lipid Matrices
Source: J Am Soc Mass Spectrom. 2025 Jun 17;36(7):1527–35. doi: 10.1021/jasms.5c00092 (PMC12339014; doi:10.1021/jasms.5c00092)
Supplement: Supplementary file 2 [file js5c00092_si_002.zip › reports by IsotoPy Software/butters/Shea_rep3.pdf]

Shea butter (replicate 3)
Isotope Analysis report from IsotoPy
Flow Injection

1. Pre Processing

1.1. Block Time and Scan Information

Information about sample and standard block times and scans:

Block	Injected	Initial Time	End Time	Number of scans
1	standard	1	6	1102
2	sample	16	23	1324
3	standard	31	37	1134
4	sample	46	53	1314
5	standard	61	68	1273
6	sample	76	83	1287
7	standard	91	98	1273

1.2. Outlier Removal

A total of 2332 scans were considered outliers and removed using the MAD method

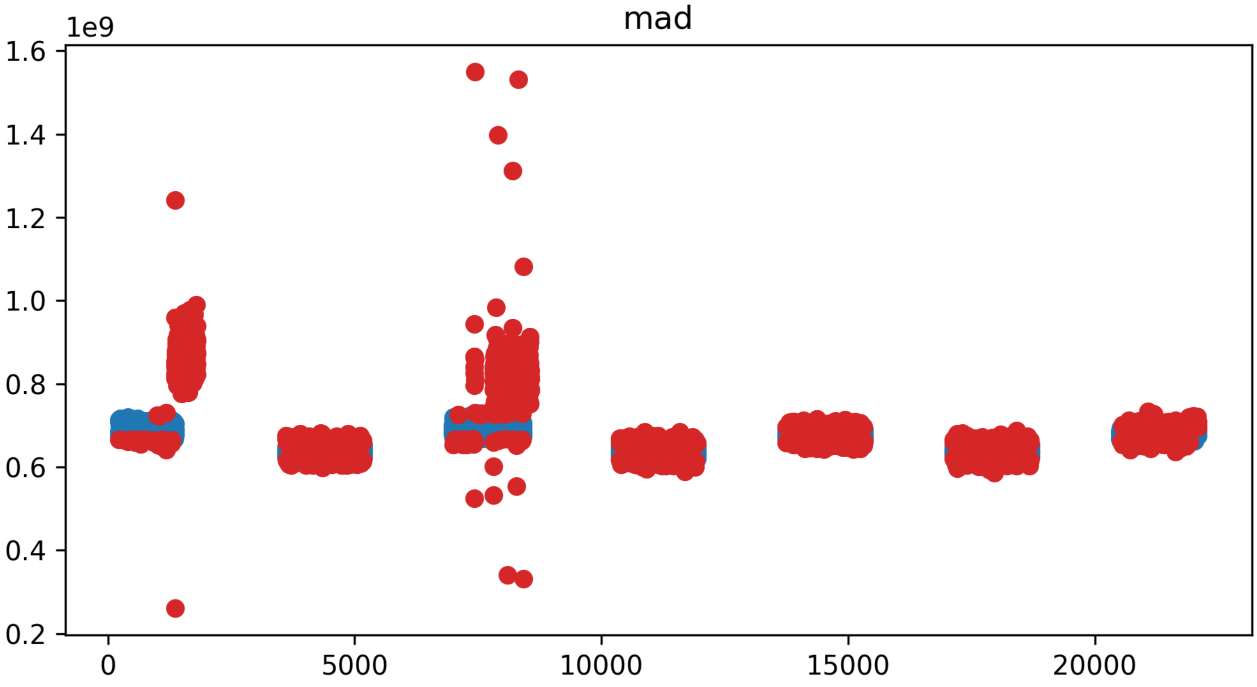

1.3. Total Ion Current (TIC)

TIC of all blocks

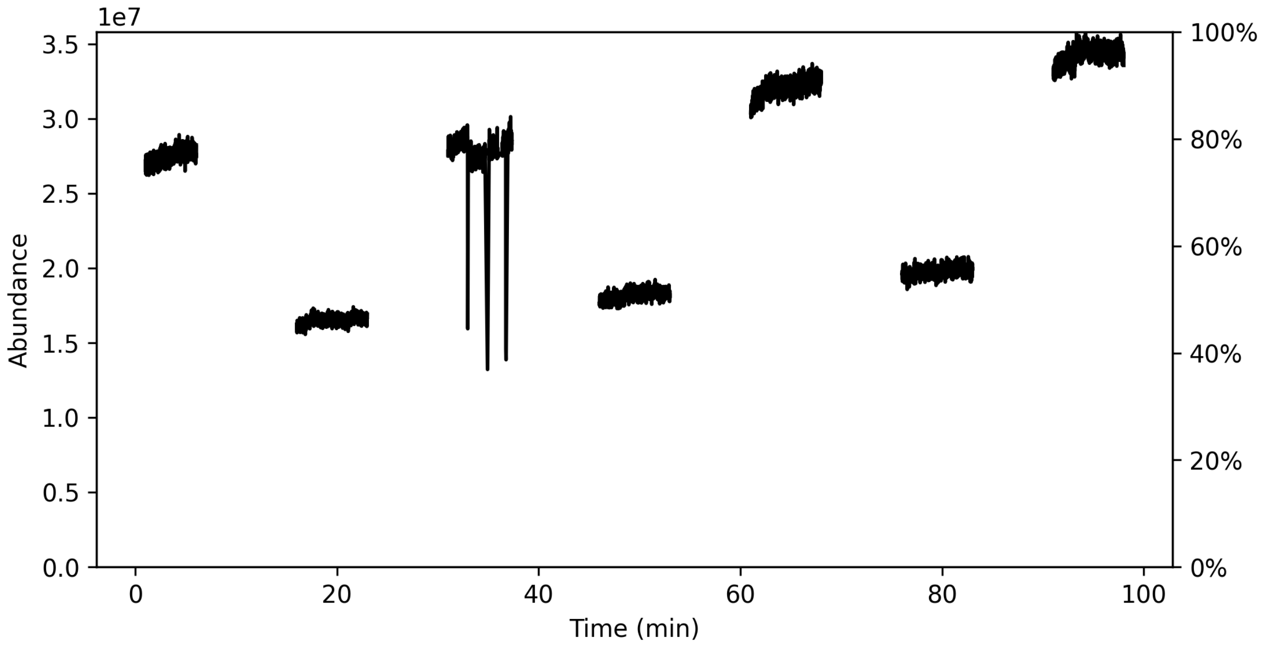

Block	TIC min	TIC max	TIC mean	RSD (%)
1	2.62e+07	2.89e+07	2.75e+07	1.71
2	1.56e+07	1.74e+07	1.65e+07	1.66
3	1.32e+07	3.01e+07	2.79e+07	3.54
4	1.73e+07	1.92e+07	1.82e+07	1.86
5	3.01e+07	3.37e+07	3.20e+07	1.80
6	1.86e+07	2.08e+07	1.98e+07	1.67
7	3.26e+07	3.58e+07	3.43e+07	1.69

2. Block Parameters

The Isotopic Ratio of the blocks were calculated by 'Mean'

2.1. 13C/M0

Block	Number of scans	Effective number of ions	Isotopic Ratio	STD	SEM	RSE
1	1102	1.59e+07	0.196575	0.001656	0.000050	0.000254
2	1324	1.82e+07	0.196936	0.001697	0.000047	0.000237
3	1134	1.60e+07	0.196566	0.001639	0.000049	0.000247
4	1314	1.76e+07	0.196780	0.001727	0.000048	0.000242
5	1273	1.75e+07	0.196976	0.001694	0.000047	0.000241
6	1287	1.68e+07	0.197078	0.001719	0.000048	0.000243
7	1273	1.72e+07	0.196883	0.001606	0.000045	0.000229

Errors and Test Paramters

Block	Acquisition Error (permil)	Shot-Noise (permil)	AE/SN ratio	Shapiro Wilk (p_value)	D'Agostino (p_value)
1	0.254	0.251	1.010	0.196	0.468
2	0.237	0.234	1.010	0.241	0.329
3	0.247	0.250	0.988	0.700	0.545
4	0.242	0.238	1.016	0.061	0.068
5	0.241	0.239	1.009	0.193	0.451
6	0.243	0.244	0.998	0.749	0.465
7	0.229	0.241	0.948	0.731	0.639

Isotopic Ratio and Errors of the Blocks

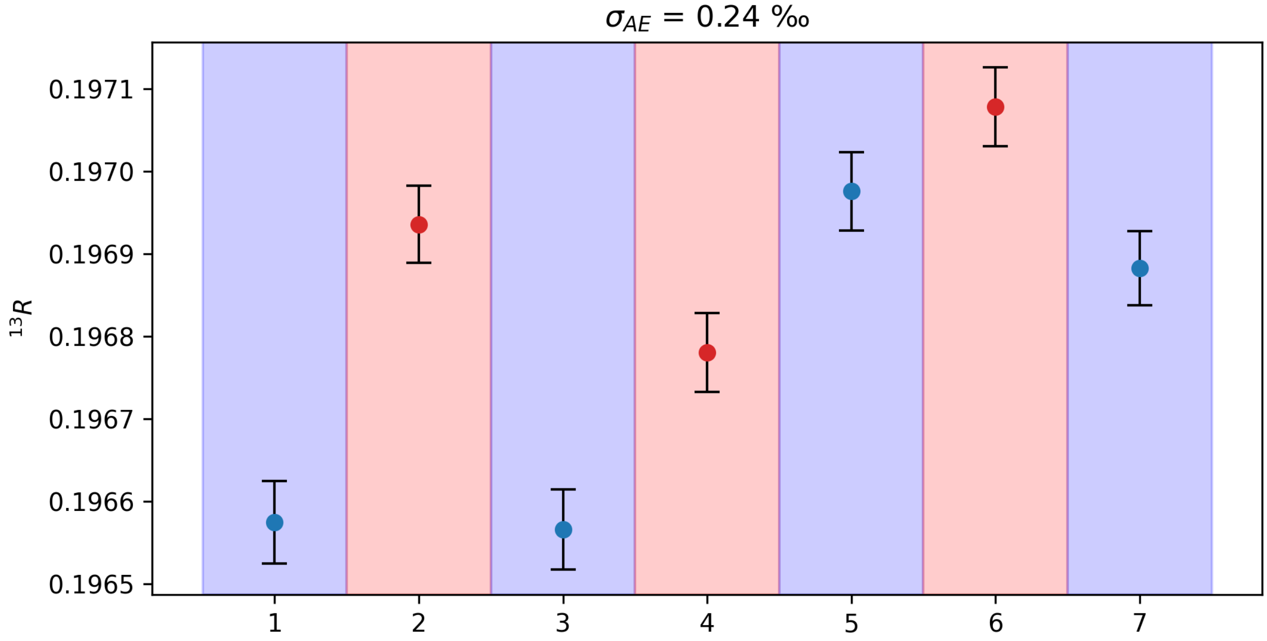

Cumulative Isotopic Ratio

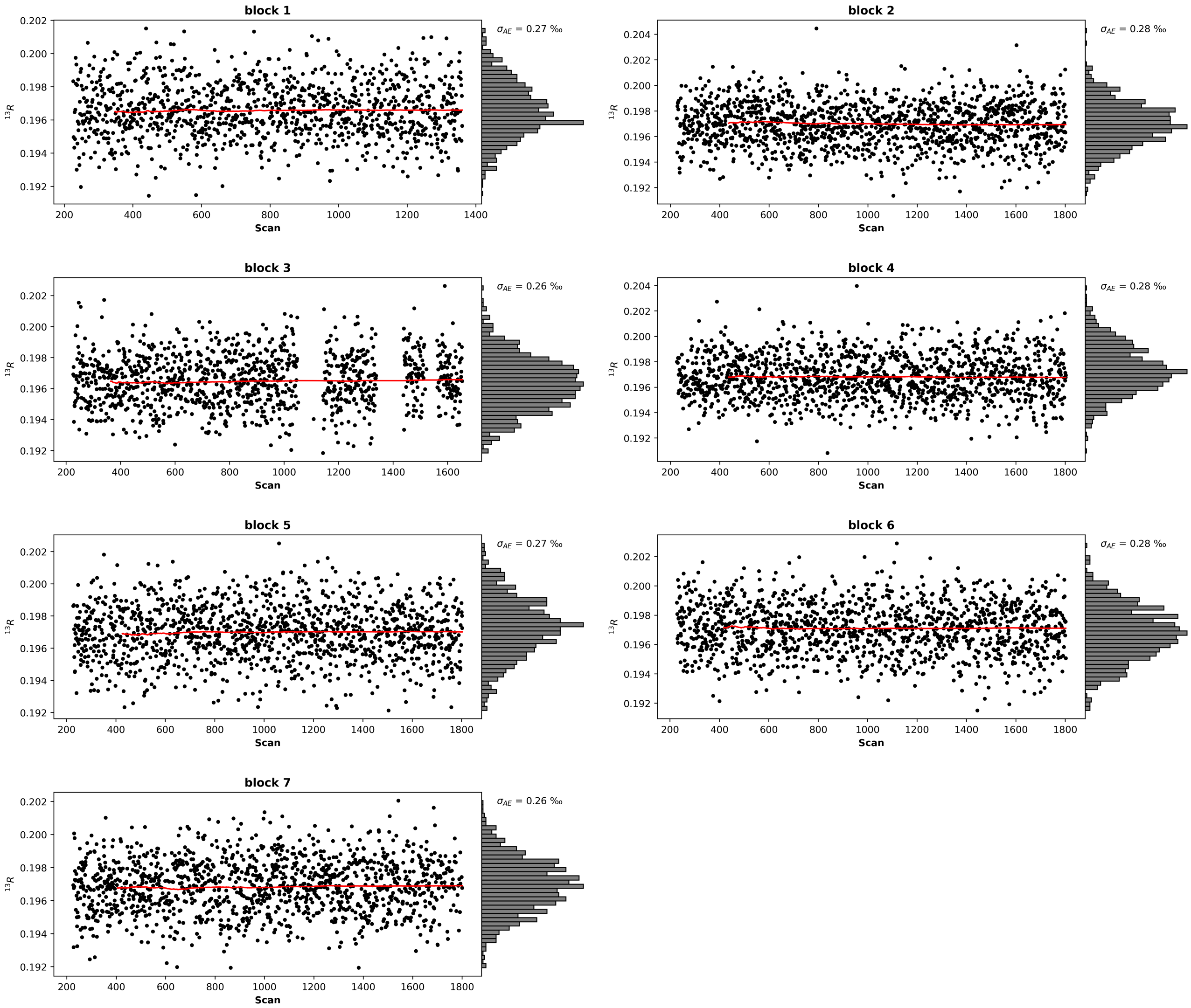

Acquisition Error and Shot-Noise

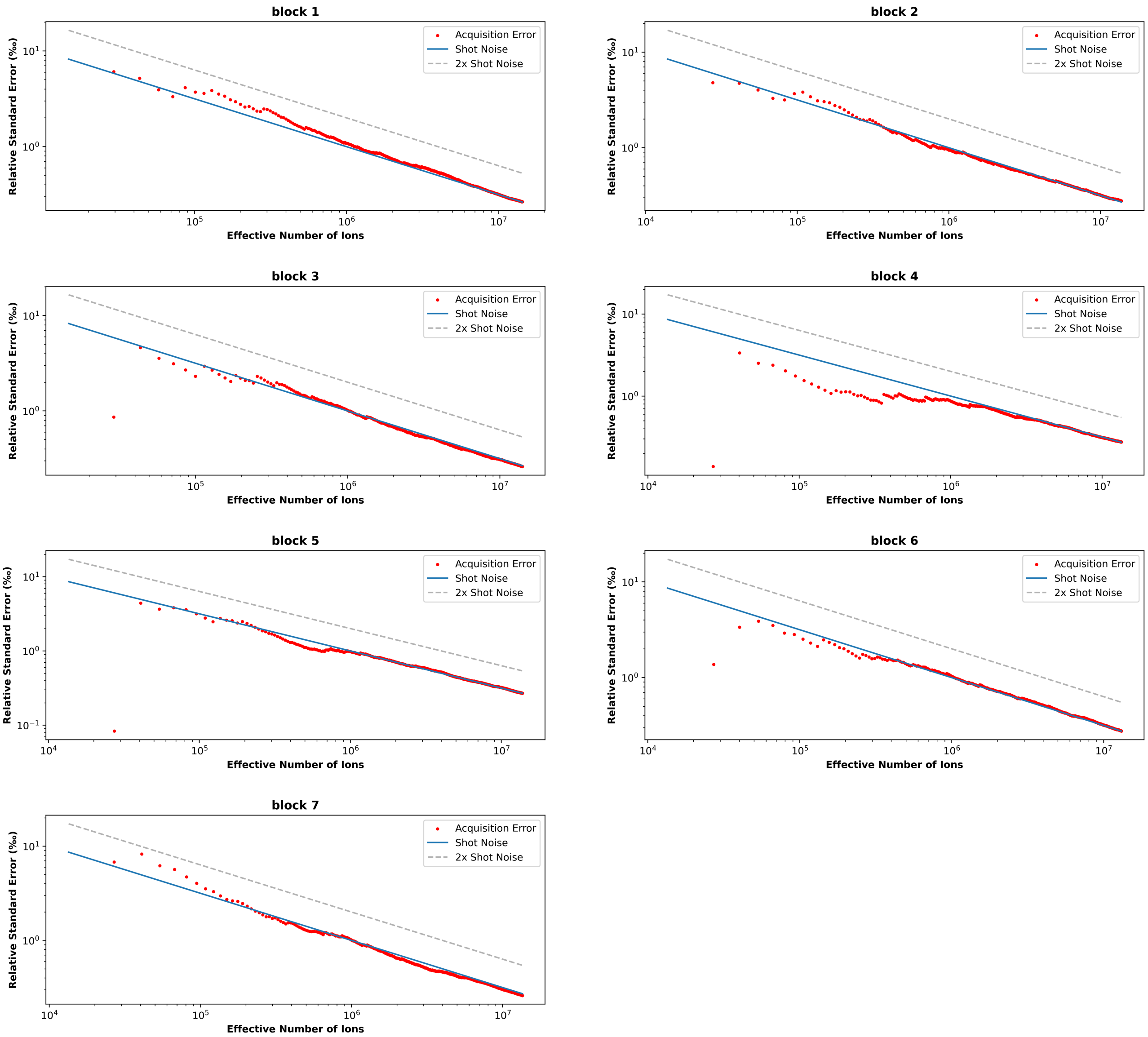

3. Delta Informations

Deltas were calculated by 'Average Of Neighboring Block Ratios'

3.1. 13C

Delta 13C was corrected by -27.80

Block	SEM	Delta corrected	Delta
2	0.24	-25.99	1.86
4	0.24	-27.75	0.05
6	0.24	-27.06	0.76

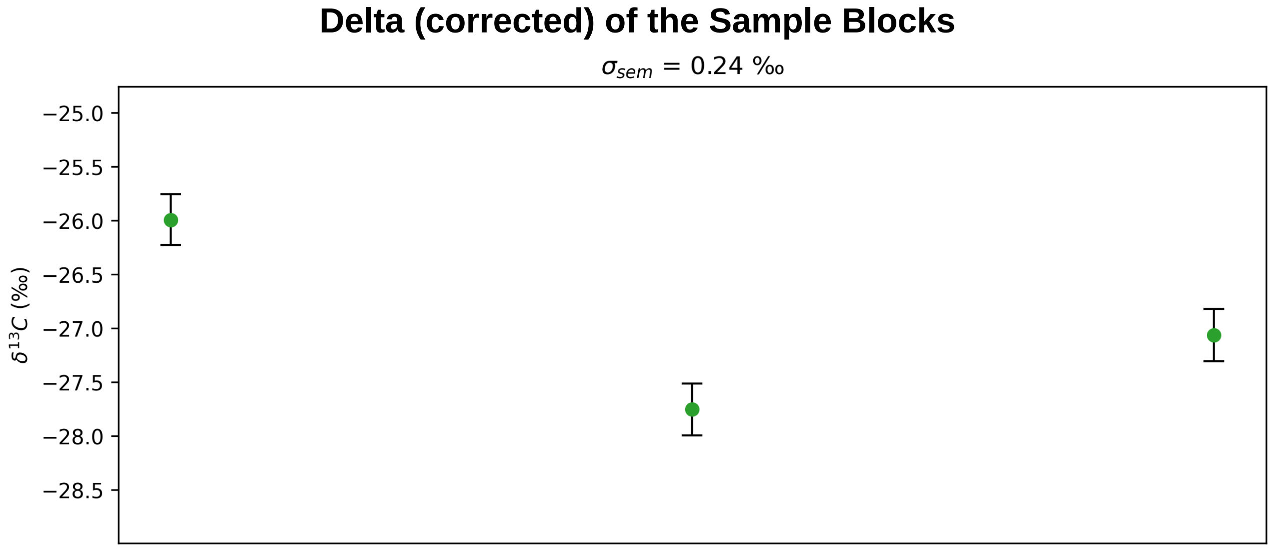

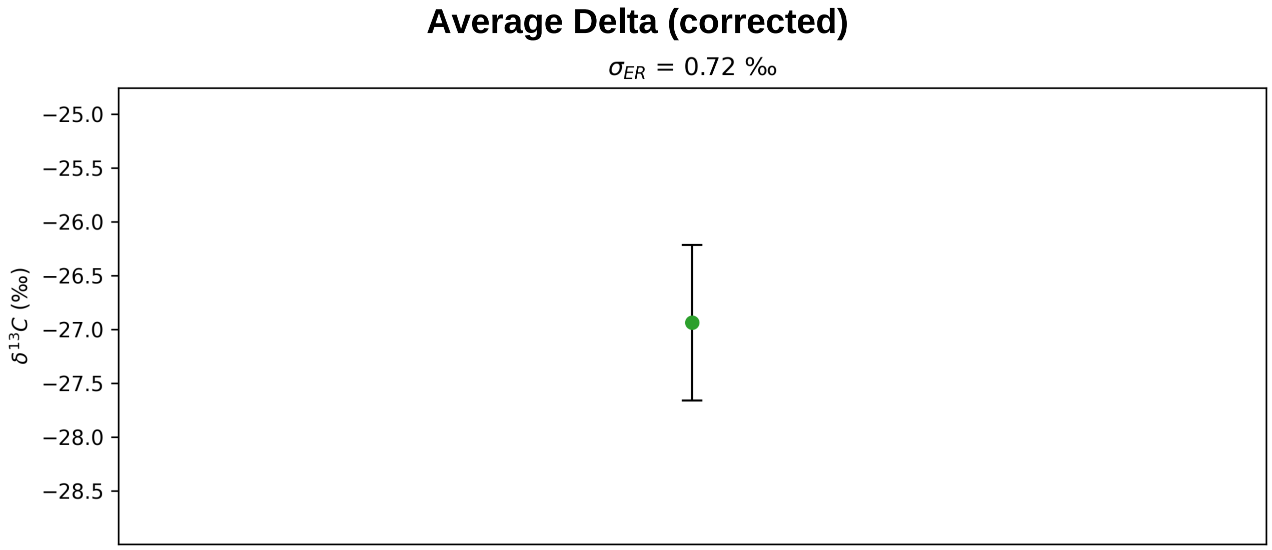

The final corrected average delta was -26.94 with a standard deviation of 0.72. Here the standard deviation is called reproducibility error.
