# Supplementary material for: Advancing Stable Isotope Analysis with Orbitrap-MS for Fatty Acid Methyl Esters and Complex Lipid Matrices
Source: J Am Soc Mass Spectrom. 2025 Jun 17;36(7):1527–35. doi: 10.1021/jasms.5c00092 (PMC12339014; doi:10.1021/jasms.5c00092)
Supplement: Supplementary file 2 [file js5c00092_si_002.zip › reports by IsotoPy Software/standards/Na+Standard4_FI.pdf]

Standard 4 - [M + Na]+
Isotope Analysis report from IsotoPy

Flow Injection

1. Pre Processing

1.1. Block Time and Scan Information

Information about sample and standard block times and scans:

Block	Injected	Initial Time	End Time	Number of scans
1	standard	1	8	1277
2	sample	16	23	1261
3	standard	31	38	1321
4	sample	46	53	1297
5	standard	61	68	1307
6	sample	76	83	1303
7	standard	91	98	1325

1.2. Outlier Removal

A total of 1948 scans were considered outliers and removed using the MAD method

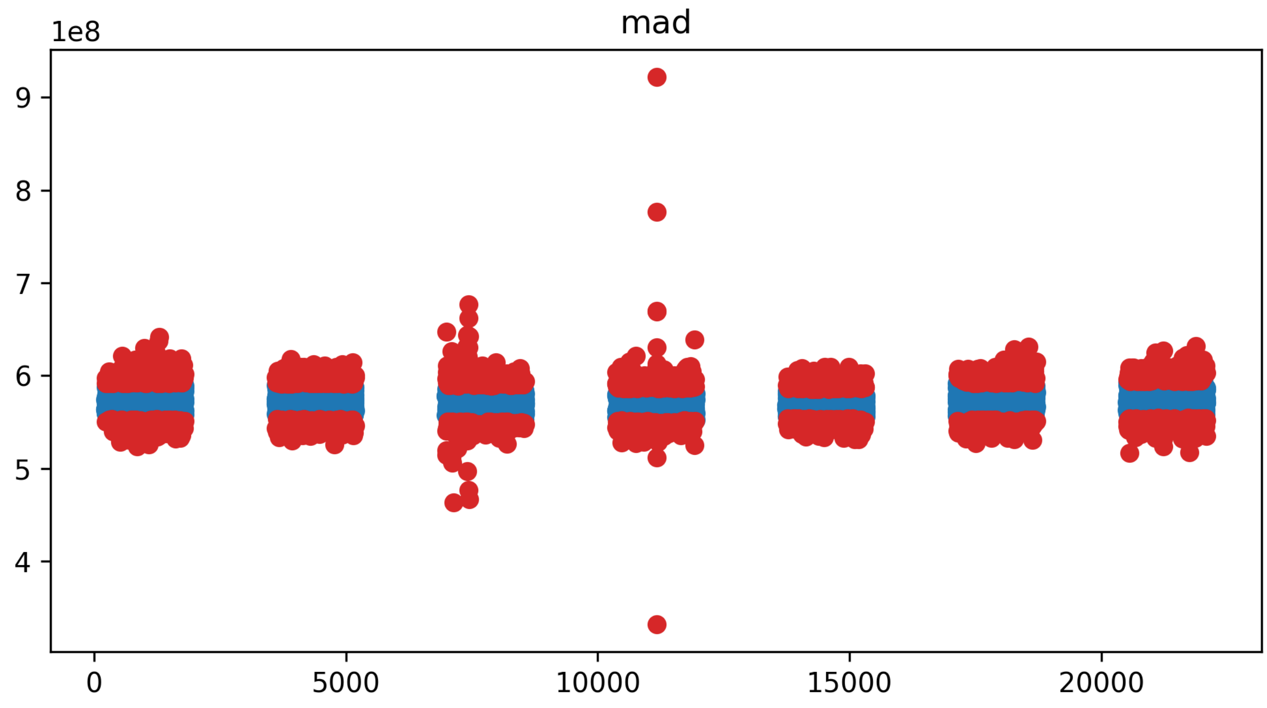

1.3. Total Ion Current (TIC)

TIC of all blocks

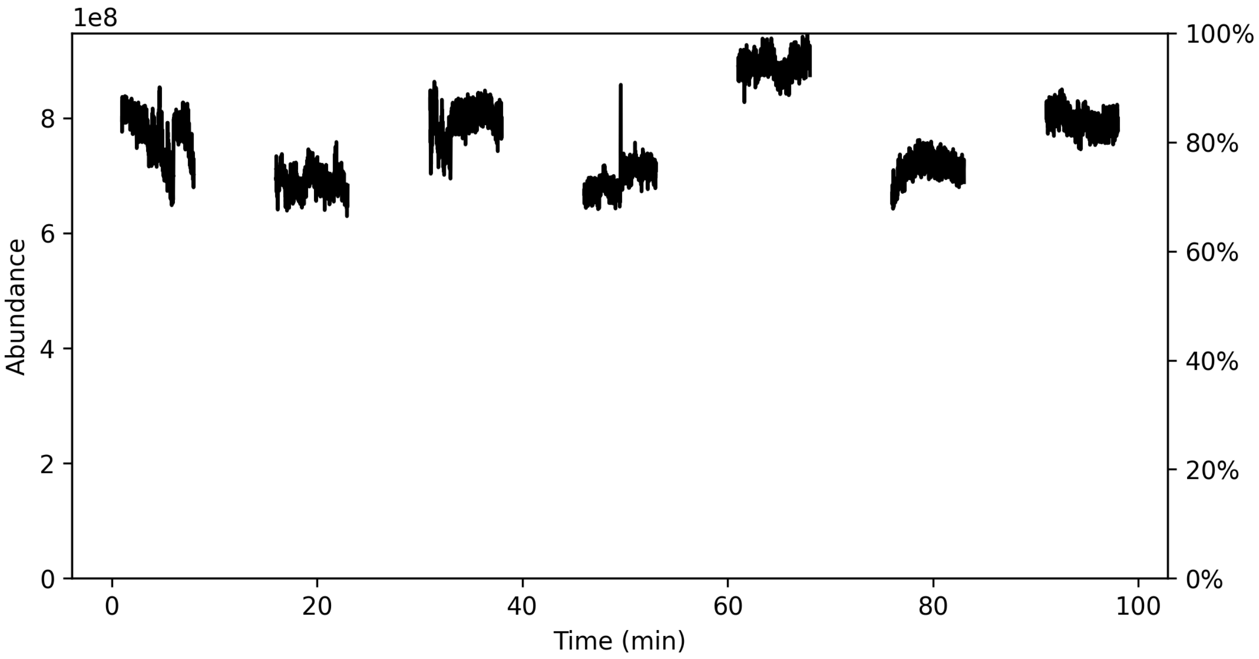

Block	TIC min	TIC max	TIC mean	RSD (%)
1	6.48e+08	8.53e+08	7.72e+08	5.09
2	6.29e+08	7.58e+08	6.89e+08	2.83
3	6.95e+08	8.63e+08	7.95e+08	3.39
4	6.42e+08	8.58e+08	6.93e+08	3.15
5	8.27e+08	9.47e+08	8.92e+08	1.86
6	6.42e+08	7.62e+08	7.15e+08	2.68
7	7.45e+08	8.50e+08	7.95e+08	2.12

2. Block Parameters

The Isotopic Ratio of the blocks were calculated by 'Mean'

2.1. 13C/M0

Block	Number of scans	Effective number of ions	Isotopic Ratio	STD	SEM	RSE
1	1277	2.21e+07	0.209460	0.001750	0.000049	0.000234
2	1261	2.17e+07	0.209584	0.001783	0.000050	0.000240
3	1321	2.25e+07	0.209735	0.001727	0.000047	0.000226
4	1297	2.18e+07	0.209611	0.001866	0.000052	0.000247
5	1307	2.17e+07	0.208816	0.001759	0.000049	0.000233
6	1303	2.15e+07	0.208991	0.001775	0.000049	0.000235
7	1325	2.17e+07	0.209249	0.001772	0.000049	0.000233

Errors and Test Paramters

Block	Acquisition Error (permil)	Shot-Noise (permil)	AE/SN ratio	Shapiro Wilk (p_value)	D'Agostino (p_value)
1	0.234	0.213	1.098	0.749	0.748
2	0.240	0.215	1.115	0.199	0.328
3	0.226	0.211	1.075	0.861	0.873
4	0.247	0.214	1.154	0.339	0.559
5	0.233	0.215	1.085	0.468	0.284
6	0.235	0.216	1.090	0.899	0.683
7	0.233	0.215	1.083	0.063	0.011

Isotopic Ratio and Errors of the Blocks

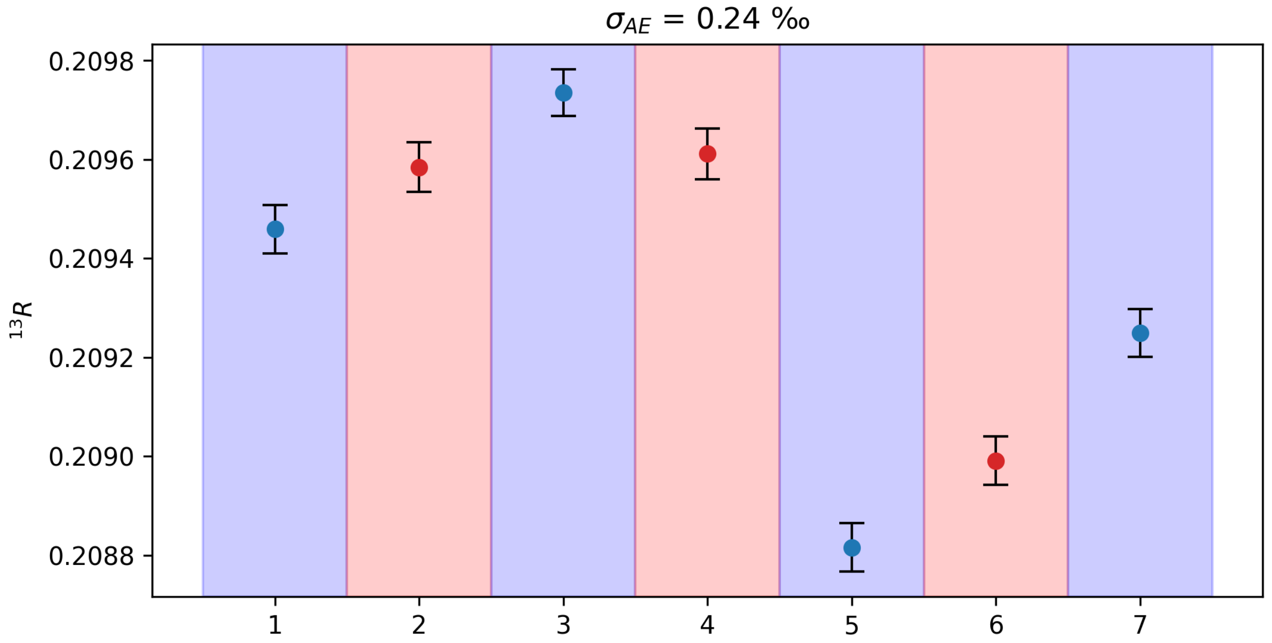

Cumulative Isotopic Ratio

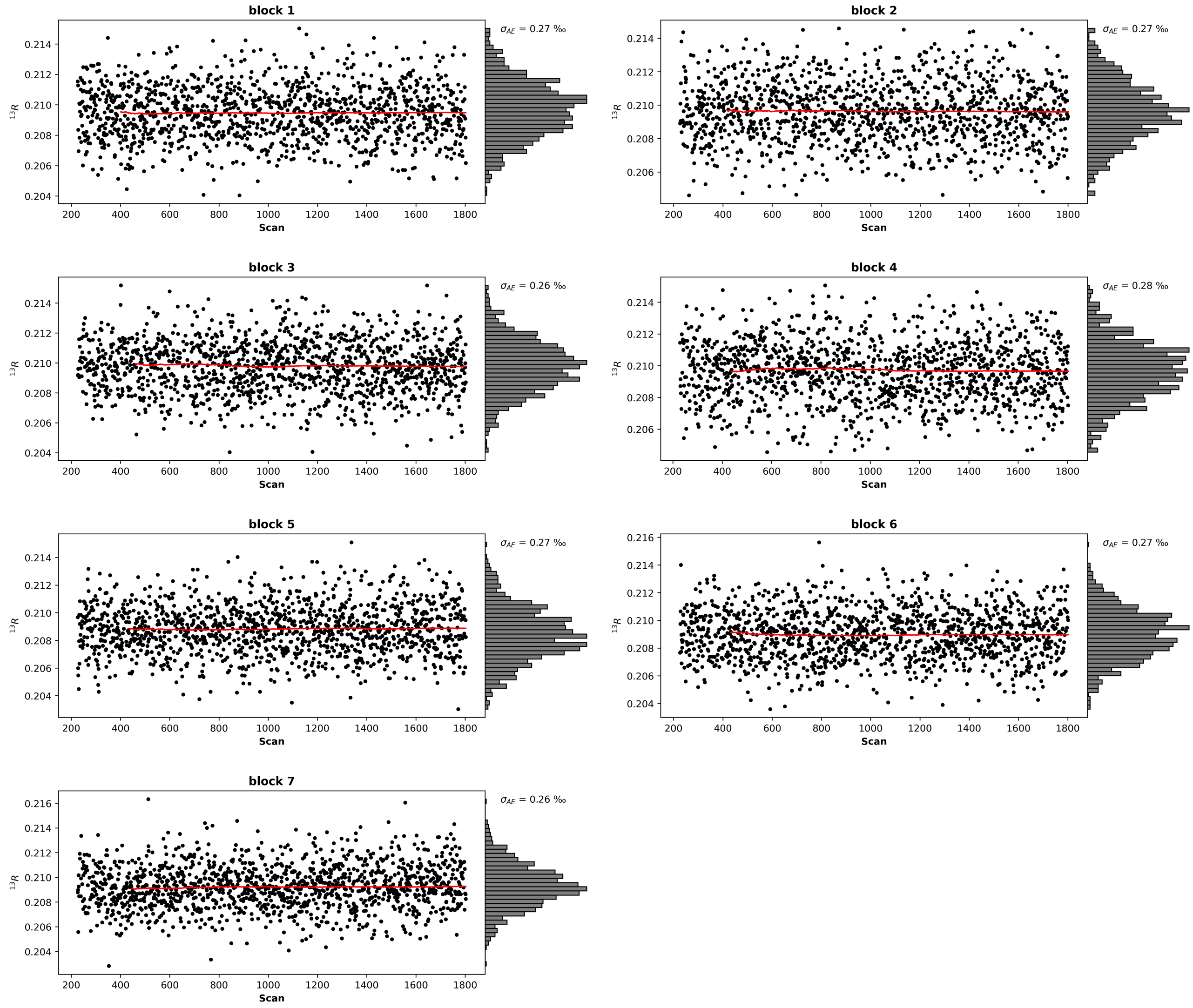

Acquisition Error and Shot-Noise

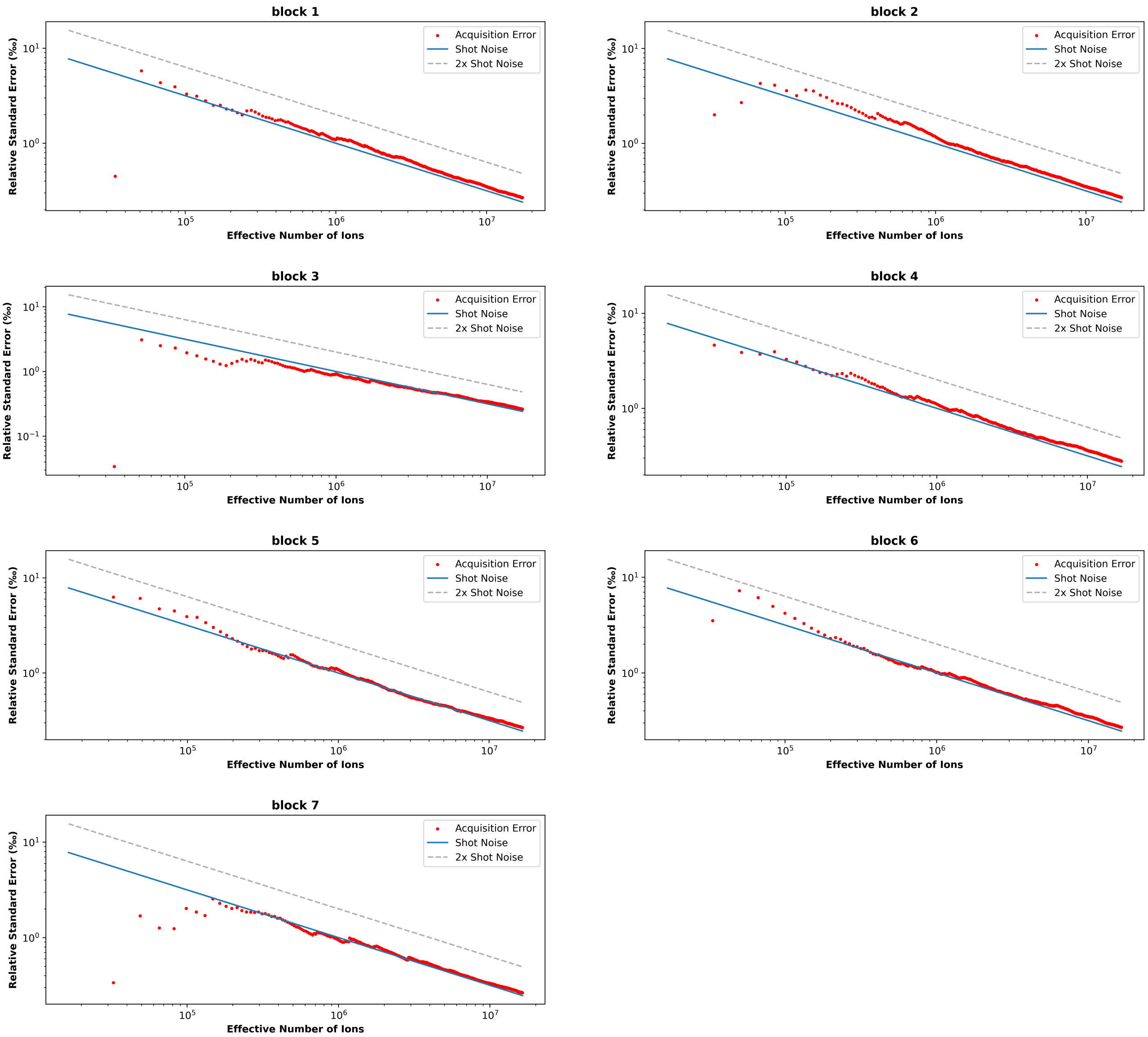

3. Delta Informations

Deltas were calculated by 'Average Of Neighboring Block Ratios'

3.1. 13C

Delta 13C was corrected by -27.80

Block	SEM	Delta corrected	Delta
2	0.24	-27.86	-0.06
4	0.25	-26.24	1.61
6	0.24	-27.99	-0.20

Delta (corrected) of the Sample Blocks

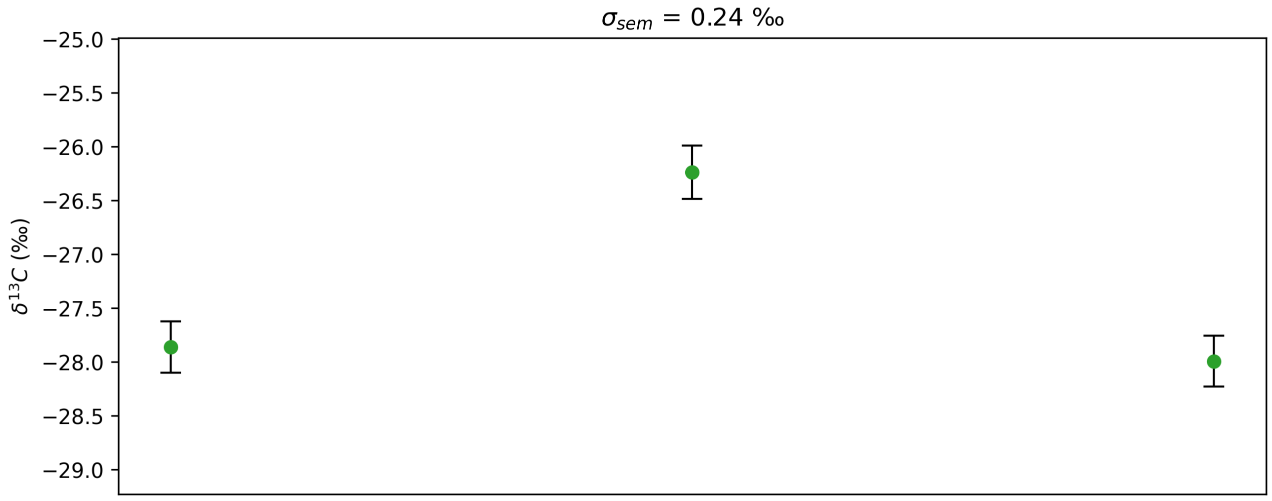

Average Delta (corrected)

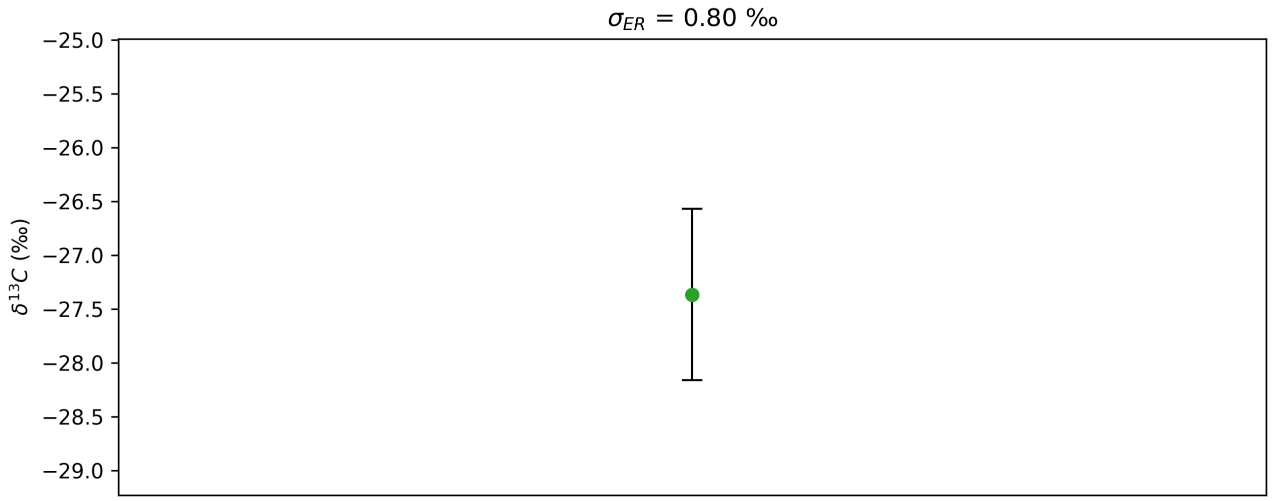

The final corrected average delta was -27.36 with a standard deviation of 0.80. Here the standard deviation is called reproducibility error.
