# Supplementary material for: Advancing Stable Isotope Analysis with Orbitrap-MS for Fatty Acid Methyl Esters and Complex Lipid Matrices
Source: J Am Soc Mass Spectrom. 2025 Jun 17;36(7):1527–35. doi: 10.1021/jasms.5c00092 (PMC12339014; doi:10.1021/jasms.5c00092)
Supplement: Supplementary file 2 [file js5c00092_si_002.zip › reports by IsotoPy Software/standards/Na+Standard8_FI.pdf]

Standard 8 - [M + Na]+

Isotope Analysis report from IsotoPy

Flow Injection

1. Pre Processing

1.1. Block Time and Scan Information

Information about sample and standard block times and scans:

Block	Injected	Initial Time	End Time	Number of scans
1	standard	1	8	1258
2	sample	16	23	1299
3	standard	31	38	1288
4	sample	46	53	1289
5	standard	61	68	1305
6	sample	76	83	1274
7	standard	91	98	1277

1.2. Outlier Removal

A total of 2049 scans were considered outliers and removed using the MAD method

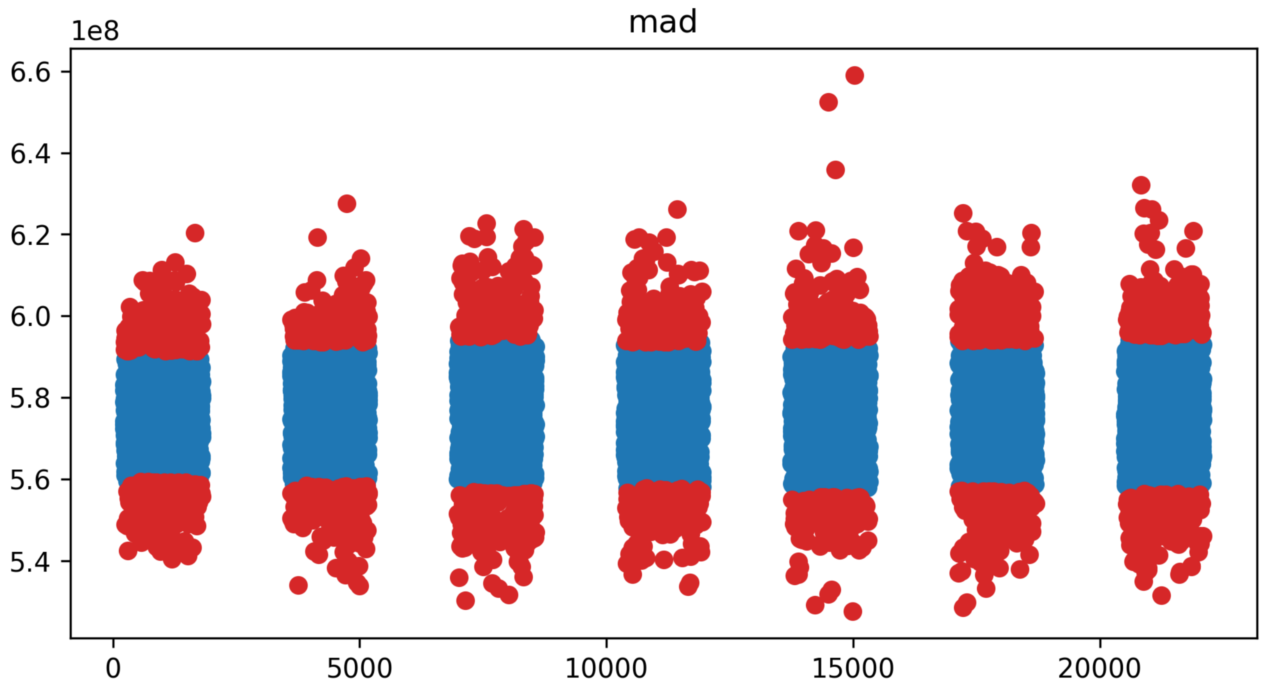

1.3. Total Ion Current (TIC)

TIC of all blocks

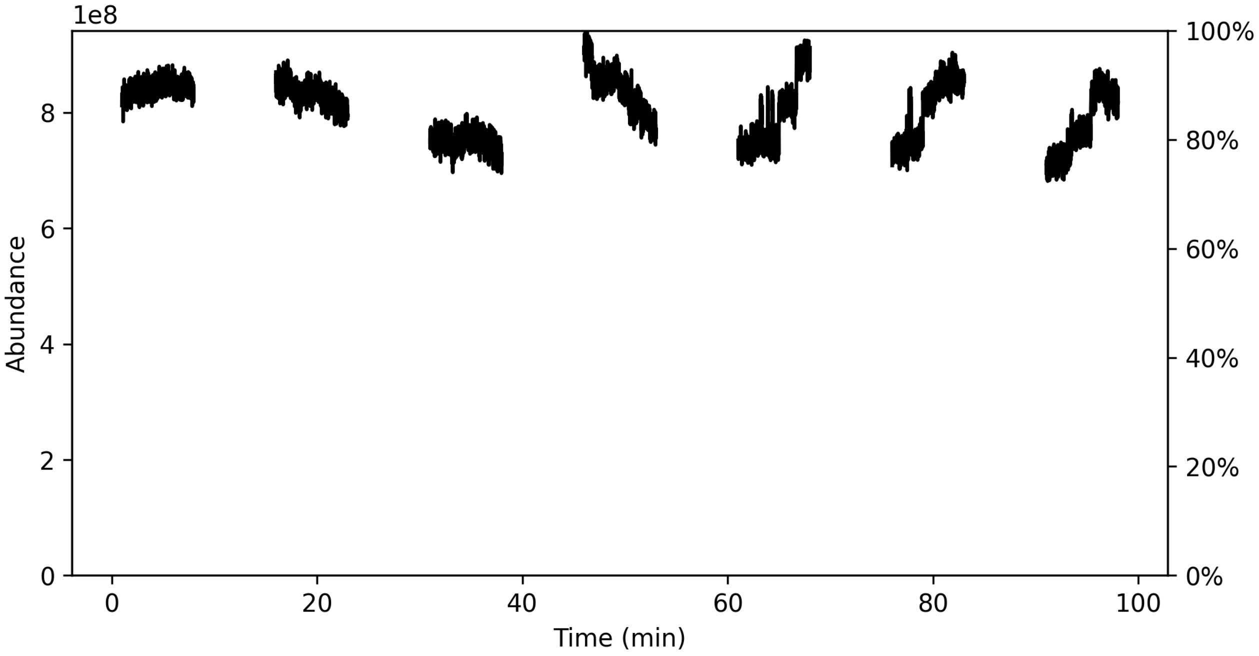

Block	TIC min	TIC max	TIC mean	RSD (%)
1	7.84e+08	8.82e+08	8.42e+08	1.61
2	7.76e+08	8.90e+08	8.31e+08	2.35
3	6.95e+08	7.98e+08	7.49e+08	2.24
4	7.44e+08	9.41e+08	8.38e+08	4.76
5	7.09e+08	9.25e+08	7.93e+08	7.49
6	7.00e+08	9.04e+08	8.06e+08	6.70
7	6.81e+08	8.76e+08	7.75e+08	6.74

2. Block Parameters

The Isotopic Ratio of the blocks were calculated by 'Mean'

2.1. 13C/M0

Block	Number of scans	Effective number of ions	Isotopic Ratio	STD	SEM	RSE
1	1258	2.02e+07	0.209257	0.001782	0.000050	0.000240
2	1299	2.09e+07	0.209295	0.001835	0.000051	0.000243
3	1288	2.07e+07	0.209541	0.001717	0.000048	0.000228
4	1289	2.07e+07	0.209272	0.001782	0.000050	0.000237
5	1305	2.10e+07	0.209522	0.001764	0.000049	0.000233
6	1274	2.05e+07	0.209407	0.001802	0.000050	0.000241
7	1277	2.05e+07	0.209376	0.001778	0.000050	0.000237

Errors and Test Paramters

Block	Acquisition Error (permil)	Shot-Noise (permil)	AE/SN ratio	Shapiro Wilk (p_value)	D'Agostino (p_value)
1	0.240	0.222	1.079	0.654	0.526
2	0.243	0.219	1.111	0.199	0.848
3	0.228	0.220	1.039	0.964	0.843
4	0.237	0.220	1.079	0.081	0.812
5	0.233	0.218	1.067	0.861	0.756
6	0.241	0.221	1.090	0.223	0.236
7	0.237	0.221	1.076	0.578	0.805

Isotopic Ratio and Errors of the Blocks

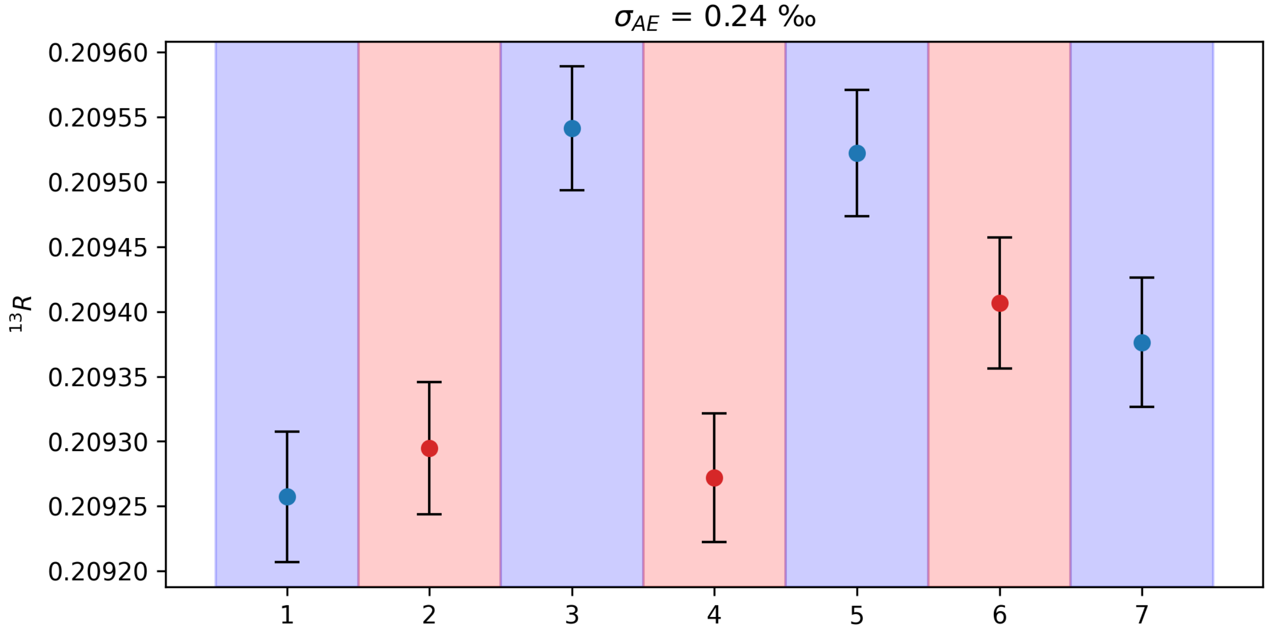

Cumulative Isotopic Ratio

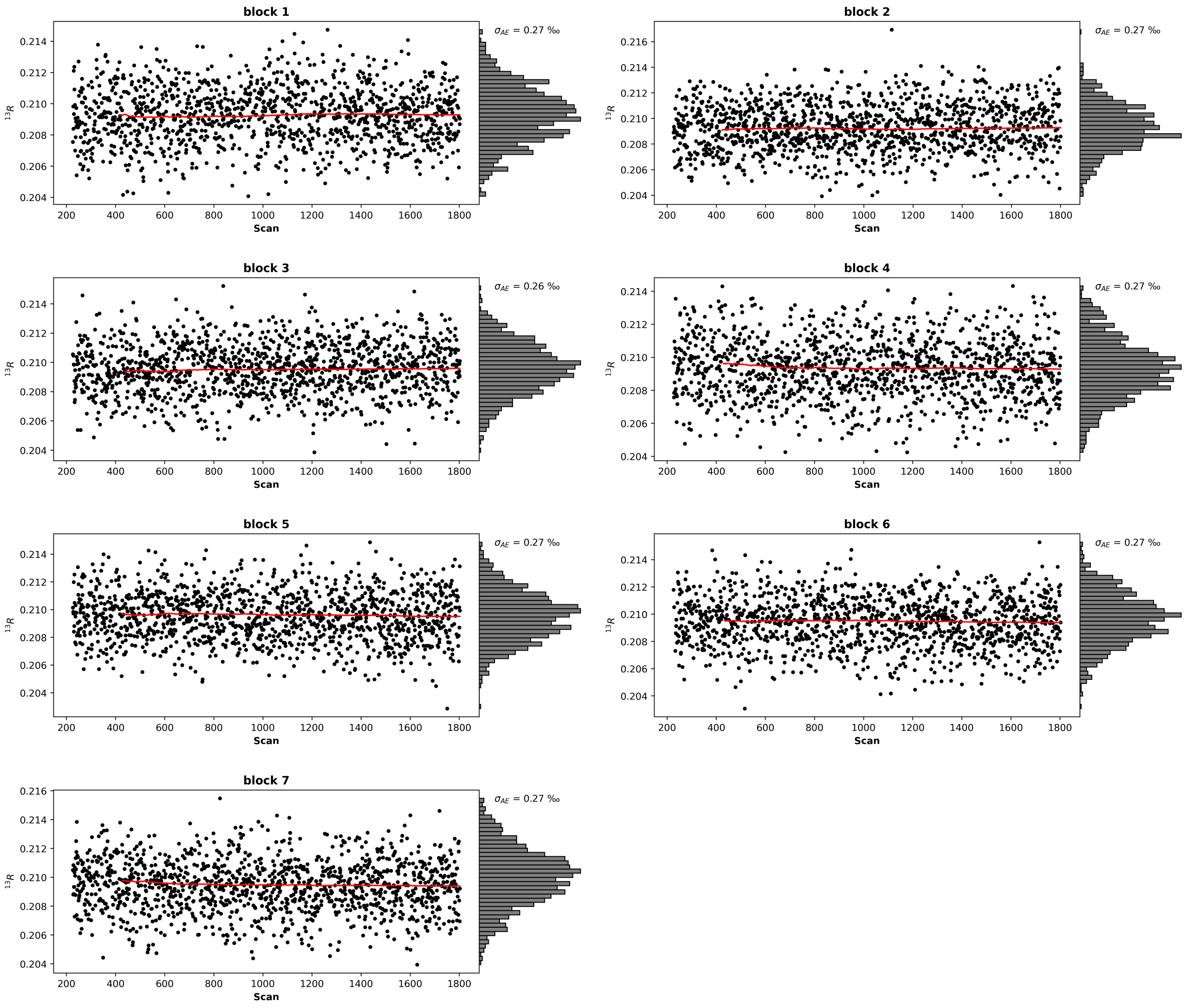

Acquisition Error and Shot-Noise

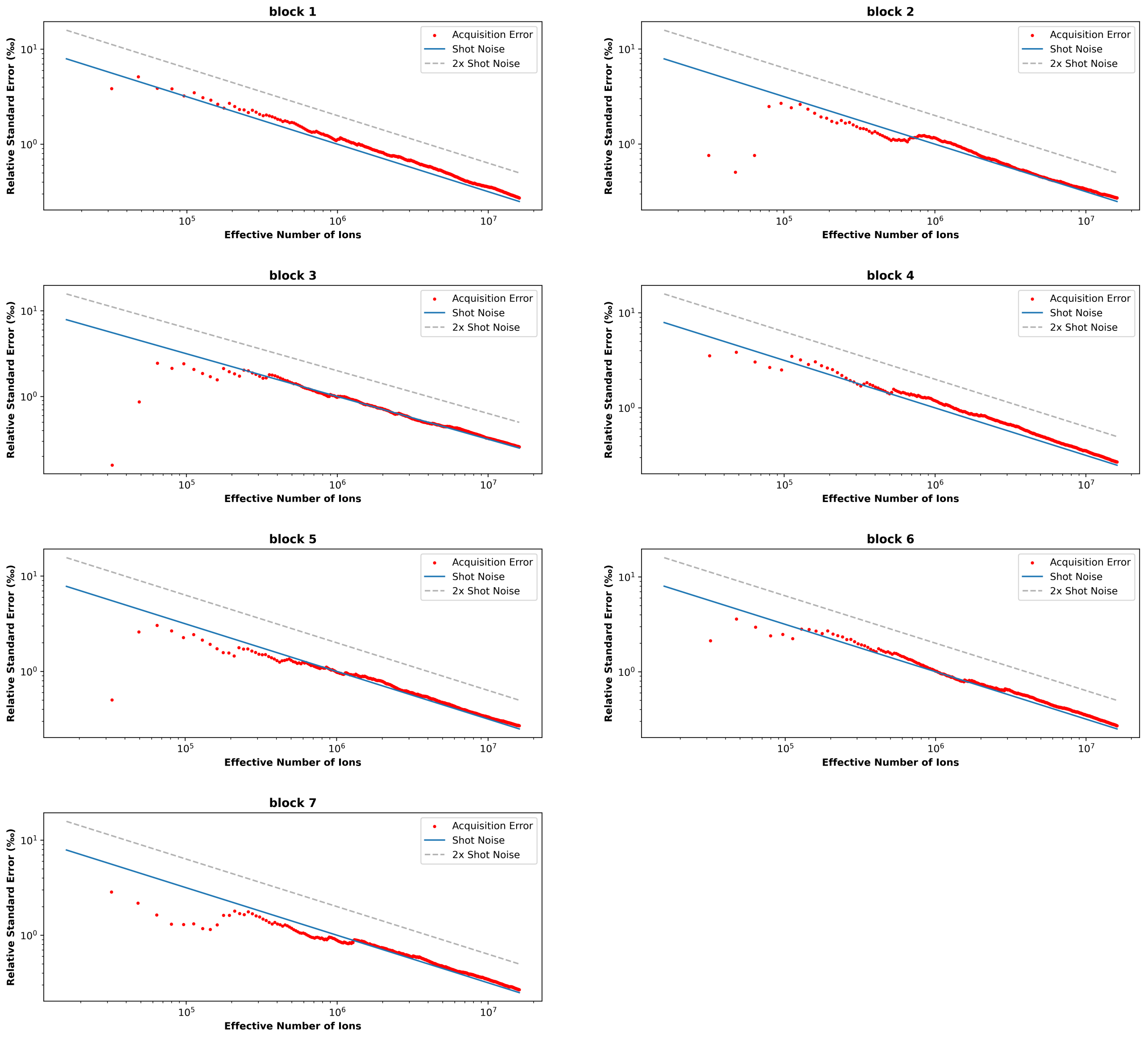

3. Delta Informations

Deltas were calculated by 'Average Of Neighboring Block Ratios'

3.1. 13C

Delta 13C was corrected by -27.80

Block	SEM	Delta corrected	Delta
2	0.24	-28.29	-0.50
4	0.24	-29.01	-1.24
6	0.24	-28.00	-0.20

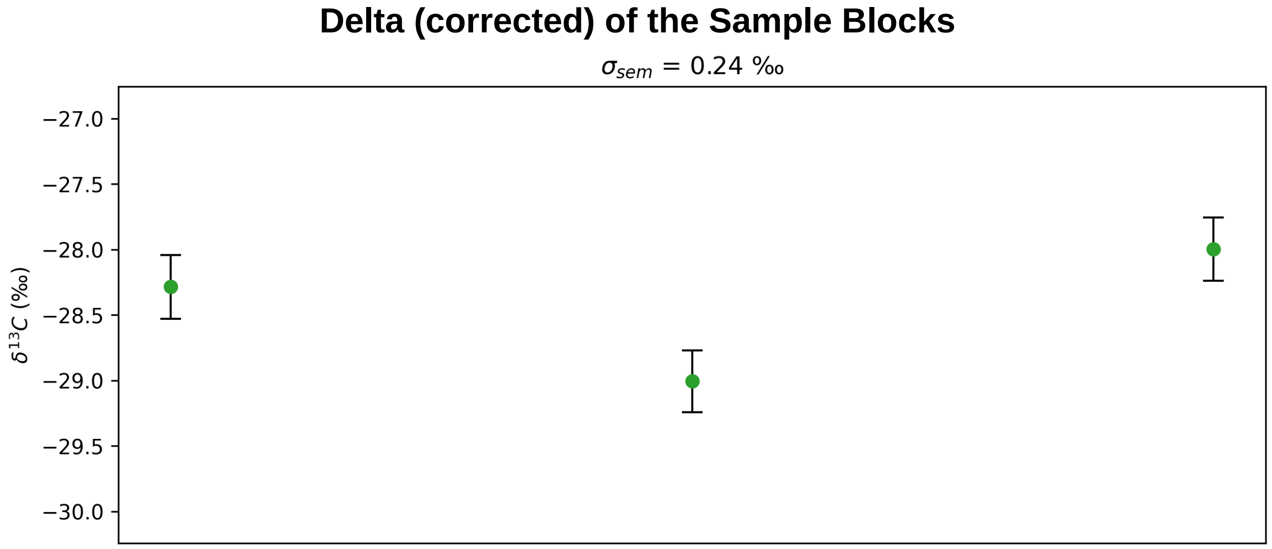

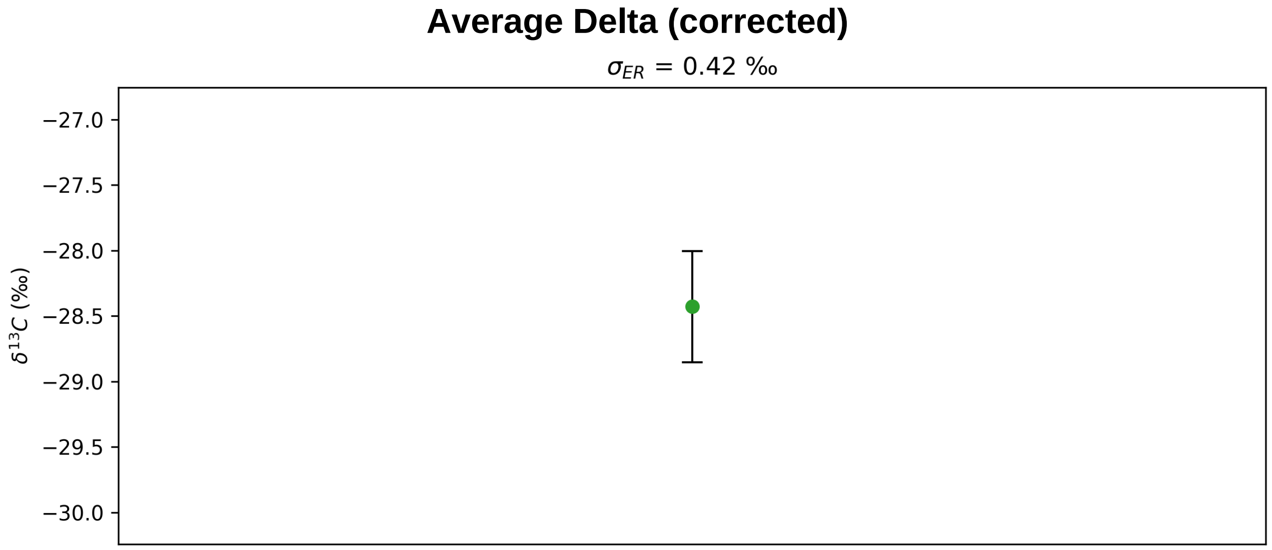

The final corrected average delta was -28.43 with a standard deviation of 0.42. Here the standard deviation is called reproducibility error.
