# Supplementary material for: Advancing Stable Isotope Analysis with Orbitrap-MS for Fatty Acid Methyl Esters and Complex Lipid Matrices
Source: J Am Soc Mass Spectrom. 2025 Jun 17;36(7):1527–35. doi: 10.1021/jasms.5c00092 (PMC12339014; doi:10.1021/jasms.5c00092)
Supplement: Supplementary file 2 [file js5c00092_si_002.zip › reports by IsotoPy Software/standards/Na+Standard3_DI.pdf]

Standard 3 - [M + Na]+
Isotope Analysis report from IsotoPy
Dual Inlet

1. Pre Processing

1.1. Block Time and Scan Information

Information about sample and standard block times and scans:

Block	Injected	Initial Time	End Time	Number of scans
1	standard	1	5	755
2	sample	6	10	728
3	standard	11	15	729
4	sample	16	20	756
5	standard	21	25	724
6	sample	26	30	723
7	standard	31	35	742

1.2. Outlier Removal

A total of 1147 scans were considered outliers and removed using the MAD method

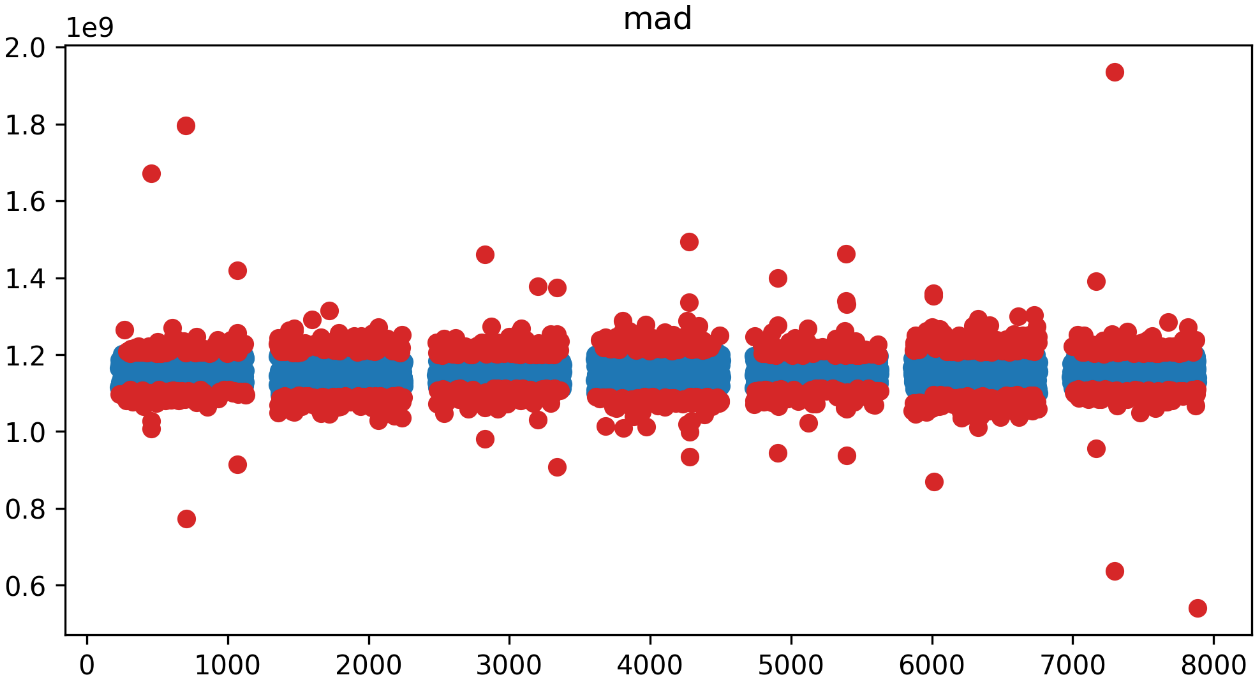

1.3. Total Ion Current (TIC)

TIC of all blocks

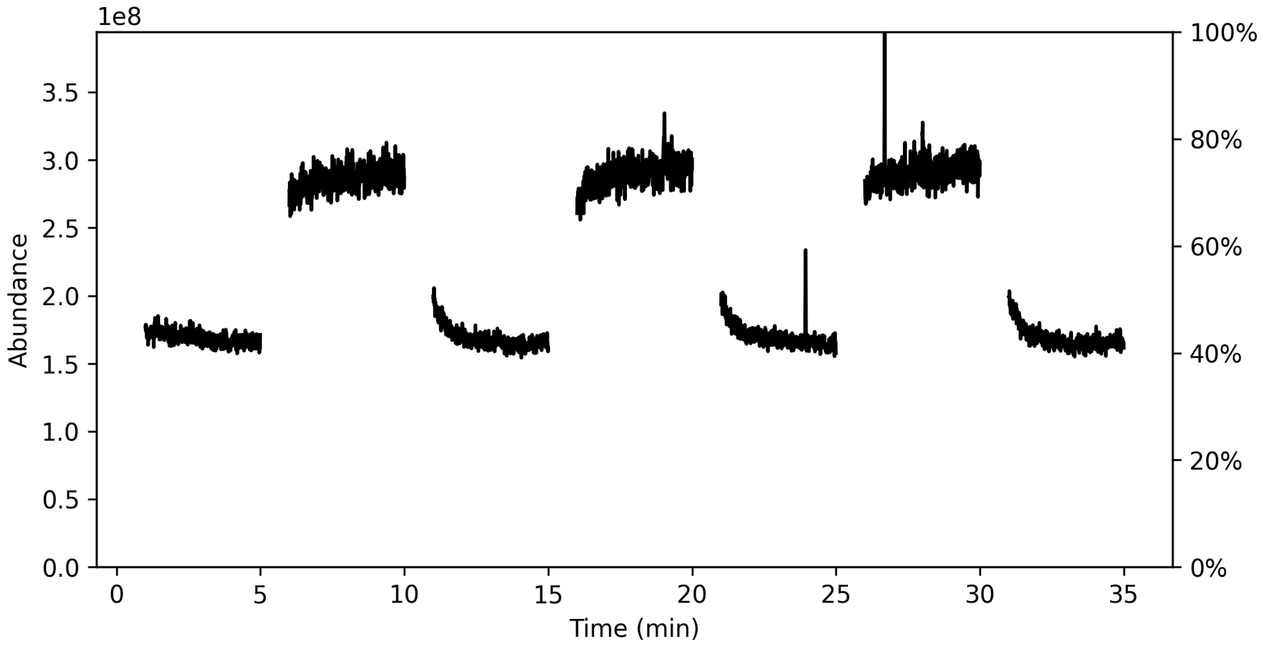

Block	TIC min	TIC max	TIC mean	RSD (%)
1	1.57e+08	1.85e+08	1.69e+08	2.69
2	2.59e+08	3.13e+08	2.86e+08	3.03
3	1.54e+08	2.06e+08	1.69e+08	4.63
4	2.56e+08	3.34e+08	2.89e+08	3.44
5	1.55e+08	2.34e+08	1.70e+08	4.87
6	2.68e+08	3.94e+08	2.92e+08	3.20
7	1.55e+08	2.03e+08	1.69e+08	4.87

2. Block Parameters

The Isotopic Ratio of the blocks were calculated by 'Mean'

2.1. 13C/M0

Block	Number of scans	Effective number of ions	Isotopic Ratio	STD	SEM	RSE
1	755	1.67e+07	0.210349	0.001383	0.000050	0.000239
2	728	1.61e+07	0.210155	0.001395	0.000052	0.000246
3	729	1.61e+07	0.210332	0.001368	0.000051	0.000241
4	756	1.67e+07	0.209855	0.001366	0.000050	0.000237
5	724	1.60e+07	0.210317	0.001421	0.000053	0.000251
6	723	1.60e+07	0.209931	0.001356	0.000050	0.000240
7	742	1.64e+07	0.210175	0.001343	0.000049	0.000234

Errors and Test Paramters

Block	Acquisition Error (permil)	Shot-Noise (permil)	AE/SN ratio	Shapiro Wilk (p_value)	D'Agostino (p_value)
1	0.239	0.245	0.976	0.681	0.776
2	0.246	0.249	0.986	0.681	0.563
3	0.241	0.249	0.966	0.583	0.901
4	0.237	0.245	0.966	0.665	0.534
5	0.251	0.250	1.004	0.844	0.975
6	0.240	0.250	0.959	0.127	0.059
7	0.234	0.247	0.949	0.777	0.617

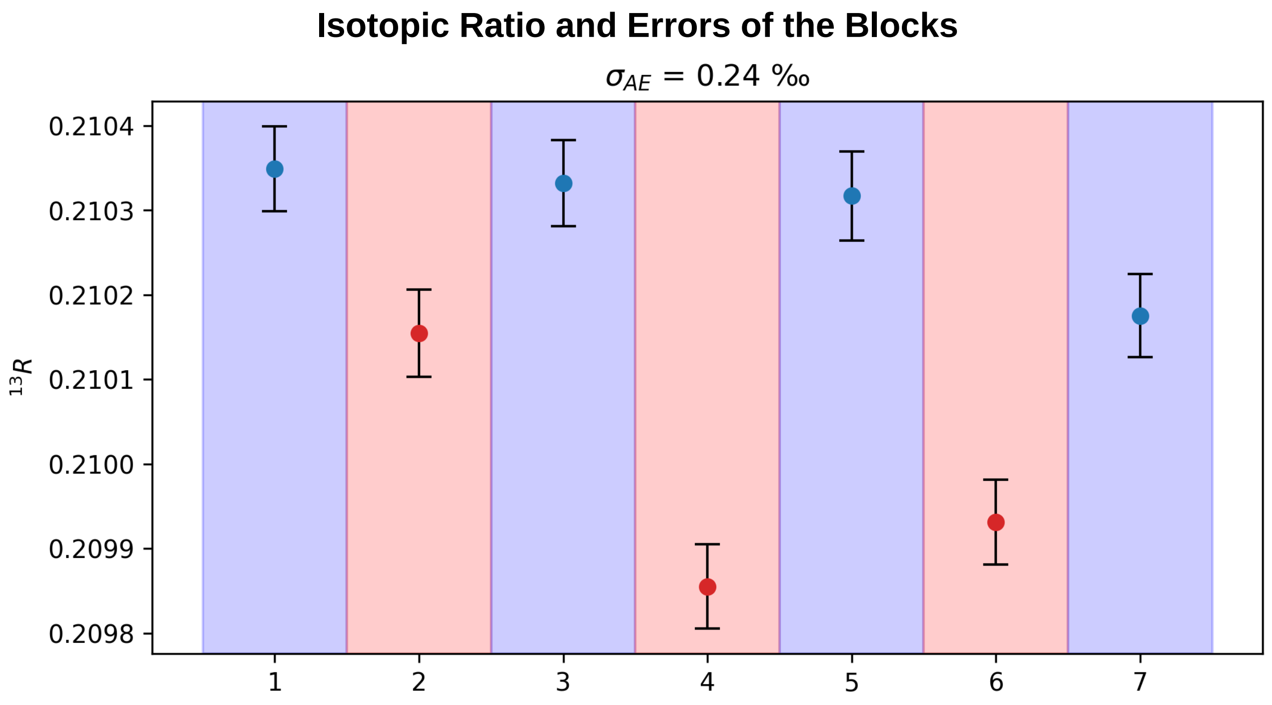
Isotopic Ratio and Errors of the Blocks
σ_AE = 0.24 ‰
13R
0.2104
0.2103
0.2102
0.2101
0.2100
0.2099
0.2098
1
2
3
4
5
6
7

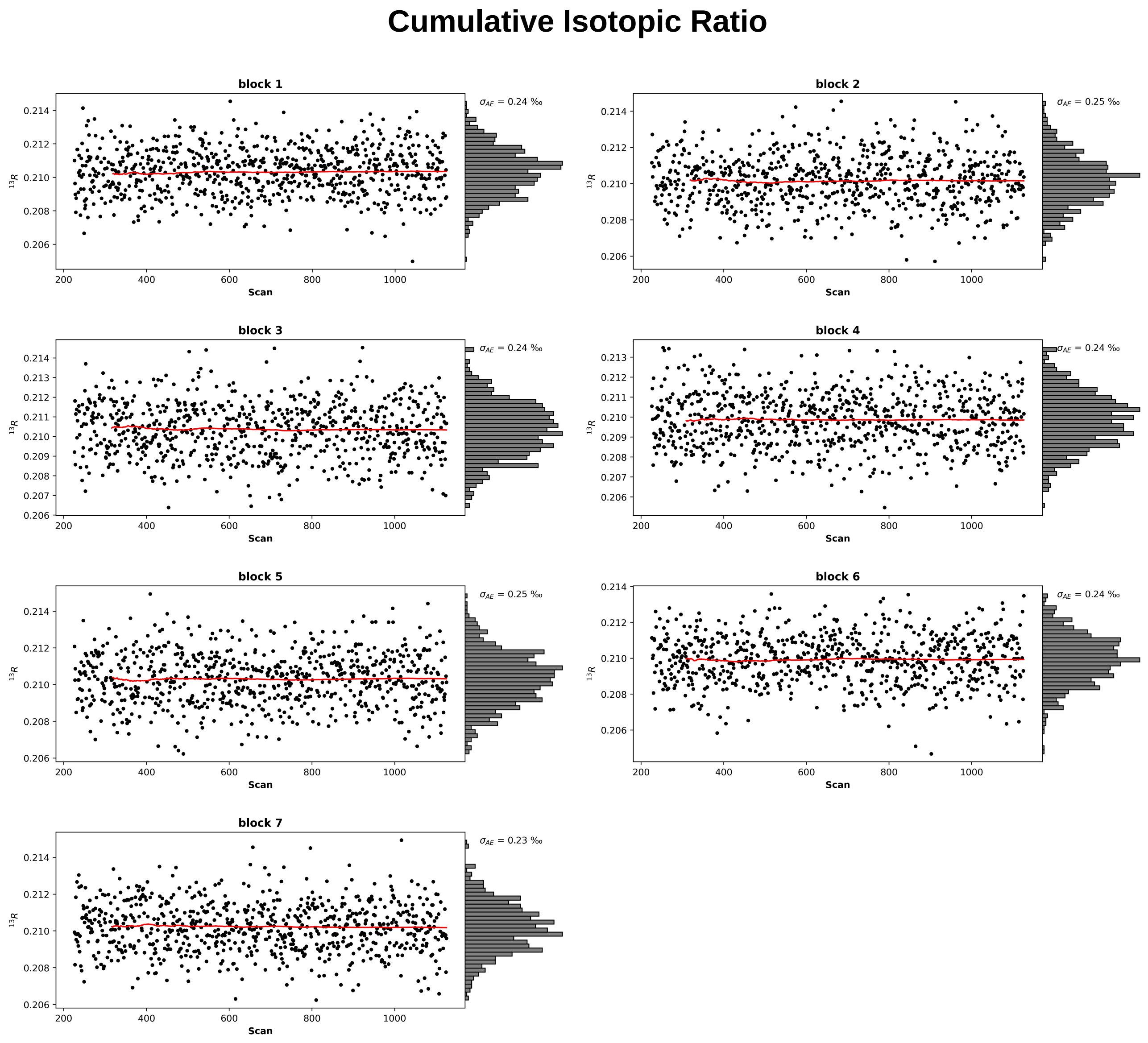
Cumulative Isotopic Ratio
block 1
σ_AE = 0.24 ‰
block 2
σ_AE = 0.25 ‰
block 3
σ_AE = 0.24 ‰
block 4
σ_AE = 0.24 ‰
block 5
σ_AE = 0.25 ‰
block 6
σ_AE = 0.24 ‰
block 7
σ_AE = 0.23 ‰
13R
Scan

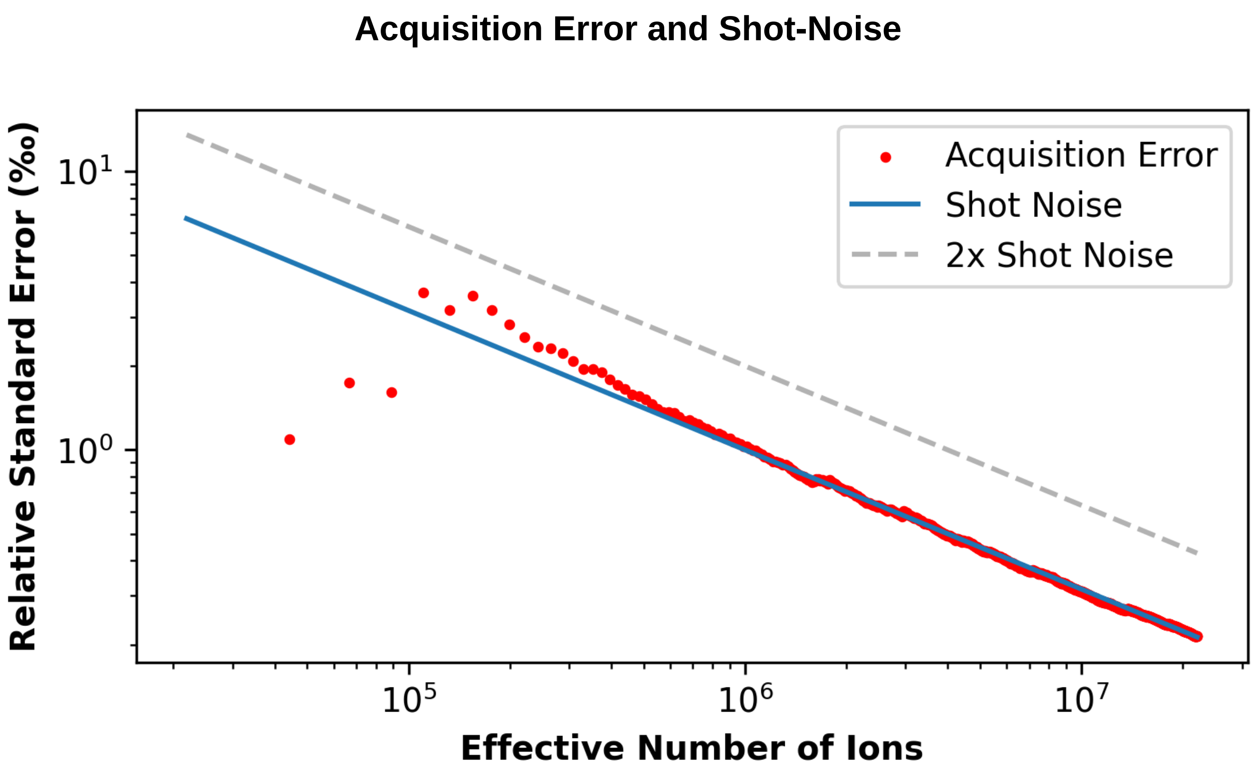
Acquisition Error and Shot-Noise
Relative Standard Error (‰)
10^1
10^0
10^5
10^6
10^7
Effective Number of Ions
Acquisition Error
Shot Noise
2x Shot Noise

3. Delta Informations

Deltas were calculated by 'Average Of Neighboring Block Ratios'

3.1. 13C

Delta 13C was corrected by -27.80

Block	SEM	Delta corrected	Delta
2	0.25	-28.66	-0.88
4	0.24	-29.97	-2.23
6	0.24	-29.26	-1.50

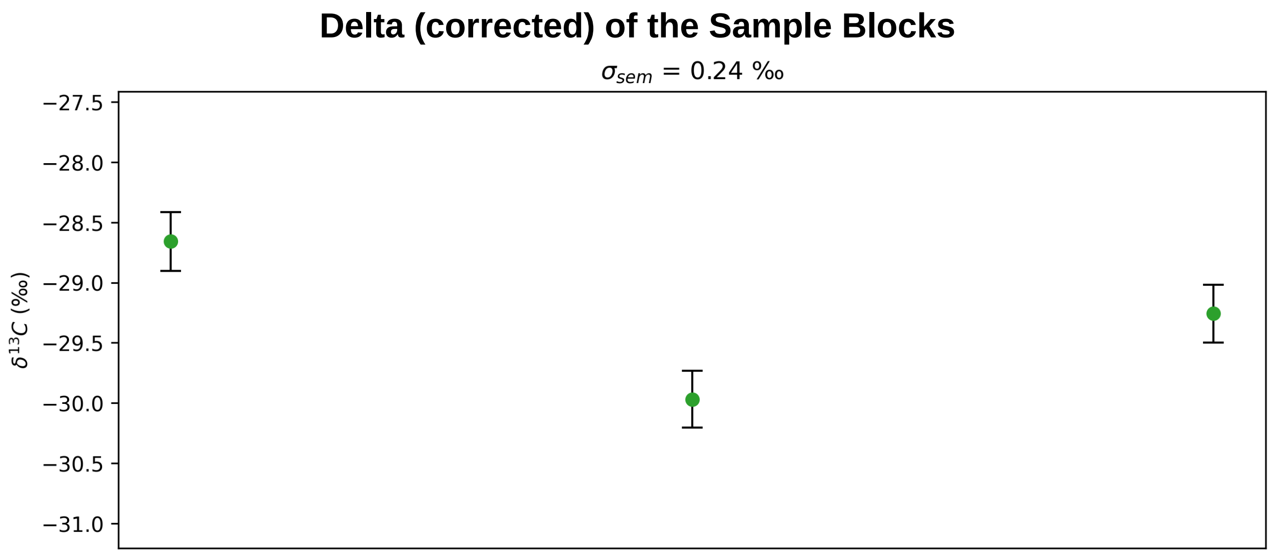

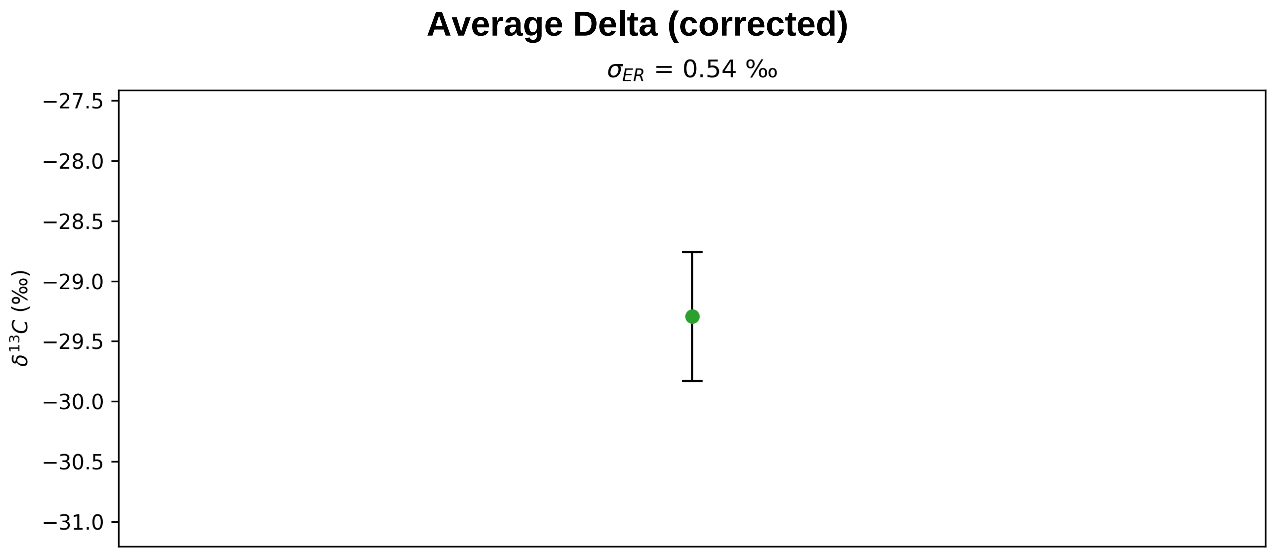

The final corrected average delta was -29.29 with a standard deviation of 0.54. Here the standard deviation is called reproducibility error.
