# Supplementary material for: Advancing Stable Isotope Analysis with Orbitrap-MS for Fatty Acid Methyl Esters and Complex Lipid Matrices
Source: J Am Soc Mass Spectrom. 2025 Jun 17;36(7):1527–35. doi: 10.1021/jasms.5c00092 (PMC12339014; doi:10.1021/jasms.5c00092)
Supplement: Supplementary file 2 [file js5c00092_si_002.zip › reports by IsotoPy Software/standards/H+Standard1_FI.pdf]

Standard 1 - [M + H]+
Isotope Analysis report from IsotoPy

Flow Injection

1. Pre Processing

1.1. Block Time and Scan Information

Information about sample and standard block times and scans:

Block	Injected	Initial Time	End Time	Number of scans
1	standard	1	8	1283
2	sample	16	23	1280
3	standard	31	38	1276
4	sample	46	53	1305
5	standard	61	68	1279
6	sample	76	83	1287
7	standard	91	98	1301

1.2. Outlier Removal

A total of 2028 scans were considered outliers and removed using the MAD method

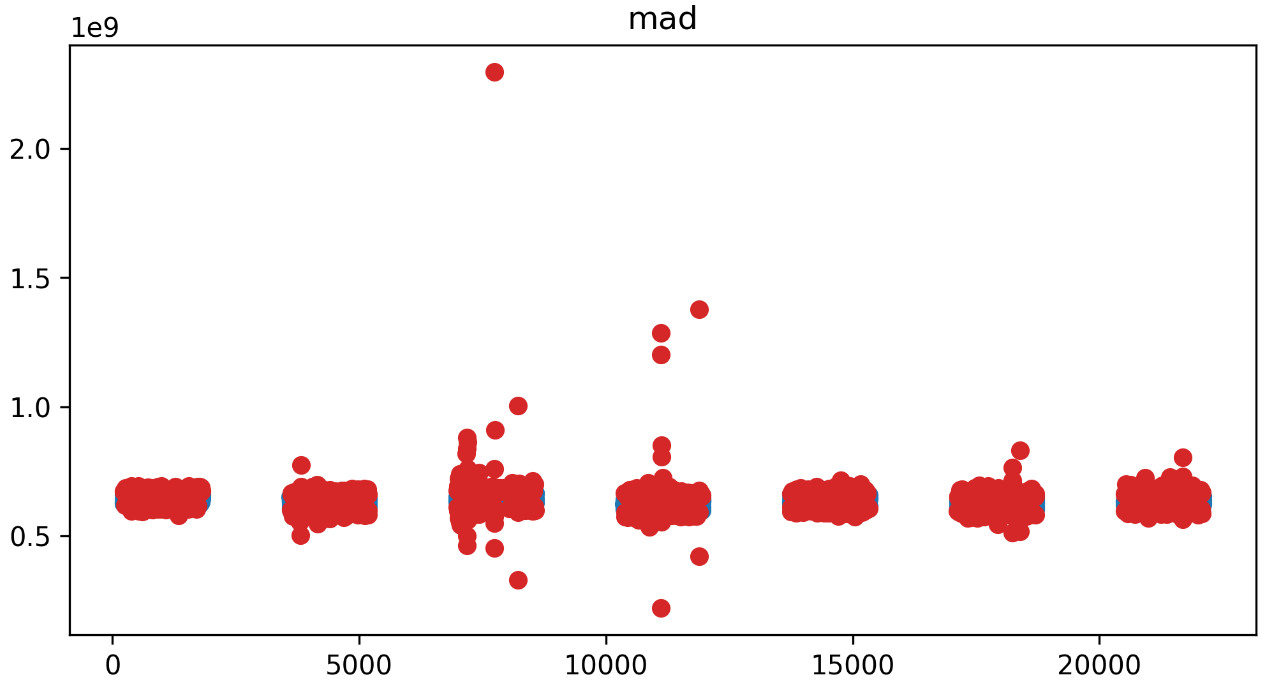

1.3. Total Ion Current (TIC)

TIC of all blocks

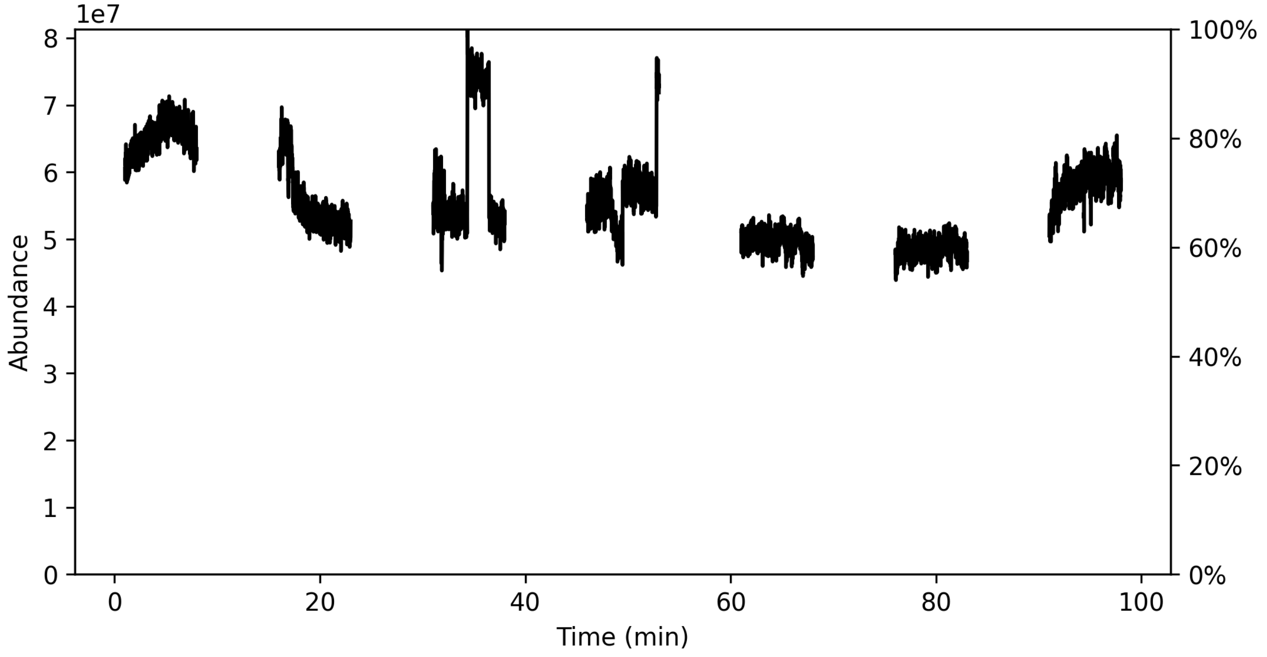

Block	TIC min	TIC max	TIC mean	RSD (%)
1	5.84e+07	7.13e+07	6.51e+07	3.77
2	4.82e+07	6.97e+07	5.54e+07	8.26
3	4.53e+07	8.13e+07	6.05e+07	16.24
4	4.62e+07	7.70e+07	5.63e+07	7.52
5	4.45e+07	5.36e+07	4.97e+07	2.94
6	4.39e+07	5.24e+07	4.85e+07	2.64
7	4.96e+07	6.55e+07	5.85e+07	4.29

2. Block Parameters

The Isotopic Ratio of the blocks were calculated by 'Mean'

2.1. 13C/M0

Block	Number of scans	Effective number of ions	Isotopic Ratio	STD	SEM	RSE
1	1283	1.73e+07	0.209272	0.001794	0.000050	0.000239
2	1280	1.75e+07	0.209244	0.001721	0.000048	0.000230
3	1276	1.74e+07	0.209414	0.001764	0.000049	0.000236
4	1305	1.77e+07	0.209540	0.001749	0.000048	0.000231
5	1279	1.73e+07	0.210204	0.001755	0.000049	0.000233
6	1287	1.73e+07	0.209947	0.001757	0.000049	0.000233
7	1301	1.75e+07	0.210194	0.001770	0.000049	0.000233

Errors and Test Paramters

Block	Acquisition Error (permil)	Shot-Noise (permil)	AE/SN ratio	Shapiro Wilk (p_value)	D'Agostino (p_value)
1	0.239	0.241	0.994	0.585	0.594
2	0.230	0.239	0.961	0.091	0.186
3	0.236	0.239	0.984	0.955	0.740
4	0.231	0.238	0.973	0.103	0.084
5	0.233	0.240	0.971	0.821	0.977
6	0.233	0.241	0.970	0.541	0.798
7	0.233	0.239	0.976	0.747	0.993

Isotopic Ratio and Errors of the Blocks

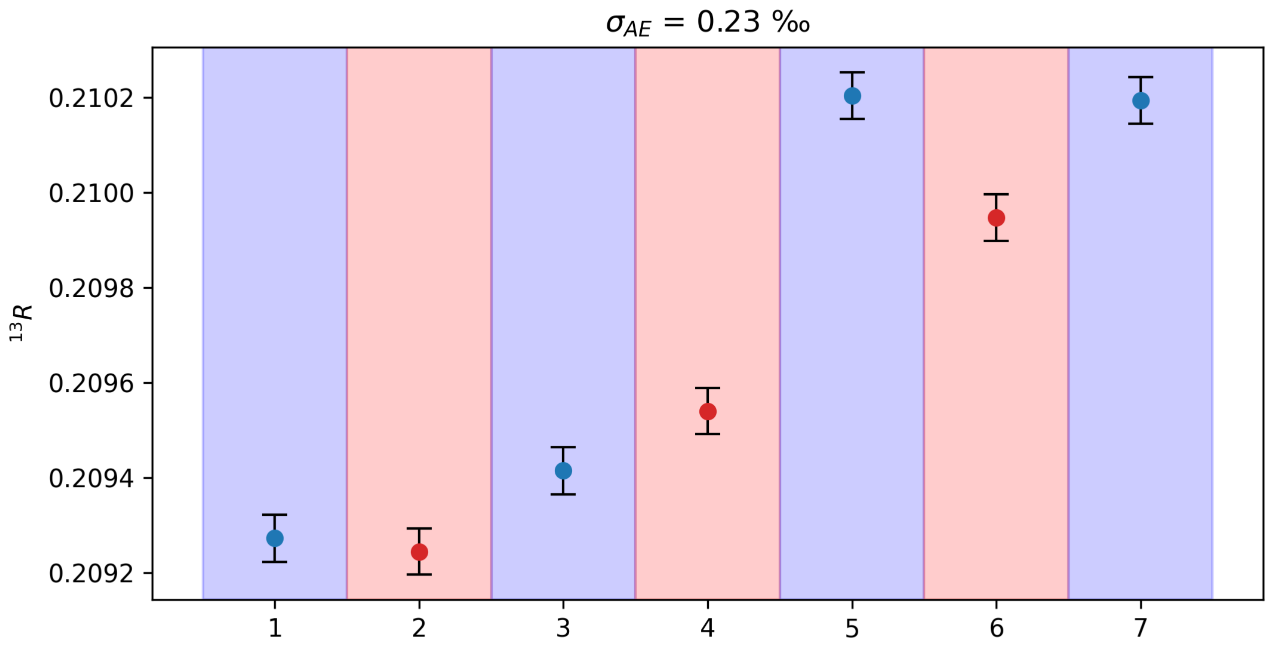

Cumulative Isotopic Ratio

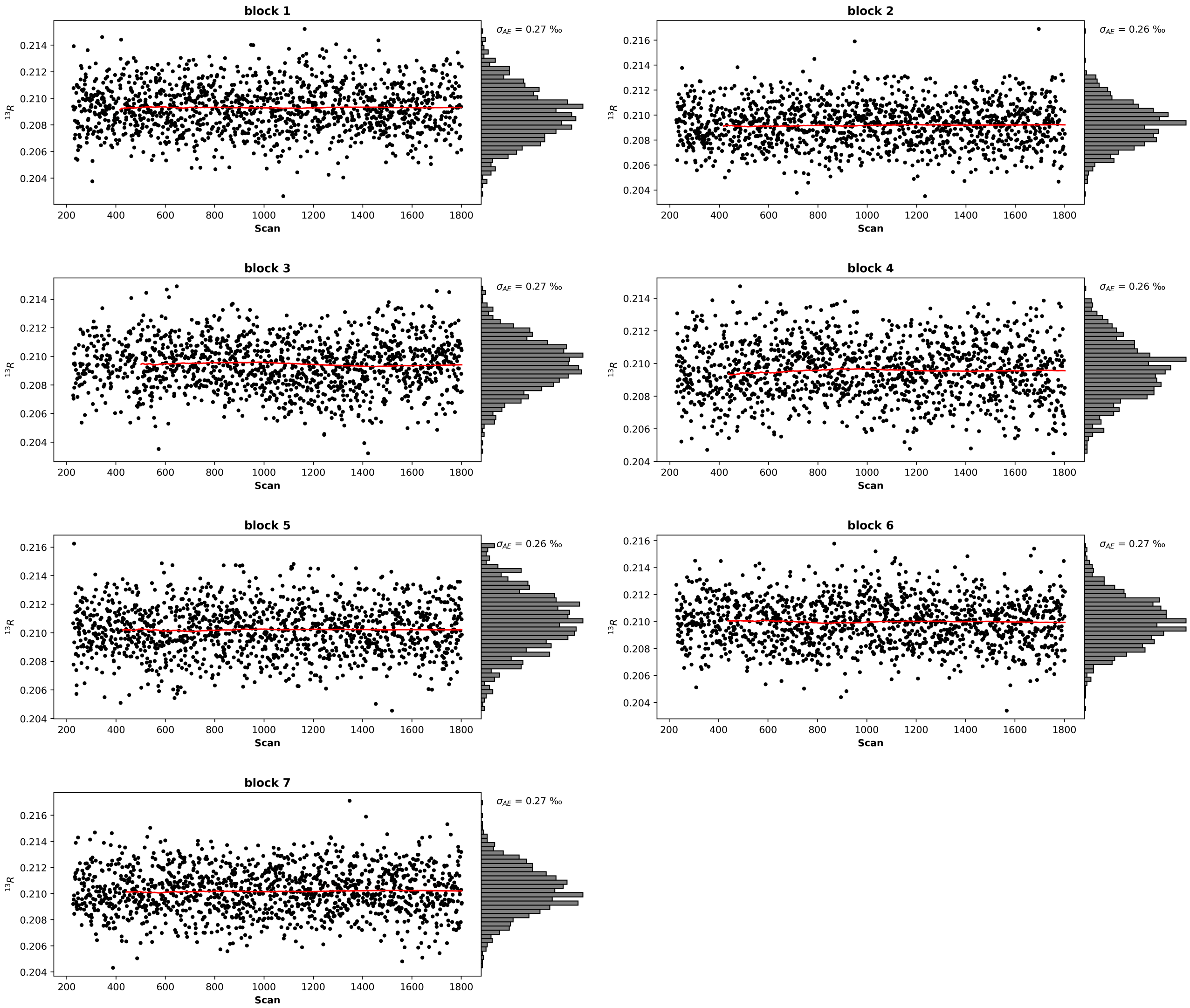

Acquisition Error and Shot-Noise

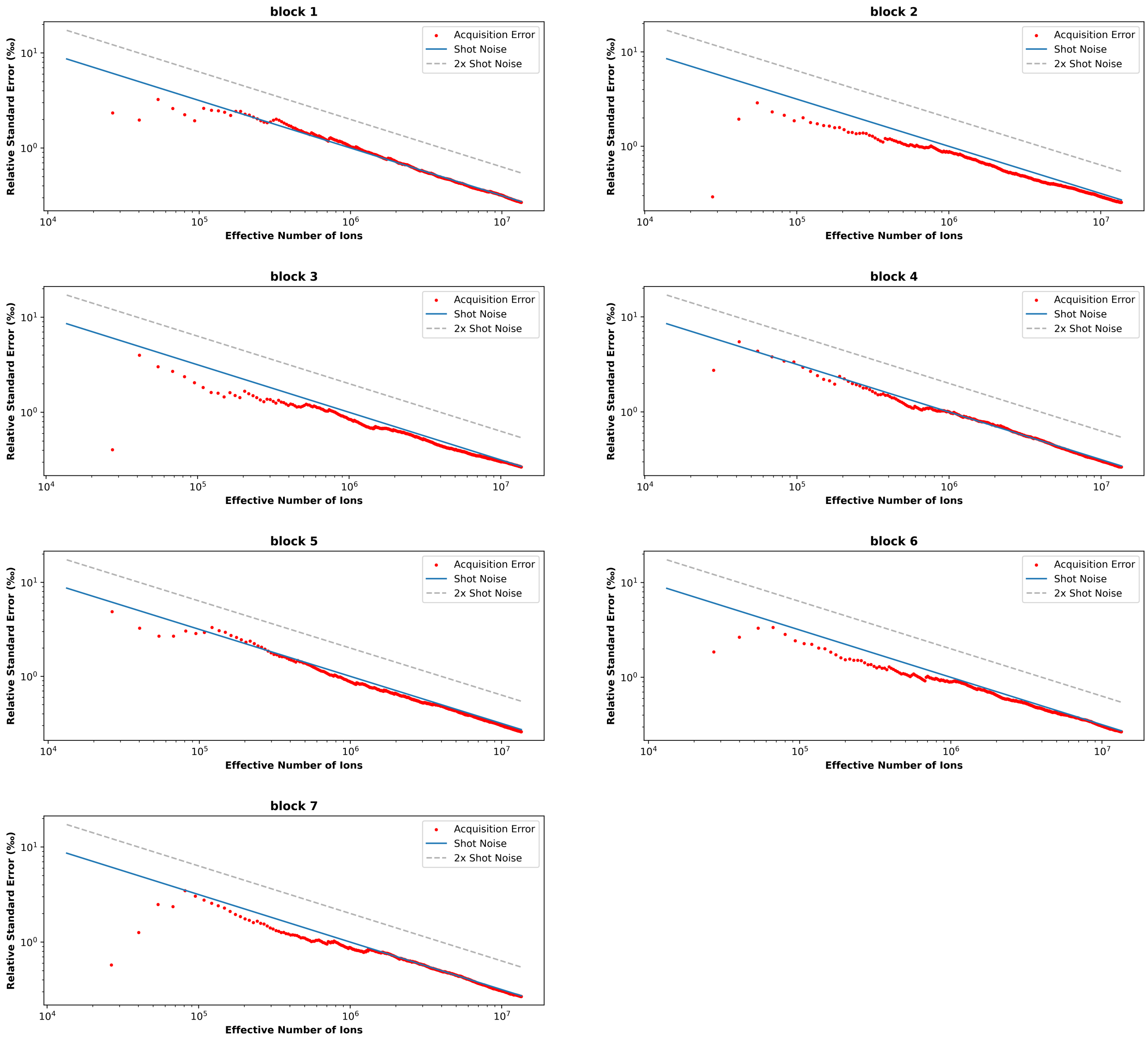

3. Delta Informations

Deltas were calculated by 'Average Of Neighboring Block Ratios'

3.1. 13C

Delta 13C was corrected by -27.80

Block	SEM	Delta corrected	Delta
2	0.23	-28.26	-0.47
4	0.23	-29.05	-1.28
6	0.23	-28.96	-1.20

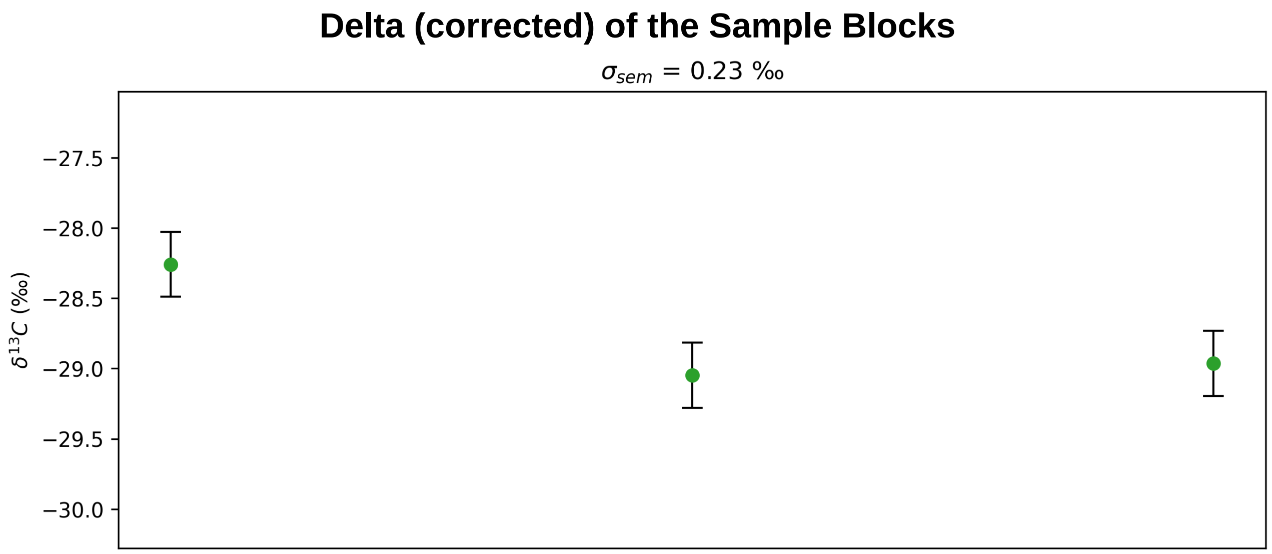

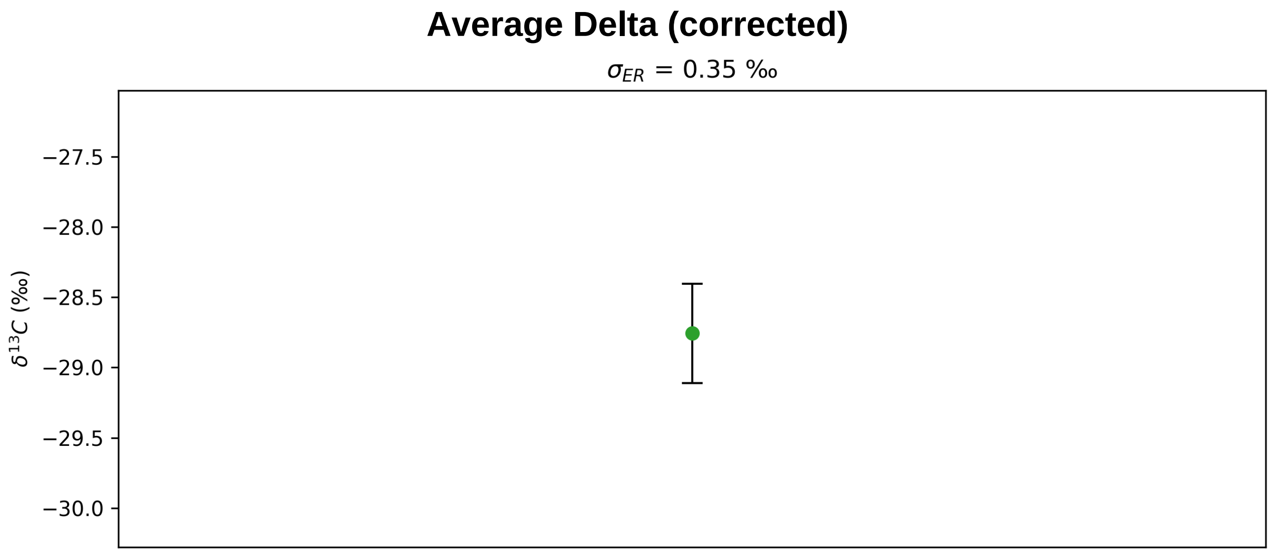

The final corrected average delta was -28.76 with a standard deviation of 0.35. Here the standard deviation is called reproducibility error.
