# Supplementary material for: Advancing Stable Isotope Analysis with Orbitrap-MS for Fatty Acid Methyl Esters and Complex Lipid Matrices
Source: J Am Soc Mass Spectrom. 2025 Jun 17;36(7):1527–35. doi: 10.1021/jasms.5c00092 (PMC12339014; doi:10.1021/jasms.5c00092)
Supplement: Supplementary file 2 [file js5c00092_si_002.zip › reports by IsotoPy Software/standards/H+Standard6_DI.pdf]

Standard 6 - [M + H]+

Isotope Analysis report from IsotoPy

Dual Inlet

1. Pre Processing

1.1. Block Time and Scan Information

Information about sample and standard block times and scans:

Block	Injected	Initial Time	End Time	Number of scans
1	standard	1	5	740
2	sample	6	10	713
3	standard	11	15	723
4	sample	16	20	718
5	standard	21	25	749
6	sample	26	30	746
7	standard	31	35	753

1.2. Outlier Removal

A total of 1165 scans were considered outliers and removed using the MAD method

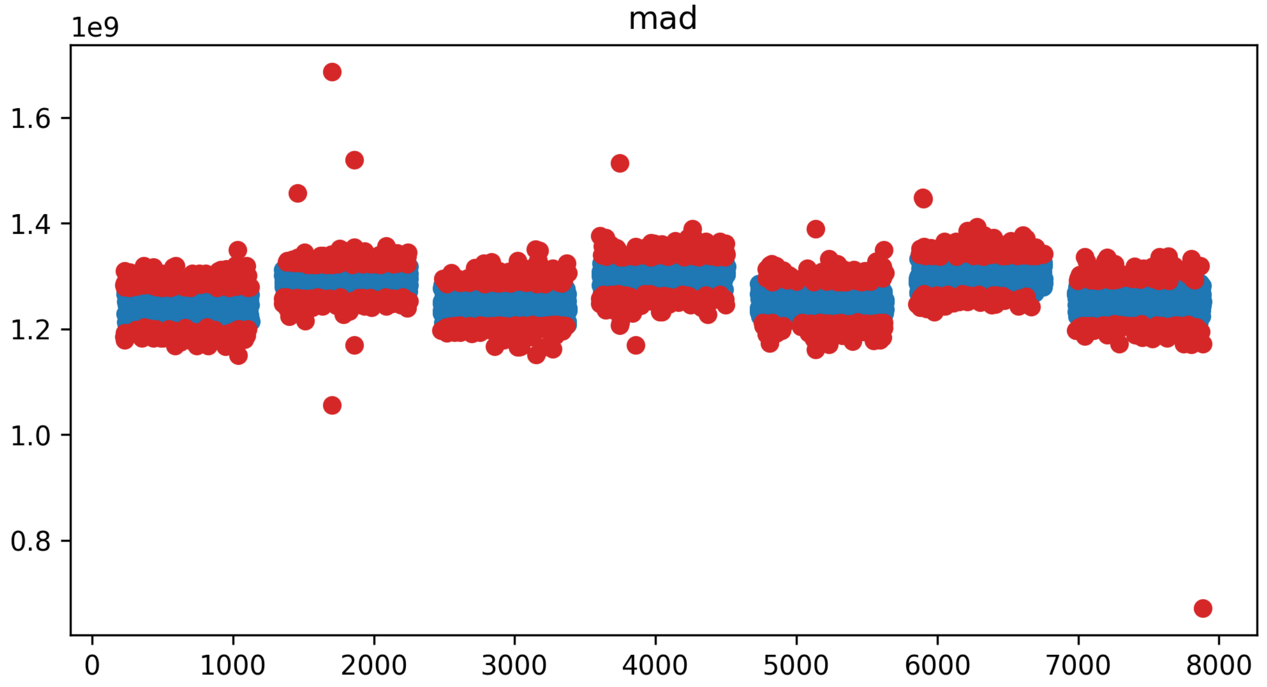

1.3. Total Ion Current (TIC)

TIC of all blocks

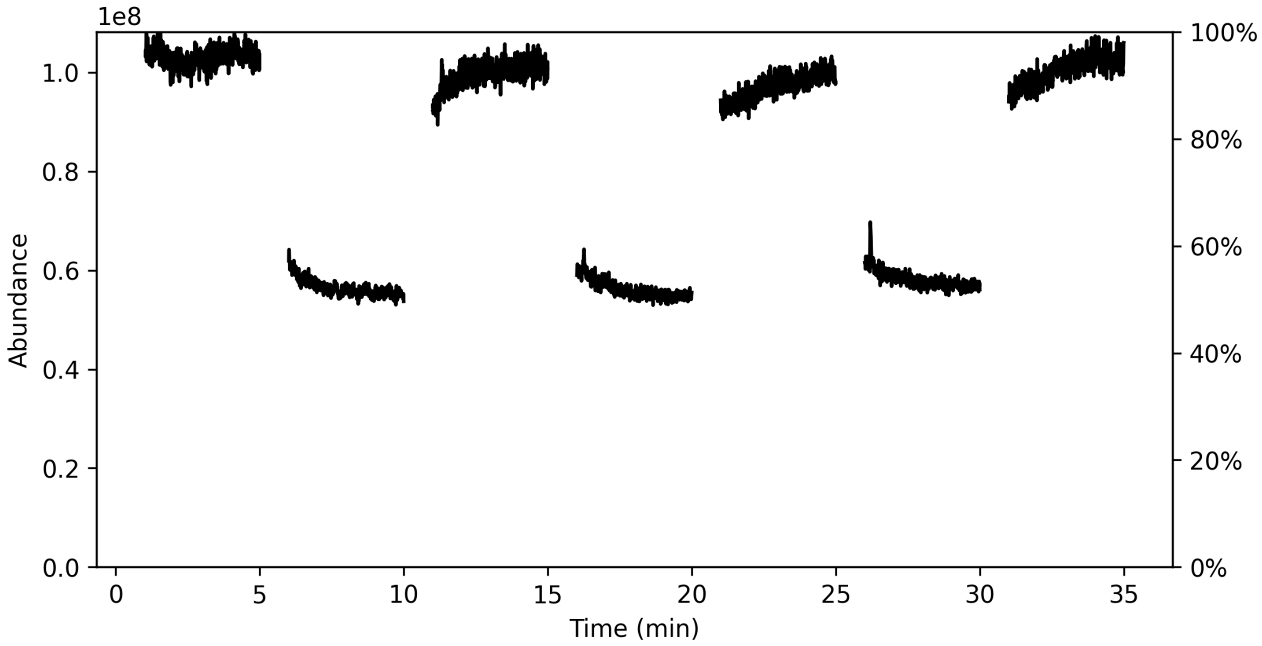

Block	TIC min	TIC max	TIC mean	RSD (%)
1	9.72e+07	1.08e+08	1.03e+08	1.77
2	5.30e+07	6.42e+07	5.64e+07	2.93
3	8.94e+07	1.06e+08	9.96e+07	2.65
4	5.29e+07	6.42e+07	5.62e+07	3.27
5	9.05e+07	1.03e+08	9.70e+07	2.73
6	5.49e+07	6.97e+07	5.81e+07	2.67
7	9.26e+07	1.07e+08	1.00e+08	2.84

2. Block Parameters

The Isotopic Ratio of the blocks were calculated by 'Mean'

2.1. 13C/M0

Block	Number of scans	Effective number of ions	Isotopic Ratio	STD	SEM	RSE
1	740	1.33e+07	0.217419	0.001356	0.000050	0.000229
2	713	1.27e+07	0.216932	0.001386	0.000052	0.000239
3	723	1.28e+07	0.217549	0.001391	0.000052	0.000238
4	718	1.27e+07	0.216952	0.001421	0.000053	0.000244
5	749	1.31e+07	0.217570	0.001391	0.000051	0.000233
6	746	1.30e+07	0.217109	0.001377	0.000050	0.000232
7	753	1.31e+07	0.217519	0.001370	0.000050	0.000229

Errors and Test Paramters

Block	Acquisition Error (permil)	Shot-Noise (permil)	AE/SN ratio	Shapiro Wilk (p_value)	D'Agostino (p_value)
1	0.229	0.274	0.835	0.100	0.073
2	0.239	0.281	0.852	0.540	0.400
3	0.238	0.279	0.852	0.784	0.414
4	0.244	0.281	0.869	0.010	0.025
5	0.233	0.276	0.846	0.691	0.407
6	0.232	0.277	0.838	0.208	0.140
7	0.229	0.276	0.832	0.378	0.180

Isotopic Ratio and Errors of the Blocks

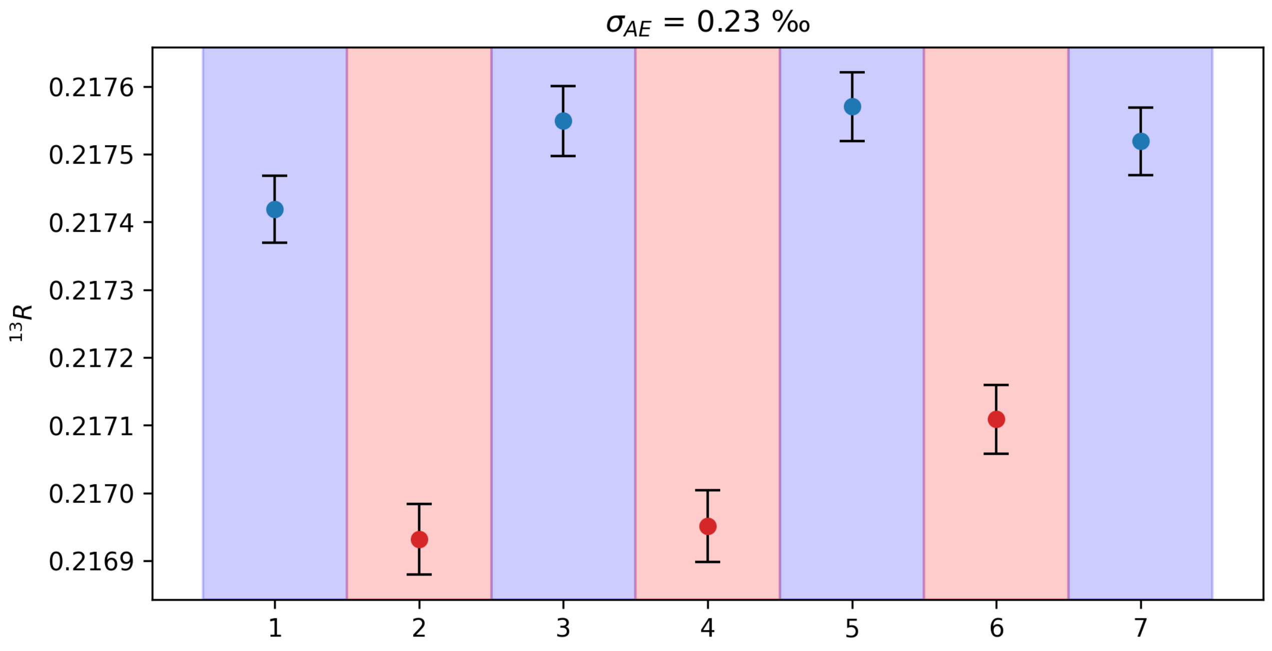

Cumulative Isotopic Ratio

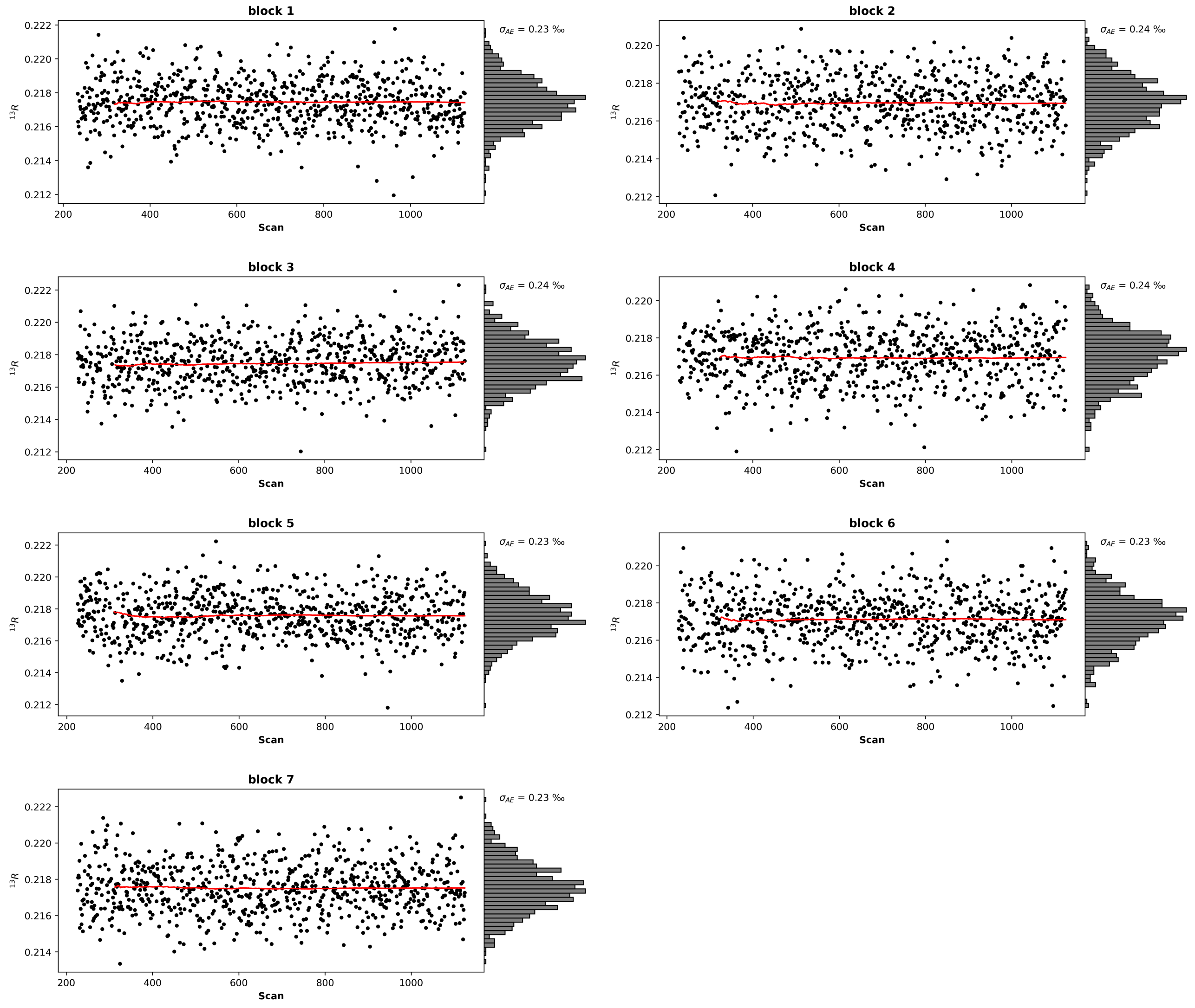

Acquisition Error and Shot-Noise

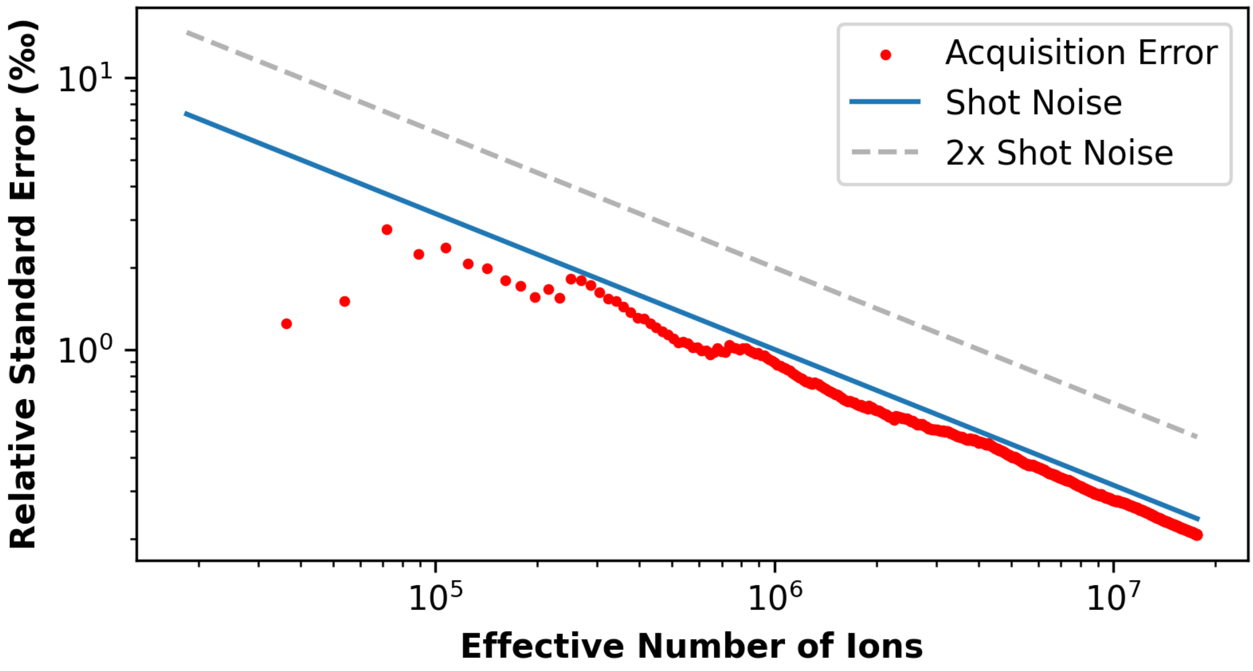

3. Delta Informations

Deltas were calculated by 'Average Of Neighboring Block Ratios'

3.1. 13C

Delta 13C was corrected by -27.80

Block	SEM	Delta corrected	Delta
2	0.24	-30.27	-2.54
4	0.24	-30.52	-2.80
6	0.23	-29.75	-2.00

Delta (corrected) of the Sample Blocks

σ_{sem} = 0.24 ‰

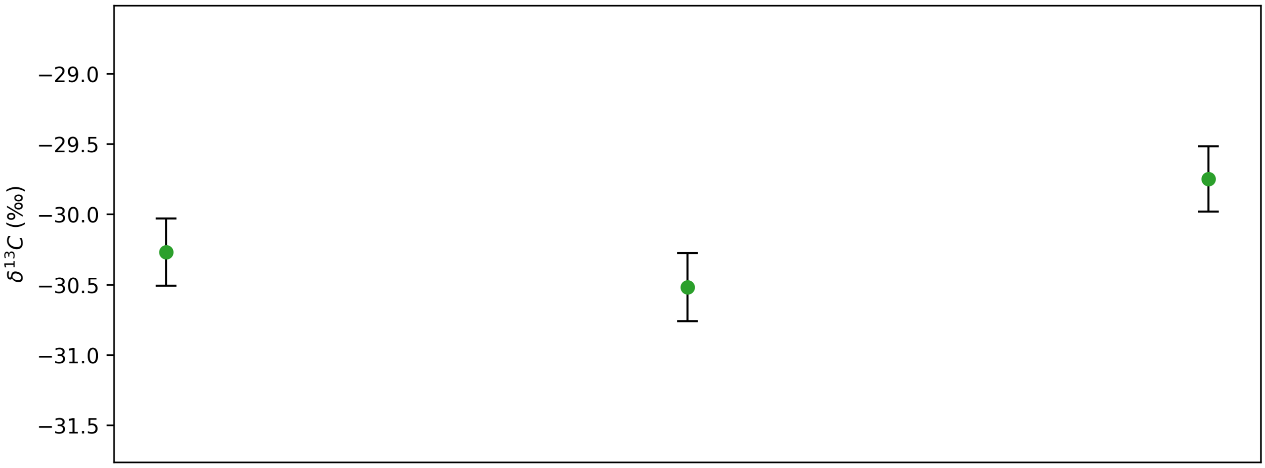

Average Delta (corrected)

σ_{ER} = 0.32 ‰

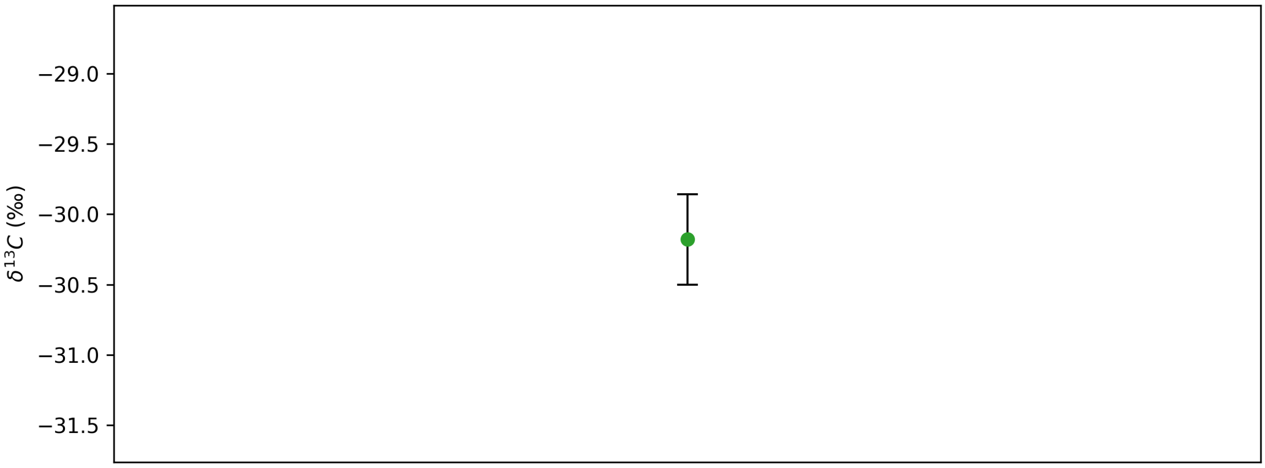

The final corrected average delta was -30.18 with a standard deviation of 0.32. Here the standard deviation is called reproducibility error.
