# Supplementary material for: Advancing Stable Isotope Analysis with Orbitrap-MS for Fatty Acid Methyl Esters and Complex Lipid Matrices
Source: J Am Soc Mass Spectrom. 2025 Jun 17;36(7):1527–35. doi: 10.1021/jasms.5c00092 (PMC12339014; doi:10.1021/jasms.5c00092)
Supplement: Supplementary file 2 [file js5c00092_si_002.zip › reports by IsotoPy Software/standards/H+Standard3_FI.pdf]

Standard 3 - [M + H]+

Isotope Analysis report from IsotoPy

Flow Injection

1. Pre Processing

1.1. Block Time and Scan Information

Information about sample and standard block times and scans:

Block	Injected	Initial Time	End Time	Number of scans
1	standard	1	8	1307
2	sample	16	23	1290
3	standard	31	38	1292
4	sample	46	53	1290
5	standard	61	68	1304
6	sample	76	83	1341
7	standard	91	98	1336

1.2. Outlier Removal

A total of 1879 scans were considered outliers and removed using the MAD method

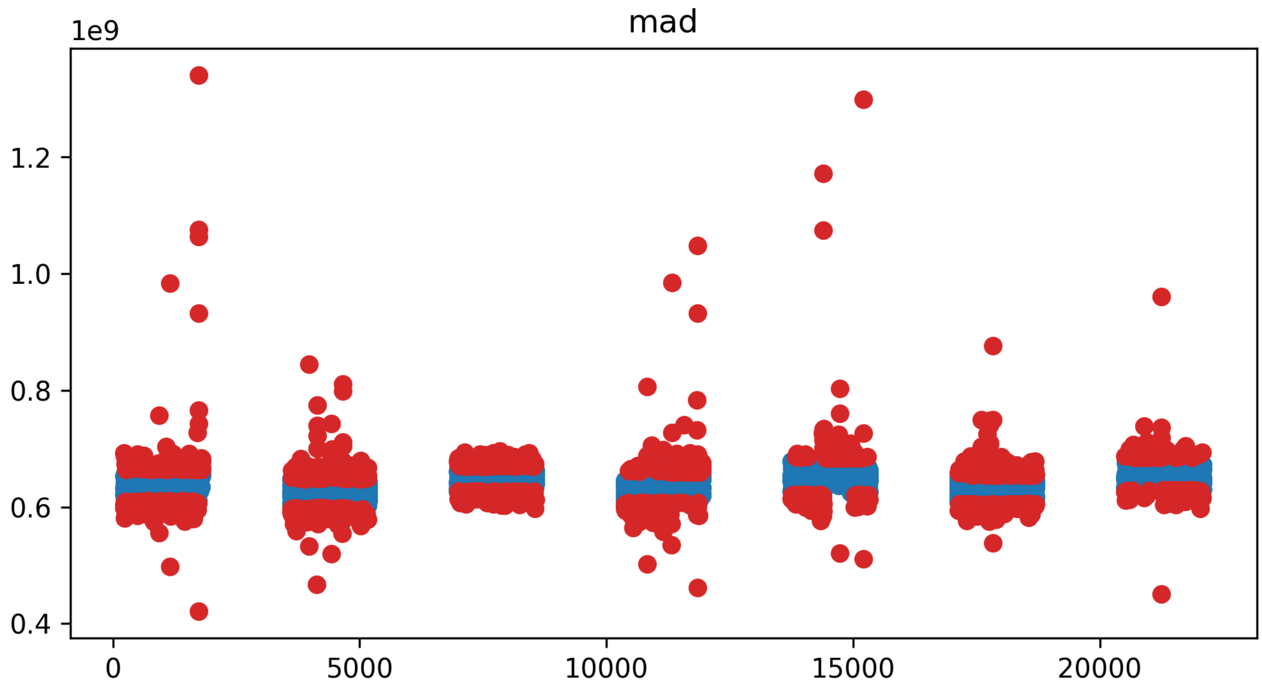

1.3. Total Ion Current (TIC)

TIC of all blocks

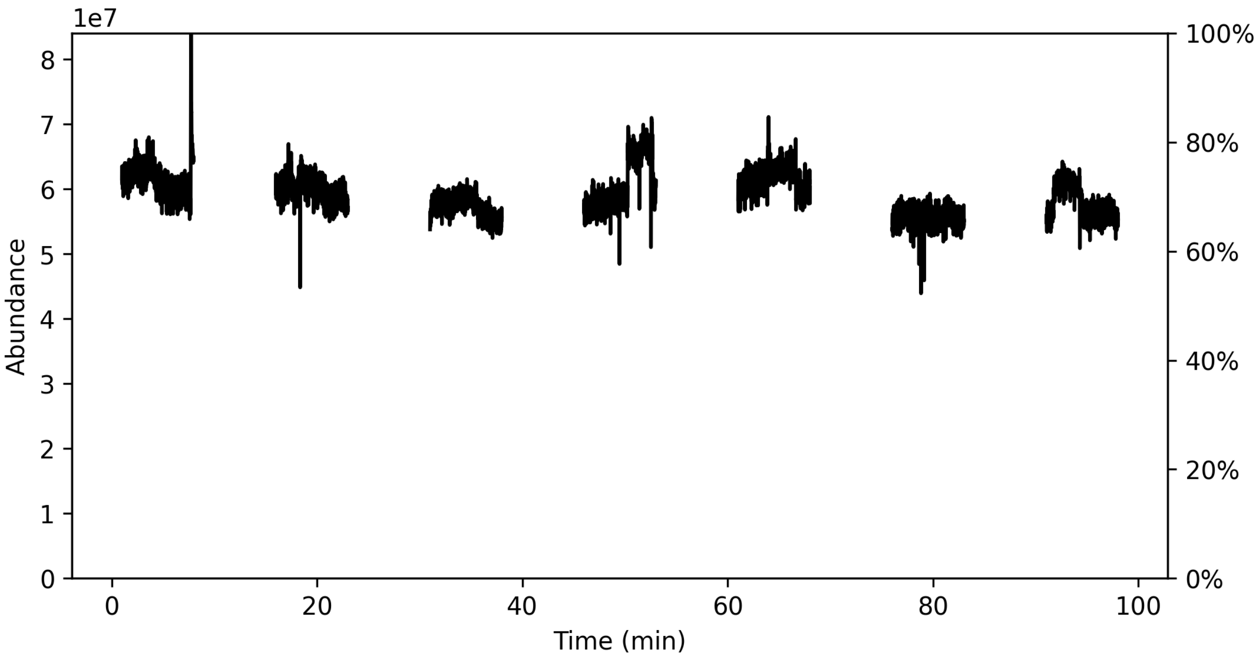

Block	TIC min	TIC max	TIC mean	RSD (%)
1	5.54e+07	8.40e+07	6.14e+07	4.20
2	4.48e+07	6.69e+07	5.99e+07	3.00
3	5.24e+07	6.15e+07	5.73e+07	2.76
4	4.85e+07	7.10e+07	6.08e+07	6.65
5	5.65e+07	7.11e+07	6.16e+07	3.41
6	4.39e+07	5.93e+07	5.57e+07	2.39
7	5.09e+07	6.42e+07	5.78e+07	4.34

2. Block Parameters

The Isotopic Ratio of the blocks were calculated by 'Mean'

2.1. 13C/M0

Block	Number of scans	Effective number of ions	Isotopic Ratio	STD	SEM	RSE
1	1307	1.75e+07	0.209842	0.001737	0.000048	0.000229
2	1290	1.72e+07	0.209704	0.001737	0.000048	0.000231
3	1292	1.73e+07	0.209760	0.001679	0.000047	0.000223
4	1290	1.72e+07	0.209568	0.001773	0.000049	0.000236
5	1304	1.74e+07	0.209640	0.001806	0.000050	0.000238
6	1341	1.79e+07	0.209781	0.001687	0.000046	0.000220
7	1336	1.79e+07	0.209810	0.001784	0.000049	0.000232

Errors and Test Paramters

Block	Acquisition Error (permil)	Shot-Noise (permil)	AE/SN ratio	Shapiro Wilk (p_value)	D'Agostino (p_value)
1	0.229	0.239	0.957	0.247	0.229
2	0.231	0.241	0.957	0.194	0.211
3	0.223	0.241	0.925	0.795	0.832
4	0.236	0.241	0.977	0.290	0.550
5	0.238	0.240	0.995	0.218	0.184
6	0.220	0.236	0.928	0.824	0.754
7	0.232	0.237	0.983	0.994	0.976

Isotopic Ratio and Errors of the Blocks

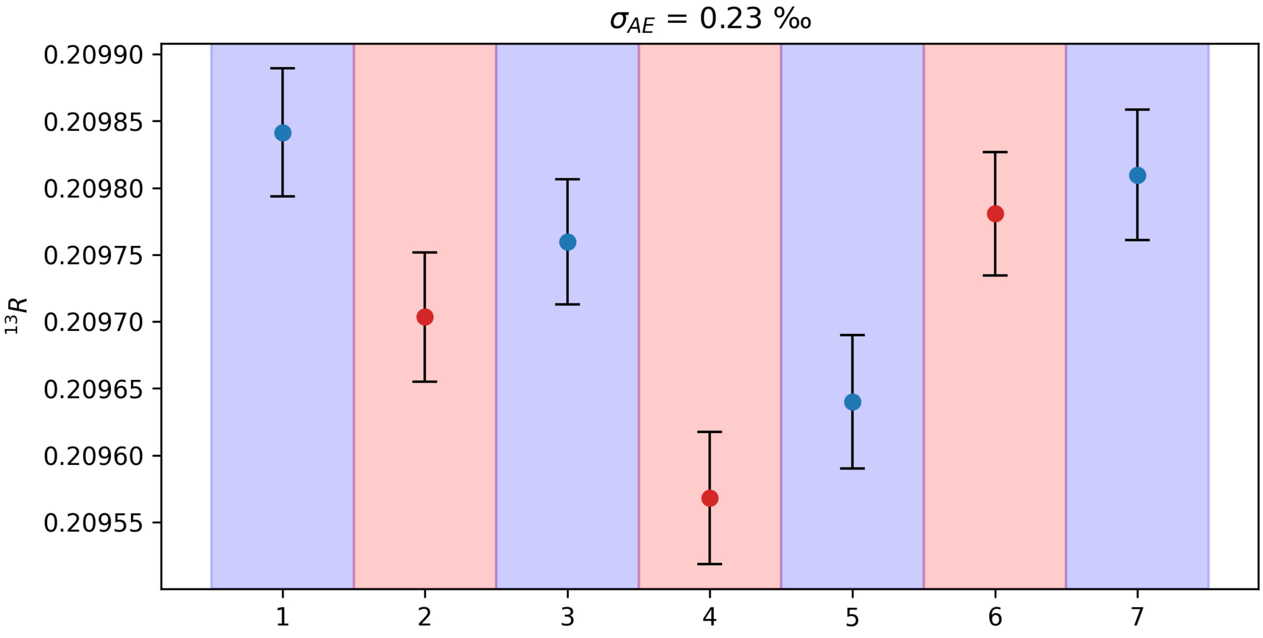

Cumulative Isotopic Ratio

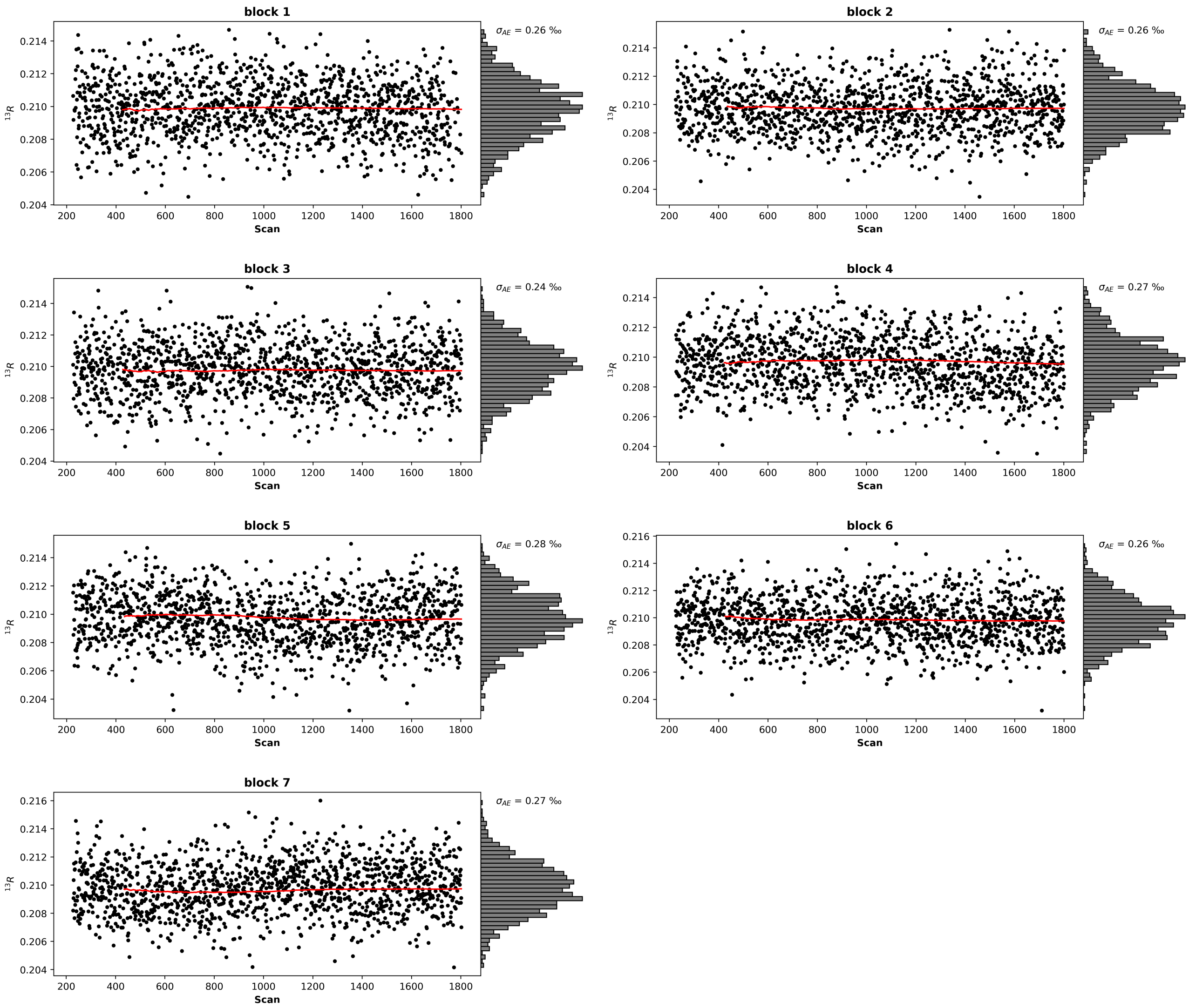

Acquisition Error and Shot-Noise

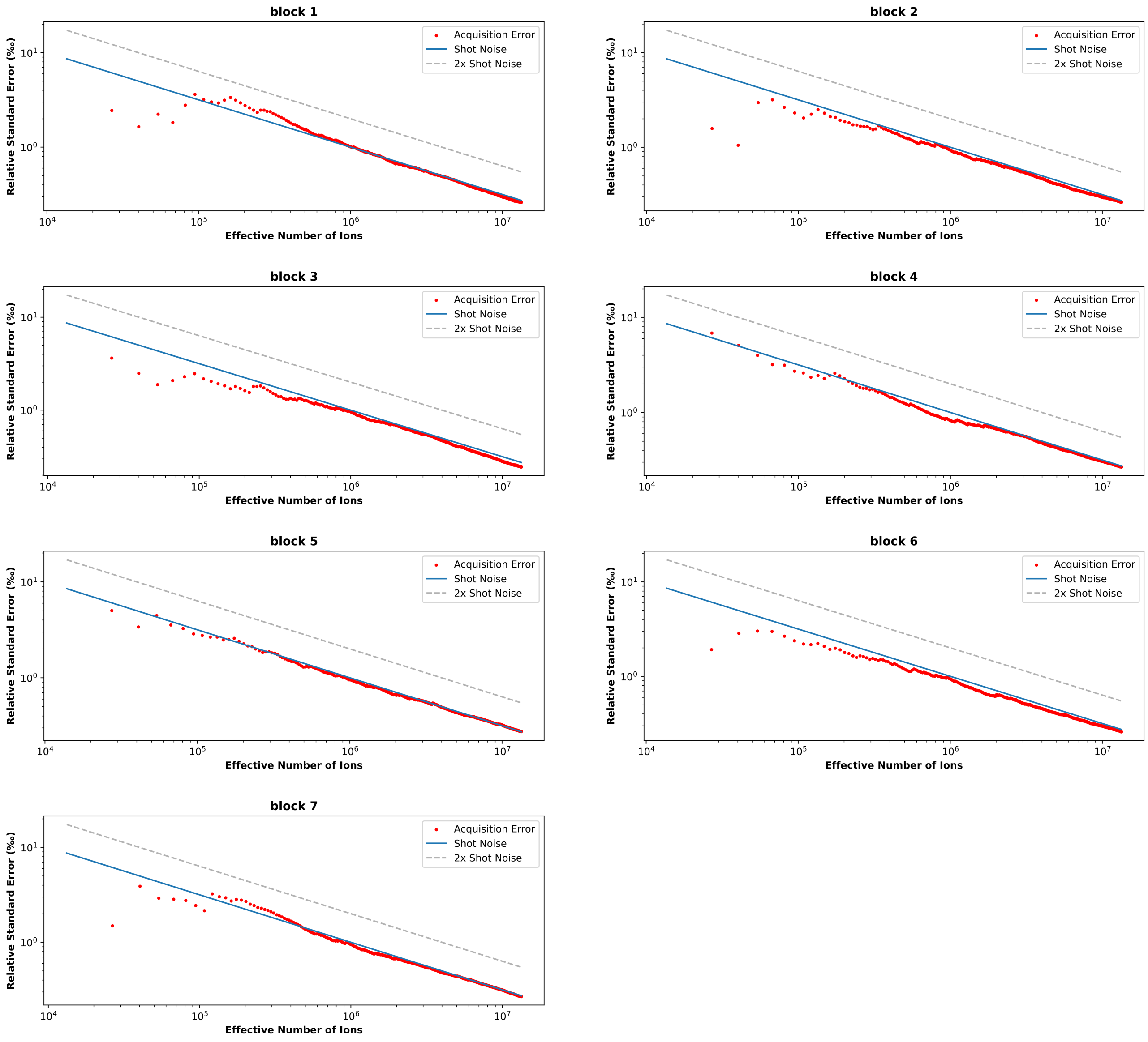

3. Delta Informations

Deltas were calculated by 'Average Of Neighboring Block Ratios'

3.1. 13C

Delta 13C was corrected by -27.80

Block	SEM	Delta corrected	Delta
2	0.23	-28.25	-0.46
4	0.24	-28.41	-0.63
6	0.22	-27.54	0.27

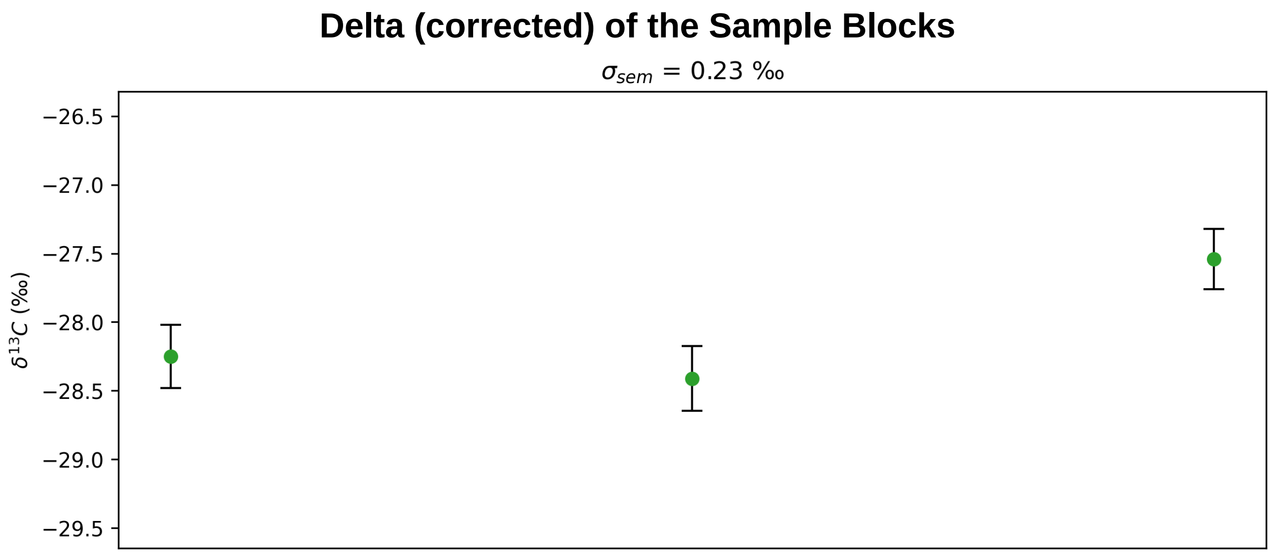

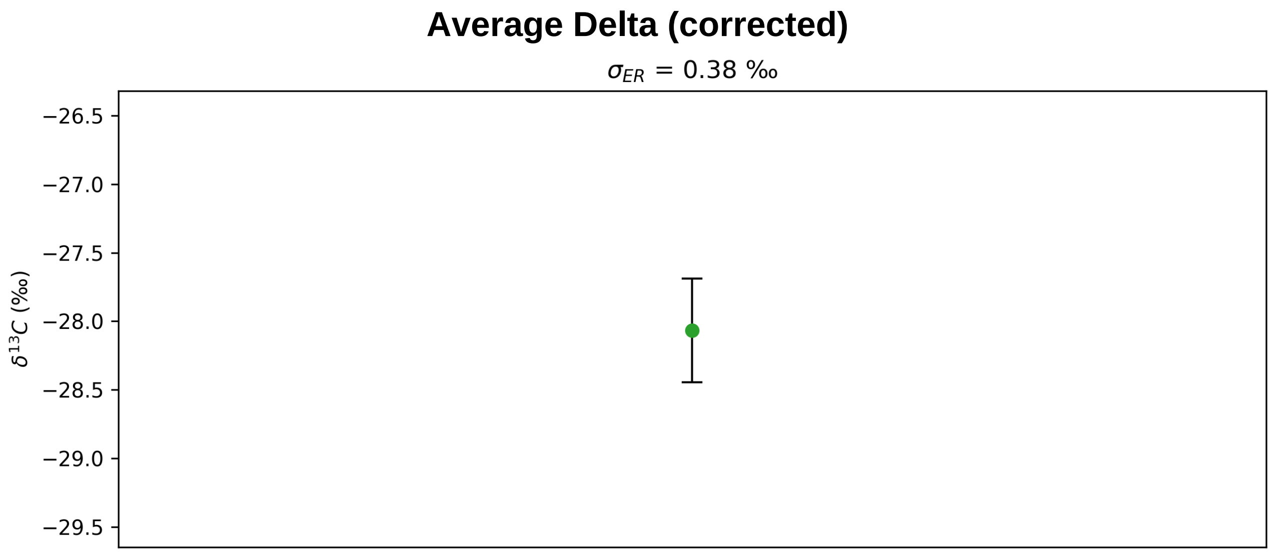

The final corrected average delta was -28.07 with a standard deviation of 0.38. Here the standard deviation is called reproducibility error.
