# Supplementary material for: Advancing Stable Isotope Analysis with Orbitrap-MS for Fatty Acid Methyl Esters and Complex Lipid Matrices
Source: J Am Soc Mass Spectrom. 2025 Jun 17;36(7):1527–35. doi: 10.1021/jasms.5c00092 (PMC12339014; doi:10.1021/jasms.5c00092)
Supplement: Supplementary file 2 [file js5c00092_si_002.zip › reports by IsotoPy Software/standards/H+Standard4_DI.pdf]

Standard 4 - [M + H]+

Isotope Analysis report from IsotoPy

Dual Inlet

1. Pre Processing

1.1. Block Time and Scan Information

Information about sample and standard block times and scans:

Block	Injected	Initial Time	End Time	Number of scans
1	standard	1	5	742
2	sample	6	10	709
3	standard	11	15	747
4	sample	16	20	729
5	standard	21	25	744
6	sample	26	30	708
7	standard	31	35	750

1.2. Outlier Removal

A total of 1174 scans were considered outliers and removed using the MAD method

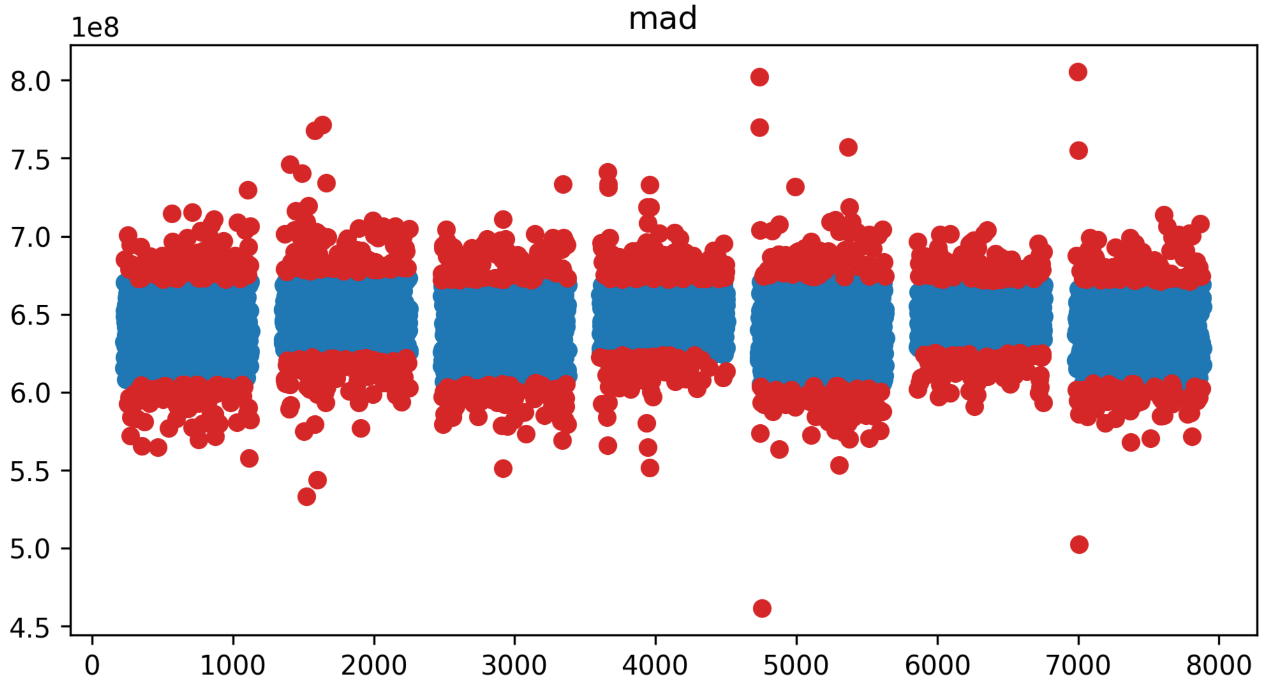

1.3. Total Ion Current (TIC)

TIC of all blocks

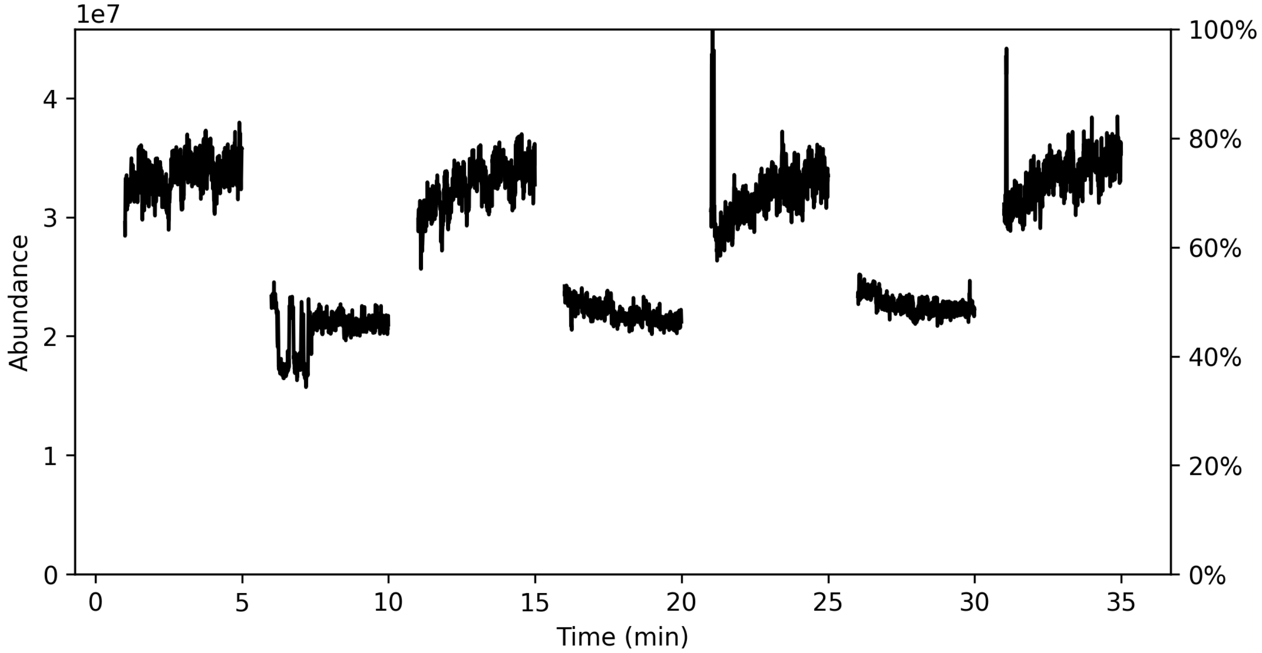

Block	TIC min	TIC max	TIC mean	RSD (%)
1	2.84e+07	3.80e+07	3.35e+07	4.47
2	1.57e+07	2.45e+07	2.06e+07	8.31
3	2.56e+07	3.70e+07	3.27e+07	5.53
4	2.02e+07	2.43e+07	2.19e+07	3.54
5	2.63e+07	4.58e+07	3.19e+07	7.73
6	2.09e+07	2.52e+07	2.26e+07	3.18
7	2.88e+07	4.42e+07	3.34e+07	5.77

2. Block Parameters

The Isotopic Ratio of the blocks were calculated by 'Mean'

2.1. 13C/M0

Block	Number of scans	Effective number of ions	Isotopic Ratio	STD	SEM	RSE
1	742	9.75e+06	0.196862	0.001635	0.000060	0.000305
2	709	9.24e+06	0.196284	0.001645	0.000062	0.000315
3	747	9.84e+06	0.196777	0.001658	0.000061	0.000308
4	729	9.53e+06	0.196388	0.001626	0.000060	0.000306
5	744	9.82e+06	0.196704	0.001672	0.000061	0.000311
6	708	9.26e+06	0.196347	0.001668	0.000063	0.000319
7	750	9.92e+06	0.196604	0.001667	0.000061	0.000309

Errors and Test Paramters

Block	Acquisition Error (permil)	Shot-Noise (permil)	AE/SN ratio	Shapiro Wilk (p_value)	D'Agostino (p_value)
1	0.305	0.320	0.952	0.839	0.384
2	0.315	0.329	0.956	0.176	0.408
3	0.308	0.319	0.966	0.266	0.227
4	0.306	0.324	0.946	0.833	0.764
5	0.311	0.319	0.976	0.631	0.400
6	0.319	0.329	0.971	0.899	0.879
7	0.309	0.317	0.975	0.435	0.896

Isotopic Ratio and Errors of the Blocks

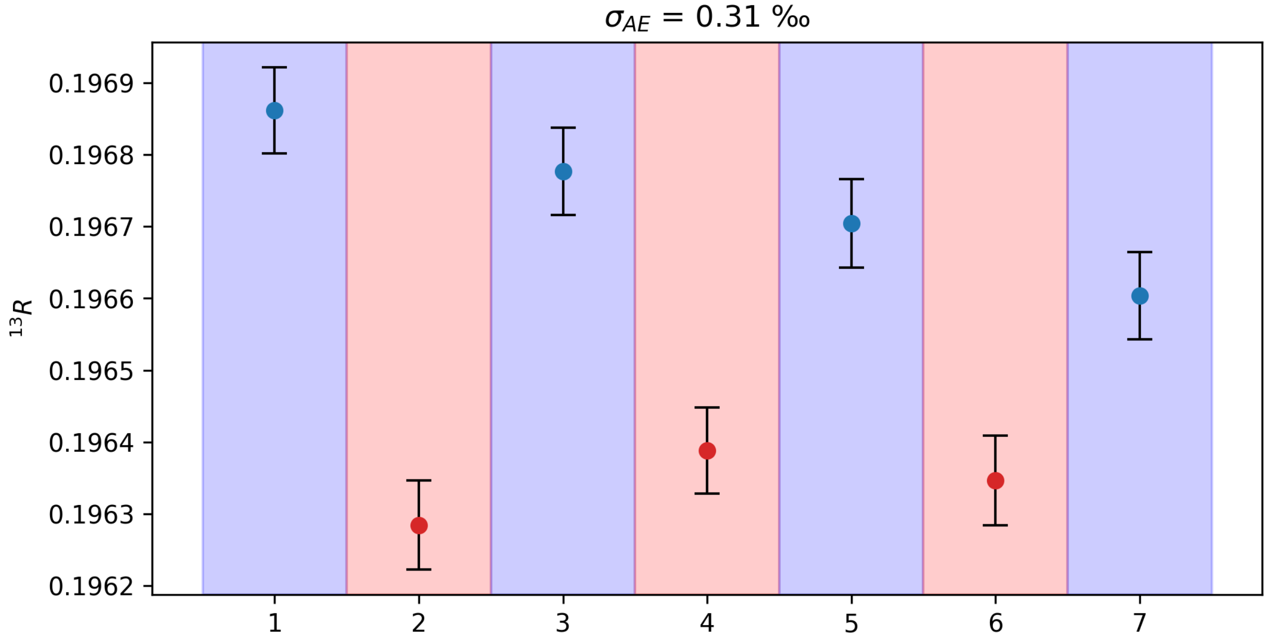

Cumulative Isotopic Ratio

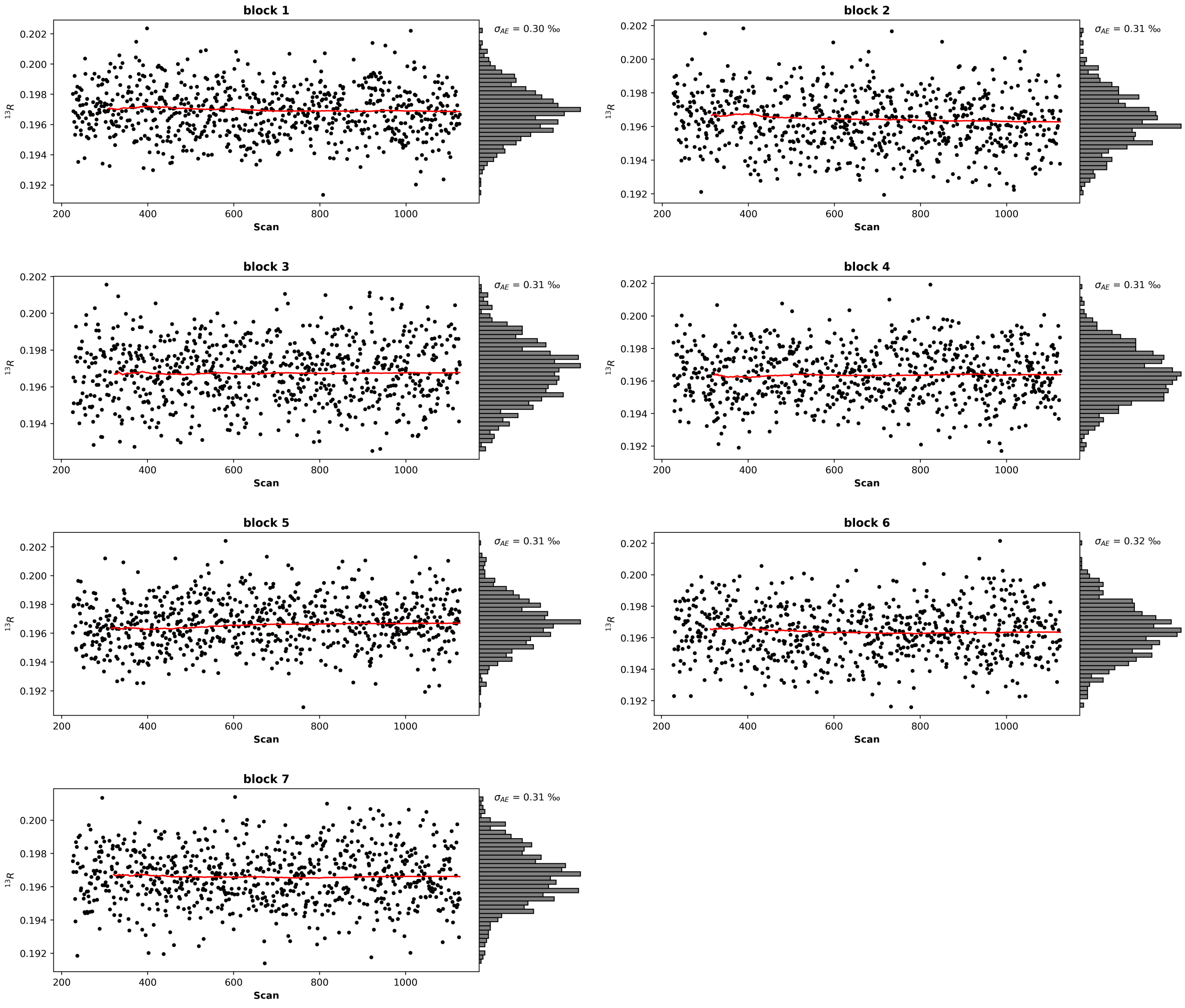

Acquisition Error and Shot-Noise

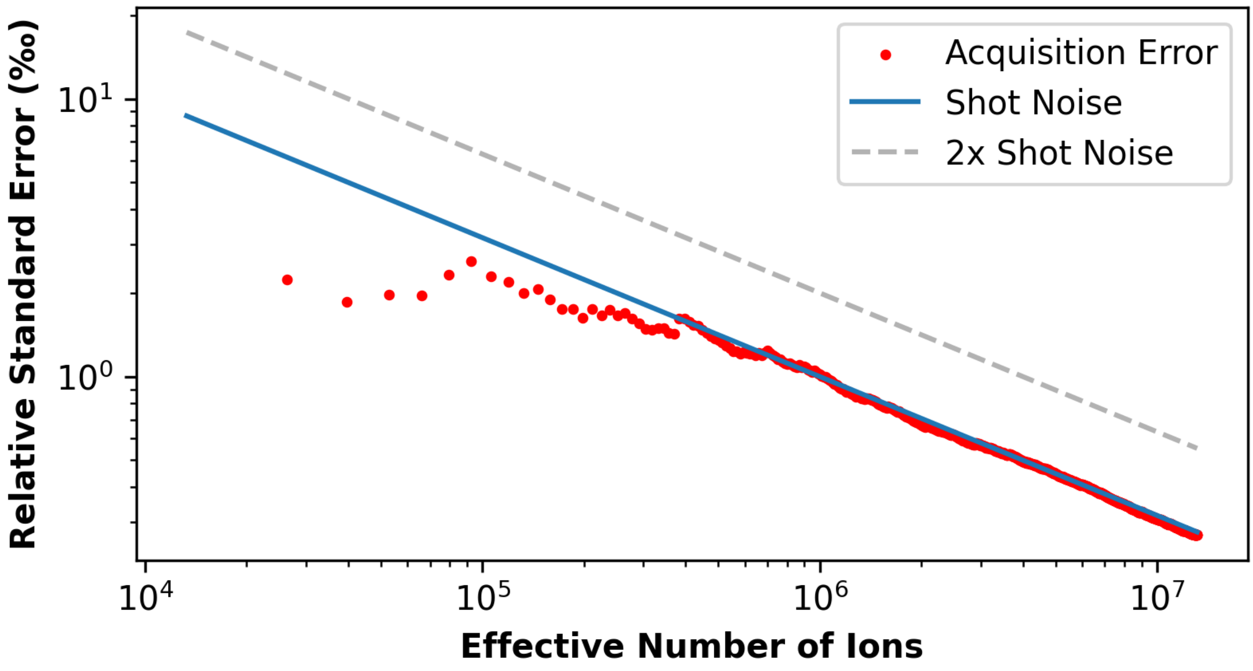

3. Delta Informations

Deltas were calculated by 'Average Of Neighboring Block Ratios'

3.1. 13C

Delta 13C was corrected by -27.80

Block	SEM	Delta corrected	Delta
2	0.31	-30.44	-2.72
4	0.31	-29.54	-1.79
6	0.32	-29.32	-1.56

Delta (corrected) of the Sample Blocks

σ_{sem} = 0.31 ‰

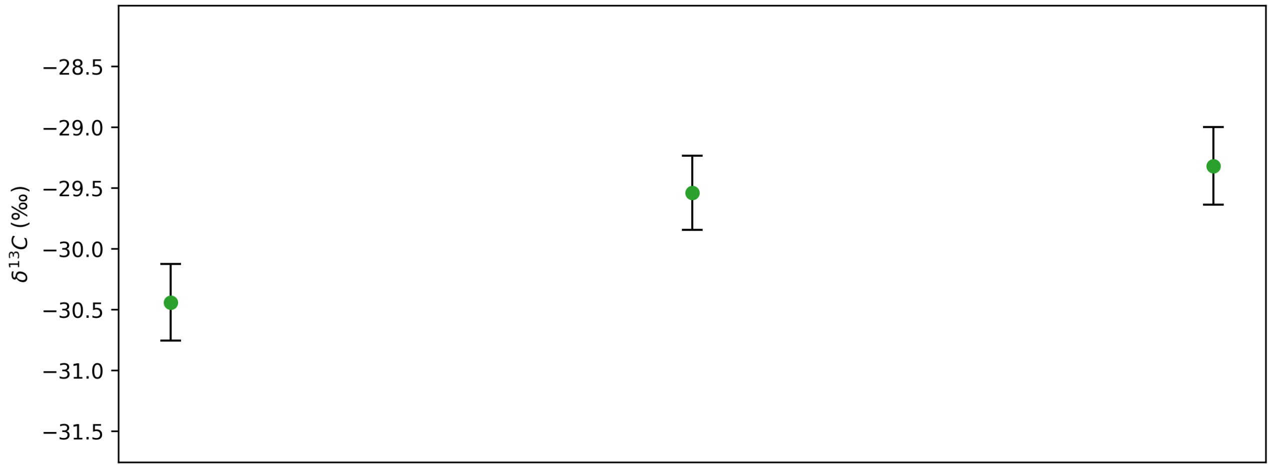

Average Delta (corrected)

σ_{ER} = 0.49 ‰

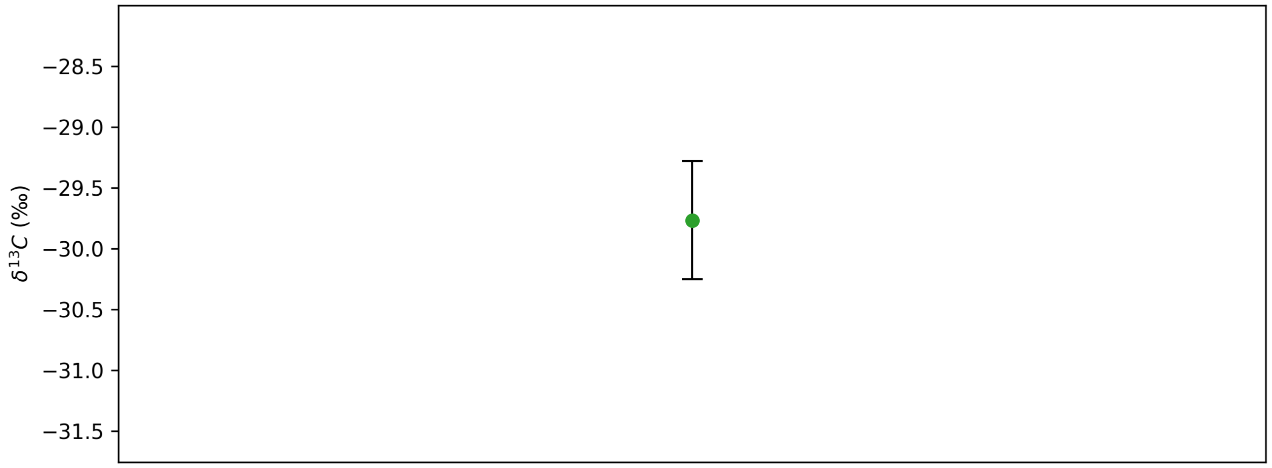

The final corrected average delta was -29.77 with a standard deviation of 0.49. Here the standard deviation is called reproducibility error.
