# Supplementary material for: Advancing Stable Isotope Analysis with Orbitrap-MS for Fatty Acid Methyl Esters and Complex Lipid Matrices
Source: J Am Soc Mass Spectrom. 2025 Jun 17;36(7):1527–35. doi: 10.1021/jasms.5c00092 (PMC12339014; doi:10.1021/jasms.5c00092)
Supplement: Supplementary file 2 [file js5c00092_si_002.zip › reports by IsotoPy Software/standards/H+Standard8_DI.pdf]

Standard 8 - [M + H]+
Isotope Analysis report from IsotoPy

Dual Inlet

1. Pre Processing

1.1. Block Time and Scan Information

Information about sample and standard block times and scans:

Block	Injected	Initial Time	End Time	Number of scans
1	standard	1	5	728
2	sample	6	10	751
3	standard	11	15	725
4	sample	16	20	739
5	standard	21	25	748
6	sample	26	30	700
7	standard	31	35	743

1.2. Outlier Removal

A total of 1168 scans were considered outliers and removed using the MAD method

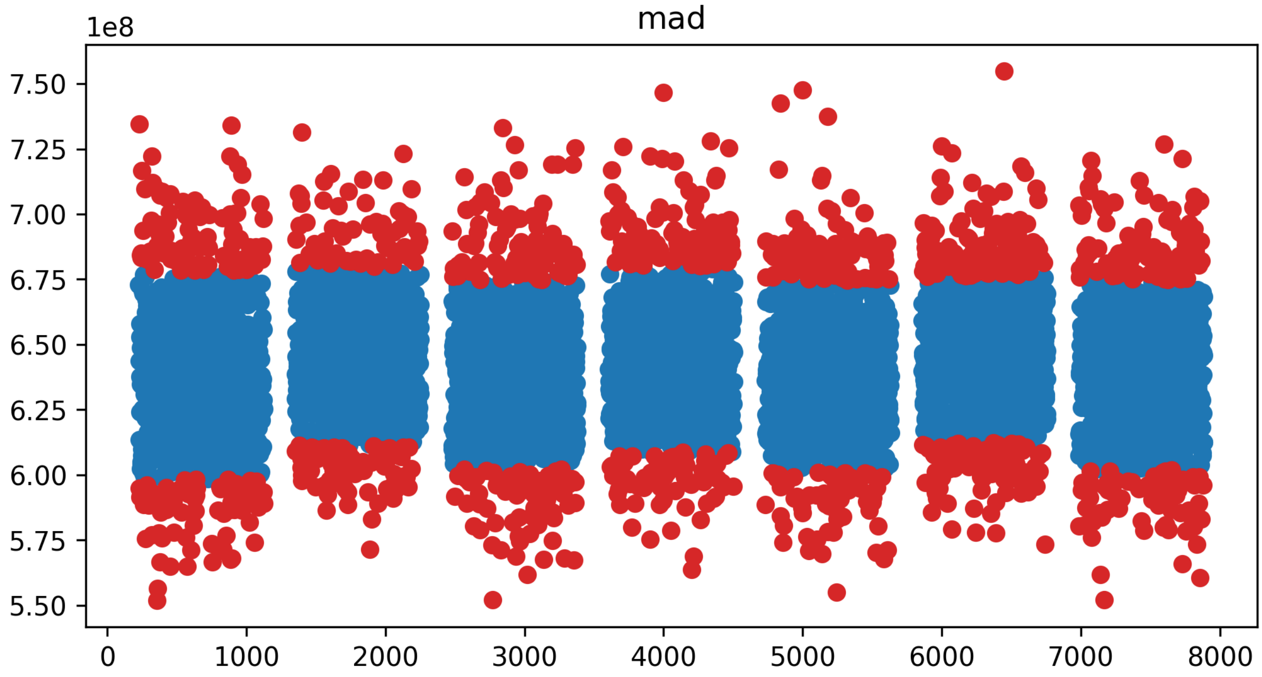

1.3. Total Ion Current (TIC)

TIC of all blocks

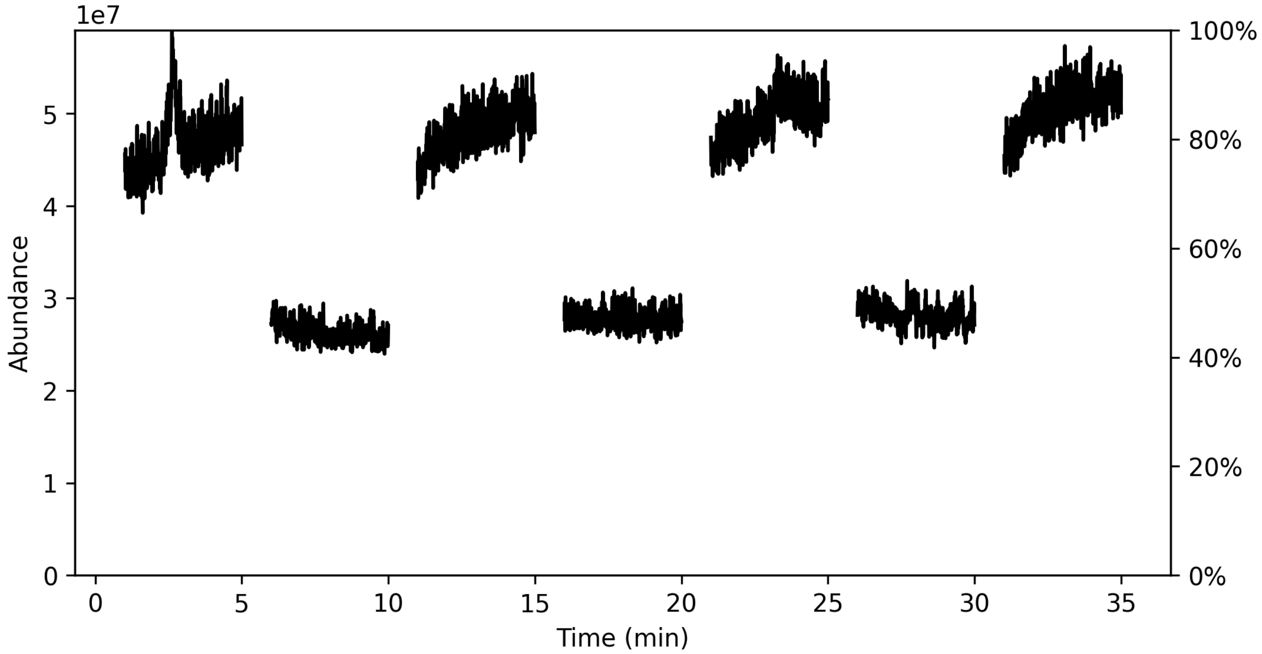

Block	TIC min	TIC max	TIC mean	RSD (%)
1	3.92e+07	5.90e+07	4.67e+07	6.51
2	2.40e+07	2.97e+07	2.63e+07	4.15
3	4.09e+07	5.43e+07	4.80e+07	5.11
4	2.52e+07	3.11e+07	2.78e+07	3.62
5	4.32e+07	5.63e+07	4.94e+07	5.20
6	2.47e+07	3.19e+07	2.82e+07	4.37
7	4.33e+07	5.73e+07	5.04e+07	5.16

2. Block Parameters

The Isotopic Ratio of the blocks were calculated by 'Mean'

2.1. 13C/M0

Block	Number of scans	Effective number of ions	Isotopic Ratio	STD	SEM	RSE
1	728	1.01e+07	0.197256	0.001747	0.000065	0.000328
2	751	1.03e+07	0.196760	0.001620	0.000059	0.000300
3	725	1.00e+07	0.197360	0.001702	0.000063	0.000320
4	739	1.02e+07	0.196924	0.001639	0.000060	0.000306
5	748	1.03e+07	0.197213	0.001592	0.000058	0.000295
6	700	9.62e+06	0.196904	0.001592	0.000060	0.000305
7	743	1.03e+07	0.197302	0.001618	0.000059	0.000301

Errors and Test Paramters

Block	Acquisition Error (permil)	Shot-Noise (permil)	AE/SN ratio	Shapiro Wilk (p_value)	D'Agostino (p_value)
1	0.328	0.315	1.042	0.272	0.214
2	0.300	0.311	0.965	0.858	0.918
3	0.320	0.315	1.014	0.106	0.703
4	0.306	0.313	0.976	0.367	0.328
5	0.295	0.311	0.948	0.418	0.800
6	0.305	0.322	0.947	0.239	0.750
7	0.301	0.312	0.963	0.963	0.797

Isotopic Ratio and Errors of the Blocks

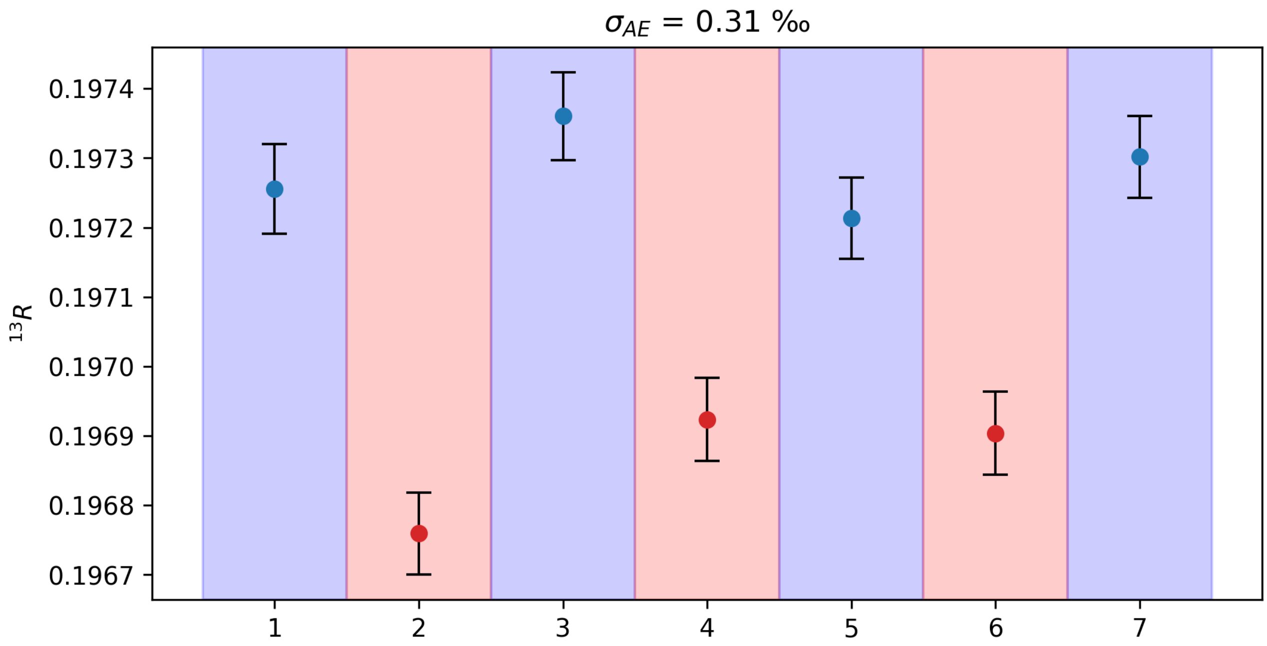

Cumulative Isotopic Ratio

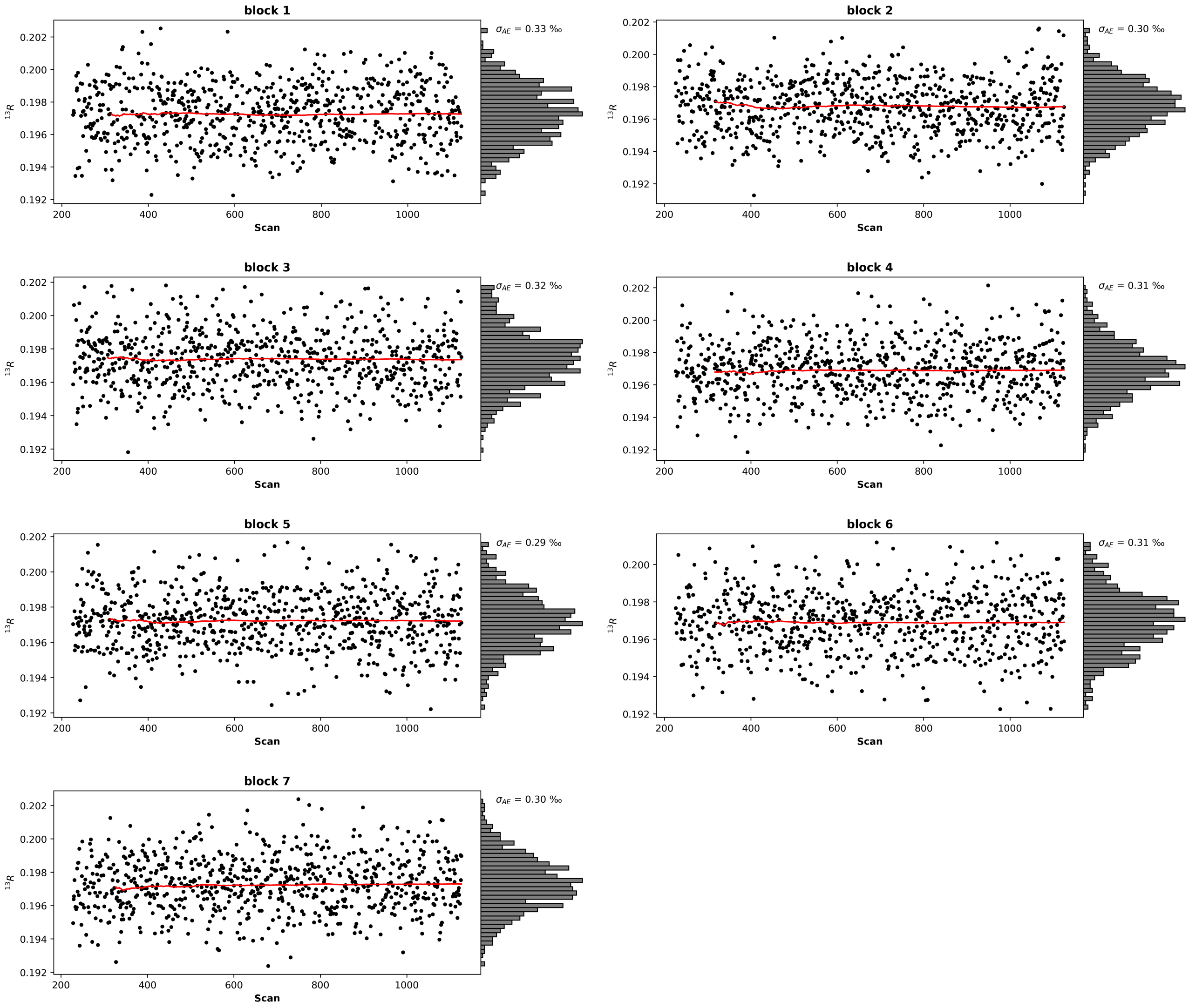

Acquisition Error and Shot-Noise

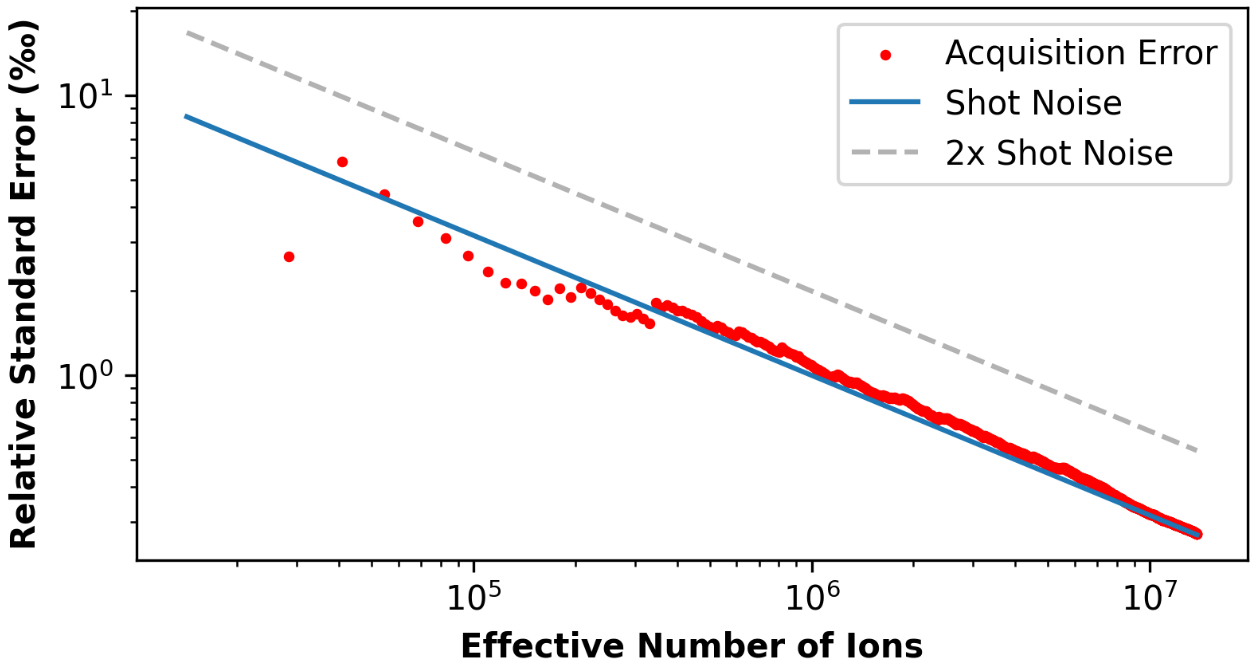

3. Delta Informations

Deltas were calculated by 'Average Of Neighboring Block Ratios'

3.1. 13C

Delta 13C was corrected by -27.80

Block	SEM	Delta corrected	Delta
2	0.30	-30.50	-2.78
4	0.31	-29.59	-1.84
6	0.30	-29.54	-1.79

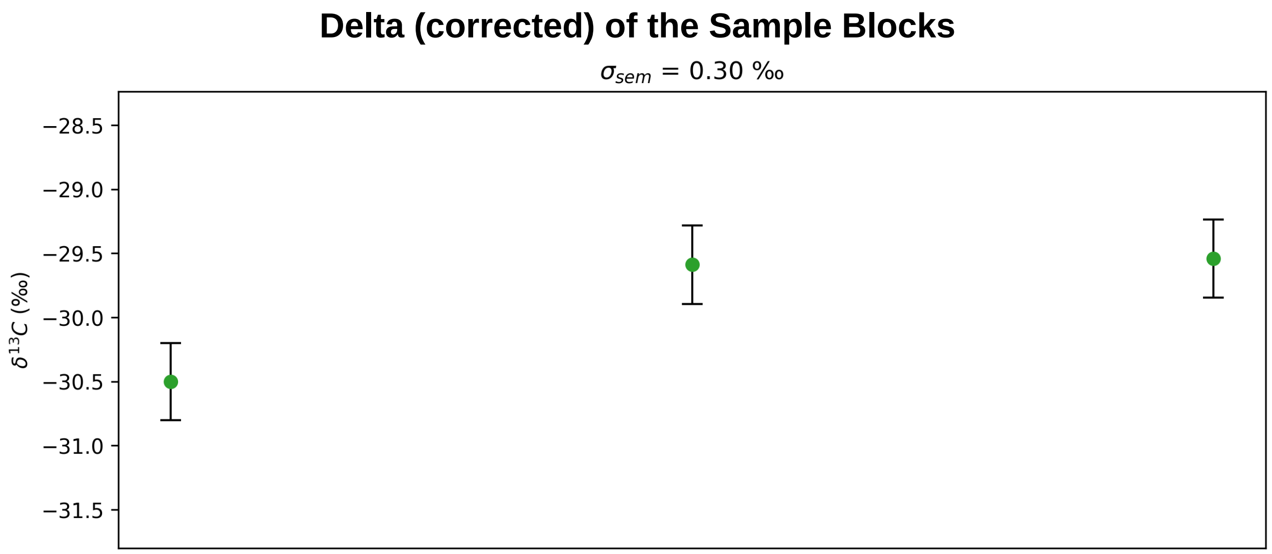

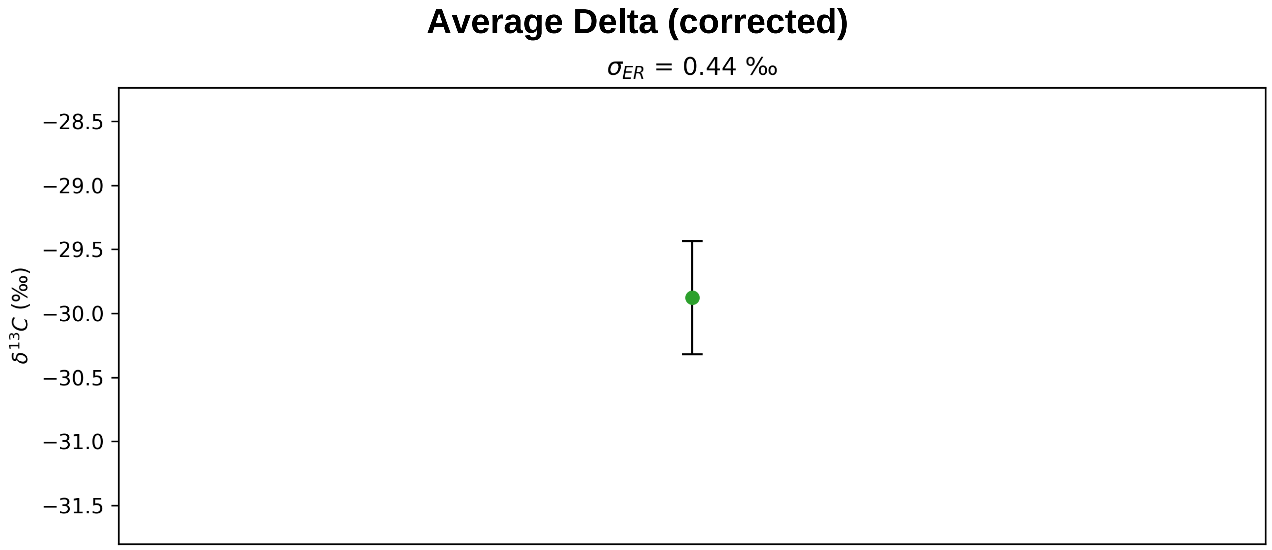

The final corrected average delta was -29.88 with a standard deviation of 0.44. Here the standard deviation is called reproducibility error.
