# Supplementary material for: Advancing Stable Isotope Analysis with Orbitrap-MS for Fatty Acid Methyl Esters and Complex Lipid Matrices
Source: J Am Soc Mass Spectrom. 2025 Jun 17;36(7):1527–35. doi: 10.1021/jasms.5c00092 (PMC12339014; doi:10.1021/jasms.5c00092)
Supplement: Supplementary file 2 [file js5c00092_si_002.zip › reports by IsotoPy Software/standards/Na+Standard6_FI.pdf]

Standard 6 - [M + Na]+
Isotope Analysis report from IsotoPy

Flow Injection

1. Pre Processing

1.1. Block Time and Scan Information

Information about sample and standard block times and scans:

Block	Injected	Initial Time	End Time	Number of scans
1	standard	1	8	1286
2	sample	16	23	1295
3	standard	31	38	1298
4	sample	46	53	1308
5	standard	61	68	1295
6	sample	76	83	1266
7	standard	91	98	1294

1.2. Outlier Removal

A total of 1997 scans were considered outliers and removed using the MAD method

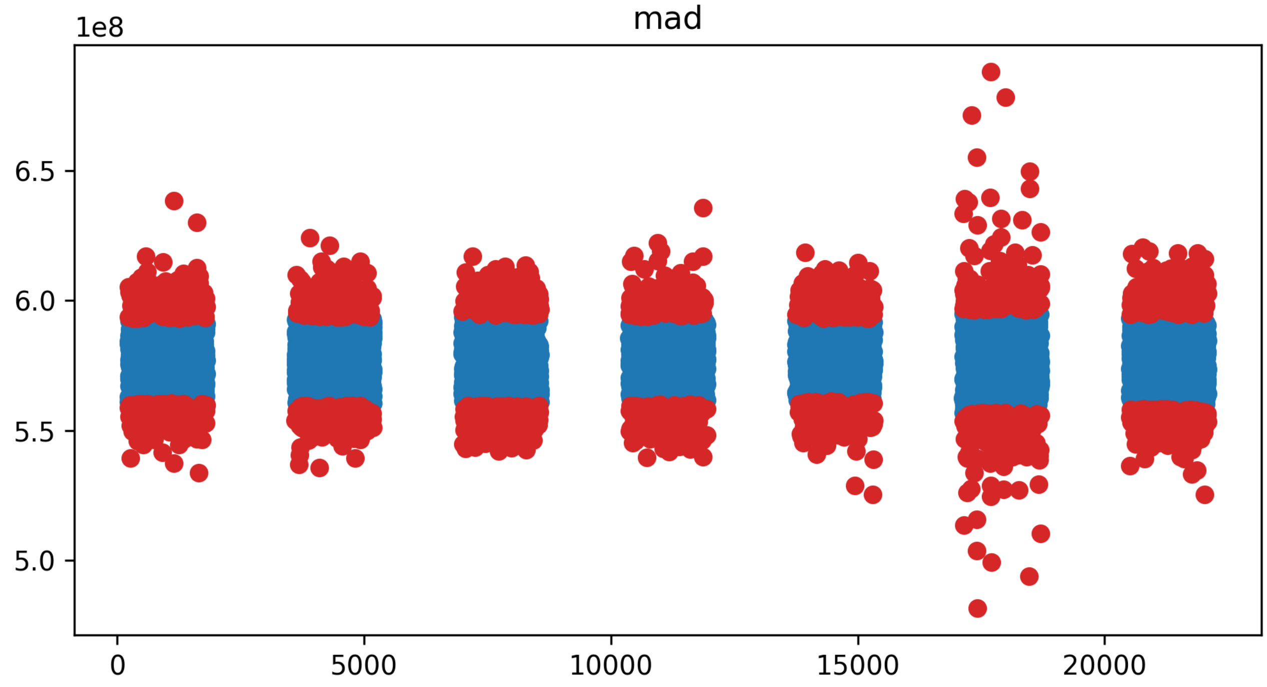

1.3. Total Ion Current (TIC)

TIC of all blocks

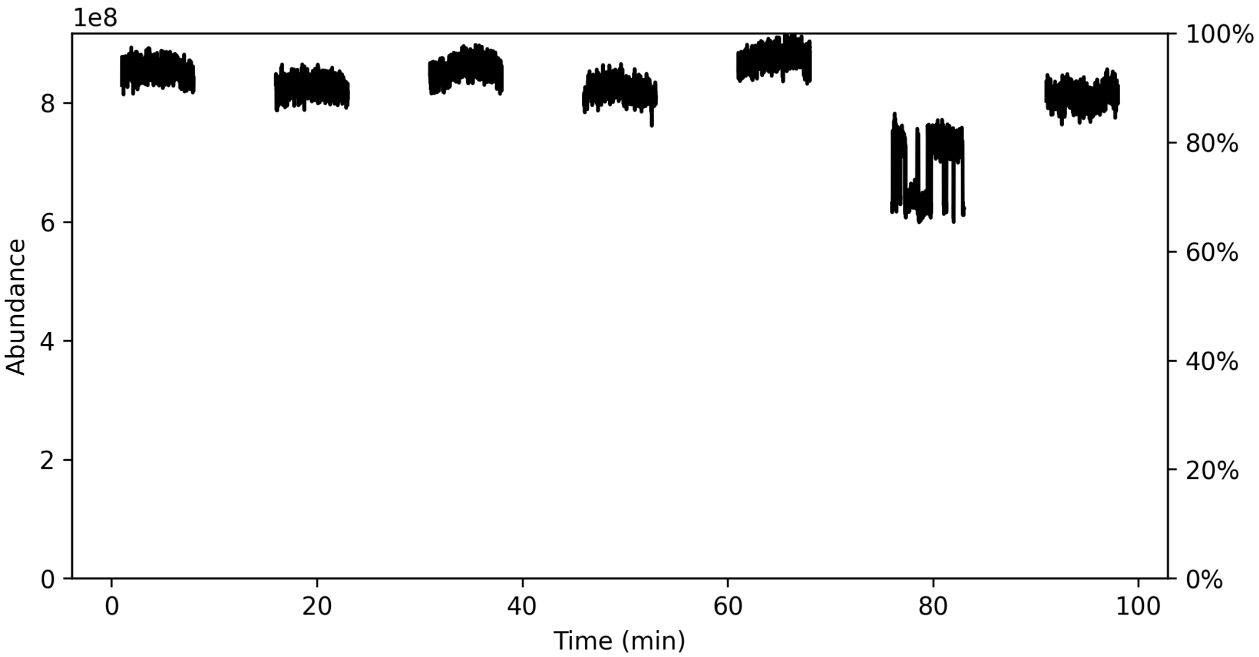

Block	TIC min	TIC max	TIC mean	RSD (%)
1	8.14e+08	8.93e+08	8.55e+08	1.53
2	7.87e+08	8.65e+08	8.27e+08	1.52
3	8.15e+08	8.98e+08	8.57e+08	1.70
4	7.61e+08	8.65e+08	8.21e+08	1.66
5	8.32e+08	9.17e+08	8.77e+08	1.65
6	5.99e+08	7.82e+08	6.92e+08	7.74
7	7.63e+08	8.57e+08	8.10e+08	1.80

2. Block Parameters

The Isotopic Ratio of the blocks were calculated by 'Mean'

2.1. 13C/M0

Block	Number of scans	Effective number of ions	Isotopic Ratio	STD	SEM	RSE
1	1286	2.07e+07	0.208876	0.001781	0.000050	0.000238
2	1295	2.08e+07	0.208635	0.001789	0.000050	0.000238
3	1298	2.09e+07	0.208913	0.001730	0.000048	0.000230
4	1308	2.10e+07	0.208728	0.001741	0.000048	0.000231
5	1295	2.08e+07	0.208837	0.001777	0.000049	0.000236
6	1266	2.03e+07	0.208753	0.001760	0.000049	0.000237
7	1294	2.08e+07	0.208998	0.001790	0.000050	0.000238

Errors and Test Paramters

Block	Acquisition Error (permil)	Shot-Noise (permil)	AE/SN ratio	Shapiro Wilk (p_value)	D'Agostino (p_value)
1	0.238	0.220	1.082	0.134	0.280
2	0.238	0.219	1.086	0.352	0.782
3	0.230	0.219	1.050	0.682	0.993
4	0.231	0.218	1.057	0.679	0.493
5	0.236	0.219	1.078	0.460	0.155
6	0.237	0.222	1.067	0.320	0.151
7	0.238	0.219	1.085	0.267	0.750

Isotopic Ratio and Errors of the Blocks

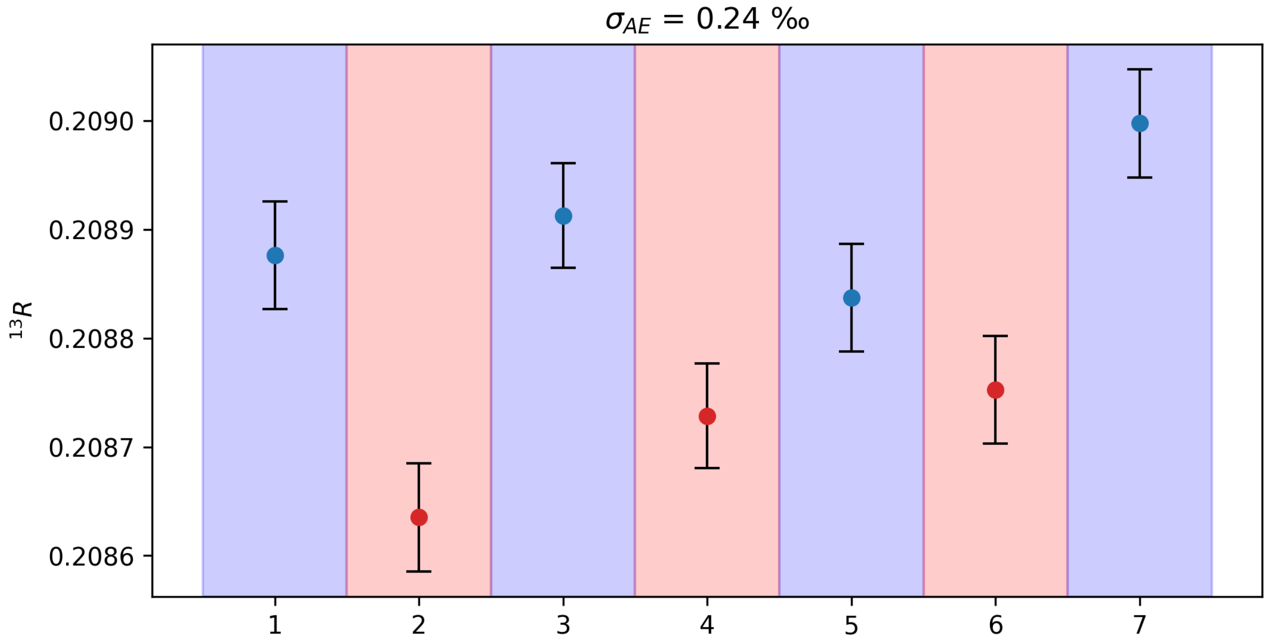

Cumulative Isotopic Ratio

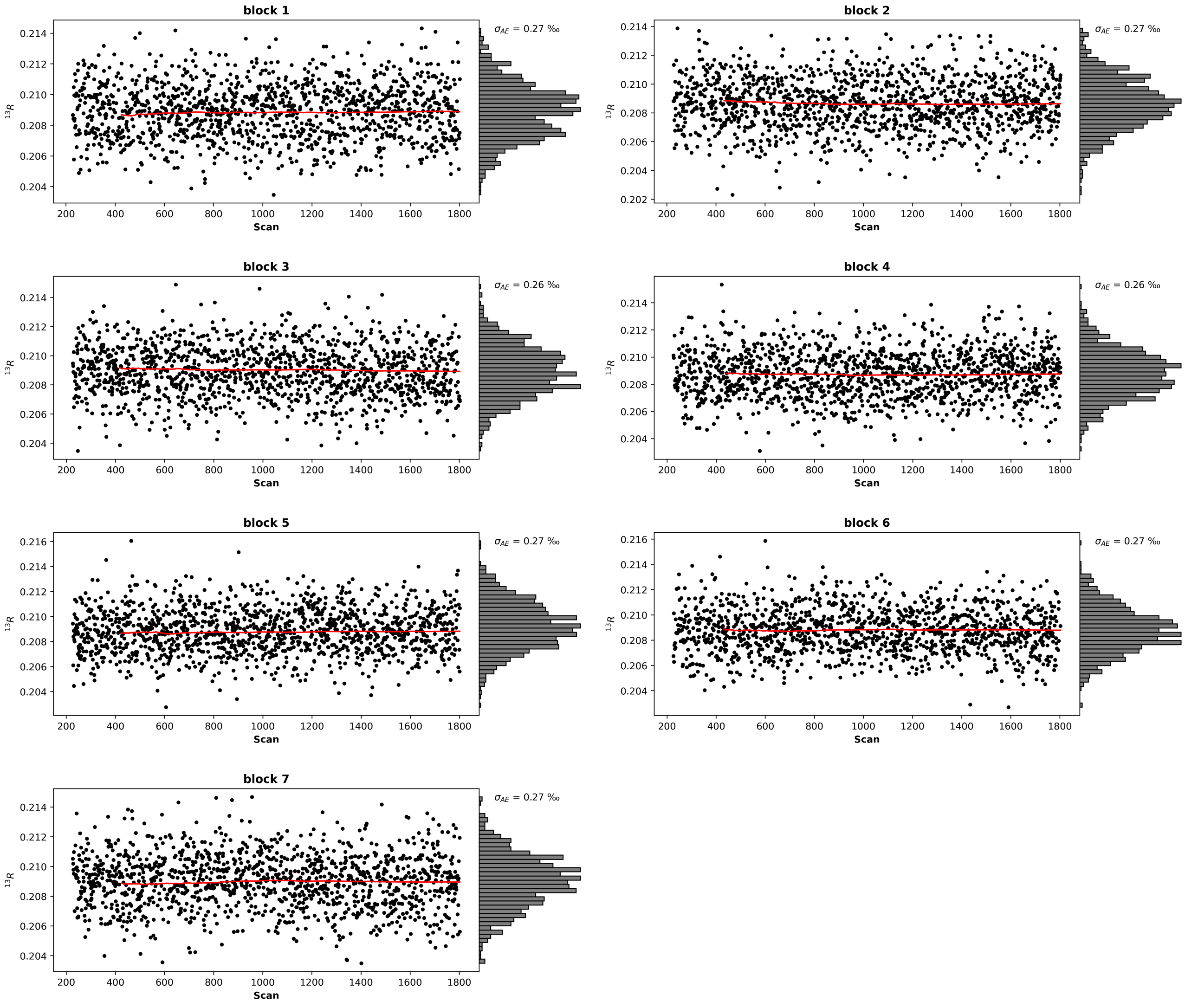

Acquisition Error and Shot-Noise

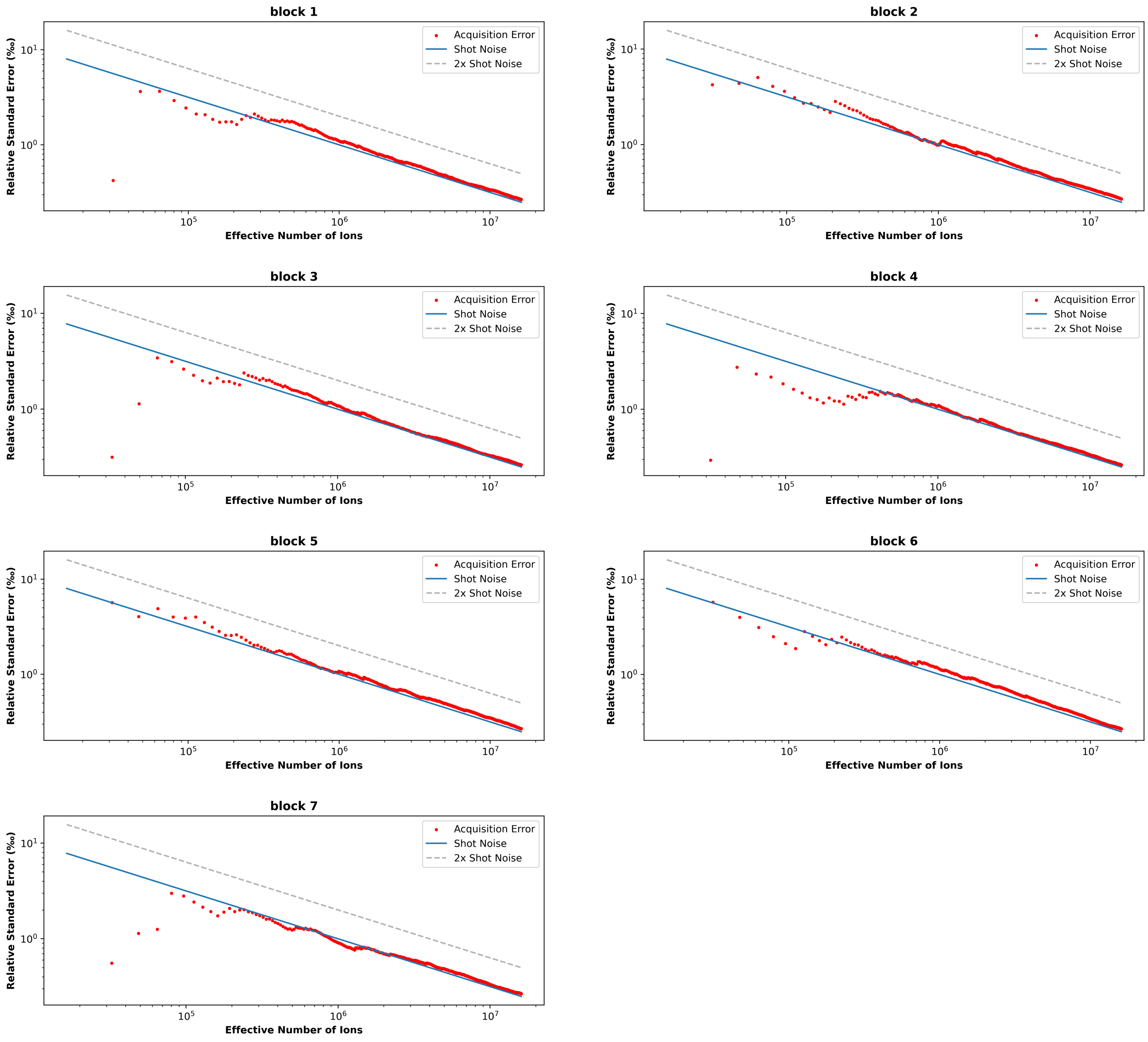

3. Delta Informations

Deltas were calculated by 'Average Of Neighboring Block Ratios'

3.1. 13C

Delta 13C was corrected by -27.80

Block	SEM	Delta corrected	Delta
2	0.24	-29.01	-1.24
4	0.23	-28.48	-0.70
6	0.24	-28.57	-0.79

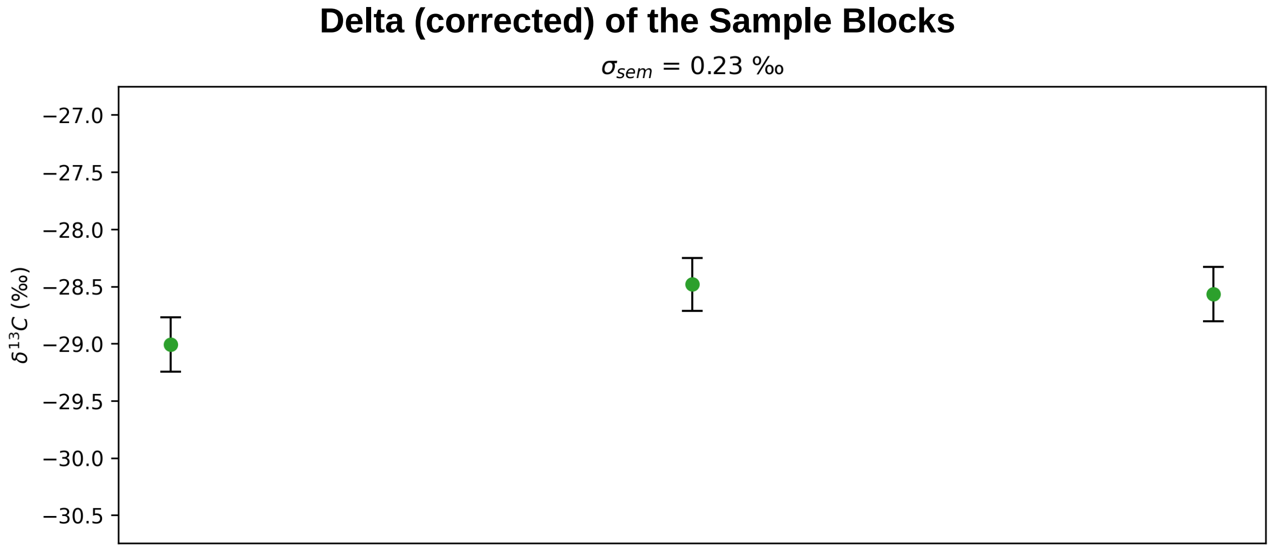

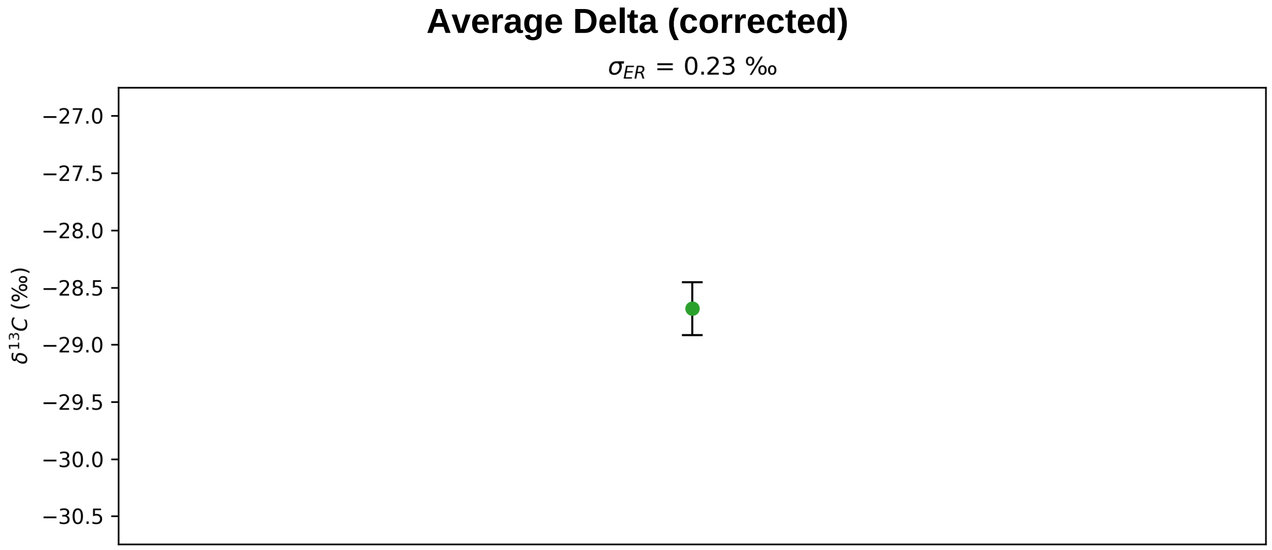

The final corrected average delta was -28.69 with a standard deviation of 0.23. Here the standard deviation is called reproducibility error.
