# Supplementary material for: Advancing Stable Isotope Analysis with Orbitrap-MS for Fatty Acid Methyl Esters and Complex Lipid Matrices
Source: J Am Soc Mass Spectrom. 2025 Jun 17;36(7):1527–35. doi: 10.1021/jasms.5c00092 (PMC12339014; doi:10.1021/jasms.5c00092)
Supplement: Supplementary file 2 [file js5c00092_si_002.zip › reports by IsotoPy Software/standards/Na+Standard1_DI.pdf]

Standard 1 - [M + Na]+
Isotope Analysis report from IsotoPy

Dual Inlet

1. Pre Processing

1.1. Block Time and Scan Information

Information about sample and standard block times and scans:

Block	Injected	Initial Time	End Time	Number of scans
1	standard	1	5	709
2	sample	6	10	744
3	standard	11	15	747
4	sample	16	20	733
5	standard	21	25	735
6	sample	26	30	738
7	standard	31	35	711

1.2. Outlier Removal

A total of 1186 scans were considered outliers and removed using the MAD method

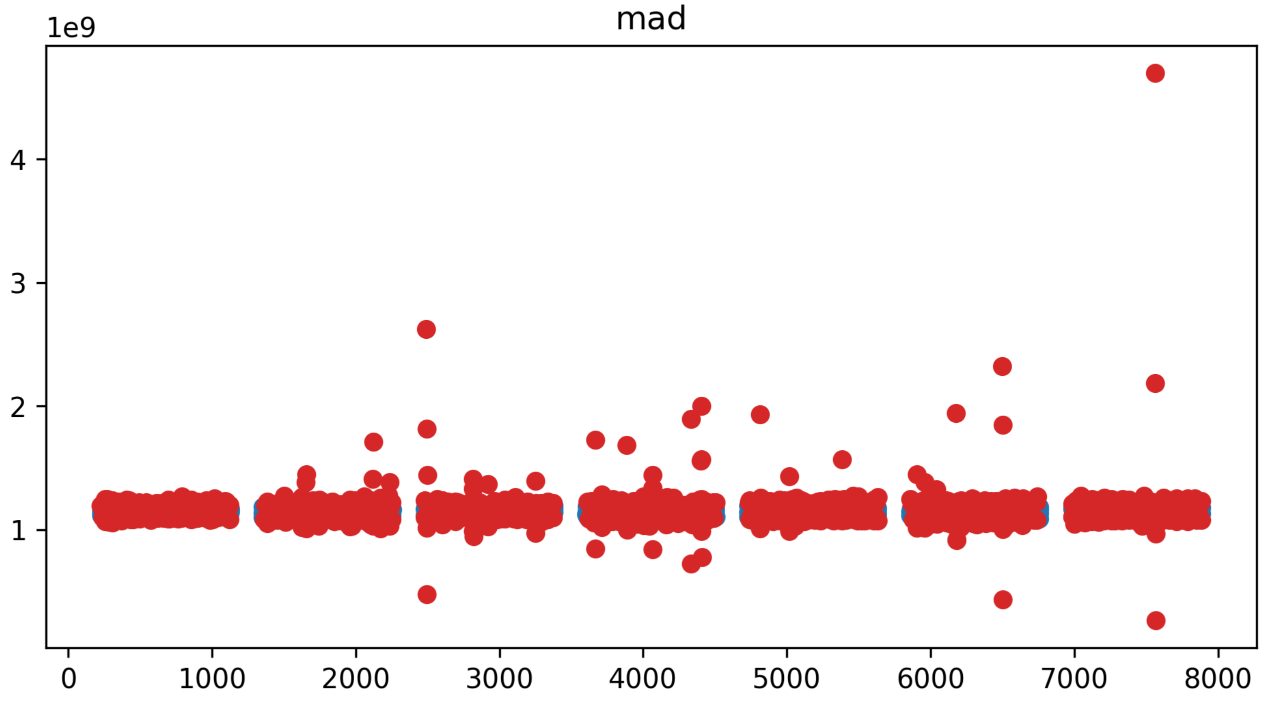

1.3. Total Ion Current (TIC)

TIC of all blocks

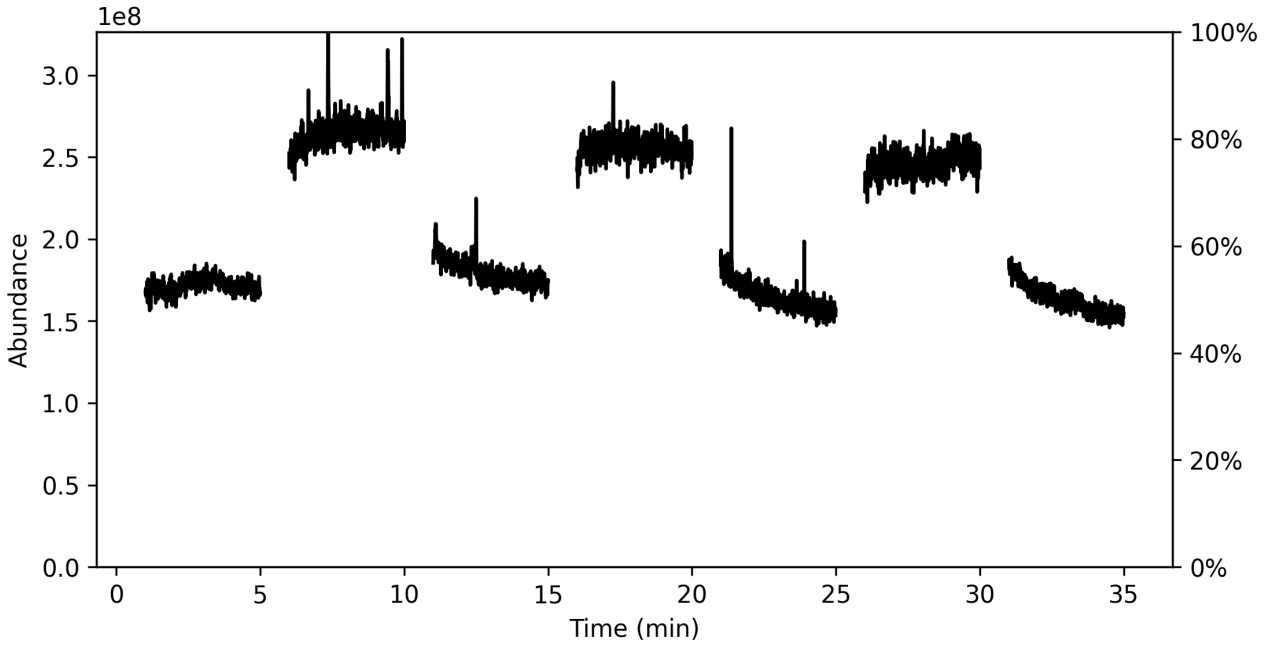

Block	TIC min	TIC max	TIC mean	RSD (%)
1	1.56e+08	1.85e+08	1.72e+08	2.62
2	2.36e+08	3.26e+08	2.65e+08	3.50
3	1.63e+08	2.25e+08	1.79e+08	4.01
4	2.32e+08	2.96e+08	2.56e+08	2.50
5	1.47e+08	2.68e+08	1.65e+08	5.56
6	2.22e+08	2.66e+08	2.47e+08	2.79
7	1.46e+08	1.89e+08	1.63e+08	5.46

2. Block Parameters

The Isotopic Ratio of the blocks were calculated by 'Mean'

2.1. 13C/M0

Block	Number of scans	Effective number of ions	Isotopic Ratio	STD	SEM	RSE
1	709	1.56e+07	0.210448	0.001337	0.000050	0.000238
2	744	1.63e+07	0.210072	0.001329	0.000049	0.000232
3	747	1.64e+07	0.210417	0.001381	0.000051	0.000240
4	733	1.60e+07	0.210046	0.001329	0.000049	0.000234
5	735	1.61e+07	0.210420	0.001371	0.000051	0.000240
6	738	1.61e+07	0.210085	0.001348	0.000050	0.000236
7	711	1.56e+07	0.210575	0.001330	0.000050	0.000237

Errors and Test Paramters

Block	Acquisition Error (permil)	Shot-Noise (permil)	AE/SN ratio	Shapiro Wilk (p_value)	D'Agostino (p_value)
1	0.238	0.254	0.941	0.284	0.145
2	0.232	0.248	0.935	0.648	0.571
3	0.240	0.247	0.971	0.534	0.313
4	0.234	0.250	0.935	0.227	0.131
5	0.240	0.249	0.964	0.841	0.664
6	0.236	0.249	0.948	0.258	0.695
7	0.237	0.253	0.935	0.466	0.194

Isotopic Ratio and Errors of the Blocks

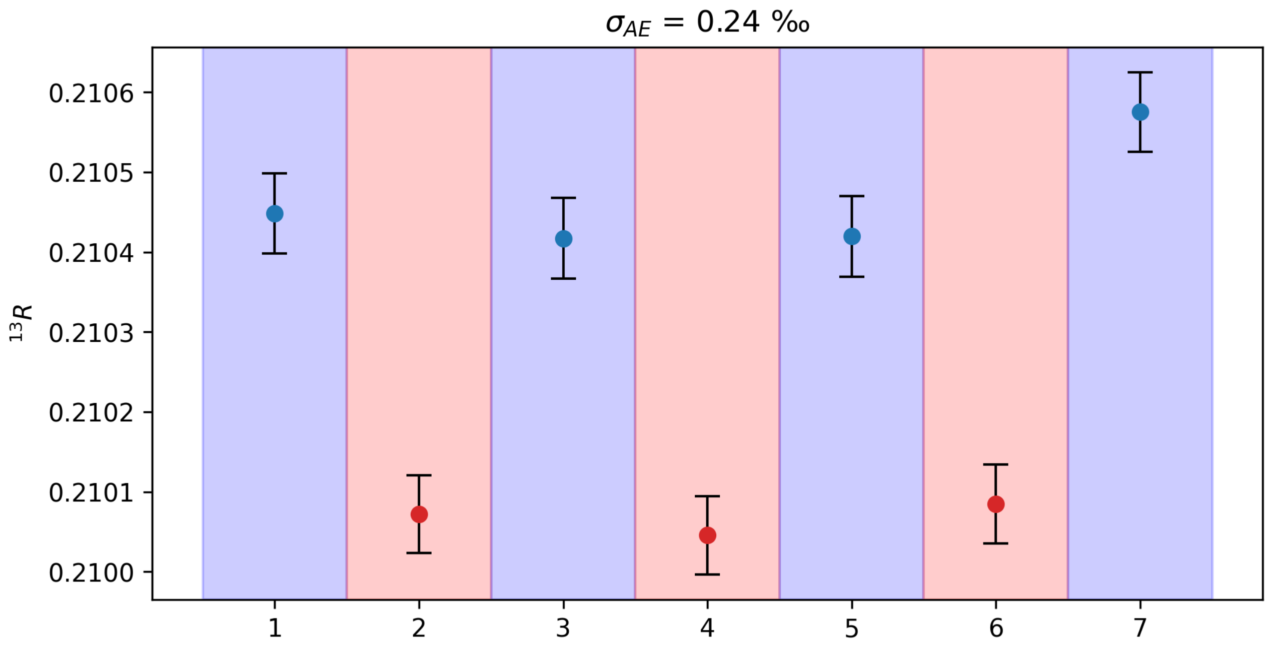

Cumulative Isotopic Ratio

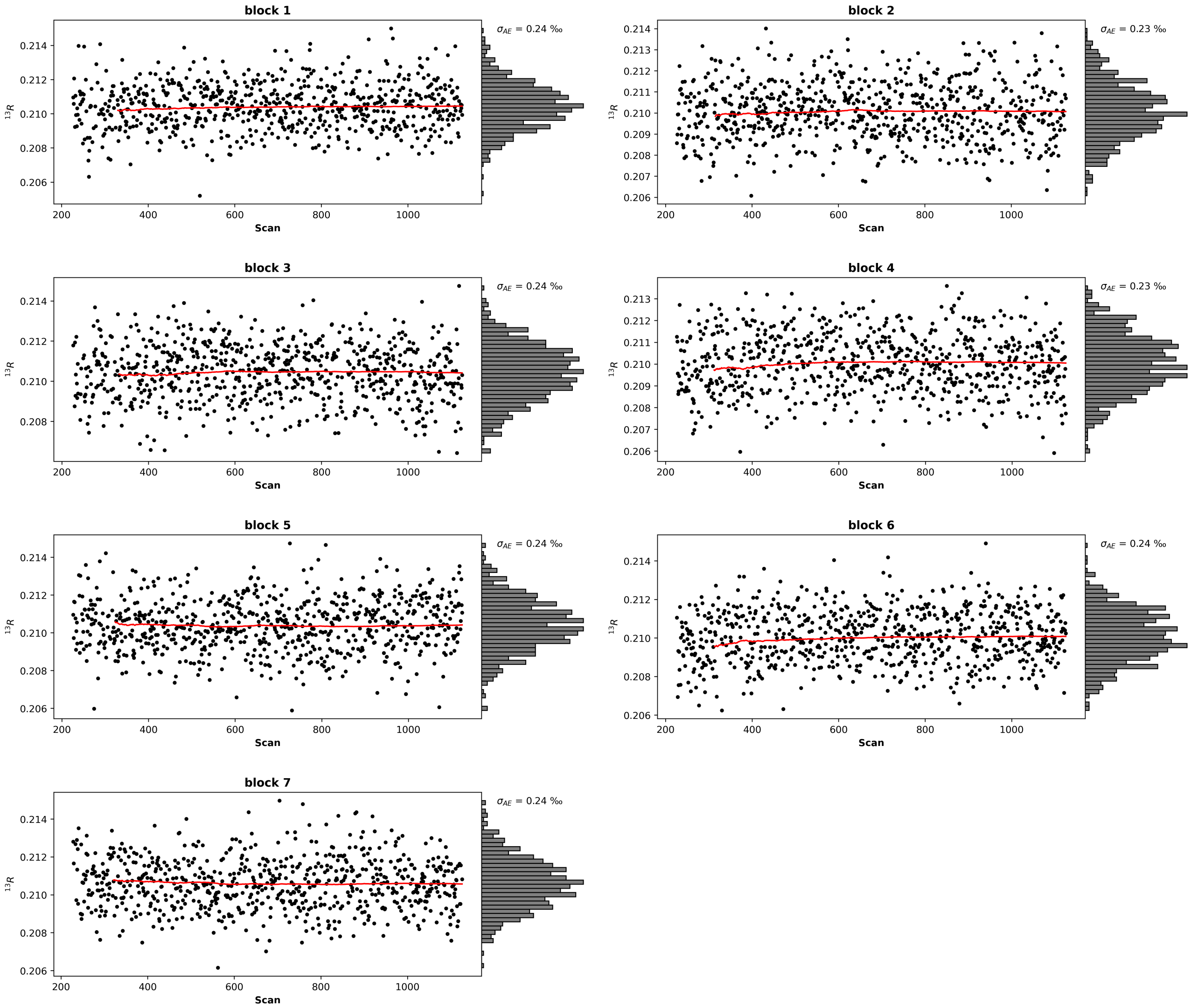

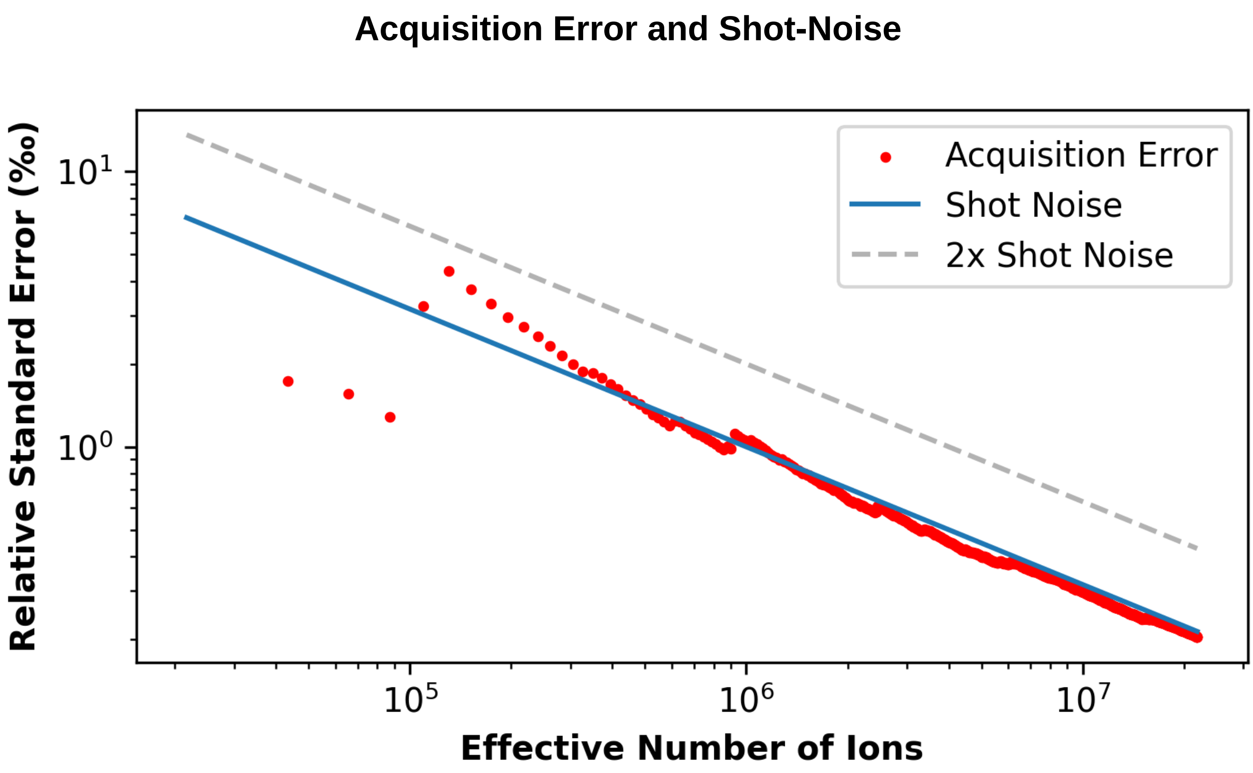

Acquisition Error and Shot-Noise
Relative Standard Error (‰)
Effective Number of Ions
Acquisition Error
Shot Noise
2x Shot Noise
10^1
10^0
10^5
10^6
10^7

3. Delta Informations

Deltas were calculated by 'Average Of Neighboring Block Ratios'

3.1. 13C

Delta 13C was corrected by -27.80

Block	SEM	Delta corrected	Delta
2	0.23	-29.47	-1.71
4	0.23	-29.52	-1.77
6	0.24	-29.71	-1.96

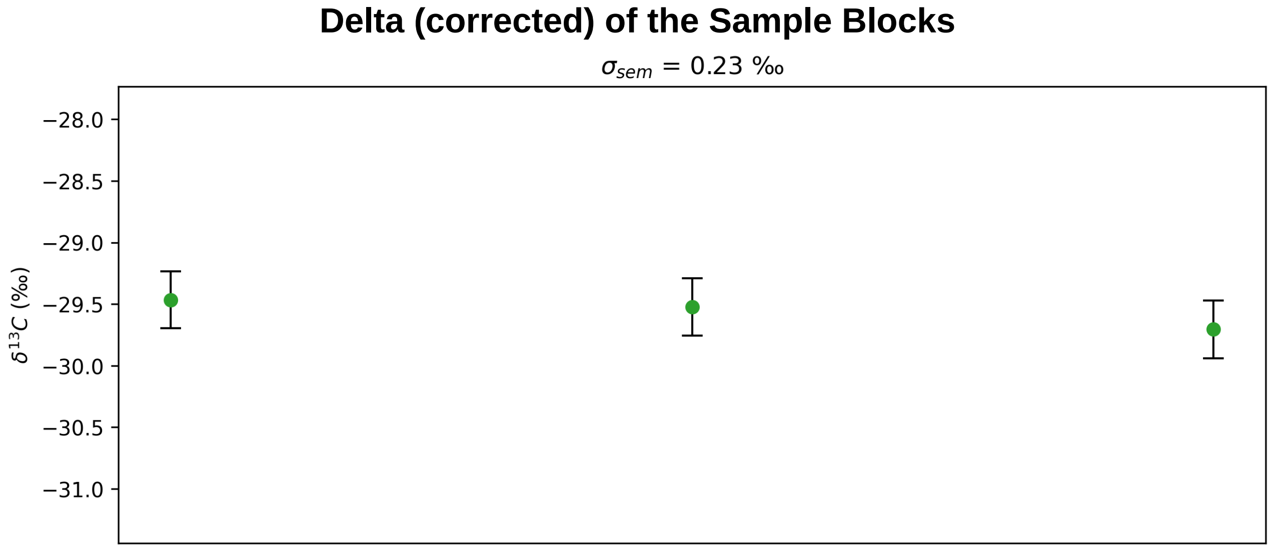

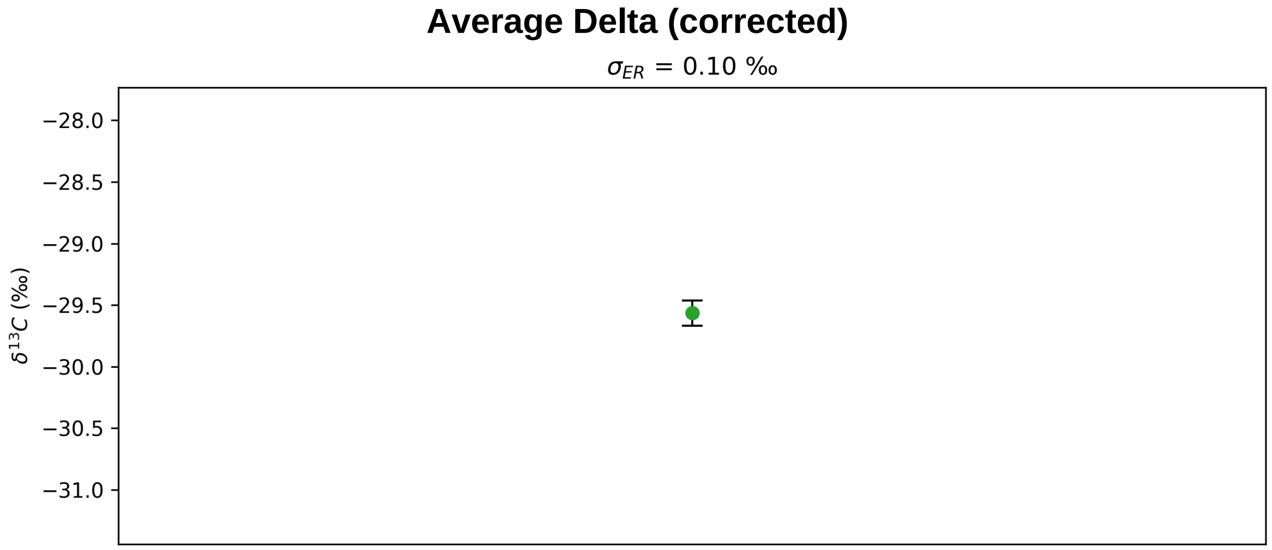

The final corrected average delta was -29.56 with a standard deviation of 0.10. Here the standard deviation is called reproducibility error.
