# Supplementary material for: Advancing Stable Isotope Analysis with Orbitrap-MS for Fatty Acid Methyl Esters and Complex Lipid Matrices
Source: J Am Soc Mass Spectrom. 2025 Jun 17;36(7):1527–35. doi: 10.1021/jasms.5c00092 (PMC12339014; doi:10.1021/jasms.5c00092)
Supplement: Supplementary file 2 [file js5c00092_si_002.zip › reports by IsotoPy Software/standards/H+Standard7_FI.pdf]

Standard 7 - [M + H]+
Isotope Analysis report from IsotoPy

Flow Injection

1. Pre Processing

1.1. Block Time and Scan Information

Information about sample and standard block times and scans:

Block	Injected	Initial Time	End Time	Number of scans
1	standard	1	8	1258
2	sample	16	23	1280
3	standard	31	38	1326
4	sample	46	53	1300
5	standard	61	68	1324
6	sample	76	83	1288
7	standard	91	98	1299

1.2. Outlier Removal

A total of 1964 scans were considered outliers and removed using the MAD method

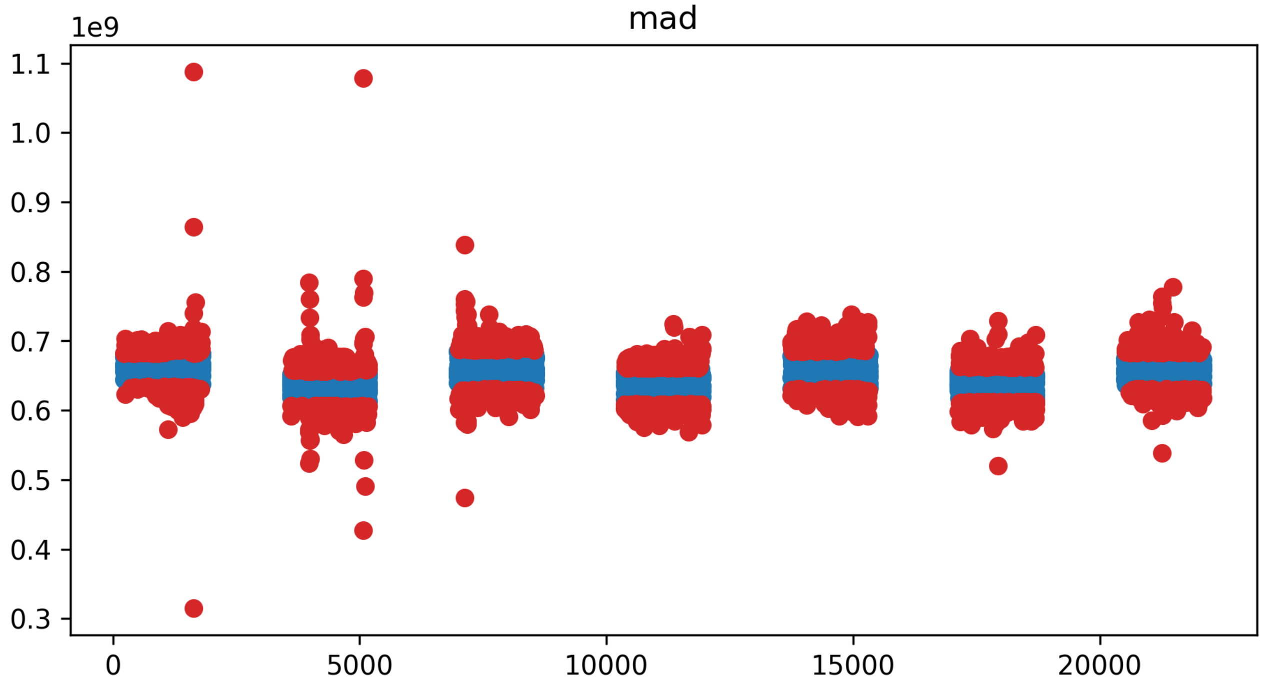

1.3. Total Ion Current (TIC)

TIC of all blocks

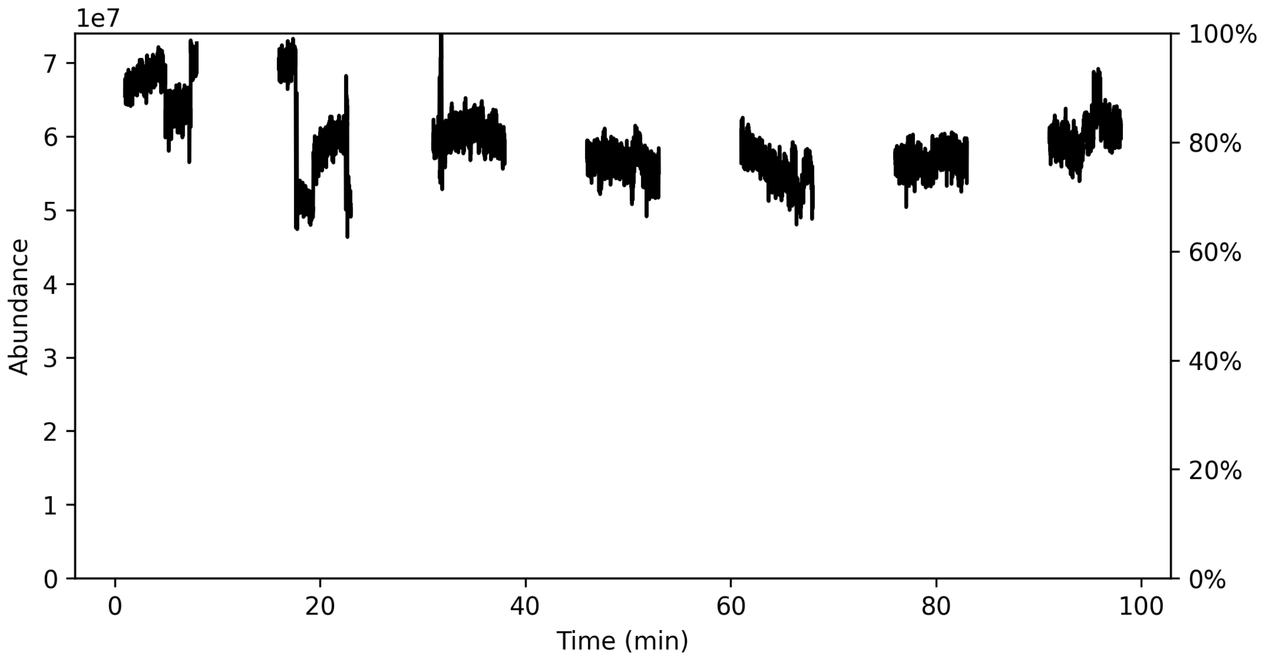

Block	TIC min	TIC max	TIC mean	RSD (%)
1	5.65e+07	7.30e+07	6.70e+07	3.94
2	4.63e+07	7.33e+07	5.99e+07	11.83
3	5.28e+07	7.40e+07	6.05e+07	3.44
4	4.91e+07	6.15e+07	5.63e+07	2.86
5	4.80e+07	6.25e+07	5.57e+07	4.20
6	5.04e+07	6.06e+07	5.67e+07	2.56
7	5.39e+07	6.92e+07	6.05e+07	4.04

2. Block Parameters

The Isotopic Ratio of the blocks were calculated by 'Mean'

2.1. 13C/M0

Block	Number of scans	Effective number of ions	Isotopic Ratio	STD	SEM	RSE
1	1258	1.70e+07	0.209823	0.001724	0.000049	0.000232
2	1280	1.72e+07	0.209763	0.001780	0.000050	0.000237
3	1326	1.80e+07	0.209812	0.001765	0.000048	0.000231
4	1300	1.75e+07	0.209761	0.001749	0.000048	0.000231
5	1324	1.78e+07	0.209957	0.001792	0.000049	0.000234
6	1288	1.73e+07	0.209627	0.001710	0.000048	0.000227
7	1299	1.75e+07	0.209931	0.001791	0.000050	0.000237

Errors and Test Paramters

Block	Acquisition Error (permil)	Shot-Noise (permil)	AE/SN ratio	Shapiro Wilk (p_value)	D'Agostino (p_value)
1	0.232	0.242	0.956	0.952	0.905
2	0.237	0.241	0.984	0.338	0.368
3	0.231	0.236	0.979	0.142	0.499
4	0.231	0.239	0.967	0.076	0.086
5	0.234	0.237	0.990	0.358	0.917
6	0.227	0.241	0.944	0.171	0.180
7	0.237	0.239	0.991	0.188	0.733

Isotopic Ratio and Errors of the Blocks

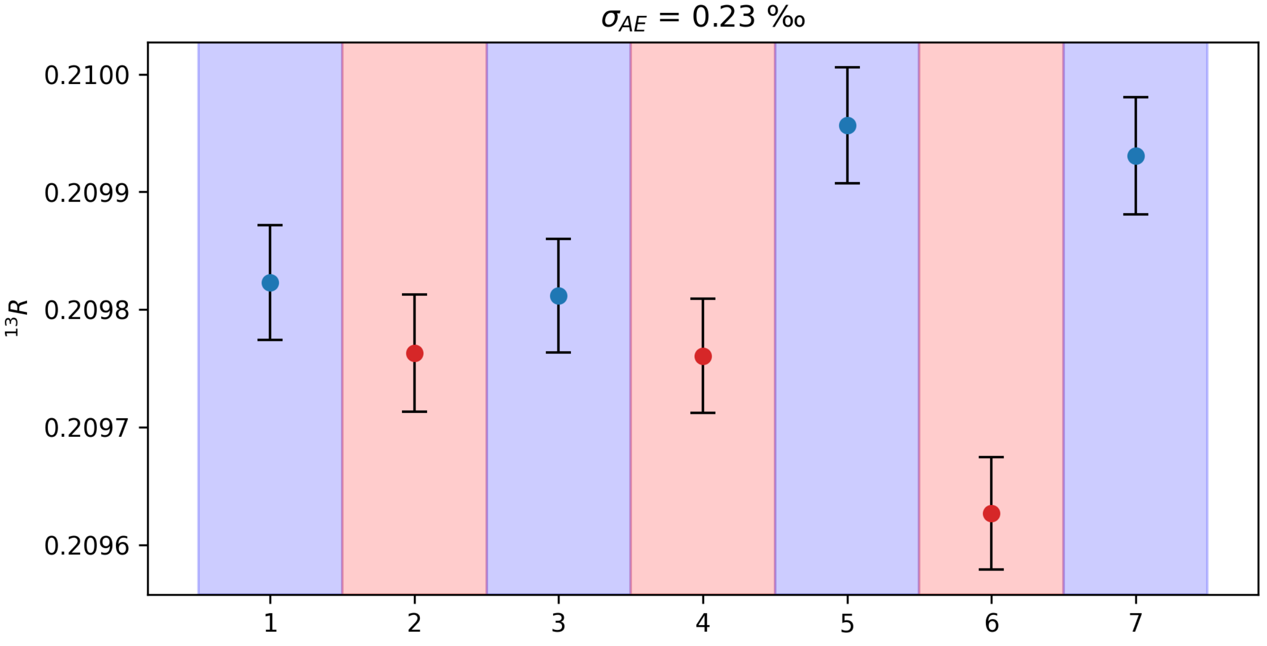

Cumulative Isotopic Ratio

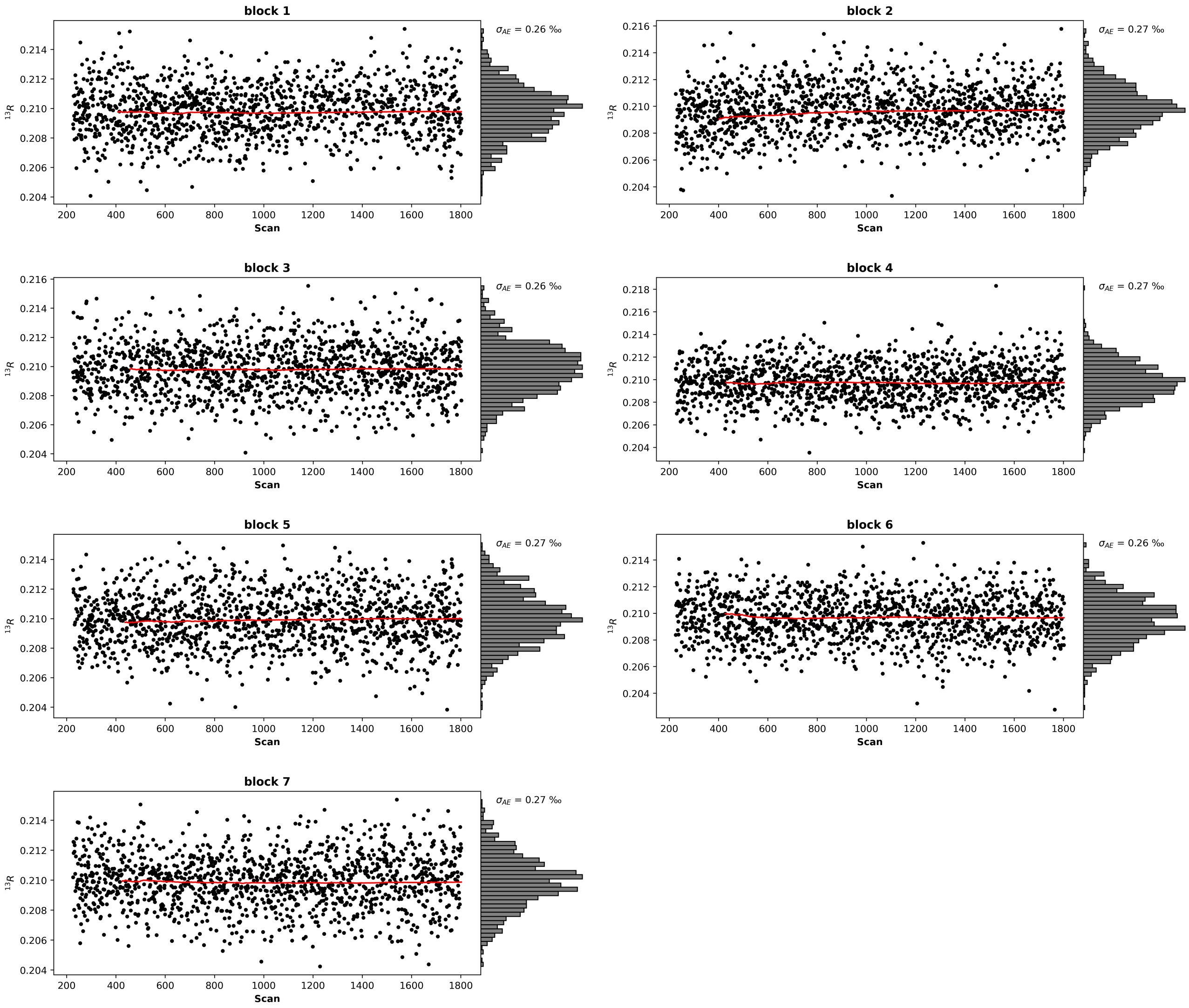

Acquisition Error and Shot-Noise

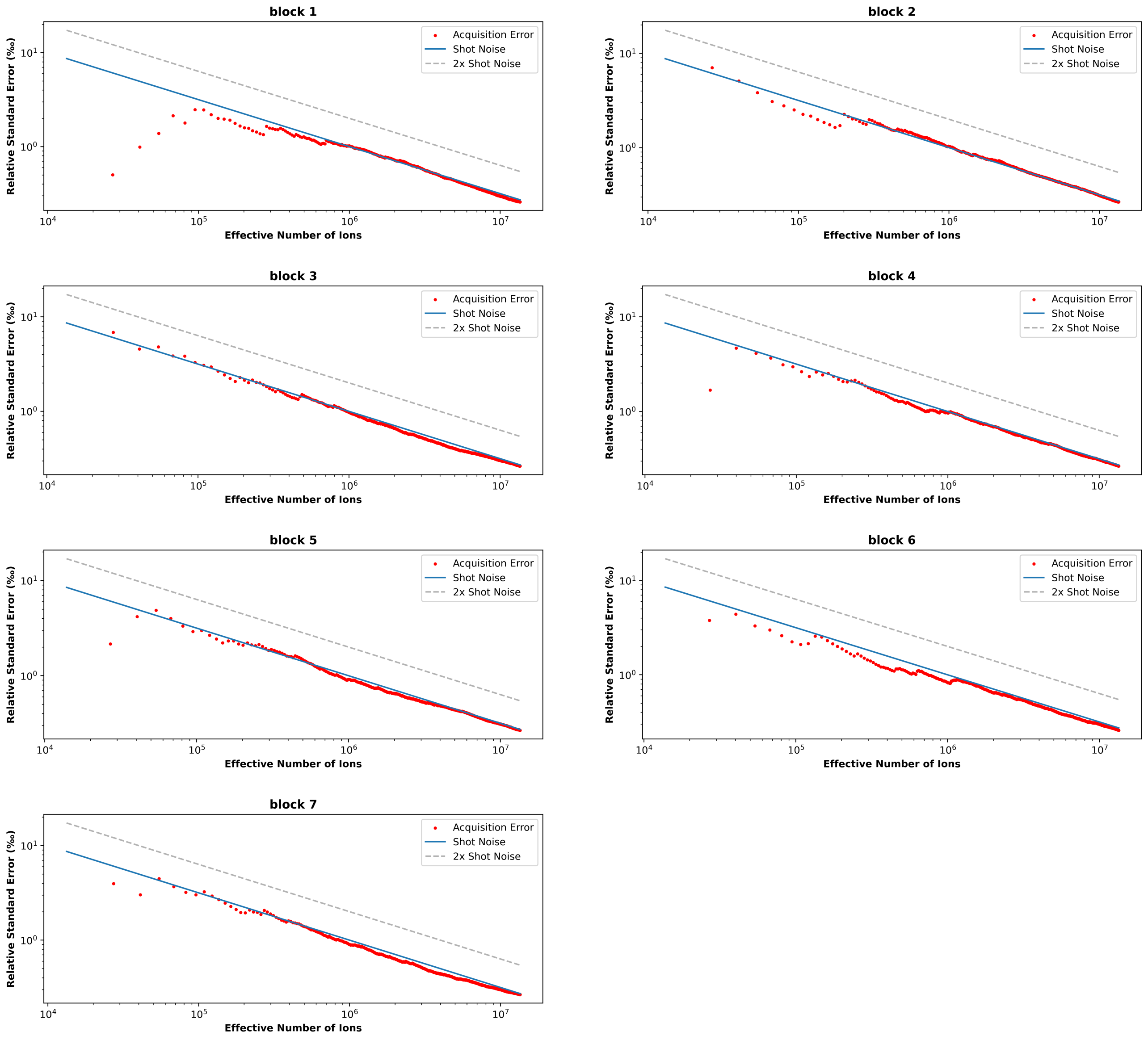

3. Delta Informations

Deltas were calculated by 'Average Of Neighboring Block Ratios'

3.1. 13C

Delta 13C was corrected by -27.80

Block	SEM	Delta corrected	Delta
2	0.24	-28.05	-0.26
4	0.23	-28.37	-0.59
6	0.23	-29.27	-1.51

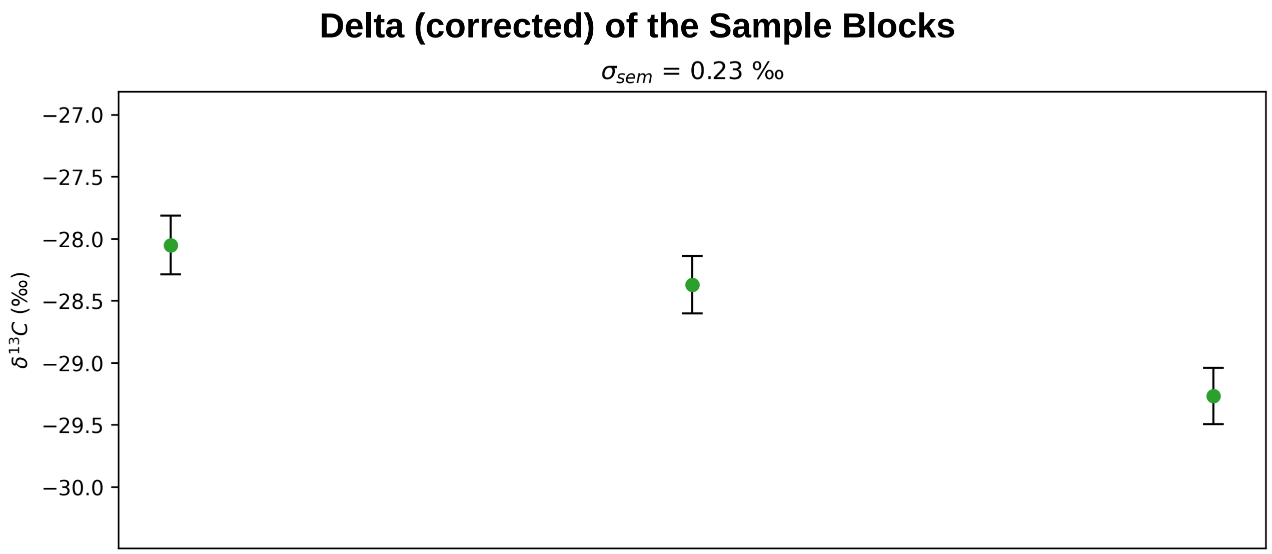

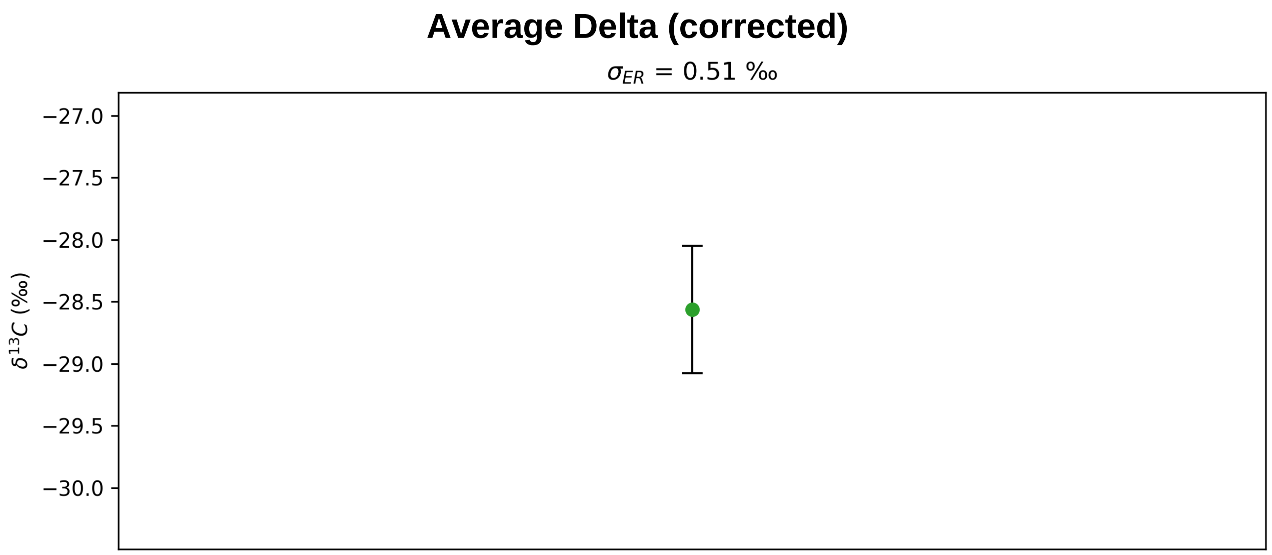

The final corrected average delta was -28.56 with a standard deviation of 0.51. Here the standard deviation is called reproducibility error.
