# Supplementary material for: Advancing Stable Isotope Analysis with Orbitrap-MS for Fatty Acid Methyl Esters and Complex Lipid Matrices
Source: J Am Soc Mass Spectrom. 2025 Jun 17;36(7):1527–35. doi: 10.1021/jasms.5c00092 (PMC12339014; doi:10.1021/jasms.5c00092)
Supplement: Supplementary file 2 [file js5c00092_si_002.zip › reports by IsotoPy Software/standards/Na+Standard9_DI.pdf]

Standard 9 - [M + Na]+
Isotope Analysis report from IsotoPy
Dual Inlet

1. Pre Processing

1.1. Block Time and Scan Information

Information about sample and standard block times and scans:

Block	Injected	Initial Time	End Time	Number of scans
1	standard	1	5	739
2	sample	6	10	746
3	standard	11	15	767
4	sample	16	20	728
5	standard	21	25	743
6	sample	26	30	742
7	standard	31	35	750

1.2. Outlier Removal

A total of 1089 scans were considered outliers and removed using the MAD method

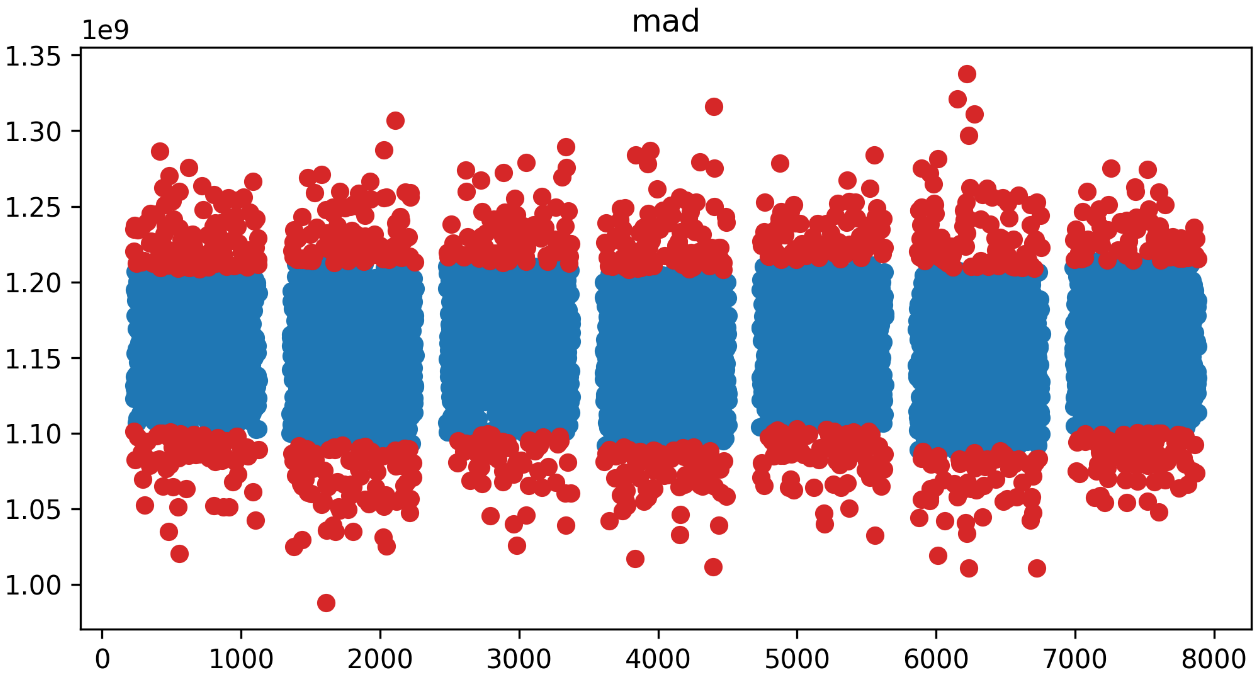

1.3. Total Ion Current (TIC)

TIC of all blocks

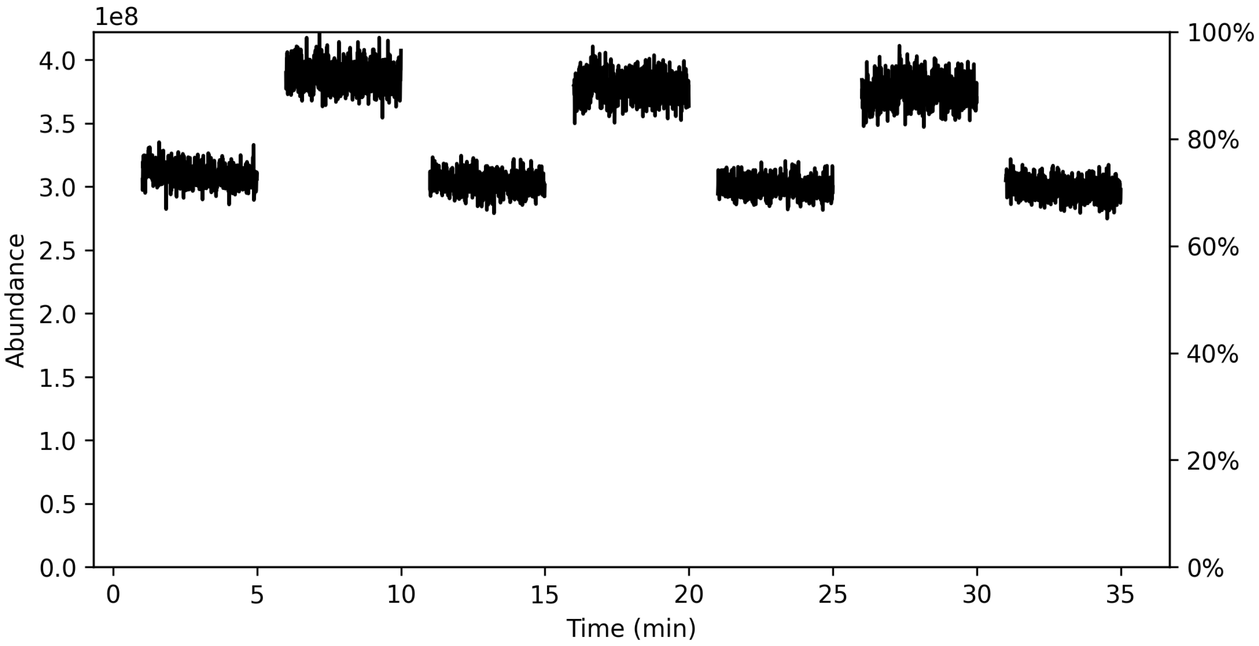

Block	TIC min	TIC max	TIC mean	RSD (%)
1	2.82e+08	3.35e+08	3.10e+08	2.44
2	3.54e+08	4.22e+08	3.88e+08	2.56
3	2.79e+08	3.24e+08	3.04e+08	2.41
4	3.50e+08	4.11e+08	3.79e+08	2.57
5	2.82e+08	3.20e+08	3.00e+08	2.27
6	3.47e+08	4.11e+08	3.76e+08	2.73
7	2.75e+08	3.22e+08	2.99e+08	2.36

2. Block Parameters

The Isotopic Ratio of the blocks were calculated by 'Mean'

2.1. 13C/M0

Block	Number of scans	Effective number of ions	Isotopic Ratio	STD	SEM	RSE
1	739	1.62e+07	0.209276	0.001411	0.000052	0.000248
2	746	1.63e+07	0.209476	0.001421	0.000052	0.000248
3	767	1.67e+07	0.209548	0.001366	0.000049	0.000235
4	728	1.59e+07	0.209380	0.001286	0.000048	0.000228
5	743	1.62e+07	0.209277	0.001298	0.000048	0.000227
6	742	1.61e+07	0.209715	0.001351	0.000050	0.000236
7	750	1.63e+07	0.209626	0.001380	0.000050	0.000240

Errors and Test Paramters

Block	Acquisition Error (permil)	Shot-Noise (permil)	AE/SN ratio	Shapiro Wilk (p_value)	D'Agostino (p_value)
1	0.248	0.249	0.996	0.171	0.072
2	0.248	0.248	1.001	0.355	0.297
3	0.235	0.245	0.961	0.398	0.355
4	0.228	0.251	0.908	0.136	0.060
5	0.227	0.248	0.916	0.362	0.203
6	0.236	0.249	0.949	0.632	0.354
7	0.240	0.248	0.969	0.982	1.000

Isotopic Ratio and Errors of the Blocks

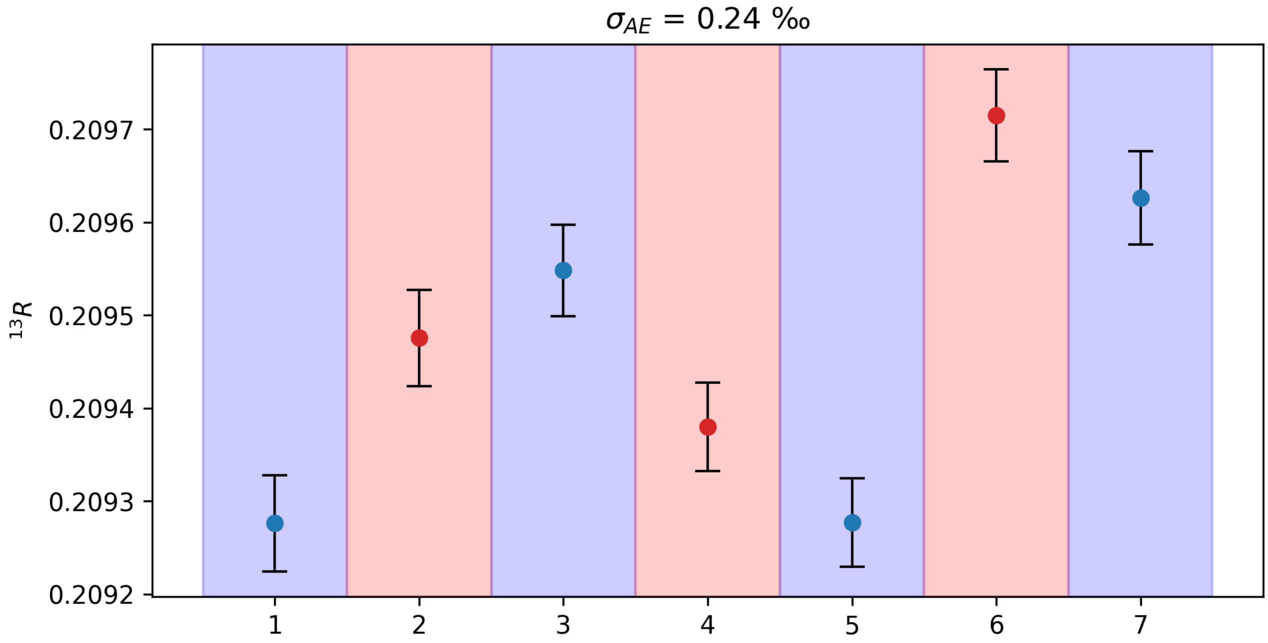

Cumulative Isotopic Ratio

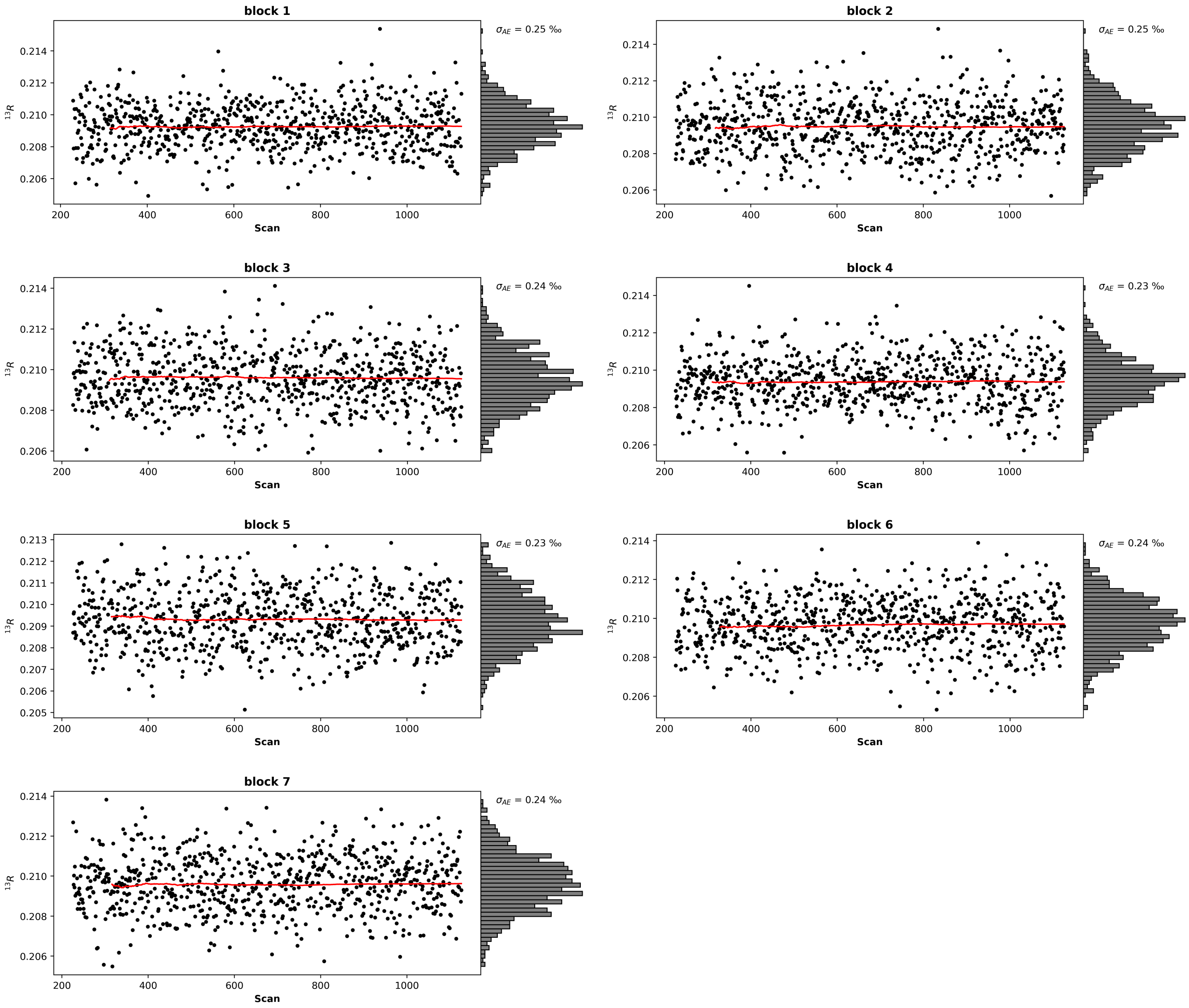

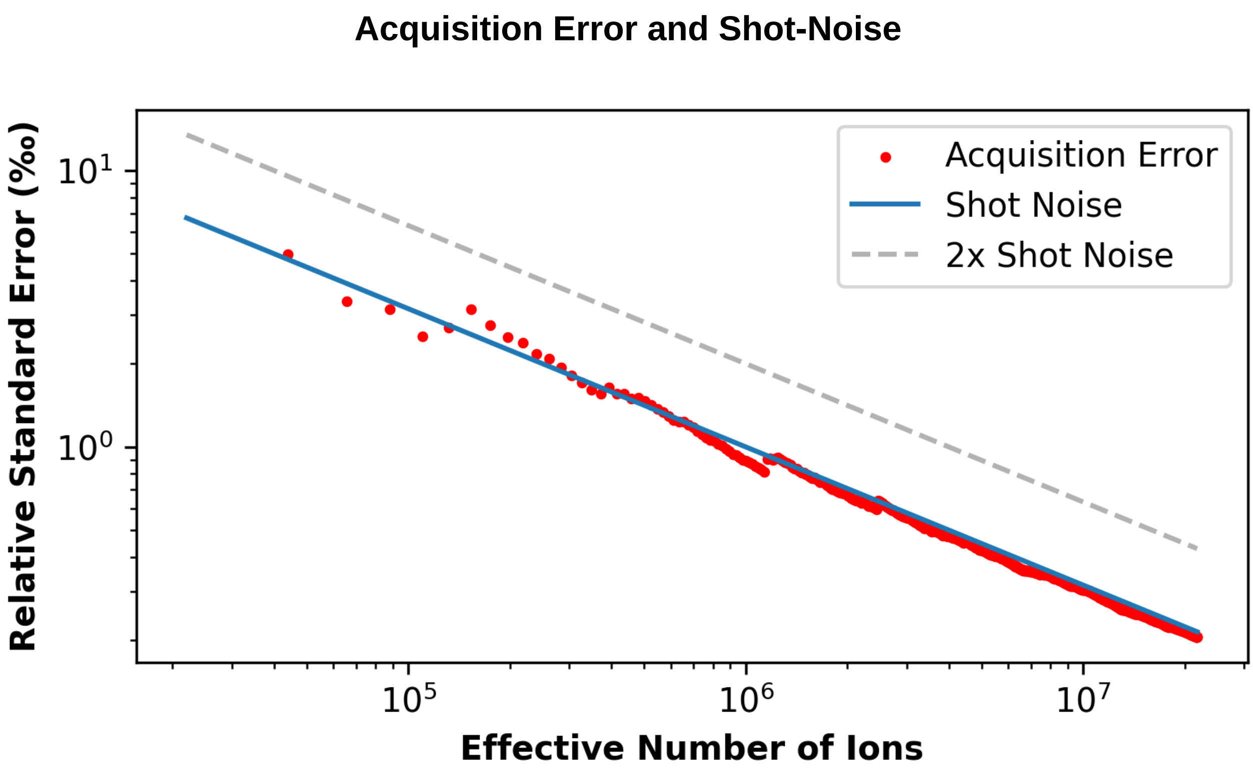
Acquisition Error and Shot-Noise
Relative Standard Error (‰)
10^1
10^0
10^5
10^6
10^7
Effective Number of Ions
Acquisition Error
Shot Noise
2x Shot Noise

3. Delta Informations

Deltas were calculated by 'Average Of Neighboring Block Ratios'

3.1. 13C

Delta 13C was corrected by -27.80

Block	SEM	Delta corrected	Delta
2	0.25	-27.51	0.30
4	0.23	-27.95	-0.16
6	0.24	-26.58	1.26

Delta (corrected) of the Sample Blocks

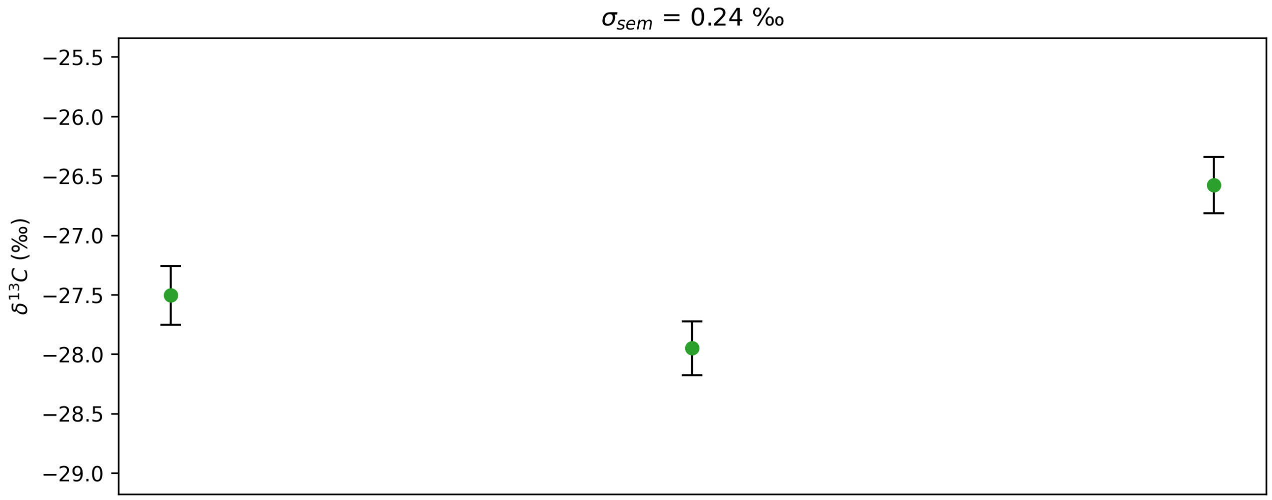

Average Delta (corrected)

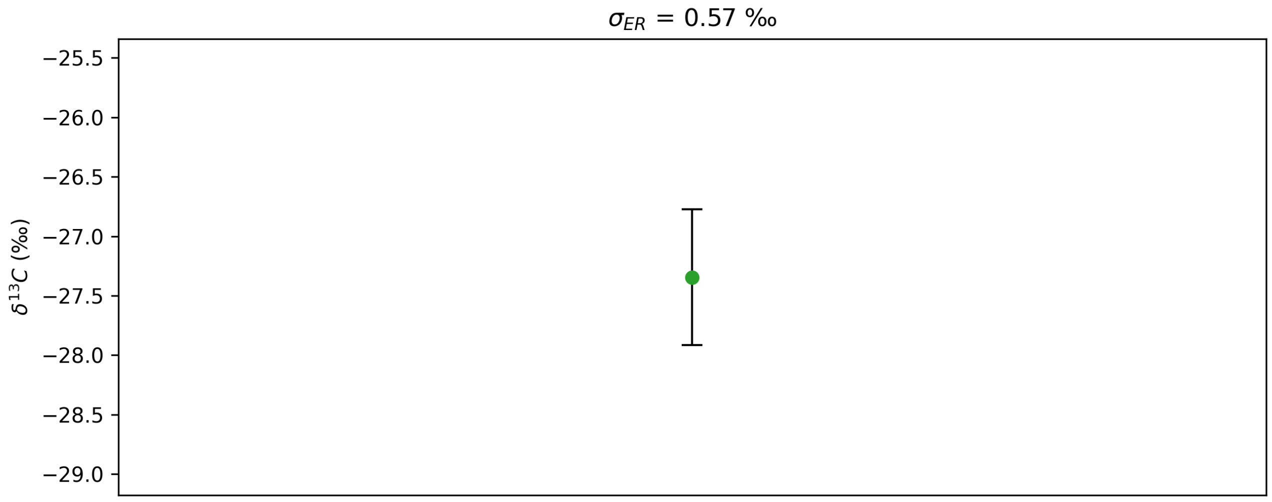

The final corrected average delta was -27.34 with a standard deviation of 0.57. Here the standard deviation is called reproducibility error.
