# Supplementary material for: Advancing Stable Isotope Analysis with Orbitrap-MS for Fatty Acid Methyl Esters and Complex Lipid Matrices
Source: J Am Soc Mass Spectrom. 2025 Jun 17;36(7):1527–35. doi: 10.1021/jasms.5c00092 (PMC12339014; doi:10.1021/jasms.5c00092)
Supplement: Supplementary file 2 [file js5c00092_si_002.zip › reports by IsotoPy Software/standards/Na+Standard5_DI.pdf]

Standard 5 - [M + Na]+
Isotope Analysis report from IsotoPy
Dual Inlet

1. Pre Processing

1.1. Block Time and Scan Information

Information about sample and standard block times and scans:

Block	Injected	Initial Time	End Time	Number of scans
1	standard	1	5	751
2	sample	6	10	707
3	standard	11	15	727
4	sample	16	20	698
5	standard	21	25	743
6	sample	26	30	725
7	standard	31	35	728

1.2. Outlier Removal

A total of 1225 scans were considered outliers and removed using the MAD method

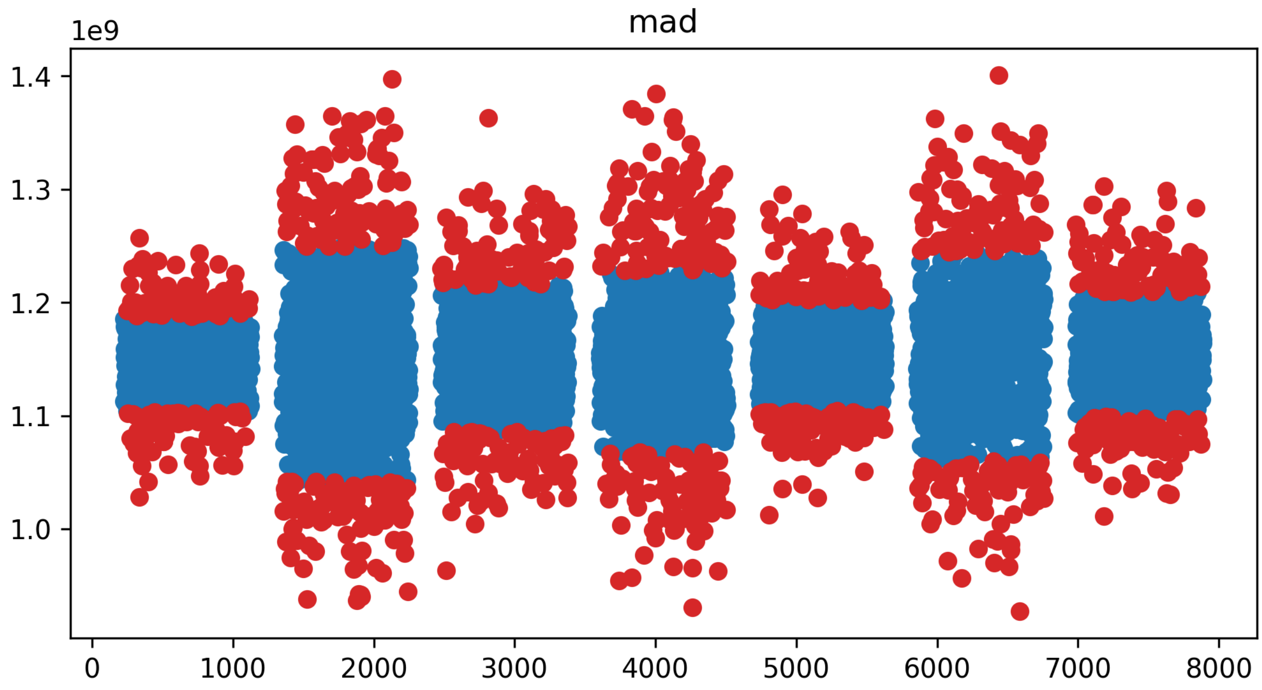

1.3. Total Ion Current (TIC)

TIC of all blocks

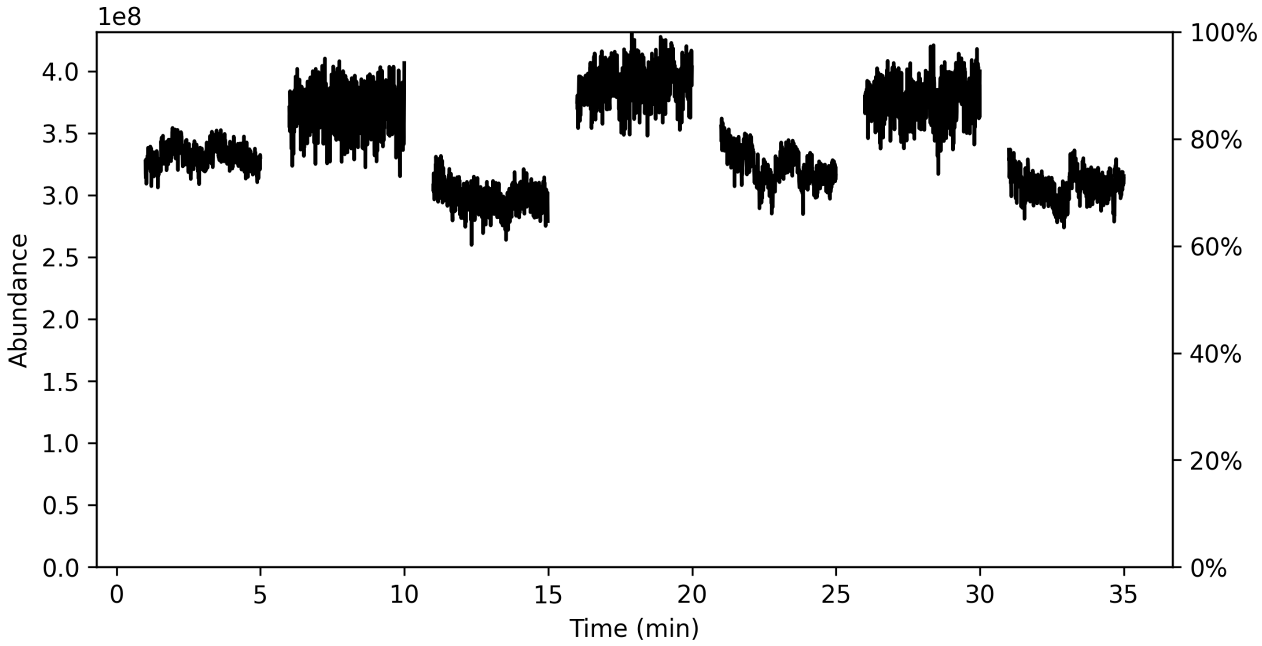

Block	TIC min	TIC max	TIC mean	RSD (%)
1	3.06e+08	3.54e+08	3.32e+08	2.46
2	3.15e+08	4.10e+08	3.69e+08	4.43
3	2.60e+08	3.32e+08	2.98e+08	3.41
4	3.48e+08	4.32e+08	3.91e+08	3.70
5	2.85e+08	3.62e+08	3.23e+08	4.12
6	3.17e+08	4.21e+08	3.78e+08	3.92
7	2.74e+08	3.37e+08	3.07e+08	3.59

2. Block Parameters

The Isotopic Ratio of the blocks were calculated by 'Mean'

2.1. 13C/M0

Block	Number of scans	Effective number of ions	Isotopic Ratio	STD	SEM	RSE
1	751	1.57e+07	0.210116	0.001467	0.000053	0.000255
2	707	1.50e+07	0.210742	0.001368	0.000051	0.000244
3	727	1.55e+07	0.210537	0.001395	0.000052	0.000246
4	698	1.51e+07	0.210327	0.001321	0.000050	0.000238
5	743	1.61e+07	0.209940	0.001368	0.000050	0.000239
6	725	1.58e+07	0.210166	0.001351	0.000050	0.000239
7	728	1.59e+07	0.209741	0.001328	0.000049	0.000234

Errors and Test Paramters

Block	Acquisition Error (permil)	Shot-Noise (permil)	AE/SN ratio	Shapiro Wilk (p_value)	D'Agostino (p_value)
1	0.255	0.253	1.008	0.563	0.495
2	0.244	0.258	0.944	0.397	0.642
3	0.246	0.254	0.968	0.151	0.547
4	0.238	0.257	0.924	0.733	0.326
5	0.239	0.249	0.960	0.397	0.299
6	0.239	0.251	0.950	0.470	0.316
7	0.234	0.250	0.936	0.380	0.298

Isotopic Ratio and Errors of the Blocks

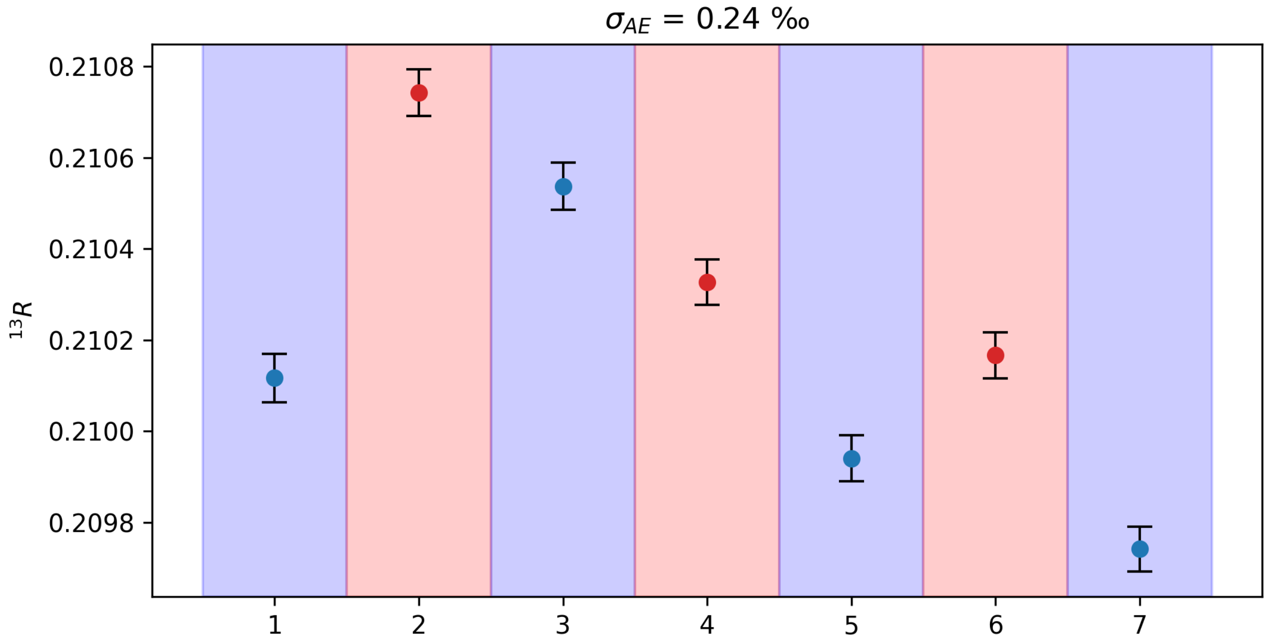

Cumulative Isotopic Ratio

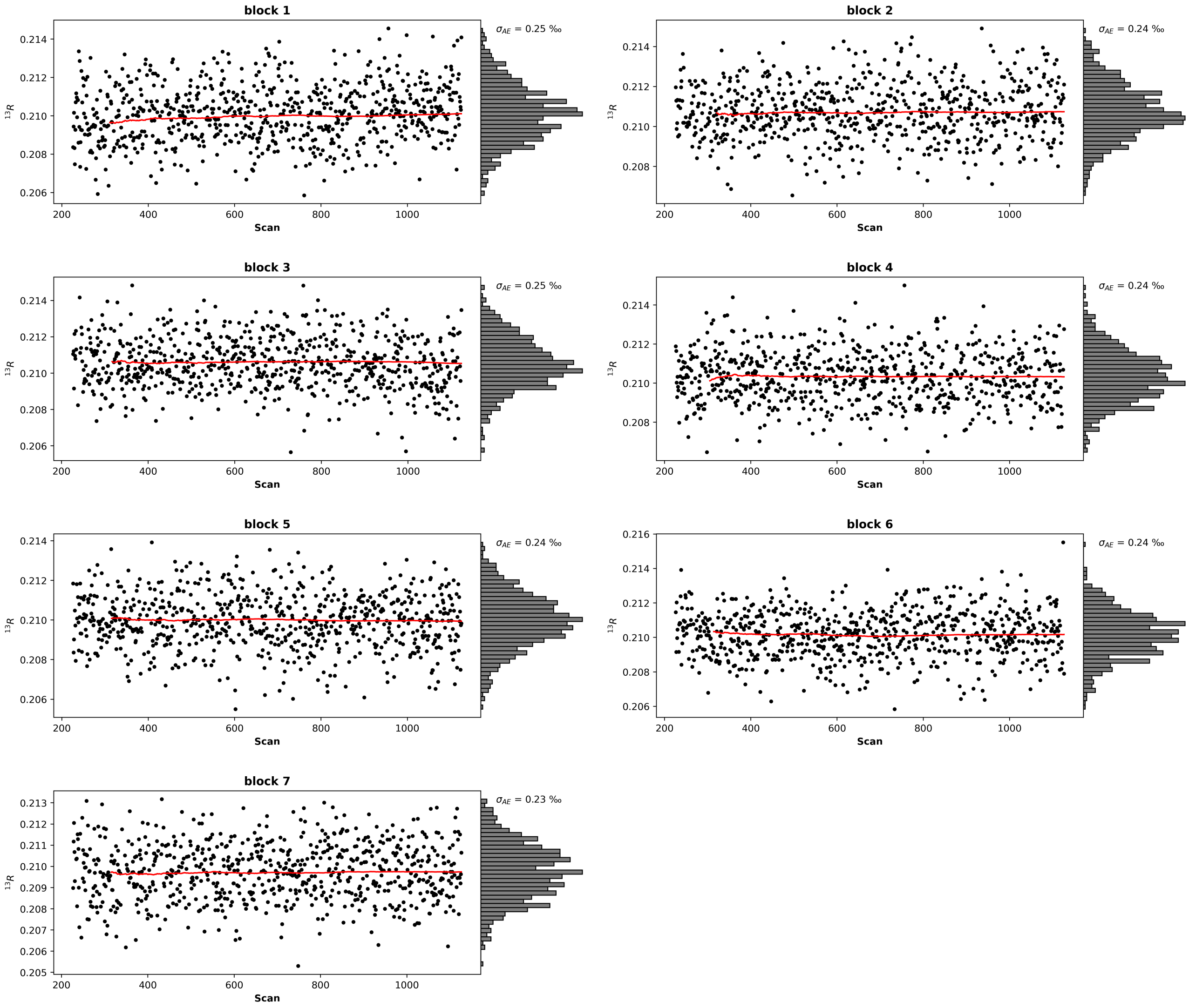

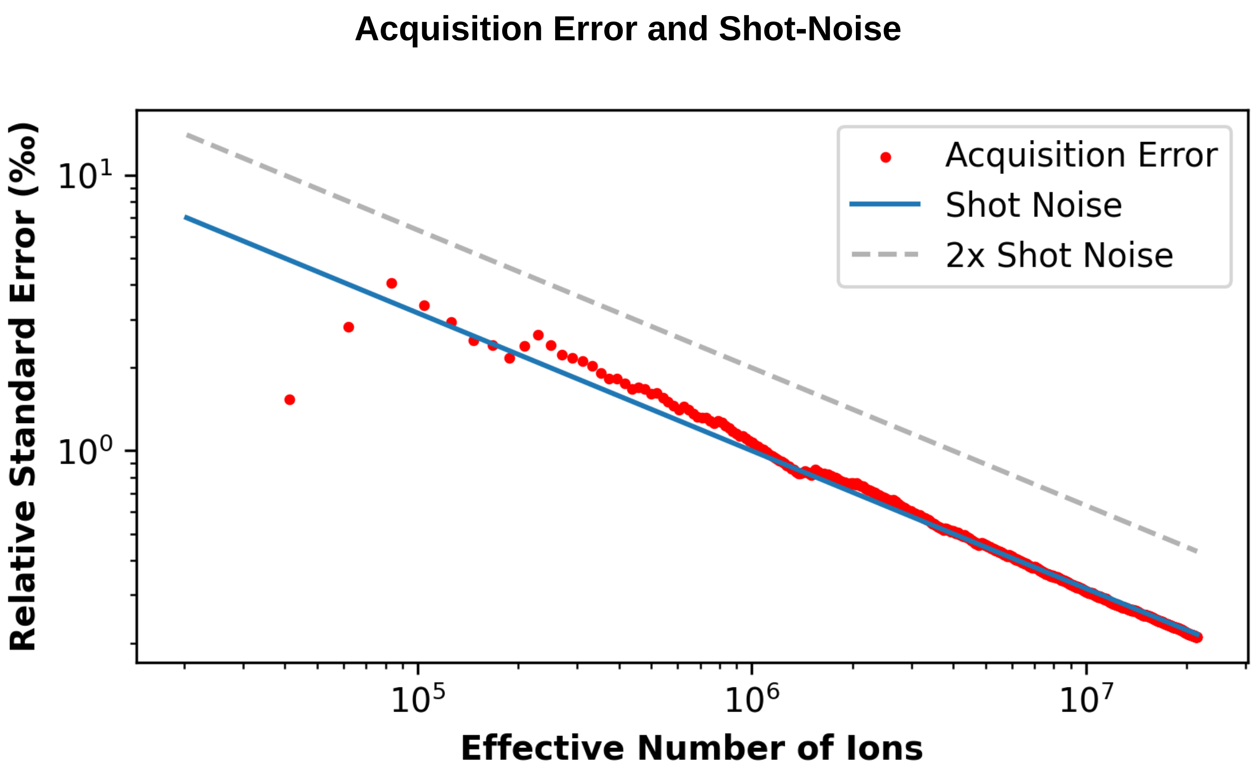
Acquisition Error and Shot-Noise
Relative Standard Error (‰)
10¹
10⁰
10⁵
10⁶
10⁷
Effective Number of Ions
Acquisition Error
Shot Noise
2x Shot Noise

3. Delta Informations

Deltas were calculated by 'Average Of Neighboring Block Ratios'

3.1. 13C

Delta 13C was corrected by -27.80

Block	SEM	Delta corrected	Delta
2	0.24	-25.88	1.98
4	0.24	-27.39	0.42
6	0.24	-26.29	1.55

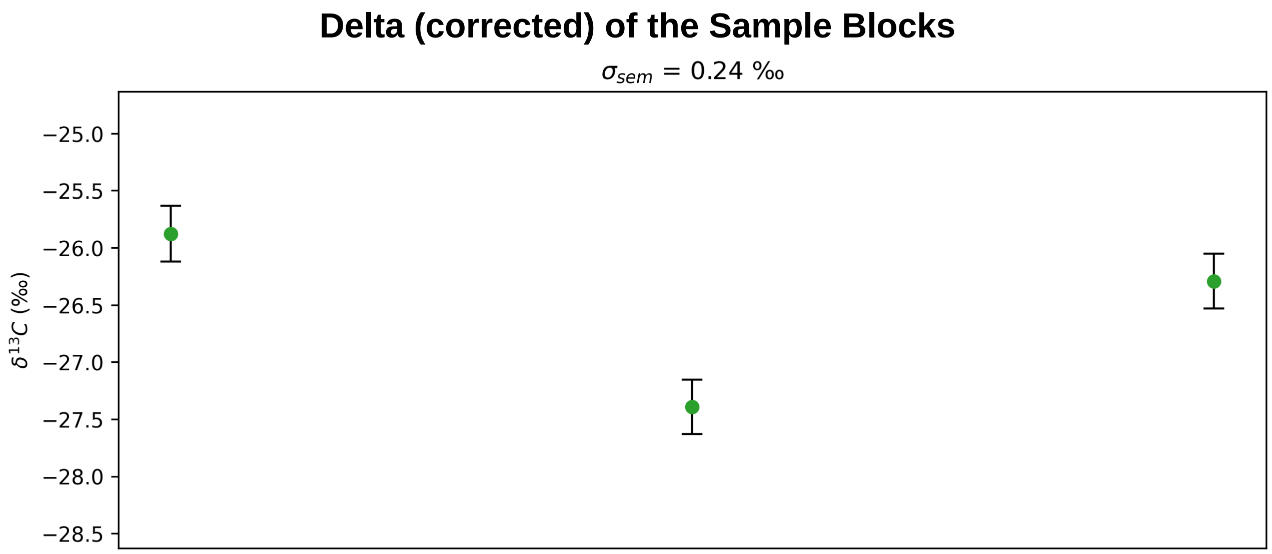

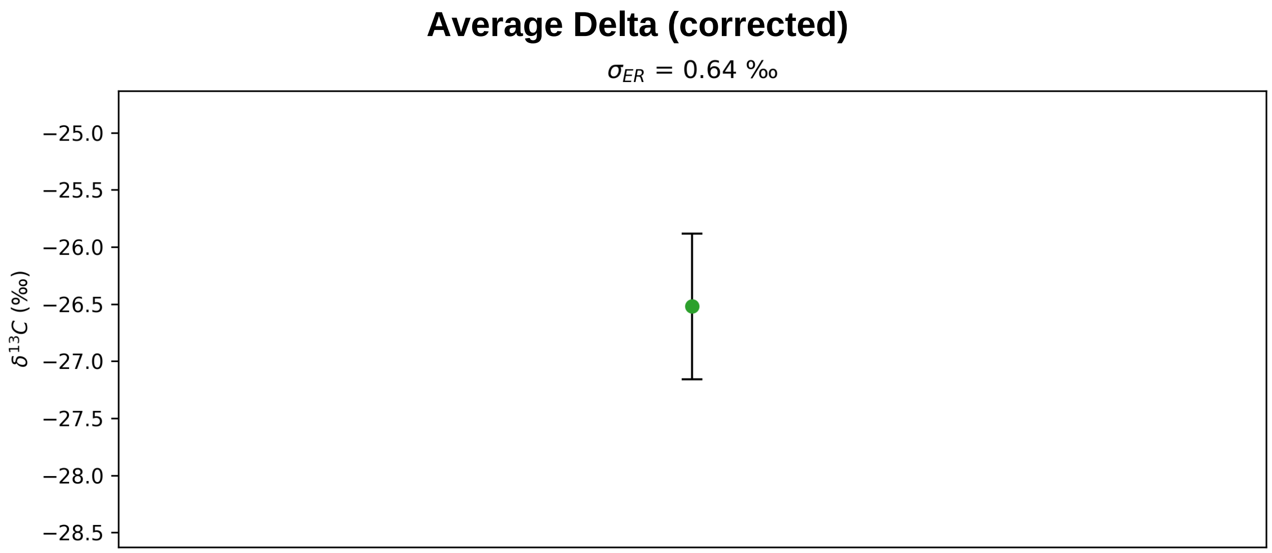

The final corrected average delta was -26.52 with a standard deviation of 0.64. Here the standard deviation is called reproducibility error.
