# Supplementary material for: Advancing Stable Isotope Analysis with Orbitrap-MS for Fatty Acid Methyl Esters and Complex Lipid Matrices
Source: J Am Soc Mass Spectrom. 2025 Jun 17;36(7):1527–35. doi: 10.1021/jasms.5c00092 (PMC12339014; doi:10.1021/jasms.5c00092)
Supplement: Supplementary file 2 [file js5c00092_si_002.zip › reports by IsotoPy Software/standards/Na+Standard2_FI.pdf]

Standard 2 - [M + Na]+
Isotope Analysis report from IsotoPy

Flow Injection

1. Pre Processing

1.1. Block Time and Scan Information

Information about sample and standard block times and scans:

Block	Injected	Initial Time	End Time	Number of scans
1	standard	1	8	1312
2	sample	16	23	1275
3	standard	31	38	1227
4	sample	46	53	1299
5	standard	61	68	1179
6	sample	76	83	1295
7	standard	91	98	1272

1.2. Outlier Removal

A total of 2180 scans were considered outliers and removed using the MAD method

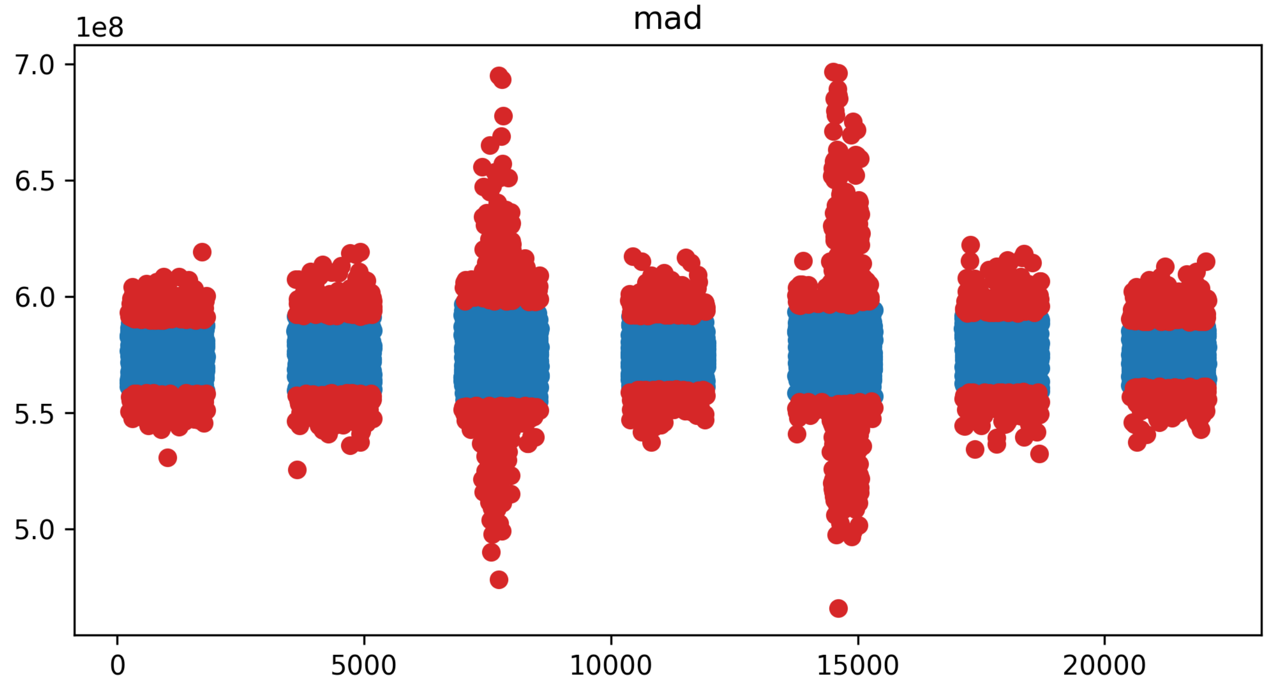

1.3. Total Ion Current (TIC)

TIC of all blocks

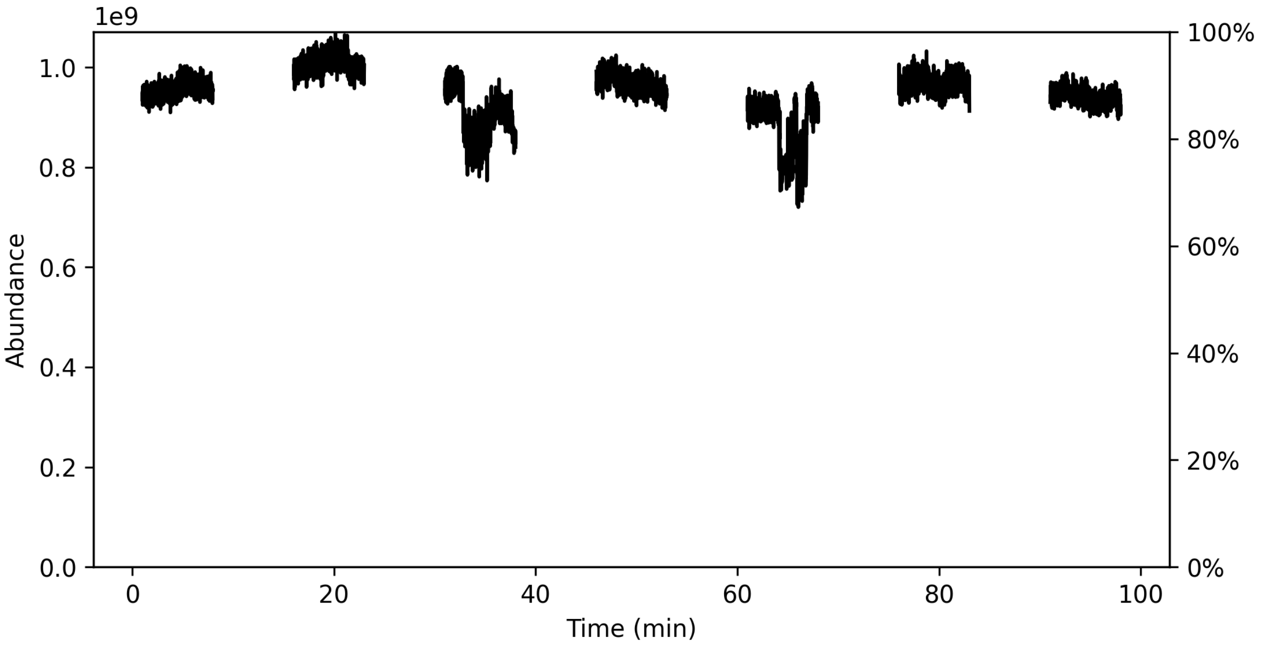

Block	TIC min	TIC max	TIC mean	RSD (%)
1	9.10e+08	1.00e+09	9.57e+08	1.58
2	9.56e+08	1.07e+09	1.01e+09	1.81
3	7.73e+08	1.00e+09	9.14e+08	5.09
4	9.14e+08	1.02e+09	9.69e+08	1.82
5	7.20e+08	9.69e+08	8.98e+08	5.27
6	9.13e+08	1.03e+09	9.69e+08	1.71
7	8.96e+08	9.89e+08	9.42e+08	1.65

2. Block Parameters

The Isotopic Ratio of the blocks were calculated by 'Mean'

2.1. 13C/M0

Block	Number of scans	Effective number of ions	Isotopic Ratio	STD	SEM	RSE
1	1312	2.14e+07	0.209228	0.001803	0.000050	0.000238
2	1275	2.06e+07	0.209118	0.001793	0.000050	0.000240
3	1227	1.98e+07	0.209594	0.001802	0.000051	0.000245
4	1299	2.08e+07	0.208819	0.001779	0.000049	0.000236
5	1179	1.89e+07	0.209010	0.001834	0.000053	0.000255
6	1295	2.08e+07	0.208783	0.001816	0.000050	0.000242
7	1272	2.05e+07	0.209432	0.001779	0.000050	0.000238

Errors and Test Paramters

Block	Acquisition Error (permil)	Shot-Noise (permil)	AE/SN ratio	Shapiro Wilk (p_value)	D'Agostino (p_value)
1	0.238	0.216	1.099	0.095	0.049
2	0.240	0.220	1.089	0.753	0.990
3	0.245	0.225	1.091	0.188	0.072
4	0.236	0.219	1.079	0.044	0.050
5	0.255	0.230	1.111	0.524	0.885
6	0.242	0.219	1.101	0.177	0.076
7	0.238	0.221	1.077	0.024	0.115

Isotopic Ratio and Errors of the Blocks

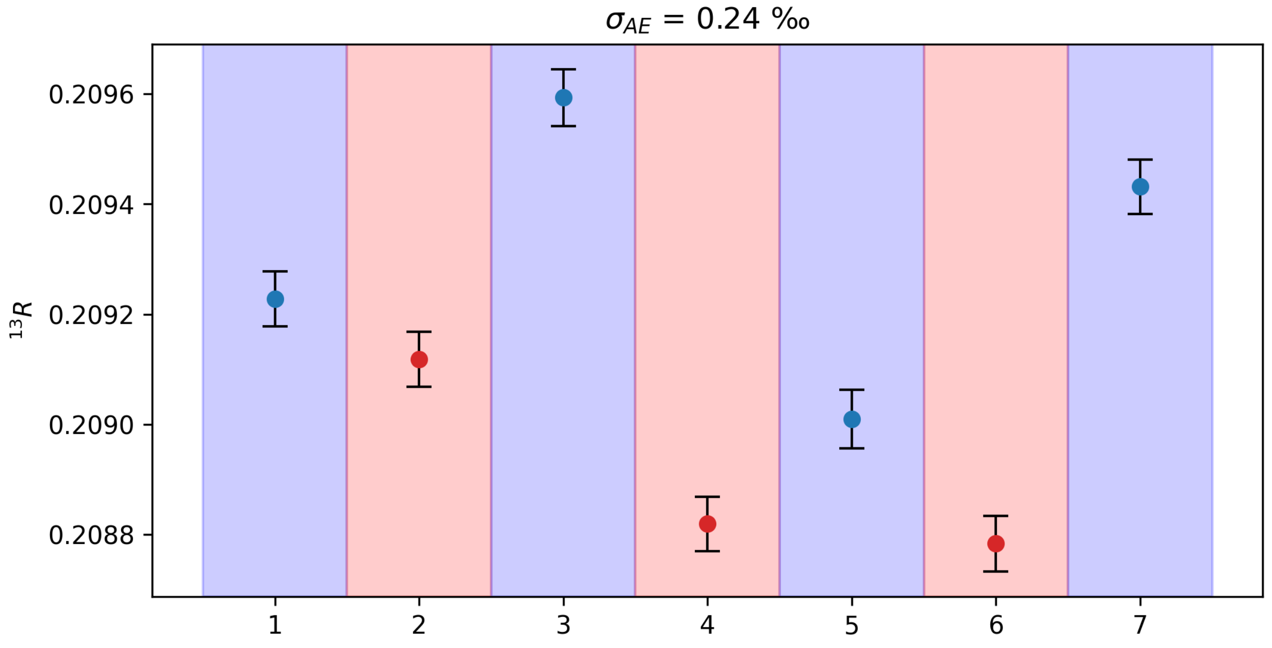

Cumulative Isotopic Ratio

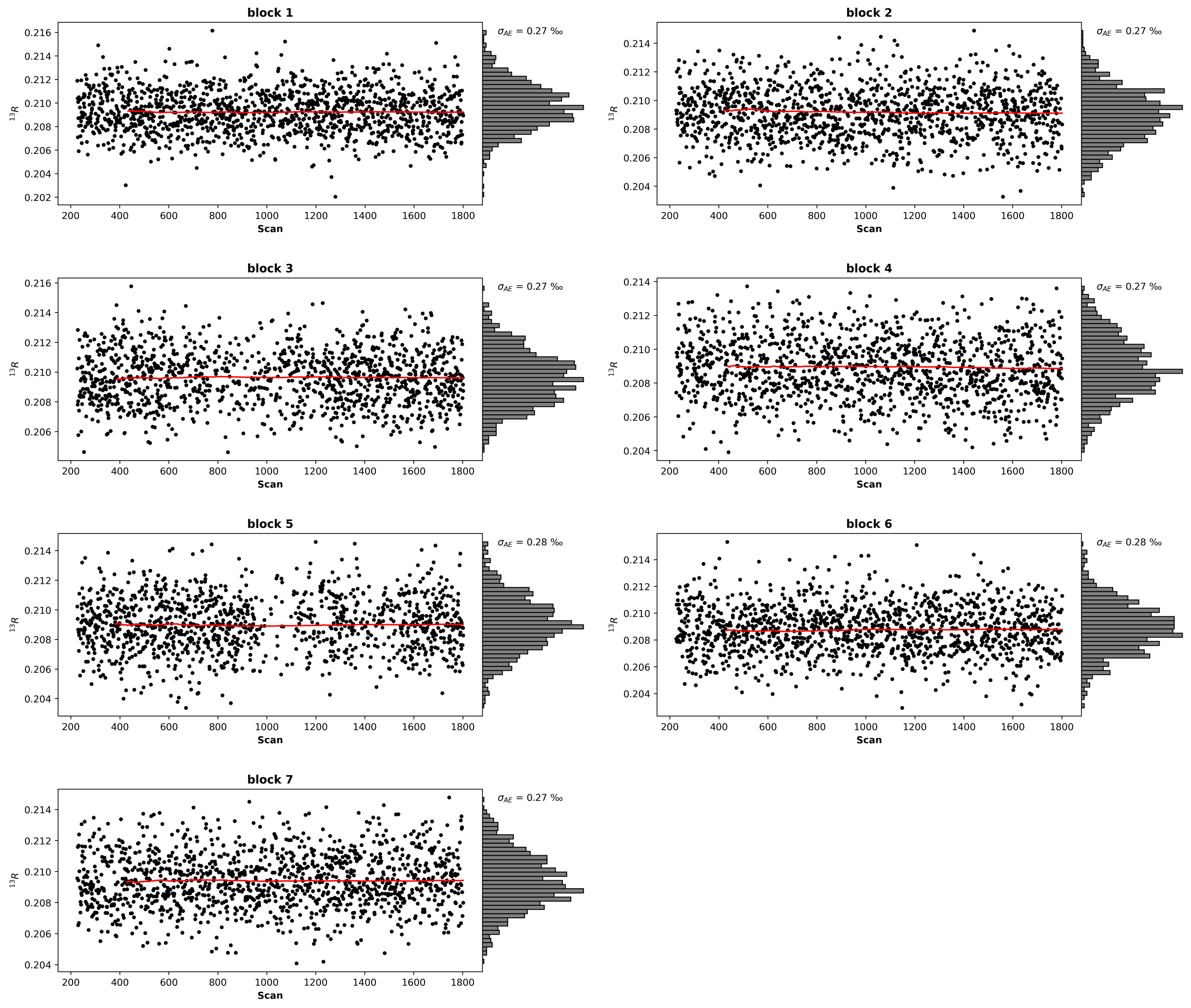

Acquisition Error and Shot-Noise

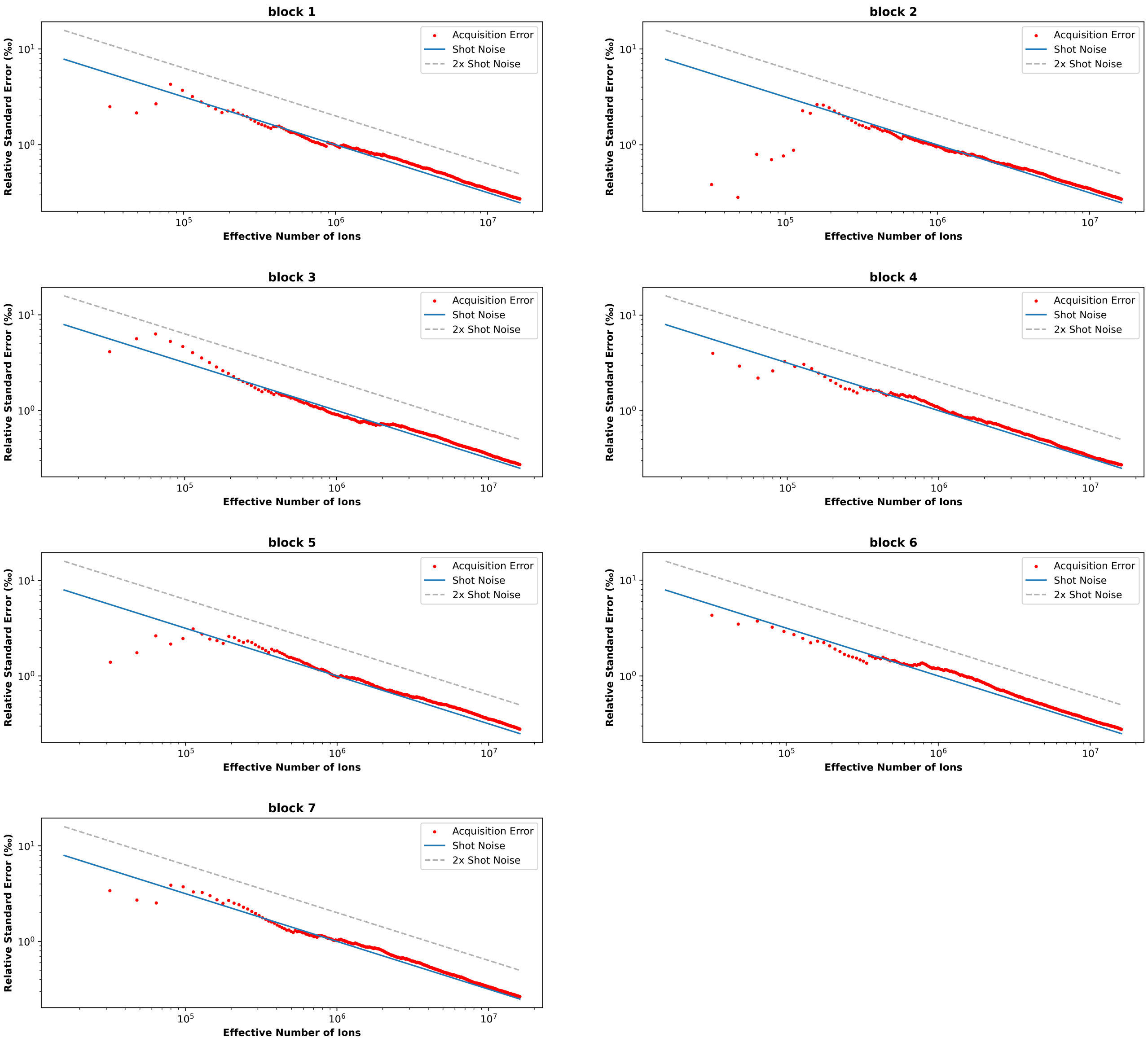

3. Delta Informations

Deltas were calculated by 'Average Of Neighboring Block Ratios'

3.1. 13C

Delta 13C was corrected by -27.80

Block	SEM	Delta corrected	Delta
2	0.24	-29.16	-1.40
4	0.24	-30.04	-2.31
6	0.24	-29.83	-2.09

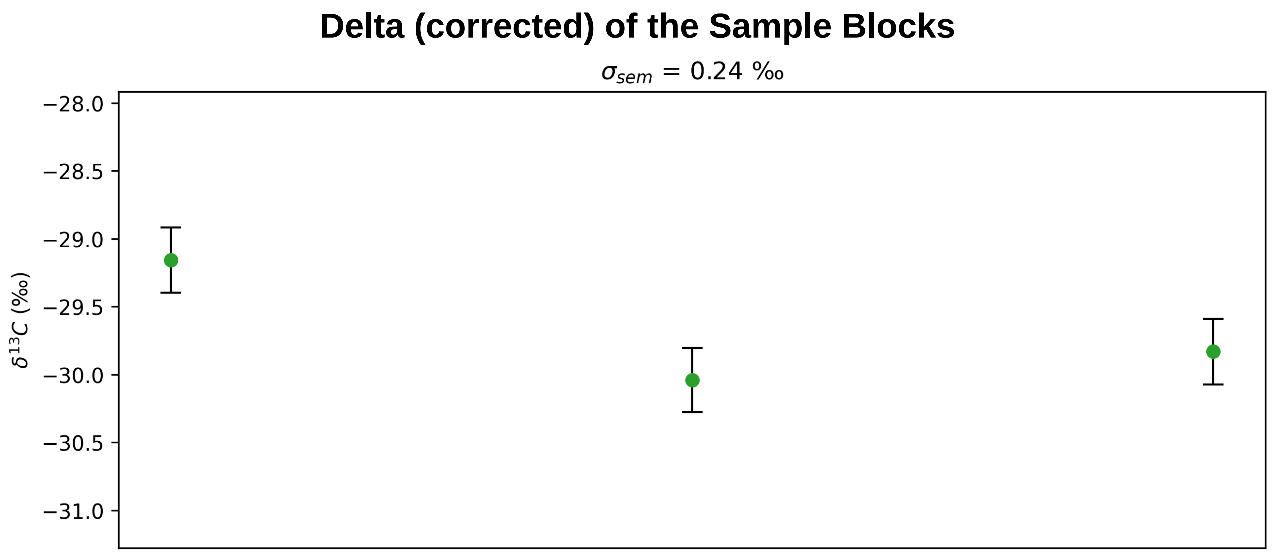

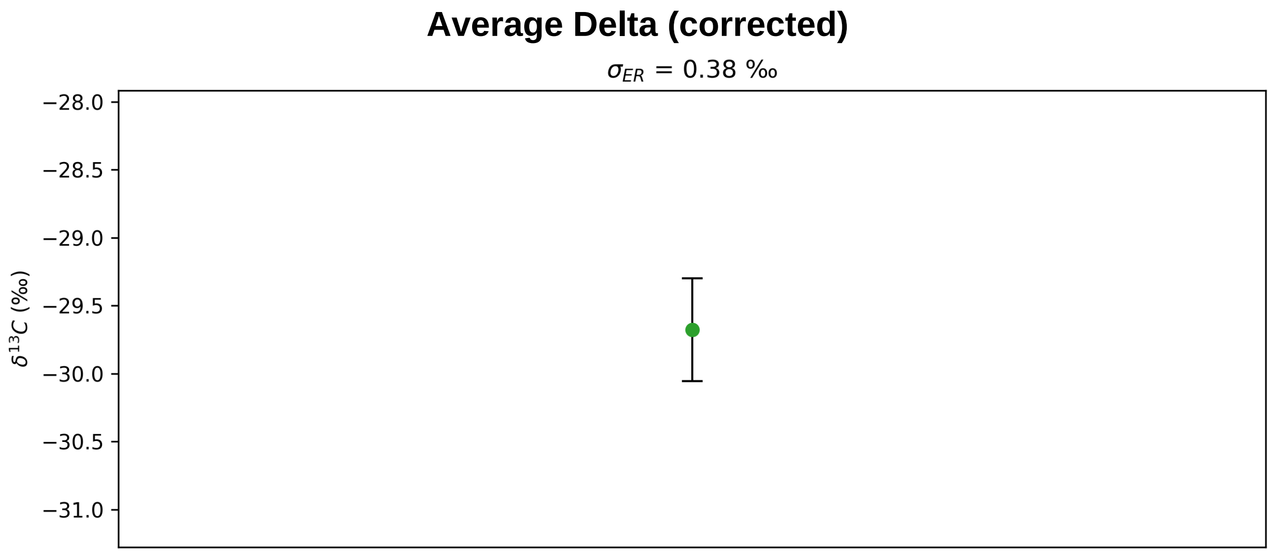

The final corrected average delta was -29.68 with a standard deviation of 0.38. Here the standard deviation is called reproducibility error.
