# Supplementary material for: Advancing Stable Isotope Analysis with Orbitrap-MS for Fatty Acid Methyl Esters and Complex Lipid Matrices
Source: J Am Soc Mass Spectrom. 2025 Jun 17;36(7):1527–35. doi: 10.1021/jasms.5c00092 (PMC12339014; doi:10.1021/jasms.5c00092)
Supplement: Supplementary file 2 [file js5c00092_si_002.zip › reports by IsotoPy Software/standards/Na+Standard7_DI.pdf]

Standard 7 - [M + Na]+
Isotope Analysis report from IsotoPy

Dual Inlet

1. Pre Processing

1.1. Block Time and Scan Information

Information about sample and standard block times and scans:

Block	Injected	Initial Time	End Time	Number of scans
1	standard	1	5	731
2	sample	6	10	743
3	standard	11	15	731
4	sample	16	20	721
5	standard	21	25	724
6	sample	26	30	721
7	standard	31	35	744

1.2. Outlier Removal

A total of 1189 scans were considered outliers and removed using the MAD method

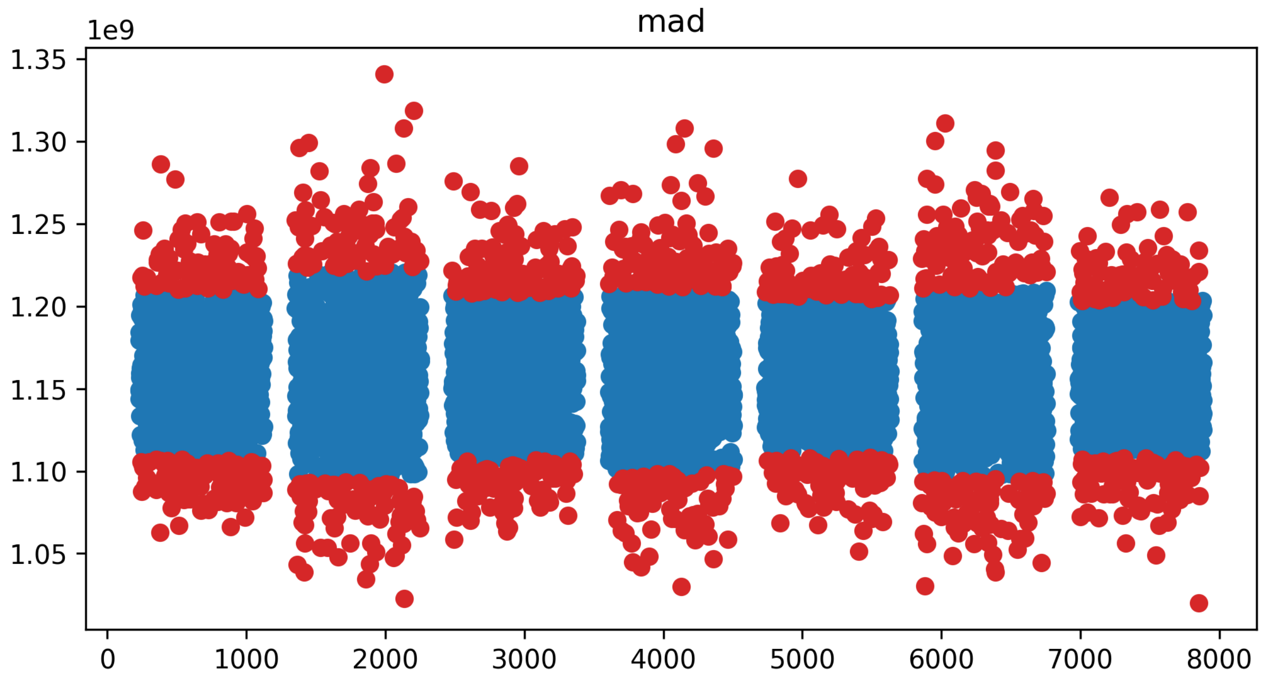

1.3. Total Ion Current (TIC)

TIC of all blocks

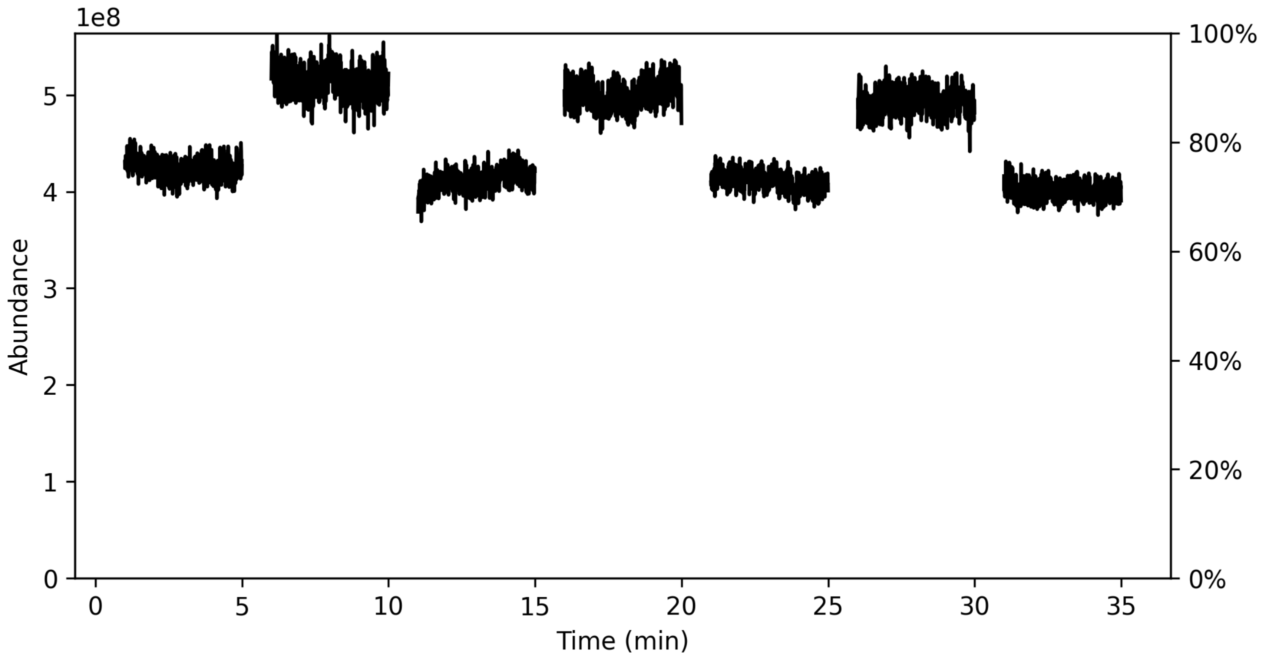

Block	TIC min	TIC max	TIC mean	RSD (%)
1	3.93e+08	4.55e+08	4.25e+08	2.42
2	4.61e+08	5.64e+08	5.15e+08	2.99
3	3.69e+08	4.43e+08	4.12e+08	2.71
4	4.61e+08	5.36e+08	5.01e+08	2.63
5	3.82e+08	4.37e+08	4.11e+08	2.35
6	4.42e+08	5.30e+08	4.93e+08	2.61
7	3.76e+08	4.31e+08	4.01e+08	2.19

2. Block Parameters

The Isotopic Ratio of the blocks were calculated by 'Mean'

2.1. 13C/M0

Block	Number of scans	Effective number of ions	Isotopic Ratio	STD	SEM	RSE
1	731	1.44e+07	0.210018	0.001403	0.000052	0.000247
2	743	1.47e+07	0.209881	0.001338	0.000049	0.000234
3	731	1.44e+07	0.209935	0.001334	0.000049	0.000235
4	721	1.43e+07	0.209803	0.001379	0.000051	0.000245
5	724	1.43e+07	0.209888	0.001371	0.000051	0.000243
6	721	1.44e+07	0.209586	0.001413	0.000053	0.000251
7	744	1.49e+07	0.209607	0.001413	0.000052	0.000247

Errors and Test Paramters

Block	Acquisition Error (permil)	Shot-Noise (permil)	AE/SN ratio	Shapiro Wilk (p_value)	D'Agostino (p_value)
1	0.247	0.264	0.936	0.199	0.240
2	0.234	0.261	0.896	0.721	0.729
3	0.235	0.264	0.891	0.235	0.191
4	0.245	0.264	0.925	0.311	0.468
5	0.243	0.264	0.918	0.820	0.487
6	0.251	0.263	0.953	0.157	0.229
7	0.247	0.259	0.955	0.185	0.460

Isotopic Ratio and Errors of the Blocks

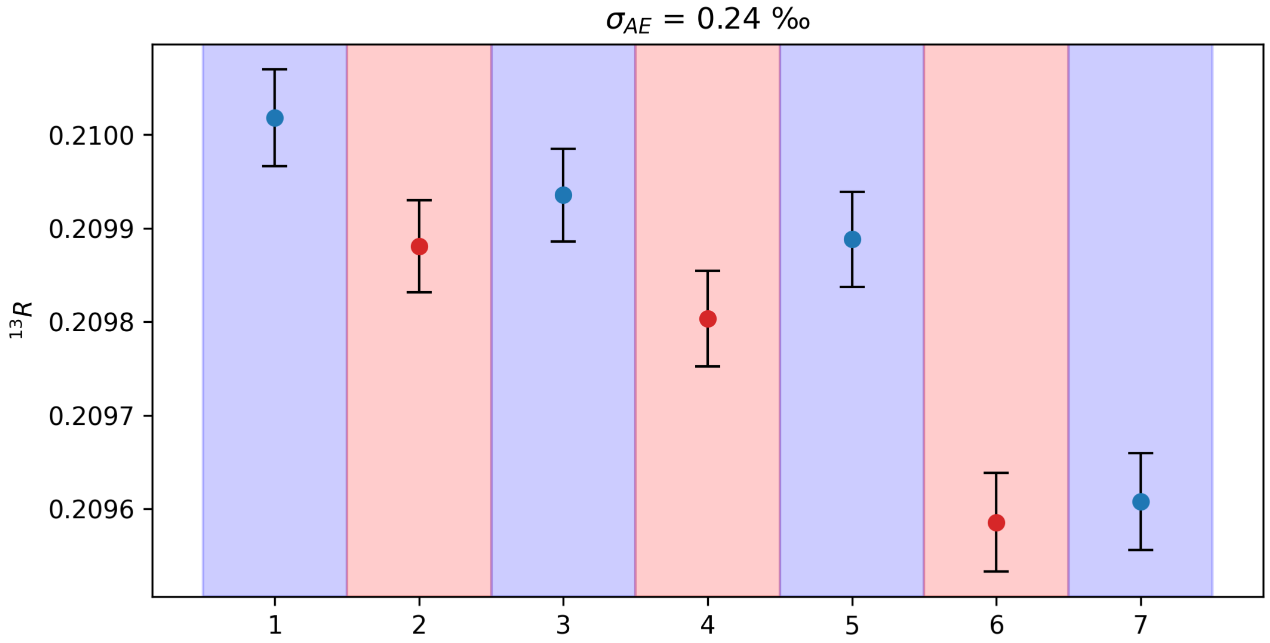

Cumulative Isotopic Ratio

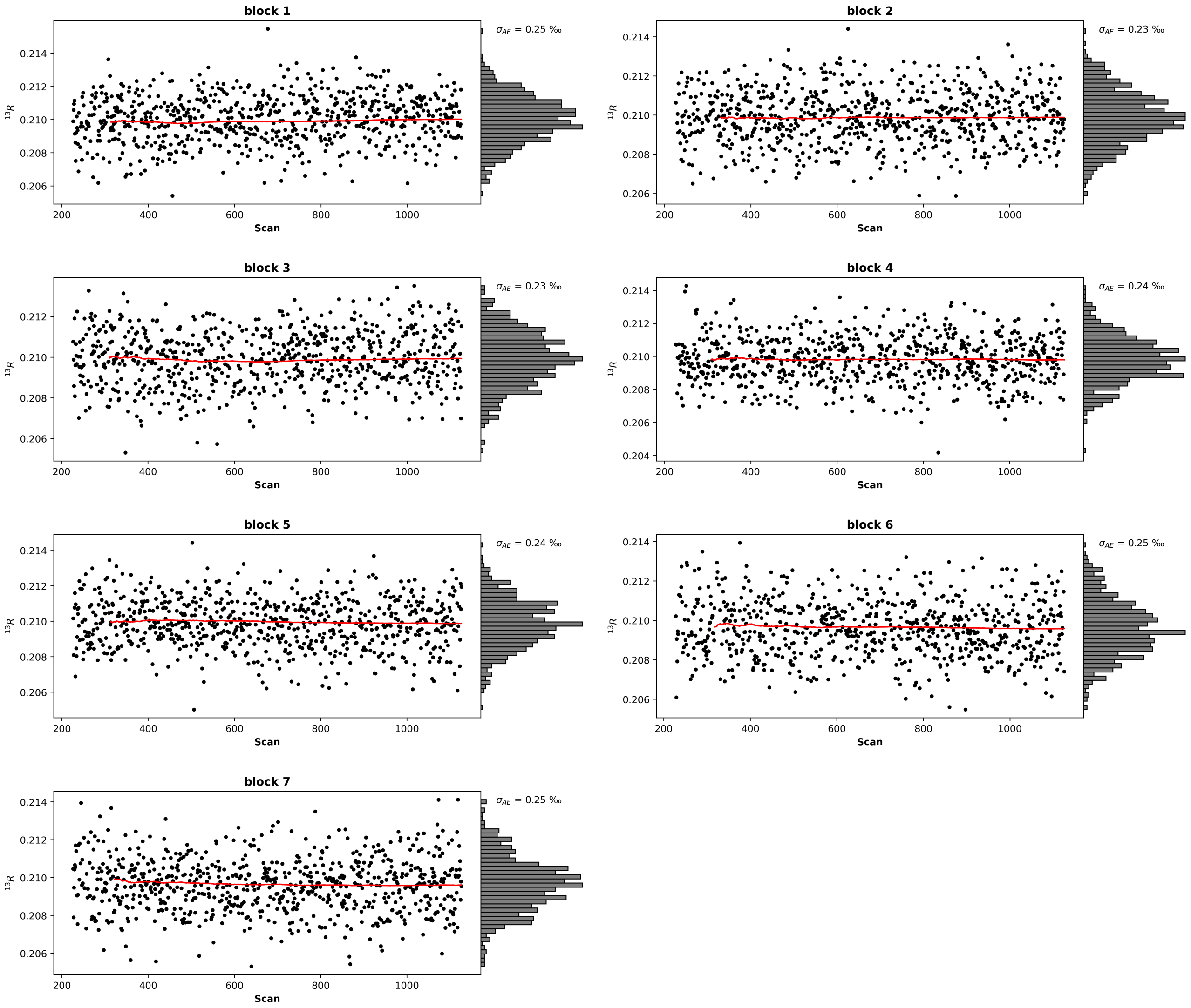

Acquisition Error and Shot-Noise

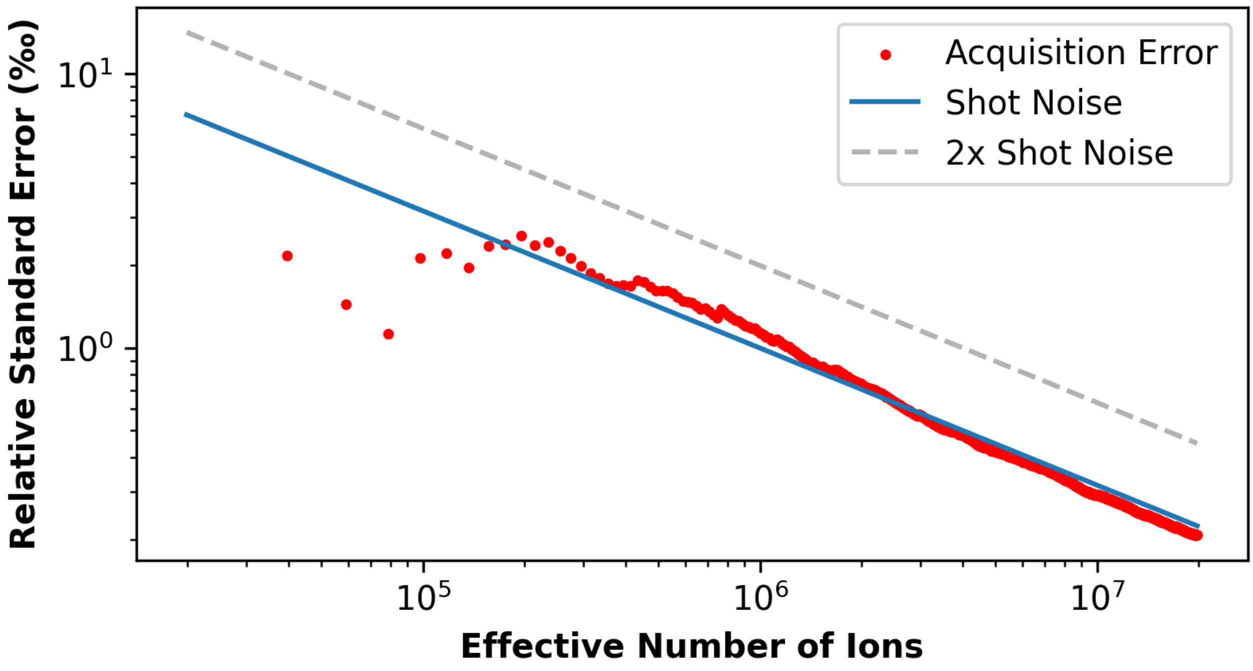

3. Delta Informations

Deltas were calculated by 'Average Of Neighboring Block Ratios'

3.1. 13C

Delta 13C was corrected by -27.80

Block	SEM	Delta corrected	Delta
2	0.23	-28.24	-0.46
4	0.24	-28.30	-0.52
6	0.25	-28.55	-0.77

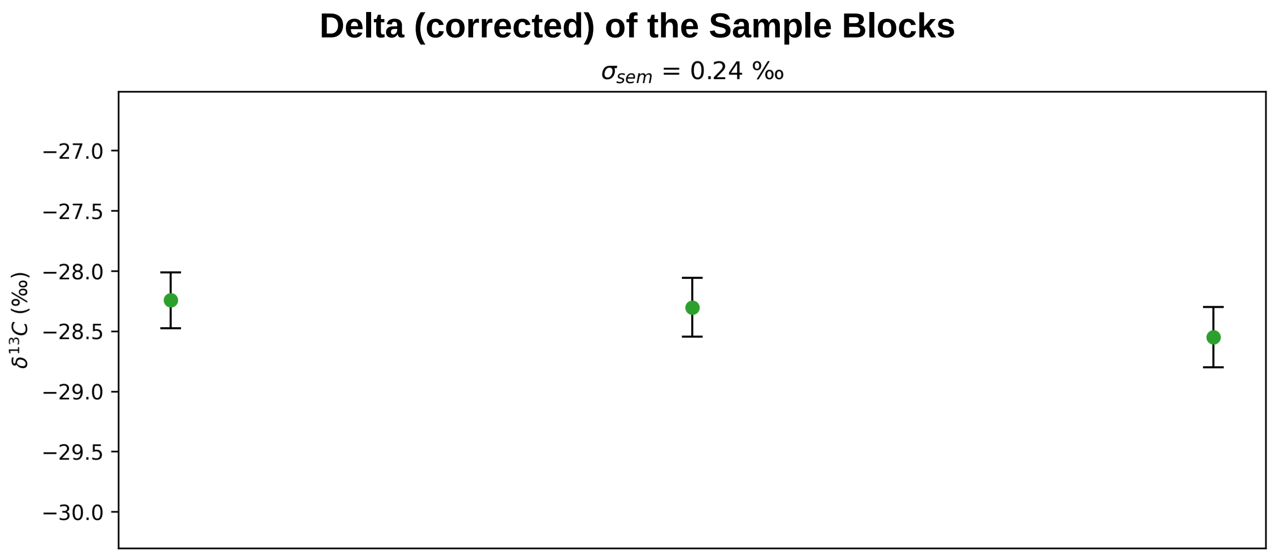

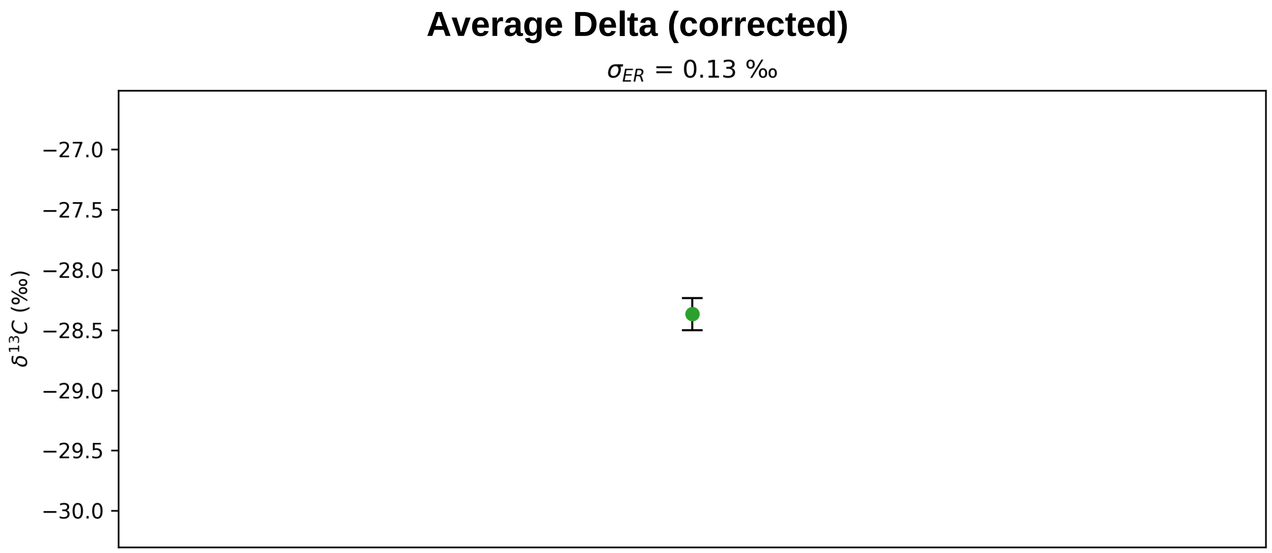

The final corrected average delta was -28.37 with a standard deviation of 0.13. Here the standard deviation is called reproducibility error.
