# Supplementary material for: Advancing Stable Isotope Analysis with Orbitrap-MS for Fatty Acid Methyl Esters and Complex Lipid Matrices
Source: J Am Soc Mass Spectrom. 2025 Jun 17;36(7):1527–35. doi: 10.1021/jasms.5c00092 (PMC12339014; doi:10.1021/jasms.5c00092)
Supplement: Supplementary file 2 [file js5c00092_si_002.zip › reports by IsotoPy Software/standards/H+Standard2_DI.pdf]

Standard 2 - [M + H]+
Isotope Analysis report from IsotoPy

Dual Inlet

1. Pre Processing

1.1. Block Time and Scan Information

Information about sample and standard block times and scans:

Block	Injected	Initial Time	End Time	Number of scans
1	standard	1	5	736
2	sample	6	10	752
3	standard	11	15	746
4	sample	16	20	751
5	standard	21	25	739
6	sample	26	30	770
7	standard	31	35	728

1.2. Outlier Removal

A total of 1081 scans were considered outliers and removed using the MAD method

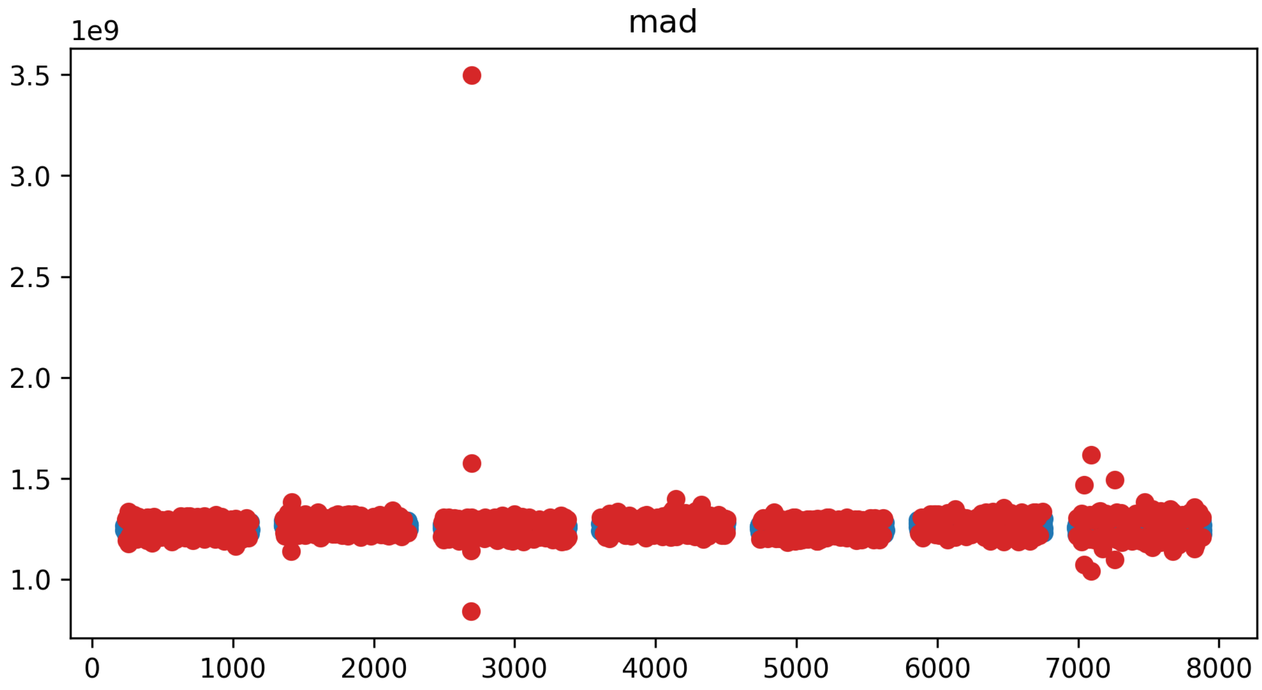

1.3. Total Ion Current (TIC)

TIC of all blocks

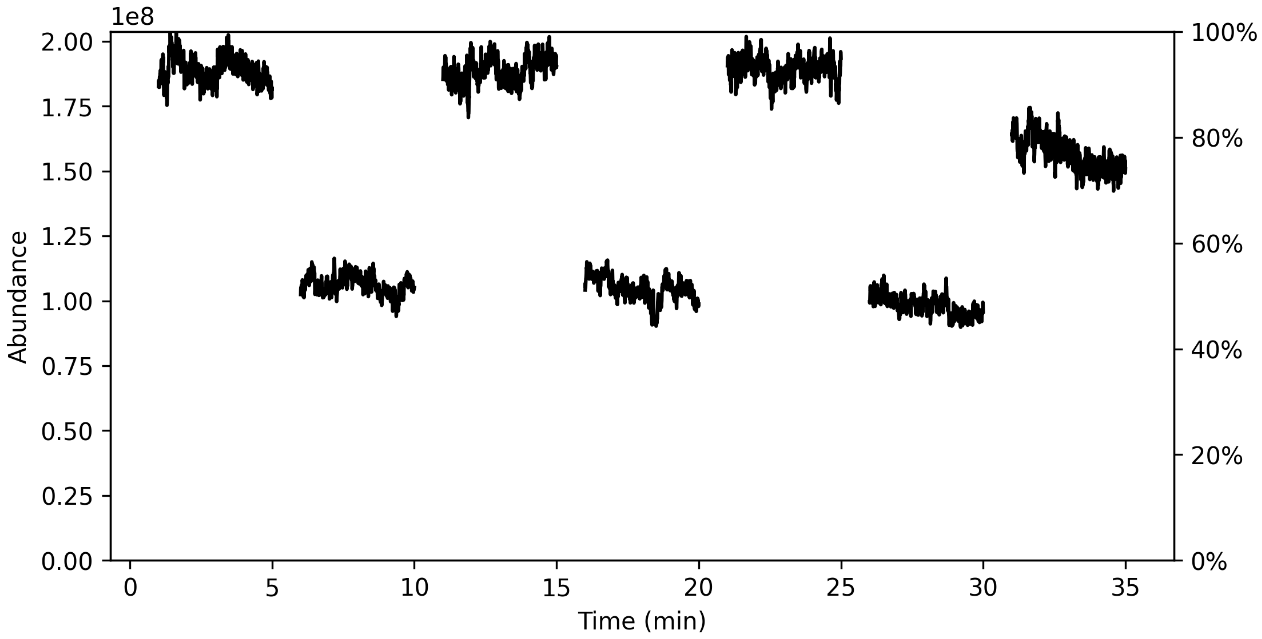

Block	TIC min	TIC max	TIC mean	RSD (%)
1	1.75e+08	2.04e+08	1.89e+08	2.62
2	9.40e+07	1.16e+08	1.06e+08	3.43
3	1.71e+08	2.02e+08	1.89e+08	2.73
4	9.03e+07	1.16e+08	1.05e+08	4.54
5	1.74e+08	2.02e+08	1.89e+08	2.33
6	8.98e+07	1.10e+08	9.83e+07	3.89
7	1.42e+08	1.74e+08	1.57e+08	4.24

2. Block Parameters

The Isotopic Ratio of the blocks were calculated by 'Mean'

2.1. 13C/M0

Block	Number of scans	Effective number of ions	Isotopic Ratio	STD	SEM	RSE
1	736	1.36e+07	0.216201	0.001384	0.000051	0.000236
2	752	1.37e+07	0.215497	0.001320	0.000048	0.000223
3	746	1.38e+07	0.216120	0.001404	0.000051	0.000238
4	751	1.37e+07	0.215661	0.001391	0.000051	0.000235
5	739	1.36e+07	0.216215	0.001354	0.000050	0.000230
6	770	1.41e+07	0.215687	0.001364	0.000049	0.000228
7	728	1.34e+07	0.216404	0.001368	0.000051	0.000234

Errors and Test Paramters

Block	Acquisition Error (permil)	Shot-Noise (permil)	AE/SN ratio	Shapiro Wilk (p_value)	D'Agostino (p_value)
1	0.236	0.271	0.868	0.709	0.378
2	0.223	0.270	0.827	0.933	0.849
3	0.238	0.270	0.882	0.537	0.500
4	0.235	0.270	0.871	0.554	0.355
5	0.230	0.271	0.849	0.023	0.009
6	0.228	0.267	0.854	0.698	0.838
7	0.234	0.273	0.857	0.479	0.428

Isotopic Ratio and Errors of the Blocks

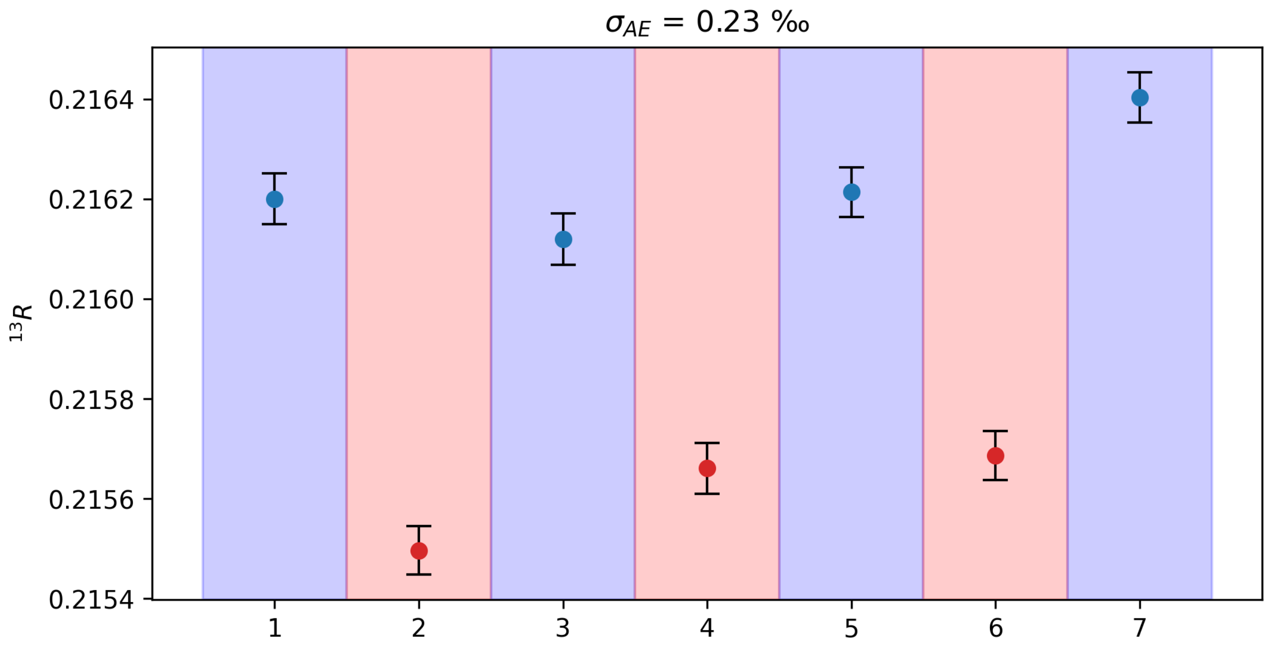

Cumulative Isotopic Ratio

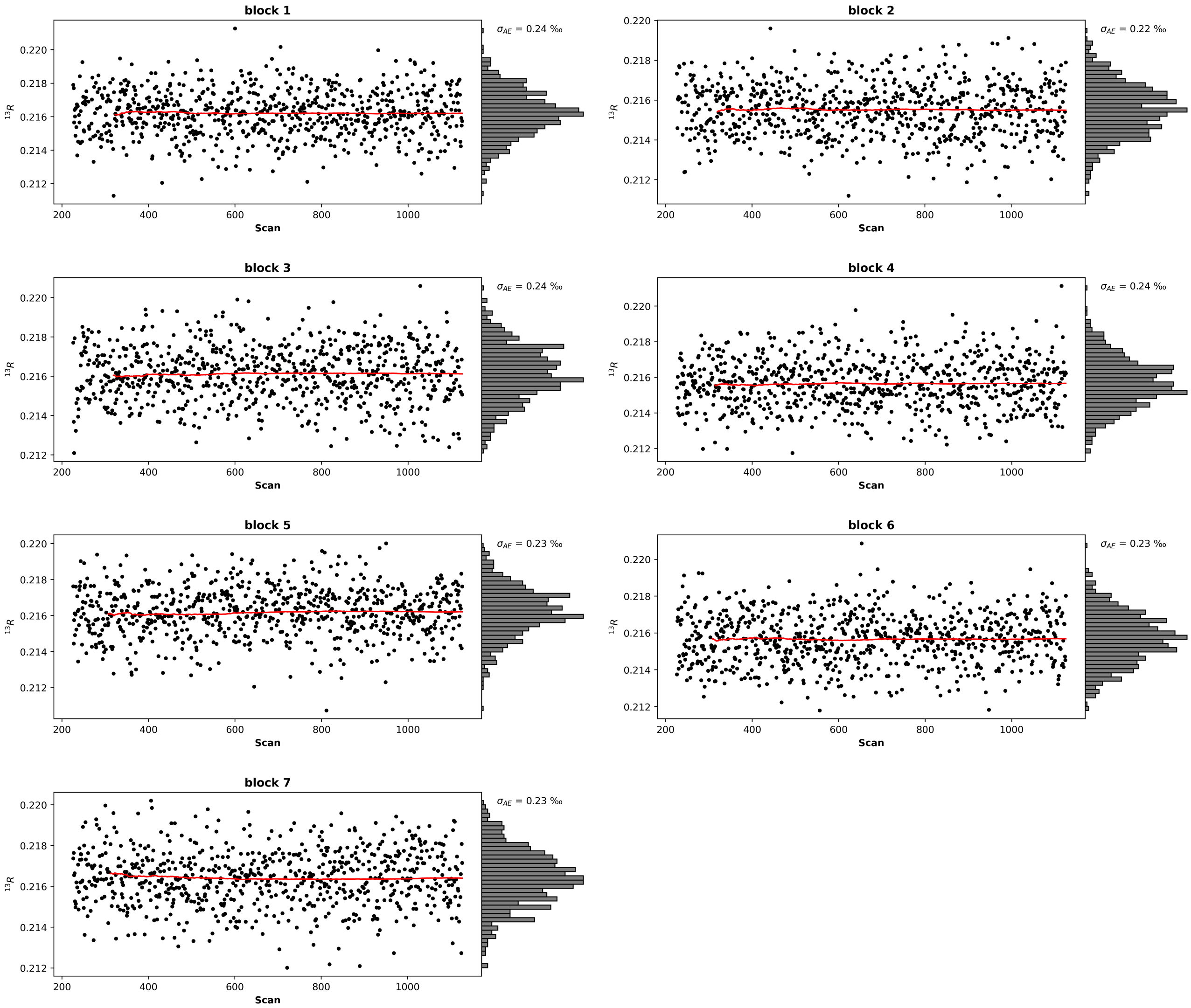

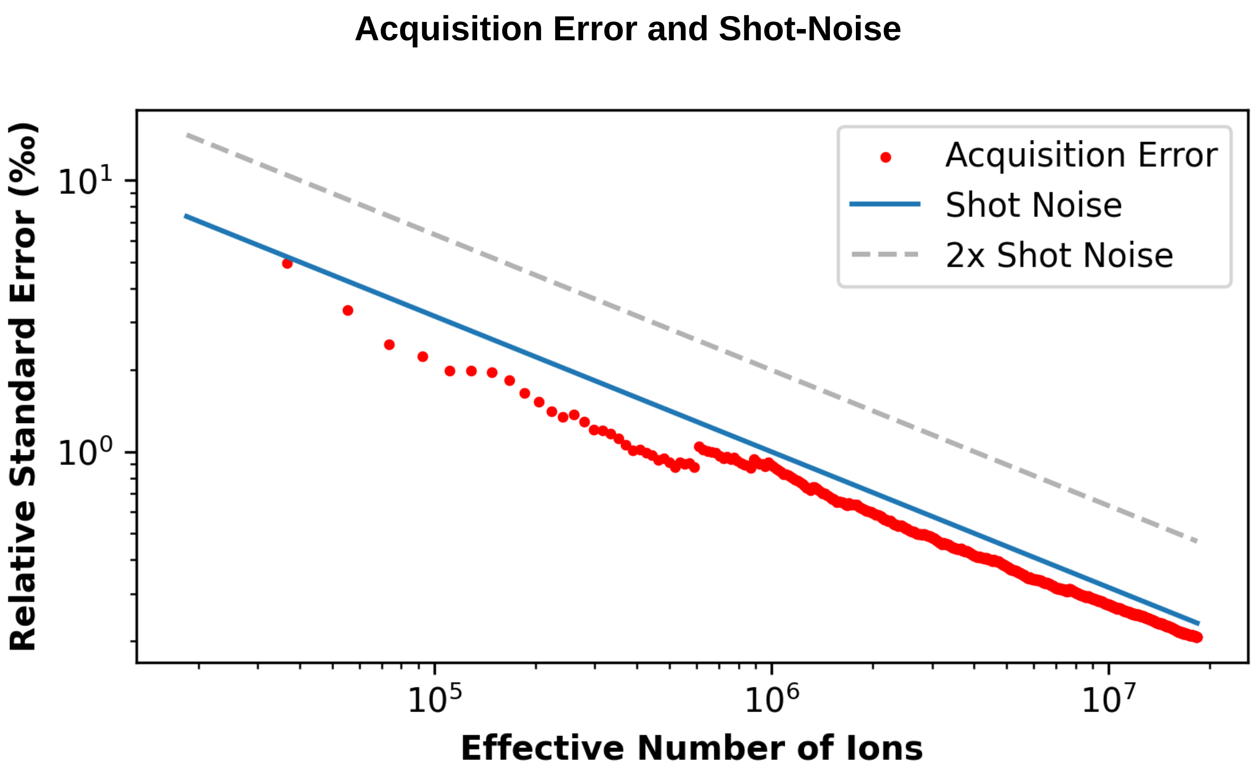
Acquisition Error and Shot-Noise
Acquisition Error
Shot Noise
2x Shot Noise
Relative Standard Error (‰)
10^1
10^0
10^5
10^6
10^7
Effective Number of Ions

3. Delta Informations

Deltas were calculated by 'Average Of Neighboring Block Ratios'

3.1. 13C

Delta 13C was corrected by -27.80

Block	SEM	Delta corrected	Delta
2	0.22	-30.79	-3.07
4	0.23	-30.08	-2.34
6	0.23	-30.60	-2.88

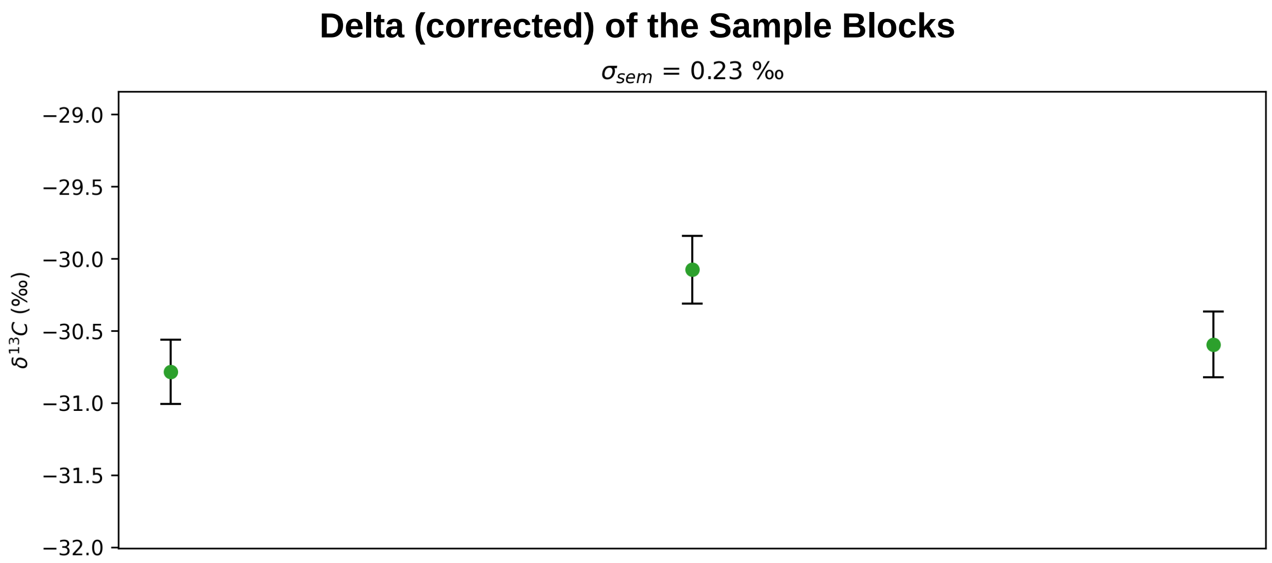

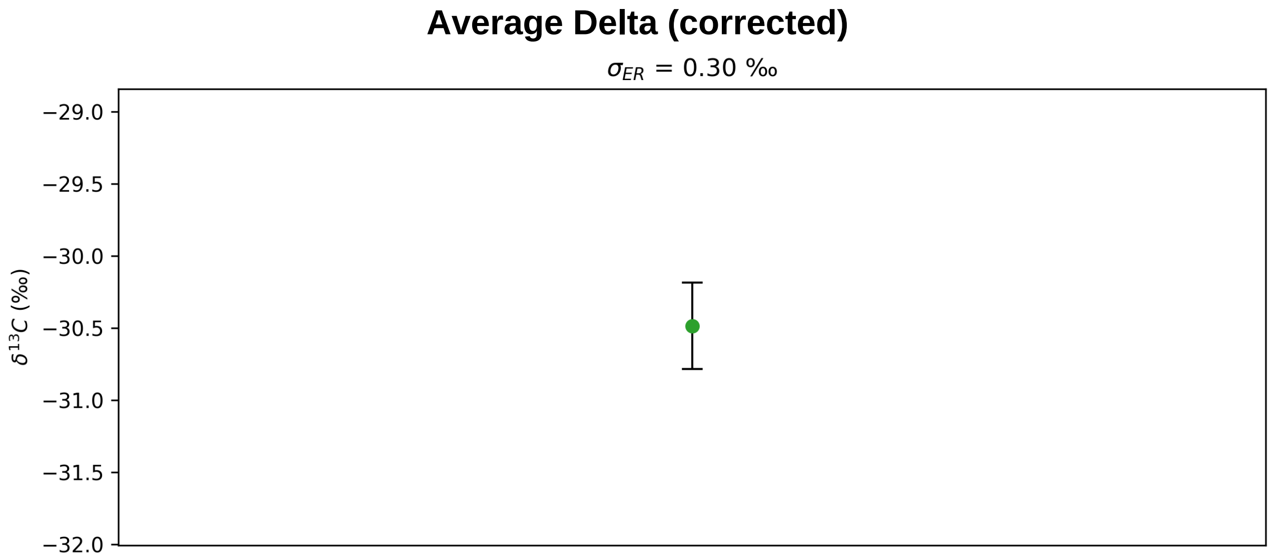

The final corrected average delta was -30.49 with a standard deviation of 0.30. Here the standard deviation is called reproducibility error.
