# Supplementary material for: Advancing Stable Isotope Analysis with Orbitrap-MS for Fatty Acid Methyl Esters and Complex Lipid Matrices
Source: J Am Soc Mass Spectrom. 2025 Jun 17;36(7):1527–35. doi: 10.1021/jasms.5c00092 (PMC12339014; doi:10.1021/jasms.5c00092)
Supplement: Supplementary file 2 [file js5c00092_si_002.zip › reports by IsotoPy Software/standards/H+Standard9_FI.pdf]

Standard 9 - [M + H]+

Isotope Analysis report from IsotoPy

Flow Injection

1. Pre Processing

1.1. Block Time and Scan Information

Information about sample and standard block times and scans:

Block	Injected	Initial Time	End Time	Number of scans
1	standard	1	8	1340
2	sample	16	23	1247
3	standard	31	38	1280
4	sample	46	53	1290
5	standard	61	68	1287
6	sample	76	83	1316
7	standard	91	98	1289

1.2. Outlier Removal

A total of 1990 scans were considered outliers and removed using the MAD method

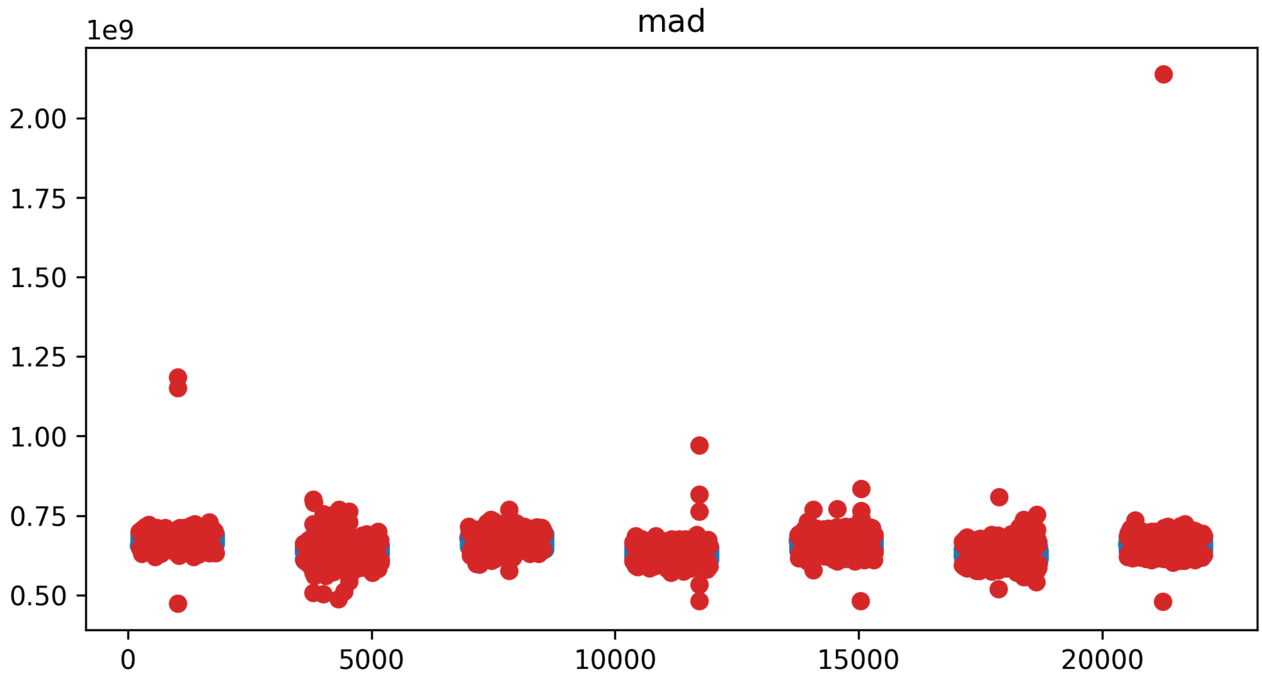

1.3. Total Ion Current (TIC)

TIC of all blocks

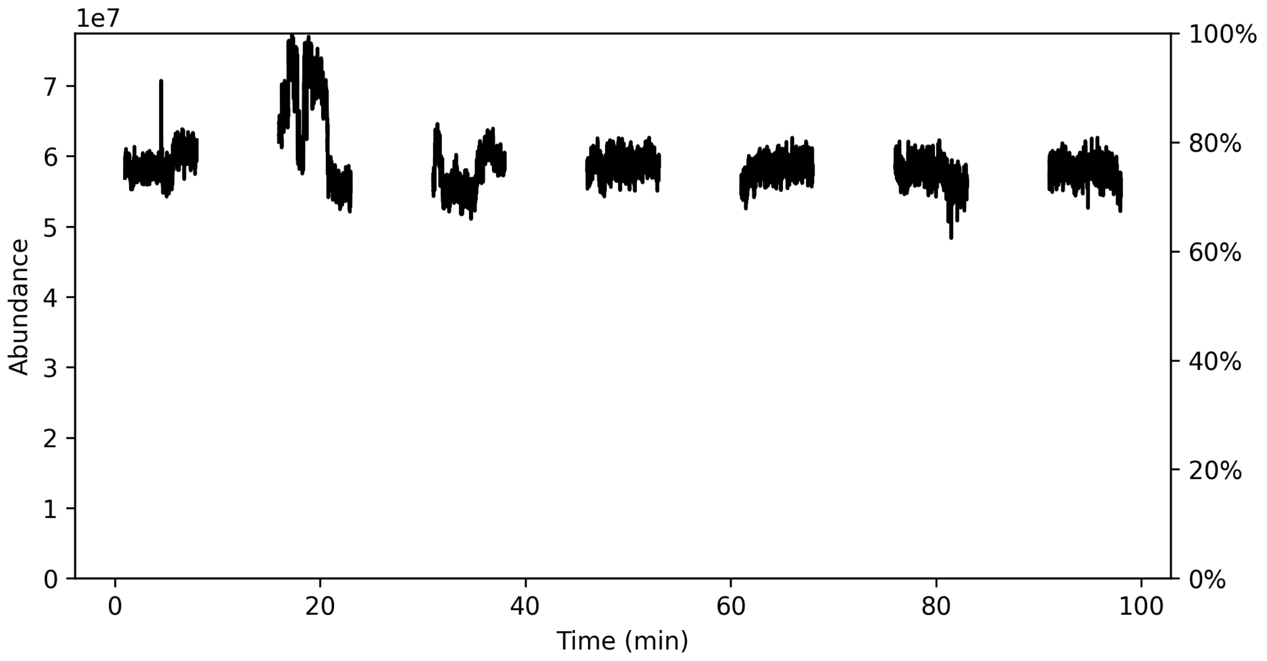

Block	TIC min	TIC max	TIC mean	RSD (%)
1	5.42e+07	7.07e+07	5.90e+07	2.87
2	5.21e+07	7.75e+07	6.47e+07	11.13
3	5.11e+07	6.46e+07	5.76e+07	4.83
4	5.43e+07	6.26e+07	5.87e+07	2.27
5	5.25e+07	6.26e+07	5.82e+07	2.48
6	4.84e+07	6.23e+07	5.76e+07	2.97
7	5.22e+07	6.26e+07	5.79e+07	2.52

2. Block Parameters

The Isotopic Ratio of the blocks were calculated by 'Mean'

2.1. 13C/M0

Block	Number of scans	Effective number of ions	Isotopic Ratio	STD	SEM	RSE
1	1340	1.80e+07	0.209801	0.001749	0.000048	0.000228
2	1247	1.67e+07	0.209799	0.001785	0.000051	0.000241
3	1280	1.72e+07	0.209955	0.001751	0.000049	0.000233
4	1290	1.73e+07	0.210092	0.001766	0.000049	0.000234
5	1287	1.73e+07	0.210040	0.001764	0.000049	0.000234
6	1316	1.77e+07	0.210256	0.001849	0.000051	0.000242
7	1289	1.74e+07	0.210172	0.001696	0.000047	0.000225

Errors and Test Paramters

Block	Acquisition Error (permil)	Shot-Noise (permil)	AE/SN ratio	Shapiro Wilk (p_value)	D'Agostino (p_value)
1	0.228	0.236	0.966	0.538	0.516
2	0.241	0.245	0.984	0.503	0.674
3	0.233	0.241	0.967	0.244	0.428
4	0.234	0.241	0.972	0.562	0.945
5	0.234	0.241	0.973	0.444	0.820
6	0.242	0.238	1.018	0.506	0.594
7	0.225	0.240	0.936	0.009	0.005

Isotopic Ratio and Errors of the Blocks

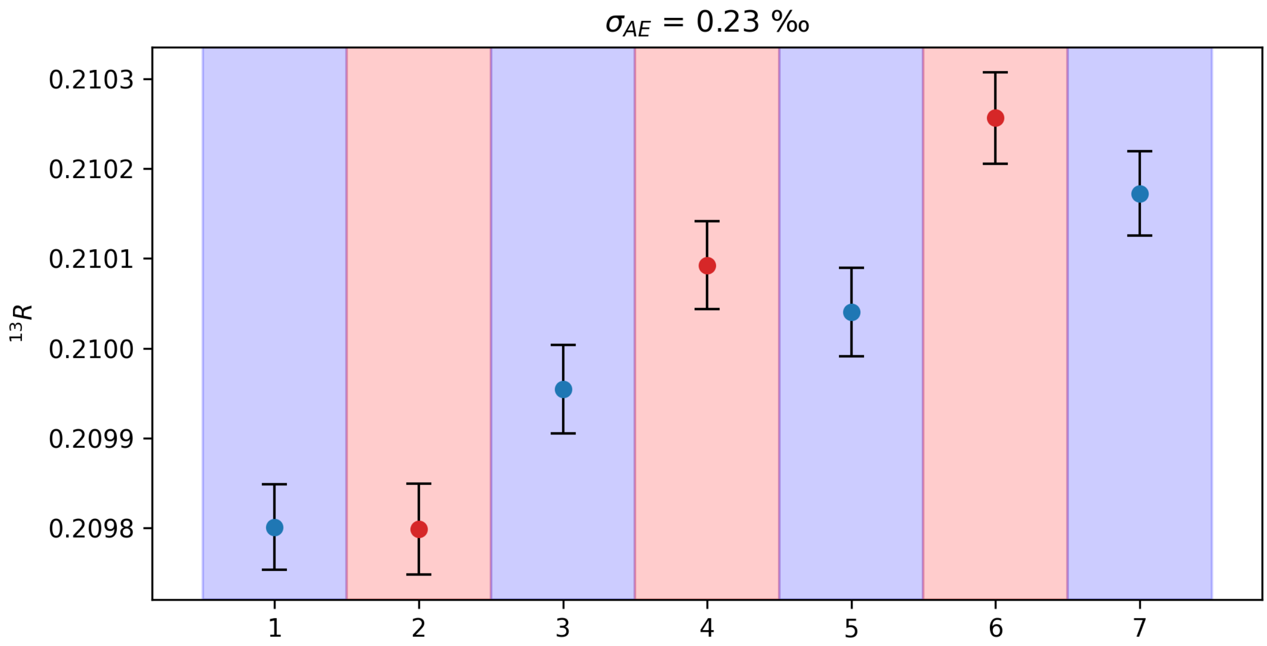

Cumulative Isotopic Ratio

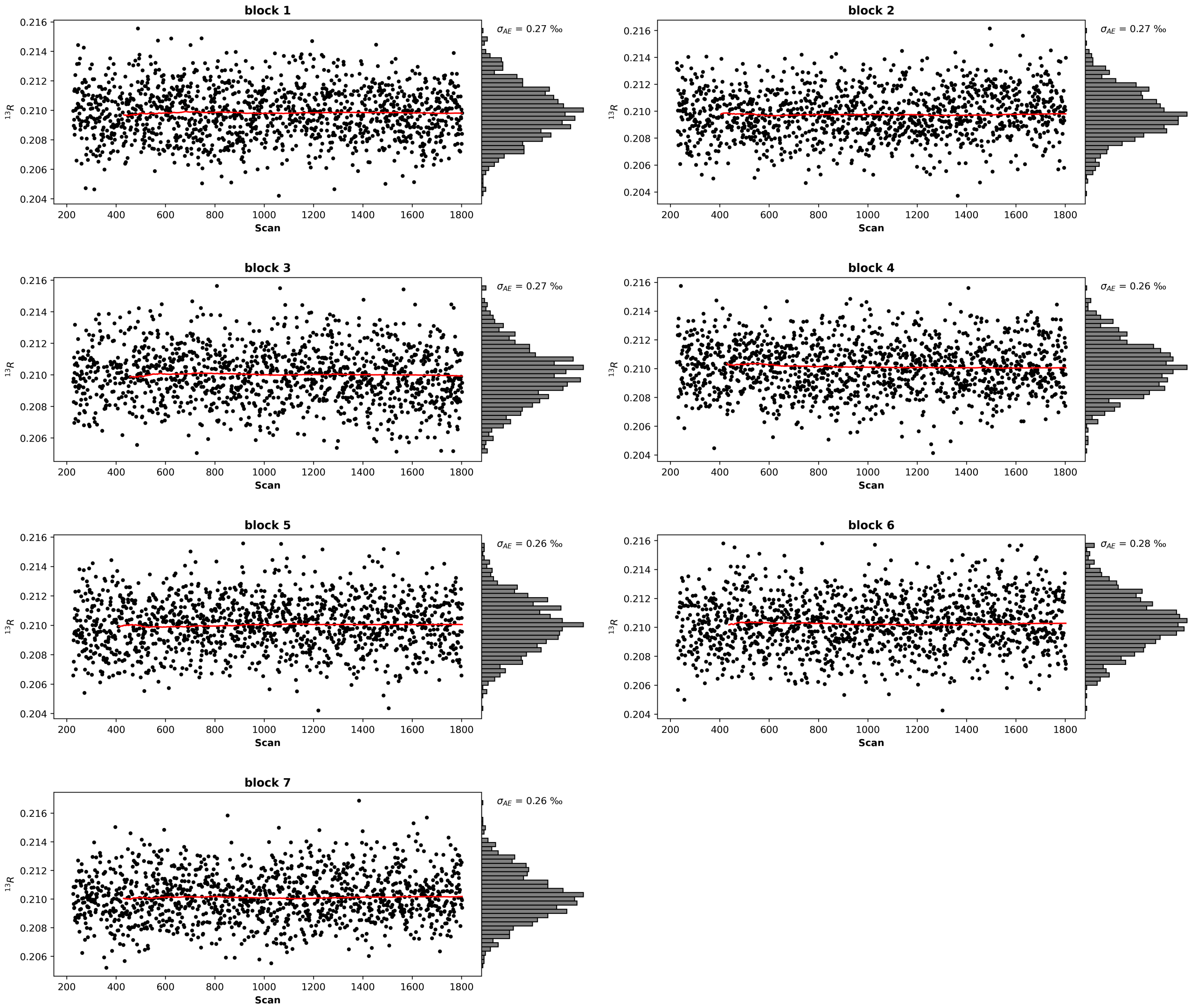

Acquisition Error and Shot-Noise

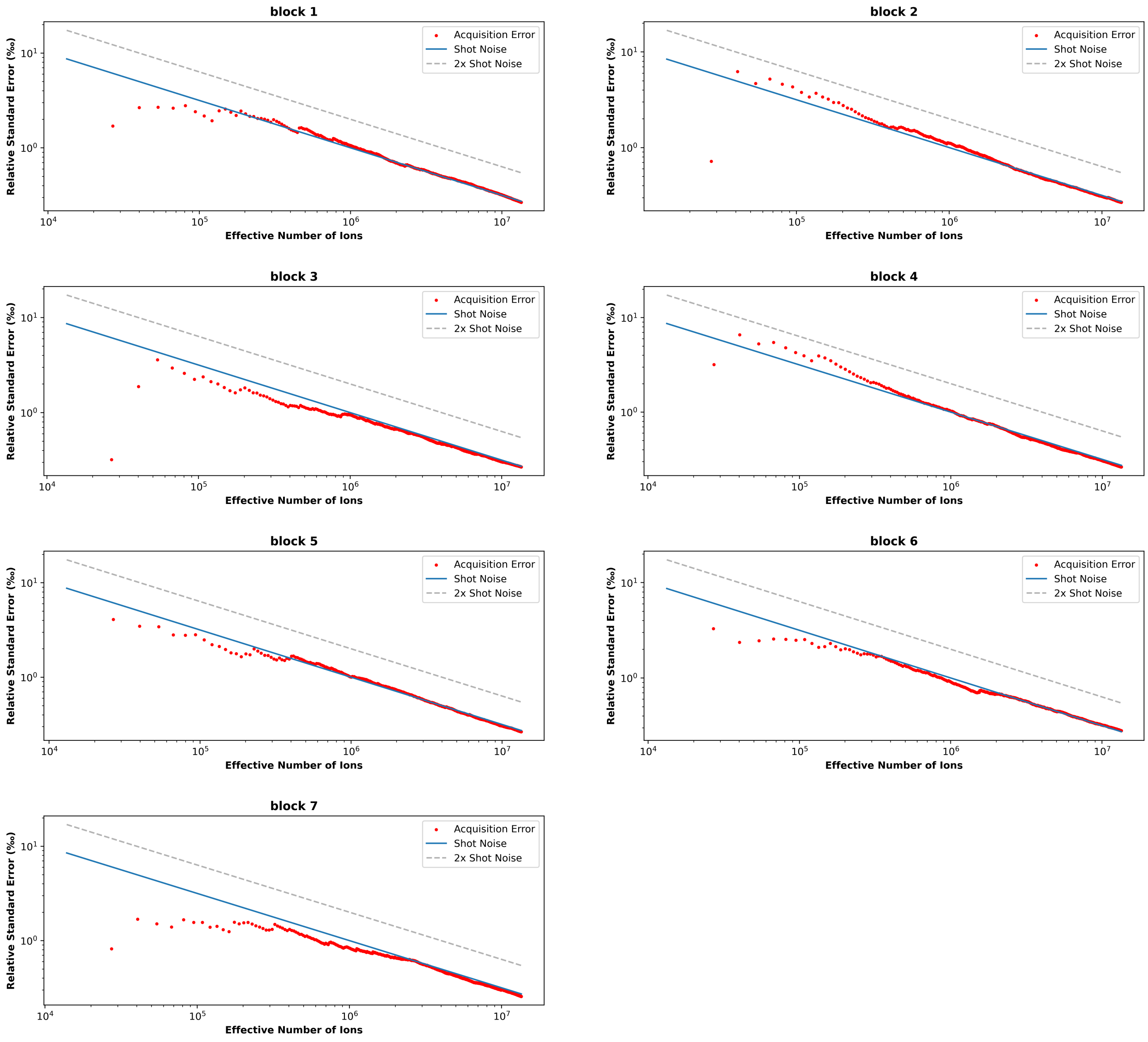

3. Delta Informations

Deltas were calculated by 'Average Of Neighboring Block Ratios'

3.1. 13C

Delta 13C was corrected by -27.80

Block	SEM	Delta corrected	Delta
2	0.24	-28.17	-0.38
4	0.23	-27.36	0.45
6	0.24	-27.11	0.71

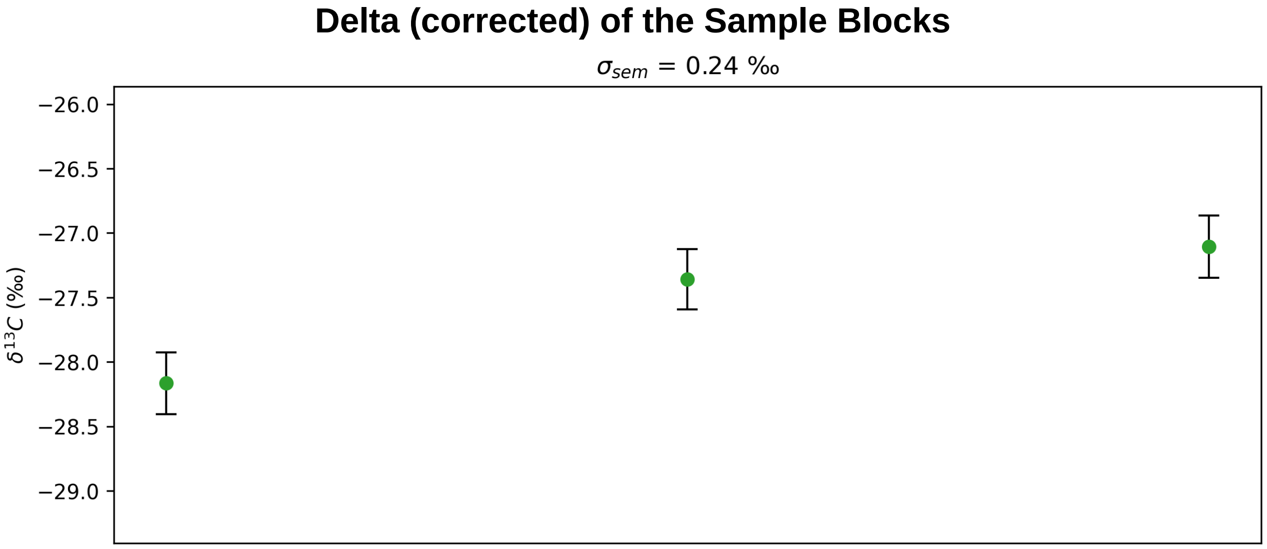

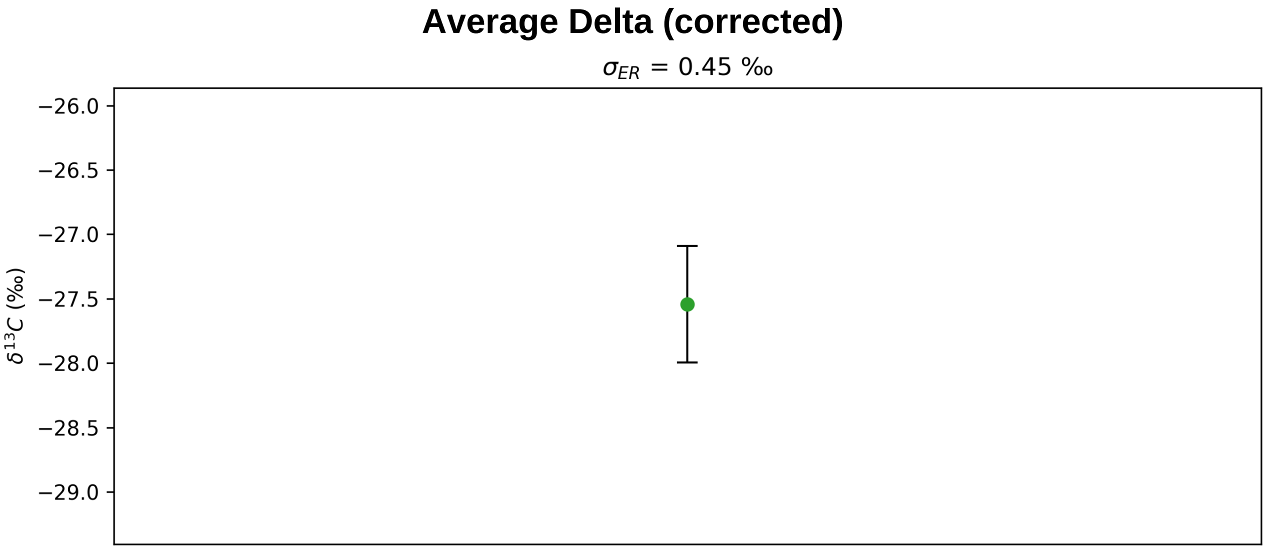

The final corrected average delta was -27.54 with a standard deviation of 0.45. Here the standard deviation is called reproducibility error.
