# Supplementary material for: Advancing Stable Isotope Analysis with Orbitrap-MS for Fatty Acid Methyl Esters and Complex Lipid Matrices
Source: J Am Soc Mass Spectrom. 2025 Jun 17;36(7):1527–35. doi: 10.1021/jasms.5c00092 (PMC12339014; doi:10.1021/jasms.5c00092)
Supplement: Supplementary file 2 [file js5c00092_si_002.zip › reports by IsotoPy Software/standards/H+Standard5_FI.pdf]

Standard 5 - [M + H]+
Isotope Analysis report from IsotoPy

Flow Injection

1. Pre Processing

1.1. Block Time and Scan Information

Information about sample and standard block times and scans:

Block	Injected	Initial Time	End Time	Number of scans
1	standard	1	8	1271
2	sample	16	23	1275
3	standard	31	38	1291
4	sample	46	53	1315
5	standard	61	68	1281
6	sample	76	83	1311
7	standard	91	98	1276

1.2. Outlier Removal

A total of 2019 scans were considered outliers and removed using the MAD method

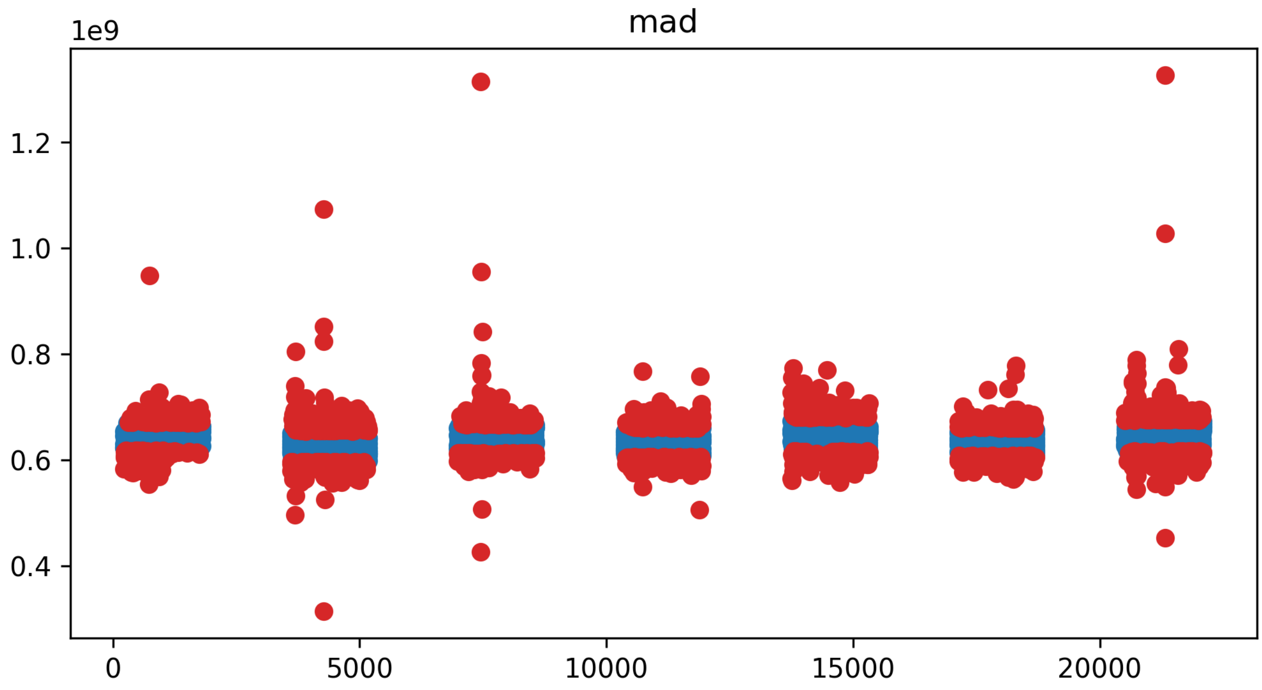

1.3. Total Ion Current (TIC)

TIC of all blocks

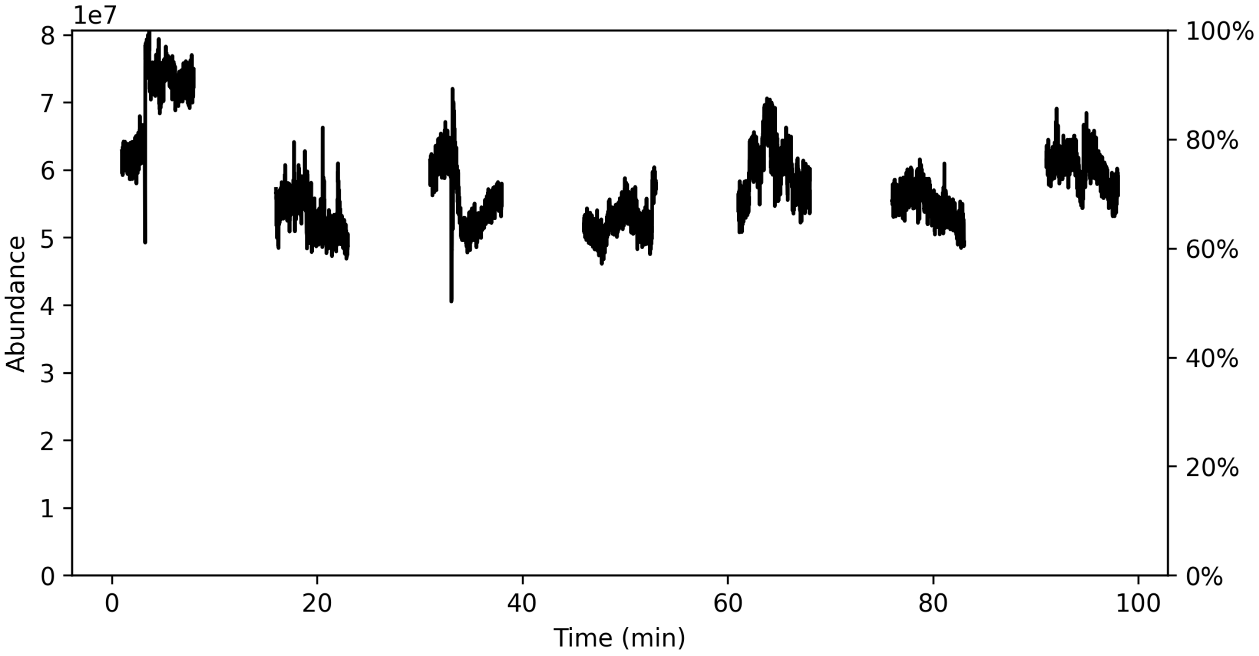

Block	TIC min	TIC max	TIC mean	RSD (%)
1	4.92e+07	8.07e+07	7.06e+07	7.88
2	4.69e+07	6.63e+07	5.33e+07	5.57
3	4.05e+07	7.20e+07	5.60e+07	8.17
4	4.61e+07	6.04e+07	5.24e+07	4.40
5	5.08e+07	7.06e+07	6.02e+07	6.78
6	4.84e+07	6.16e+07	5.50e+07	4.17
7	5.31e+07	6.91e+07	6.02e+07	4.23

2. Block Parameters

The Isotopic Ratio of the blocks were calculated by 'Mean'

2.1. 13C/M0

Block	Number of scans	Effective number of ions	Isotopic Ratio	STD	SEM	RSE
1	1271	1.70e+07	0.209452	0.001818	0.000051	0.000243
2	1275	1.69e+07	0.210117	0.001788	0.000050	0.000238
3	1291	1.73e+07	0.209975	0.001745	0.000049	0.000231
4	1315	1.74e+07	0.209979	0.001723	0.000048	0.000226
5	1281	1.72e+07	0.209939	0.001726	0.000048	0.000230
6	1311	1.74e+07	0.209979	0.001728	0.000048	0.000227
7	1276	1.72e+07	0.210035	0.001697	0.000048	0.000226

Errors and Test Paramters

Block	Acquisition Error (permil)	Shot-Noise (permil)	AE/SN ratio	Shapiro Wilk (p_value)	D'Agostino (p_value)
1	0.243	0.243	1.003	0.005	0.135
2	0.238	0.243	0.981	0.300	0.835
3	0.231	0.241	0.961	0.559	0.646
4	0.226	0.239	0.945	0.095	0.139
5	0.230	0.241	0.952	0.842	0.902
6	0.227	0.240	0.949	0.112	0.052
7	0.226	0.241	0.937	0.621	0.668

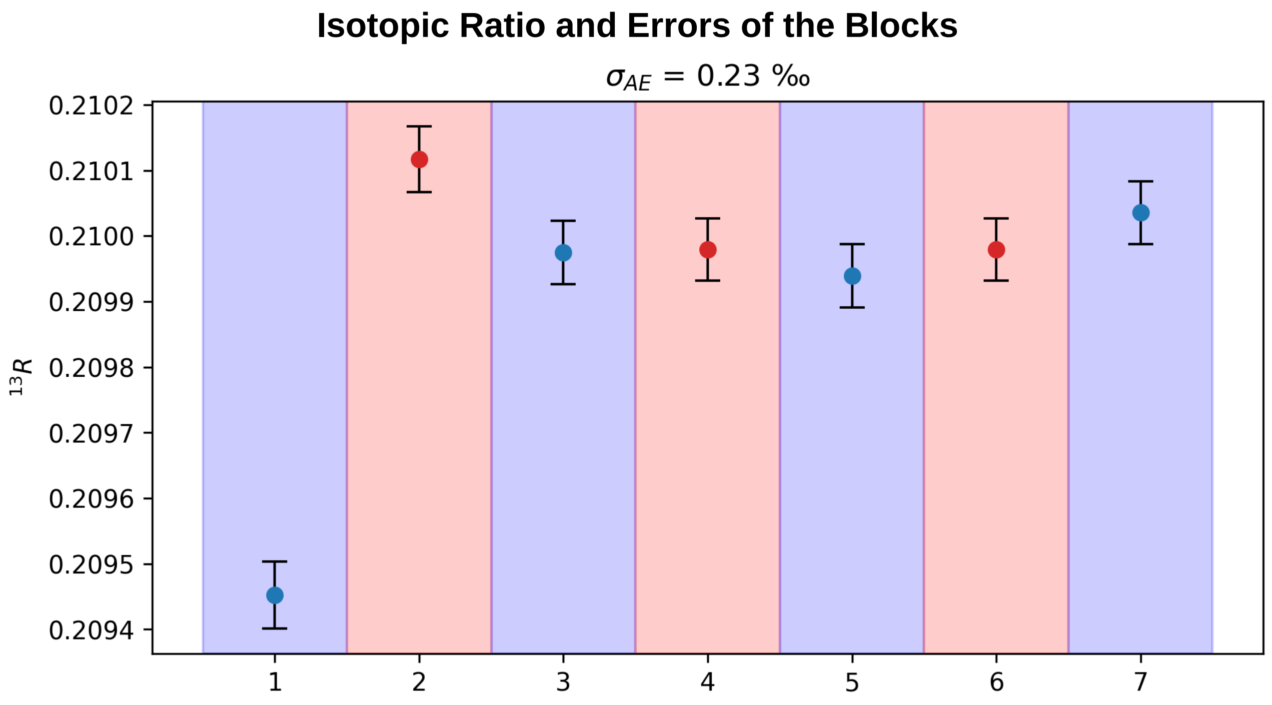

Isotopic Ratio and Errors of the Blocks
σ_{AE} = 0.23 ‰
^{13}R
0.2102
0.2101
0.2100
0.2099
0.2098
0.2097
0.2096
0.2095
0.2094
1
2
3
4
5
6
7

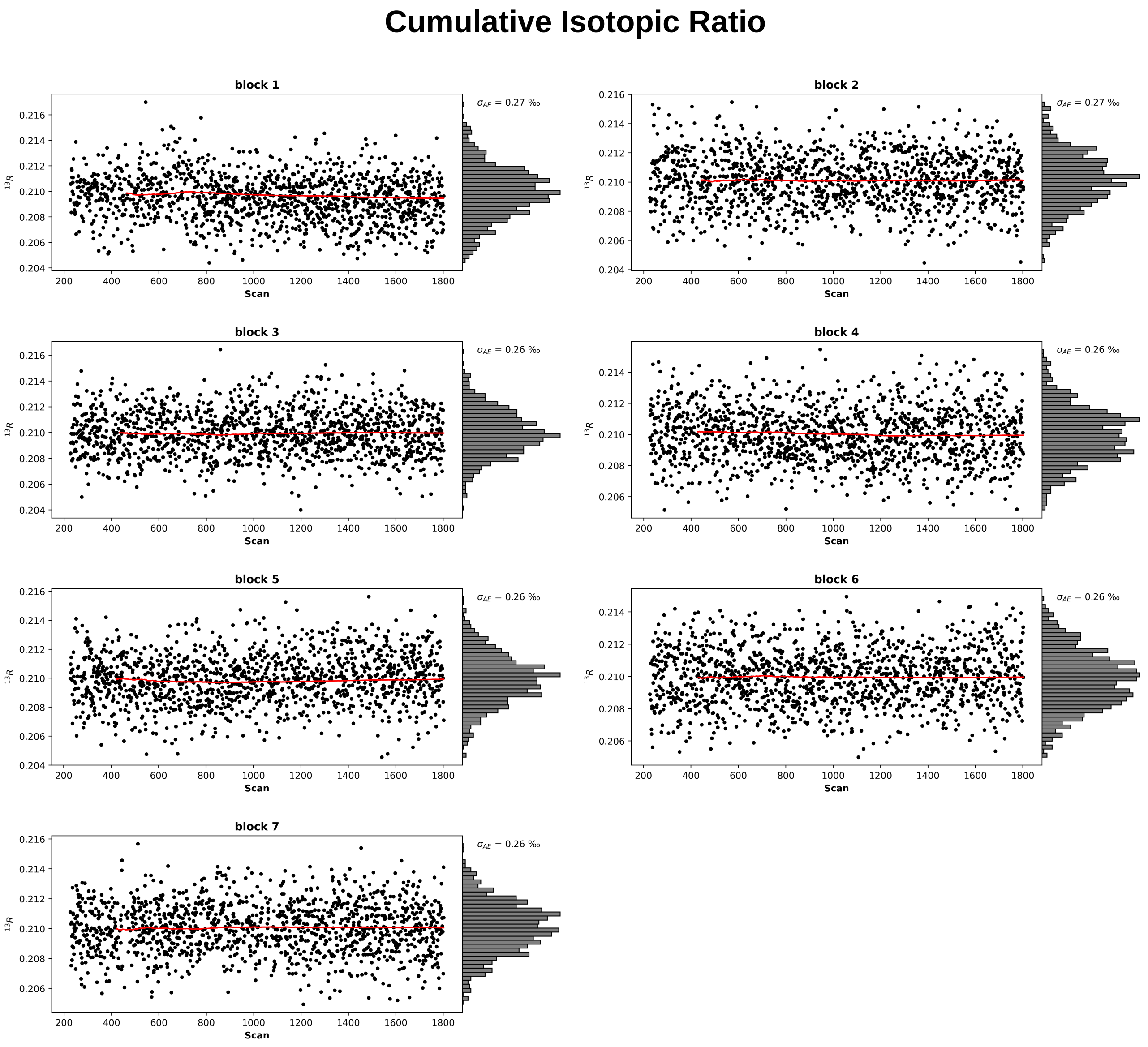

Cumulative Isotopic Ratio
block 1
σ_{AE} = 0.27 ‰
block 2
σ_{AE} = 0.27 ‰
block 3
σ_{AE} = 0.26 ‰
block 4
σ_{AE} = 0.26 ‰
block 5
σ_{AE} = 0.26 ‰
block 6
σ_{AE} = 0.26 ‰
block 7
σ_{AE} = 0.26 ‰
^{13}R
Scan

Acquisition Error and Shot-Noise

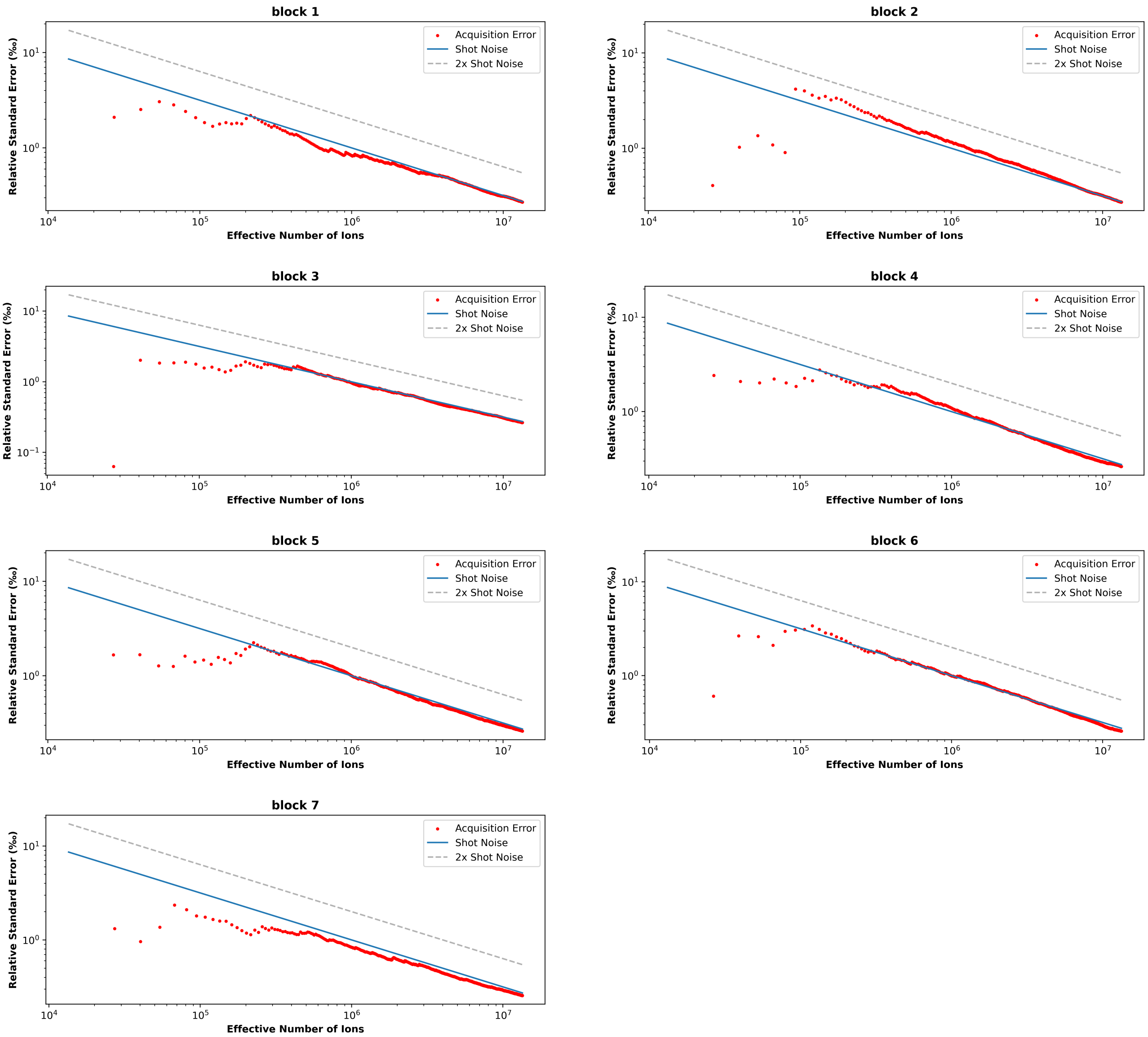

3. Delta Informations

Deltas were calculated by 'Average Of Neighboring Block Ratios'

3.1. 13C

Delta 13C was corrected by -27.80

Block	SEM	Delta corrected	Delta
2	0.24	-25.93	1.92
4	0.23	-27.70	0.11
6	0.23	-27.84	-0.04

Delta (corrected) of the Sample Blocks

σ_{sem} = 0.23 ‰

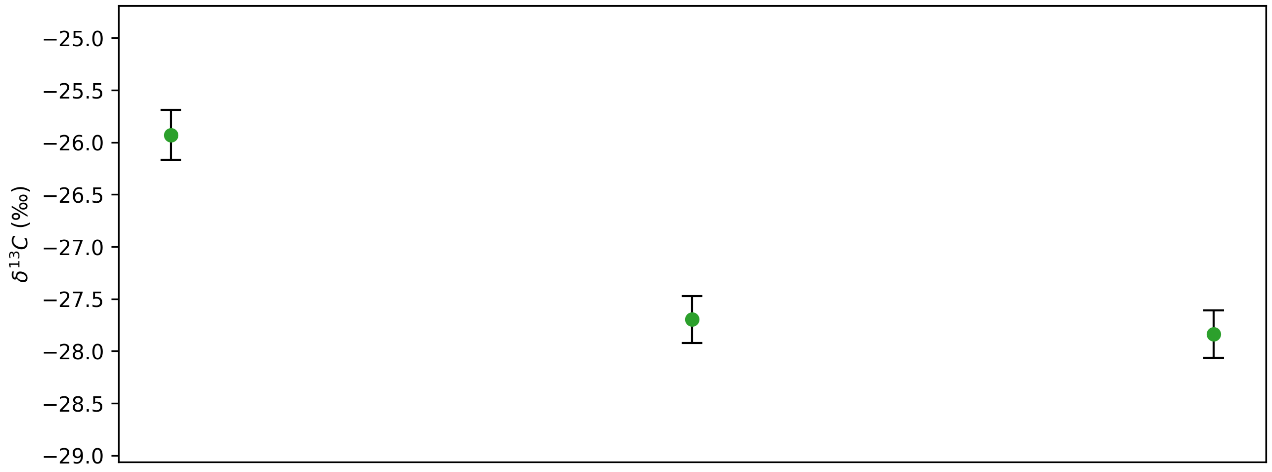

Average Delta (corrected)

σ_{ER} = 0.87 ‰

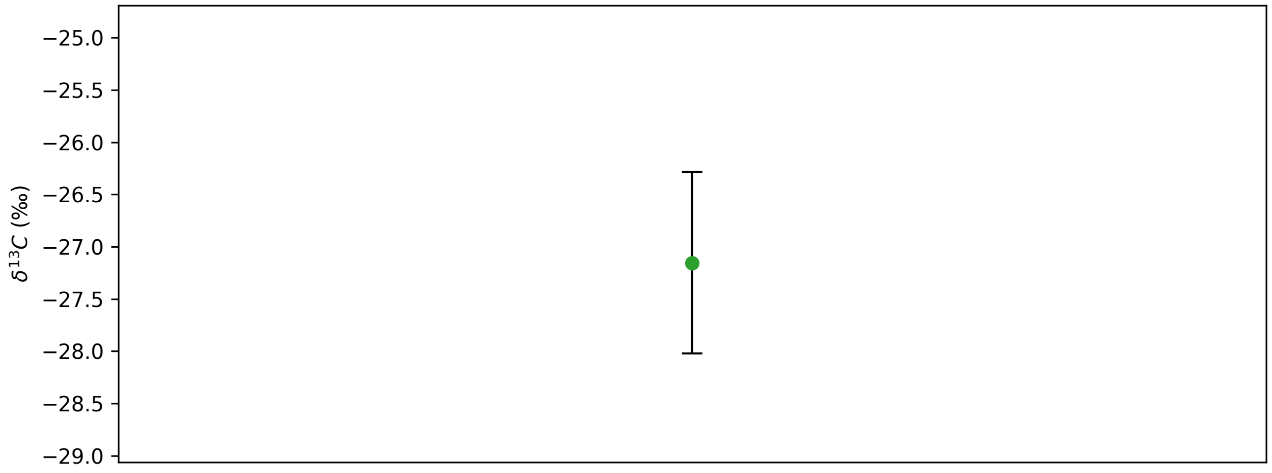

The final corrected average delta was -27.15 with a standard deviation of 0.87. Here the standard deviation is called reproducibility error.
