# Supplementary material for: Advancing Stable Isotope Analysis with Orbitrap-MS for Fatty Acid Methyl Esters and Complex Lipid Matrices
Source: J Am Soc Mass Spectrom. 2025 Jun 17;36(7):1527–35. doi: 10.1021/jasms.5c00092 (PMC12339014; doi:10.1021/jasms.5c00092)
Supplement: Supplementary file 2 [file js5c00092_si_002.zip › reports by IsotoPy Software/standards/H+Standard7_DI.pdf]

Standard 7 - [M + H]+
Isotope Analysis report from IsotoPy
Dual Inlet

1. Pre Processing

1.1. Block Time and Scan Information

Information about sample and standard block times and scans:

Block	Injected	Initial Time	End Time	Number of scans
1	standard	1	5	723
2	sample	6	10	742
3	standard	11	15	746
4	sample	16	20	718
5	standard	21	25	706
6	sample	26	30	747
7	standard	31	35	747

1.2. Outlier Removal

A total of 1179 scans were considered outliers and removed using the MAD method

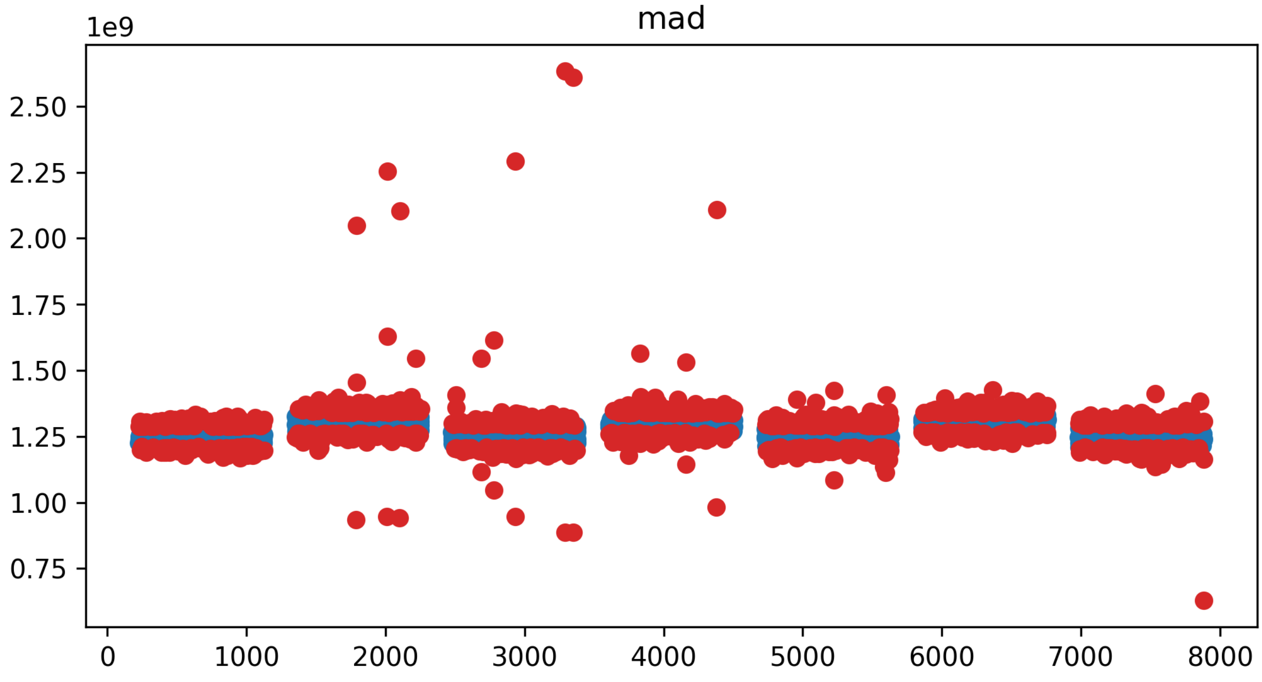

1.3. Total Ion Current (TIC)

TIC of all blocks

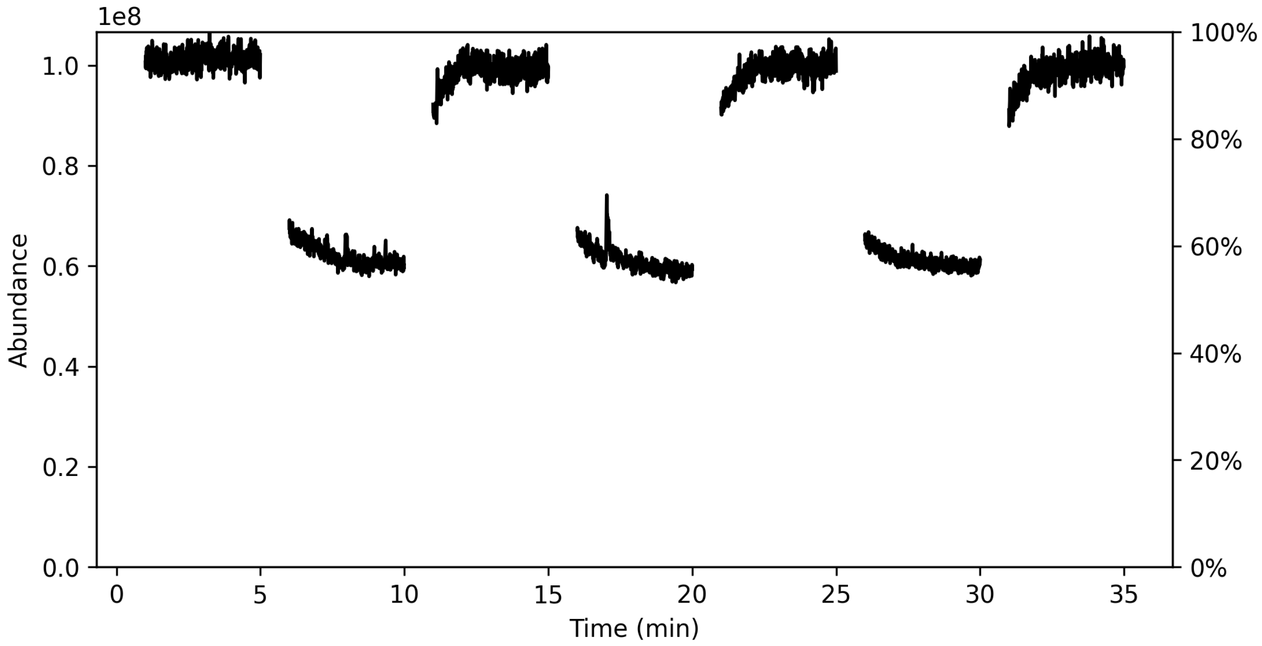

Block	TIC min	TIC max	TIC mean	RSD (%)
1	9.65e+07	1.07e+08	1.01e+08	1.55
2	5.79e+07	6.92e+07	6.22e+07	3.63
3	8.84e+07	1.04e+08	9.87e+07	2.50
4	5.67e+07	7.41e+07	6.11e+07	3.92
5	9.01e+07	1.05e+08	9.88e+07	2.68
6	5.81e+07	6.68e+07	6.13e+07	2.69
7	8.79e+07	1.06e+08	9.87e+07	2.78

2. Block Parameters

The Isotopic Ratio of the blocks were calculated by 'Mean'

2.1. 13C/M0

Block	Number of scans	Effective number of ions	Isotopic Ratio	STD	SEM	RSE
1	723	1.24e+07	0.217410	0.001319	0.000049	0.000225
2	742	1.28e+07	0.216816	0.001363	0.000050	0.000231
3	746	1.29e+07	0.217417	0.001392	0.000051	0.000234
4	718	1.23e+07	0.217015	0.001317	0.000049	0.000226
5	706	1.21e+07	0.217542	0.001398	0.000053	0.000242
6	747	1.28e+07	0.216995	0.001364	0.000050	0.000230
7	747	1.26e+07	0.217485	0.001398	0.000051	0.000235

Errors and Test Paramters

Block	Acquisition Error (permil)	Shot-Noise (permil)	AE/SN ratio	Shapiro Wilk (p_value)	D'Agostino (p_value)
1	0.225	0.284	0.793	0.155	0.217
2	0.231	0.280	0.824	0.360	0.183
3	0.234	0.279	0.840	0.238	0.309
4	0.226	0.285	0.794	0.948	0.940
5	0.242	0.288	0.839	0.602	0.301
6	0.230	0.280	0.822	0.985	0.890
7	0.235	0.281	0.835	0.295	0.999

Isotopic Ratio and Errors of the Blocks

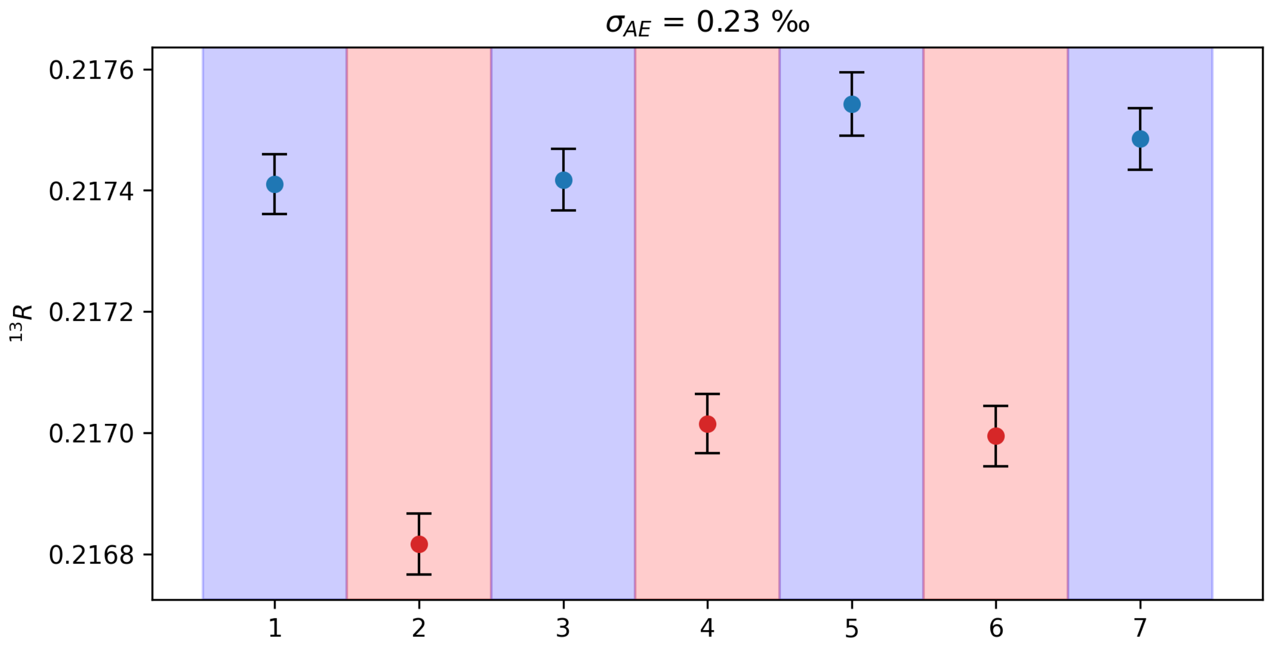

Cumulative Isotopic Ratio

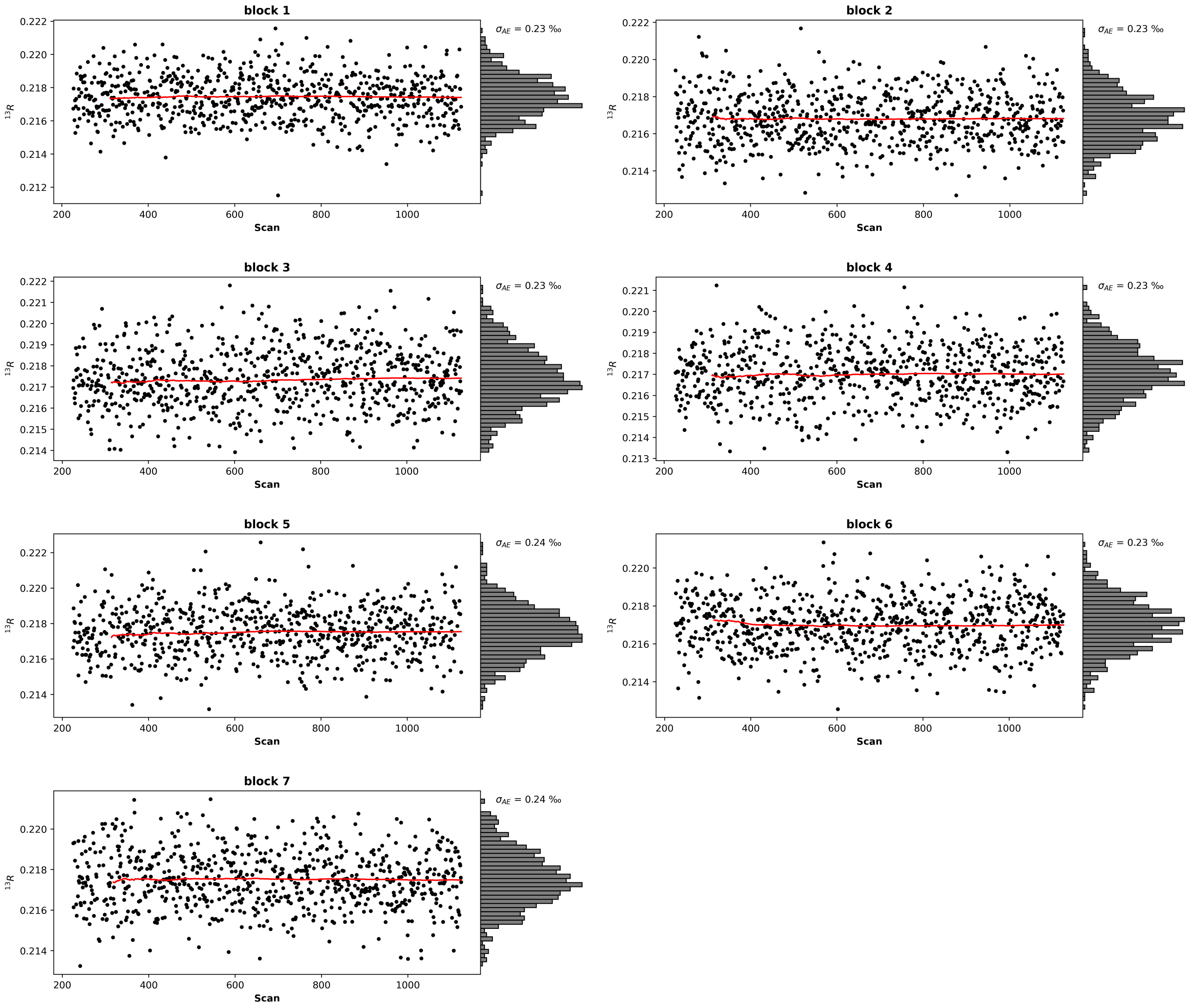

Acquisition Error and Shot-Noise

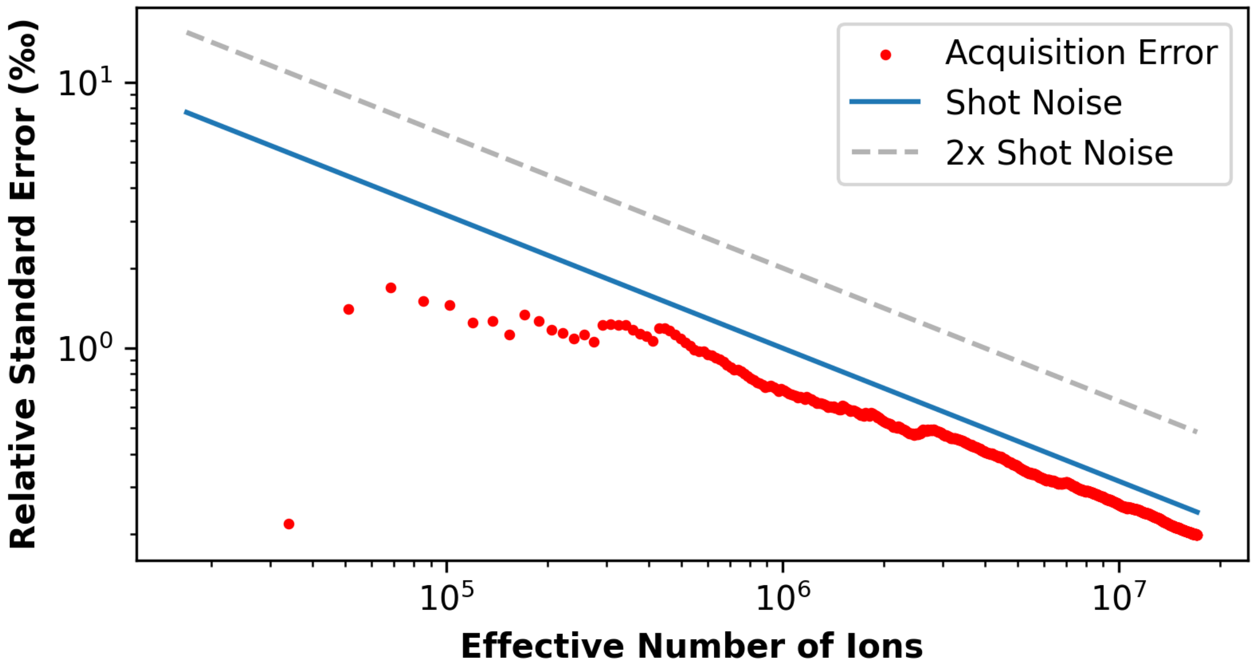

3. Delta Informations

Deltas were calculated by 'Average Of Neighboring Block Ratios'

3.1. 13C

Delta 13C was corrected by -27.80

Block	SEM	Delta corrected	Delta
2	0.23	-30.47	-2.75
4	0.23	-29.88	-2.14
6	0.23	-30.12	-2.39

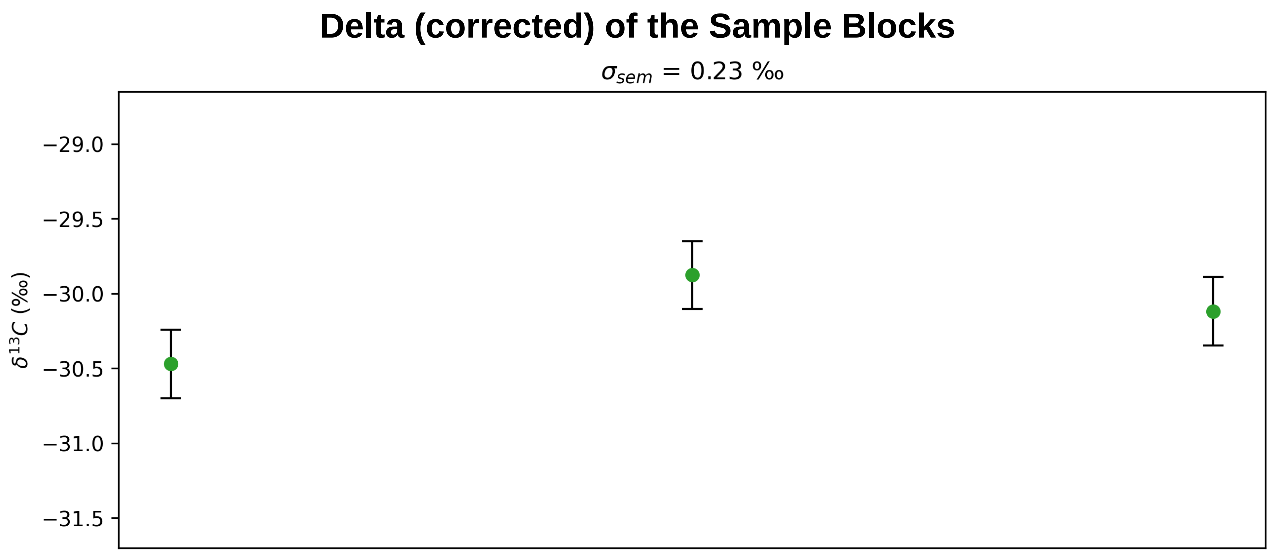

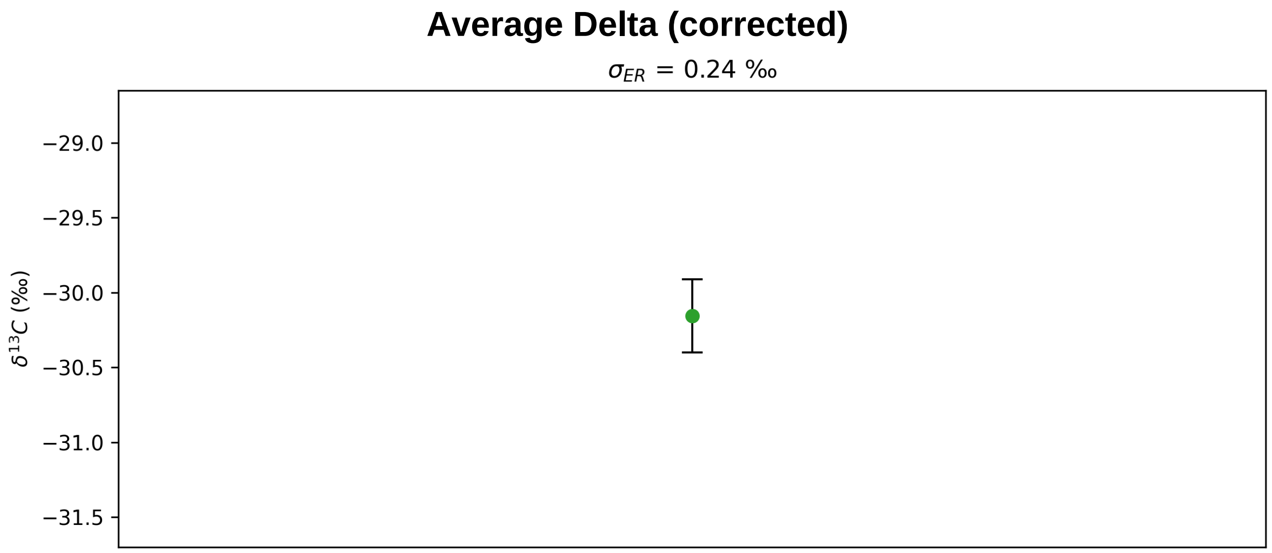

The final corrected average delta was -30.16 with a standard deviation of 0.24. Here the standard deviation is called reproducibility error.
