# Supplementary material for: Advancing Stable Isotope Analysis with Orbitrap-MS for Fatty Acid Methyl Esters and Complex Lipid Matrices
Source: J Am Soc Mass Spectrom. 2025 Jun 17;36(7):1527–35. doi: 10.1021/jasms.5c00092 (PMC12339014; doi:10.1021/jasms.5c00092)
Supplement: Supplementary file 2 [file js5c00092_si_002.zip › reports by IsotoPy Software/standards/Na+Standard2_DI.pdf]

# Standard 2 - [M + Na]+
# Isotope Analysis report from IsotoPy

Dual Inlet

# 1. Pre Processing

## 1.1. Block Time and Scan Information

Information about sample and standard block times and scans:

| Block | Injected | Initial Time | End Time | Number of scans |
|---|---|---|---|---|
| 1 | standard | 1 | 5 | 716 |
| 2 | sample | 6 | 10 | 721 |
| 3 | standard | 11 | 15 | 739 |
| 4 | sample | 16 | 20 | 718 |
| 5 | standard | 21 | 25 | 715 |
| 6 | sample | 26 | 30 | 702 |
| 7 | standard | 31 | 35 | 731 |

## 1.2. Outlier Removal

A total of 1263 scans were considered outliers and removed using the MAD method

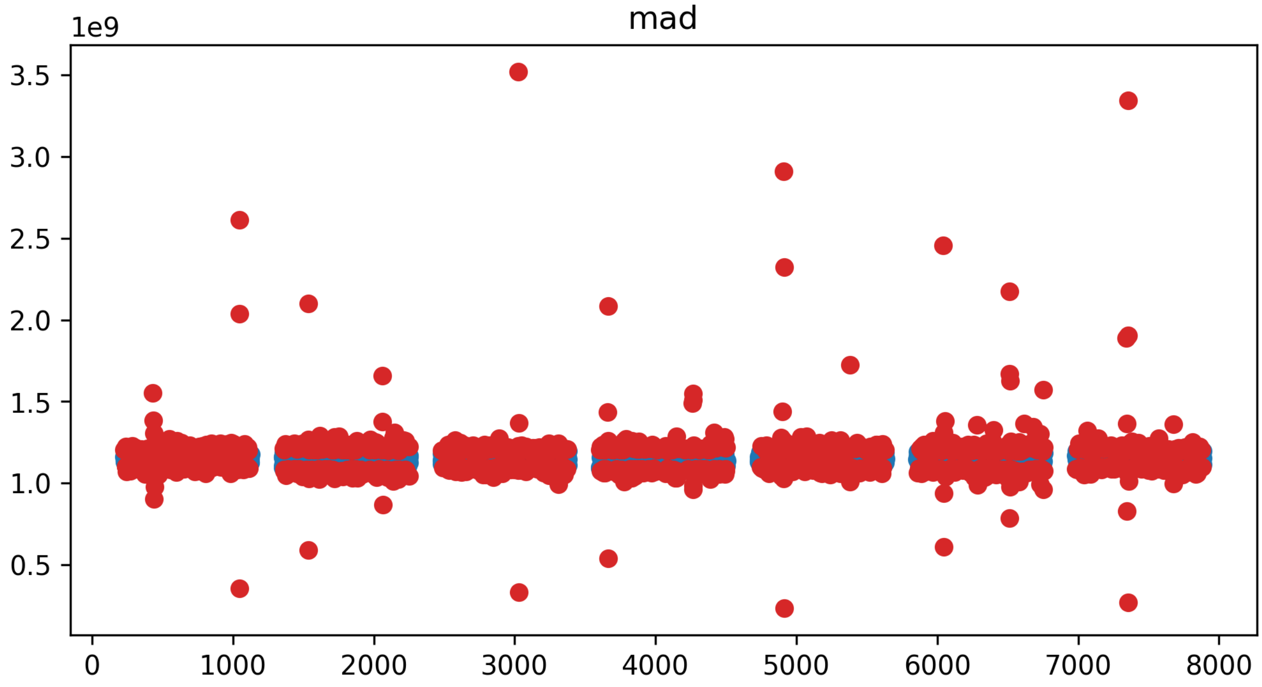

## 1.3. Total Ion Current (TIC)

TIC of all blocks

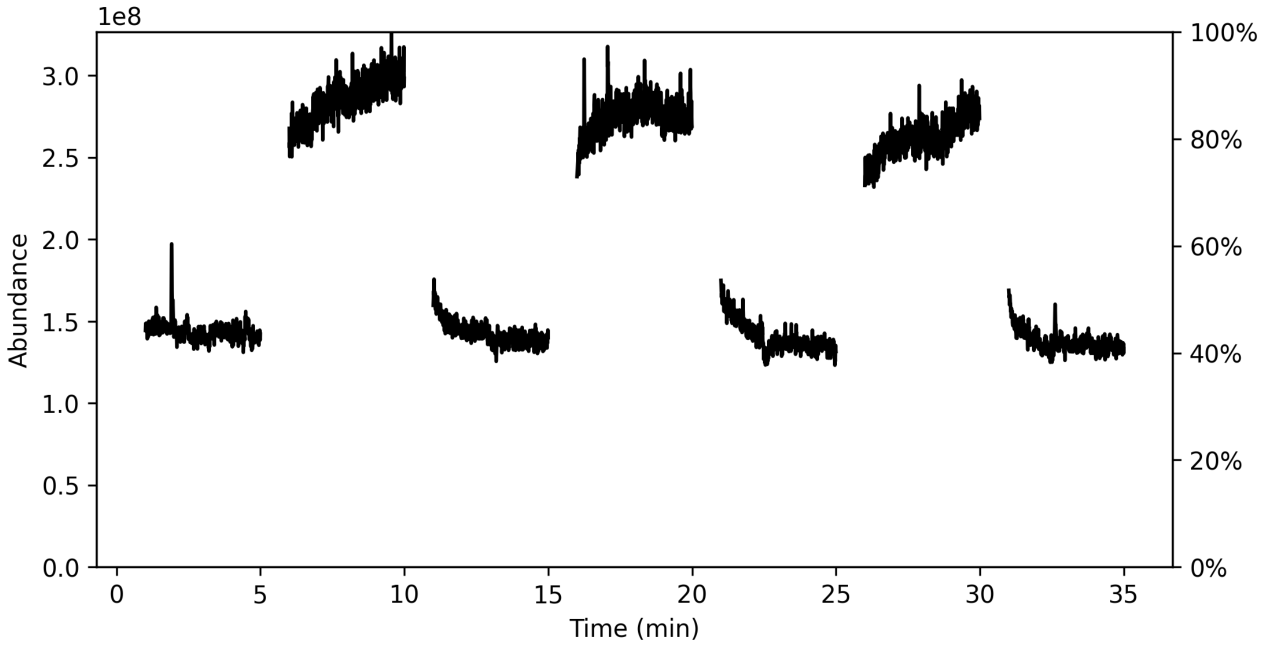


| Block | TIC min | TIC max | TIC mean | RSD (%) |
|---|---|---|---|---|
| 1 | 1.31e+08 | 1.97e+08 | 1.43e+08 | 3.83 |
| 2 | 2.50e+08 | 3.27e+08 | 2.85e+08 | 4.70 |
| 3 | 1.26e+08 | 1.76e+08 | 1.43e+08 | 5.12 |
| 4 | 2.38e+08 | 3.18e+08 | 2.75e+08 | 3.83 |
| 5 | 1.23e+08 | 1.75e+08 | 1.41e+08 | 6.86 |
| 6 | 2.32e+08 | 2.97e+08 | 2.62e+08 | 4.44 |
| 7 | 1.25e+08 | 1.69e+08 | 1.38e+08 | 4.90 |

# 2. Block Parameters

The Isotopic Ratio of the blocks were calculated by 'Mean'

## 2.1. 13C/M0

| Block | Number of scans | Effective number of ions | Isotopic Ratio | STD | SEM | RSE |
|---|---|---|---|---|---|---|
| 1 | 716 | 1.57e+07 | 0.210730 | 0.001407 | 0.000053 | 0.000249 |
| 2 | 721 | 1.59e+07 | 0.210342 | 0.001357 | 0.000050 | 0.000240 |
| 3 | 739 | 1.63e+07 | 0.210817 | 0.001347 | 0.000050 | 0.000235 |
| 4 | 718 | 1.58e+07 | 0.210345 | 0.001389 | 0.000052 | 0.000246 |
| 5 | 715 | 1.57e+07 | 0.210877 | 0.001354 | 0.000051 | 0.000240 |
| 6 | 702 | 1.54e+07 | 0.210478 | 0.001412 | 0.000053 | 0.000253 |
| 7 | 731 | 1.61e+07 | 0.210830 | 0.001343 | 0.000050 | 0.000235 |

**Errors and Test Paramters**

| Block | Acquisition Error (permil) | Shot-Noise (permil) | AE/SN ratio | Shapiro Wilk (p_value) | D'Agostino (p_value) |
|---|---|---|---|---|---|
| 1 | 0.249 | 0.252 | 0.989 | 0.640 | 0.669 |
| 2 | 0.240 | 0.251 | 0.956 | 0.233 | 0.149 |
| 3 | 0.235 | 0.248 | 0.948 | 0.499 | 0.642 |
| 4 | 0.246 | 0.252 | 0.978 | 0.123 | 0.472 |
| 5 | 0.240 | 0.252 | 0.952 | 0.600 | 0.286 |
| 6 | 0.253 | 0.254 | 0.994 | 0.319 | 0.119 |
| 7 | 0.235 | 0.249 | 0.944 | 0.553 | 0.373 |

# Isotopic Ratio and Errors of the Blocks

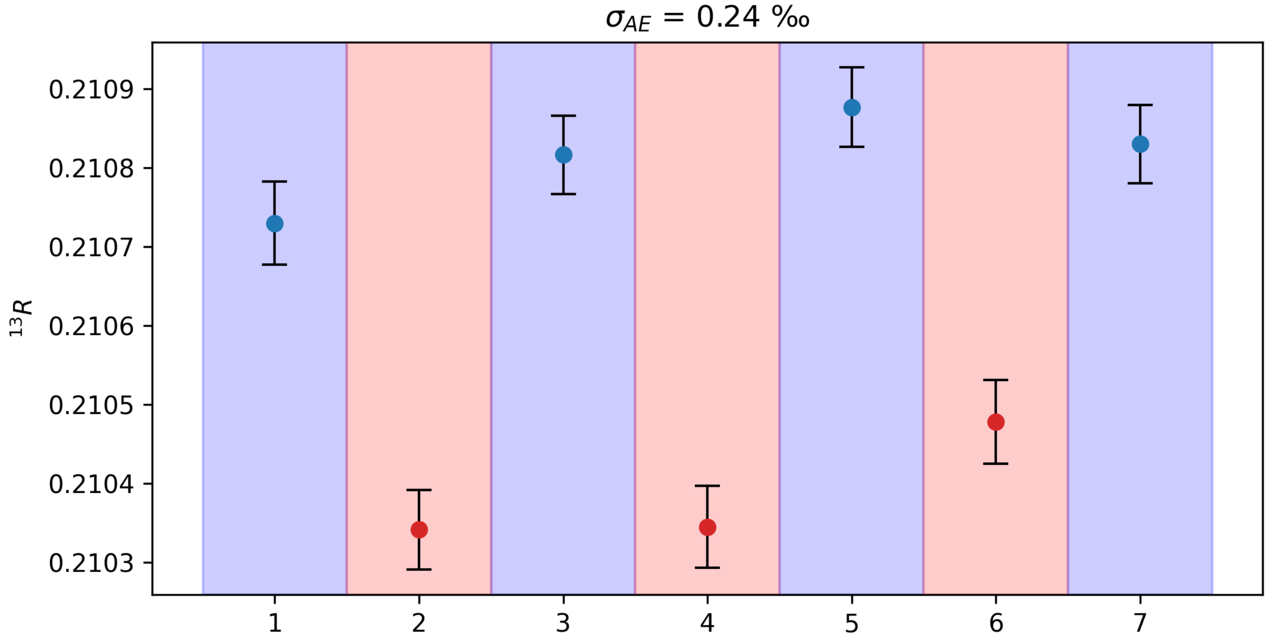


# Cumulative Isotopic Ratio

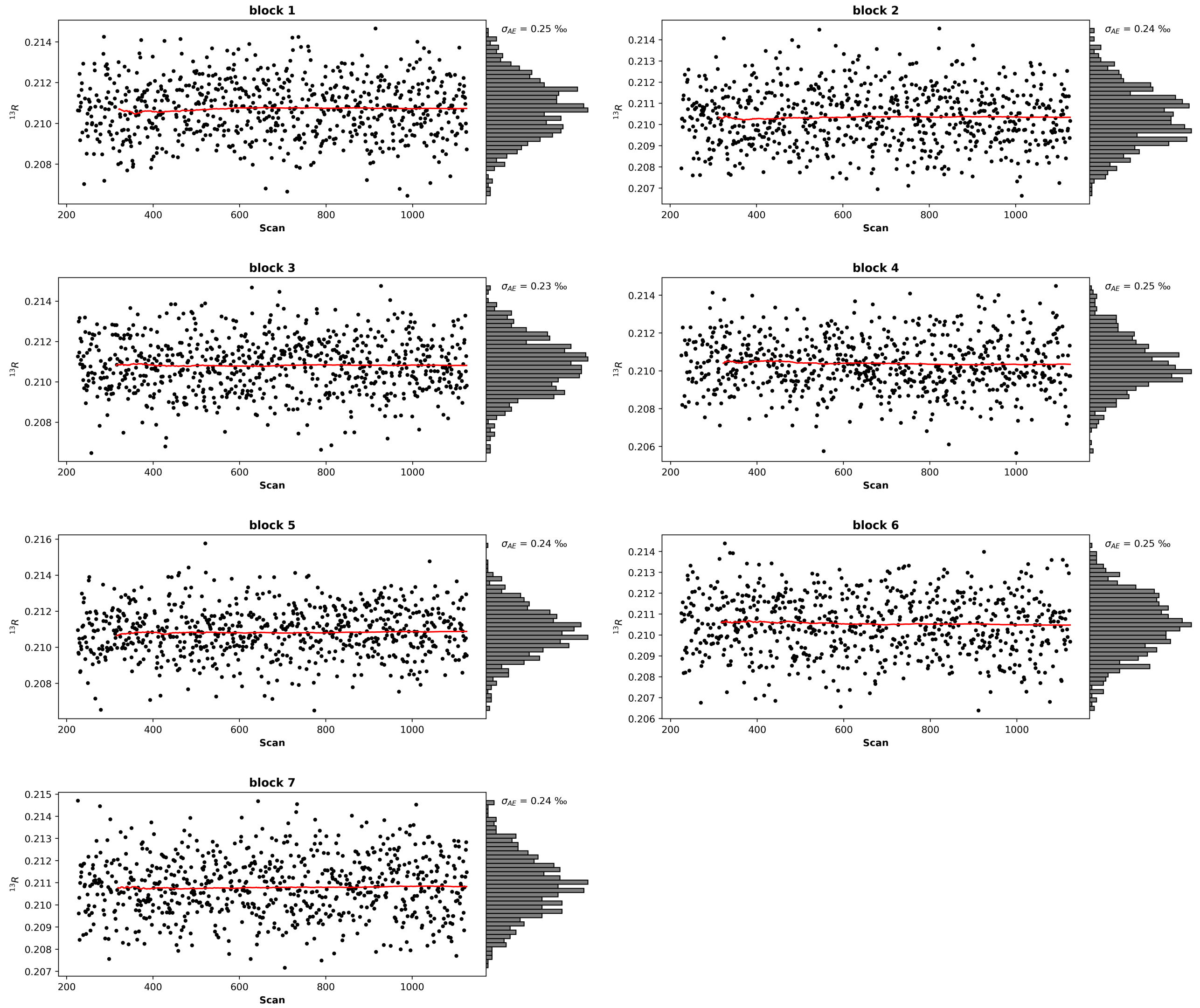

**Acquisition Error and Shot-Noise**

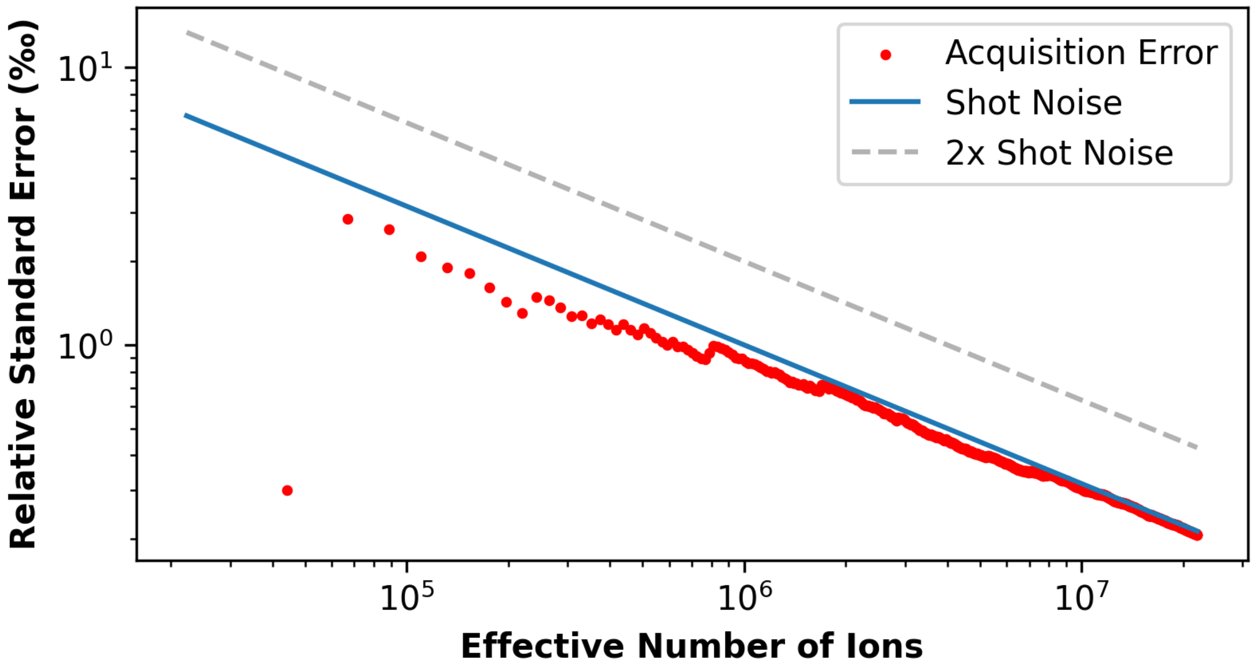

# 3. Delta Informations

Deltas were calculated by 'Average Of Neighboring Block Ratios'

## 3.1. 13C

Delta 13C was corrected by -27.80

| Block | SEM | Delta corrected | Delta |
|---|---|---|---|
| 2 | 0.24 | -29.79 | -2.05 |
| 4 | 0.25 | -30.11 | -2.38 |
| 6 | 0.25 | -29.53 | -1.78 |

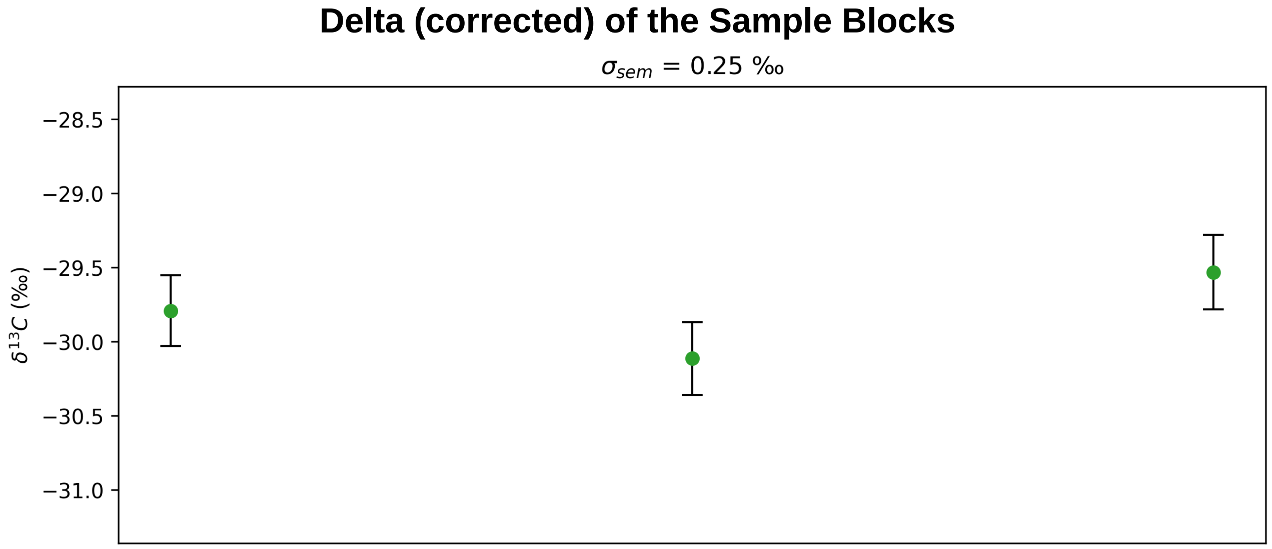


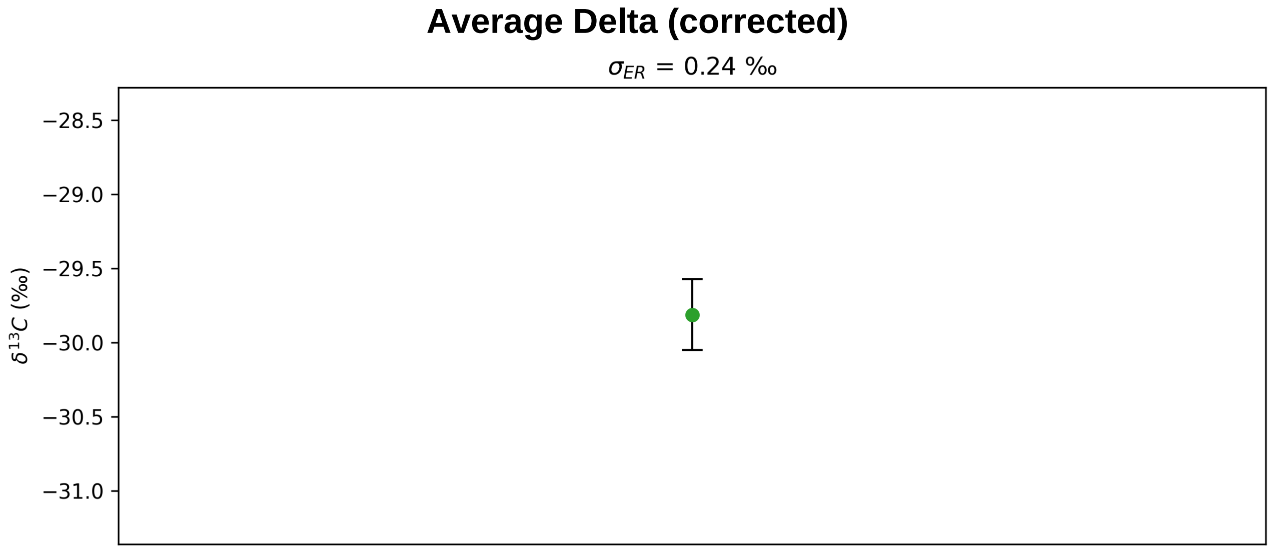


The final corrected average delta was -29.81 with a standard deviation of 0.24. Here the standard deviation is called reproducibility error.
