# Supplementary material for: Advancing Stable Isotope Analysis with Orbitrap-MS for Fatty Acid Methyl Esters and Complex Lipid Matrices
Source: J Am Soc Mass Spectrom. 2025 Jun 17;36(7):1527–35. doi: 10.1021/jasms.5c00092 (PMC12339014; doi:10.1021/jasms.5c00092)
Supplement: Supplementary file 2 [file js5c00092_si_002.zip › reports by IsotoPy Software/standards/Na+Standard5_FI.pdf]

# Standard 5 - [M + Na]+
# Isotope Analysis report from IsotoPy

Flow Injection

# 1. Pre Processing

## 1.1. Block Time and Scan Information

Information about sample and standard block times and scans:

| Block | Injected | Initial Time | End Time | Number of scans |
|---|---|---|---|---|
| 1 | standard | 1 | 8 | 1296 |
| 2 | sample | 16 | 23 | 1251 |
| 3 | standard | 31 | 38 | 1304 |
| 4 | sample | 46 | 53 | 1306 |
| 5 | standard | 61 | 68 | 1297 |
| 6 | sample | 76 | 83 | 1267 |
| 7 | standard | 91 | 98 | 1258 |

## 1.2. Outlier Removal

A total of 2060 scans were considered outliers and removed using the MAD method

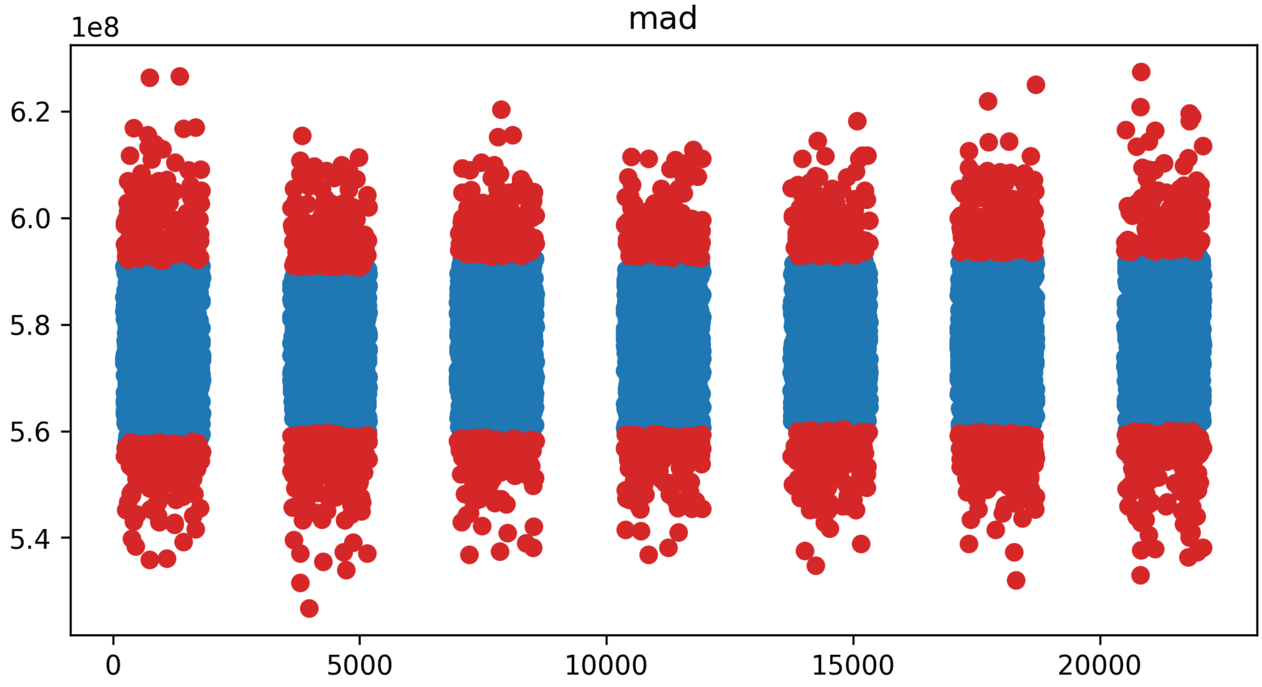

## 1.3. Total Ion Current (TIC)

TIC of all blocks

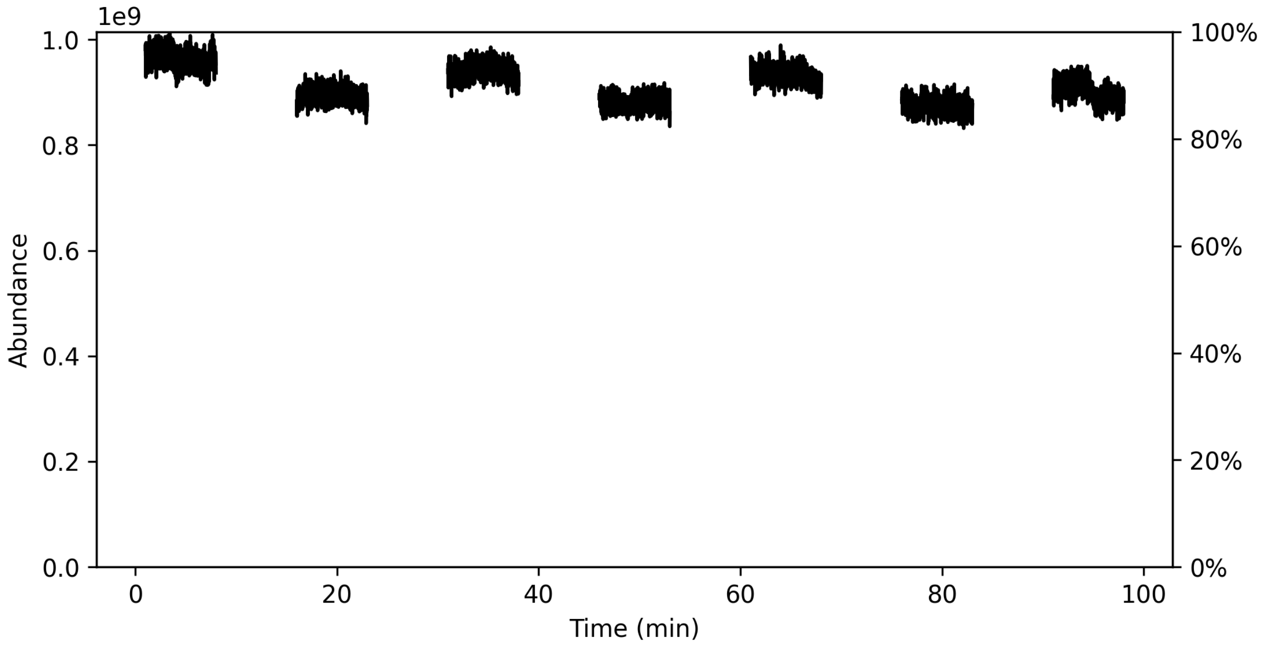


| Block | TIC min | TIC max | TIC mean | RSD (%) |
|---:|---:|---:|---:|---:|
| 1 | 9.11e+08 | 1.01e+09 | 9.64e+08 | 1.81 |
| 2 | 8.41e+08 | 9.40e+08 | 8.94e+08 | 1.51 |
| 3 | 8.92e+08 | 9.86e+08 | 9.41e+08 | 1.56 |
| 4 | 8.36e+08 | 9.18e+08 | 8.80e+08 | 1.42 |
| 5 | 8.89e+08 | 9.89e+08 | 9.34e+08 | 1.61 |
| 6 | 8.32e+08 | 9.15e+08 | 8.75e+08 | 1.57 |
| 7 | 8.48e+08 | 9.50e+08 | 9.02e+08 | 2.22 |

# 2. Block Parameters

The Isotopic Ratio of the blocks were calculated by 'Mean'

## 2.1. 13C/M0

| Block | Number of scans | Effective number of ions | Isotopic Ratio | STD | SEM | RSE |
|---|---|---|---|---|---|---|
| 1 | 1296 | 2.09e+07 | 0.209506 | 0.001784 | 0.000050 | 0.000236 |
| 2 | 1251 | 2.01e+07 | 0.209744 | 0.001798 | 0.000051 | 0.000242 |
| 3 | 1304 | 2.09e+07 | 0.209181 | 0.001771 | 0.000049 | 0.000234 |
| 4 | 1306 | 2.10e+07 | 0.209294 | 0.001784 | 0.000049 | 0.000236 |
| 5 | 1297 | 2.08e+07 | 0.208936 | 0.001824 | 0.000051 | 0.000242 |
| 6 | 1267 | 2.03e+07 | 0.208971 | 0.001722 | 0.000048 | 0.000231 |
| 7 | 1258 | 2.02e+07 | 0.208923 | 0.001800 | 0.000051 | 0.000243 |

**Errors and Test Paramters**

| Block | Acquisition Error (permil) | Shot-Noise (permil) | AE/SN ratio | Shapiro Wilk (p_value) | D'Agostino (p_value) |
|---|---|---|---|---|---|
| 1 | 0.236 | 0.219 | 1.082 | 0.721 | 0.804 |
| 2 | 0.242 | 0.223 | 1.087 | 0.386 | 0.749 |
| 3 | 0.234 | 0.219 | 1.072 | 0.934 | 0.971 |
| 4 | 0.236 | 0.218 | 1.080 | 0.108 | 0.161 |
| 5 | 0.242 | 0.219 | 1.106 | 0.376 | 0.169 |
| 6 | 0.231 | 0.222 | 1.043 | 0.181 | 0.456 |
| 7 | 0.243 | 0.222 | 1.092 | 0.256 | 0.550 |

# Isotopic Ratio and Errors of the Blocks

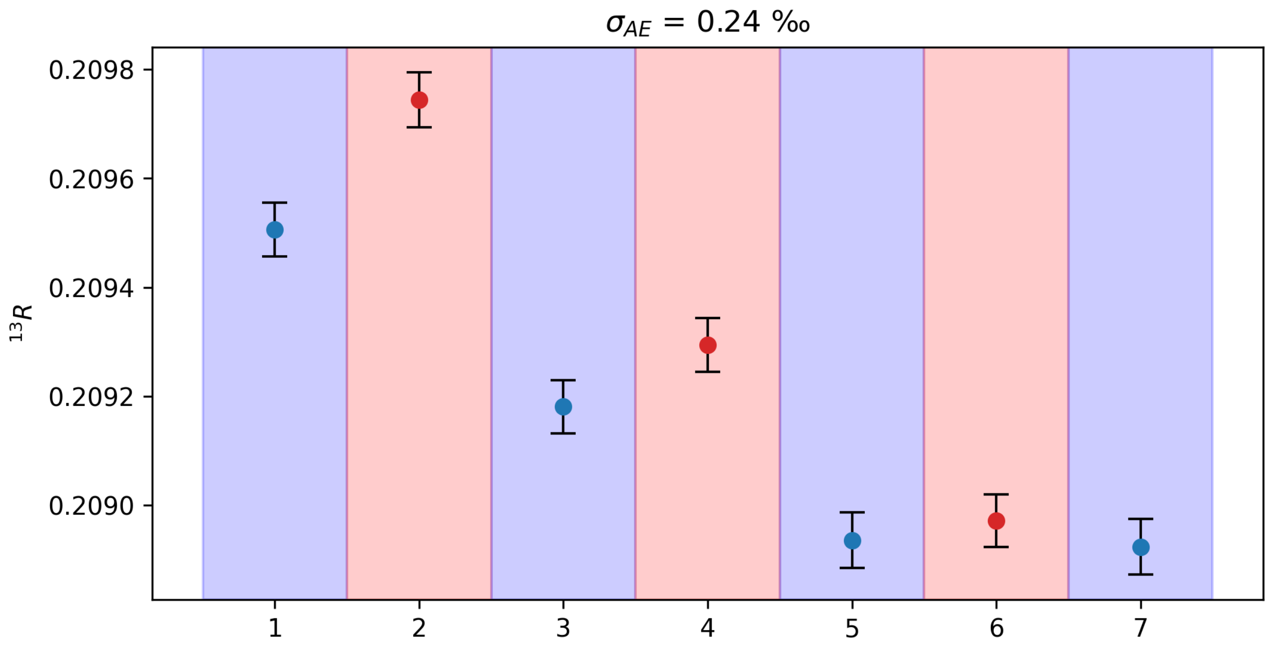


# Cumulative Isotopic Ratio

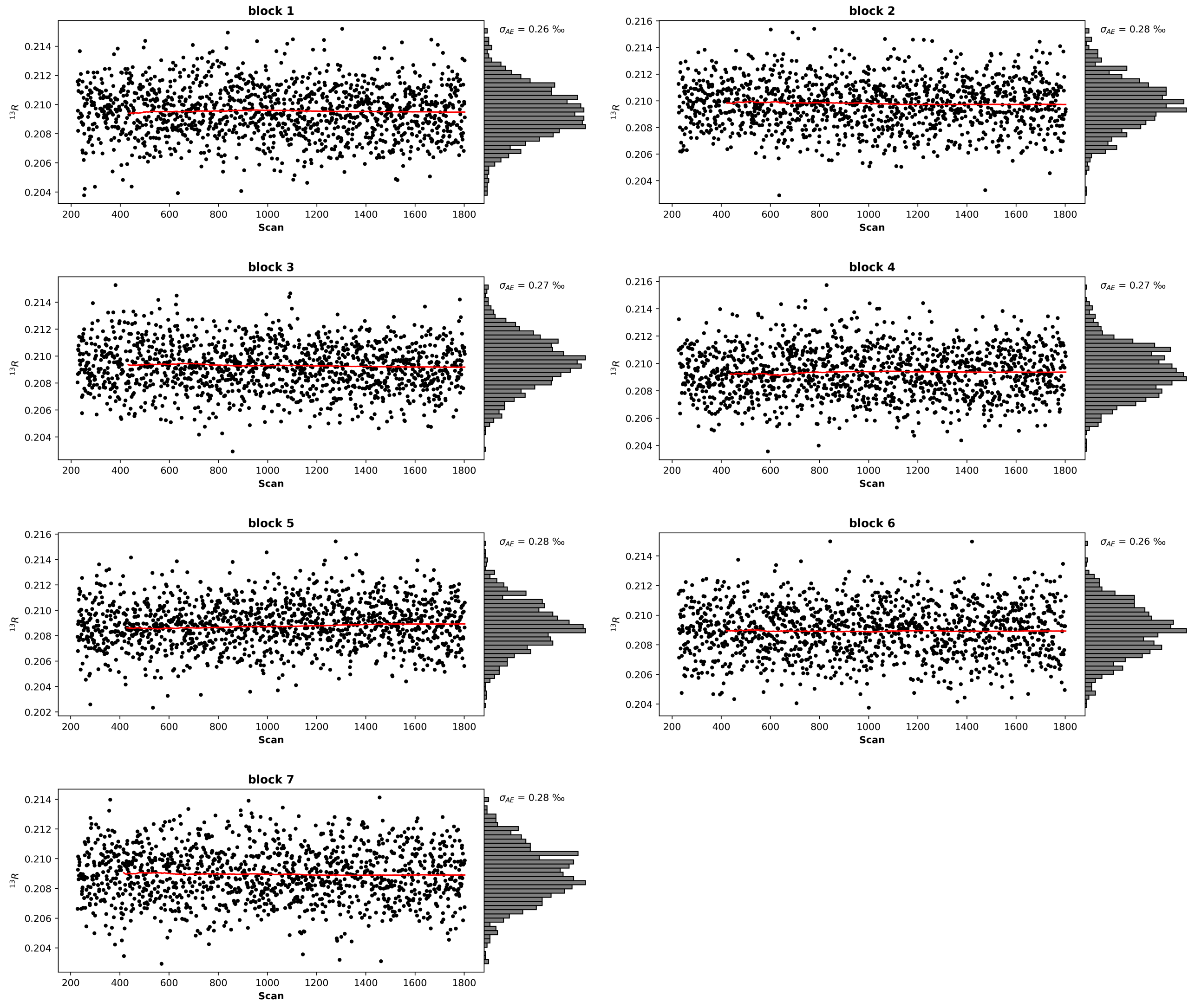

# Acquisition Error and Shot-Noise

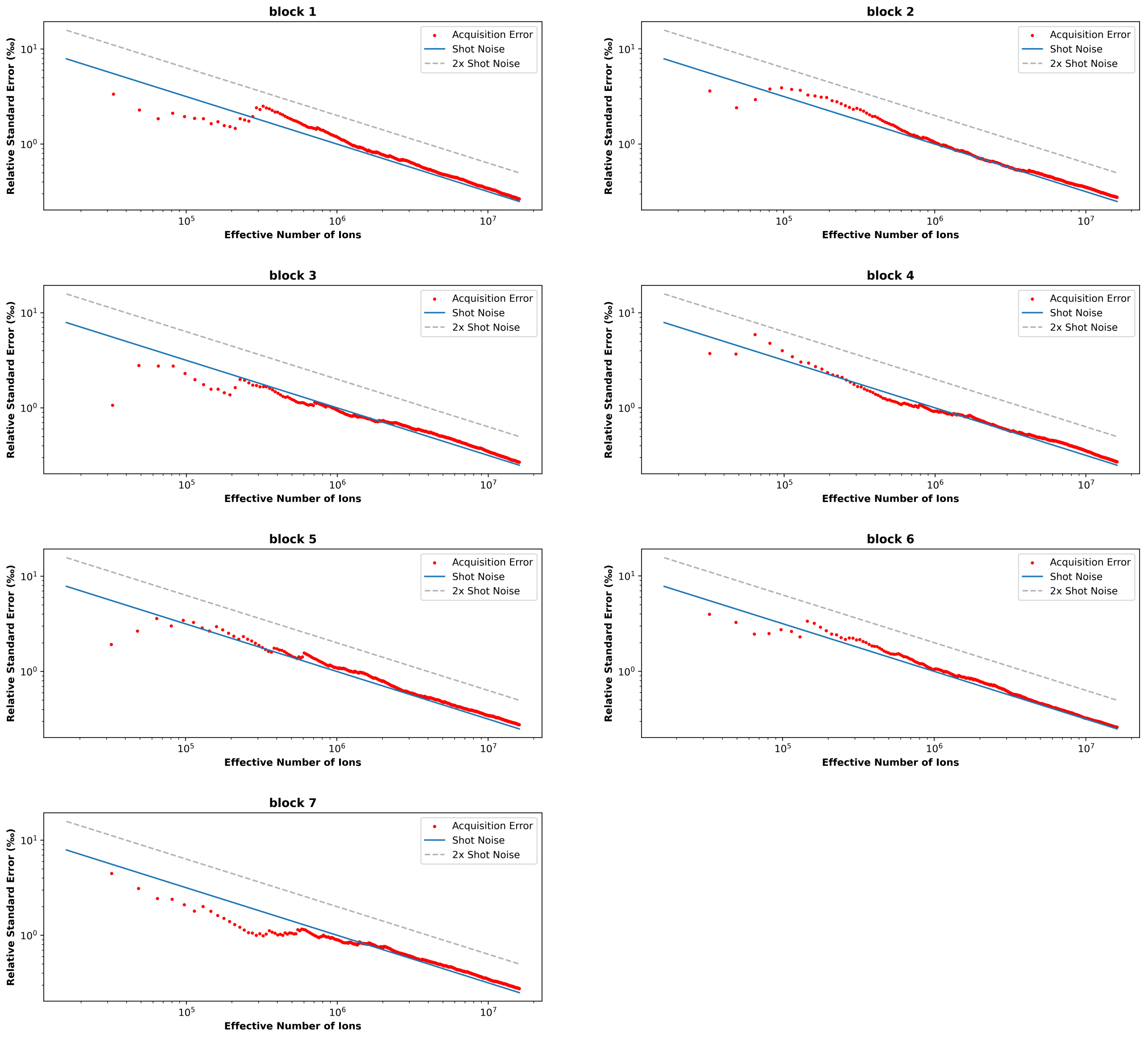

# 3. Delta Informations

Deltas were calculated by 'Average Of Neighboring Block Ratios'

## 3.1. 13C

Delta 13C was corrected by -27.80

| Block | SEM | Delta corrected | Delta |
|---|---|---|---|
| 2 | 0.24 | -25.94 | 1.91 |
| 4 | 0.24 | -26.70 | 1.13 |
| 6 | 0.23 | -27.61 | 0.20 |

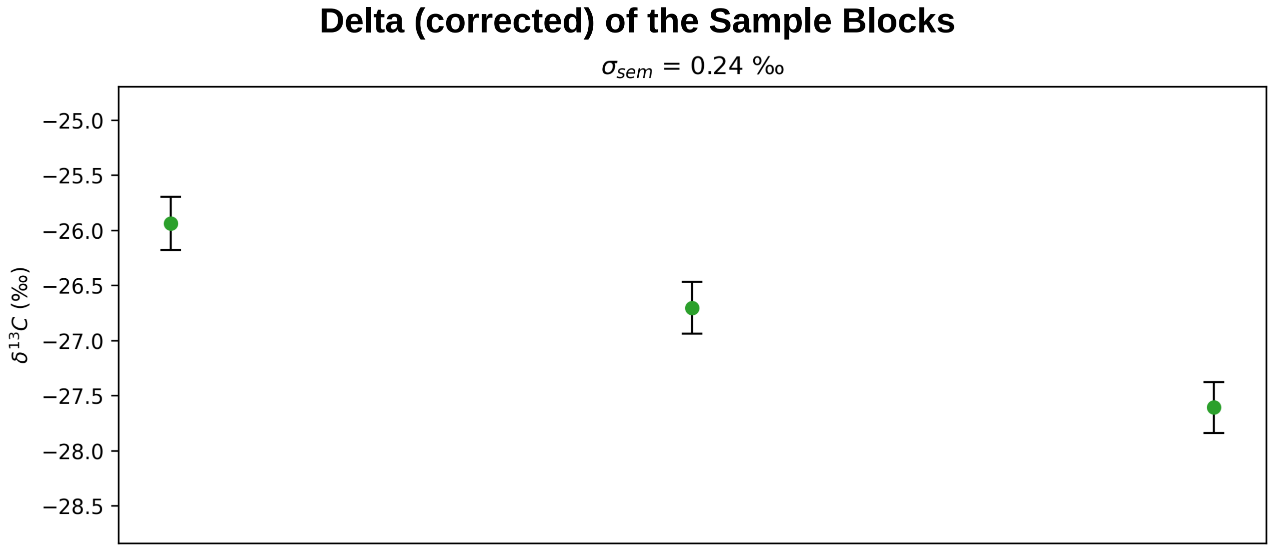


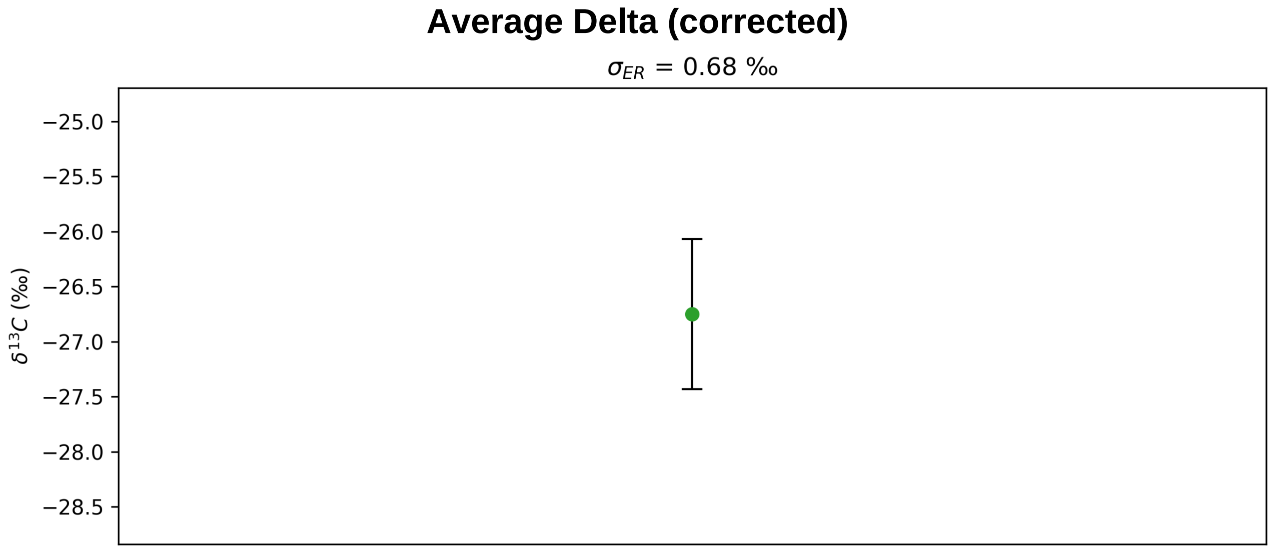


The final corrected average delta was -26.75 with a standard deviation of 0.68. Here the standard deviation is called reproducibility error.
