# Supplementary material for: Advancing Stable Isotope Analysis with Orbitrap-MS for Fatty Acid Methyl Esters and Complex Lipid Matrices
Source: J Am Soc Mass Spectrom. 2025 Jun 17;36(7):1527–35. doi: 10.1021/jasms.5c00092 (PMC12339014; doi:10.1021/jasms.5c00092)
Supplement: Supplementary file 2 [file js5c00092_si_002.zip › reports by IsotoPy Software/standards/Na+Standard9_FI.pdf]

Standard 9 - [M + Na]+
Isotope Analysis report from IsotoPy
Flow Injection

1. Pre Processing

1.1. Block Time and Scan Information

Information about sample and standard block times and scans:

Block	Injected	Initial Time	End Time	Number of scans
1	standard	1	8	1273
2	sample	16	23	1275
3	standard	31	38	1273
4	sample	46	53	1286
5	standard	61	68	1281
6	sample	76	83	1267
7	standard	91	98	1289

1.2. Outlier Removal

A total of 2095 scans were considered outliers and removed using the MAD method

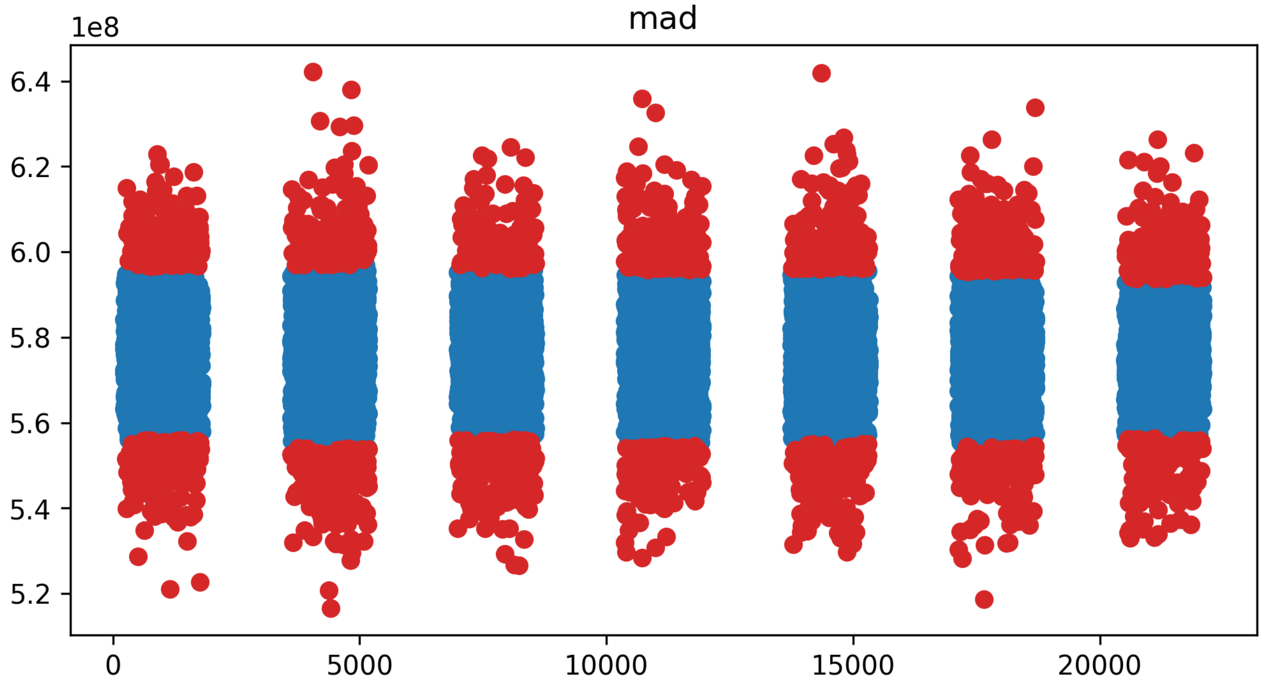

1.3. Total Ion Current (TIC)

TIC of all blocks

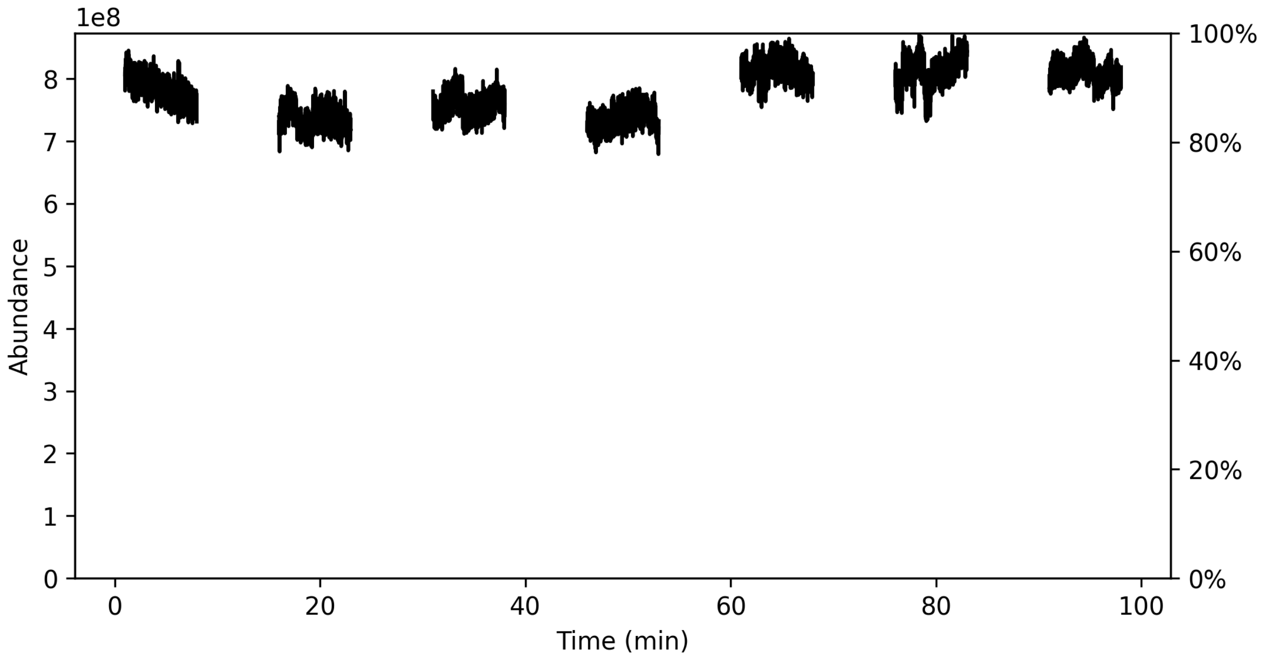

Block	TIC min	TIC max	TIC mean	RSD (%)
1	7.29e+08	8.45e+08	7.85e+08	2.55
2	6.83e+08	7.89e+08	7.36e+08	2.41
3	7.12e+08	8.16e+08	7.57e+08	2.39
4	6.79e+08	7.85e+08	7.36e+08	2.24
5	7.54e+08	8.64e+08	8.14e+08	2.03
6	7.32e+08	8.73e+08	8.12e+08	2.88
7	7.51e+08	8.66e+08	8.10e+08	2.02

2. Block Parameters

The Isotopic Ratio of the blocks were calculated by 'Mean'

2.1. 13C/M0

Block	Number of scans	Effective number of ions	Isotopic Ratio	STD	SEM	RSE
1	1273	2.05e+07	0.209284	0.001820	0.000051	0.000244
2	1275	2.05e+07	0.209677	0.001721	0.000048	0.000230
3	1273	2.05e+07	0.209647	0.001757	0.000049	0.000235
4	1286	2.07e+07	0.209602	0.001790	0.000050	0.000238
5	1281	2.06e+07	0.209468	0.001775	0.000050	0.000237
6	1267	2.04e+07	0.209641	0.001762	0.000049	0.000236
7	1289	2.07e+07	0.209447	0.001732	0.000048	0.000230

Errors and Test Paramters

Block	Acquisition Error (permil)	Shot-Noise (permil)	AE/SN ratio	Shapiro Wilk (p_value)	D'Agostino (p_value)
1	0.244	0.221	1.102	0.493	0.372
2	0.230	0.221	1.041	0.443	0.402
3	0.235	0.221	1.064	0.508	0.891
4	0.238	0.220	1.083	0.638	0.407
5	0.237	0.220	1.074	0.652	0.587
6	0.236	0.221	1.066	0.693	0.432
7	0.230	0.220	1.048	0.668	0.880

Isotopic Ratio and Errors of the Blocks

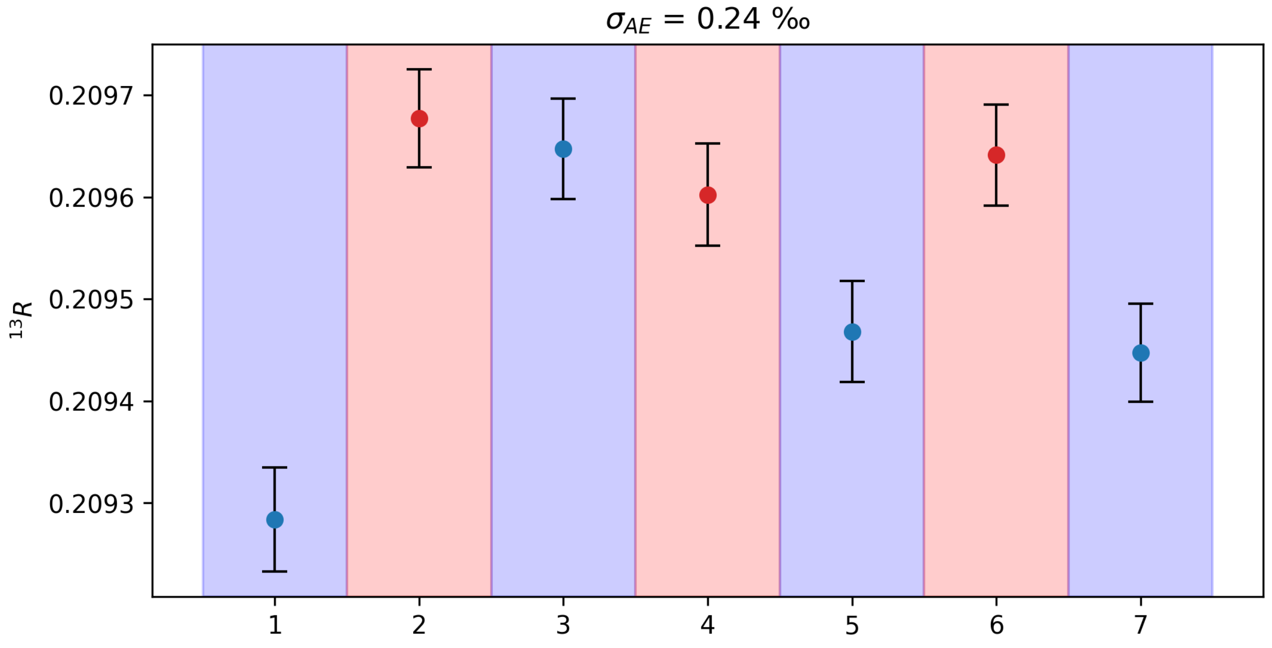

Cumulative Isotopic Ratio

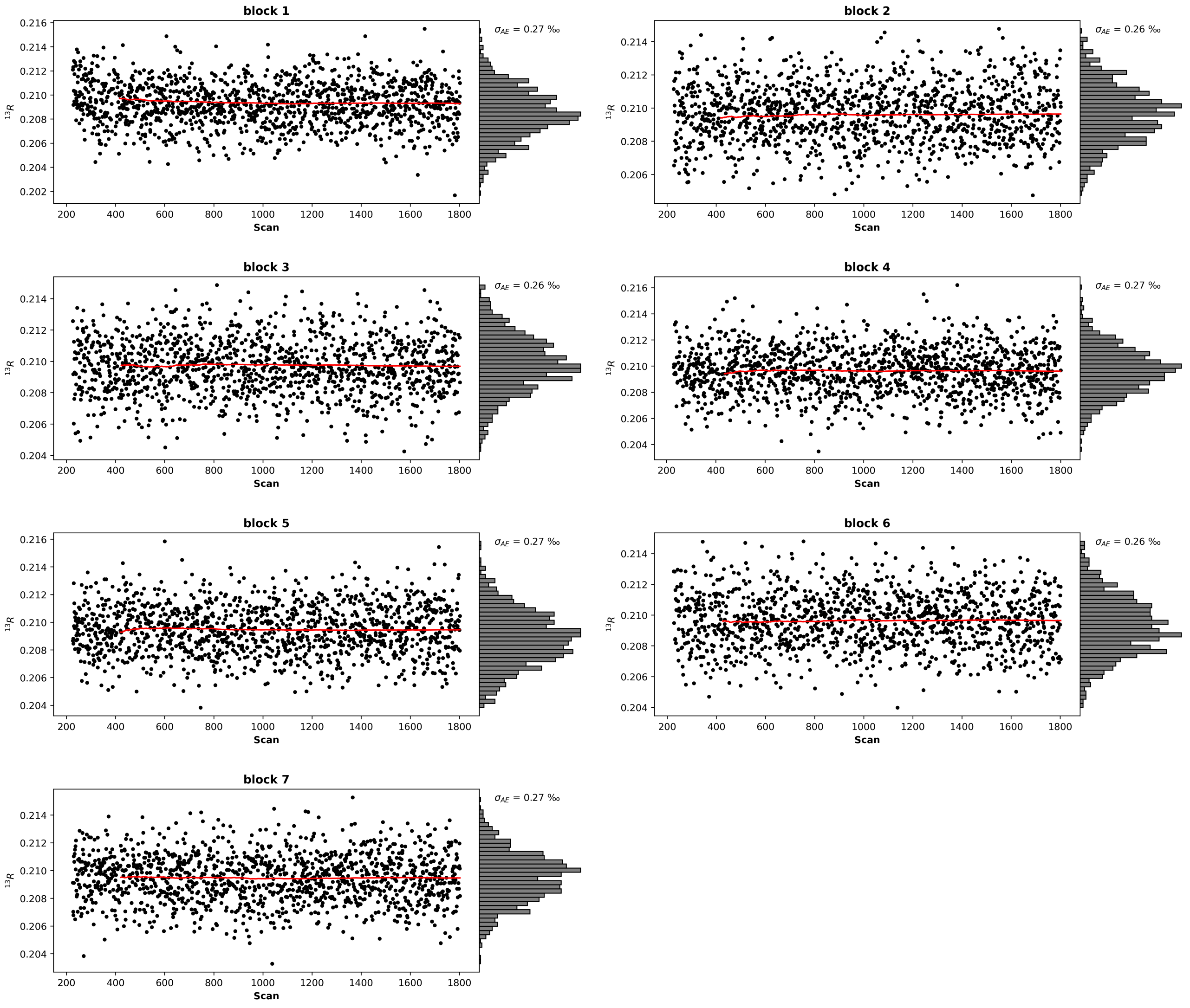

Acquisition Error and Shot-Noise

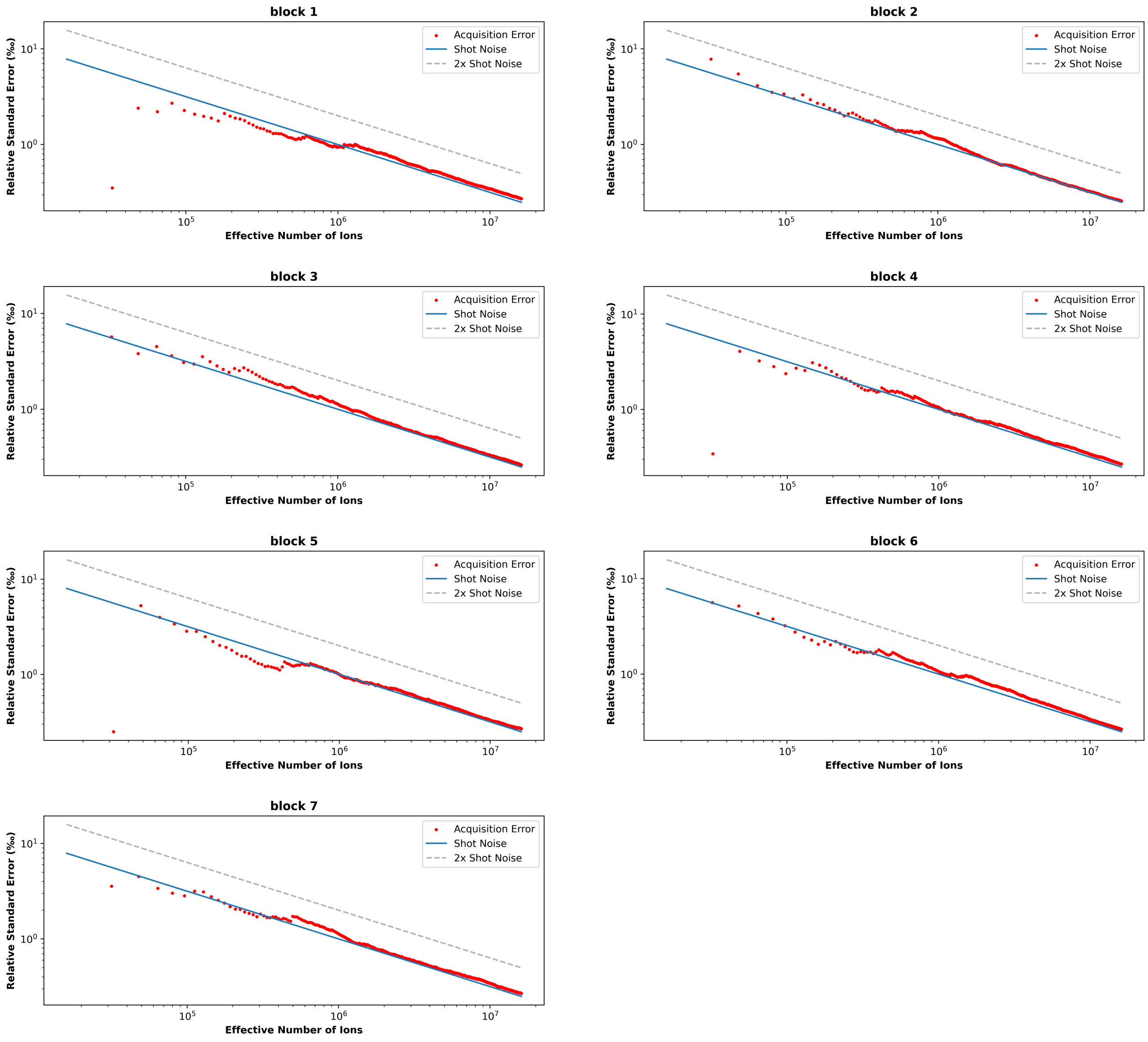

3. Delta Informations

Deltas were calculated by 'Average Of Neighboring Block Ratios'

3.1. 13C

Delta 13C was corrected by -27.80

Block	SEM	Delta corrected	Delta
2	0.23	-26.82	1.01
4	0.24	-27.59	0.21
6	0.24	-26.95	0.88

Delta (corrected) of the Sample Blocks

σ_{sem} = 0.23 ‰

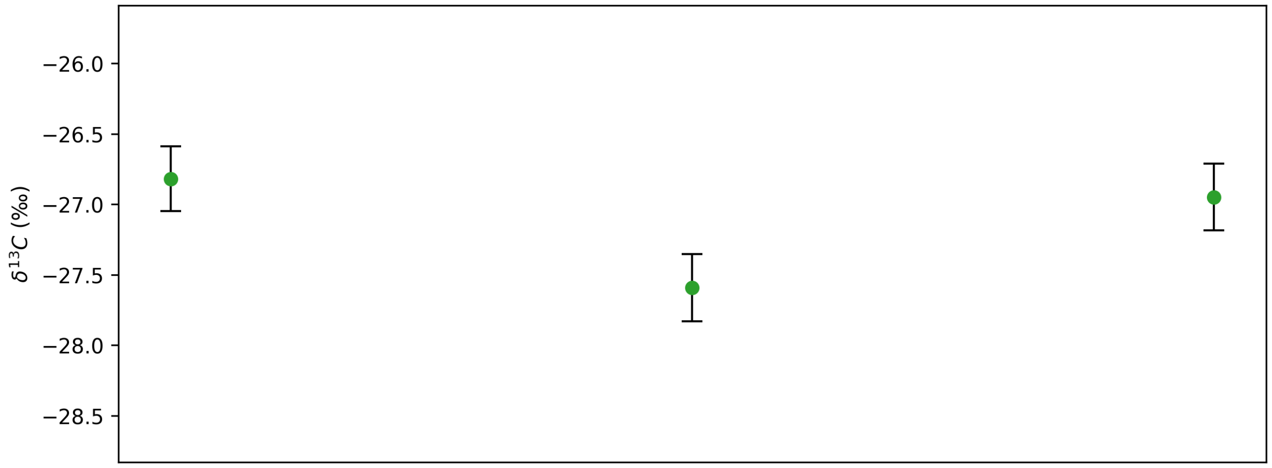

Average Delta (corrected)

σ_{ER} = 0.34 ‰

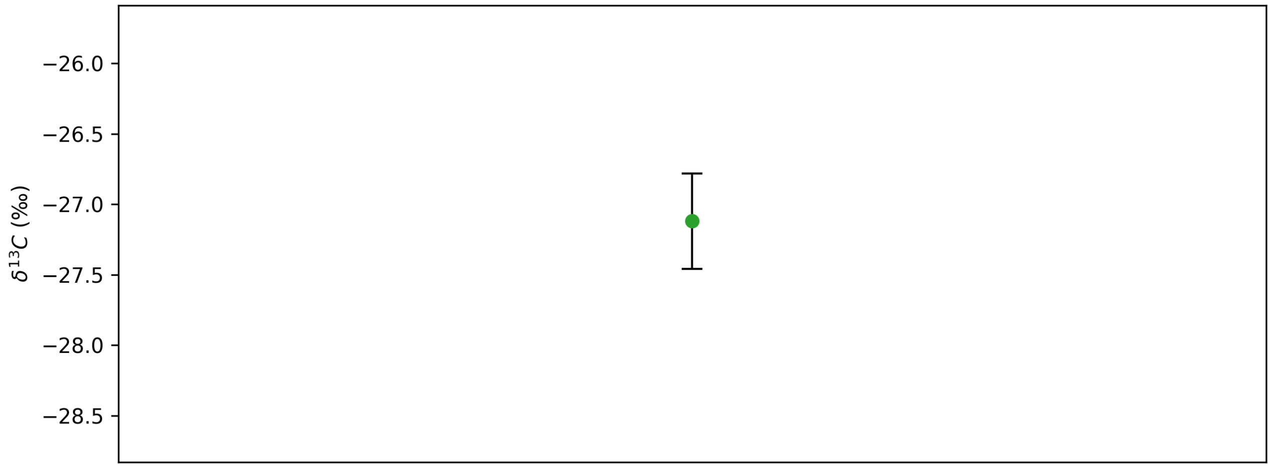

The final corrected average delta was -27.12 with a standard deviation of 0.34. Here the standard deviation is called reproducibility error.
