# Supplementary material for: Advancing Stable Isotope Analysis with Orbitrap-MS for Fatty Acid Methyl Esters and Complex Lipid Matrices
Source: J Am Soc Mass Spectrom. 2025 Jun 17;36(7):1527–35. doi: 10.1021/jasms.5c00092 (PMC12339014; doi:10.1021/jasms.5c00092)
Supplement: Supplementary file 2 [file js5c00092_si_002.zip › reports by IsotoPy Software/standards/Na+Standard7_FI.pdf]

Standard 7 - [M + Na]+
Isotope Analysis report from IsotoPy

Flow Injection

1. Pre Processing

1.1. Block Time and Scan Information

Information about sample and standard block times and scans:

Block	Injected	Initial Time	End Time	Number of scans
1	standard	1	8	1314
2	sample	16	23	1304
3	standard	31	38	1296
4	sample	46	53	1278
5	standard	61	68	1274
6	sample	76	83	1298
7	standard	91	98	1293

1.2. Outlier Removal

A total of 1982 scans were considered outliers and removed using the MAD method

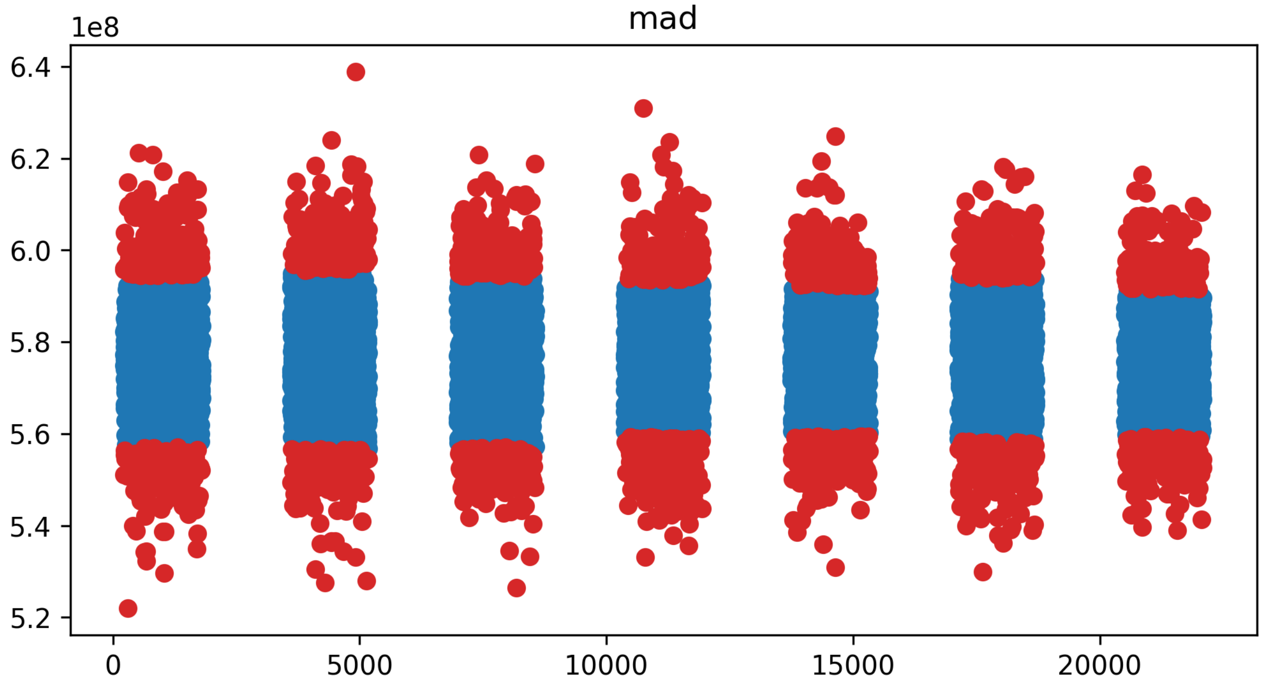

1.3. Total Ion Current (TIC)

TIC of all blocks

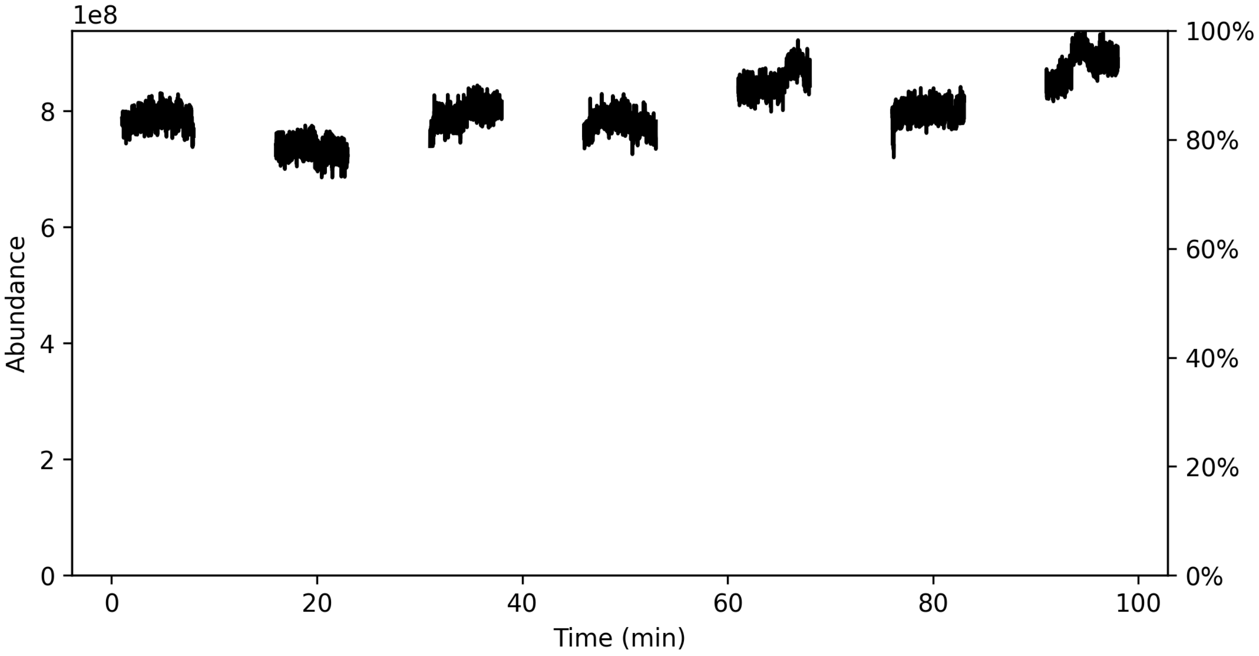

Block	TIC min	TIC max	TIC mean	RSD (%)
1	7.38e+08	8.31e+08	7.87e+08	1.80
2	6.85e+08	7.75e+08	7.34e+08	2.01
3	7.39e+08	8.44e+08	7.97e+08	2.25
4	7.26e+08	8.30e+08	7.80e+08	1.98
5	7.99e+08	9.22e+08	8.52e+08	2.62
6	7.20e+08	8.42e+08	7.99e+08	1.83
7	8.17e+08	9.38e+08	8.82e+08	2.97

2. Block Parameters

The Isotopic Ratio of the blocks were calculated by 'Mean'

2.1. 13C/M0

Block	Number of scans	Effective number of ions	Isotopic Ratio	STD	SEM	RSE
1	1314	2.11e+07	0.209026	0.001788	0.000049	0.000236
2	1304	2.09e+07	0.209097	0.001801	0.000050	0.000238
3	1296	2.08e+07	0.209127	0.001833	0.000051	0.000243
4	1278	2.05e+07	0.208939	0.001742	0.000049	0.000233
5	1274	2.05e+07	0.209235	0.001831	0.000051	0.000245
6	1298	2.08e+07	0.209043	0.001704	0.000047	0.000226
7	1293	2.08e+07	0.209298	0.001737	0.000048	0.000231

Errors and Test Paramters

Block	Acquisition Error (permil)	Shot-Noise (permil)	AE/SN ratio	Shapiro Wilk (p_value)	D'Agostino (p_value)
1	0.236	0.218	1.083	0.023	0.026
2	0.238	0.219	1.091	0.396	0.733
3	0.243	0.219	1.110	0.388	0.368
4	0.233	0.221	1.056	0.109	0.100
5	0.245	0.221	1.109	0.918	0.805
6	0.226	0.219	1.033	0.912	0.766
7	0.231	0.219	1.052	0.894	0.865

Isotopic Ratio and Errors of the Blocks

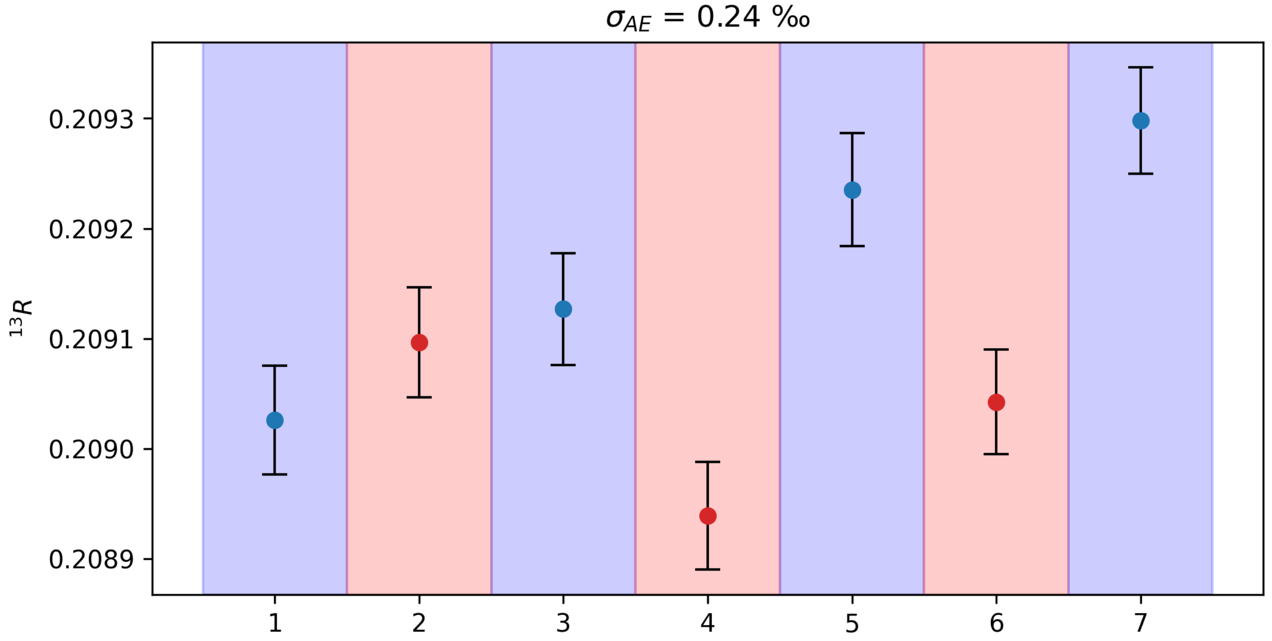

Cumulative Isotopic Ratio

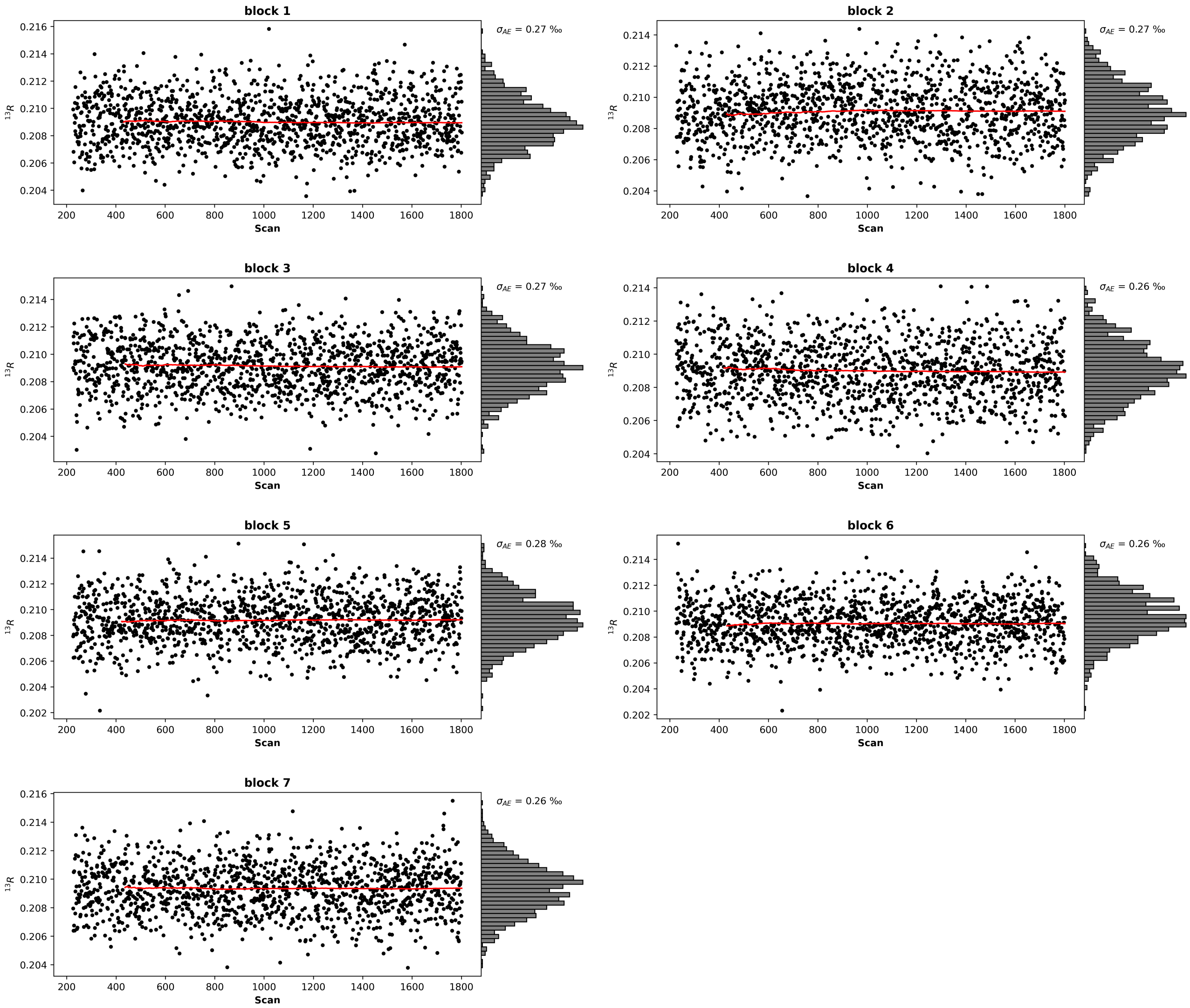

Acquisition Error and Shot-Noise

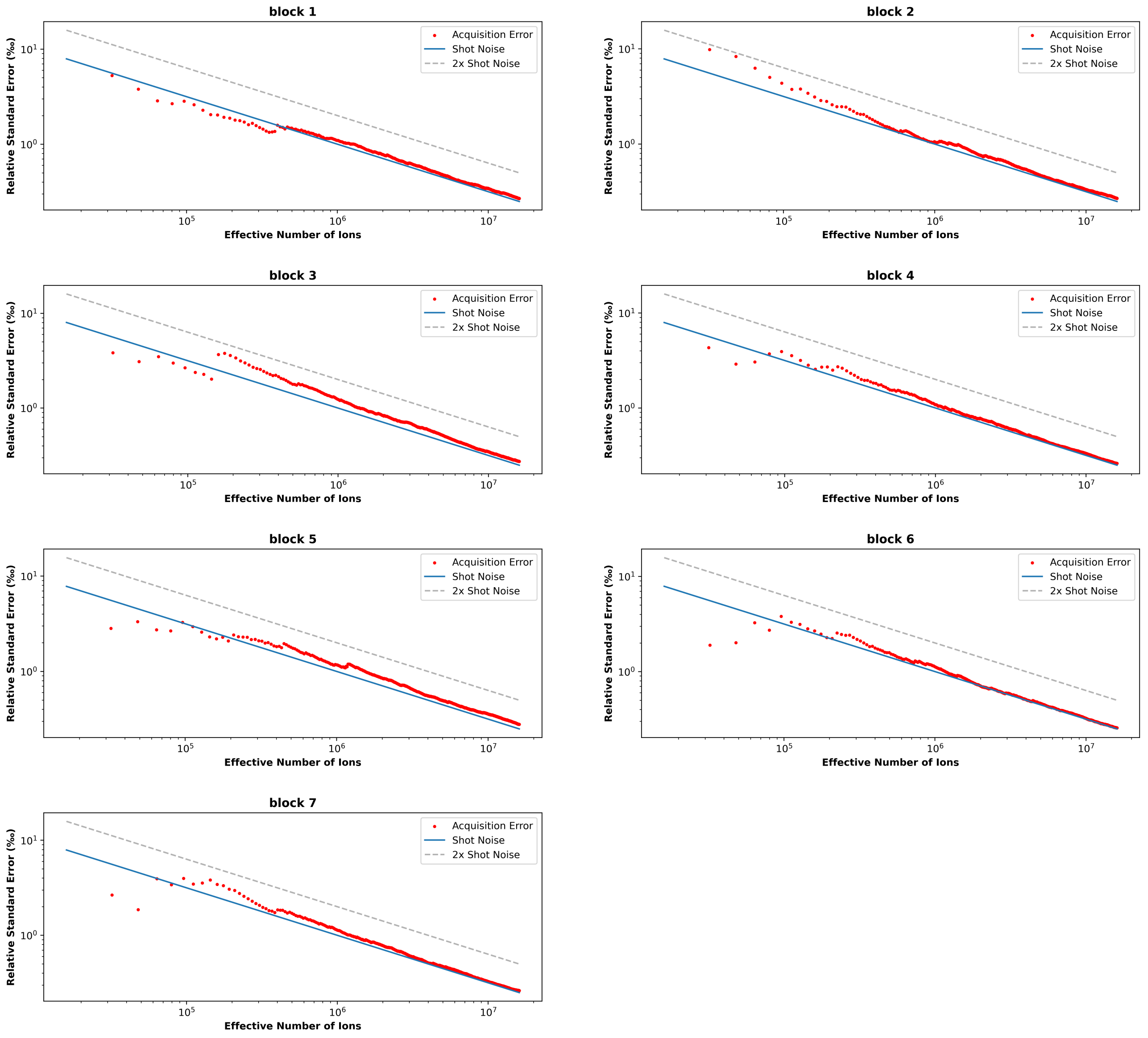

3. Delta Informations

Deltas were calculated by 'Average Of Neighboring Block Ratios'

3.1. 13C

Delta 13C was corrected by -27.80

Block	SEM	Delta corrected	Delta
2	0.24	-27.71	0.10
4	0.23	-28.92	-1.16
6	0.23	-28.84	-1.07

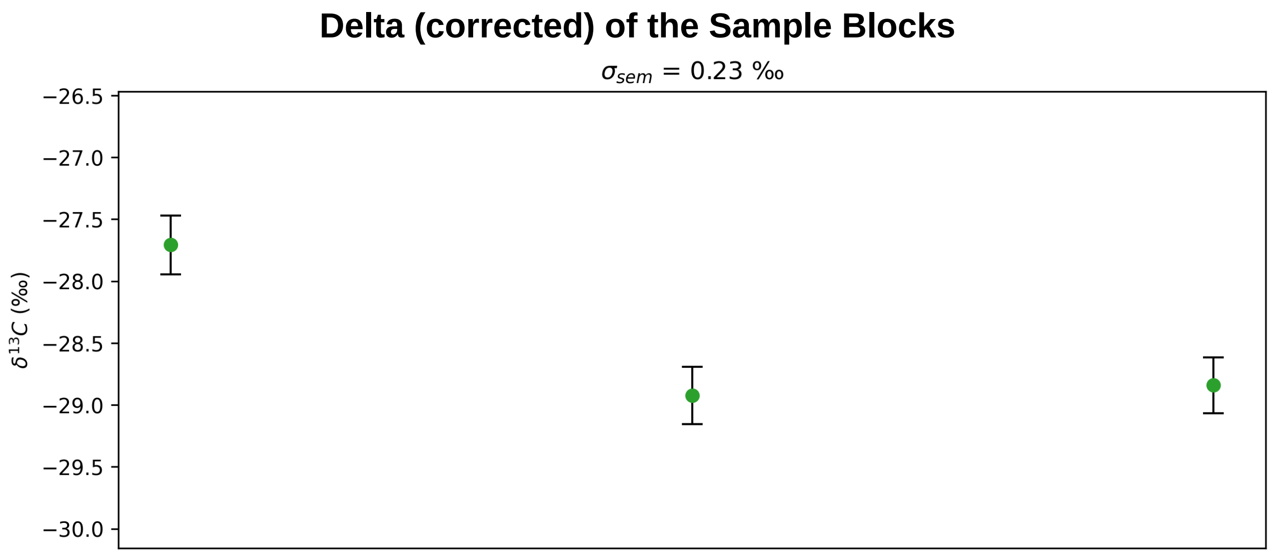

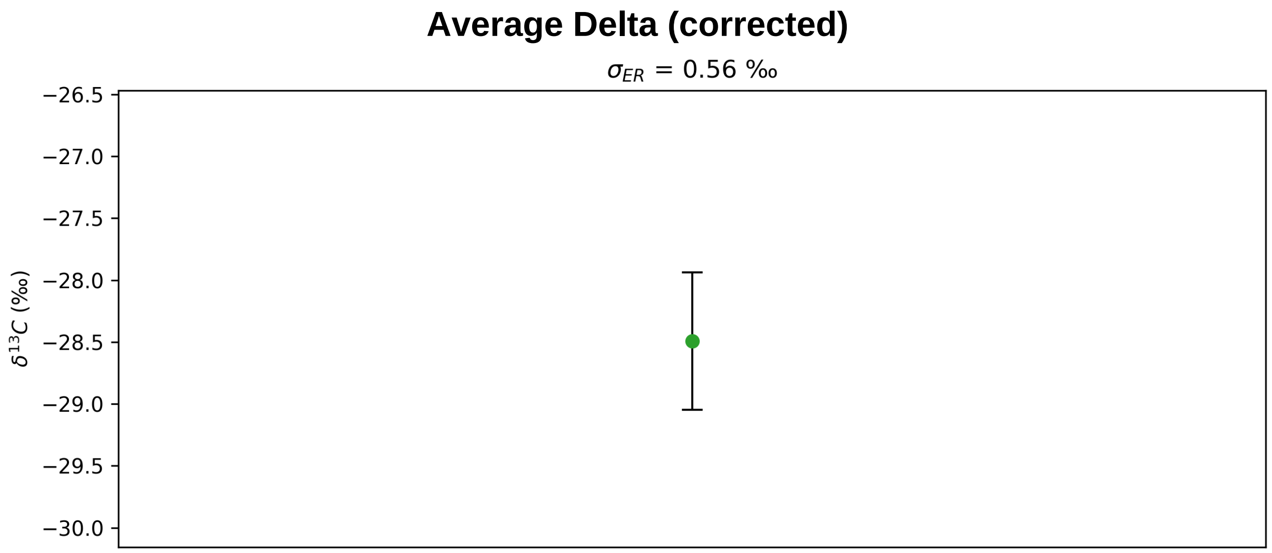

The final corrected average delta was -28.49 with a standard deviation of 0.56. Here the standard deviation is called reproducibility error.
