# Supplementary material for: Advancing Stable Isotope Analysis with Orbitrap-MS for Fatty Acid Methyl Esters and Complex Lipid Matrices
Source: J Am Soc Mass Spectrom. 2025 Jun 17;36(7):1527–35. doi: 10.1021/jasms.5c00092 (PMC12339014; doi:10.1021/jasms.5c00092)
Supplement: Supplementary file 2 [file js5c00092_si_002.zip › reports by IsotoPy Software/standards/H+Standard5_DI.pdf]

Standard 5 - [M + H]+
Isotope Analysis report from IsotoPy

Dual Inlet

1. Pre Processing

1.1. Block Time and Scan Information

Information about sample and standard block times and scans:

Block	Injected	Initial Time	End Time	Number of scans
1	standard	1	5	731
2	sample	6	10	760
3	standard	11	15	717
4	sample	16	20	714
5	standard	21	25	729
6	sample	26	30	744
7	standard	31	35	733

1.2. Outlier Removal

A total of 1180 scans were considered outliers and removed using the MAD method

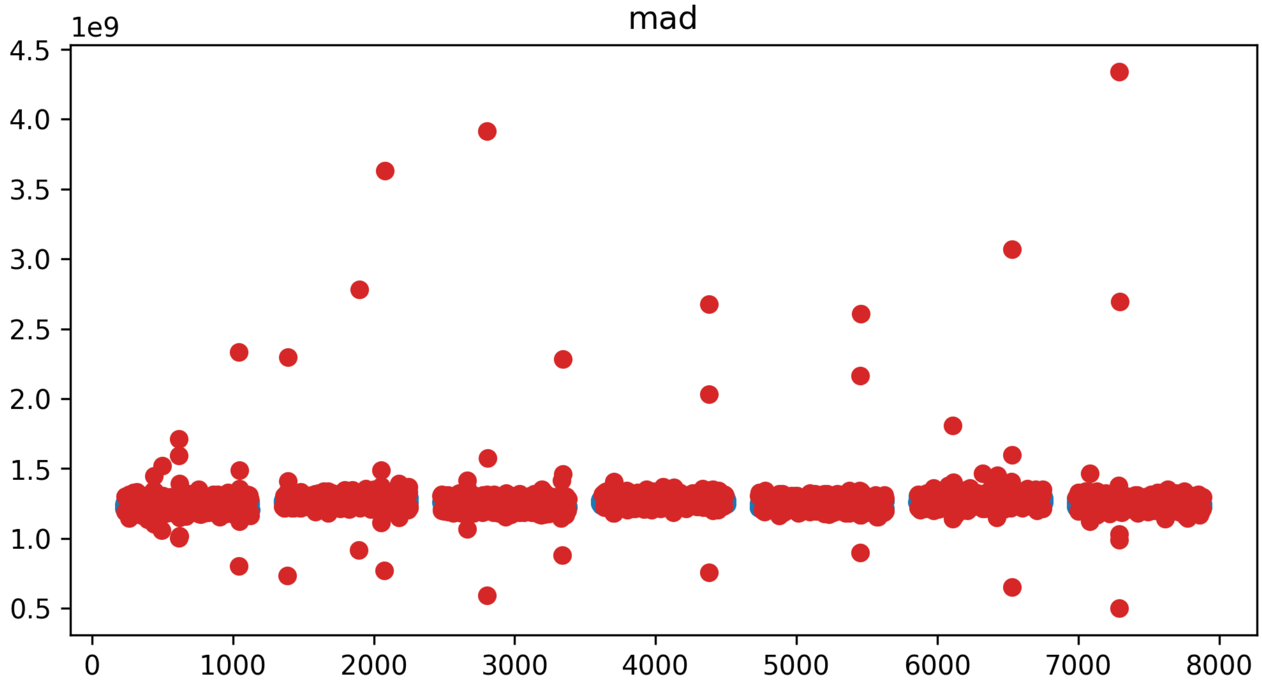

1.3. Total Ion Current (TIC)

TIC of all blocks

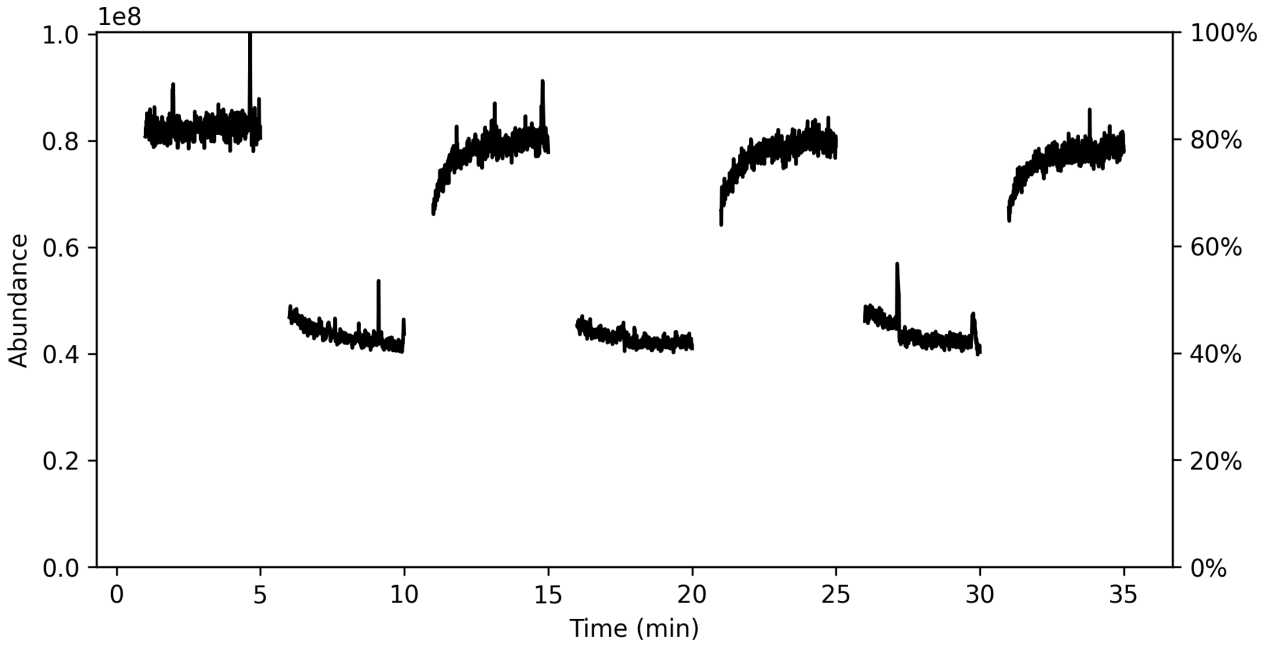

Block	TIC min	TIC max	TIC mean	RSD (%)
1	7.80e+07	1.00e+08	8.23e+07	2.05
2	4.03e+07	5.37e+07	4.35e+07	4.08
3	6.62e+07	9.12e+07	7.81e+07	4.20
4	4.02e+07	4.71e+07	4.29e+07	2.87
5	6.42e+07	8.43e+07	7.76e+07	4.01
6	3.98e+07	5.69e+07	4.38e+07	5.05
7	6.49e+07	8.58e+07	7.65e+07	3.69

2. Block Parameters

The Isotopic Ratio of the blocks were calculated by 'Mean'

2.1. 13C/M0

Block	Number of scans	Effective number of ions	Isotopic Ratio	STD	SEM	RSE
1	731	1.32e+07	0.217317	0.001317	0.000049	0.000224
2	760	1.38e+07	0.217090	0.001322	0.000048	0.000221
3	717	1.30e+07	0.216972	0.001422	0.000053	0.000245
4	714	1.30e+07	0.216897	0.001385	0.000052	0.000239
5	729	1.34e+07	0.217039	0.001427	0.000053	0.000243
6	744	1.37e+07	0.217082	0.001350	0.000049	0.000228
7	733	1.35e+07	0.217002	0.001334	0.000049	0.000227

Errors and Test Paramters

Block	Acquisition Error (permil)	Shot-Noise (permil)	AE/SN ratio	Shapiro Wilk (p_value)	D'Agostino (p_value)
1	0.224	0.275	0.814	0.330	0.243
2	0.221	0.269	0.819	0.374	0.332
3	0.245	0.277	0.883	0.921	0.792
4	0.239	0.277	0.862	0.357	0.551
5	0.243	0.274	0.890	0.988	0.964
6	0.228	0.271	0.842	0.115	0.230
7	0.227	0.272	0.834	0.538	0.305

Isotopic Ratio and Errors of the Blocks

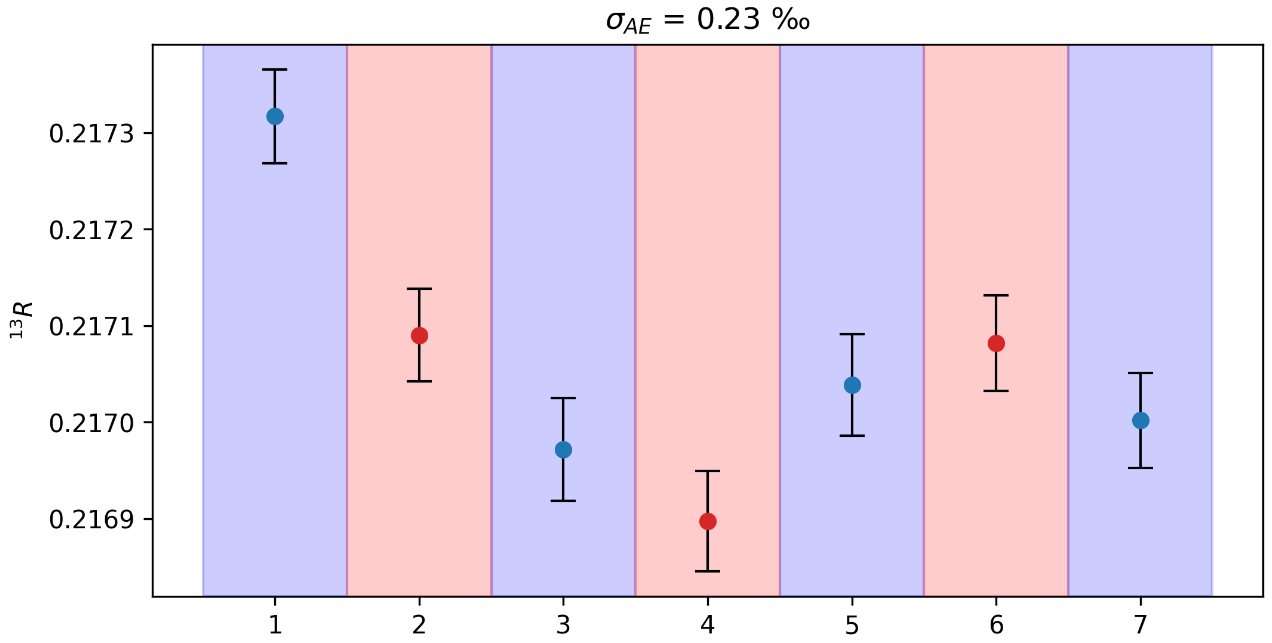

Cumulative Isotopic Ratio

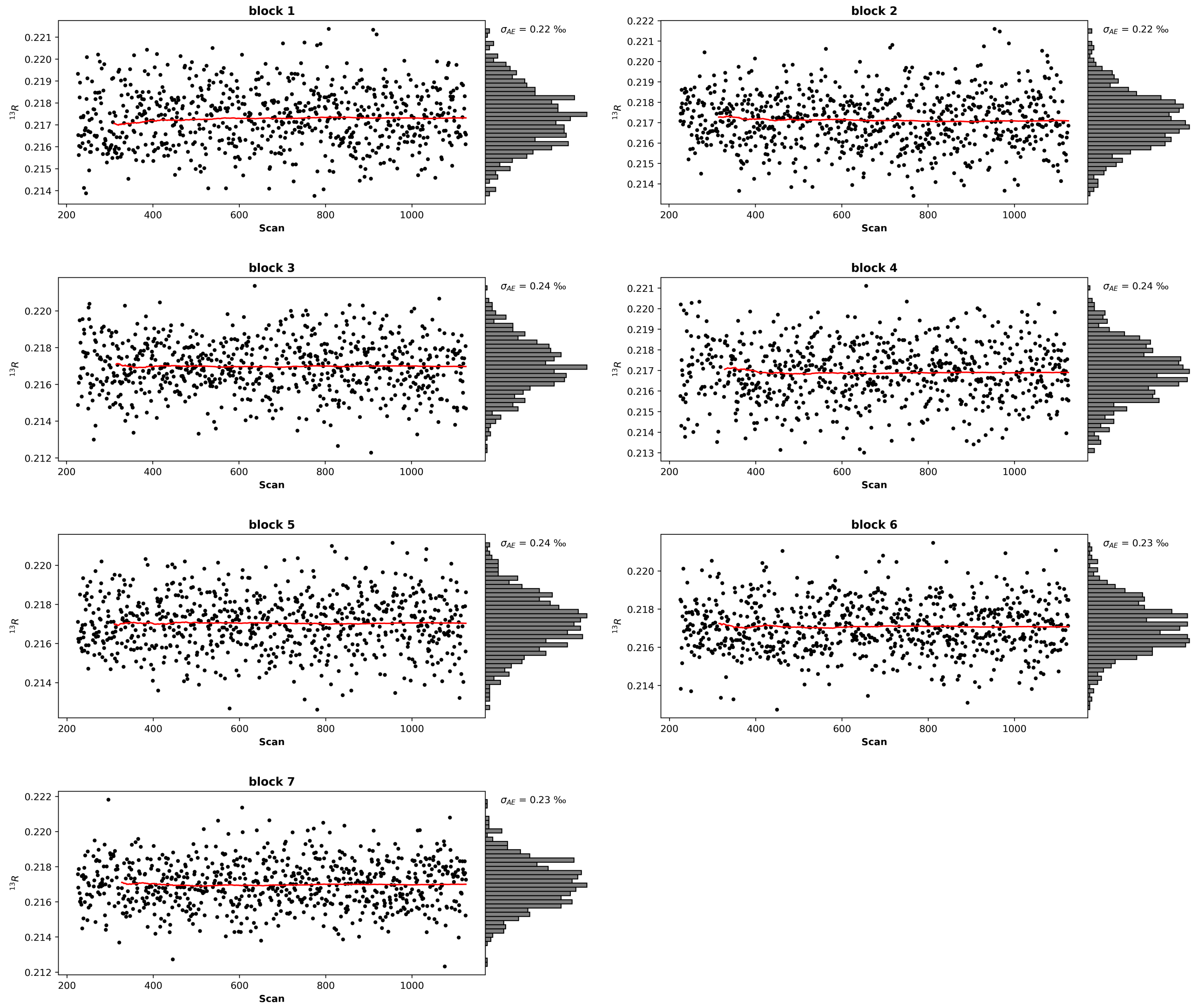

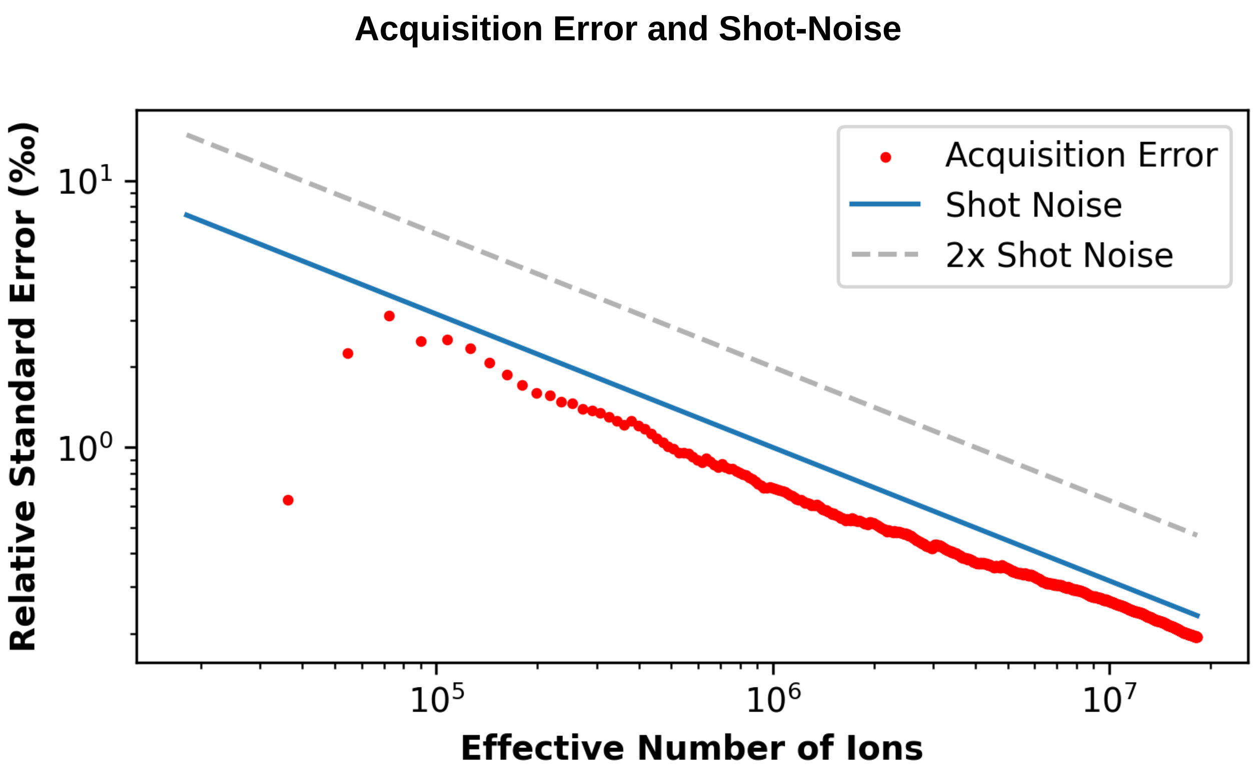
Acquisition Error and Shot-Noise
Relative Standard Error (‰)
10^1
10^0
Effective Number of Ions
10^5
10^6
10^7
Acquisition Error
Shot Noise
2x Shot Noise

3. Delta Informations

Deltas were calculated by 'Average Of Neighboring Block Ratios'

3.1. 13C

Delta 13C was corrected by -27.80

Block	SEM	Delta corrected	Delta
2	0.22	-28.04	-0.25
4	0.24	-28.28	-0.50
6	0.23	-27.52	0.28

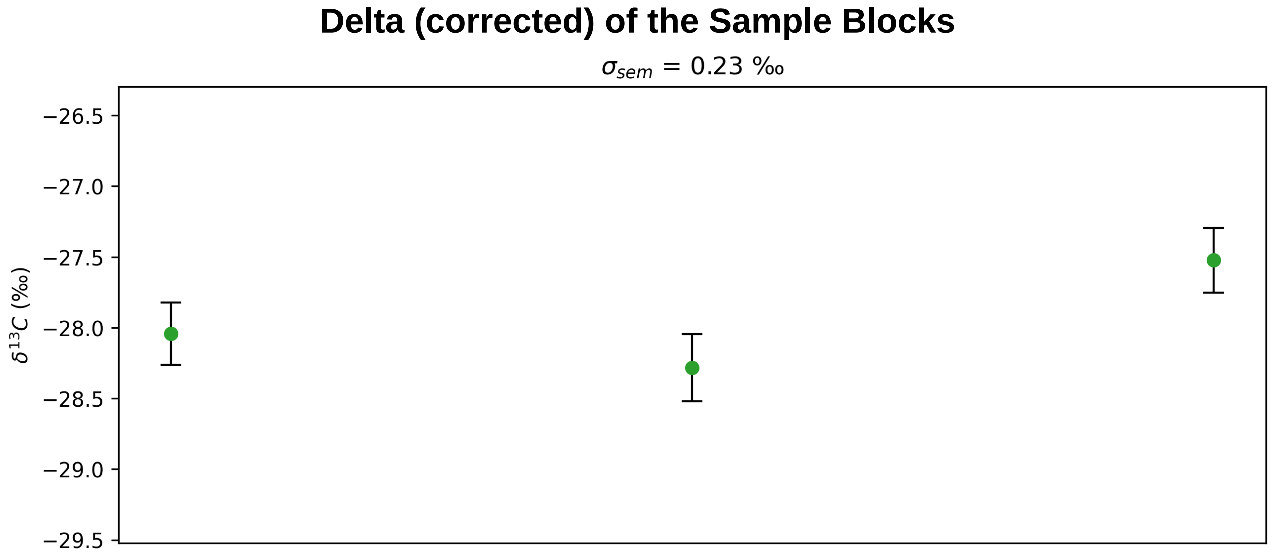

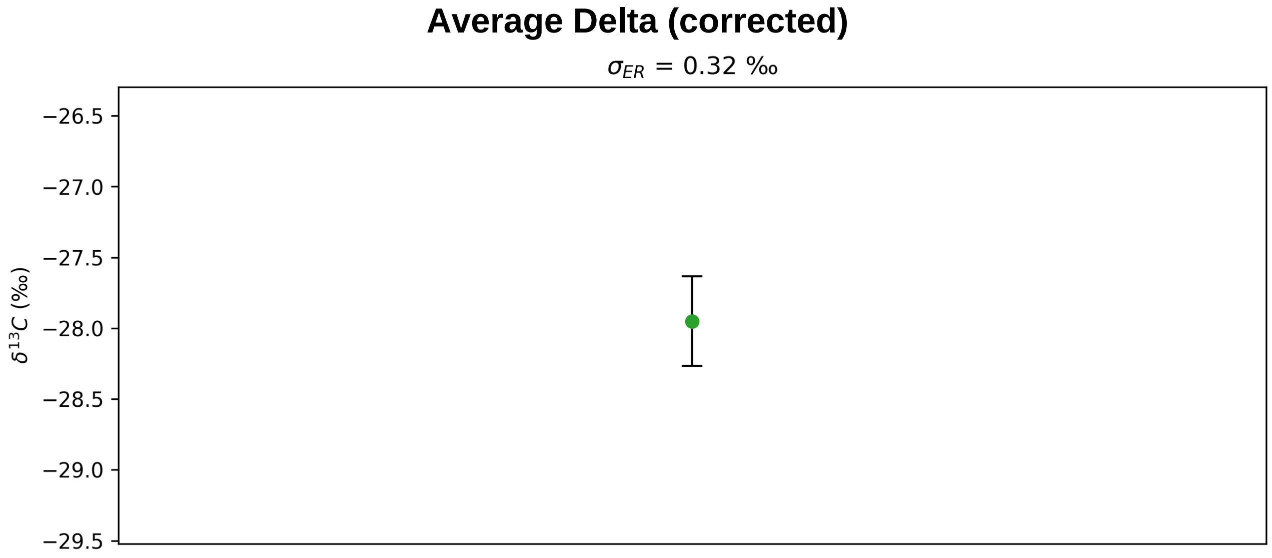

The final corrected average delta was -27.95 with a standard deviation of 0.32. Here the standard deviation is called reproducibility error.
