# Supplementary material for: Advancing Stable Isotope Analysis with Orbitrap-MS for Fatty Acid Methyl Esters and Complex Lipid Matrices
Source: J Am Soc Mass Spectrom. 2025 Jun 17;36(7):1527–35. doi: 10.1021/jasms.5c00092 (PMC12339014; doi:10.1021/jasms.5c00092)
Supplement: Supplementary file 2 [file js5c00092_si_002.zip › reports by IsotoPy Software/standards/H+Standard9_DI.pdf]

Standard 9 - [M + H]+
Isotope Analysis report from IsotoPy
Dual Inlet

1. Pre Processing

1.1. Block Time and Scan Information

Information about sample and standard block times and scans:

Block	Injected	Initial Time	End Time	Number of scans
1	standard	1	5	723
2	sample	6	10	752
3	standard	11	15	742
4	sample	16	20	746
5	standard	21	25	759
6	sample	26	30	750
7	standard	31	35	750

1.2. Outlier Removal

A total of 1086 scans were considered outliers and removed using the MAD method

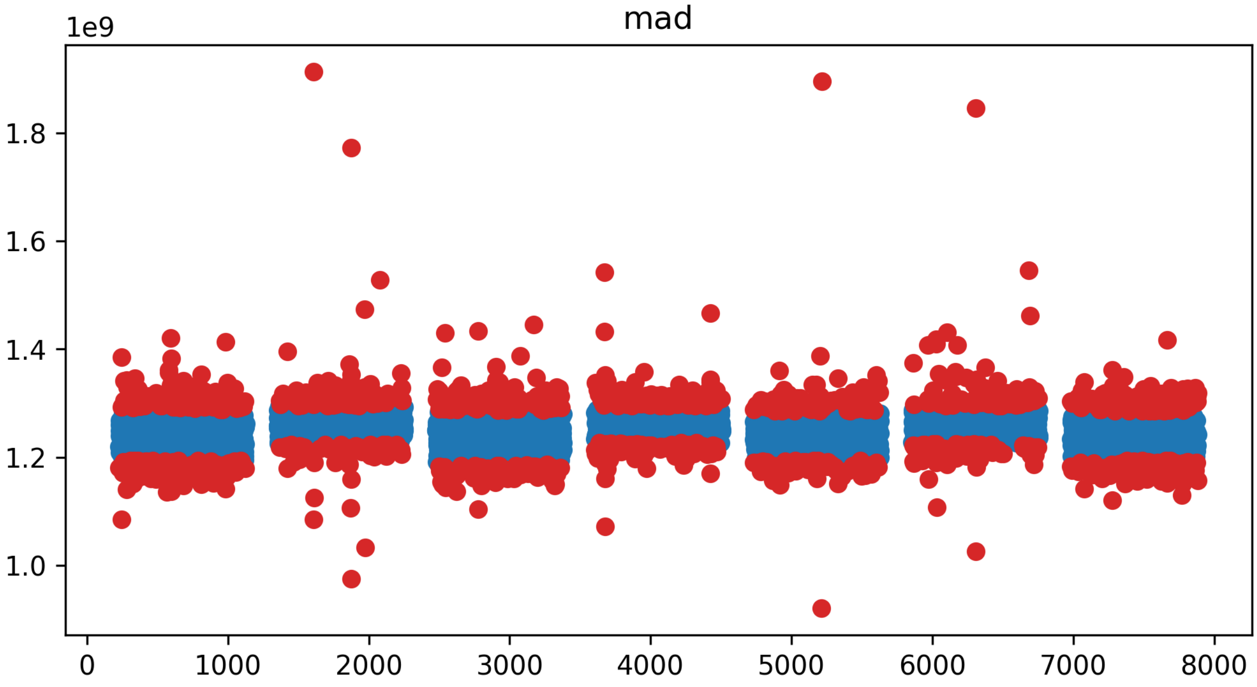

1.3. Total Ion Current (TIC)

TIC of all blocks

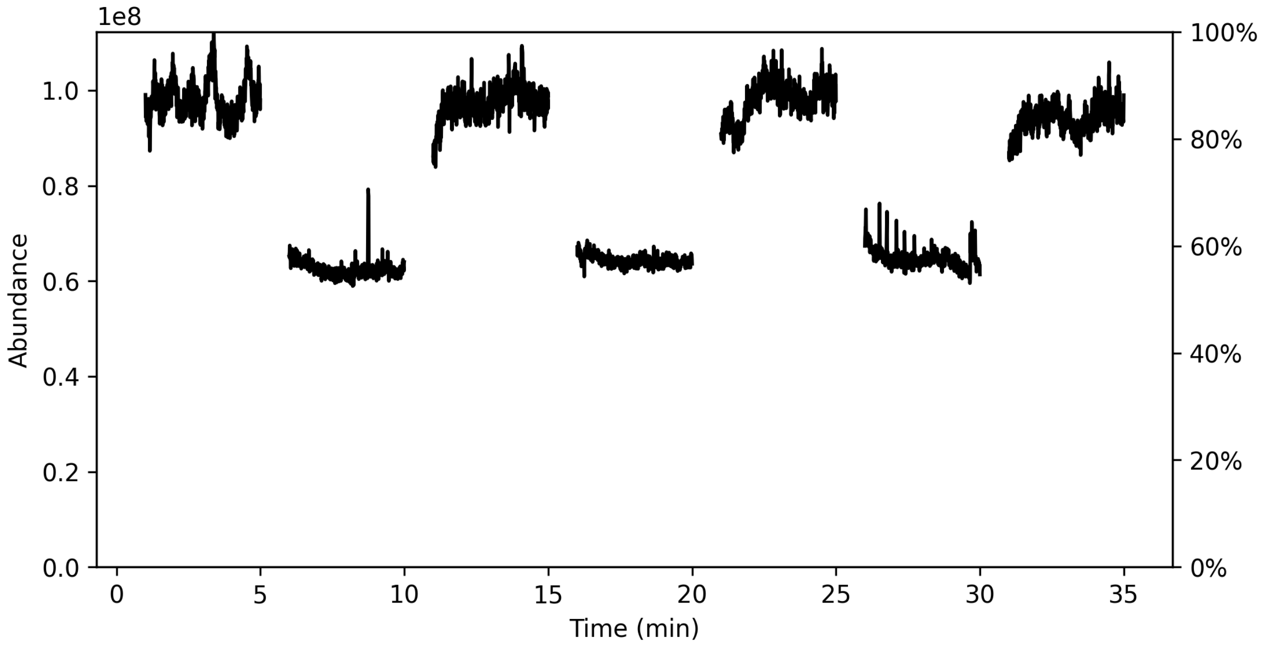

Block	TIC min	TIC max	TIC mean	RSD (%)
1	8.73e+07	1.12e+08	9.83e+07	4.05
2	5.89e+07	7.92e+07	6.25e+07	2.87
3	8.39e+07	1.09e+08	9.74e+07	3.54
4	6.09e+07	6.85e+07	6.43e+07	1.71
5	8.69e+07	1.09e+08	9.80e+07	4.03
6	5.95e+07	7.63e+07	6.46e+07	2.97
7	8.53e+07	1.06e+08	9.41e+07	3.24

2. Block Parameters

The Isotopic Ratio of the blocks were calculated by 'Mean'

2.1. 13C/M0

Block	Number of scans	Effective number of ions	Isotopic Ratio	STD	SEM	RSE
1	723	1.20e+07	0.217815	0.001346	0.000050	0.000230
2	752	1.24e+07	0.217403	0.001433	0.000052	0.000240
3	742	1.24e+07	0.217844	0.001363	0.000050	0.000230
4	746	1.24e+07	0.217245	0.001356	0.000050	0.000228
5	759	1.28e+07	0.217627	0.001345	0.000049	0.000224
6	750	1.26e+07	0.217318	0.001391	0.000051	0.000234
7	750	1.28e+07	0.217660	0.001333	0.000049	0.000223

Errors and Test Paramters

Block	Acquisition Error (permil)	Shot-Noise (permil)	AE/SN ratio	Shapiro Wilk (p_value)	D'Agostino (p_value)
1	0.230	0.289	0.796	0.381	0.211
2	0.240	0.284	0.845	0.838	0.779
3	0.230	0.284	0.807	0.723	0.480
4	0.228	0.284	0.804	0.702	0.398
5	0.224	0.279	0.803	0.389	0.230
6	0.234	0.281	0.830	0.137	0.127
7	0.223	0.280	0.799	0.708	0.617

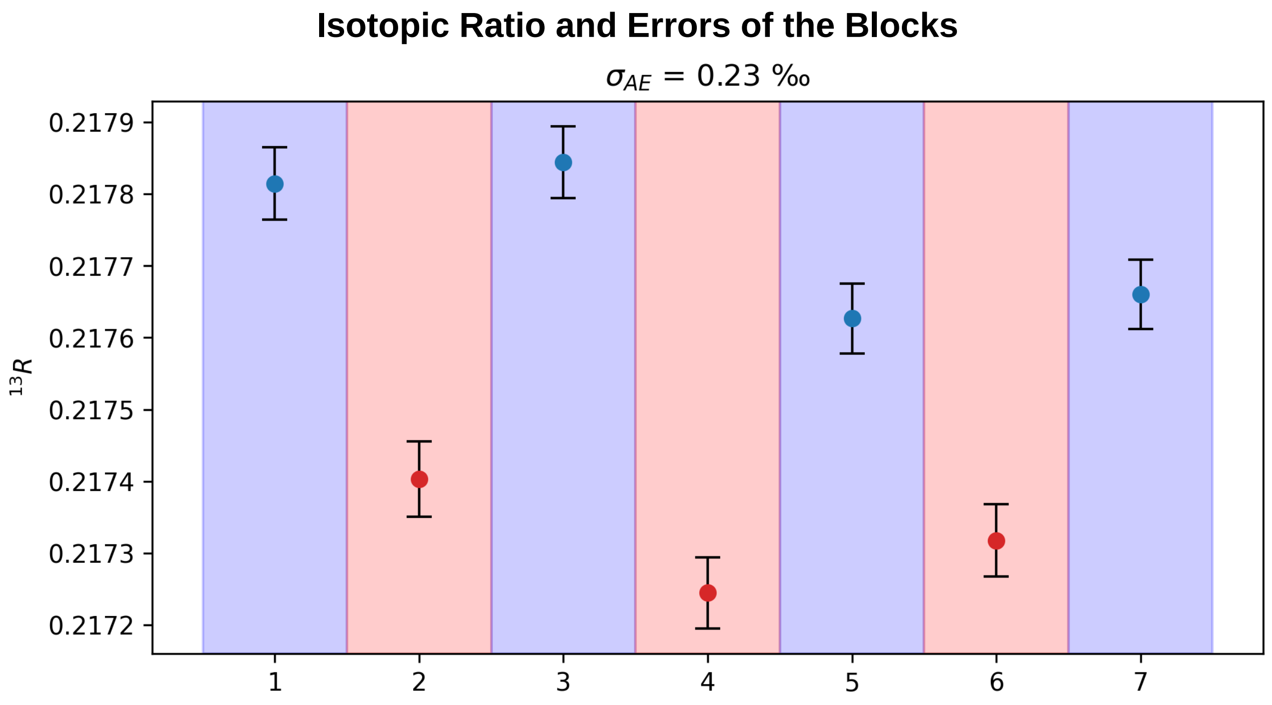
Isotopic Ratio and Errors of the Blocks
σAE = 0.23 ‰
13R
0.2179
0.2178
0.2177
0.2176
0.2175
0.2174
0.2173
0.2172
1
2
3
4
5
6
7

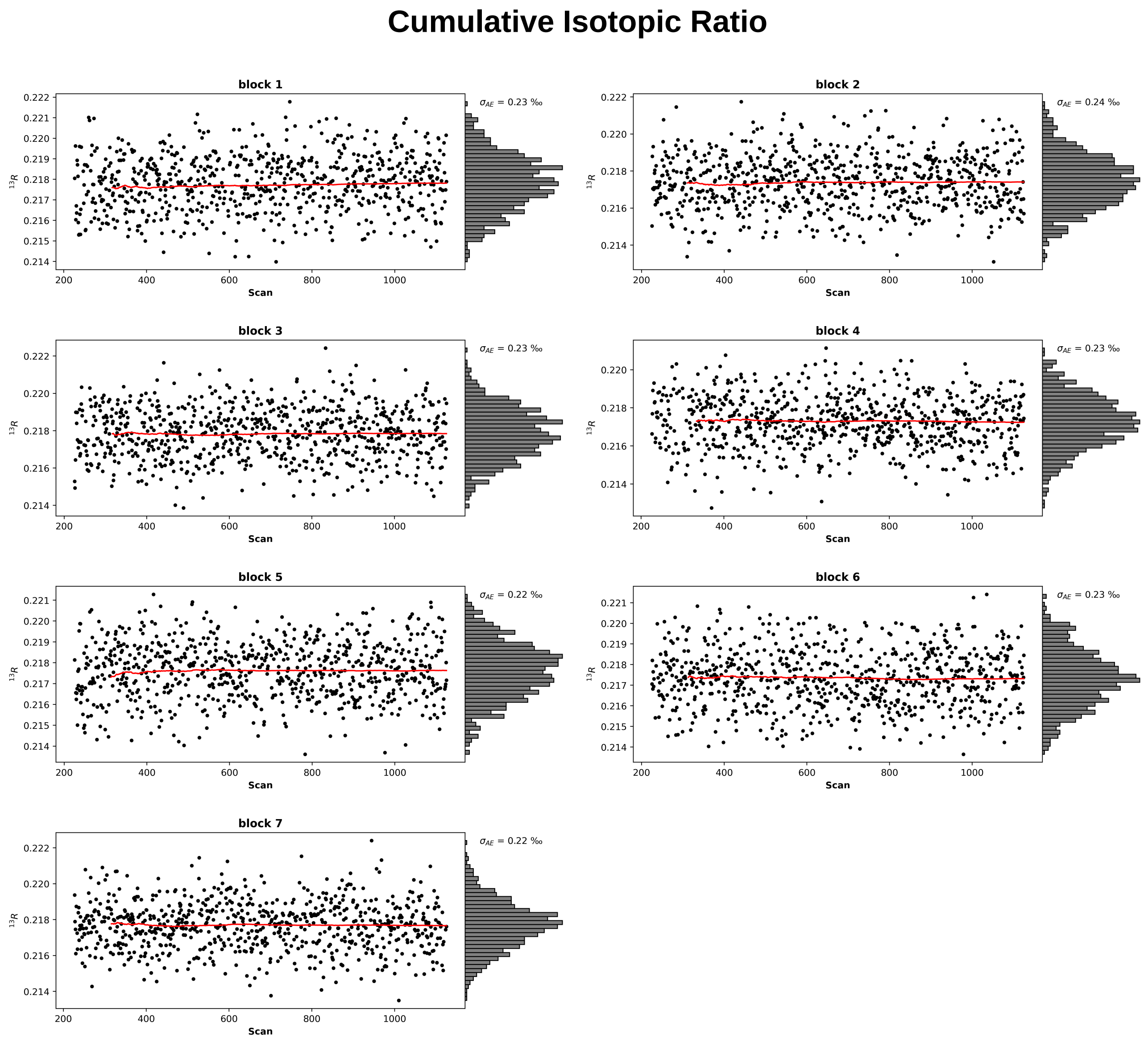
Cumulative Isotopic Ratio
block 1
σAE = 0.23 ‰
block 2
σAE = 0.24 ‰
block 3
σAE = 0.23 ‰
block 4
σAE = 0.23 ‰
block 5
σAE = 0.22 ‰
block 6
σAE = 0.23 ‰
block 7
σAE = 0.22 ‰
13R
Scan

Acquisition Error and Shot-Noise

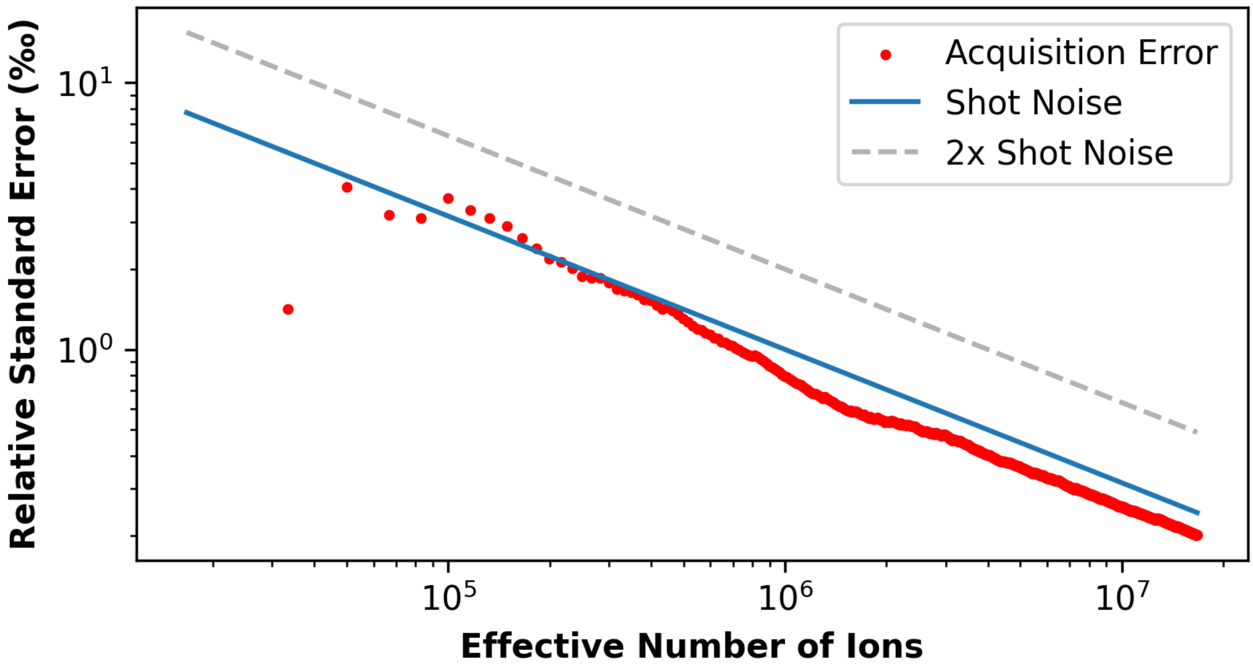

3. Delta Informations

Deltas were calculated by 'Average Of Neighboring Block Ratios'

3.1. 13C

Delta 13C was corrected by -27.80

Block	SEM	Delta corrected	Delta
2	0.24	-29.70	-1.96
4	0.23	-29.99	-2.26
6	0.23	-29.26	-1.50

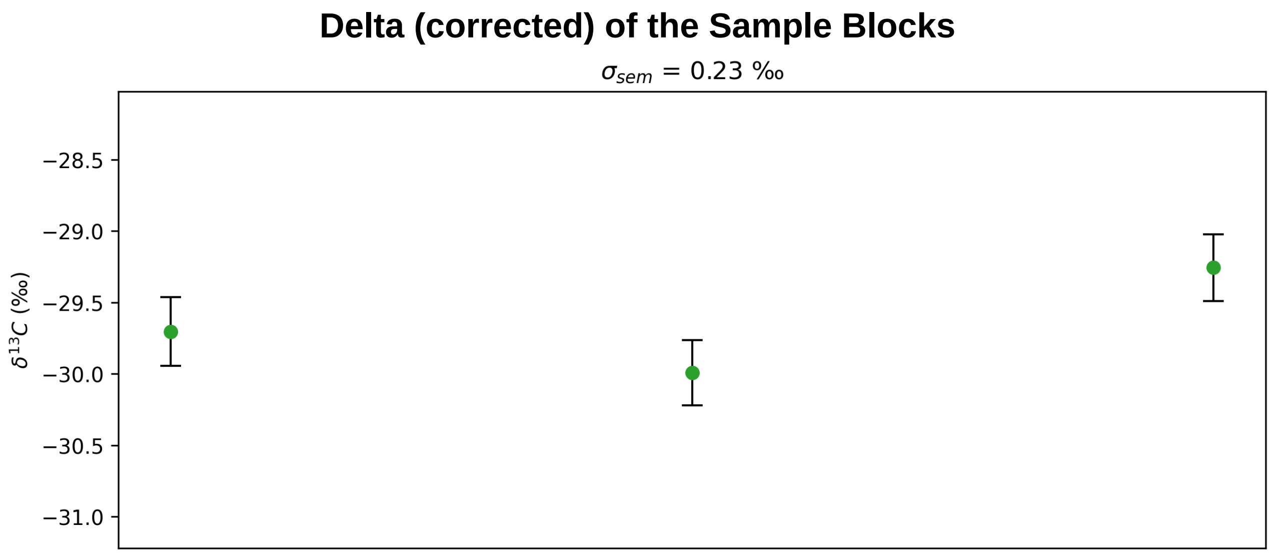

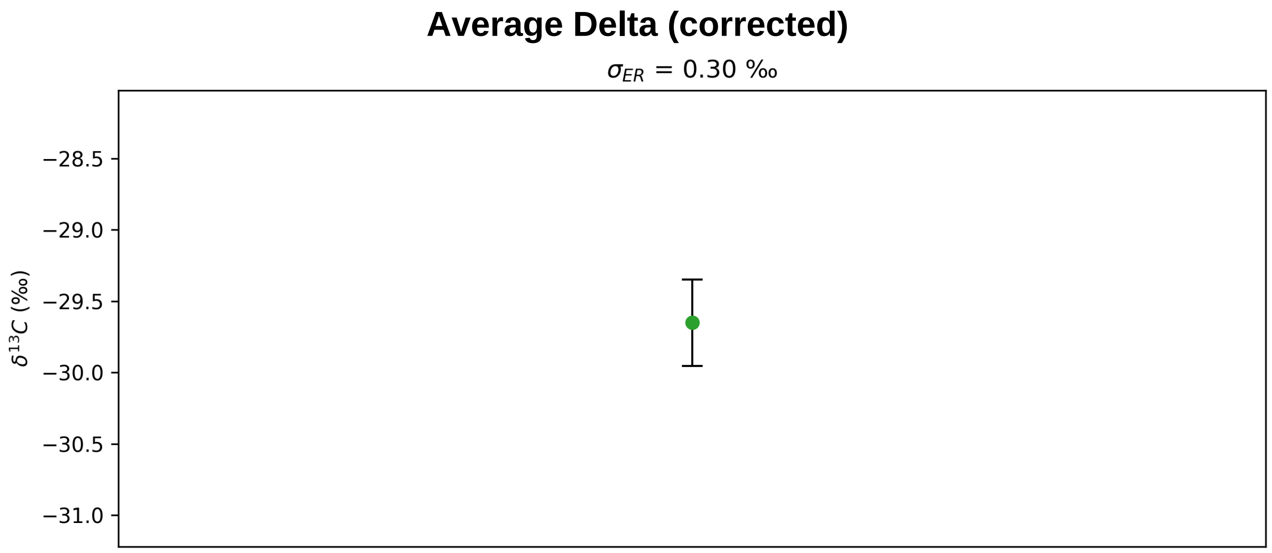

The final corrected average delta was -29.65 with a standard deviation of 0.30. Here the standard deviation is called reproducibility error.
