# Supplementary material for: Advancing Stable Isotope Analysis with Orbitrap-MS for Fatty Acid Methyl Esters and Complex Lipid Matrices
Source: J Am Soc Mass Spectrom. 2025 Jun 17;36(7):1527–35. doi: 10.1021/jasms.5c00092 (PMC12339014; doi:10.1021/jasms.5c00092)
Supplement: Supplementary file 2 [file js5c00092_si_002.zip › reports by IsotoPy Software/standards/H+Standard2_FI.pdf]

Standard 2 - [M + H]+

Isotope Analysis report from IsotoPy

Flow Injection

1. Pre Processing

1.1. Block Time and Scan Information

Information about sample and standard block times and scans:

Block	Injected	Initial Time	End Time	Number of scans
1	standard	1	8	1283
2	sample	16	23	1279
3	standard	31	38	1350
4	sample	46	53	1392
5	standard	61	68	1325
6	sample	76	83	1304
7	standard	91	98	1298

1.2. Outlier Removal

A total of 1808 scans were considered outliers and removed using the MAD method

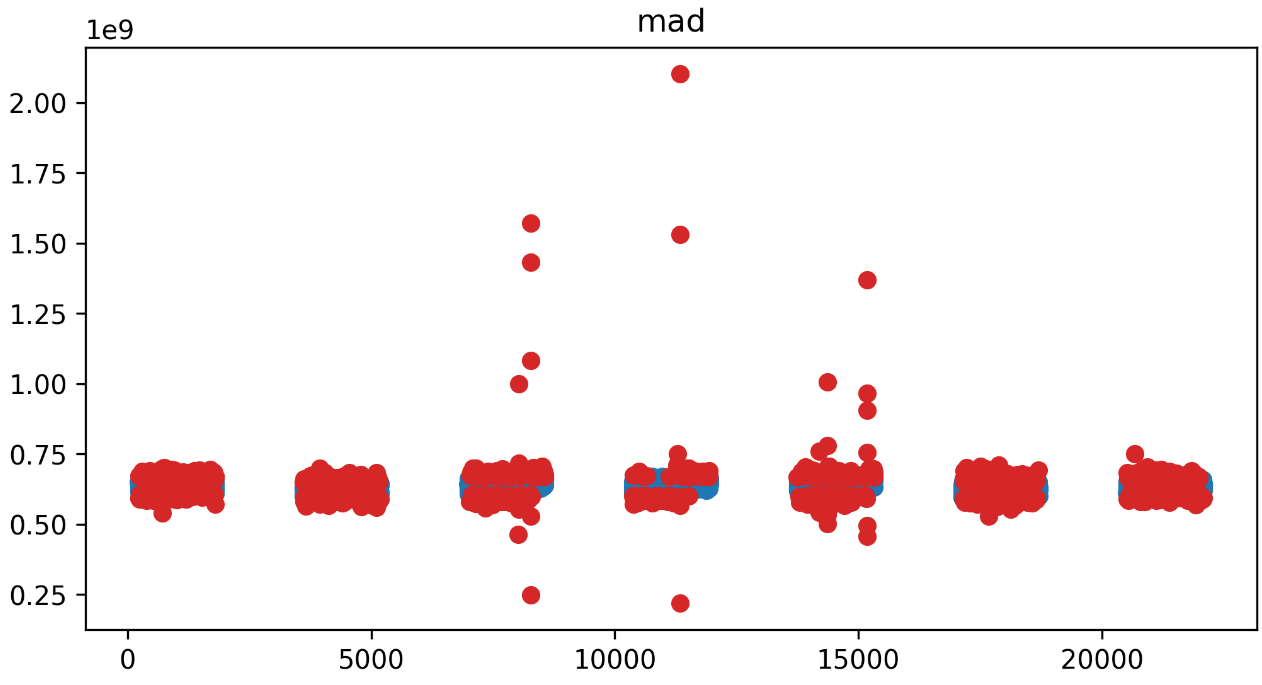

1.3. Total Ion Current (TIC)

TIC of all blocks

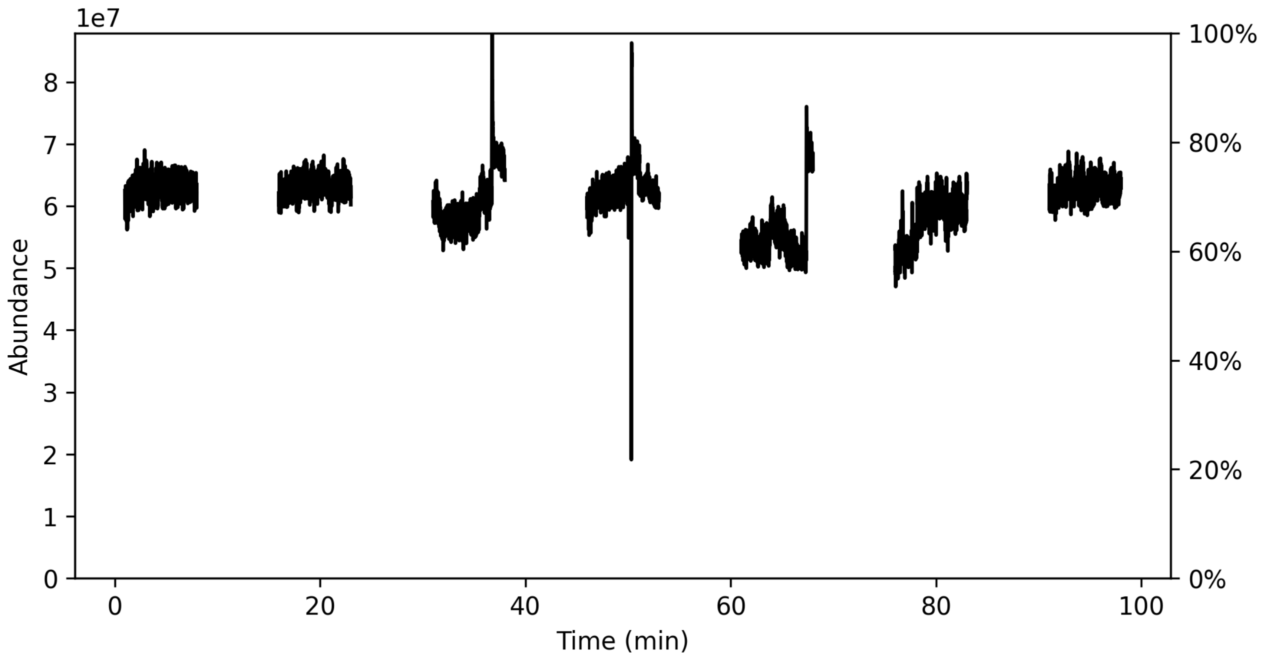

Block	TIC min	TIC max	TIC mean	RSD (%)
1	5.62e+07	6.90e+07	6.28e+07	2.76
2	5.89e+07	6.82e+07	6.32e+07	2.43
3	5.28e+07	8.78e+07	6.02e+07	7.28
4	1.91e+07	8.63e+07	6.29e+07	4.63
5	4.93e+07	7.60e+07	5.50e+07	7.96
6	4.70e+07	6.53e+07	5.76e+07	6.37
7	5.77e+07	6.88e+07	6.29e+07	2.54

2. Block Parameters

The Isotopic Ratio of the blocks were calculated by 'Mean'

2.1. 13C/M0

Block	Number of scans	Effective number of ions	Isotopic Ratio	STD	SEM	RSE
1	1283	1.72e+07	0.209937	0.001724	0.000048	0.000229
2	1279	1.70e+07	0.209637	0.001716	0.000048	0.000229
3	1350	1.81e+07	0.209888	0.001770	0.000048	0.000229
4	1392	1.85e+07	0.209218	0.001765	0.000047	0.000226
5	1325	1.77e+07	0.210020	0.001748	0.000048	0.000229
6	1304	1.74e+07	0.209646	0.001678	0.000046	0.000222
7	1298	1.73e+07	0.209855	0.001696	0.000047	0.000224

Errors and Test Paramters

Block	Acquisition Error (permil)	Shot-Noise (permil)	AE/SN ratio	Shapiro Wilk (p_value)	D'Agostino (p_value)
1	0.229	0.241	0.950	0.701	0.442
2	0.229	0.242	0.943	0.113	0.108
3	0.229	0.235	0.975	0.020	0.012
4	0.226	0.232	0.973	0.771	0.576
5	0.229	0.238	0.962	0.249	0.747
6	0.222	0.240	0.923	0.880	0.832
7	0.224	0.240	0.934	0.925	0.930

Isotopic Ratio and Errors of the Blocks

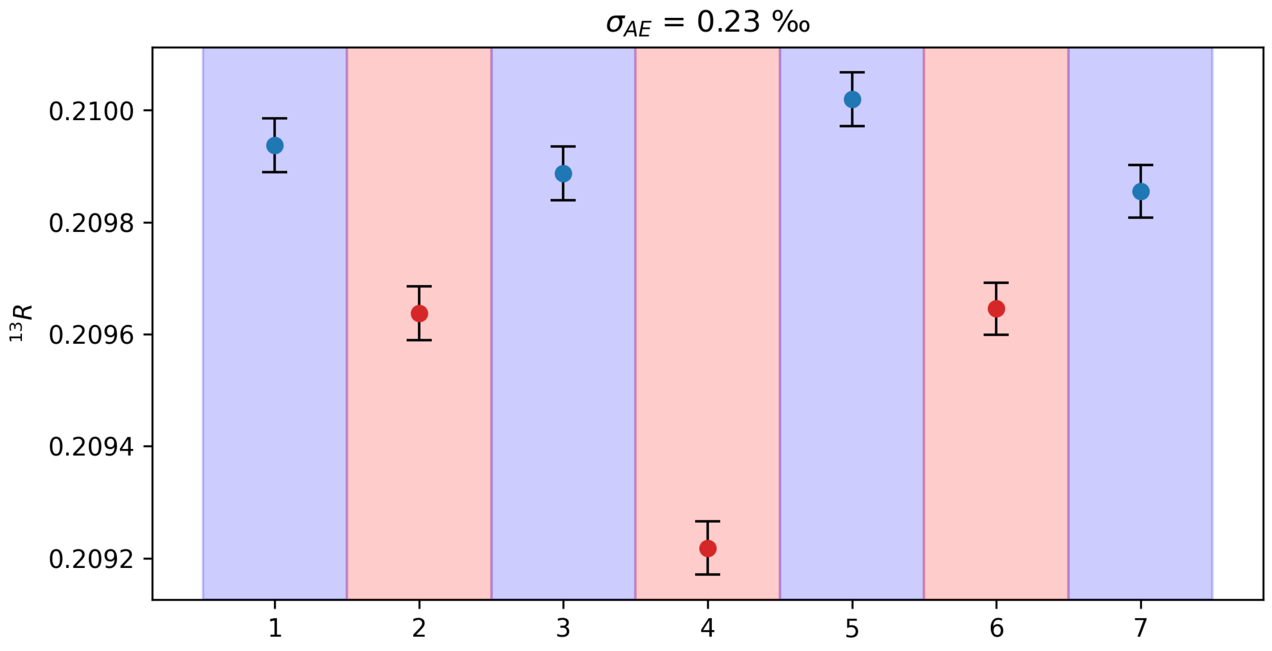

Cumulative Isotopic Ratio

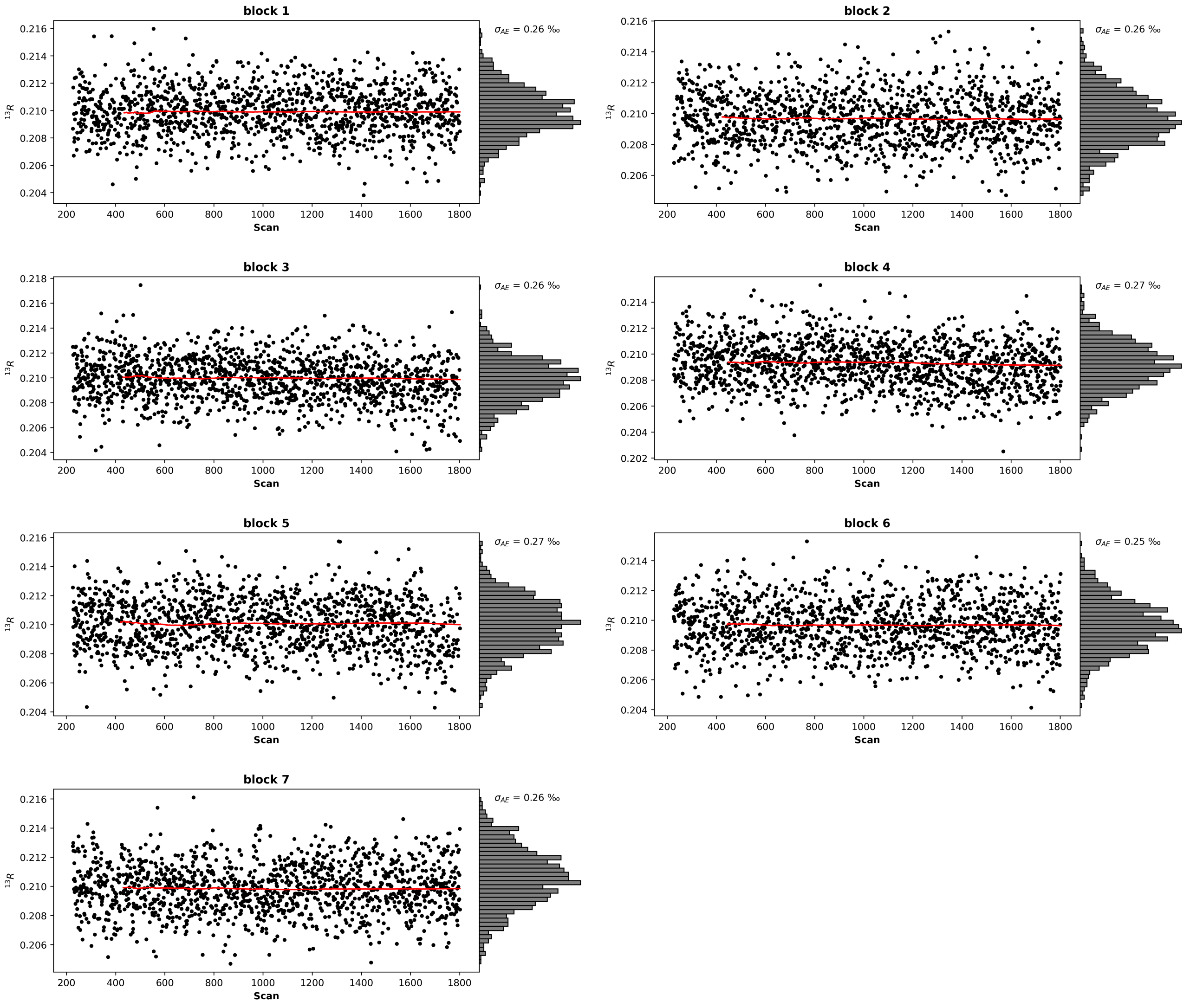

Acquisition Error and Shot-Noise

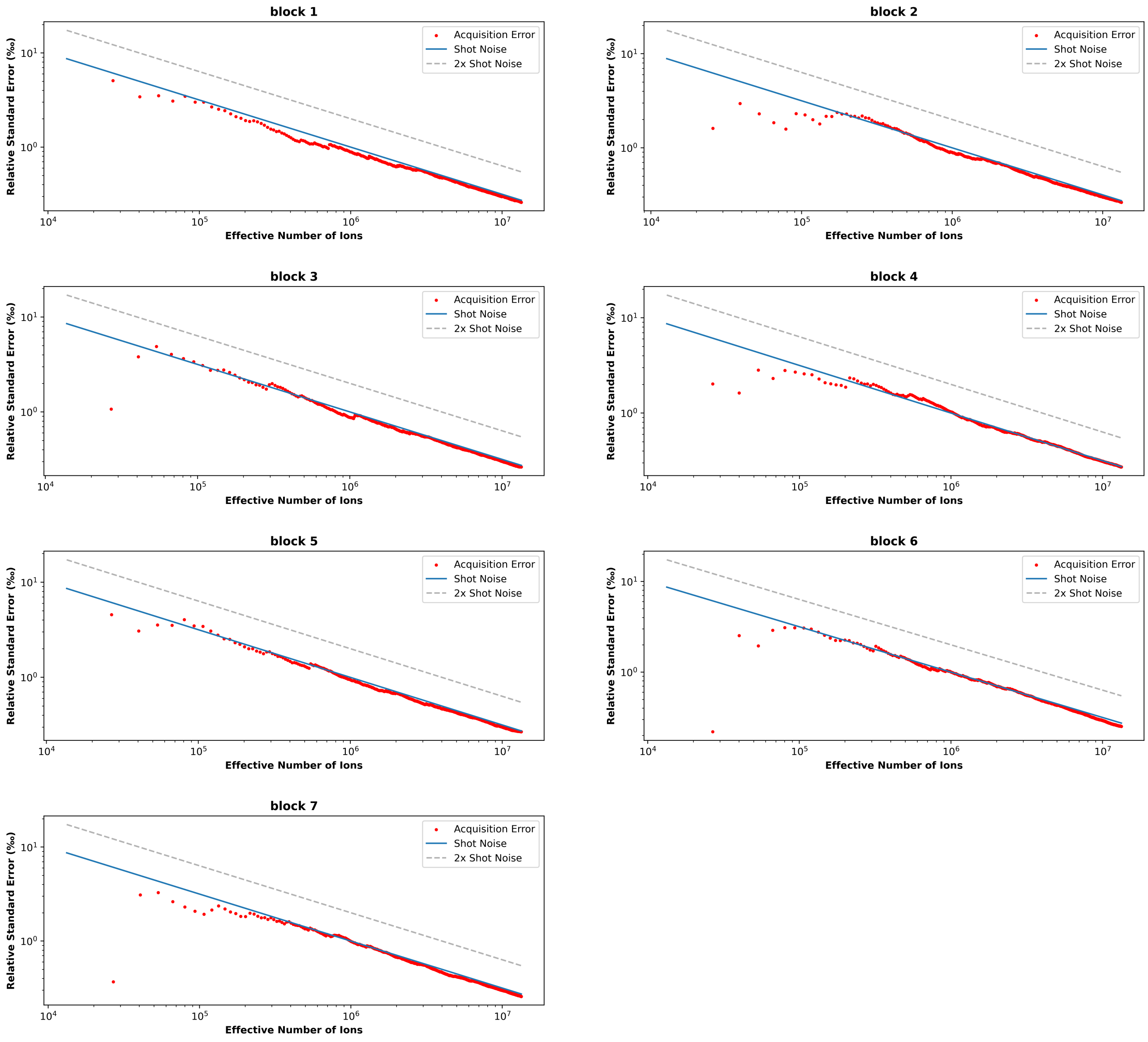

3. Delta Informations

Deltas were calculated by 'Average Of Neighboring Block Ratios'

3.1. 13C

Delta 13C was corrected by -27.80

Block	SEM	Delta corrected	Delta
2	0.23	-29.08	-1.31
4	0.23	-31.21	-3.50
6	0.22	-29.15	-1.39

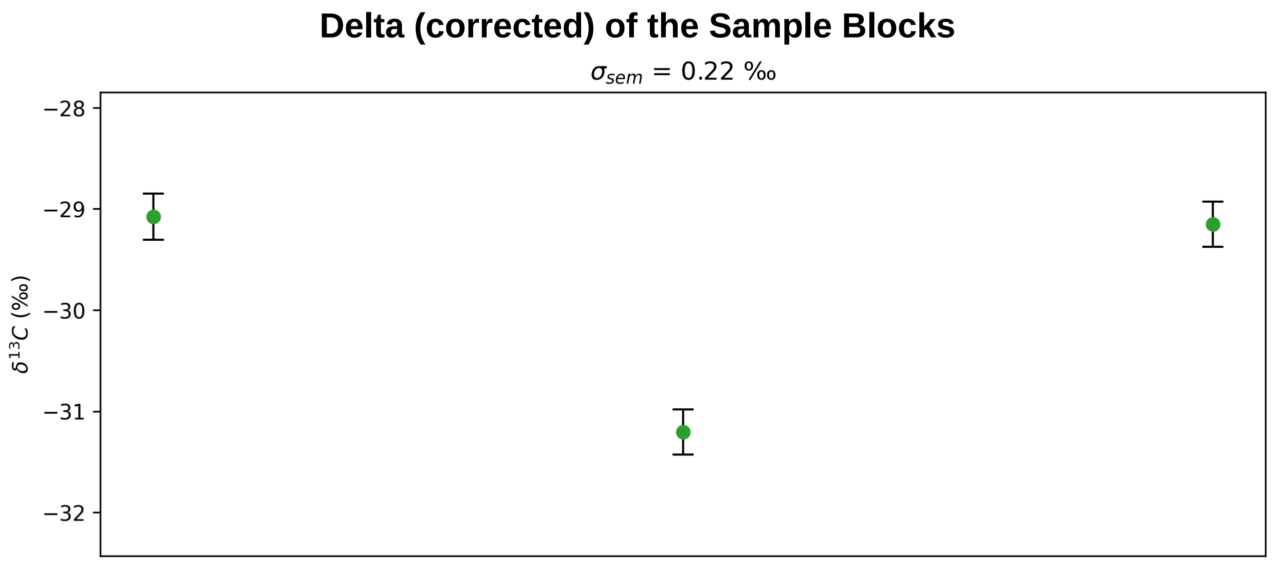

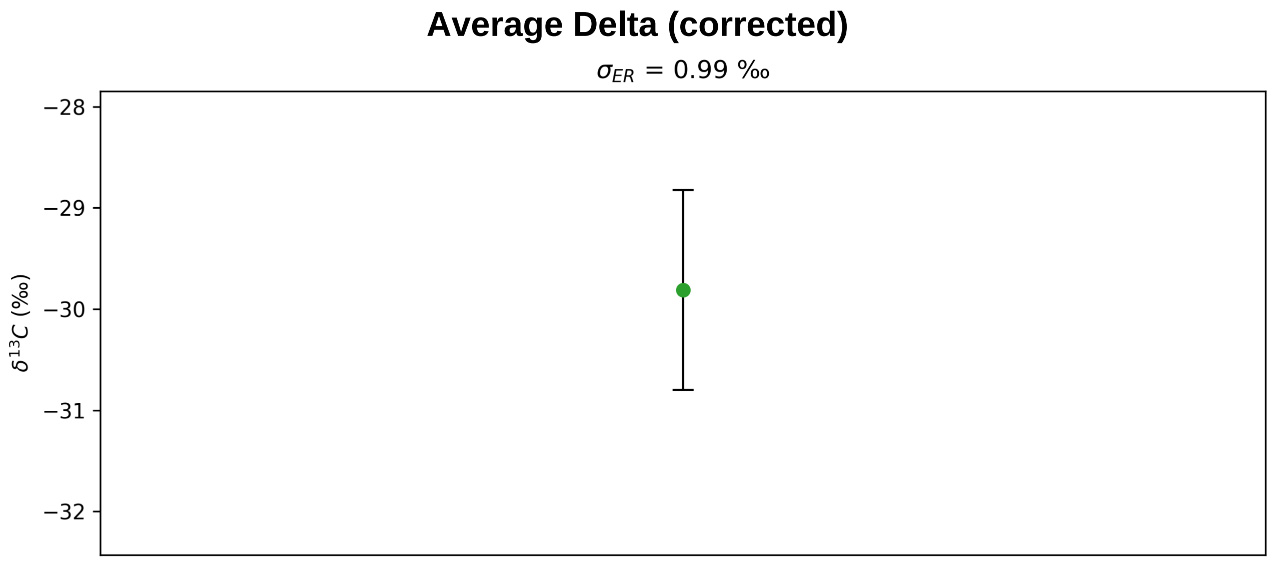

The final corrected average delta was -29.81 with a standard deviation of 0.99. Here the standard deviation is called reproducibility error.
