# Supplementary material for: Advancing Stable Isotope Analysis with Orbitrap-MS for Fatty Acid Methyl Esters and Complex Lipid Matrices
Source: J Am Soc Mass Spectrom. 2025 Jun 17;36(7):1527–35. doi: 10.1021/jasms.5c00092 (PMC12339014; doi:10.1021/jasms.5c00092)
Supplement: Supplementary file 2 [file js5c00092_si_002.zip › reports by IsotoPy Software/standards/Na+Standard3_FI.pdf]

Standard 3 - [M + Na]+
Isotope Analysis report from IsotoPy

Flow Injection

1. Pre Processing

1.1. Block Time and Scan Information

Information about sample and standard block times and scans:

Block	Injected	Initial Time	End Time	Number of scans
1	standard	1	8	1280
2	sample	16	23	1254
3	standard	31	38	1296
4	sample	46	53	1302
5	standard	61	68	1281
6	sample	76	83	1296
7	standard	91	98	1278

1.2. Outlier Removal

A total of 2052 scans were considered outliers and removed using the MAD method

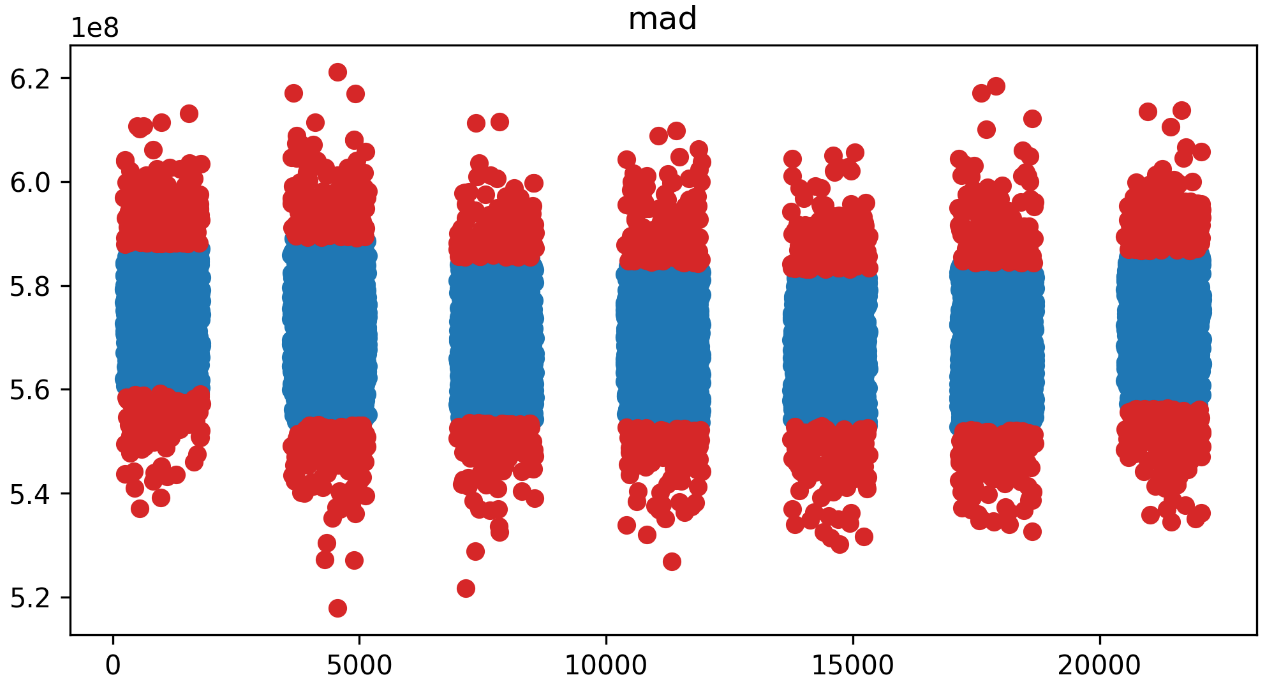

1.3. Total Ion Current (TIC)

TIC of all blocks

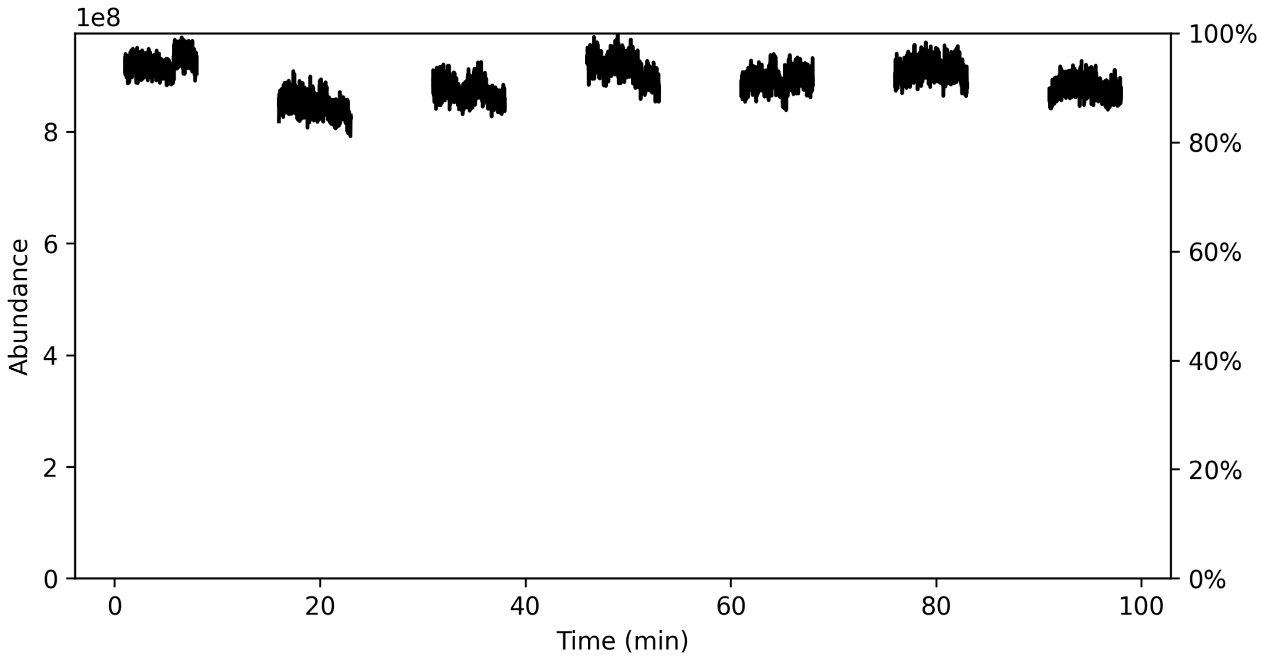

Block	TIC min	TIC max	TIC mean	RSD (%)
1	8.83e+08	9.69e+08	9.22e+08	1.69
2	7.92e+08	9.09e+08	8.48e+08	2.10
3	8.27e+08	9.25e+08	8.72e+08	1.86
4	8.54e+08	9.76e+08	9.14e+08	2.11
5	8.39e+08	9.39e+08	8.92e+08	1.72
6	8.64e+08	9.60e+08	9.13e+08	1.63
7	8.40e+08	9.27e+08	8.78e+08	1.68

2. Block Parameters

The Isotopic Ratio of the blocks were calculated by 'Mean'

2.1. 13C/M0

Block	Number of scans	Effective number of ions	Isotopic Ratio	STD	SEM	RSE
1	1280	2.09e+07	0.208953	0.001732	0.000048	0.000232
2	1254	2.07e+07	0.208703	0.001840	0.000052	0.000249
3	1296	2.15e+07	0.208799	0.001838	0.000051	0.000244
4	1302	2.16e+07	0.208575	0.001746	0.000048	0.000232
5	1281	2.16e+07	0.209351	0.001765	0.000049	0.000235
6	1296	2.21e+07	0.209344	0.001743	0.000048	0.000231
7	1278	2.20e+07	0.209454	0.001787	0.000050	0.000239

Errors and Test Paramters

Block	Acquisition Error (permil)	Shot-Noise (permil)	AE/SN ratio	Shapiro Wilk (p_value)	D'Agostino (p_value)
1	0.232	0.219	1.059	0.522	0.915
2	0.249	0.220	1.131	0.226	0.399
3	0.244	0.216	1.134	0.149	0.450
4	0.232	0.215	1.079	0.970	0.920
5	0.235	0.215	1.093	0.374	0.083
6	0.231	0.213	1.086	0.947	0.836
7	0.239	0.213	1.118	0.771	0.542

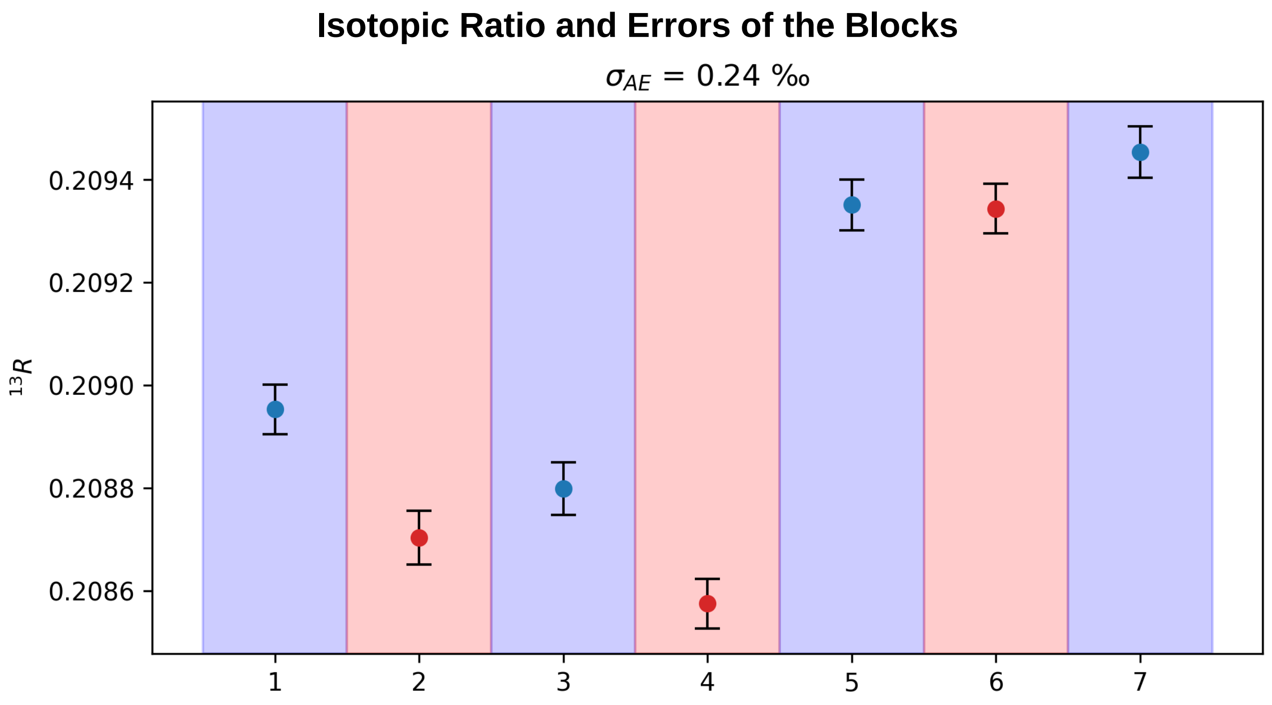

Cumulative Isotopic Ratio

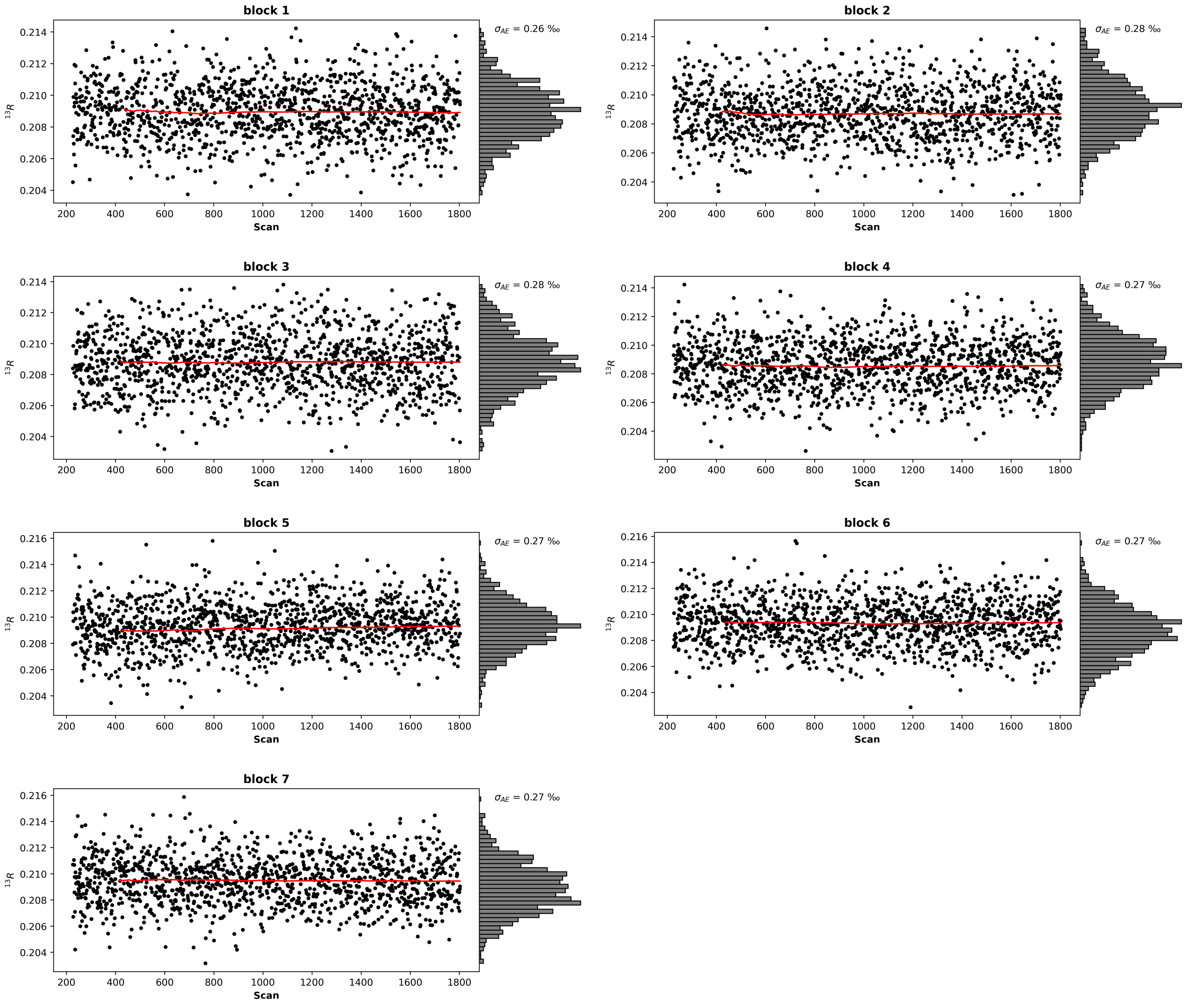

Acquisition Error and Shot-Noise

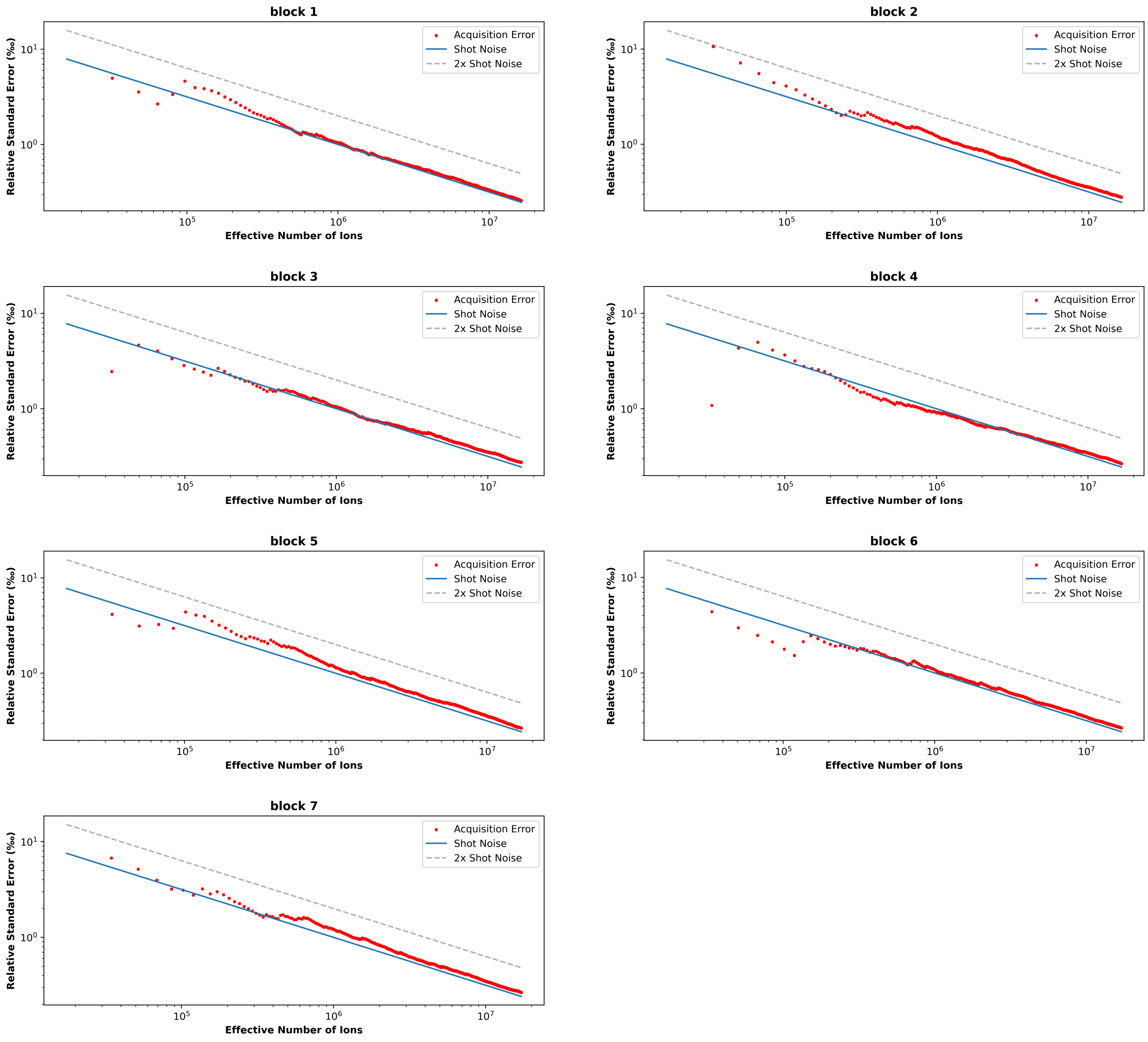

3. Delta Informations

Deltas were calculated by 'Average Of Neighboring Block Ratios'

3.1. 13C

Delta 13C was corrected by -27.80

Block	SEM	Delta corrected	Delta
2	0.25	-28.60	-0.83
4	0.23	-30.13	-2.39
6	0.23	-28.07	-0.28

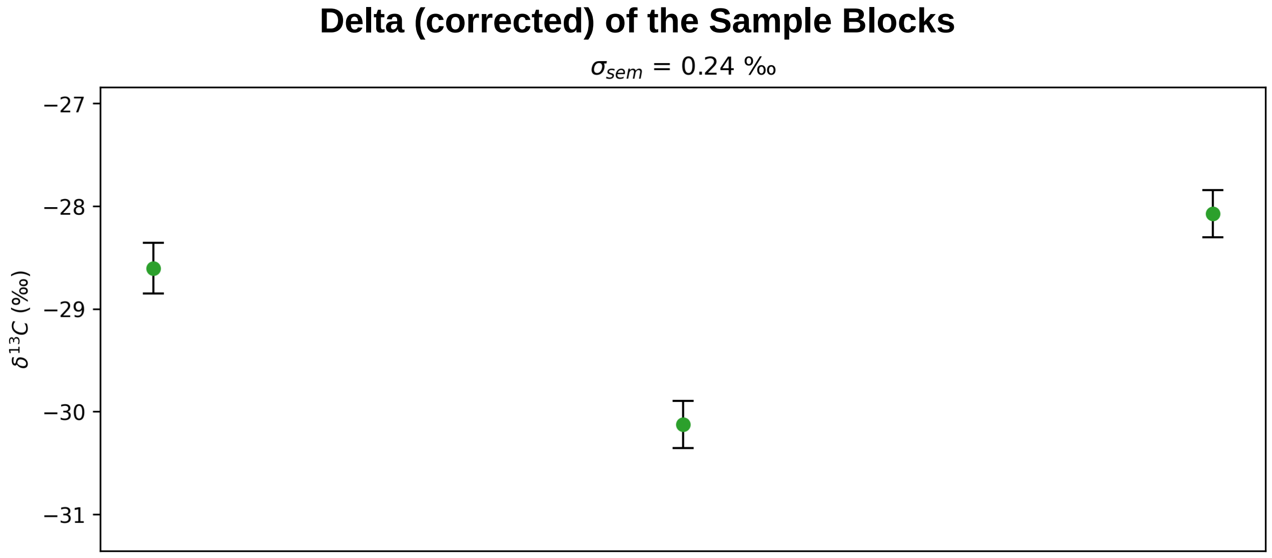

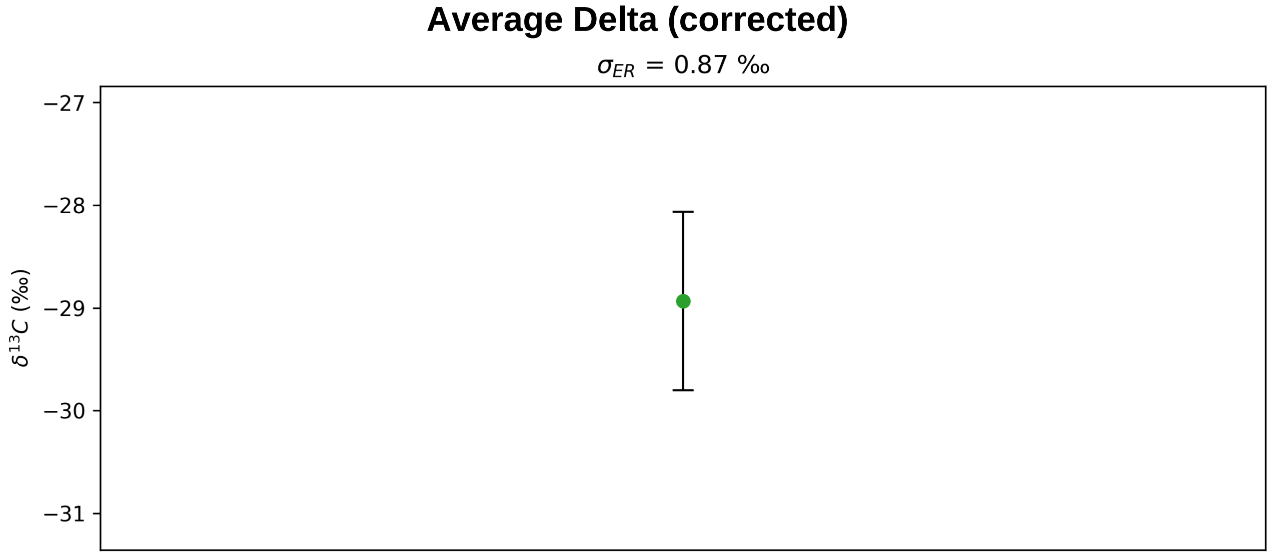

The final corrected average delta was -28.93 with a standard deviation of 0.87. Here the standard deviation is called reproducibility error.
