# Supplementary material for: Advancing Stable Isotope Analysis with Orbitrap-MS for Fatty Acid Methyl Esters and Complex Lipid Matrices
Source: J Am Soc Mass Spectrom. 2025 Jun 17;36(7):1527–35. doi: 10.1021/jasms.5c00092 (PMC12339014; doi:10.1021/jasms.5c00092)
Supplement: Supplementary file 2 [file js5c00092_si_002.zip › reports by IsotoPy Software/standards/Na+Standard8_DI.pdf]

Standard 8 - [M + Na]+
Isotope Analysis report from IsotoPy
Dual Inlet

1. Pre Processing

1.1. Block Time and Scan Information

Information about sample and standard block times and scans:

Block	Injected	Initial Time	End Time	Number of scans
1	standard	1	5	758
2	sample	6	10	714
3	standard	11	15	715
4	sample	16	20	741
5	standard	21	25	724
6	sample	26	30	749
7	standard	31	35	735

1.2. Outlier Removal

A total of 1168 scans were considered outliers and removed using the MAD method

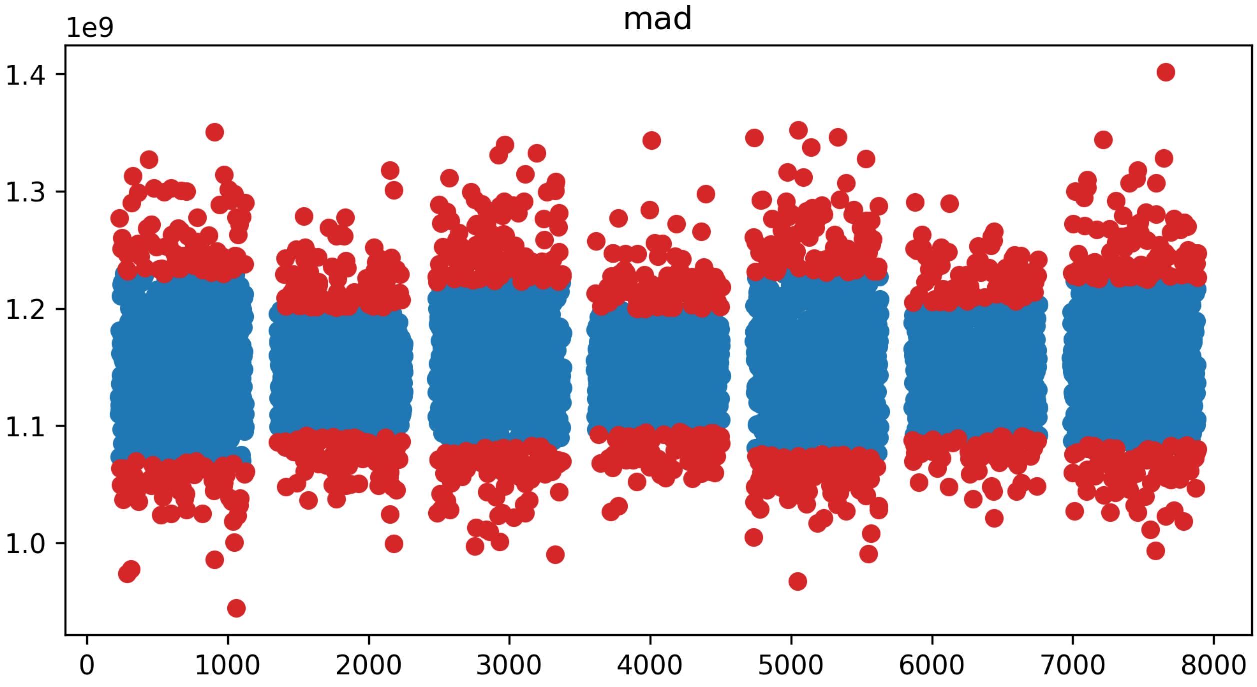

1.3. Total Ion Current (TIC)

TIC of all blocks

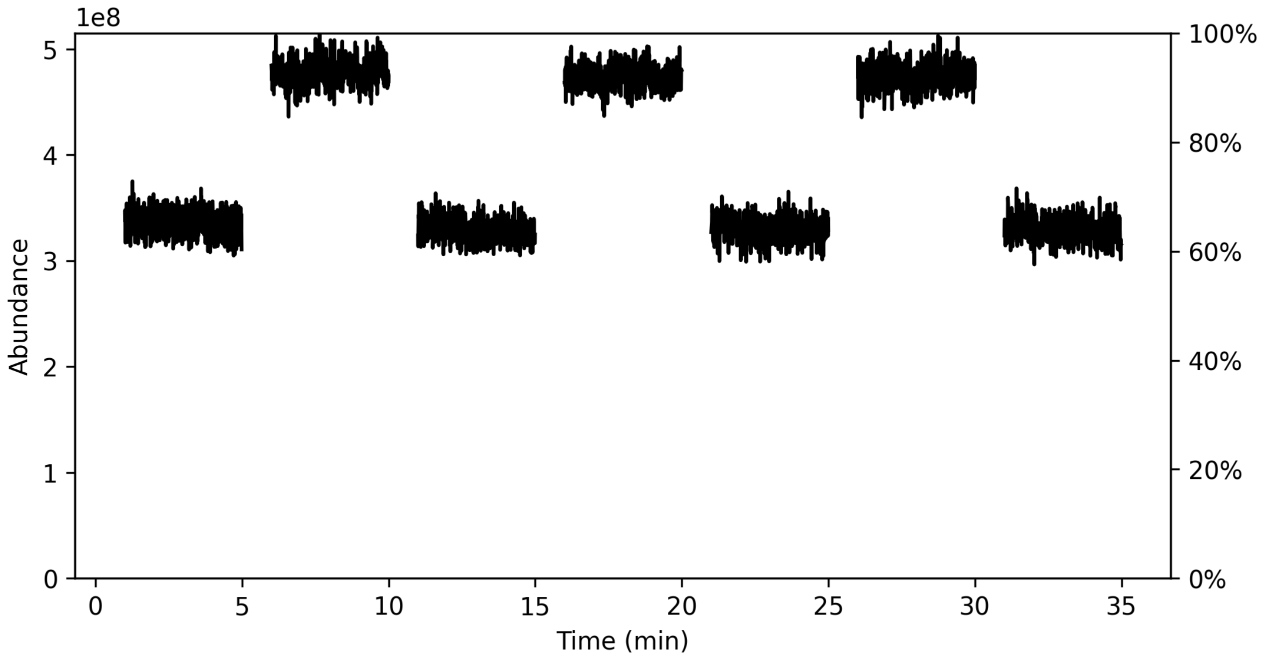

Block	TIC min	TIC max	TIC mean	RSD (%)
1	3.05e+08	3.75e+08	3.35e+08	3.46
2	4.36e+08	5.15e+08	4.79e+08	2.40
3	3.05e+08	3.64e+08	3.30e+08	3.01
4	4.37e+08	5.03e+08	4.74e+08	2.22
5	2.99e+08	3.65e+08	3.30e+08	3.30
6	4.36e+08	5.13e+08	4.74e+08	2.43
7	2.96e+08	3.69e+08	3.30e+08	3.24

2. Block Parameters

The Isotopic Ratio of the blocks were calculated by 'Mean'

2.1. 13C/M0

Block	Number of scans	Effective number of ions	Isotopic Ratio	STD	SEM	RSE
1	758	1.61e+07	0.210141	0.001404	0.000051	0.000243
2	714	1.54e+07	0.210074	0.001318	0.000049	0.000235
3	715	1.54e+07	0.209800	0.001375	0.000051	0.000245
4	741	1.60e+07	0.209955	0.001359	0.000050	0.000238
5	724	1.57e+07	0.209566	0.001375	0.000051	0.000244
6	749	1.63e+07	0.209617	0.001348	0.000049	0.000235
7	735	1.60e+07	0.209398	0.001417	0.000052	0.000249

Errors and Test Paramters

Block	Acquisition Error (permil)	Shot-Noise (permil)	AE/SN ratio	Shapiro Wilk (p_value)	D'Agostino (p_value)
1	0.243	0.249	0.974	0.950	0.904
2	0.235	0.255	0.921	0.987	0.972
3	0.245	0.254	0.963	0.716	0.752
4	0.238	0.250	0.952	0.841	0.672
5	0.244	0.253	0.964	0.506	0.577
6	0.235	0.248	0.948	0.207	0.759
7	0.249	0.250	0.997	0.448	0.569

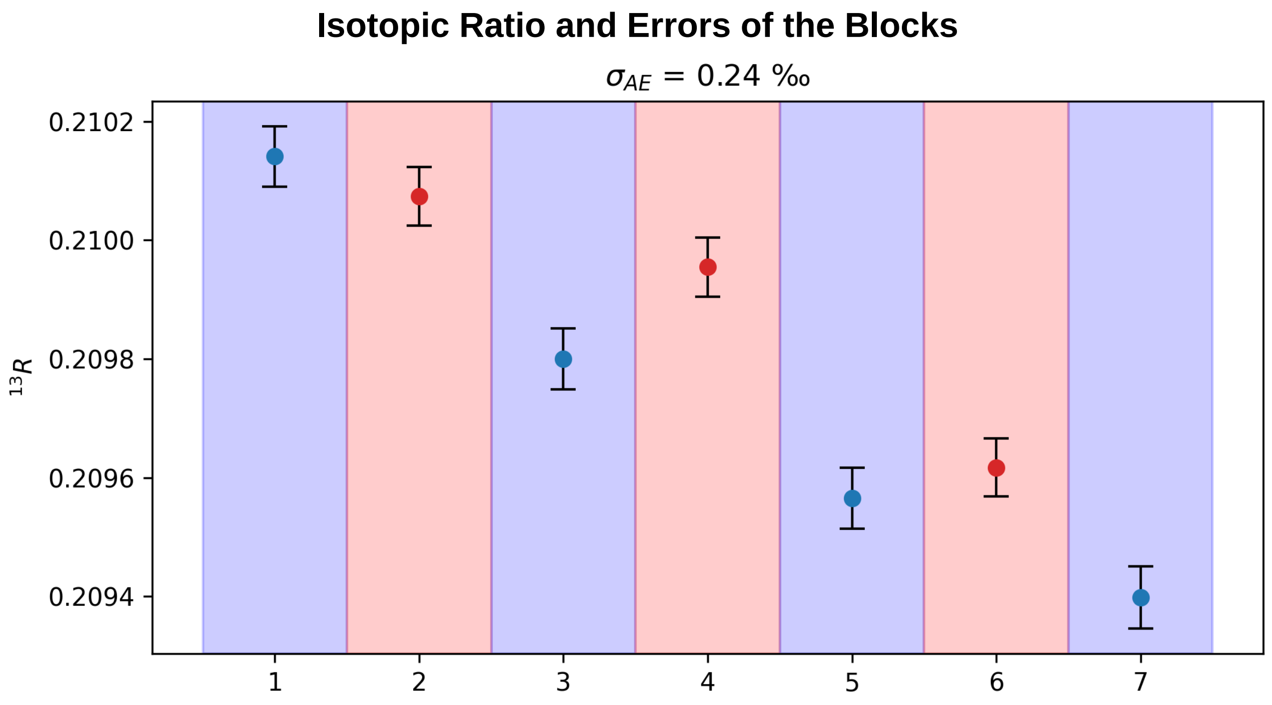
Isotopic Ratio and Errors of the Blocks
σ_{AE} = 0.24 ‰
^{13}R
0.2102
0.2100
0.2098
0.2096
0.2094
1
2
3
4
5
6
7

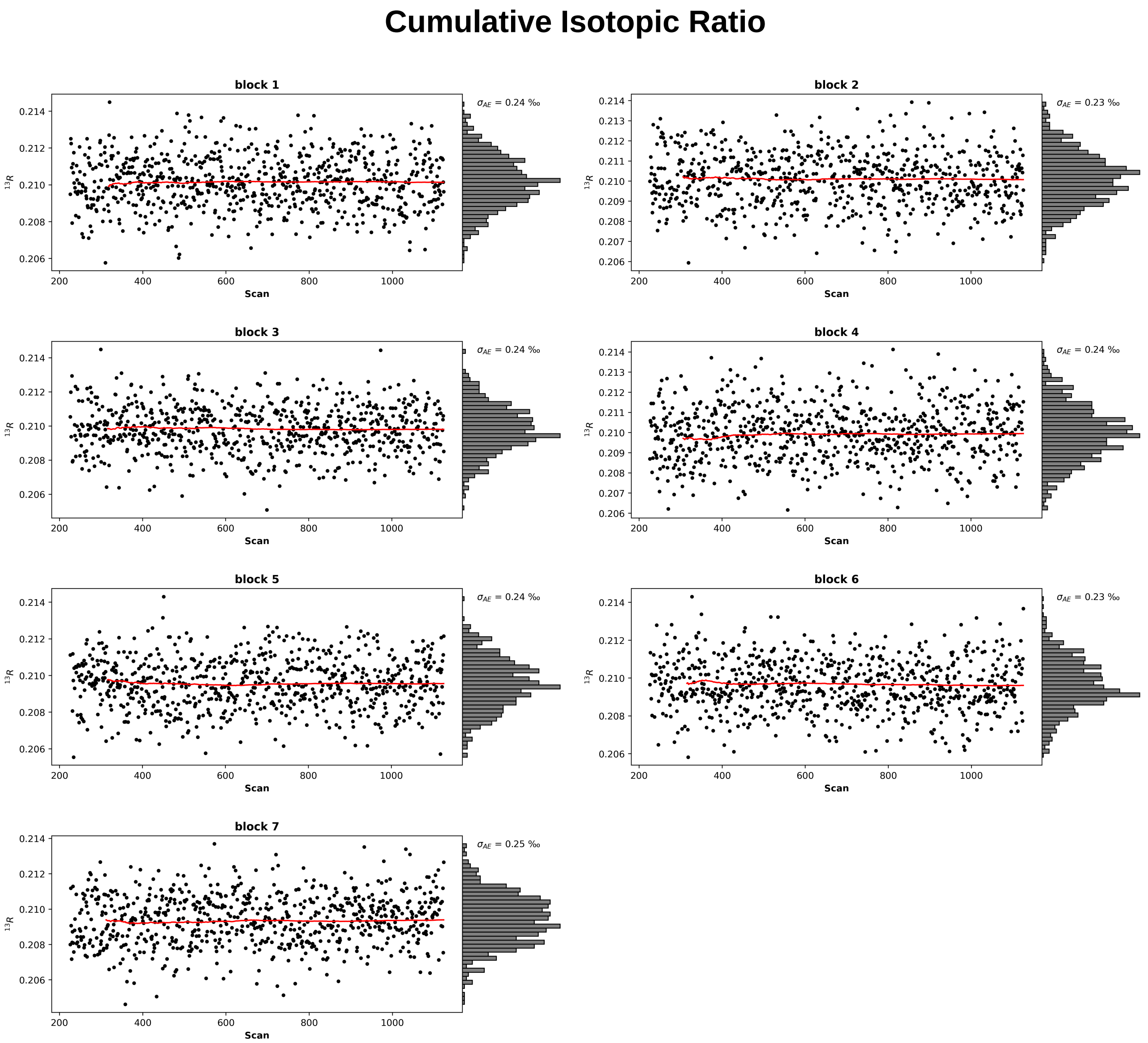
Cumulative Isotopic Ratio
block 1
σ_{AE} = 0.24 ‰
block 2
σ_{AE} = 0.23 ‰
block 3
σ_{AE} = 0.24 ‰
block 4
σ_{AE} = 0.24 ‰
block 5
σ_{AE} = 0.24 ‰
block 6
σ_{AE} = 0.23 ‰
block 7
σ_{AE} = 0.25 ‰
^{13}R
Scan

Acquisition Error and Shot-Noise

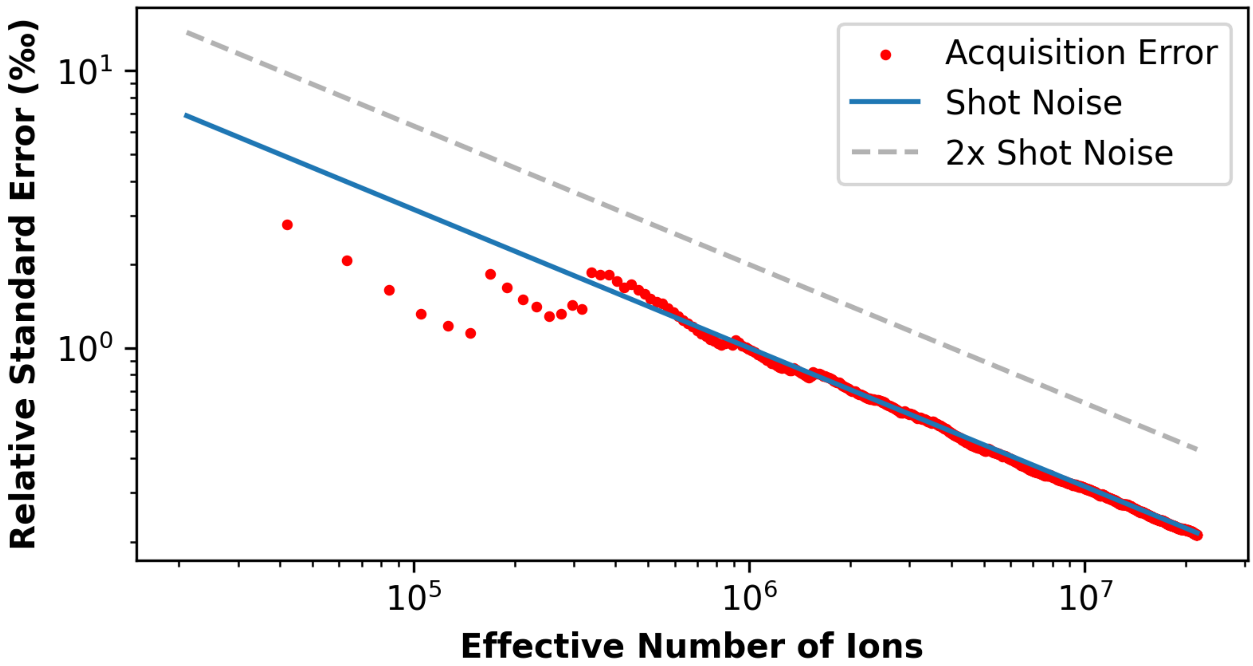

3. Delta Informations

Deltas were calculated by 'Average Of Neighboring Block Ratios'

3.1. 13C

Delta 13C was corrected by -27.80

Block	SEM	Delta corrected	Delta
2	0.23	-27.32	0.49
4	0.24	-26.54	1.30
6	0.23	-27.17	0.65

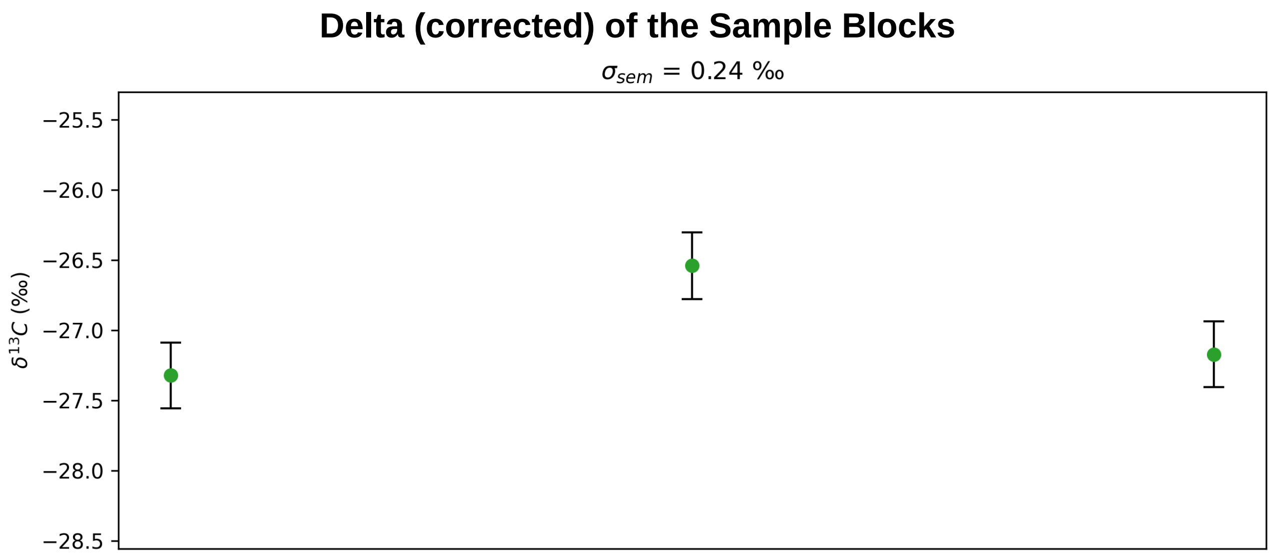

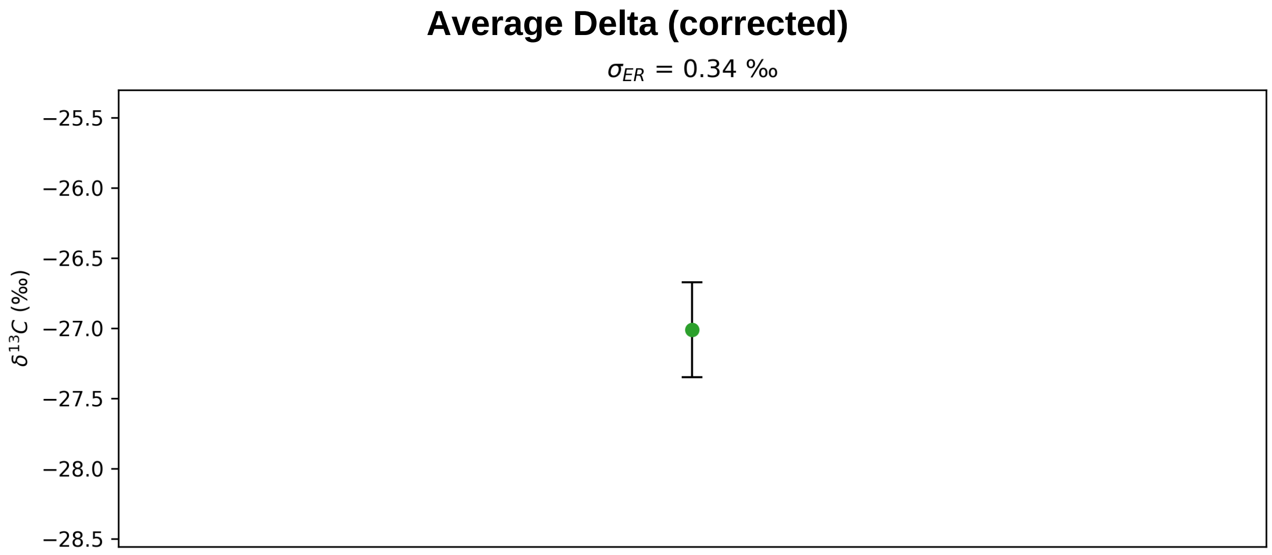

The final corrected average delta was -27.01 with a standard deviation of 0.34. Here the standard deviation is called reproducibility error.
