# Supplementary material for: Advancing Stable Isotope Analysis with Orbitrap-MS for Fatty Acid Methyl Esters and Complex Lipid Matrices
Source: J Am Soc Mass Spectrom. 2025 Jun 17;36(7):1527–35. doi: 10.1021/jasms.5c00092 (PMC12339014; doi:10.1021/jasms.5c00092)
Supplement: Supplementary file 2 [file js5c00092_si_002.zip › reports by IsotoPy Software/standards/Na+Standard4_DI.pdf]

Standard 4 - [M + Na]+
Isotope Analysis report from IsotoPy

Dual Inlet

1. Pre Processing

1.1. Block Time and Scan Information

Information about sample and standard block times and scans:

Block	Injected	Initial Time	End Time	Number of scans
1	standard	1	5	747
2	sample	6	10	744
3	standard	11	15	718
4	sample	16	20	733
5	standard	21	25	719
6	sample	26	30	749
7	standard	31	35	721

1.2. Outlier Removal

A total of 1173 scans were considered outliers and removed using the MAD method

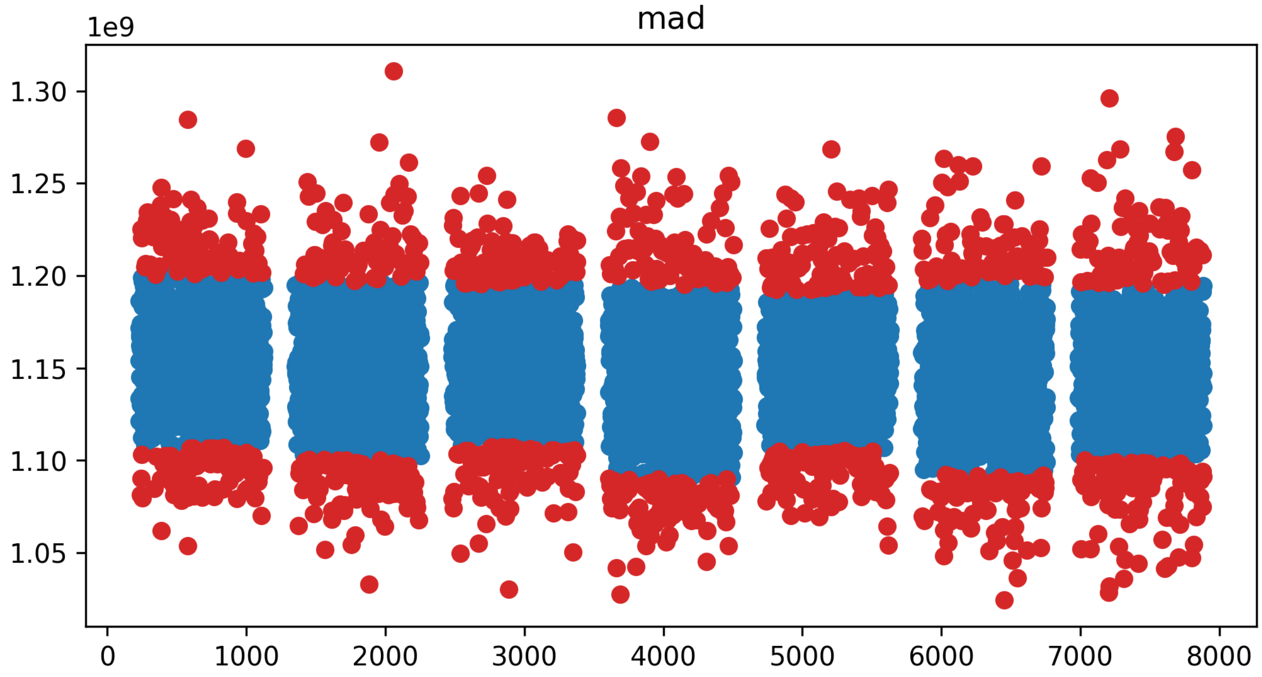

1.3. Total Ion Current (TIC)

TIC of all blocks

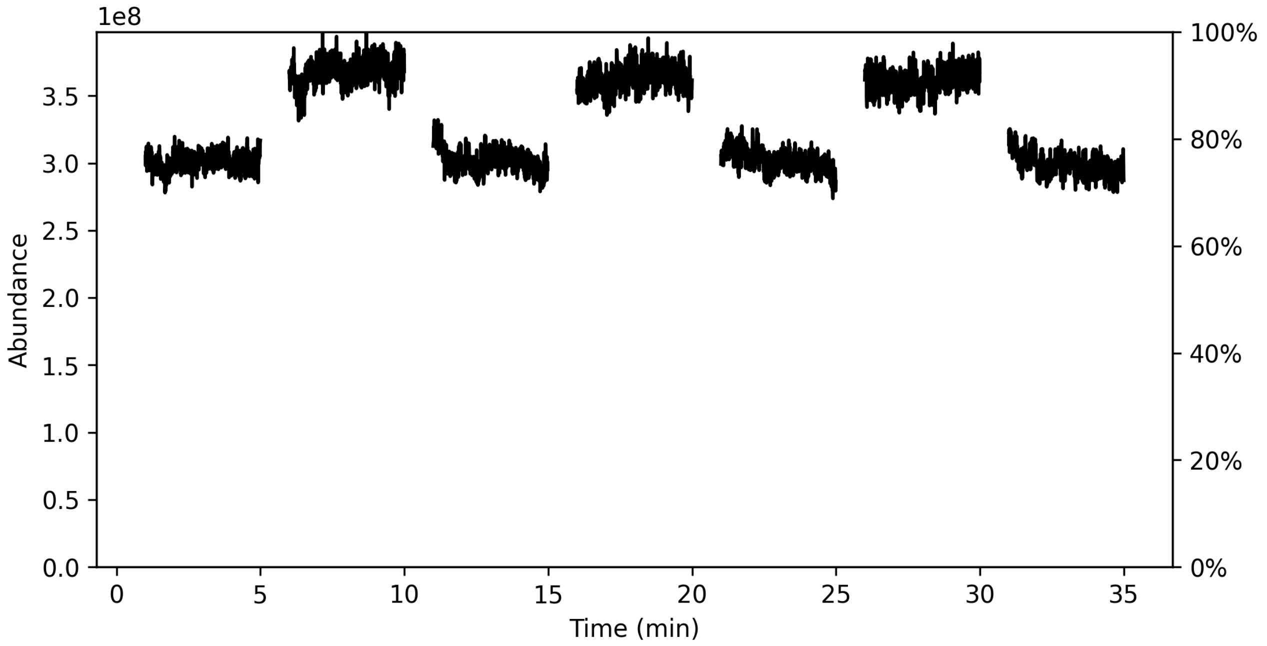

Block	TIC min	TIC max	TIC mean	RSD (%)
1	2.78e+08	3.20e+08	3.01e+08	2.15
2	3.32e+08	3.97e+08	3.68e+08	2.59
3	2.79e+08	3.32e+08	3.02e+08	2.85
4	3.36e+08	3.93e+08	3.64e+08	2.47
5	2.74e+08	3.28e+08	3.02e+08	2.74
6	3.37e+08	3.89e+08	3.62e+08	2.35
7	2.78e+08	3.25e+08	2.99e+08	2.95

2. Block Parameters

The Isotopic Ratio of the blocks were calculated by 'Mean'

2.1. 13C/M0

Block	Number of scans	Effective number of ions	Isotopic Ratio	STD	SEM	RSE
1	747	1.62e+07	0.210178	0.001372	0.000050	0.000239
2	744	1.60e+07	0.210133	0.001382	0.000051	0.000241
3	718	1.54e+07	0.210495	0.001361	0.000051	0.000241
4	733	1.56e+07	0.210564	0.001385	0.000051	0.000243
5	719	1.53e+07	0.210690	0.001370	0.000051	0.000242
6	749	1.60e+07	0.210572	0.001347	0.000049	0.000234
7	721	1.52e+07	0.210590	0.001373	0.000051	0.000243

Errors and Test Paramters

Block	Acquisition Error (permil)	Shot-Noise (permil)	AE/SN ratio	Shapiro Wilk (p_value)	D'Agostino (p_value)
1	0.239	0.248	0.961	0.277	0.465
2	0.241	0.250	0.965	0.345	0.159
3	0.241	0.255	0.947	0.006	0.020
4	0.243	0.253	0.960	0.347	0.479
5	0.242	0.255	0.949	0.698	0.577
6	0.234	0.250	0.933	0.201	0.489
7	0.243	0.257	0.945	0.983	0.986

Isotopic Ratio and Errors of the Blocks

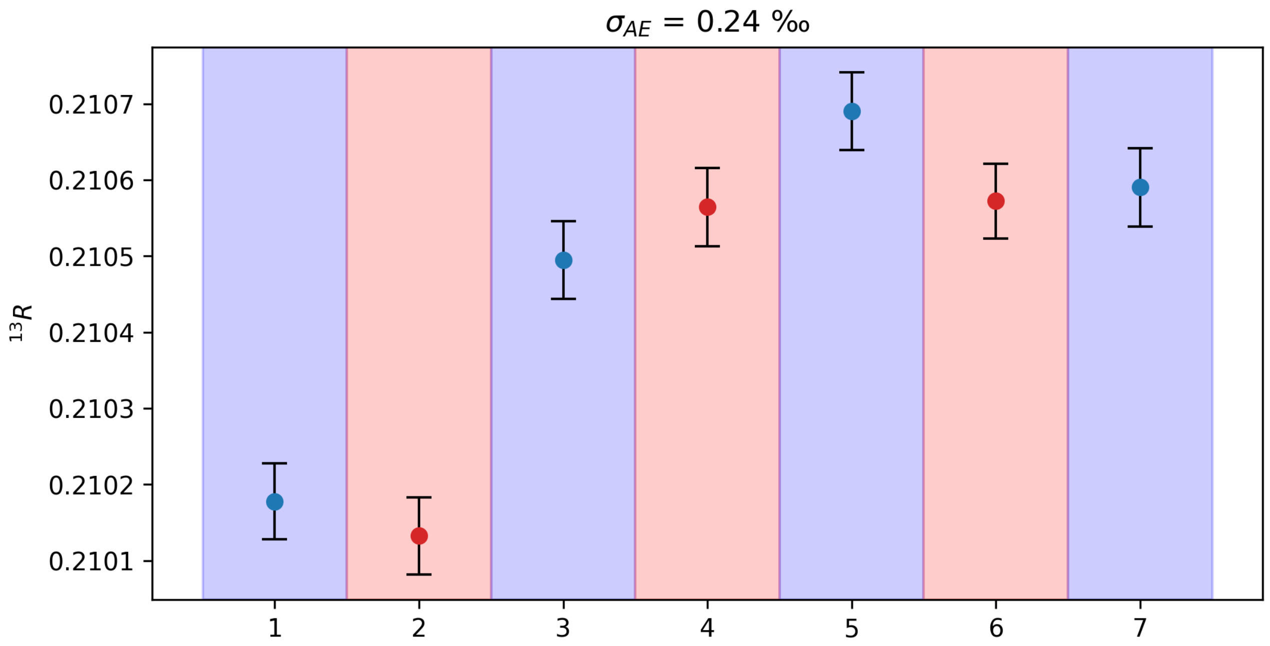

Cumulative Isotopic Ratio

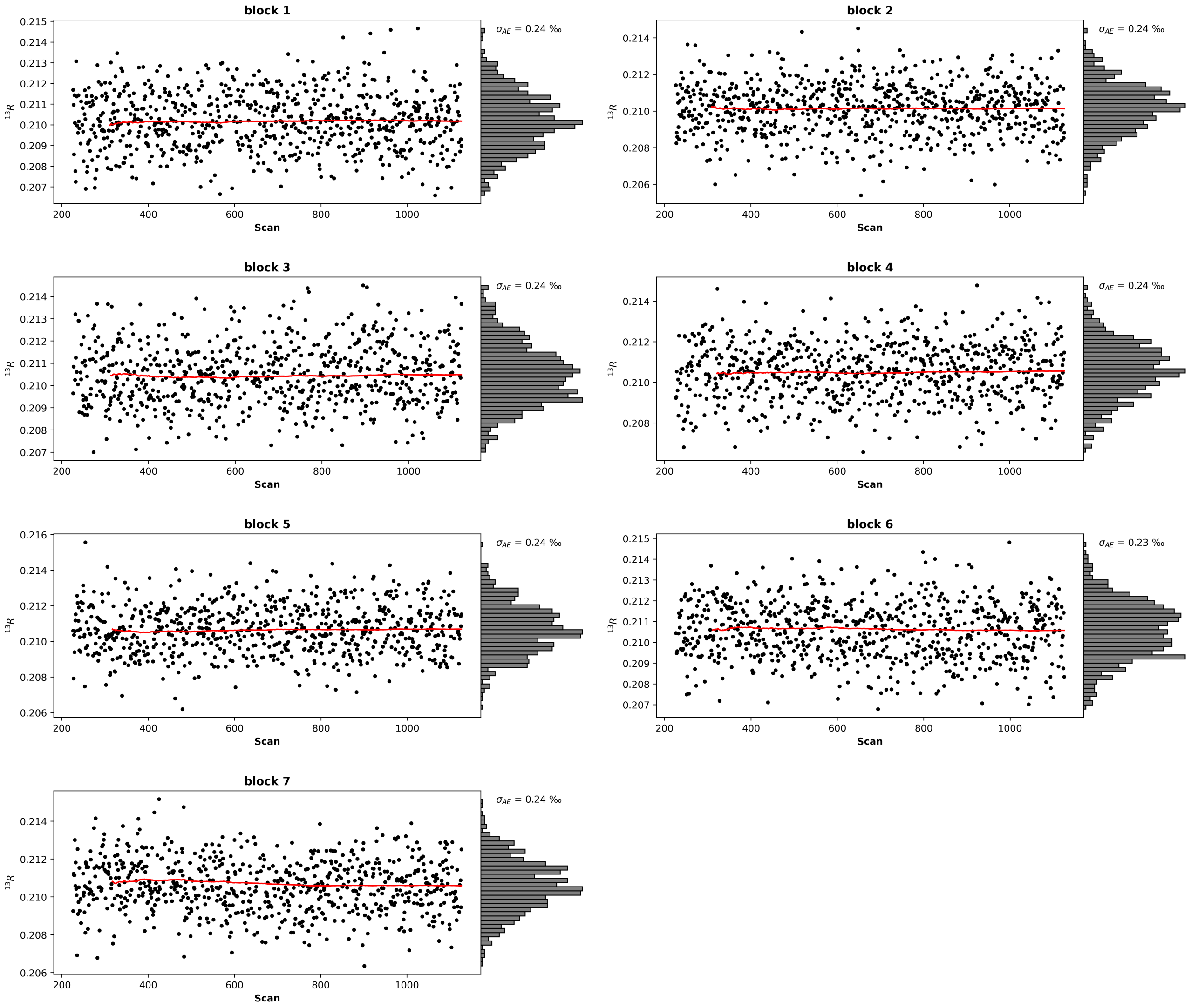

Acquisition Error and Shot-Noise

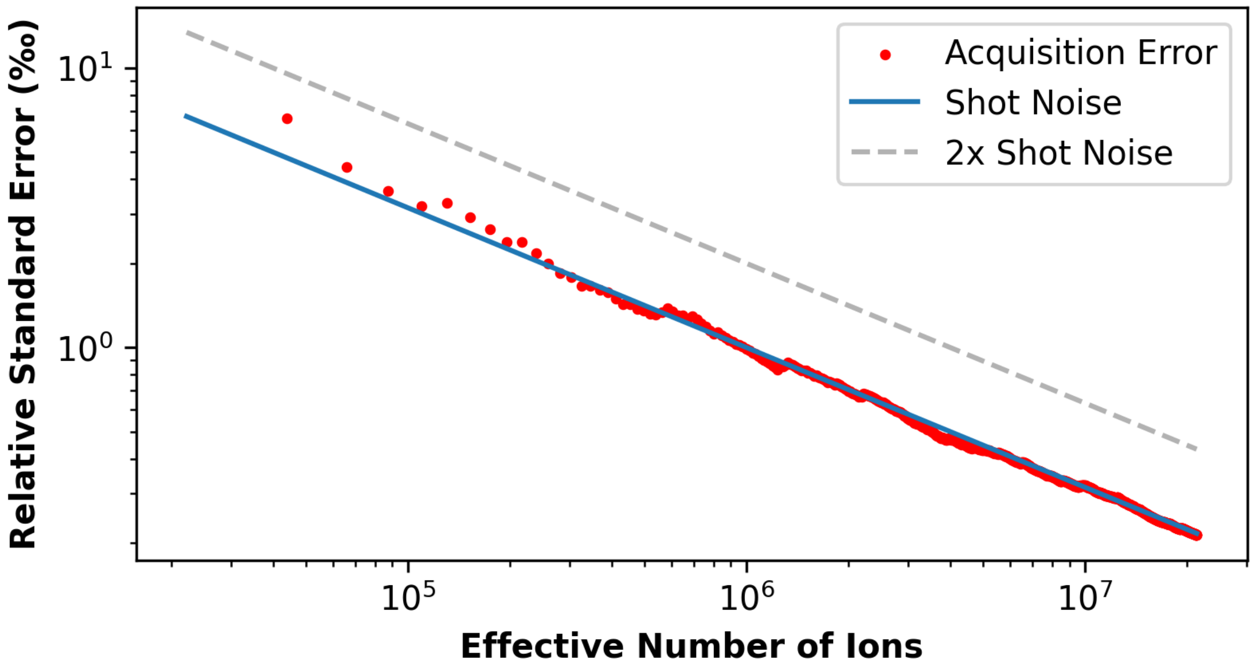

3. Delta Informations

Deltas were calculated by 'Average Of Neighboring Block Ratios'

3.1. 13C

Delta 13C was corrected by -27.80

Block	SEM	Delta corrected	Delta
2	0.24	-28.74	-0.97
4	0.24	-27.93	-0.13
6	0.23	-28.11	-0.32

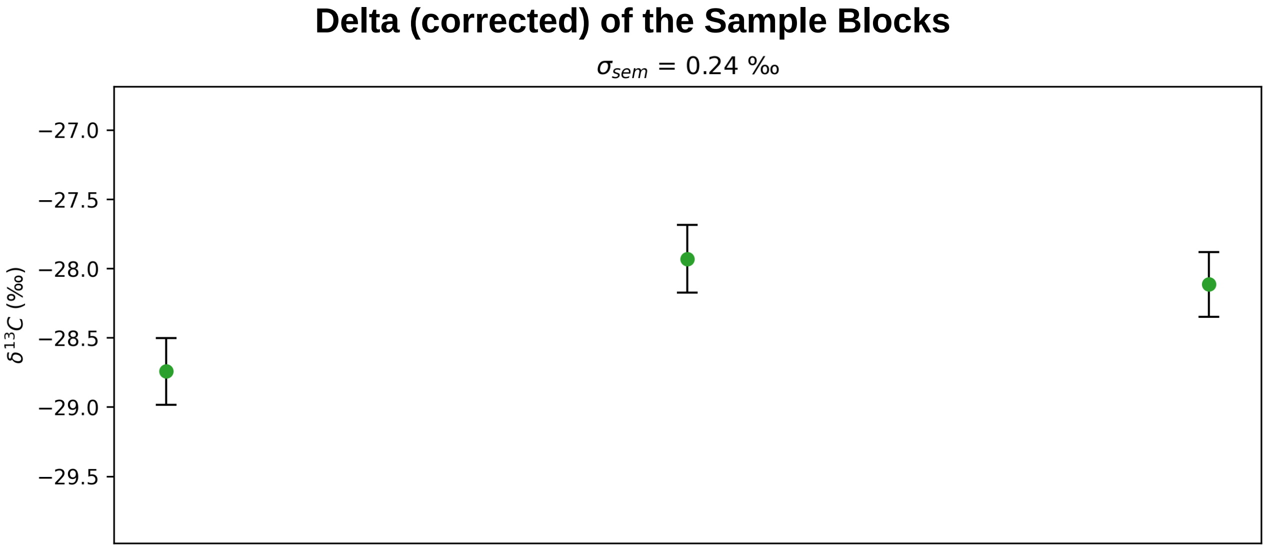

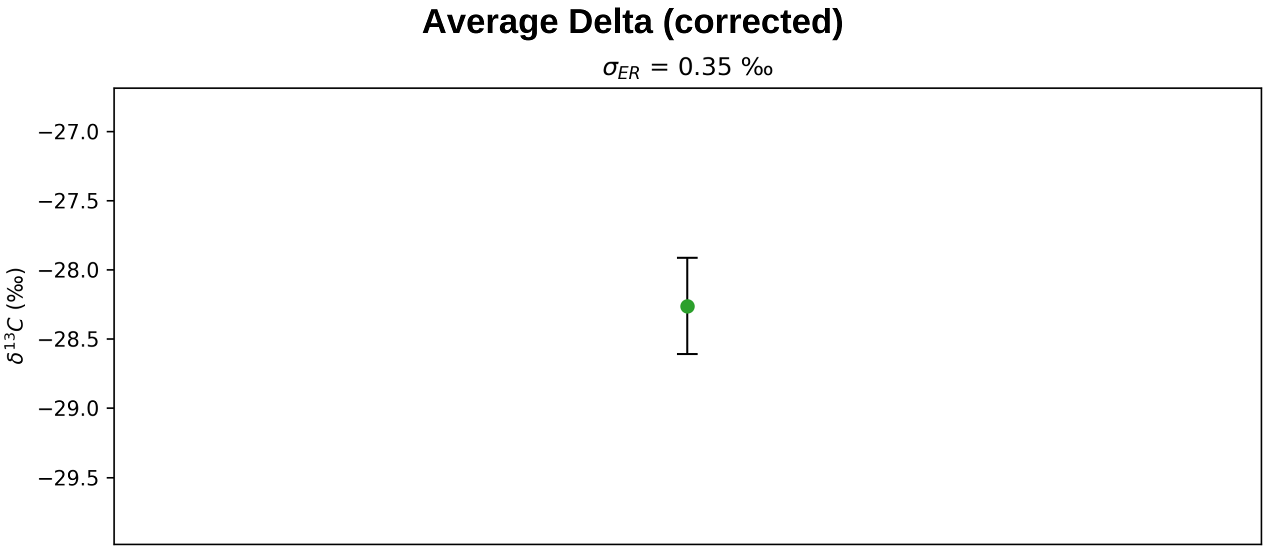

The final corrected average delta was -28.26 with a standard deviation of 0.35. Here the standard deviation is called reproducibility error.
