# Supplementary material for: Advancing Stable Isotope Analysis with Orbitrap-MS for Fatty Acid Methyl Esters and Complex Lipid Matrices
Source: J Am Soc Mass Spectrom. 2025 Jun 17;36(7):1527–35. doi: 10.1021/jasms.5c00092 (PMC12339014; doi:10.1021/jasms.5c00092)
Supplement: Supplementary file 2 [file js5c00092_si_002.zip › reports by IsotoPy Software/standards/H+Standard6_FI.pdf]

Standard 6 - [M + H]+
Isotope Analysis report from IsotoPy

Flow Injection

1. Pre Processing

1.1. Block Time and Scan Information

Information about sample and standard block times and scans:

Block	Injected	Initial Time	End Time	Number of scans
1	standard	1	8	1311
2	sample	16	23	1304
3	standard	31	38	1295
4	sample	46	53	1294
5	standard	61	68	1308
6	sample	76	83	1274
7	standard	91	98	1271

1.2. Outlier Removal

A total of 1982 scans were considered outliers and removed using the MAD method

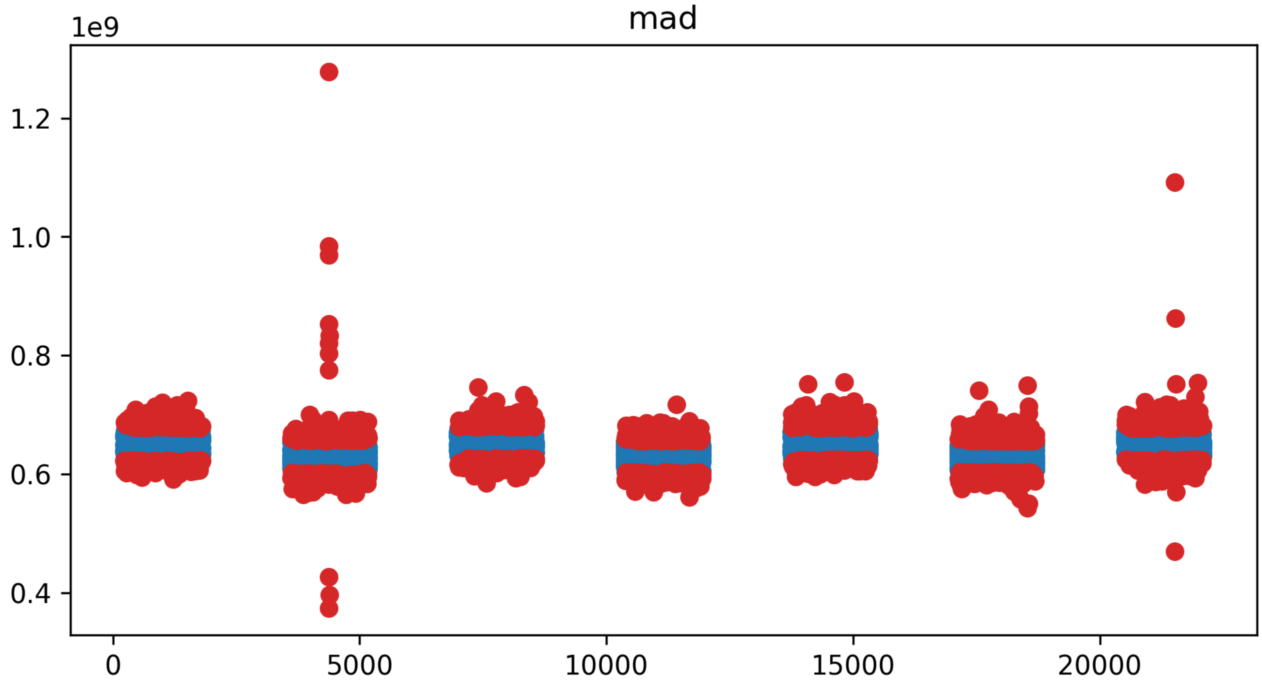

1.3. Total Ion Current (TIC)

TIC of all blocks

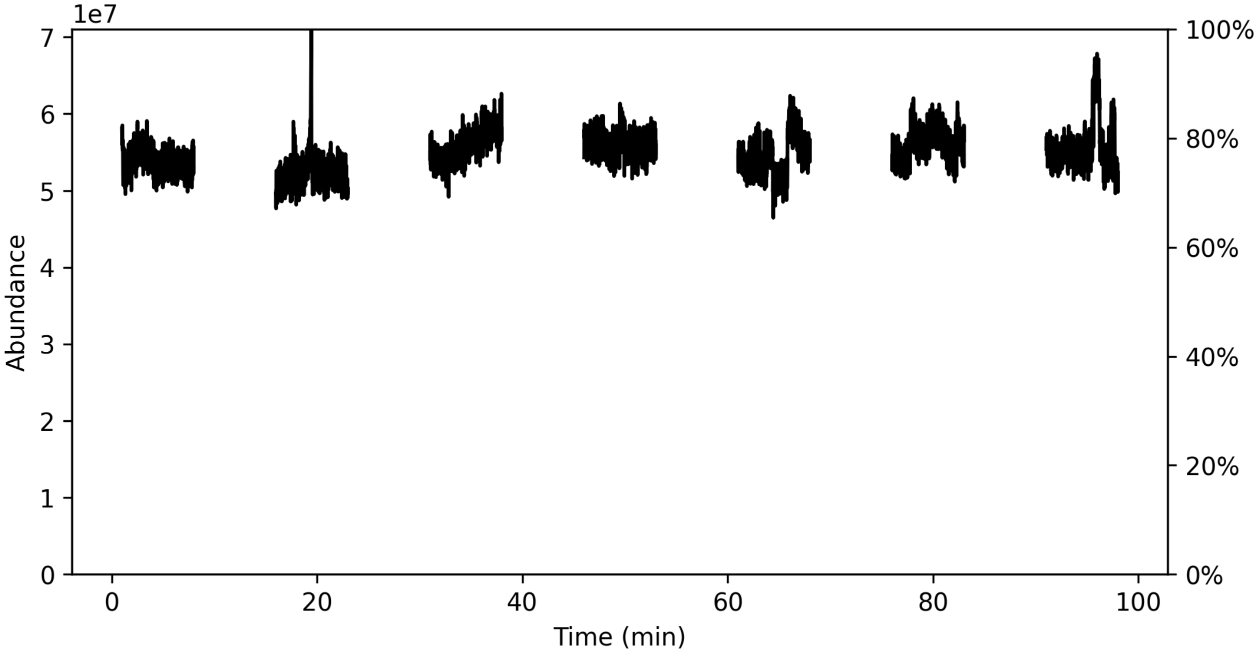

Block	TIC min	TIC max	TIC mean	RSD (%)
1	4.95e+07	5.91e+07	5.39e+07	2.82
2	4.77e+07	7.10e+07	5.22e+07	3.33
3	4.92e+07	6.26e+07	5.59e+07	3.61
4	5.15e+07	6.13e+07	5.60e+07	2.58
5	4.65e+07	6.23e+07	5.45e+07	4.88
6	5.12e+07	6.20e+07	5.61e+07	3.48
7	4.96e+07	6.78e+07	5.54e+07	5.55

2. Block Parameters

The Isotopic Ratio of the blocks were calculated by 'Mean'

2.1. 13C/M0

Block	Number of scans	Effective number of ions	Isotopic Ratio	STD	SEM	RSE
1	1311	1.76e+07	0.209971	0.001749	0.000048	0.000230
2	1304	1.74e+07	0.209717	0.001740	0.000048	0.000230
3	1295	1.74e+07	0.209956	0.001774	0.000049	0.000235
4	1294	1.73e+07	0.209661	0.001772	0.000049	0.000235
5	1308	1.76e+07	0.210072	0.001716	0.000047	0.000226
6	1274	1.71e+07	0.209759	0.001765	0.000049	0.000236
7	1271	1.71e+07	0.210091	0.001720	0.000048	0.000230

Errors and Test Paramters

Block	Acquisition Error (permil)	Shot-Noise (permil)	AE/SN ratio	Shapiro Wilk (p_value)	D'Agostino (p_value)
1	0.230	0.238	0.965	0.099	0.072
2	0.230	0.239	0.959	0.577	0.569
3	0.235	0.239	0.980	0.135	0.154
4	0.235	0.240	0.978	0.358	0.850
5	0.226	0.238	0.948	0.875	0.848
6	0.236	0.242	0.974	0.435	0.595
7	0.230	0.242	0.951	0.163	0.099

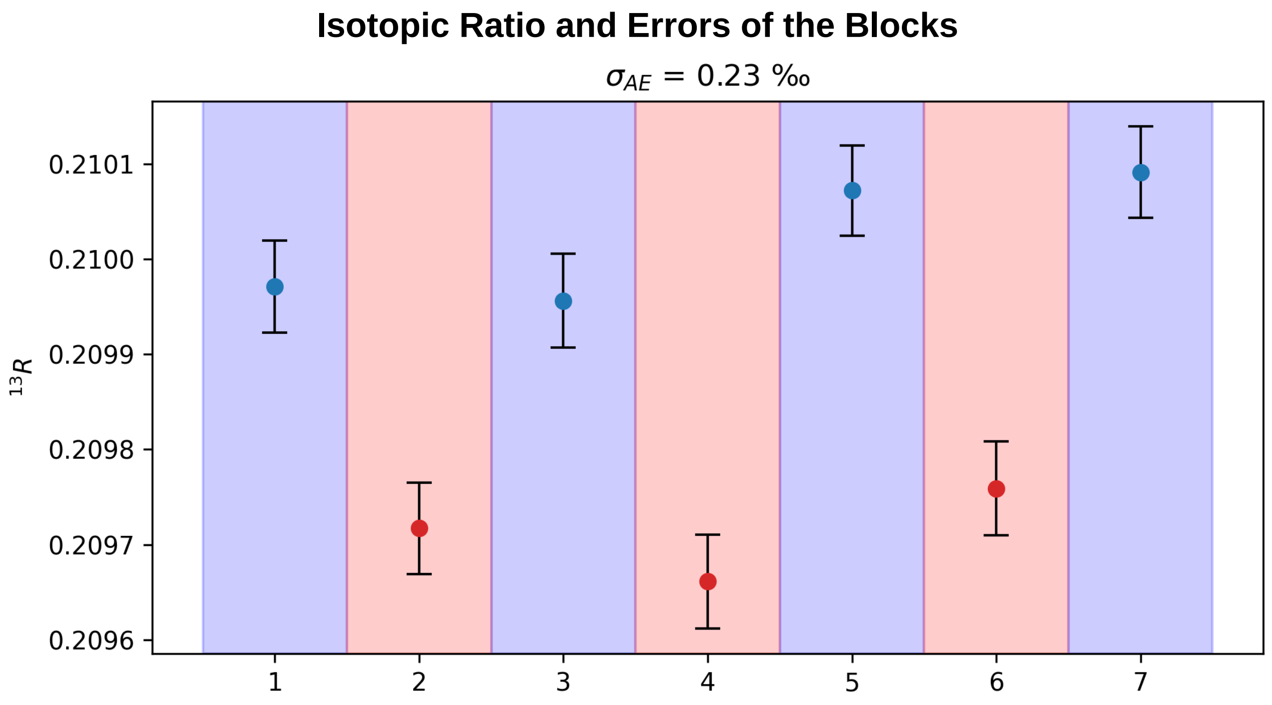
Isotopic Ratio and Errors of the Blocks
σ_{AE} = 0.23 ‰
^{13}R
0.2101
0.2100
0.2099
0.2098
0.2097
0.2096
1
2
3
4
5
6
7

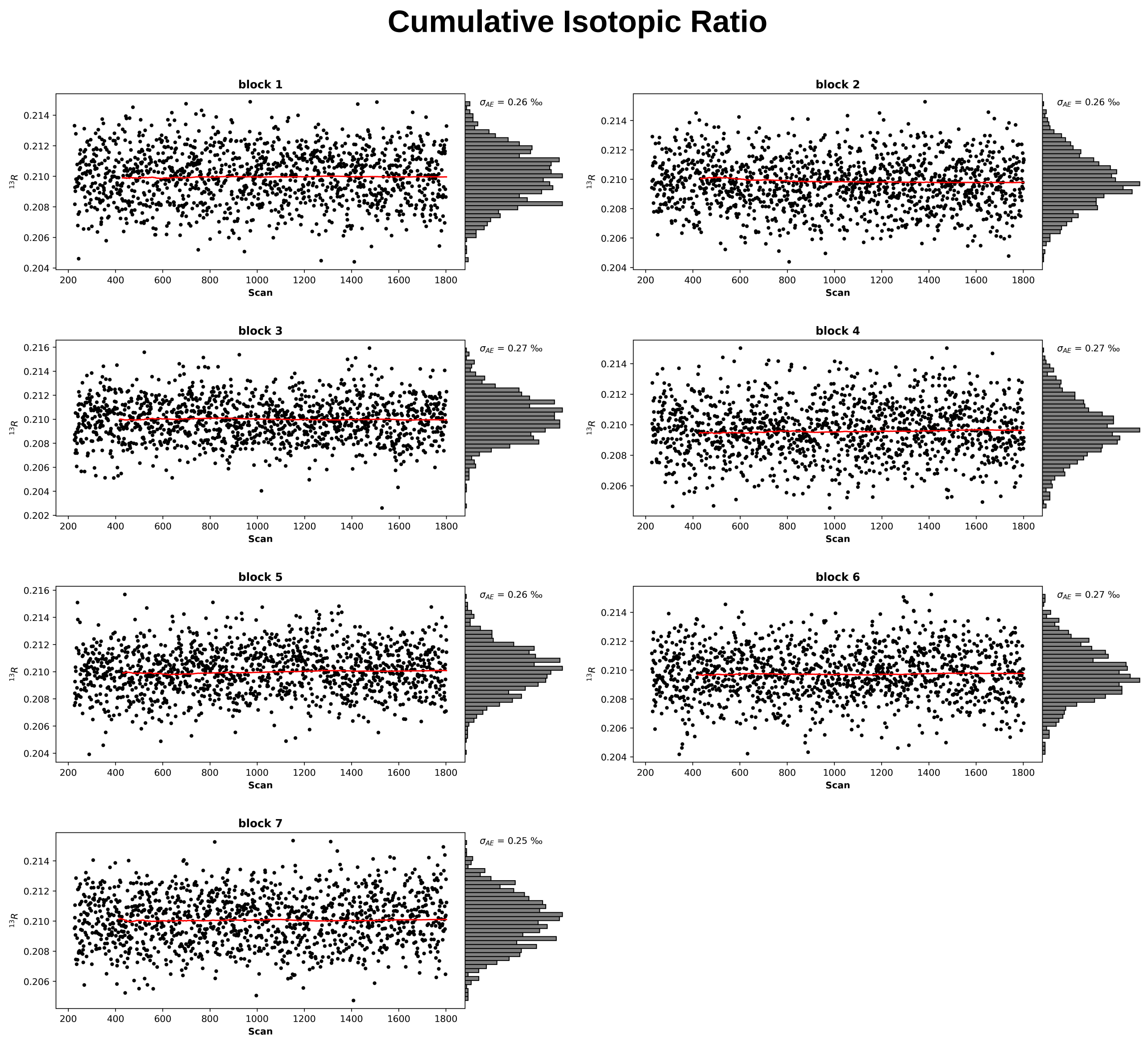
Cumulative Isotopic Ratio
block 1
σ_{AE} = 0.26 ‰
block 2
σ_{AE} = 0.26 ‰
block 3
σ_{AE} = 0.27 ‰
block 4
σ_{AE} = 0.27 ‰
block 5
σ_{AE} = 0.26 ‰
block 6
σ_{AE} = 0.27 ‰
block 7
σ_{AE} = 0.25 ‰
^{13}R
Scan

Acquisition Error and Shot-Noise

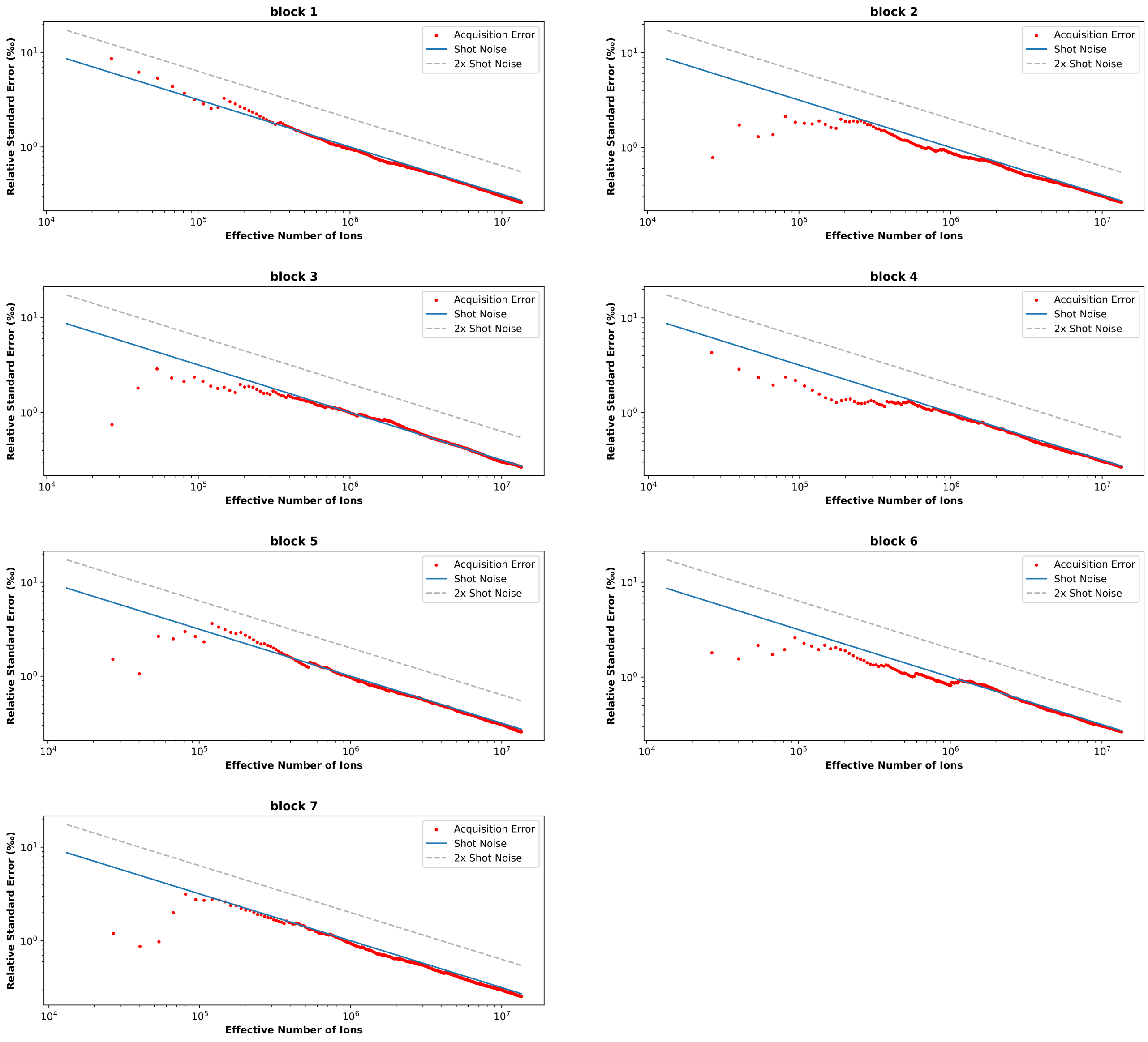

3. Delta Informations

Deltas were calculated by 'Average Of Neighboring Block Ratios'

3.1. 13C

Delta 13C was corrected by -27.80

Block	SEM	Delta corrected	Delta
2	0.23	-28.94	-1.17
4	0.23	-29.43	-1.68
6	0.24	-29.29	-1.54

Delta (corrected) of the Sample Blocks

σ_{sem} = 0.23 ‰

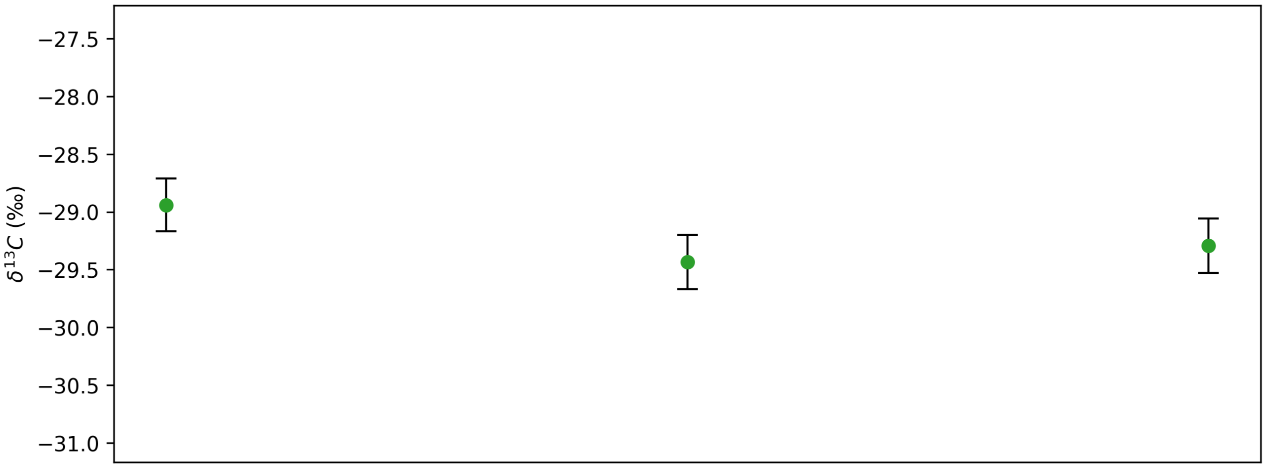

Average Delta (corrected)

σ_{ER} = 0.21 ‰

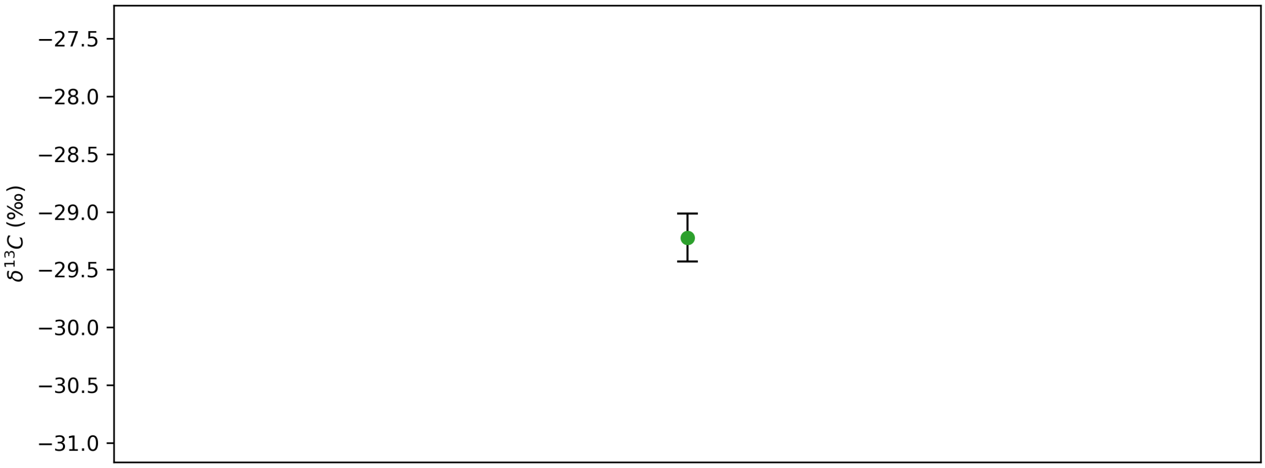

The final corrected average delta was -29.22 with a standard deviation of 0.21. Here the standard deviation is called reproducibility error.
