# Supplementary material for: Advancing Stable Isotope Analysis with Orbitrap-MS for Fatty Acid Methyl Esters and Complex Lipid Matrices
Source: J Am Soc Mass Spectrom. 2025 Jun 17;36(7):1527–35. doi: 10.1021/jasms.5c00092 (PMC12339014; doi:10.1021/jasms.5c00092)
Supplement: Supplementary file 2 [file js5c00092_si_002.zip › reports by IsotoPy Software/standards/H+Standard1_DI.pdf]

Standard 1 - [M + H]+
Isotope Analysis report from IsotoPy

Dual Inlet

1. Pre Processing

1.1. Block Time and Scan Information

Information about sample and standard block times and scans:

Block	Injected	Initial Time	End Time	Number of scans
1	standard	1	5	744
2	sample	6	10	732
3	standard	11	15	742
4	sample	16	20	712
5	standard	21	25	729
6	sample	26	30	731
7	standard	31	35	726

1.2. Outlier Removal

A total of 1192 scans were considered outliers and removed using the MAD method

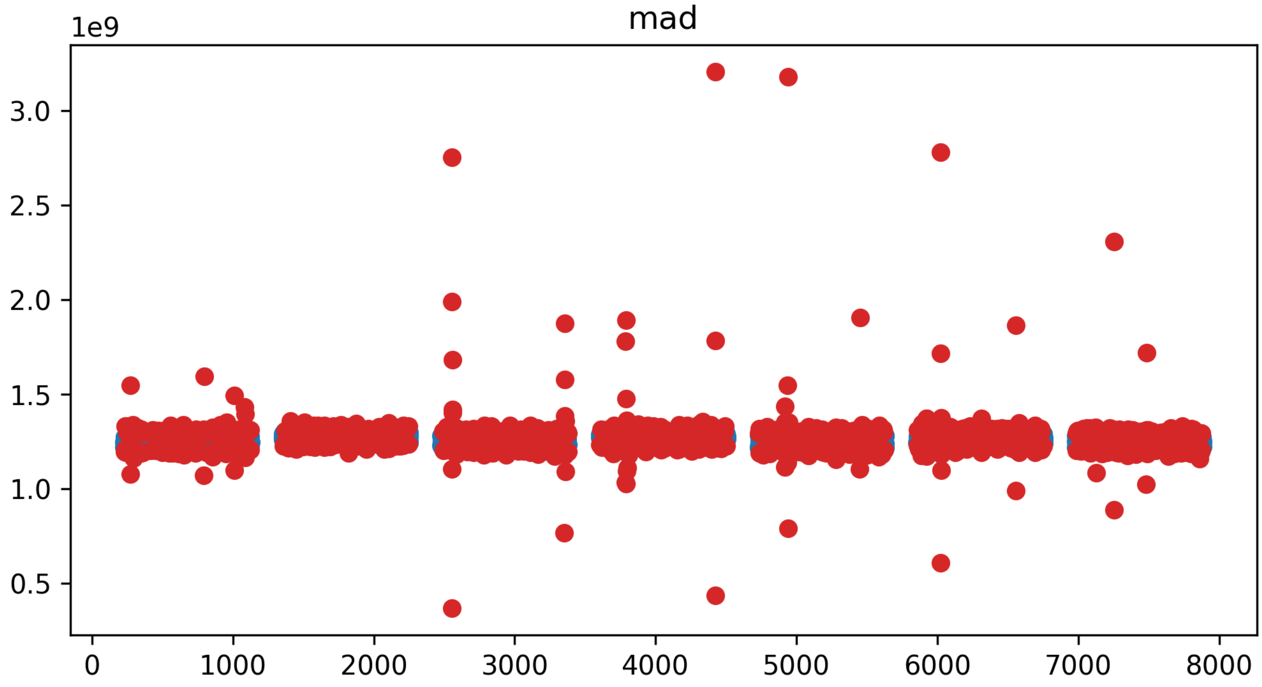

1.3. Total Ion Current (TIC)

TIC of all blocks

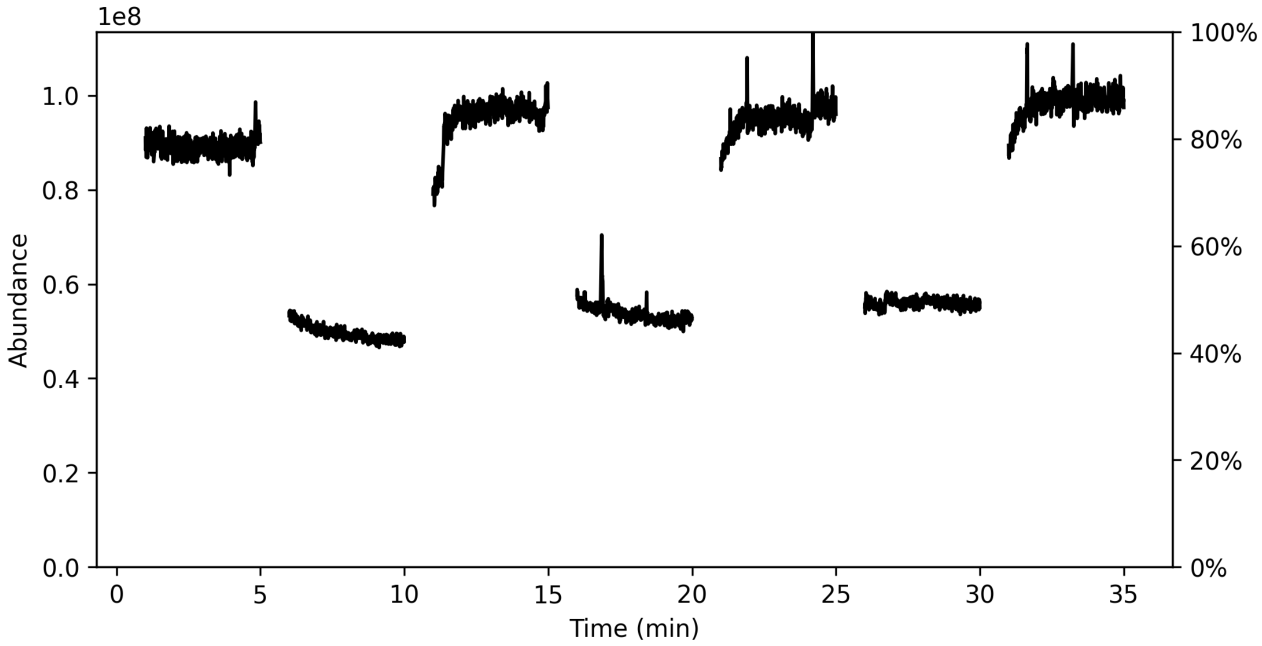

Block	TIC min	TIC max	TIC mean	RSD (%)
1	8.31e+07	9.86e+07	8.91e+07	1.87
2	4.65e+07	5.44e+07	4.96e+07	3.17
3	7.66e+07	1.03e+08	9.52e+07	4.74
4	4.99e+07	7.04e+07	5.38e+07	3.61
5	8.42e+07	1.13e+08	9.48e+07	2.99
6	5.35e+07	5.84e+07	5.60e+07	1.52
7	8.67e+07	1.11e+08	9.83e+07	3.00

2. Block Parameters

The Isotopic Ratio of the blocks were calculated by 'Mean'

2.1. 13C/M0

Block	Number of scans	Effective number of ions	Isotopic Ratio	STD	SEM	RSE
1	744	1.36e+07	0.216760	0.001314	0.000048	0.000222
2	732	1.32e+07	0.216152	0.001363	0.000050	0.000233
3	742	1.34e+07	0.216803	0.001337	0.000049	0.000226
4	712	1.27e+07	0.216154	0.001340	0.000050	0.000232
5	729	1.31e+07	0.216682	0.001383	0.000051	0.000236
6	731	1.30e+07	0.216144	0.001390	0.000051	0.000238
7	726	1.31e+07	0.216932	0.001314	0.000049	0.000225

Errors and Test Paramters

Block	Acquisition Error (permil)	Shot-Noise (permil)	AE/SN ratio	Shapiro Wilk (p_value)	D'Agostino (p_value)
1	0.222	0.271	0.818	0.325	0.842
2	0.233	0.275	0.846	0.449	0.909
3	0.226	0.273	0.829	0.139	0.075
4	0.232	0.281	0.827	0.282	0.513
5	0.236	0.277	0.854	0.443	0.901
6	0.238	0.277	0.858	0.230	0.073
7	0.225	0.276	0.813	0.118	0.299

Isotopic Ratio and Errors of the Blocks

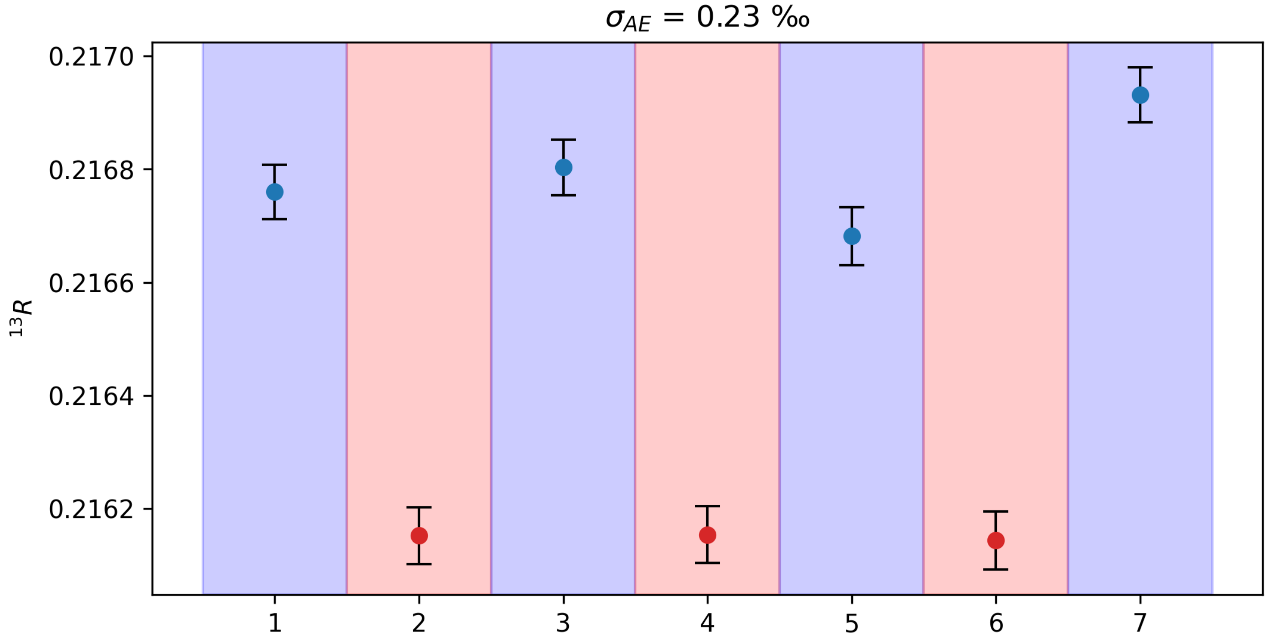

Cumulative Isotopic Ratio

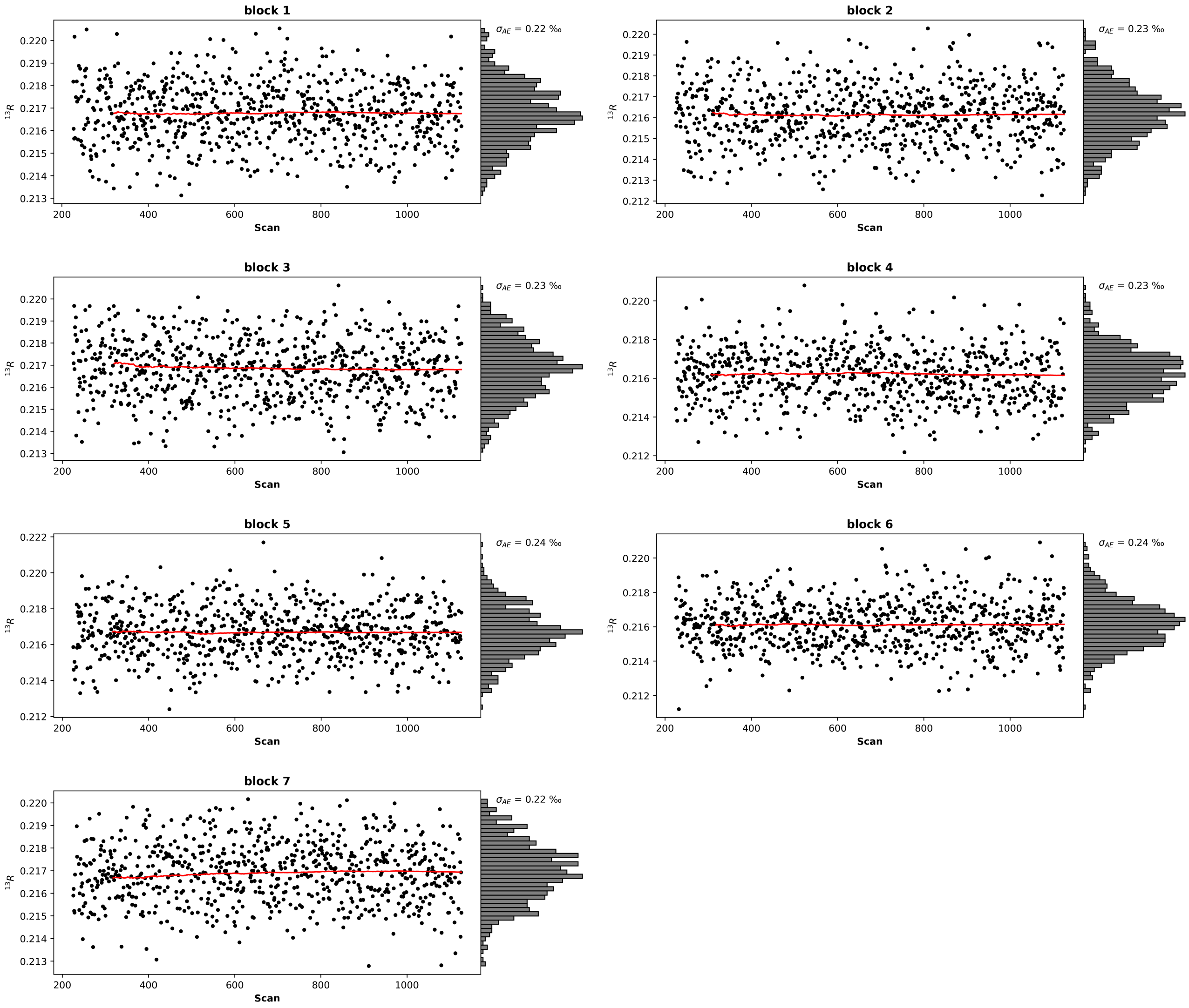

Acquisition Error and Shot-Noise

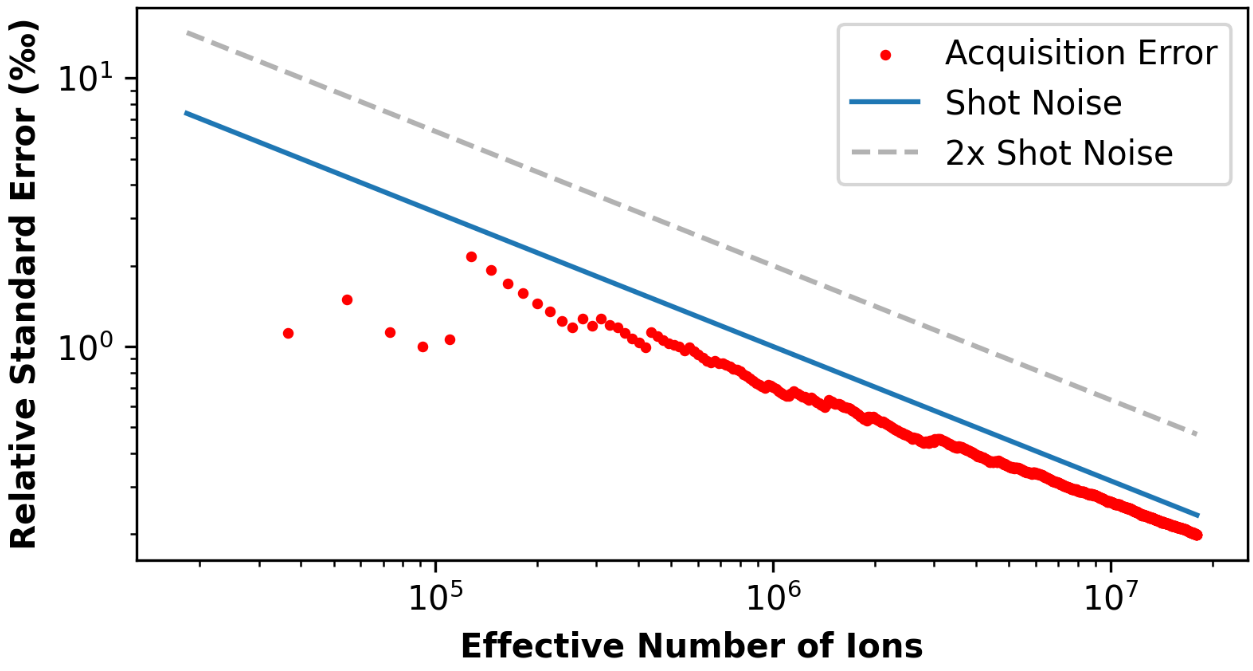

3. Delta Informations

Deltas were calculated by 'Average Of Neighboring Block Ratios'

3.1. 13C

Delta 13C was corrected by -27.80

Block	SEM	Delta corrected	Delta
2	0.23	-30.62	-2.90
4	0.23	-30.44	-2.72
6	0.24	-30.77	-3.06

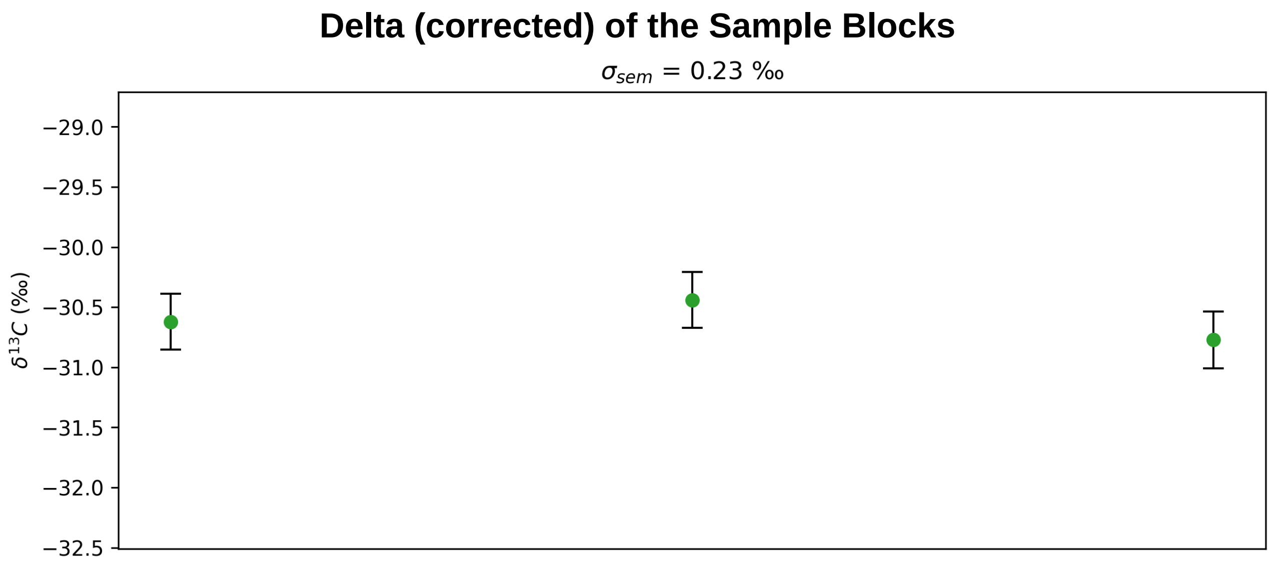

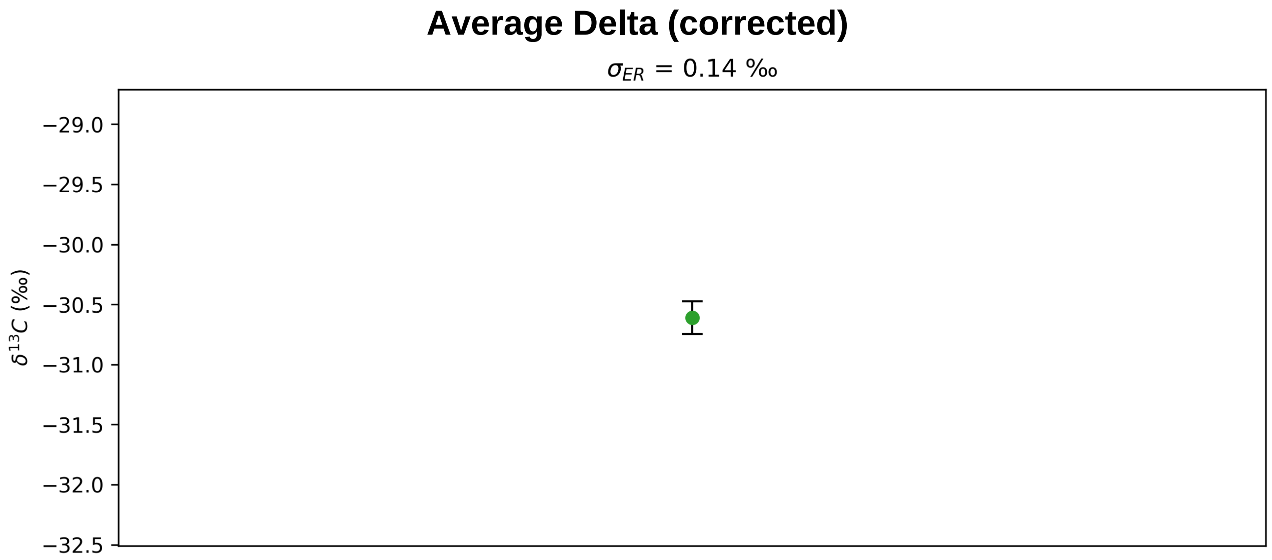

The final corrected average delta was -30.61 with a standard deviation of 0.14. Here the standard deviation is called reproducibility error.
