# Supplementary material for: Advancing Stable Isotope Analysis with Orbitrap-MS for Fatty Acid Methyl Esters and Complex Lipid Matrices
Source: J Am Soc Mass Spectrom. 2025 Jun 17;36(7):1527–35. doi: 10.1021/jasms.5c00092 (PMC12339014; doi:10.1021/jasms.5c00092)
Supplement: Supplementary file 2 [file js5c00092_si_002.zip › reports by IsotoPy Software/standards/H+Standard8_FI.pdf]

Standard 8 - [M + H]+
Isotope Analysis report from IsotoPy

Flow Injection

1. Pre Processing

1.1. Block Time and Scan Information

Information about sample and standard block times and scans:

Block	Injected	Initial Time	End Time	Number of scans
1	standard	1	8	1349
2	sample	16	23	1297
3	standard	31	38	1347
4	sample	46	53	1297
5	standard	61	68	1296
6	sample	76	83	1289
7	standard	91	98	1265

1.2. Outlier Removal

A total of 1899 scans were considered outliers and removed using the MAD method

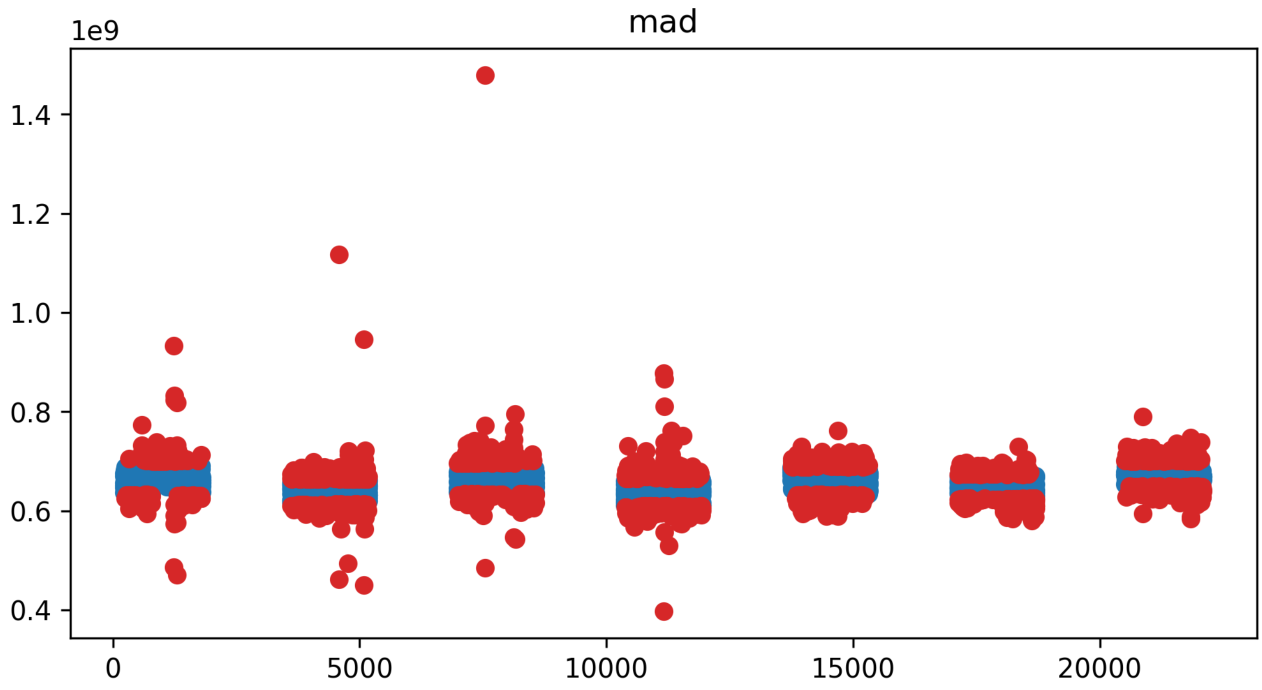

1.3. Total Ion Current (TIC)

TIC of all blocks

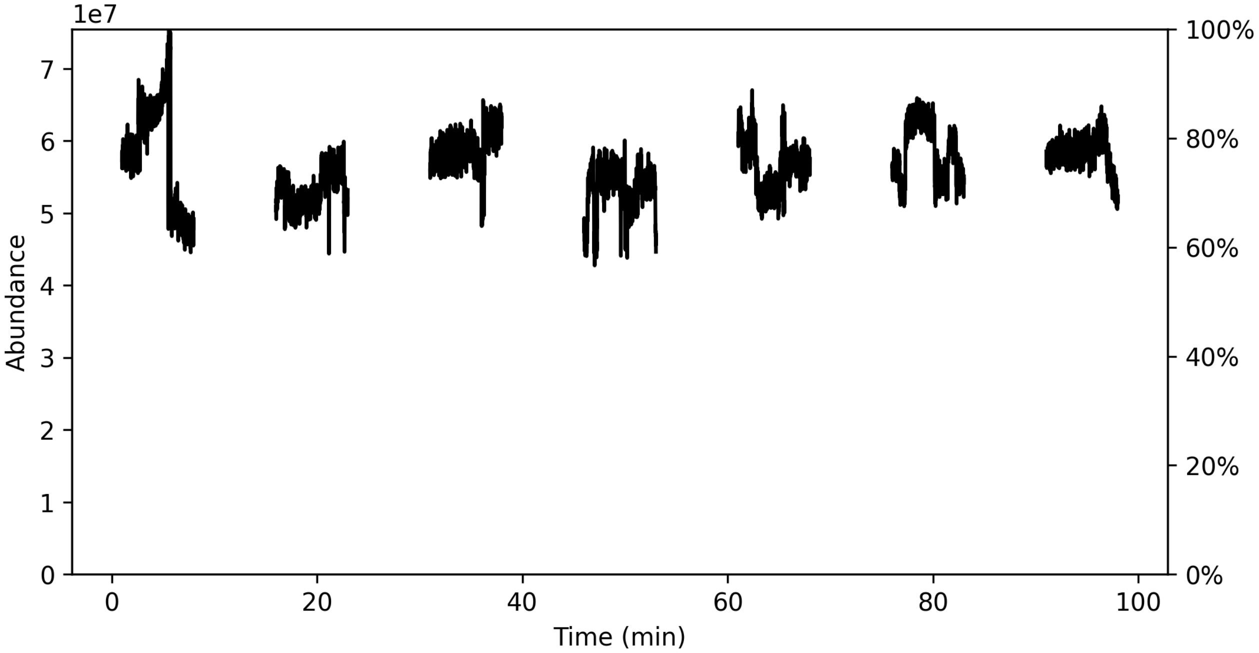

Block	TIC min	TIC max	TIC mean	RSD (%)
1	4.45e+07	7.54e+07	5.79e+07	13.12
2	4.44e+07	5.99e+07	5.31e+07	4.74
3	4.82e+07	6.56e+07	5.89e+07	4.10
4	4.27e+07	6.01e+07	5.34e+07	6.14
5	4.92e+07	6.70e+07	5.63e+07	6.06
6	5.09e+07	6.59e+07	5.88e+07	6.99
7	5.05e+07	6.48e+07	5.85e+07	3.71

2. Block Parameters

The Isotopic Ratio of the blocks were calculated by 'Mean'

2.1. 13C/M0

Block	Number of scans	Effective number of ions	Isotopic Ratio	STD	SEM	RSE
1	1349	1.82e+07	0.209790	0.001816	0.000049	0.000236
2	1297	1.73e+07	0.209889	0.001736	0.000048	0.000230
3	1347	1.81e+07	0.209967	0.001719	0.000047	0.000223
4	1297	1.74e+07	0.209808	0.001759	0.000049	0.000233
5	1296	1.75e+07	0.210036	0.001681	0.000047	0.000222
6	1289	1.71e+07	0.209422	0.001730	0.000048	0.000230
7	1265	1.70e+07	0.209811	0.001770	0.000050	0.000237

Errors and Test Paramters

Block	Acquisition Error (permil)	Shot-Noise (permil)	AE/SN ratio	Shapiro Wilk (p_value)	D'Agostino (p_value)
1	0.236	0.234	1.005	0.283	0.241
2	0.230	0.241	0.955	0.863	0.636
3	0.223	0.235	0.949	0.461	0.929
4	0.233	0.240	0.970	0.288	0.208
5	0.222	0.239	0.929	0.812	0.610
6	0.230	0.242	0.951	0.578	0.289
7	0.237	0.242	0.979	0.873	0.519

Isotopic Ratio and Errors of the Blocks

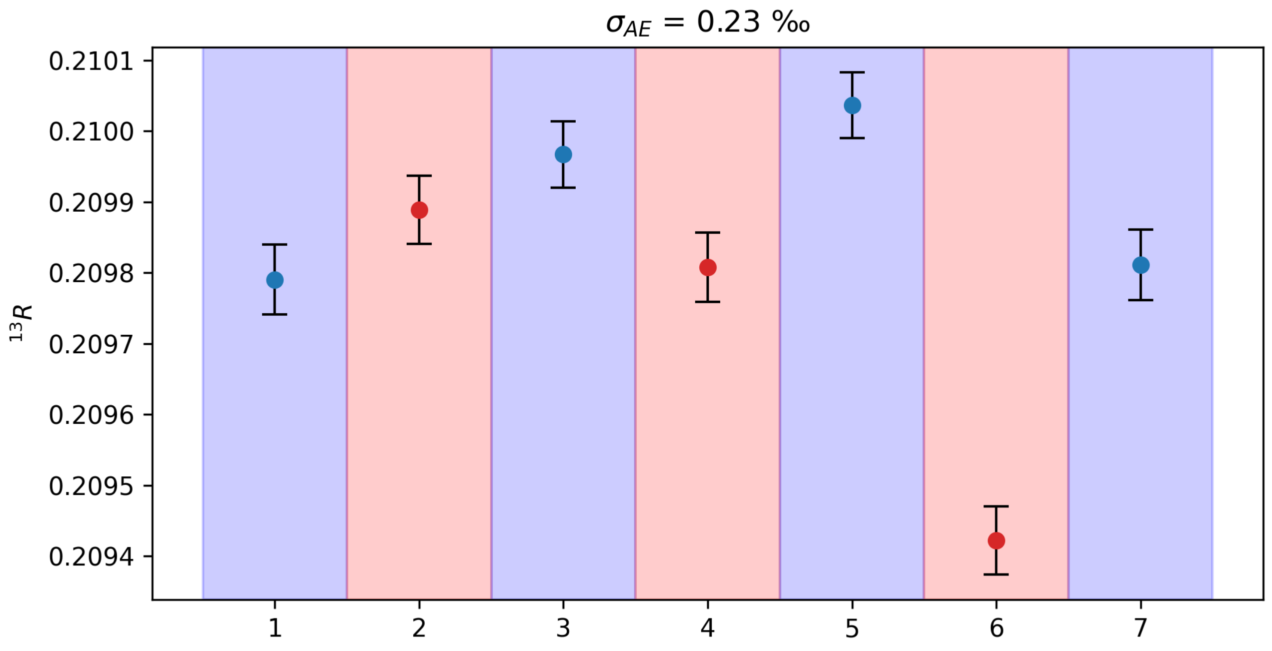

Cumulative Isotopic Ratio

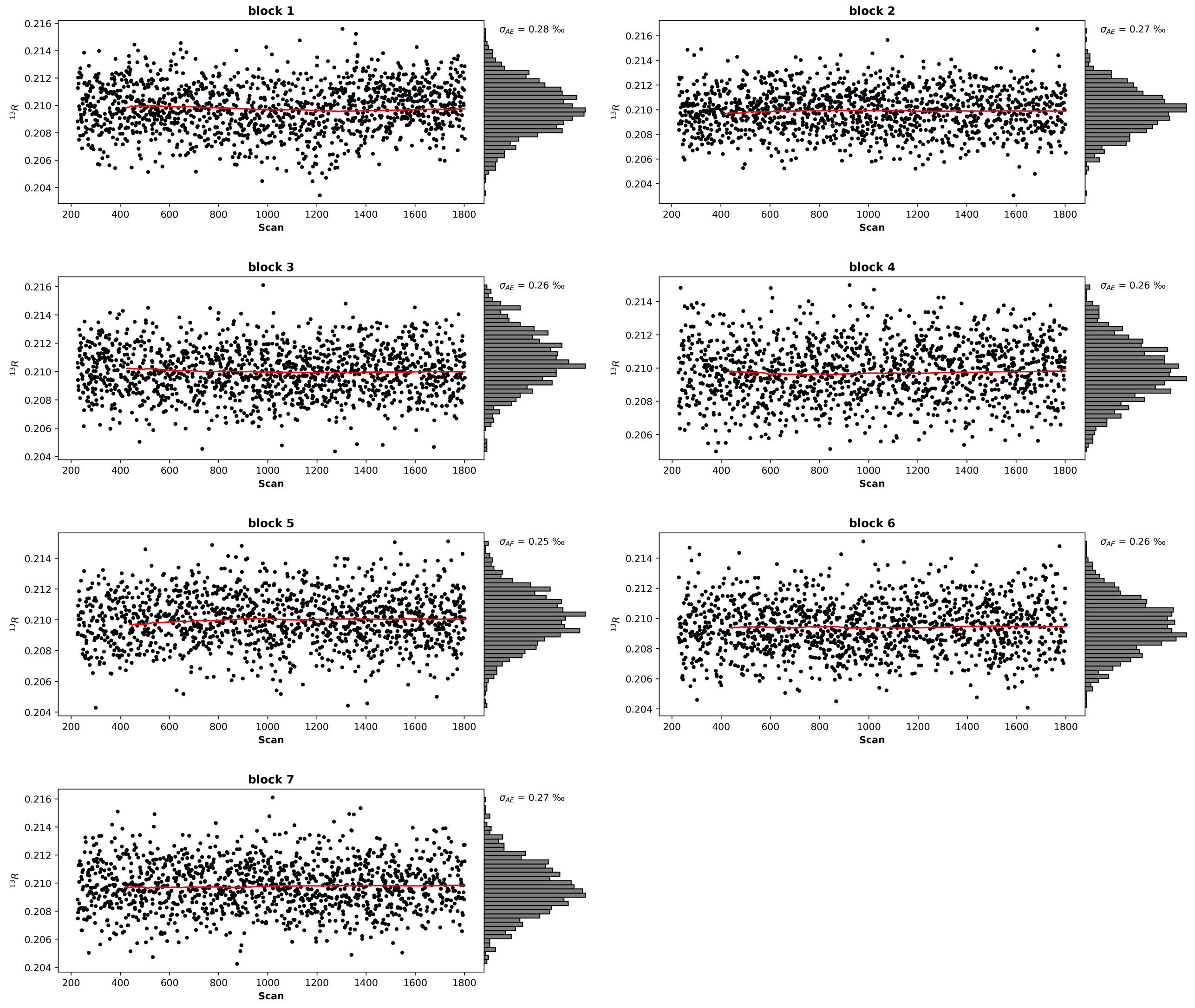

Acquisition Error and Shot-Noise

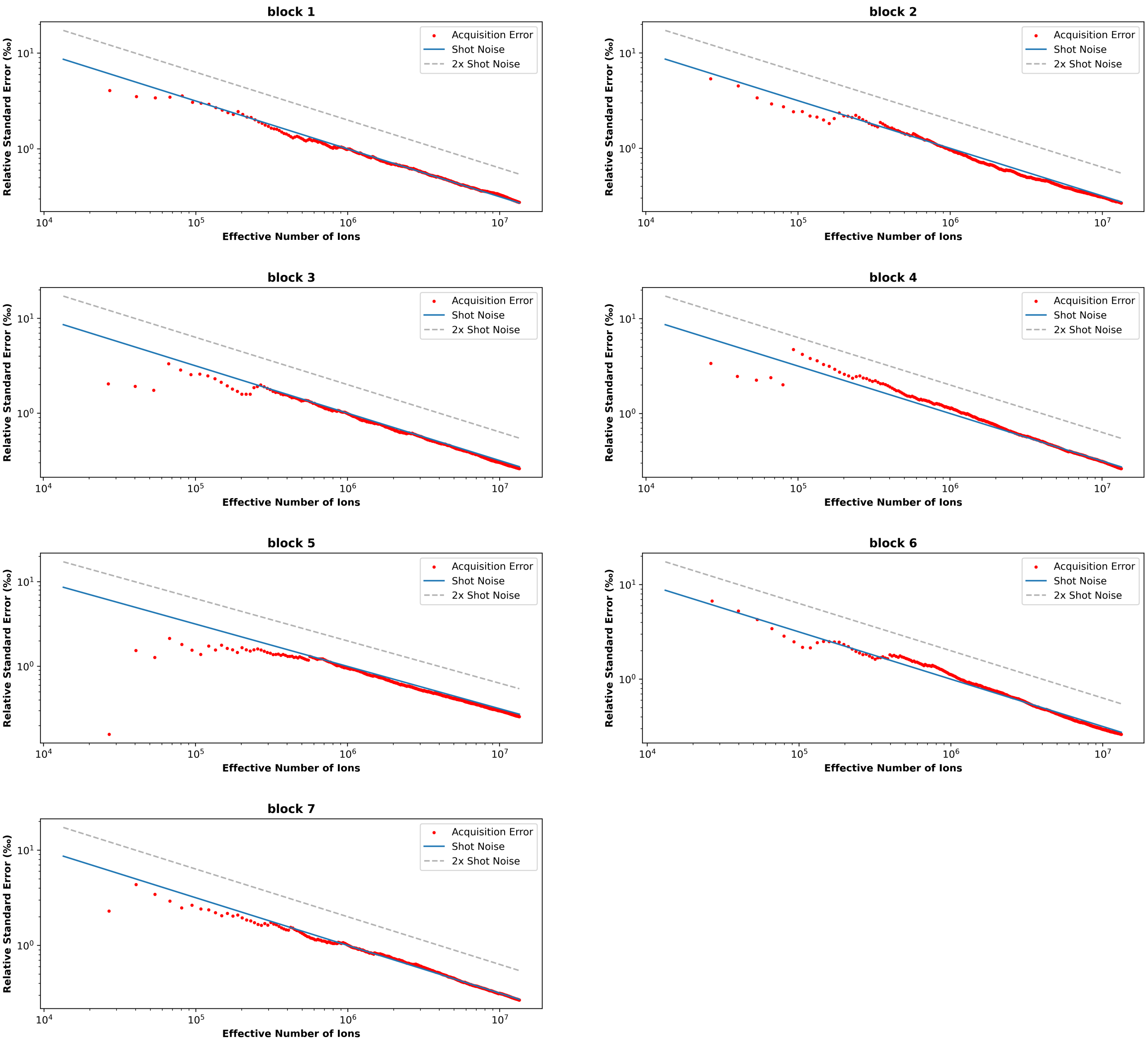

3. Delta Informations

Deltas were calculated by 'Average Of Neighboring Block Ratios'

3.1. 13C

Delta 13C was corrected by -27.80

Block	SEM	Delta corrected	Delta
2	0.23	-27.75	0.05
4	0.23	-28.70	-0.92
6	0.23	-30.12	-2.39

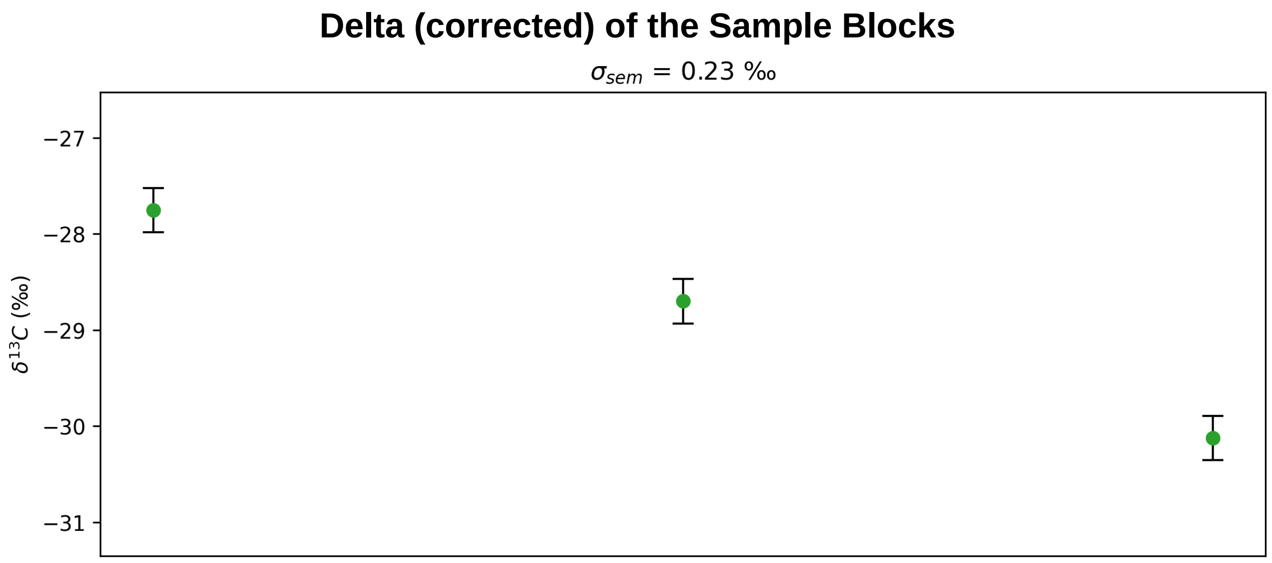

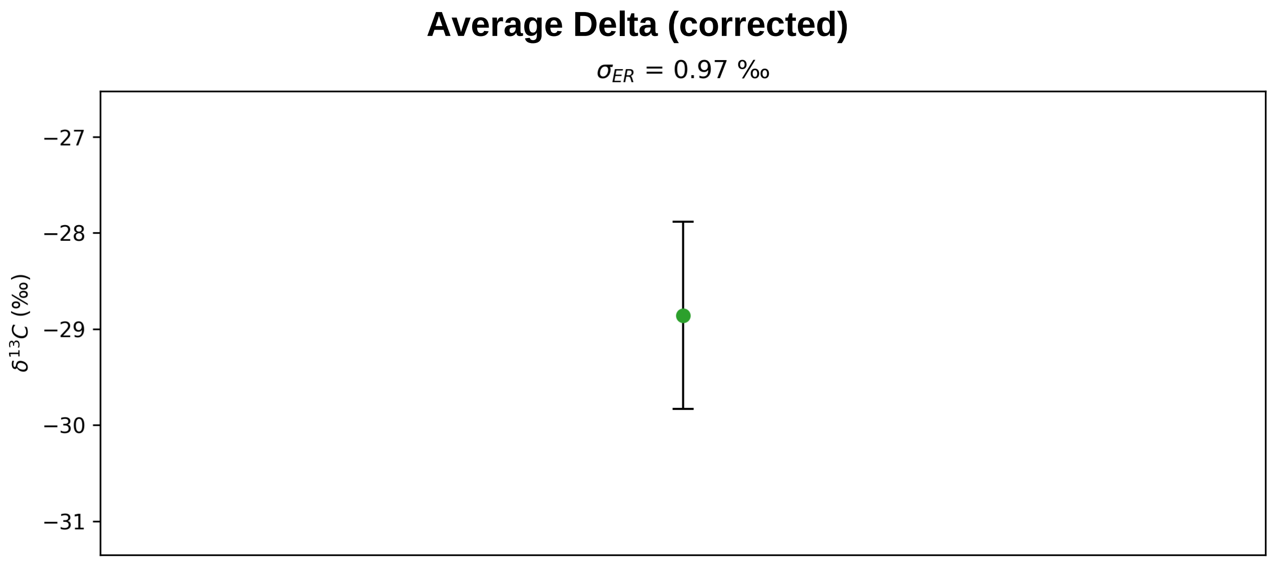

The final corrected average delta was -28.86 with a standard deviation of 0.97. Here the standard deviation is called reproducibility error.
