# Supplementary material for: Advancing Stable Isotope Analysis with Orbitrap-MS for Fatty Acid Methyl Esters and Complex Lipid Matrices
Source: J Am Soc Mass Spectrom. 2025 Jun 17;36(7):1527–35. doi: 10.1021/jasms.5c00092 (PMC12339014; doi:10.1021/jasms.5c00092)
Supplement: Supplementary file 2 [file js5c00092_si_002.zip › reports by IsotoPy Software/standards/H+Standard4_FI.pdf]

Standard 4 - [M + H]+
Isotope Analysis report from IsotoPy

Flow Injection

1. Pre Processing

1.1. Block Time and Scan Information

Information about sample and standard block times and scans:

Block	Injected	Initial Time	End Time	Number of scans
1	standard	1	8	1305
2	sample	16	23	1284
3	standard	31	38	1309
4	sample	46	53	1275
5	standard	61	68	1302
6	sample	76	83	1293
7	standard	91	98	1291

1.2. Outlier Removal

A total of 1980 scans were considered outliers and removed using the MAD method

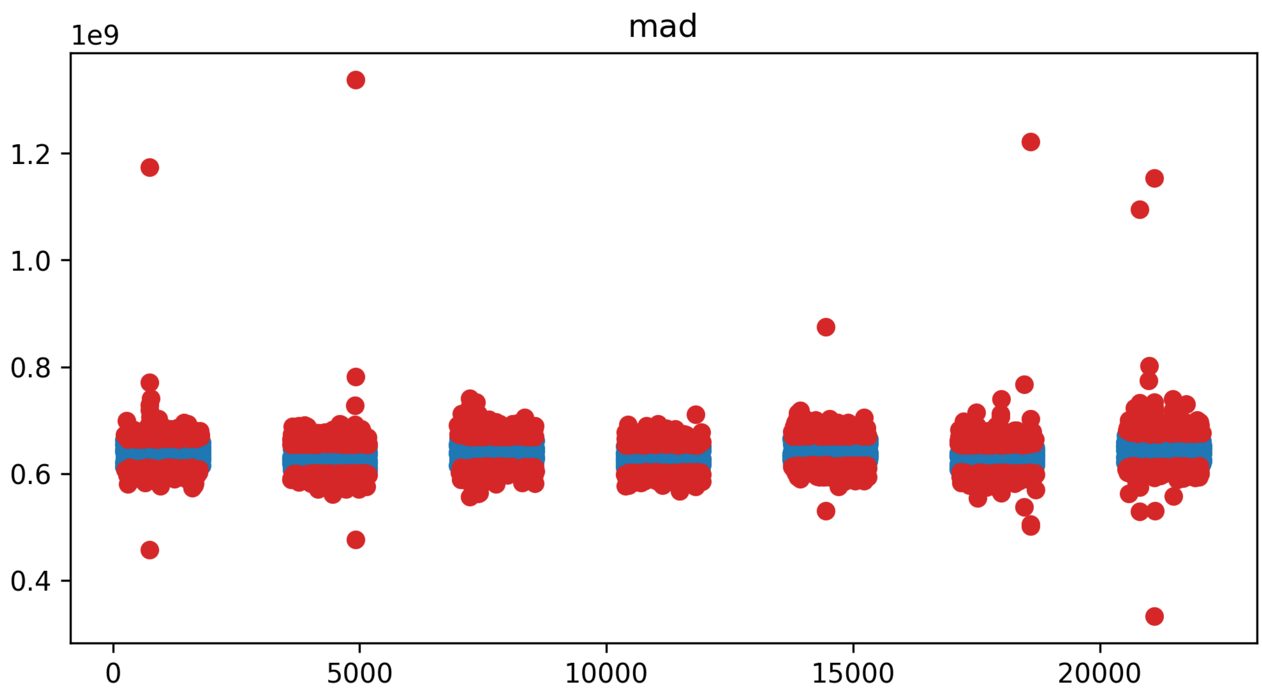

1.3. Total Ion Current (TIC)

TIC of all blocks

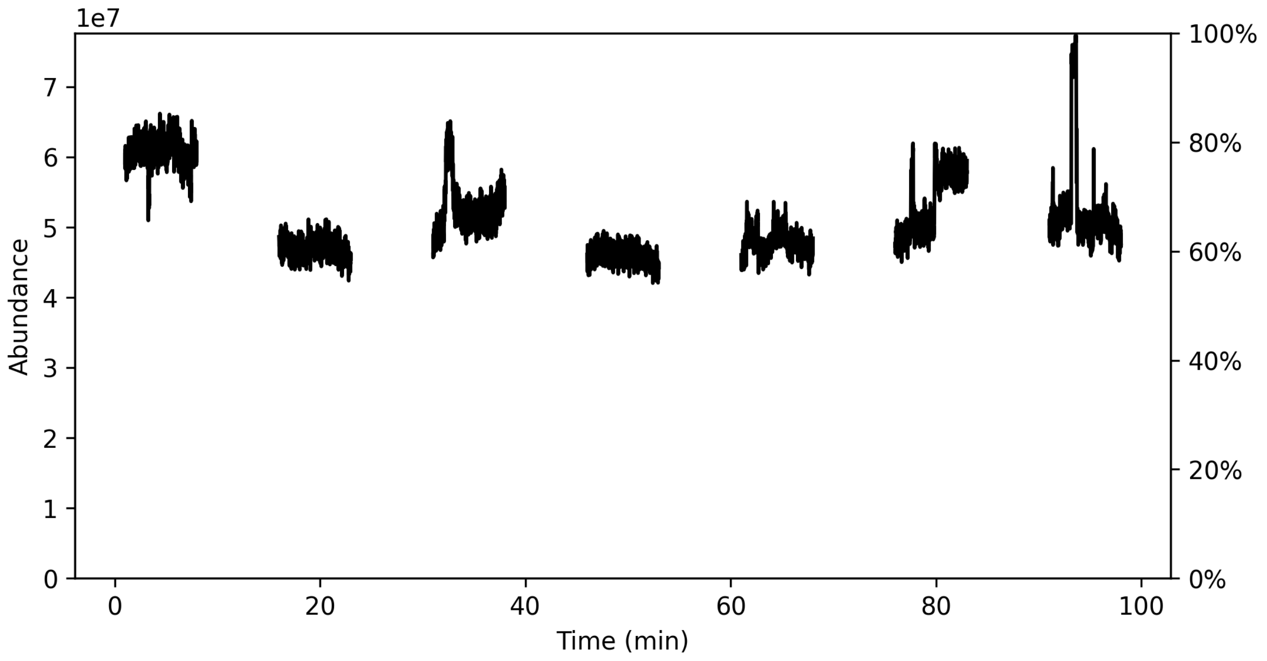

Block	TIC min	TIC max	TIC mean	RSD (%)
1	5.10e+07	6.62e+07	6.08e+07	3.12
2	4.24e+07	5.11e+07	4.70e+07	2.74
3	4.57e+07	6.51e+07	5.26e+07	6.47
4	4.20e+07	4.95e+07	4.60e+07	2.53
5	4.32e+07	5.36e+07	4.79e+07	3.60
6	4.50e+07	6.19e+07	5.37e+07	8.34
7	4.52e+07	7.76e+07	5.15e+07	9.47

2. Block Parameters

The Isotopic Ratio of the blocks were calculated by 'Mean'

2.1. 13C/M0

Block	Number of scans	Effective number of ions	Isotopic Ratio	STD	SEM	RSE
1	1305	1.75e+07	0.209958	0.001786	0.000049	0.000235
2	1284	1.72e+07	0.210007	0.001756	0.000049	0.000233
3	1309	1.75e+07	0.210136	0.001778	0.000049	0.000234
4	1275	1.71e+07	0.210072	0.001757	0.000049	0.000234
5	1302	1.74e+07	0.210195	0.001685	0.000047	0.000222
6	1293	1.73e+07	0.209839	0.001722	0.000048	0.000228
7	1291	1.72e+07	0.209945	0.001780	0.000050	0.000236

Errors and Test Paramters

Block	Acquisition Error (permil)	Shot-Noise (permil)	AE/SN ratio	Shapiro Wilk (p_value)	D'Agostino (p_value)
1	0.235	0.239	0.983	0.720	0.737
2	0.233	0.241	0.967	0.032	0.059
3	0.234	0.239	0.978	0.339	0.577
4	0.234	0.242	0.967	0.008	0.016
5	0.222	0.240	0.927	0.711	0.865
6	0.228	0.241	0.949	0.599	0.532
7	0.236	0.241	0.979	0.774	0.997

Isotopic Ratio and Errors of the Blocks

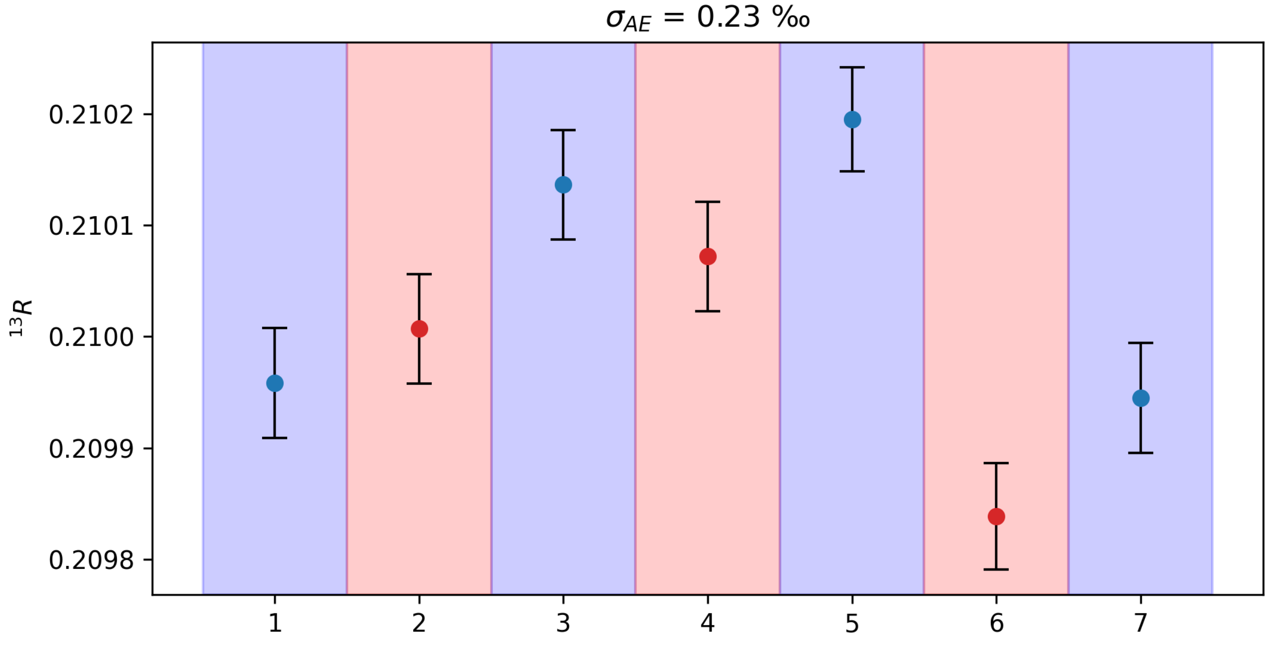

Cumulative Isotopic Ratio

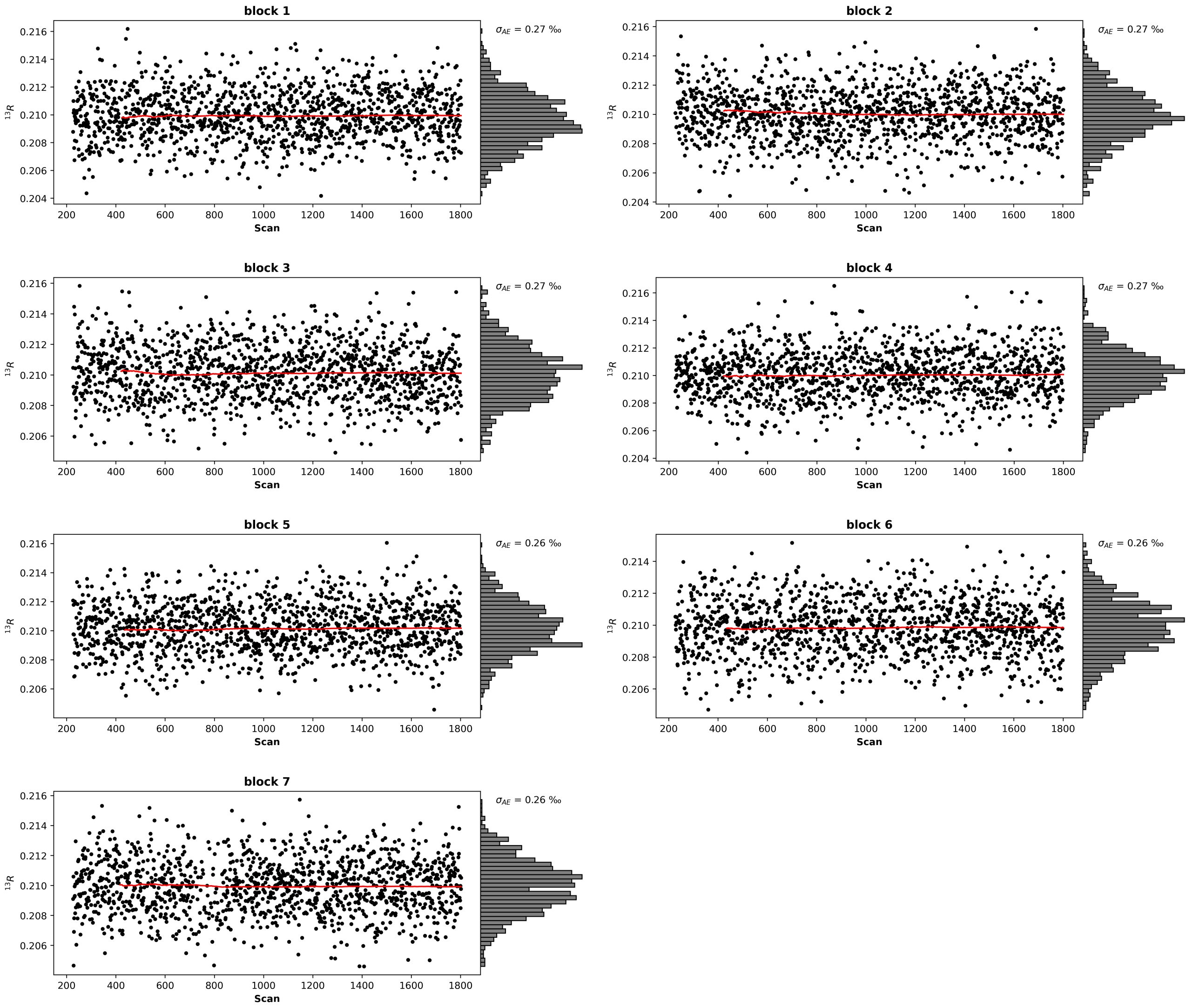

Acquisition Error and Shot-Noise

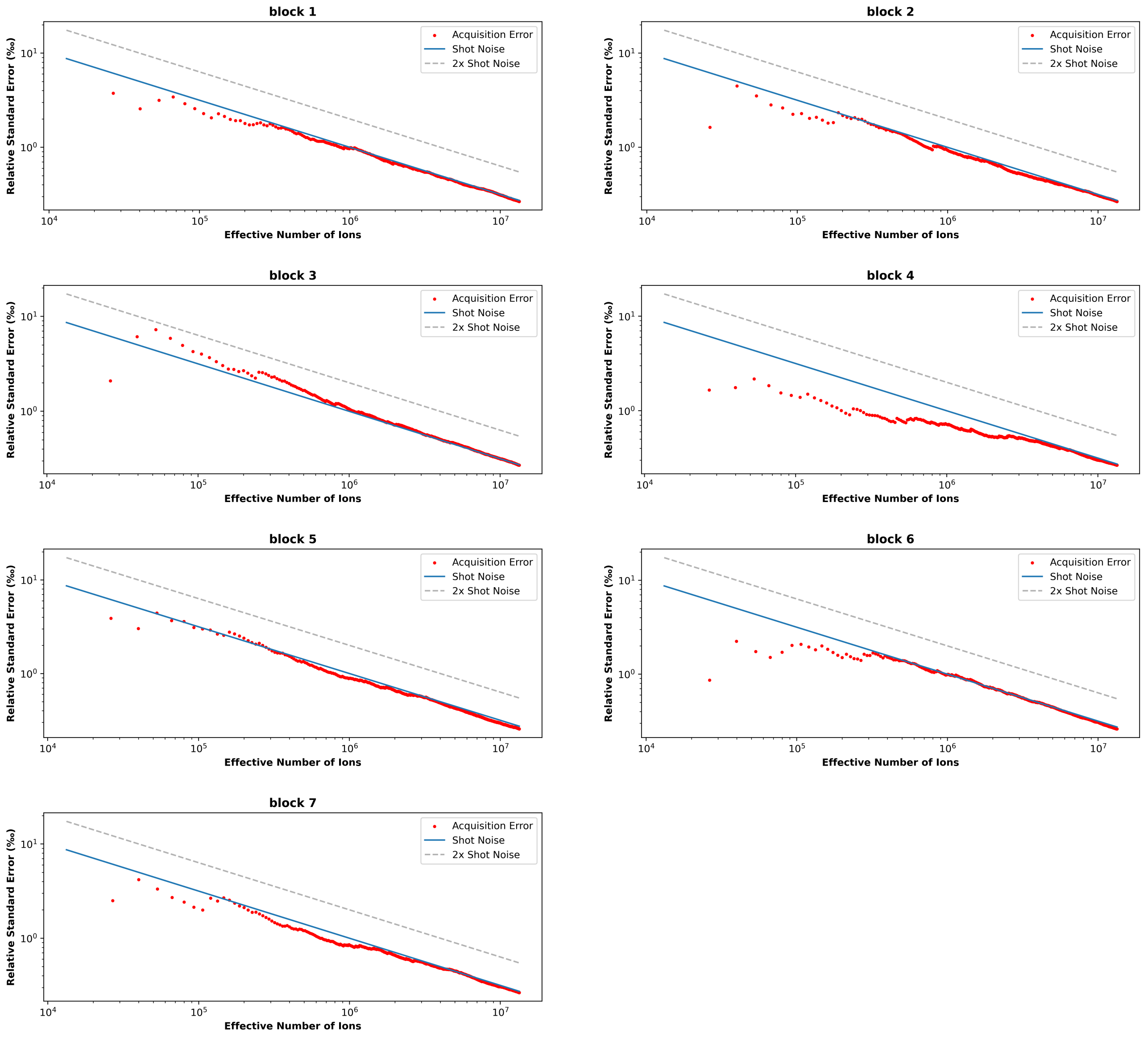

3. Delta Informations

Deltas were calculated by 'Average Of Neighboring Block Ratios'

3.1. 13C

Delta 13C was corrected by -27.80

Block	SEM	Delta corrected	Delta
2	0.23	-27.99	-0.19
4	0.23	-28.23	-0.45
6	0.23	-28.87	-1.10

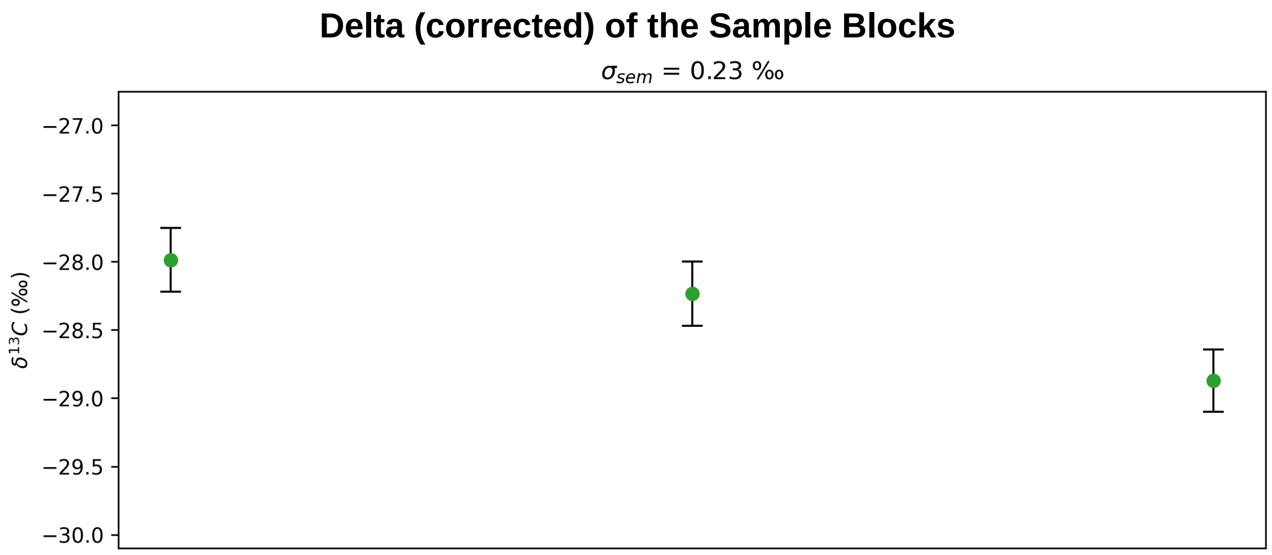

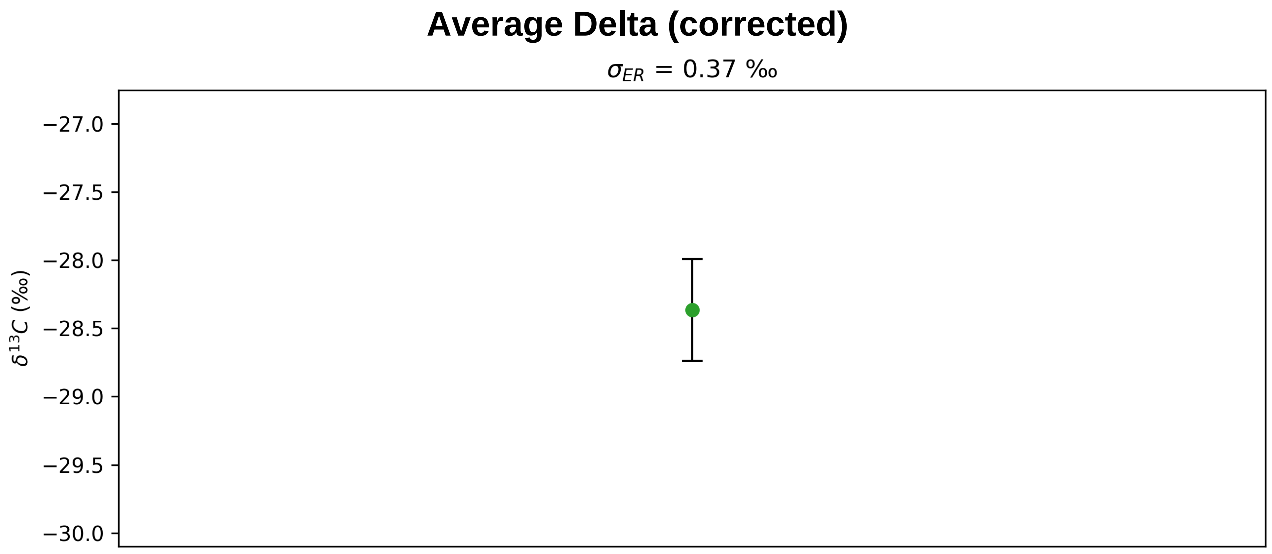

The final corrected average delta was -28.36 with a standard deviation of 0.37. Here the standard deviation is called reproducibility error.
