# Supplementary material for: Advancing Stable Isotope Analysis with Orbitrap-MS for Fatty Acid Methyl Esters and Complex Lipid Matrices
Source: J Am Soc Mass Spectrom. 2025 Jun 17;36(7):1527–35. doi: 10.1021/jasms.5c00092 (PMC12339014; doi:10.1021/jasms.5c00092)
Supplement: Supplementary file 2 [file js5c00092_si_002.zip › reports by IsotoPy Software/standards/H+Standard3_DI.pdf]

Standard 3 - [M + H]+
Isotope Analysis report from IsotoPy

Dual Inlet

1. Pre Processing

1.1. Block Time and Scan Information

Information about sample and standard block times and scans:

Block	Injected	Initial Time	End Time	Number of scans
1	standard	1	5	750
2	sample	6	10	761
3	standard	11	15	752
4	sample	16	20	717
5	standard	21	25	733
6	sample	26	30	734
7	standard	31	35	751

1.2. Outlier Removal

A total of 1105 scans were considered outliers and removed using the MAD method

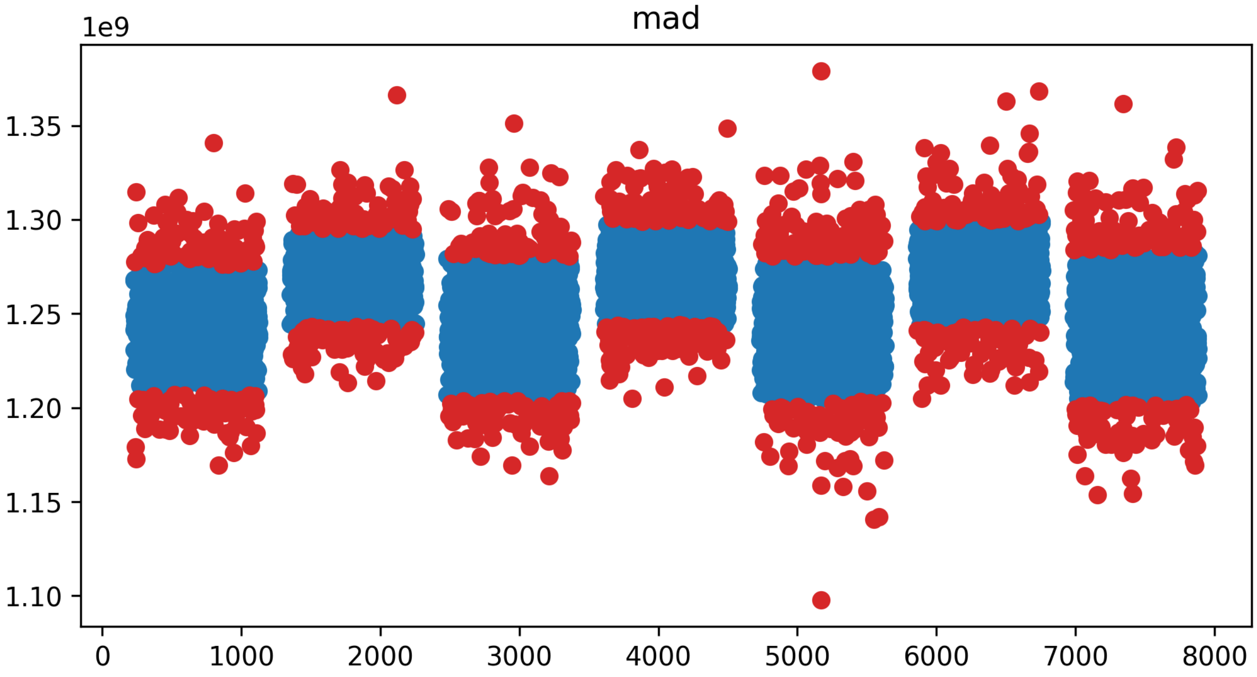

1.3. Total Ion Current (TIC)

TIC of all blocks

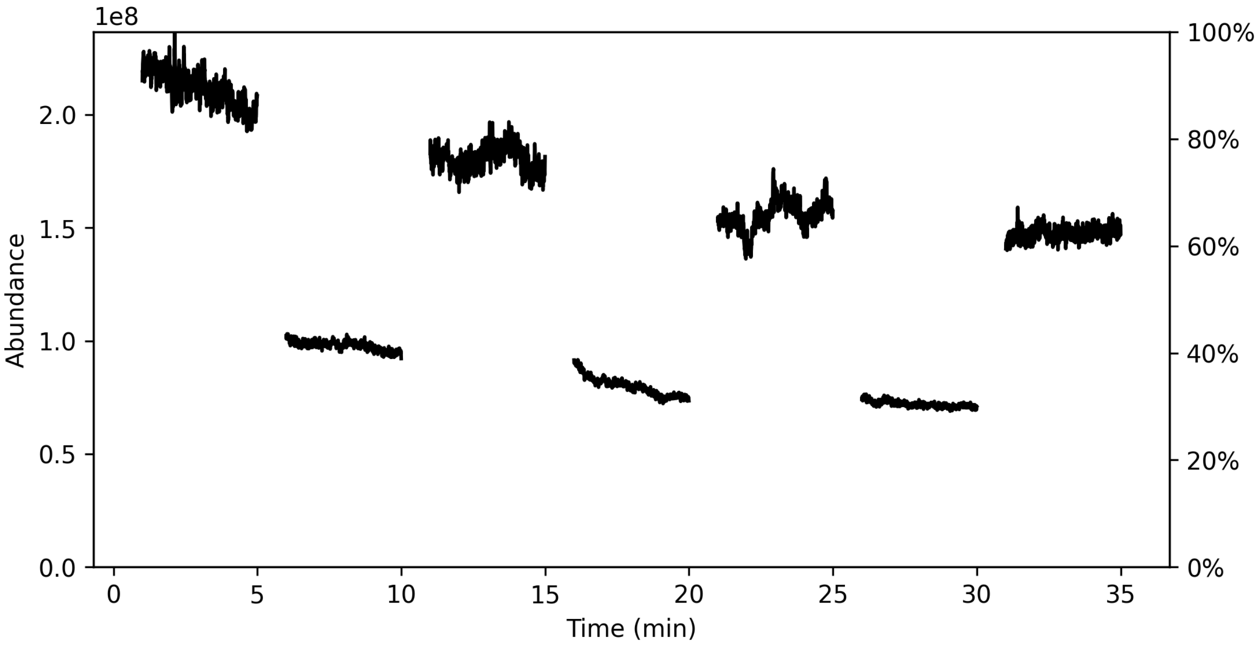

Block	TIC min	TIC max	TIC mean	RSD (%)
1	1.93e+08	2.37e+08	2.11e+08	3.84
2	9.22e+07	1.03e+08	9.79e+07	2.09
3	1.66e+08	1.97e+08	1.81e+08	3.05
4	7.23e+07	9.16e+07	7.98e+07	5.30
5	1.36e+08	1.76e+08	1.56e+08	4.08
6	6.89e+07	7.64e+07	7.21e+07	1.93
7	1.40e+08	1.59e+08	1.48e+08	2.03

2. Block Parameters

The Isotopic Ratio of the blocks were calculated by 'Mean'

2.1. 13C/M0

Block	Number of scans	Effective number of ions	Isotopic Ratio	STD	SEM	RSE
1	750	1.38e+07	0.216102	0.001308	0.000048	0.000221
2	761	1.39e+07	0.215654	0.001377	0.000050	0.000231
3	752	1.39e+07	0.216397	0.001407	0.000051	0.000237
4	717	1.32e+07	0.216022	0.001355	0.000051	0.000234
5	733	1.36e+07	0.216627	0.001324	0.000049	0.000226
6	734	1.35e+07	0.216181	0.001390	0.000051	0.000237
7	751	1.39e+07	0.216667	0.001385	0.000051	0.000233

Errors and Test Paramters

Block	Acquisition Error (permil)	Shot-Noise (permil)	AE/SN ratio	Shapiro Wilk (p_value)	D'Agostino (p_value)
1	0.221	0.269	0.821	0.508	0.555
2	0.231	0.268	0.863	0.758	0.515
3	0.237	0.268	0.883	0.501	0.797
4	0.234	0.276	0.850	0.857	0.963
5	0.226	0.272	0.831	0.372	0.117
6	0.237	0.272	0.871	0.290	0.778
7	0.233	0.269	0.868	0.204	0.182

Isotopic Ratio and Errors of the Blocks

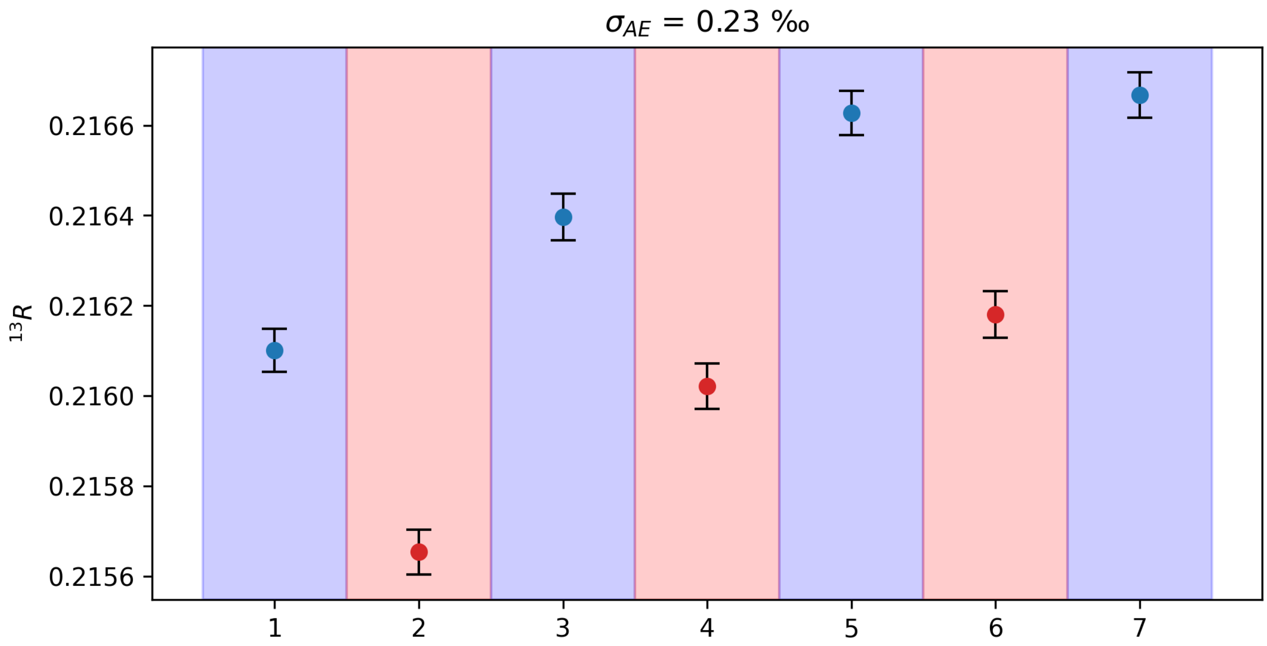

Cumulative Isotopic Ratio

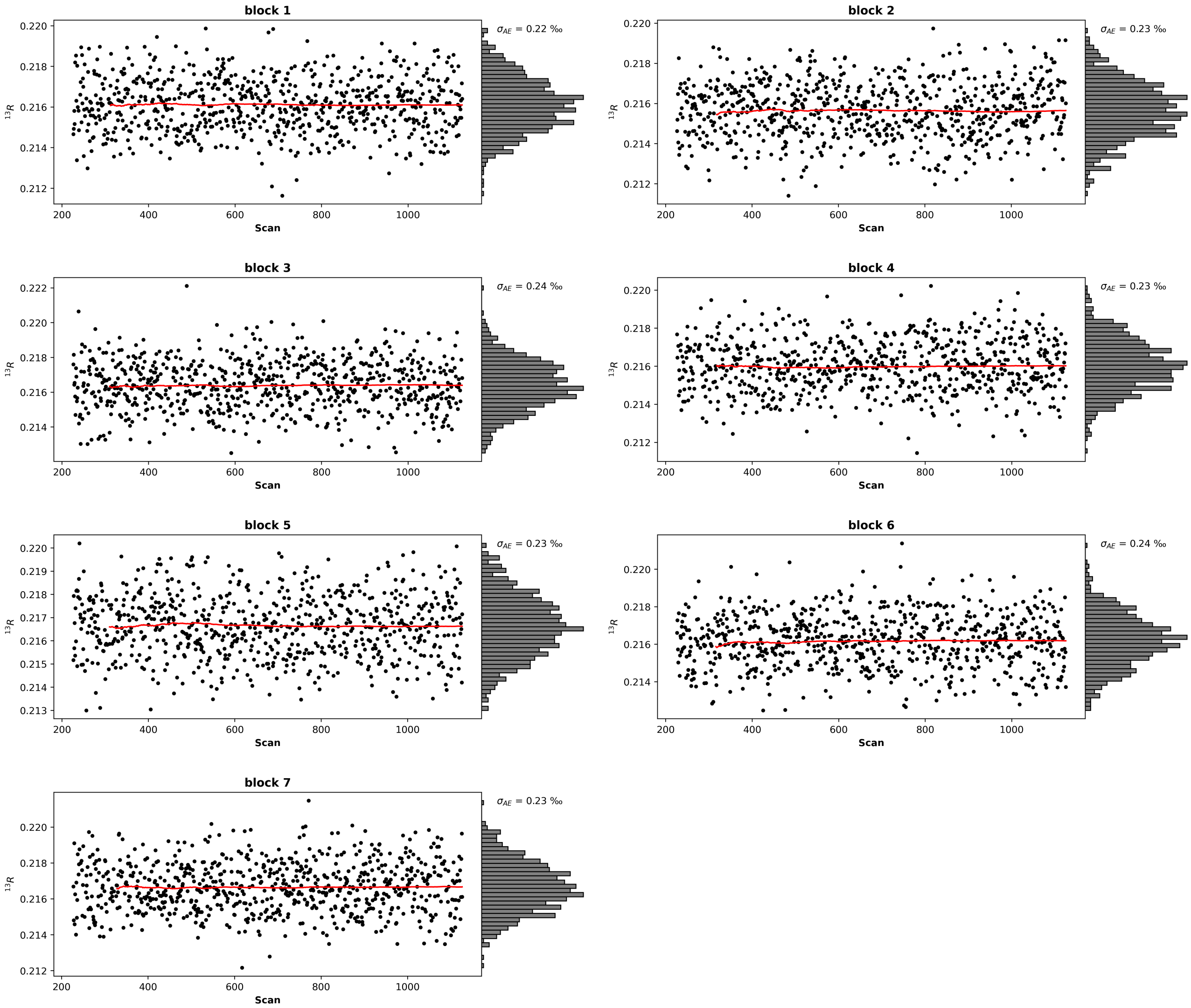

Acquisition Error and Shot-Noise

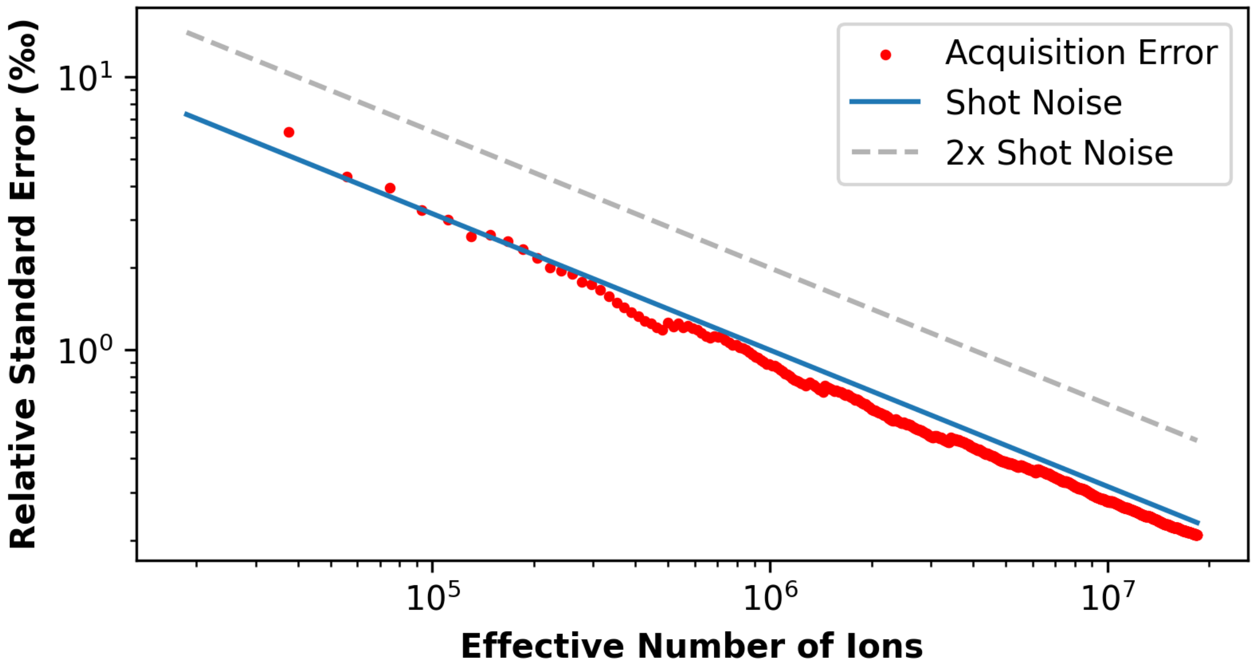

3. Delta Informations

Deltas were calculated by 'Average Of Neighboring Block Ratios'

3.1. 13C

Delta 13C was corrected by -27.80

Block	SEM	Delta corrected	Delta
2	0.23	-30.47	-2.75
4	0.23	-30.00	-2.26
6	0.24	-29.89	-2.15

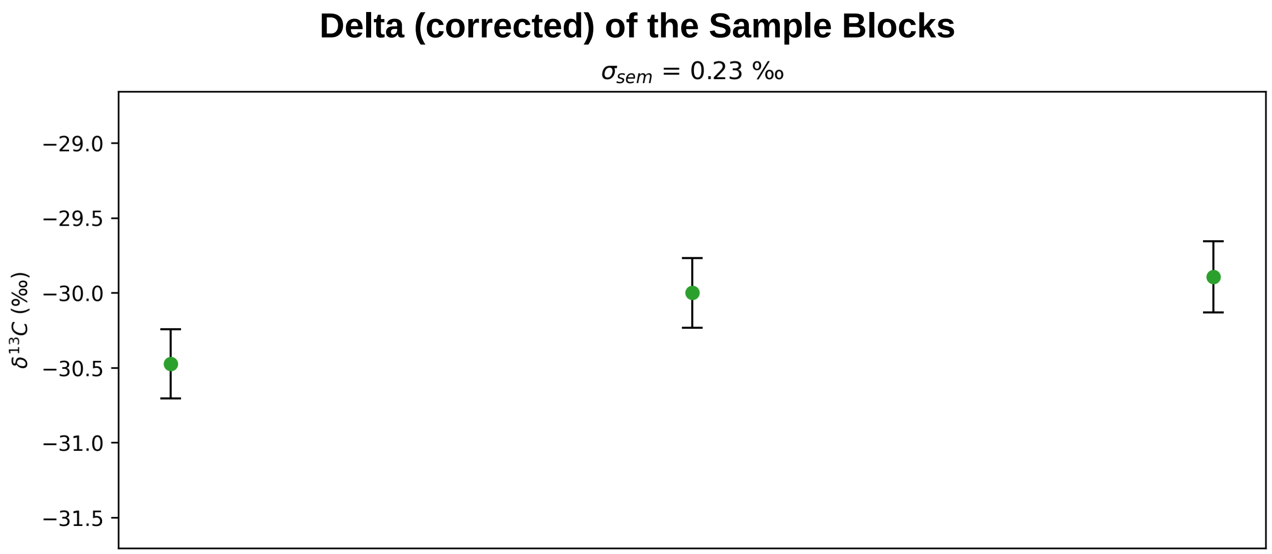

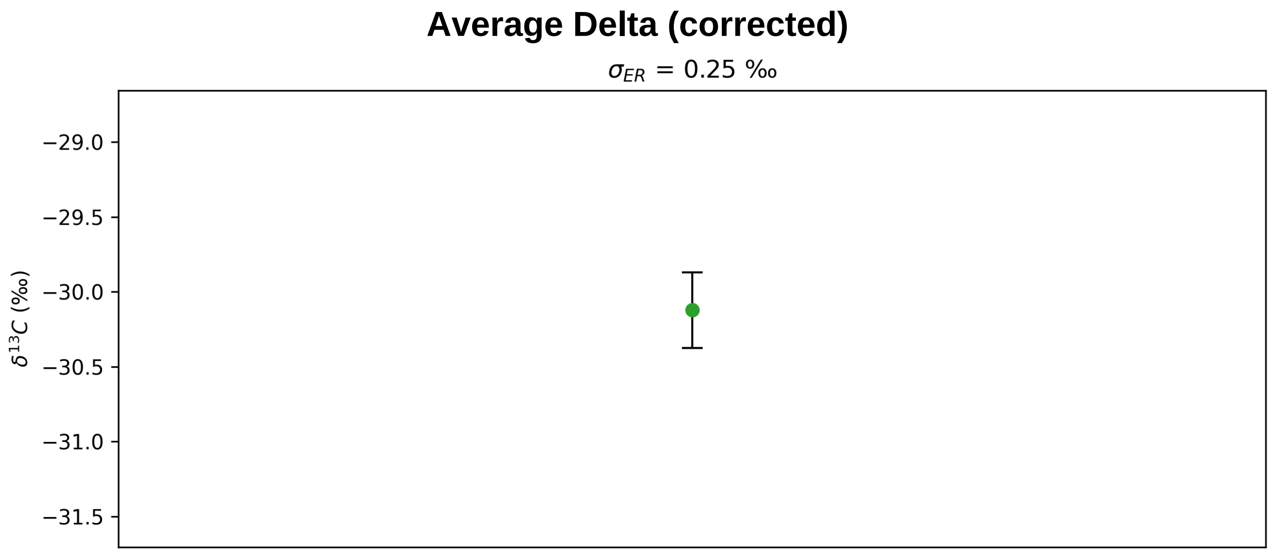

The final corrected average delta was -30.12 with a standard deviation of 0.25. Here the standard deviation is called reproducibility error.
