# Supplementary material for: Advancing Stable Isotope Analysis with Orbitrap-MS for Fatty Acid Methyl Esters and Complex Lipid Matrices
Source: J Am Soc Mass Spectrom. 2025 Jun 17;36(7):1527–35. doi: 10.1021/jasms.5c00092 (PMC12339014; doi:10.1021/jasms.5c00092)
Supplement: Supplementary file 2 [file js5c00092_si_002.zip › reports by IsotoPy Software/standards/Na+Standard1_FI.pdf]

Standard 1 - [M + Na]+
Isotope Analysis report from IsotoPy

Flow Injection

1. Pre Processing

1.1. Block Time and Scan Information

Information about sample and standard block times and scans:

Block	Injected	Initial Time	End Time	Number of scans
1	standard	1	8	1262
2	sample	16	23	1291
3	standard	31	38	1342
4	sample	46	53	1318
5	standard	61	68	1287
6	sample	76	83	1287
7	standard	91	98	1293

1.2. Outlier Removal

A total of 1959 scans were considered outliers and removed using the MAD method

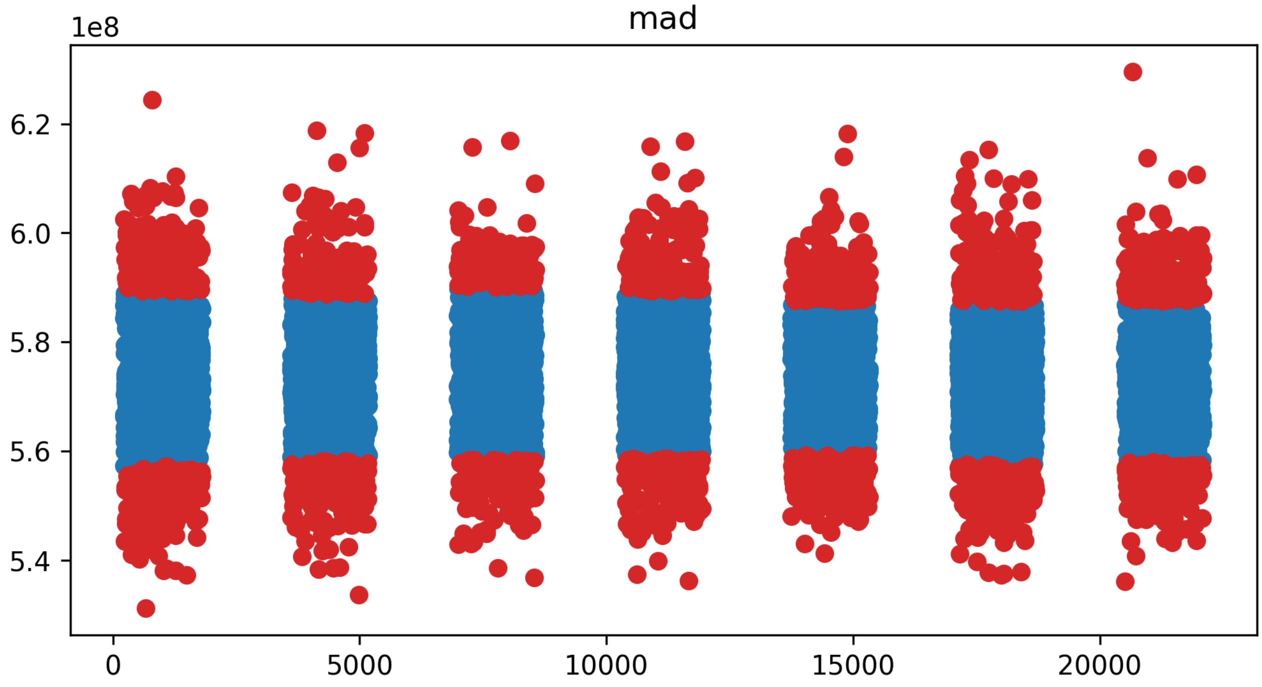

1.3. Total Ion Current (TIC)

TIC of all blocks

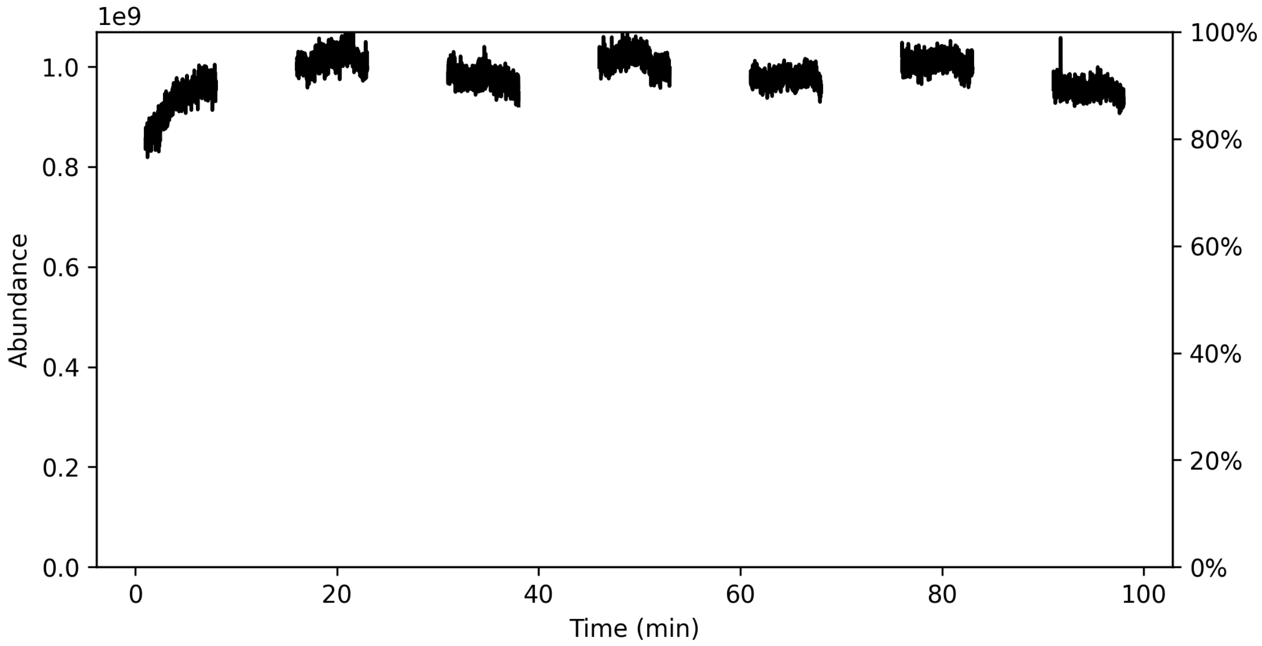

Block	TIC min	TIC max	TIC mean	RSD (%)
1	8.19e+08	1.00e+09	9.29e+08	4.23
2	9.58e+08	1.07e+09	1.01e+09	1.73
3	9.22e+08	1.04e+09	9.77e+08	1.70
4	9.58e+08	1.07e+09	1.01e+09	1.82
5	9.30e+08	1.02e+09	9.79e+08	1.36
6	9.59e+08	1.05e+09	1.01e+09	1.38
7	9.07e+08	1.06e+09	9.55e+08	1.49

2. Block Parameters

The Isotopic Ratio of the blocks were calculated by 'Mean'

2.1. 13C/M0

Block	Number of scans	Effective number of ions	Isotopic Ratio	STD	SEM	RSE
1	1262	2.07e+07	0.208760	0.001719	0.000048	0.000232
2	1291	2.11e+07	0.208699	0.001748	0.000049	0.000233
3	1342	2.19e+07	0.209100	0.001701	0.000046	0.000222
4	1318	2.15e+07	0.208669	0.001787	0.000049	0.000236
5	1287	2.11e+07	0.208922	0.001767	0.000049	0.000236
6	1287	2.11e+07	0.208646	0.001765	0.000049	0.000236
7	1293	2.12e+07	0.208956	0.001798	0.000050	0.000239

Errors and Test Paramters

Block	Acquisition Error (permil)	Shot-Noise (permil)	AE/SN ratio	Shapiro Wilk (p_value)	D'Agostino (p_value)
1	0.232	0.220	1.054	0.298	0.379
2	0.233	0.218	1.071	0.465	0.632
3	0.222	0.214	1.040	0.146	0.383
4	0.236	0.216	1.093	0.410	0.757
5	0.236	0.218	1.082	0.202	0.045
6	0.236	0.218	1.083	0.351	0.253
7	0.239	0.217	1.102	0.676	0.385

Isotopic Ratio and Errors of the Blocks

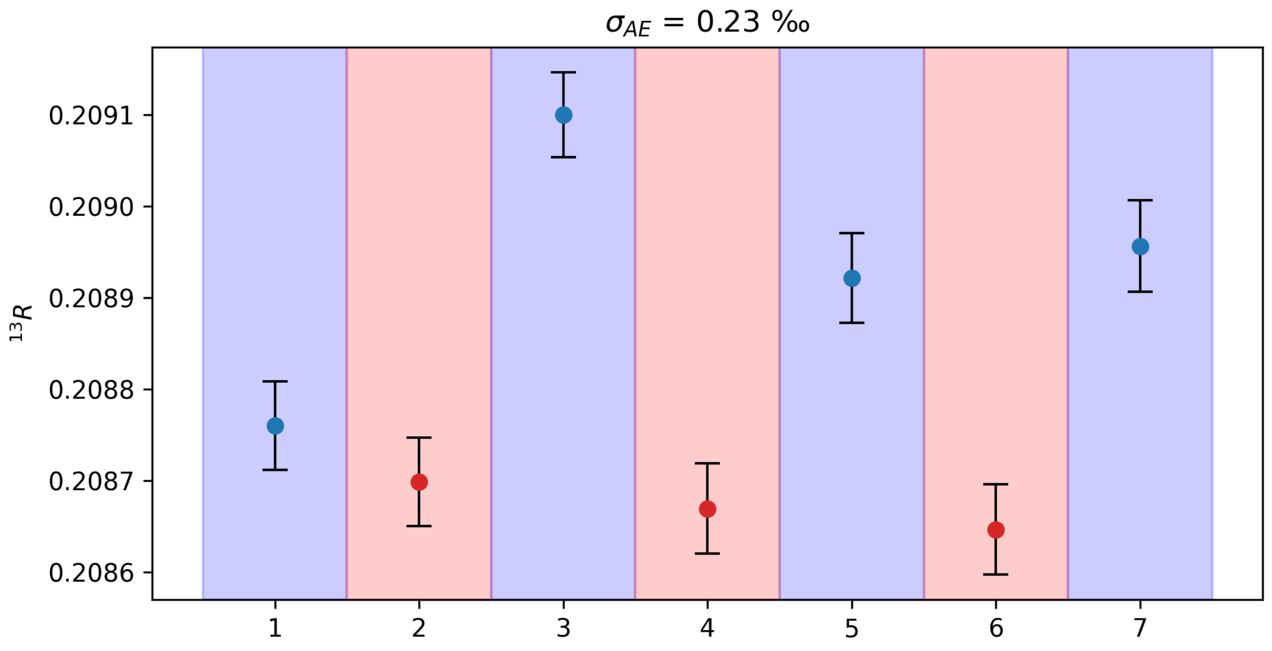

Cumulative Isotopic Ratio

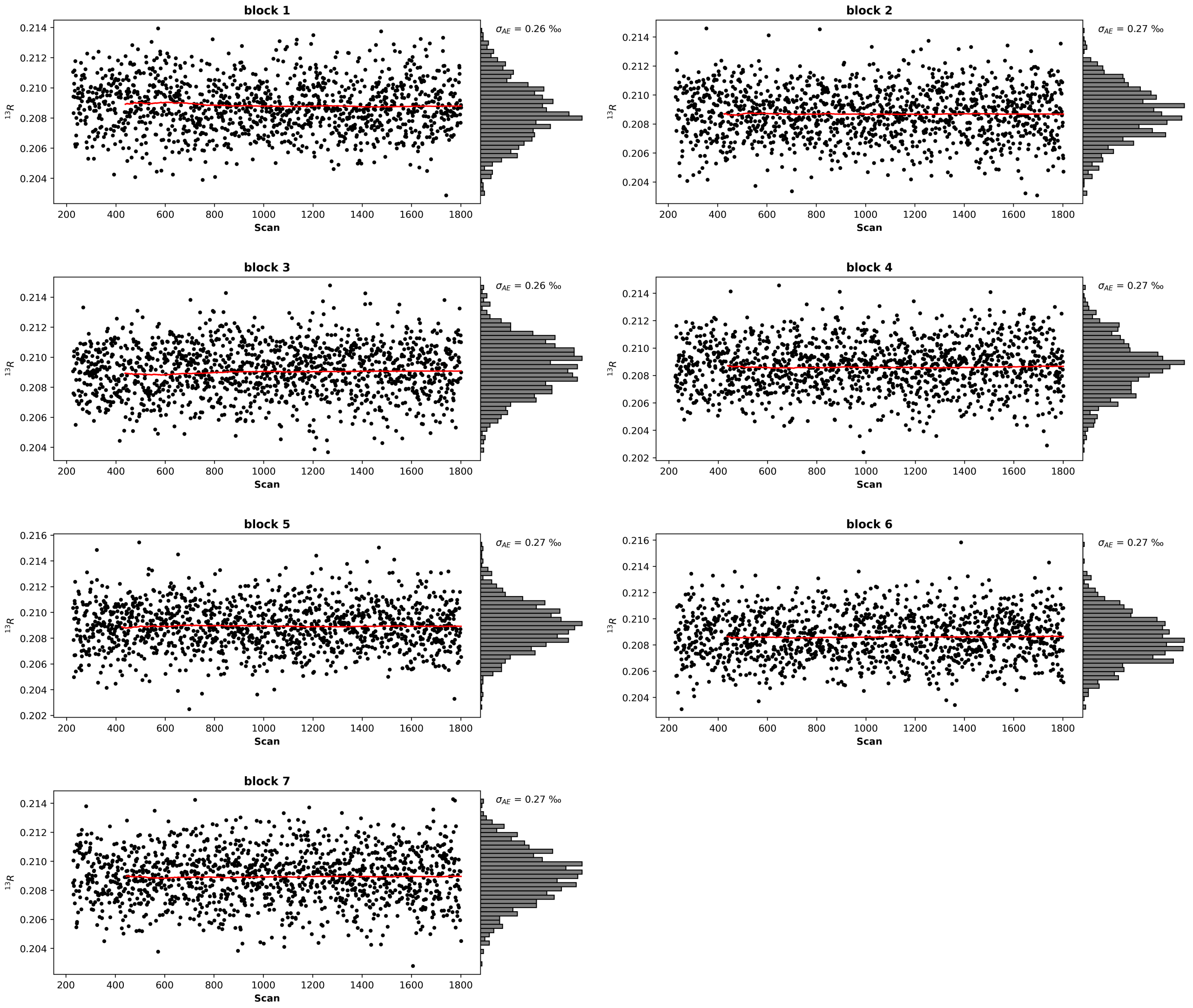

Acquisition Error and Shot-Noise

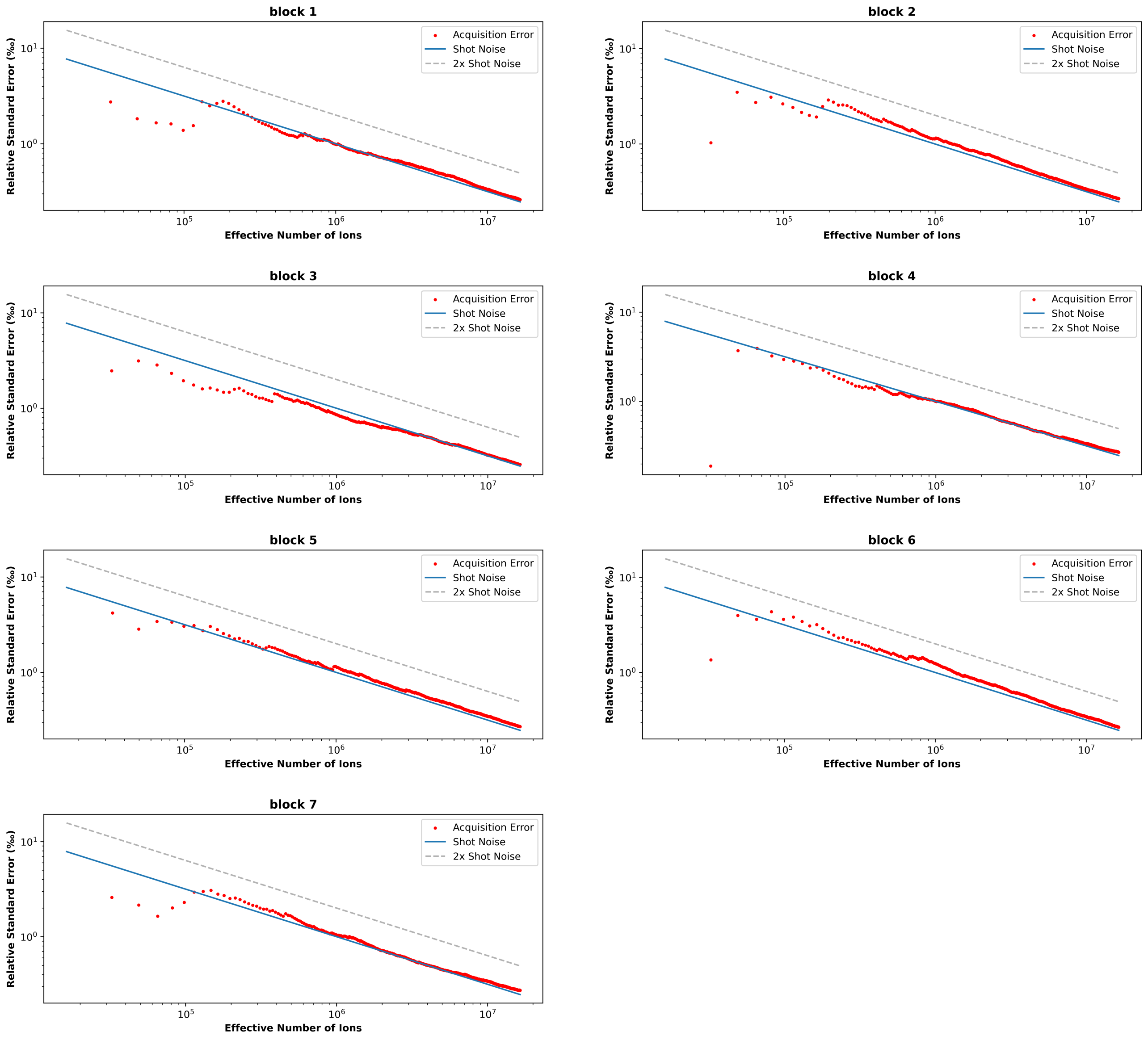

3. Delta Informations

Deltas were calculated by 'Average Of Neighboring Block Ratios'

3.1. 13C

Delta 13C was corrected by -27.80

Block	SEM	Delta corrected	Delta
2	0.23	-28.88	-1.11
4	0.24	-29.39	-1.63
6	0.24	-29.16	-1.40

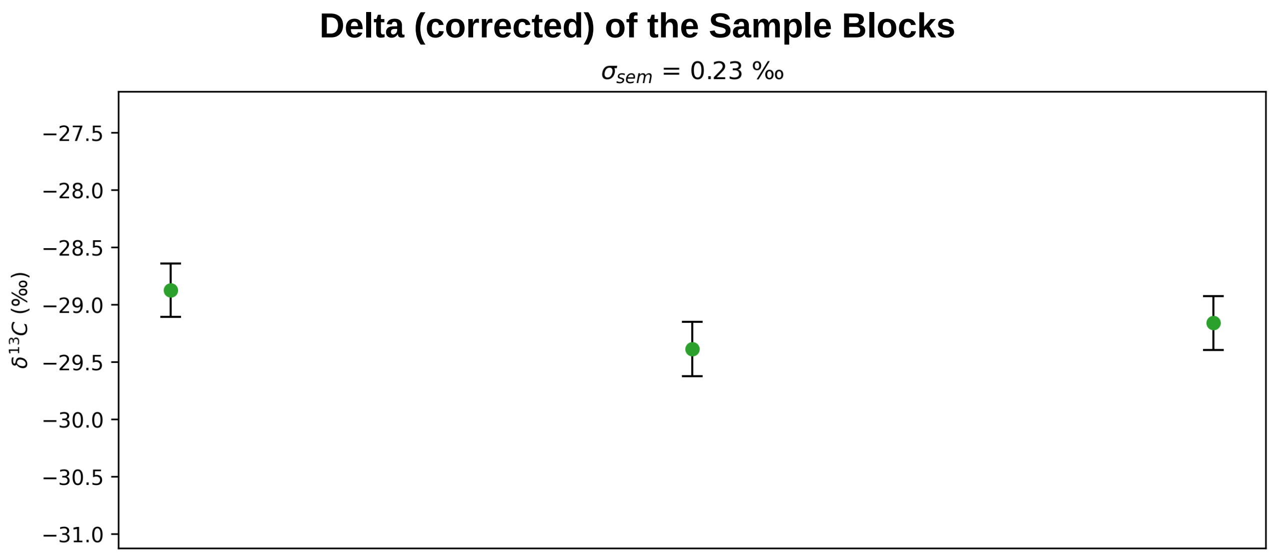

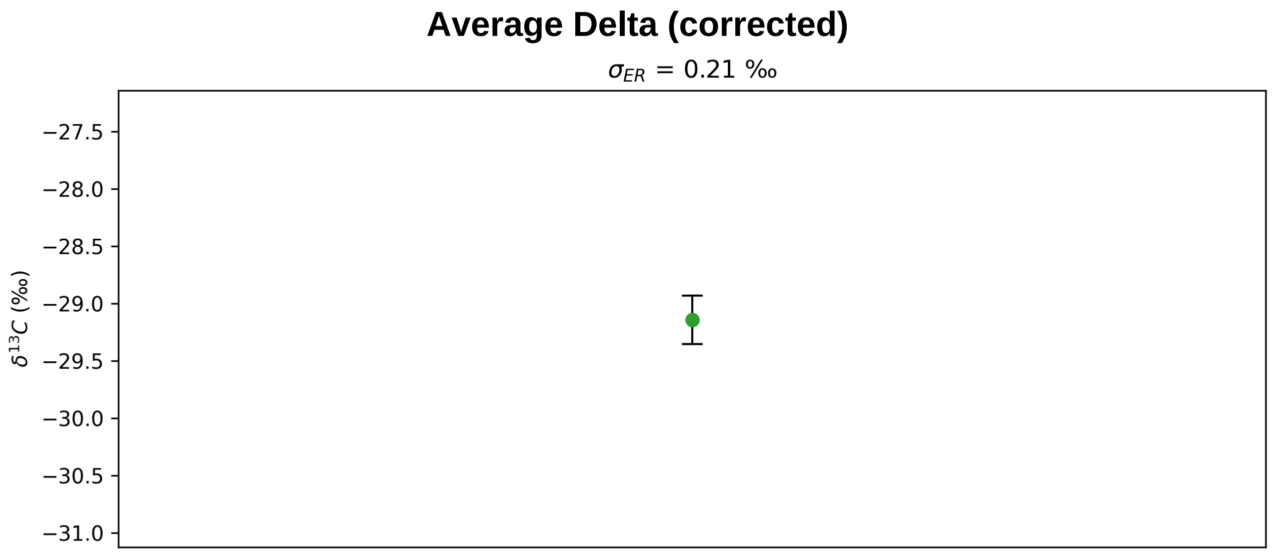

The final corrected average delta was -29.14 with a standard deviation of 0.21. Here the standard deviation is called reproducibility error.
