# Supplementary material for: Advancing Stable Isotope Analysis with Orbitrap-MS for Fatty Acid Methyl Esters and Complex Lipid Matrices
Source: J Am Soc Mass Spectrom. 2025 Jun 17;36(7):1527–35. doi: 10.1021/jasms.5c00092 (PMC12339014; doi:10.1021/jasms.5c00092)
Supplement: Supplementary file 2 [file js5c00092_si_002.zip › reports by IsotoPy Software/standards/Na+Standard6_DI.pdf]

Standard 6 - [M + Na]+
Isotope Analysis report from IsotoPy

Dual Inlet

1. Pre Processing

1.1. Block Time and Scan Information

Information about sample and standard block times and scans:

Block	Injected	Initial Time	End Time	Number of scans
1	standard	1	5	739
2	sample	6	10	720
3	standard	11	15	750
4	sample	16	20	745
5	standard	21	25	734
6	sample	26	30	727
7	standard	31	35	731

1.2. Outlier Removal

A total of 1158 scans were considered outliers and removed using the MAD method

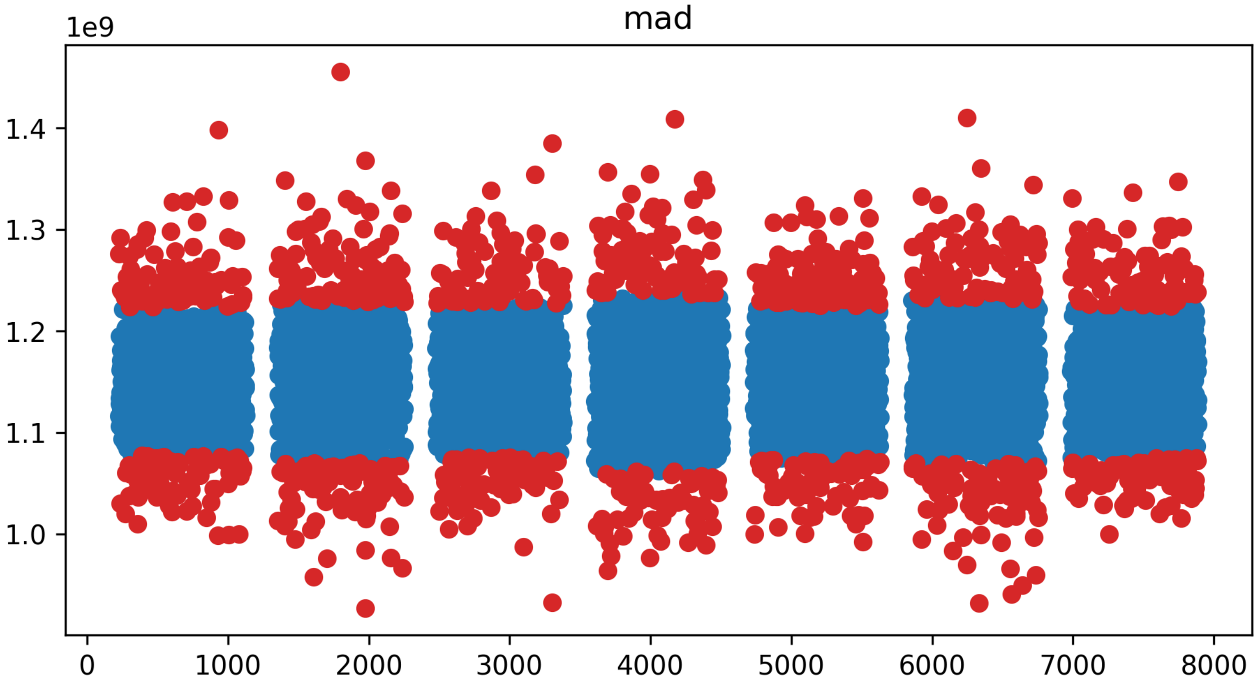

1.3. Total Ion Current (TIC)

TIC of all blocks

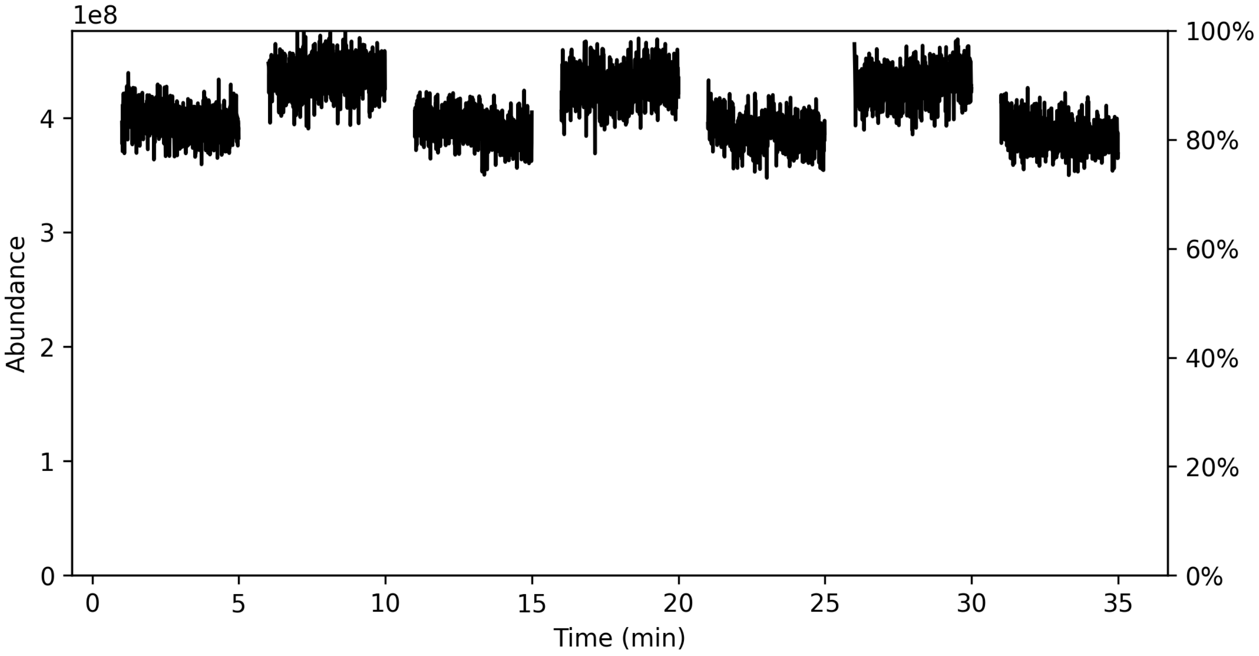

Block	TIC min	TIC max	TIC mean	RSD (%)
1	3.59e+08	4.39e+08	3.97e+08	3.20
2	3.90e+08	4.76e+08	4.38e+08	3.43
3	3.50e+08	4.24e+08	3.93e+08	3.16
4	3.69e+08	4.69e+08	4.30e+08	3.60
5	3.47e+08	4.33e+08	3.88e+08	3.36
6	3.85e+08	4.69e+08	4.30e+08	3.29
7	3.50e+08	4.26e+08	3.86e+08	3.42

2. Block Parameters

The Isotopic Ratio of the blocks were calculated by 'Mean'

2.1. 13C/M0

Block	Number of scans	Effective number of ions	Isotopic Ratio	STD	SEM	RSE
1	739	1.62e+07	0.209504	0.001414	0.000052	0.000248
2	720	1.58e+07	0.209319	0.001414	0.000053	0.000252
3	750	1.65e+07	0.209577	0.001413	0.000052	0.000246
4	745	1.64e+07	0.209316	0.001360	0.000050	0.000238
5	734	1.61e+07	0.209510	0.001416	0.000052	0.000249
6	727	1.60e+07	0.209469	0.001407	0.000052	0.000249
7	731	1.61e+07	0.209667	0.001330	0.000049	0.000235

Errors and Test Paramters

Block	Acquisition Error (permil)	Shot-Noise (permil)	AE/SN ratio	Shapiro Wilk (p_value)	D'Agostino (p_value)
1	0.248	0.248	0.999	0.724	0.923
2	0.252	0.251	1.001	0.817	0.941
3	0.246	0.246	0.999	0.878	0.973
4	0.238	0.247	0.963	0.892	0.957
5	0.249	0.249	1.001	0.902	0.833
6	0.249	0.250	0.996	0.336	0.290
7	0.235	0.249	0.940	0.235	0.656

Isotopic Ratio and Errors of the Blocks

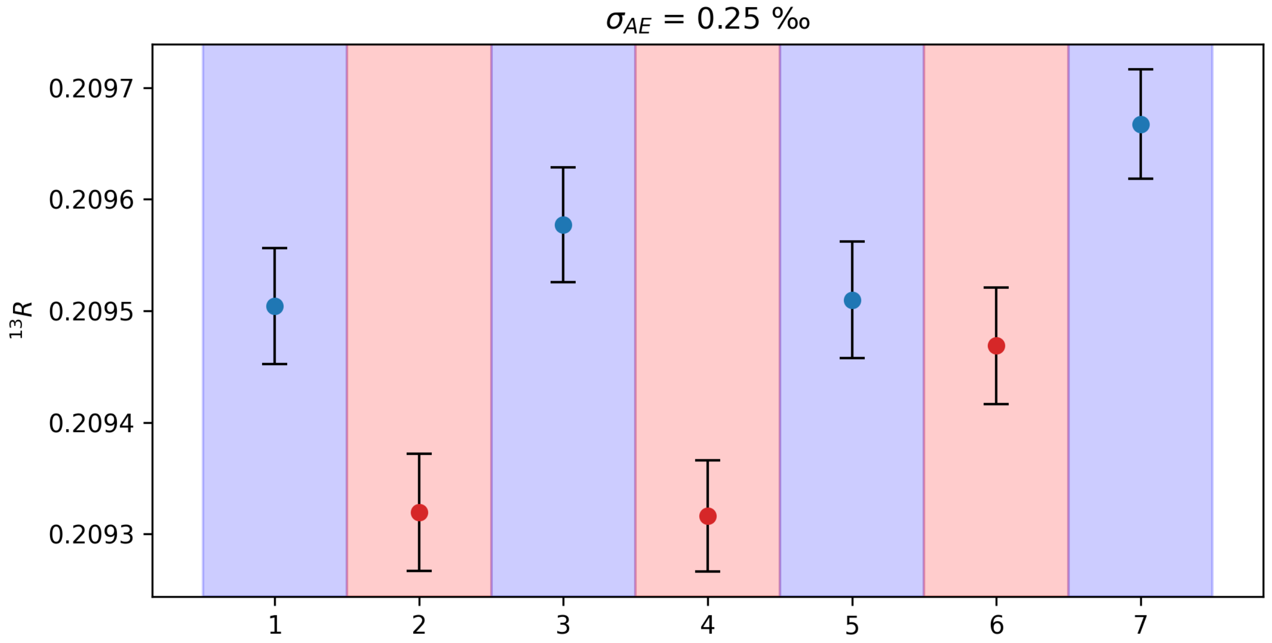

Cumulative Isotopic Ratio

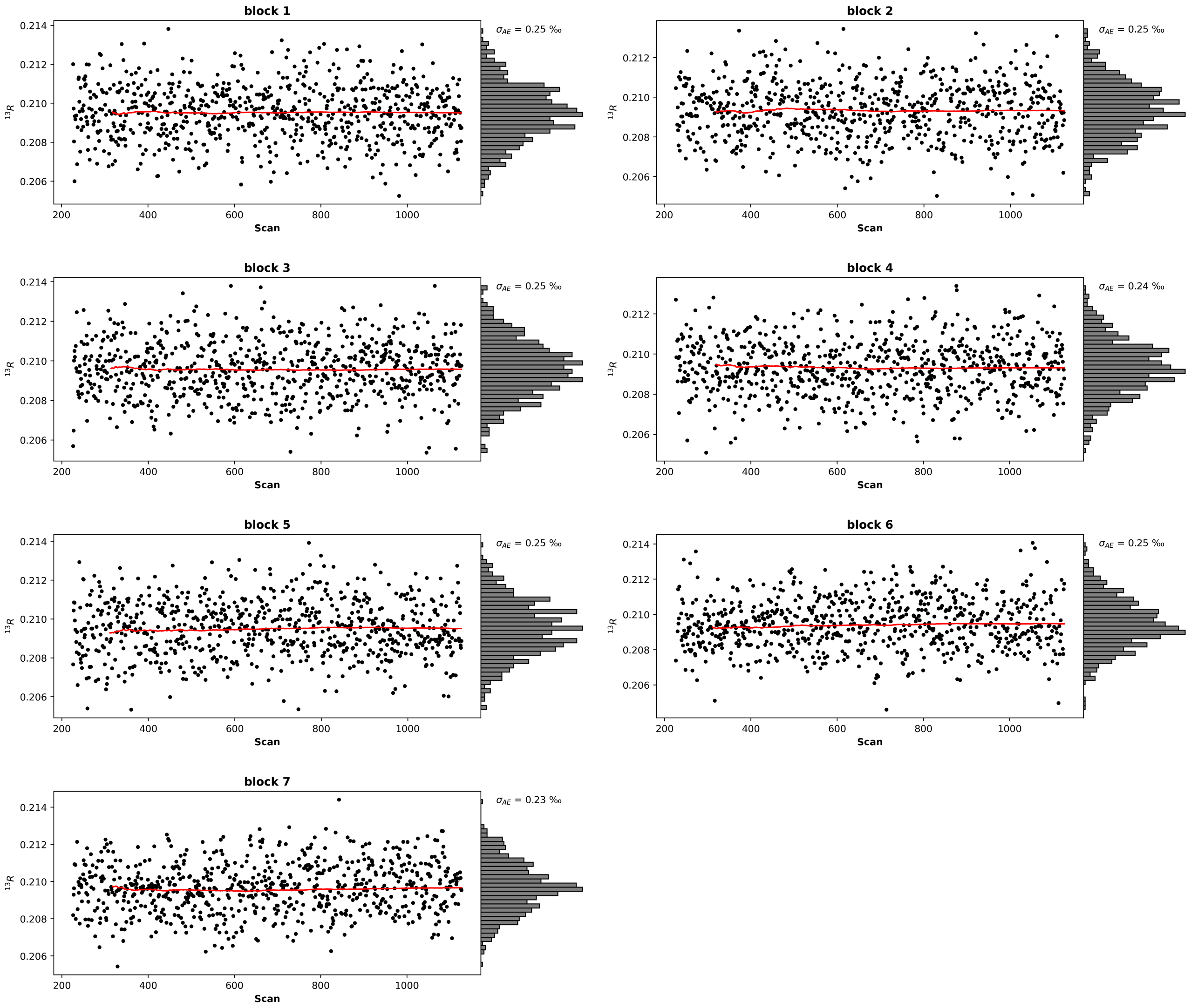

Acquisition Error and Shot-Noise

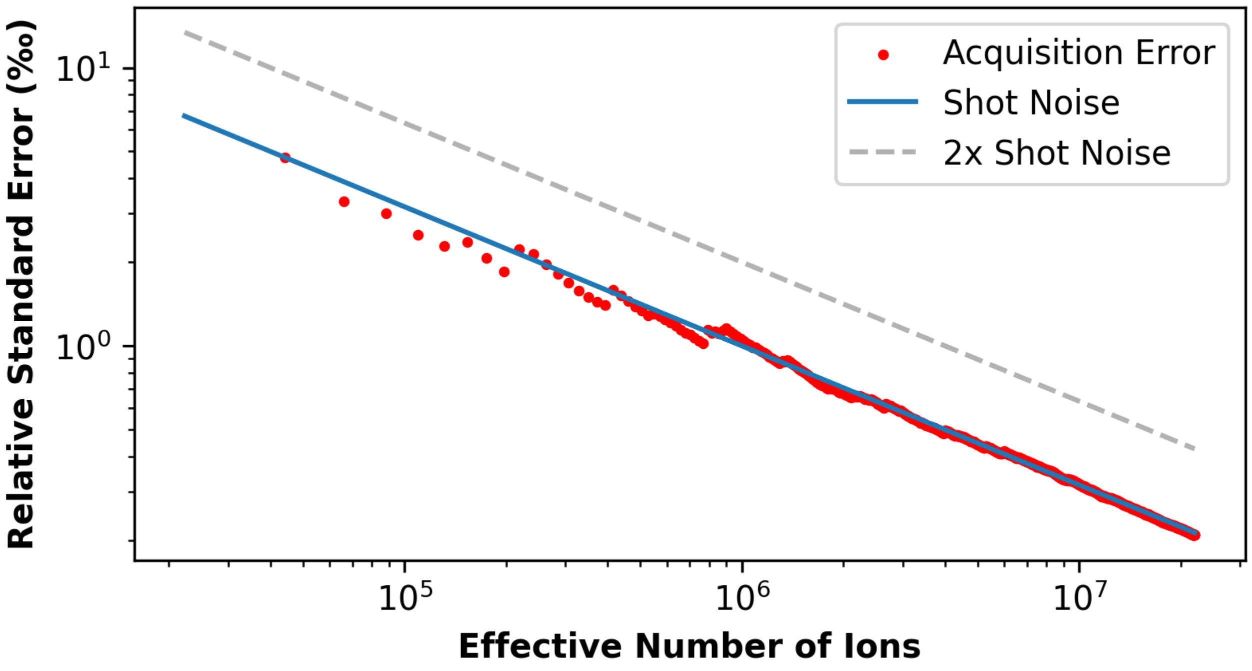

3. Delta Informations

Deltas were calculated by 'Average Of Neighboring Block Ratios'

3.1. 13C

Delta 13C was corrected by -27.80

Block	SEM	Delta corrected	Delta
2	0.25	-28.83	-1.06
4	0.24	-28.85	-1.08
6	0.25	-28.36	-0.57

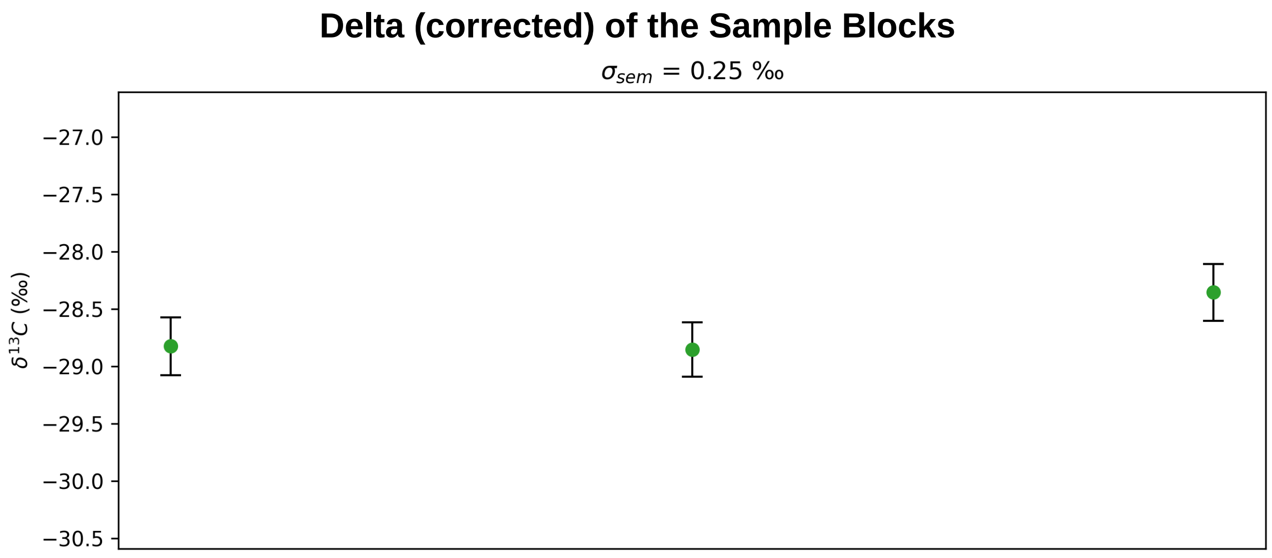

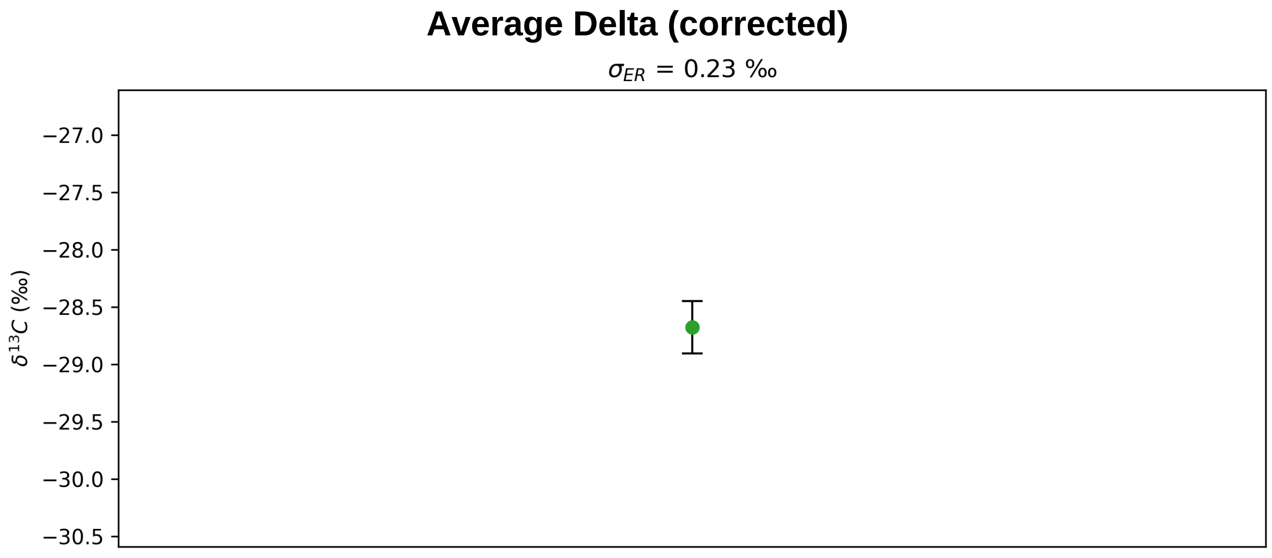

The final corrected average delta was -28.68 with a standard deviation of 0.23. Here the standard deviation is called reproducibility error.
